# CYP27A1 deficiency promoted osteoclast differentiation

**DOI:** 10.7717/peerj.15041

**Published:** 2023-03-03

**Authors:** Ziqi Fang, Guangdong Cheng, Mengting He, Yanliang Lin

**Affiliations:** 1 Department of Clinical Laboratory, Shandong Provincial Hospital, Shandong University, Jinan, China; 2 Department of Reproductive Medicine, Shandong Provincial Hospital Affiliated to Shandong First Medical University, Jinan, China; 3 Department of Critical Care Medicine, Shandong University of Traditional Chinese Medicine, Jinan, China

**Keywords:** CYP27A1, Osteoclasts, Bone loss, RNA sequencing, Expression profile

## Abstract

**Background:**

The elevating osteoclast differentiation can lead to an imbalance in bone homeostasis, which was responsible for bone loss and bone diseases, such as osteoporosis. Multiple pathways and molecules have been involved in osteoclast formation, but the role of CYP27A1 in osteoclast differentiation has never been explored.

**Methods:**

CYP27A1 deficient mice were constructed using CRISPR-Cas9 system. Osteoclast differentiation was detected by TRAP staining. Differentially expressed genes (DEGs) were identified using RNA-seq analysis and were confirmed by qRT-PCR and Western blot.

**Results:**

The results showed that CYP27A1 knockout (KO) promoted osteoclast differentiation and bone loss. The transcriptomic analysis revealed that CYP27A1 KO led to differential expression of multiple genes, including ELANE, LY6C2, S100A9, GM20708, BGN, SPARC, and COL1A2, which were confirmed by qRT-PCR and Western blot. Enrichment analysis indicated that these differential genes were significantly associated with osteogenesis-related pathways, such as PPAR signaling, IL-17 signaling, and PI3K/AKT signaling, which were confirmed by qRT-PCR and Western blot.

**Conclusions:**

These results suggested that CYP27A1 was involved in osteoclast differentiation, providing a novel therapeutic target for osteoclast-related diseases.

## Introduction

Osteoclasts are multinucleated cells differentiated from blood mononuclear progenitors, and mainly distribute on the bone tissue surface and bone marrow cavity. The coordination between osteoclasts and osteoblasts regulates the development and integrity of bone by actively absorbing organic matter and minerals in bone matrix (Seeman & Martin, 2019). Hyperfunction of osteoclast can lead to abnormal bone resorption, causing the bone degenerative diseases including osteoporosis and arthritis (Kular et al., 2012). Thus, it is necessary to explore the novel molecular mechanism of osteoclast differentiation.

CYP27A1 is a member of the cytochrome p450 superfamily. Cytochrome p450 proteins are monooxygenases that catalyze many reactions involved in drug metabolism as well as the synthesis of cholesterol, steroids, and other lipids. CYP27A1 mutations cause the cerebrotendinous xanthomatosis, a rare autosomal recessive inherited lipid storage disease. Mutations or deletion of CYP27A1 gene triggers the accumulation of cholestanol and cholesterol in serum and multiple tissues, enhancing various lesions (Freedman et al., 2019, Kuriyama et al., 1993). Osteolytic metastasis caused by osteoclast activation is the main reason of tumor-related death. Osteoclast hyperactivation is crucial in the osseous metastases, such as breast cancer and lung cancer, and therefore, osteoclast is considered as a therapeutic target for osseous metastases (Fornetti, Welm & Stewart, 2018). However, it remains unknown whether CYP27A1 affects osteoclast differentiation. Our results have evidenced that the conditioned medium of lung adenocarcinoma cells could induce the osteoclast differentiation, especially in the presence of 27-hydroxycholesterol (Zhang et al., 2019). Considering that CYP27A1 catalyzes the generation of 27-hydroxycholesterol, CYP27A1 might be implicated in the osteoclast differentiation.

In this study, we demonstrated that CYP27A1 knockout (KO) promoted the osteoclast differentiation of bone marrow monocytes. The transcriptomic results revealed that CYP27A1 KO caused the differential expression of multiple genes, including ELANE, LY6C2, S100A9, GM20708, BGN, SPARC, and COL1A2. These results provided novel targets for the therapy of osteoclast-related diseases.

## Materials and Methods

### Construction of CYP27A1 knockout mice

CYP27A1 knockout (KO) mice were generated using the CRISPR/Cas9 system. Firstly, one sgRNA targeting the near sequence of the inserted site was constructed and transcribed *in vitro*. The donor vector with the inserted fragment was designed and constructed *in vitro*. Subsequently, Cas9 mRNA, sgRNA and donor were co-injected into zygotes, followed by transplantation into the oviduct of pseudopregnant ICR female mice at 0.5 dpc. After 19–21 days of transplantation, F0 mice were birthed, all the offspring of ICR females (F0 mice) were identified by PCR and sequencing of tail DNA. Finally, positive F0 mice were crossed with wild type mice to construct heterozygous mice. Homozygous mice were generated by mating heterozygous mice. Cas9 endonucleases in introns 1–2 and 2–3 of the CYP27A1 gene were directly cleaved by sgRNAs, and double-strand break (DSBS) were generated. Such breaks were repaired by non-homologous end joining (NHEJ), and resulted in the disruption of CYP27A1. The sequences was used as follows: gRNA1, CAGTAGTCTCCCTTGTTGTA; gRNA2, GTCTCTGTCTACAGAACCGC; gRNA3, TACCAACACGTTGTGTCATT; gRNA4, AGAAGAGGCAGTGGCTGTTC.

### Animal experiments

Mice were obtained from the Nanjing Institute of Biomedicine, Nanjing University, and were maintained under the specific pathogen-free conditions in the animal facility. The water and food were supplemented every 3 days, and bedding was changed every 6 days. The animals were euthanized in the CO_2_ incubator. Eight-week-old male mice were used for all experiments. All experiments were approved by the Laboratory Animal Ethics Committee of Shandong Provincial Hospital (NSFC: No. 2022-050).

### Osteoclast differentiation

Eight-week-old wild type (WT) and homozygous C57/BL6 mice were used for the isolation of osteoclastic precursors. After euthanasia, the femur and tibia of mice were obtained and the bone marrow was rinsed with the α-MEM culture medium using a syringe. After filtration, bone marrow-derived monocytes (BMMs) were obtained by centrifugation. BMMs were cultured in the α-MEM complete culture medium in a 5% CO_2_ incubator at 37 °C and 100% humidity for 24 h. After lysing erythrocytes, BMMs were cultured in the α-MEM complete culture medium containing 30 ng/ml M-CSF for 48 h. Subsequently, the medium was replaced with the complete medium containing 30 ng/ml M-CSF and 50 ng/ml RANKL every 2 days. Osteoclast formation was observed after 5 days of culture. Cells containing three or more nuclei stained by TRAP were considered as multinucleated osteoclasts.

### TRAP staining

BMMs were cultured in 96-well plates, and were differentiated into osteoclasts, followed by immobilization for 20 min using 4% paraformaldehyde. The cells were subsequently incubated with TRAP solution at 37 °C in the dark for 45 min, and were stained with hematoxylin solution for 5 min. The staining cells were captured using an olympus microscope (IX53).

### Construction and sequencing process of the mRNA gene library

Osteoclasts were lysed in the trizol for RNA extraction. The integrity and quantification of RNA were determined using the agarose gel electrophoresis and Nanodrop 2000 (Thermo Fisher, Waltham, MA, USA), respectively. Samples were divided into the control and KO groups with three repetitions. The library was obtained through a series of operational modifications.

The original image data obtained from sequencing were converted into sequence data by base calling, which were called raw data or raw reads. In order to ensure data quality, the original data should be filtered before information analysis to reduce the interference brought by invalid data. First, the raw reads were quality controlled using FASTQ (Chen et al., 2018) to filter low-quality data and obtain clean reads. StringTie was used to assemble the reads of RNAseq into transcripts (Pertea et al., 2015). From the alignment results of HISAT2, we reconstructed the transcripts using StringTie and calculated the expression levels of all genes in each sample using RSEM (Li & Dewey, 2011). The expression information was analyzed using DESeq2 software (Love, Huber & Anders, 2014). Based on the results of the differential analysis, significant differential genes were screened (FDR < 0.05 and |log2FC | > 1).

### Enrichment analysis of differentially expressed genes (DEGs)

Differential genes were mapped to each term of the GO database (http://www.geneontology.org/), and the number of differential genes for each term was calculated to obtain the number of differential genes in the list of differential genes with a certain GO function (Yu et al., 2012). A hypergeometric test was then applied to find out the GO entries that were significantly enriched in the differential genes as compared to the background. The KEGG is the main public database about the pathways. Pathway significance enrichment analysis was performed in KEGG pathways units using a hypergeometric test to identify pathway significantly enriched in differential genes compared to the entire background (Ogata et al., 1999). The most important biochemical metabolic pathways and signal transduction pathways were identified by pathway significance enrichment.

### Analysis of the protein interaction networks

The STRING protein interaction database (http://string-db.org) was mainly used to analyze the interaction network among differential genes (Szklarczyk et al., 2015). For the species included in the database, the differential gene set was extracted from the database to build the interaction network map using cytoscape (Shannon et al., 2003). For the species not included in the database, the sequences in the target gene set were first blastx aligned to the protein sequence of the reference species included in the string database, and the protein interaction relationship of the reference species on the alignment was used to build the interaction network.

### Determination of the key genes

GeneMANIA (http://www.genemania.org) is an online analysis tool, and can search many large, publicly available biological datasets to find related genes, which include protein-protein, protein-DNA and genetic interactions, pathways, reactions, gene and protein expression data, protein domains and phenotypic screening profiles (Franz et al., 2018). Data is regularly updated. Using the gene tool, the interaction of the targeted genes with other genes was found.

### Quantitative real-time PCR

Total RNA was extracted from cells in the CYP27A1 WT and KO groups (*n* = 3 per group) using Trizol reagent. The concentration and purity of total RNA were detected using a Nanodrop 2000 spectrophotometer (Thermo Fisher, Waltham, MA, USA). Reverse transcription was preformed to synthesize cDNA using Hiscript®iii Rt Supermix for qRT-PCR (RC323-01; Nanjing Vazyme Biotech Co, Nanjing, China). The cDNA was used as the template to amplify targeted sequences with specific primers using the Chamq Universal SYBR qRT-PCR Master Mix (Q711-02; Nanjing Vazyme Biotech Co, Nanjing, China). The results were analyzed by the 2^−ΔΔCT^ method (Livak & Schmittgen, 2001). The sequences of the eight primers were shown in Table 1.

**Table 1:** Primer sequences of the eight hub genes.

Gene	5′–3′
Mus-GAPDH-182 F	TGTCTCCTGCGACTTCAACA
Mus-GAPDH-182 R	GGTGGTCCAGGGTTTCTTACT
Mus-CYP27A1-79F	GTACTCAGGAGACCATCGGC
Mus-CYP27A1-79R	GCCCATGTCAGTGTGTTGGA
Mus-BGN-150F	TCCCTGAGACCCTGAACGAA
Mus-BGN-150R	GCAGAAAACTCAGGCTCCCA
Mus-S100A9-154 F	TCAGATGGAGCGCAGCATAA
Mus-S100A9-154 R	GGCTTCATTTCTCTTCTCTTTCTTC
Mus-COL1A2-79F	GGTCCAAGAGGAGAACGTGG
Mus-COL1A2-79R	TGGGACCTCGGCTTCCAATA
Mus-ELANE-82F	TCAGAGATTGTTGGTGGCCG
Mus-ELANE-82R	AGAAATGACCTCCACGCCTC
Mus-SPARC-195F	ACCTGGACTACATCGGACCA
Mus-SPARC-195R	CCAGGCGCTTCTCATTCTCA
Mus-GM20708-135F	TGGCGAAGGGGAGAAACTGG
Mus-GM20708-135R	GGGAAAGCTGTAAGACAAGGCA
Mus-LY6C2-106F	TGGACAGTACTCACGCTACA
Mus-LY6C2-106R	GGCACTCCATAGCACTCGTA

### Western blot analysis

The total protein was extracted using RIPA lysis buffer. The protein concentration was measured using BCA method. Equal amount of protein was subjected to 10% SDS-PAGE electrophoresis, and was then transferred to PVDF membrane, followed by blocking with 5% nonfat milk for 2 h at room temperature. After rinsed in TBST buffer, the protein was labeled by the corresponding primary antibody at 4 °C overnight and HRP-coupled secondary antibody at room temperature for 1 h. The protein bands were visualized using the enhanced chemiluminescence method. The antibodies used were as follows: anti-MMP9 (1:1,000, 502095; Zen-Bio Inc, Durham, NC, USA); anti-NFATc1 (1:1,000, 251865; Zen-Bio Inc, Durham, NC, USA); anti-c-Fos (1:1,000, A0236; ABclonal Technology, Woburn, MA, USA); anti-TRAP (1:1,000, 382344; Zen-Bio Inc, Durham, NC, USA); anti-CTSK (1:1,000, A1782; ABclonal Technology, Woburn, MA, USA); anti-PPAR-γ (1:1,000, 340844; Zen-Bio Inc, Durham, NC, USA); anti-AKT (1:1,000, R23412; Zen-Bio Inc, Durham, NC, USA); anti-IL7 (1:1,000, A1650; ABclonal Technology, Woburn, MA, USA); anti-COL1A2 (1:1,000, 380760; Zen-Bio Inc, Durham, NC, USA); anti-BGN (1:1,000, 16409-1-AP, ptgcn); anti-SPARC (1:1,000, 121259; Zen-Bio Inc, Durham, NC, USA); anti-CYP27A1 (1:1,000, A1982; ABclonal Technology, Woburn, MA, USA); anti-ELANE (1:1,000, A13015; ABclonal Technology, Woburn, MA, USA); anti-S100A9 (1:1,000, R25648; Zen-Bio Inc, Durham, NC, USA); anti-LY6C2 (1:1,000, ET1702-78; HUABIO, New Boston, MI, USA); anti-GAPDH (1:1,000, 10494-1-AP, ptgcn); anti-actin (1:1,000, 81115-1-RR, ptgcn).

### Statistical analysis

The statistical analysis was performed using GraphPad Prism software (version 8.3.0, San Diego, CA). Data are presented as the mean ± standard deviation (SD). The difference between two groups was analyzed using Student’s t-test. *p* < 0.05 was considered statistically significant.

## Results

### CYP27A1 KO promoted osteoclast differentiation

**Figure 1:**
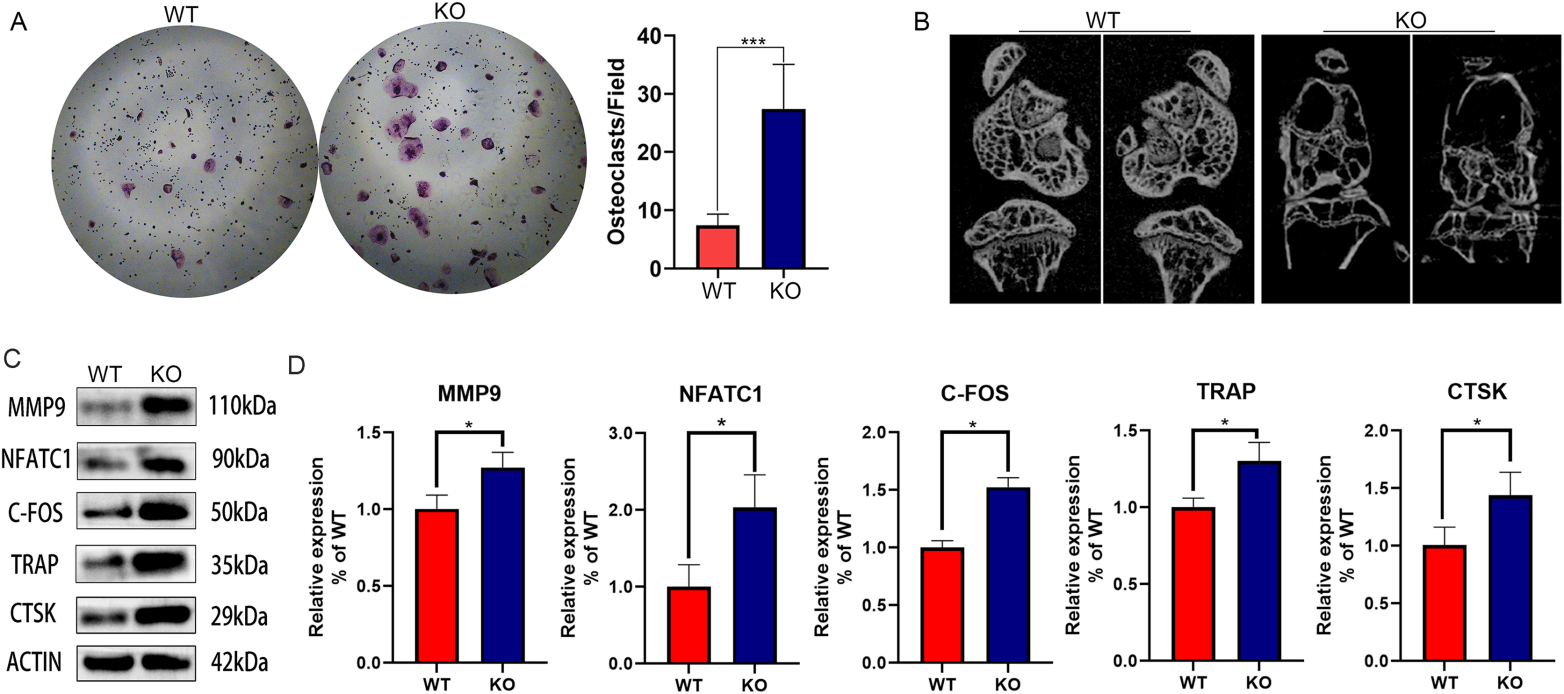
The effect of CYP27A1 knockout (KO) on the osteoclast differentiation, bone loss and gene expression. (A) CYP27A1 KO promoted the osteoclast differentiation. Osteoclast differentiation was determined by TRAP staining. ***, *p* < 0.001. (B) CYP27A1 KO facilitated bone loss. *In vivo* imaging of bones was captured by micro-CT. (C) CYP27A1 KO promoted the expression of MMP9, NFATC1, C-FOS, TRAP and CTSK. The protein expression was determined using Western blot analysis. (D) The mRNA expression was determined using qRT-PCR. *, *p* < 0.05.

Since our previous findings have confirmed that 27HC-stimulated conditional medium of lung adenocarcinoma cells promotes osteoclast differentiation, and CYP27A1 is a synthetase of 27HC, we speculated that CYP27A1 KO might affect osteoclast differentiation. However, contrary to expectations, CYP27A1 KO also significantly promoted osteoclast formation (Fig. 1A). The *in vivo* imaging of bones from CYP27A1 WT and KO mice was performed using micro-CT. The results showed that CYP27A1 KO led to bone loss (Fig. 1B). Further investigation demonstrated that CYP27A1 KO promoted the expression of osteoclast-related genes, including MMP9, NFATc1, c-Fos, TRAP and CTSK (Figs. 1C and 1D).

### Identification of DEGs between CYP27A1 WT and KO osteoclasts

**Figure 2:**
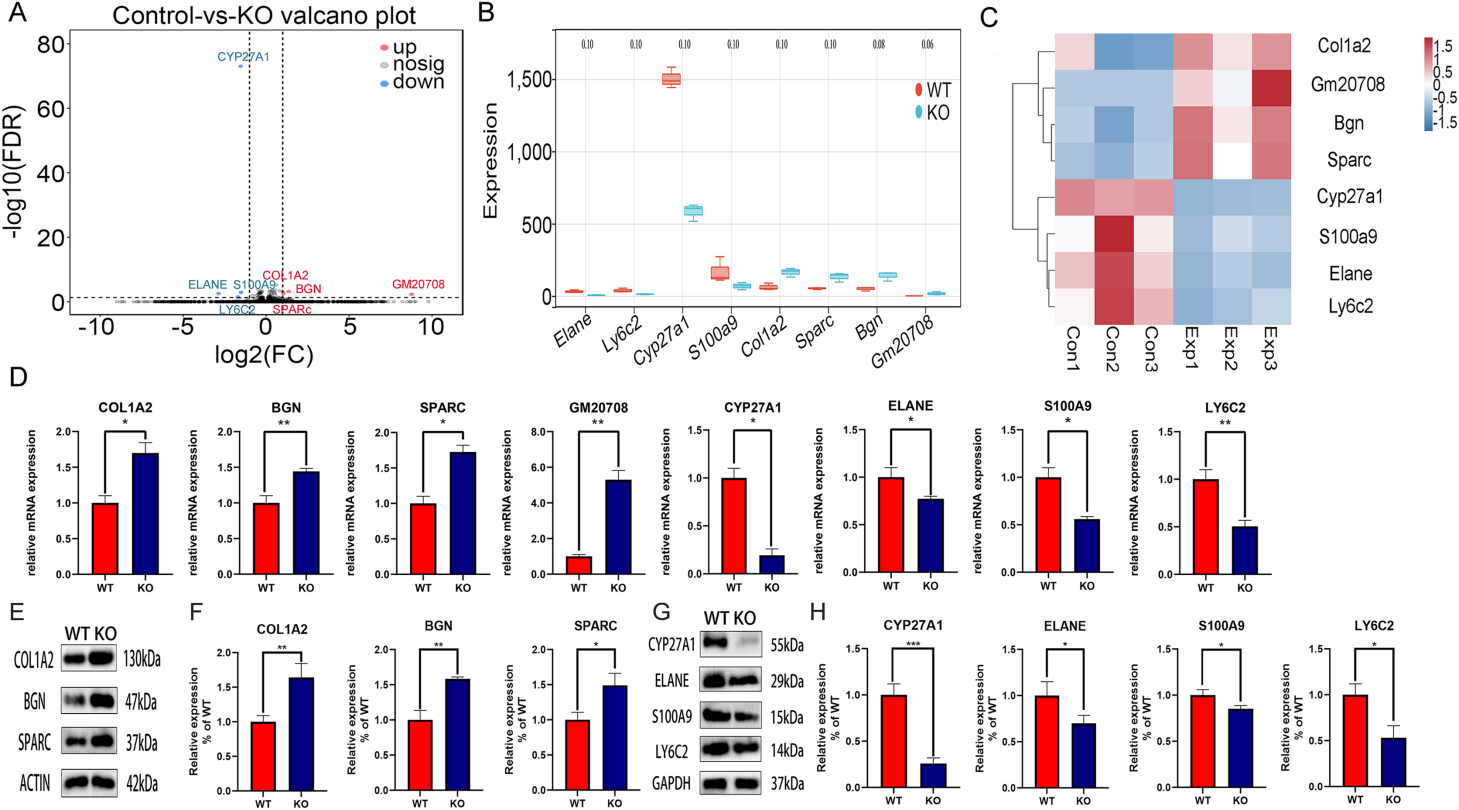
Transcriptomic analysis of the differentially expressed genes (DEGs) between CYP27A1 KO and WT osteoclasts. (A) Volcano map of the differential gene expression between CYP27A1 WT and KO groups. (B) Box plots of the differential genes between CYP27A1 WT and KO groups. (C) Heatmap of the differential gene clusters between CYP27A1 WT and KO groups. (D) The effect of CYP27A1 KO on the mRNA expression of COL1A2, BGN, SPARC, GM20708, CYP27A1, ELANE, S100A9 and LY6C2. The mRNA expression was determined using qRT-PCR. *, *p* < 0.05; **, *p* < 0.01. (E) CYP27A1 KO promoted the expression of COL1A2, BGN and SPARC. The protein expression was determined using Western blot analysis. (F) The relative expression of COL1A2, BGN and SPARC. *, *p* < 0.05; **, *p* < 0.01. (G) CYP27A1 KO inhibited the expression of ELANE, S100A9 and LY6C2. The protein expression was determined using Western blot analysis. (H) The relative expression of ELANE, S100A9 and LY6C2. *, *p* < 0.05; ***, *p* < 0.001.

To explore the molecular mechanism of CYP27A1 KO in osteoclast differentiation, RNA sequencing was performed to identify the DEGs between the CYP27A1 WT and KO groups. The DEGs between the two cohorts were shown in the form of volcano plot (Fig. 2A). Figure 2B illustrated eight top DEGs with four up-regulated and four down-regulated genes, including ELANE, LY6C2, S100A9, GM20708, BGN, SPARC, and COL1A2. The expression patterns of eight DEGs were hierarchically clustered, which was presented in the heatmap (Fig. 2C). These genes with similar expression patterns might have common functions or participate in common metabolic pathways and signaling pathways. The expression of hub genes was verified by qRT–PCR and Western blot. As shown in Fig. 2, all eight hub genes exhibited a similar tendency among the RNA-sequencing analysis, qRT–PCR and Western blot analysis. The mRNA and protein expression of COL1A2, BGN, SPARC and GM20708 was up-regulated in the osteoclasts from CYP27A1 KO group (Figs. 2D–2F), while the mRNA and protein levels of CYP27A1, ELANE, S100A9 and LY6C2 were down-regulated in CYP27A1 KO group (Figs. 2G and 2H), which indicated the accuracy and reliability of the RNA-sequencing results. The detailed descriptions of the eight genes were listed in Table 2. Transcriptome data have been uploaded to the NCBI database (BioProject ID, PRJNA847887).

**Table 2:** Description of the eight hub genes.

Gene	Full name	Synonyms	Gene biotype	Location	Function
CYP27A1	Cytochrome p450 family 27 subfamily A member 1	CYP27	Protein coding	Chromosome 1	This protein is important for overall cholesterol homeostasis.
ELANE	Elastase, neutrophil expressed	NE ELA2	Protein coding	Chromosome 10	This gene encodes a member of the chymotrypsin-like family of serine protease enzymes that hydrolyzes a broad range of proteins ubstrates including elastin.
LY6C2	Lymphocyte antigen 6 complex	LY-6C2	Protein coding	Chromosome 15	This gene enables acetylcholine receptor binding activity and acetylcholine receptor inhibitor activity.
S100A9	s100 calcium binding protein A9	GAGB L1Ag	Protein coding	Chromosome 3	Involved in peptidyl-cysteine S-nitrosylation, positive regulation of blood coagulation and regulation of translation.
COL1A2	Collagen, type L, alpha 2	COLA2	Protein coding	Chromosome 6	This gene encodes the alpha-2 subunit of the fibriI-forming type I collagen.
SPARC	Secreted acidic cysteine rich glycoprotein	BM-40	Protein coding	Chromosome 11	This acts upstream of or within several processes, including bone development, cellular response to growth factor stimulus and pigmentation.
BGN	Biglycan	BG PGI	Protein coding	Chromosome X	This gene encodes a small, leucine-rich repeat proteoglycan that plays important roles in bone mineralization and connective tissue etabolism.
GM20708	LOC6608141	GM20708-PA	Protein coding	Chromosome 2R	The function of the gene is unclear.

### Functional enrichment analysis of the DEGs

**Figure 3:**
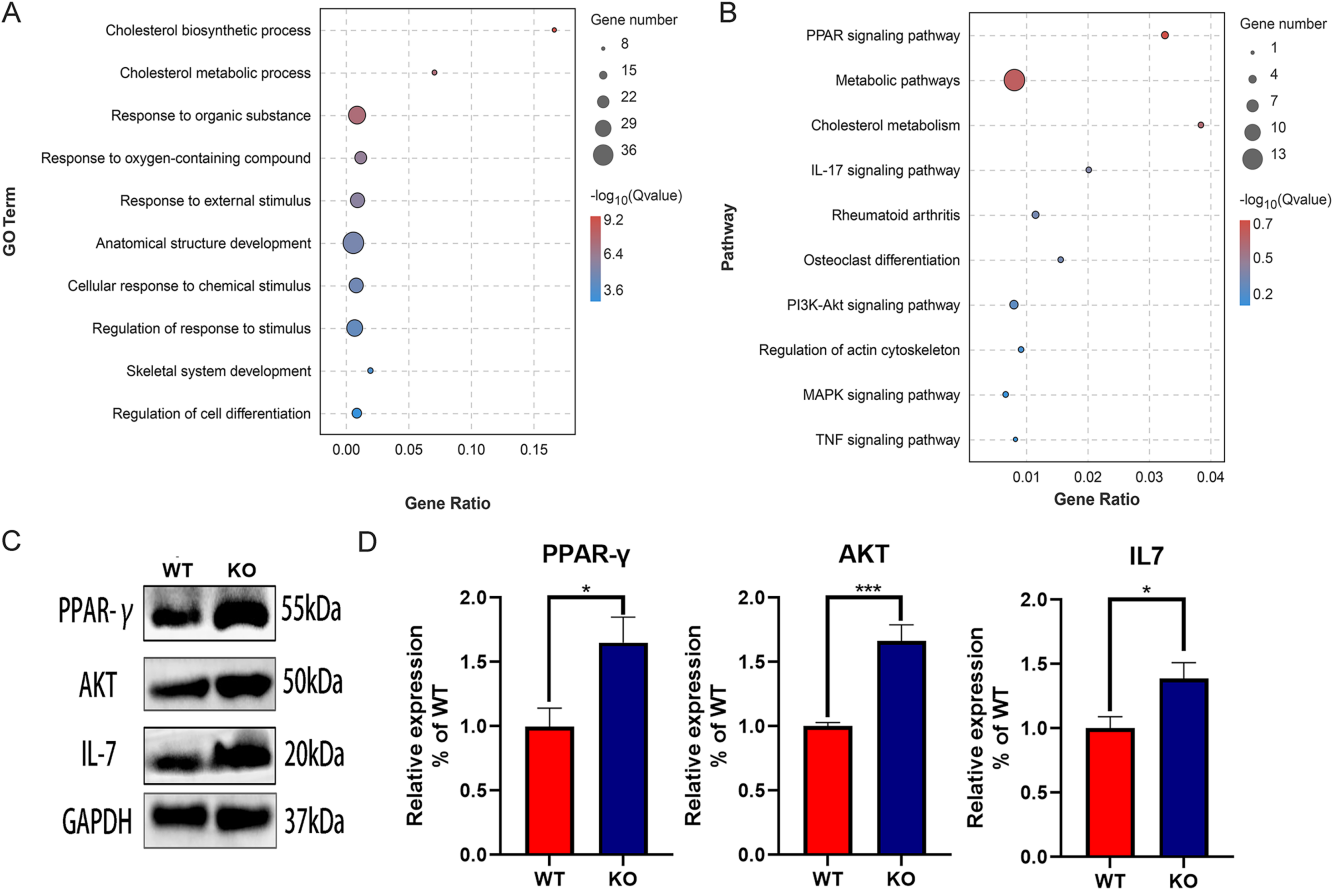
Functional enrichment analysis of the DEGs. (A) GO enrichment analysis of DEGs. (B) KEGG enrichment analysis of of DEGs. (C) CYP27A1 KO promoted the expression of PPAR-γ, AKT and IL-7. The protein expression was determined using Western blot analysis. (D) The relative expression of PPAR-γ, AKT and IL-7. *, *p* < 0.05; ***, *p* < 0.001.

The function of DEGs was explored by GO and KEGG enrichment analysis. The GO enrichment analysis showed that the DEGs were significantly associated with cholesterol biosynthetic process, cholesterol metabolic process, and response to organic substance, especially with skeletal system development and regulation of cell differentiation, suggesting the key role of CYP27A1 in osteoclast differentiation (Fig. 3A). The KEGG enrichment analysis revealed that the DEGs were mainly involved in metabolic pathways, PPAR signaling pathway, cholesterol metabolism, IL-17 signaling pathway and the PI3K/AKT signaling pathway, especially osteoclast-related pathways, including rheumatoid arthritis, osteoclast differentiation and the TNF signaling pathway (Fig. 3B). Also, the expression of major enrichment pathway related genes was verified by Western blot analysis (Figs. 3C and 3D).

### PPI protein network and gene network

**Figure 4:**
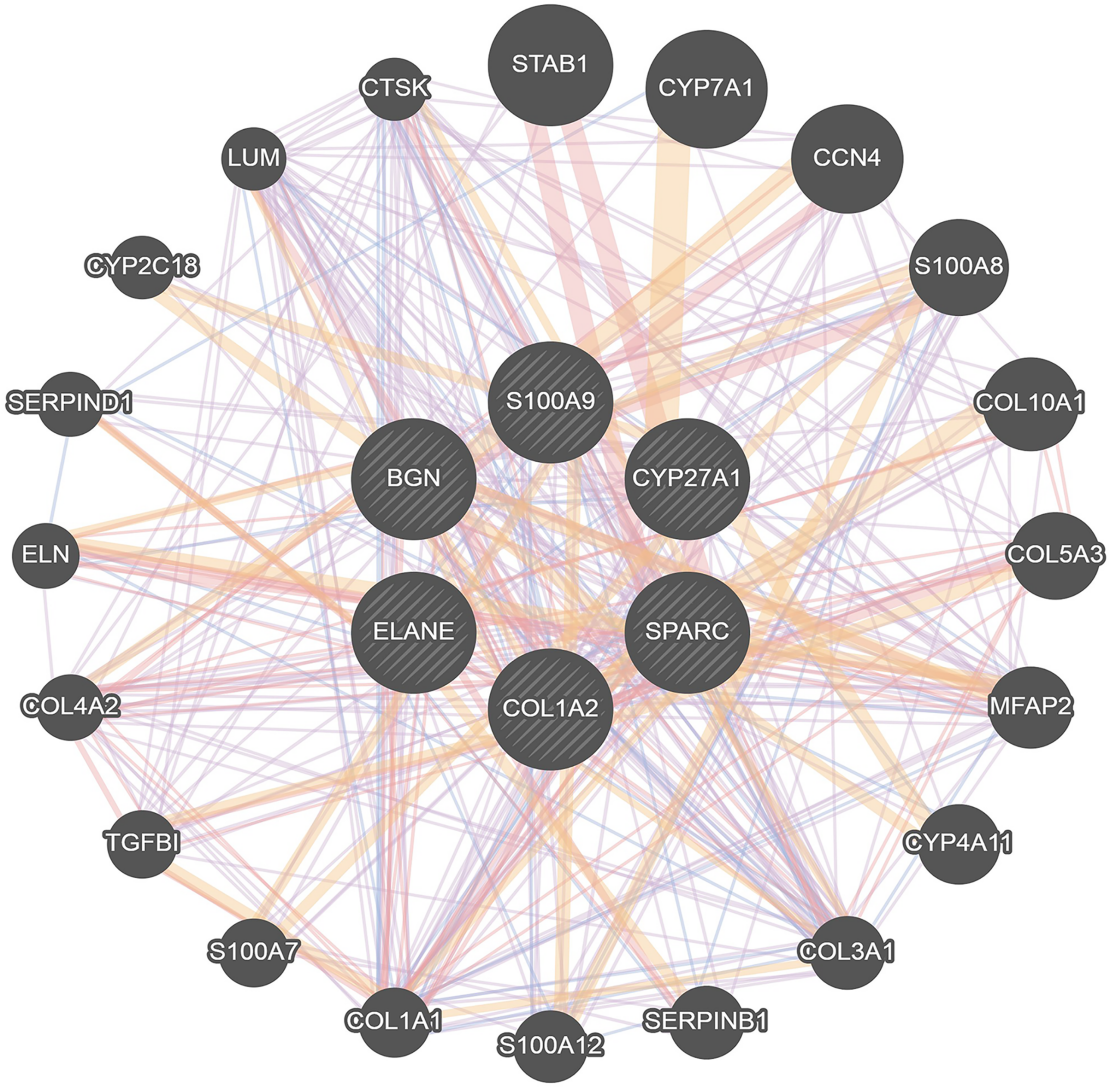
Related genes interacted with central hubs.

To further investigate the interaction of DEGs, a visual PPI network was created using cytoscape (Fig. S1). These key genes interacted and influenced each other, forming a protein interaction network centered on BGN that is closely linked to the osteoclasts differentiation. To further investigate the interaction and function of these core genes, GeneMANIA was applied to construct the gene network that used BGN, S100A9, CYP27A1, SPARC, COL1A2 and ELANE as the central hub with 19 associated genes (Fig. 4). Among these genes, most genes were closely linked to the osteoclasts differentiation. These results suggested the correlation of CYP27A1 to the osteoclast differentiation.

## Conclusions

Osteoclasts are multinucleated cells that differentiate from blood mononuclear precursor cells, which regulate skeletal development and integrity by actively resorbing minerals and interacting with osteoblasts. Overactive osteoclasts cause bone degenerative diseases, including osteoporosis, arthritis and bone metastasis of tumors. Thus, it is necessary to explore the molecular mechanism of osteoclast differentiation. 27-hydroxycholesterol (27HC), an oxysterol from cholesterol catalyzed by CYP27A1, affects the population of immature stem and progenitor cells, which impairs immature HSPC population (Woo et al., 2022). CYP27A1 deficient mice show the thinner trabeculae than the control mice due to the insufficient 27HC, indicating the key role of CYP27A1 in the bone composition and architecture (DuSell et al., 2010). In this study, we provided the evidences that CYP27A1 KO promoted the osteoclast differentiation and bone loss. There were little findings about the role of CYP27A1 in osteoclast differentiation. Our previous results have demonstrated that the condition medium of lung adenocacinoma cells stimulated by 27-HC promotes the osteoclast differentiation. We also evidence that exposure of lung adenocacinoma cells to 27HC triggers the secretion of IL-6 and FGF2 (Li et al., 2022), which is likely to explain the positive role of 27HC in the osteoclast differentiation, suggesting that other signaling pathways should be involved in osteoclast differentiation mediated by CYP27A1 deficiency. In addition, bone remodeling is regulated by the interaction between osteoclasts and osteoblasts. Therefore, it is necessary to investigate whether CYP27A1 deficiency affects the osteoblast differentiation.

To explore the signaling pathways mediated by CYP27A1 deficiency, transcriptomic analysis was performed. Compared to control groups, CYP27A1 KO led to the differential expression of eight genes, including four up-regulated genes and four down-regulated genes, respectively. GO and KEGG enrichment analysis suggested that these differentially expressed genes were significantly associated with cholesterol metabolism, the PPAR signaling pathway, the IL-17 signaling pathway, rheumatoid arthritis, osteoclast differentiation and the PI3K/AKT signaling pathway. Cholesterol activates ERRα and enhances the interaction between ERRα and PGC1β, reversing the inhibitory effect of carnosic acid on the osteoclastogenesis (Zheng et al., 2020). Activation of PPAR-γ exacerbates the osteoclastogenesis in a receptor-dependent manner by positively regulating c-Fos expression (Wan, Chong & Evans, 2007). IL-17 facilitates bone loss and osteoclast differentiation by increasing the expression of c-Fos and NFATc1 (Tan et al., 2021). In addition, PI3K/AKT signaling has been widely involved in the osteoclast differentiation (Ma et al., 2021).

CYP27A1 KO resulted in eight top DEGs, including ELANE, LY6C2, S100A9, GM20708, BGN, SPARC, and COL1A2, among which BGN acted as a central hub. The BGN has been involved in the pathogenesis of atherosclerosis by retaining lipoproteins and LDL (Scuruchi et al., 2020). The BGN also directly collaborates with TNF-α and RANKL to control the bone mass and osteoclastogenesis (Kram et al., 2017). The SPARC is required for the calcification of collagen in bone, and is also involved in maintaining cell shape (Durkin et al., 2022). COL1A2 encodes pro-alpha2 chain of type I collagen, and its mutations cause the osteogenesis imperfecta (Makitie et al., 2019). S100A9 is the calprotectin of the S-100 protein family with anti-inflammatory and antimicrobial properties (Rochette et al., 2022), which hampers osteoclast differentiation by reducing RANK expression (Di Ceglie et al., 2019). ELANE plays key roles in degenerative and inflammatory diseases through the proteolysis of collagen-IV and elastin, and has been implicated in osteoarthritis (Zhang et al., 2020). These results suggested that CYP27A1 KO might facilitate osteoclast differentiation *via* the above signaling pathways.

In summary, we evidenced that CYP27A1 deficiency promoted the osteoclast differentiation and bone loss. A comprehensive transcriptomic analysis was performed to determine the differentially expressed genes between CYP27A1 WT and KO mice, and identified the probable signaling pathways mediated CYP27A1. These results suggested that CYP27A1 was a novel therapeutic target for osteoclast-related diseases.

## Supplemental Information

10.7717/peerj.15041/supp-1 Supplemental Information 1 PPI network with cytoscape.

10.7717/peerj.15041/supp-2 Supplemental Information 2 Raw data.

10.7717/peerj.15041/supp-3 Supplemental Information 3 GAPDH and IL7.

10.7717/peerj.15041/supp-4 Supplemental Information 4 AKT and PPAR-γ.

10.7717/peerj.15041/supp-5 Supplemental Information 5 ACTIN and CTSK.

10.7717/peerj.15041/supp-6 Supplemental Information 6 MMP9 and NFATC1.

10.7717/peerj.15041/supp-7 Supplemental Information 7 C-FOS and TRAP.

10.7717/peerj.15041/supp-8 Supplemental Information 8 ACTIN and GAPDH.

10.7717/peerj.15041/supp-9 Supplemental Information 9 BGN and SPARC.

10.7717/peerj.15041/supp-10 Supplemental Information 10 COL1A2 and CYP27A1.

10.7717/peerj.15041/supp-11 Supplemental Information 11 ELANE and S100A9.

10.7717/peerj.15041/supp-12 Supplemental Information 12 LY6C2.

10.7717/peerj.15041/supp-13 Supplemental Information 13 Vertebrate animals approval documentation.

## Additional Information and Declarations

### Data Availability

The following information was supplied regarding data availability: The transcriptome data is available at NCBI GenBank: PRJNA847887. The qRT-PCR and Western blot data are available in the Supplemental Files.
