# Supplementary figures and images for: CYP27A1 deficiency promoted osteoclast differentiation (part 1 of 3)
Source: PeerJ. 2023 Mar 3;11:e15041. doi: 10.7717/peerj.15041 (PMC9987298; doi:10.7717/peerj.15041)

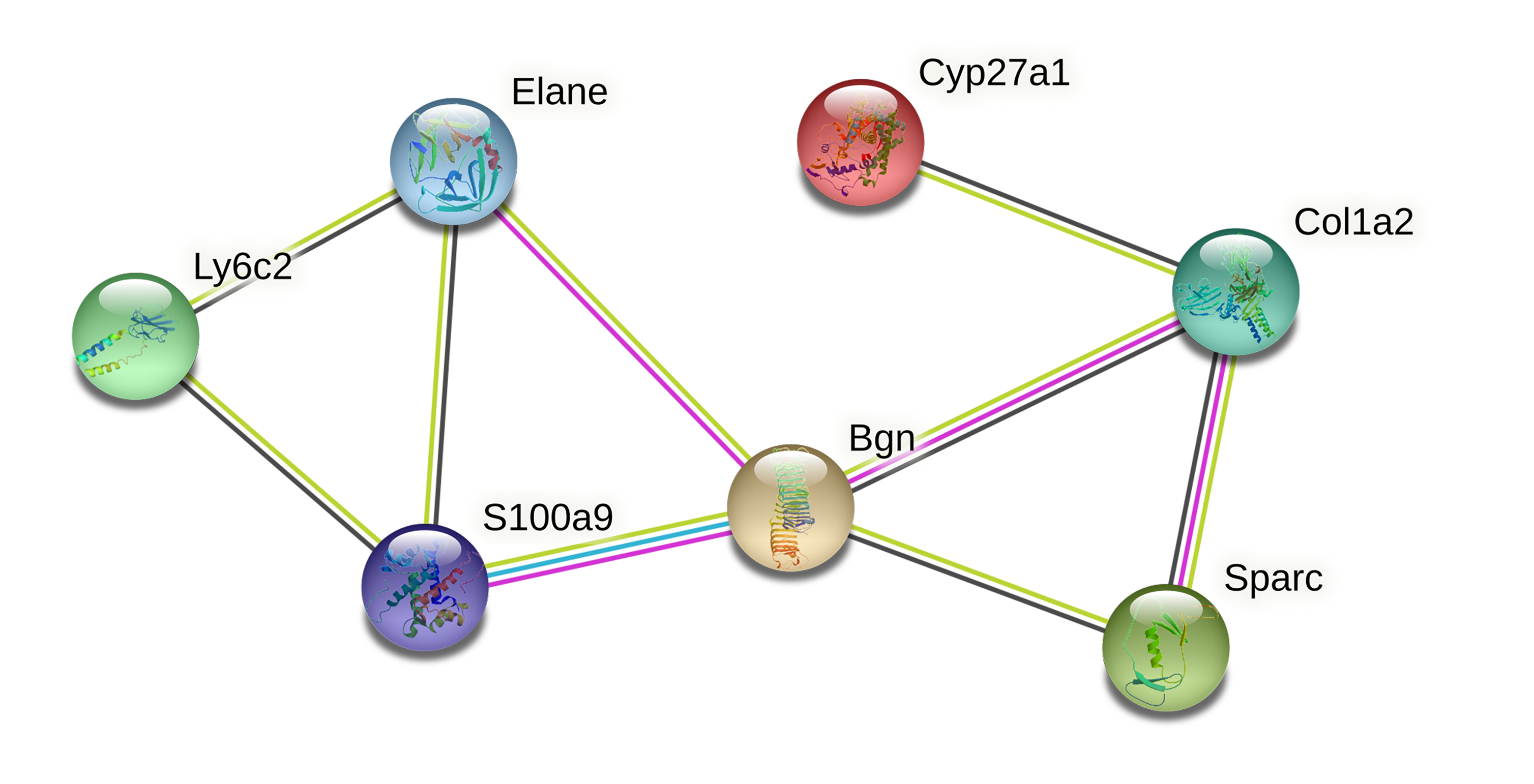

Supplement: Supplemental Information 1 [file peerj-11-15041-s001.png]

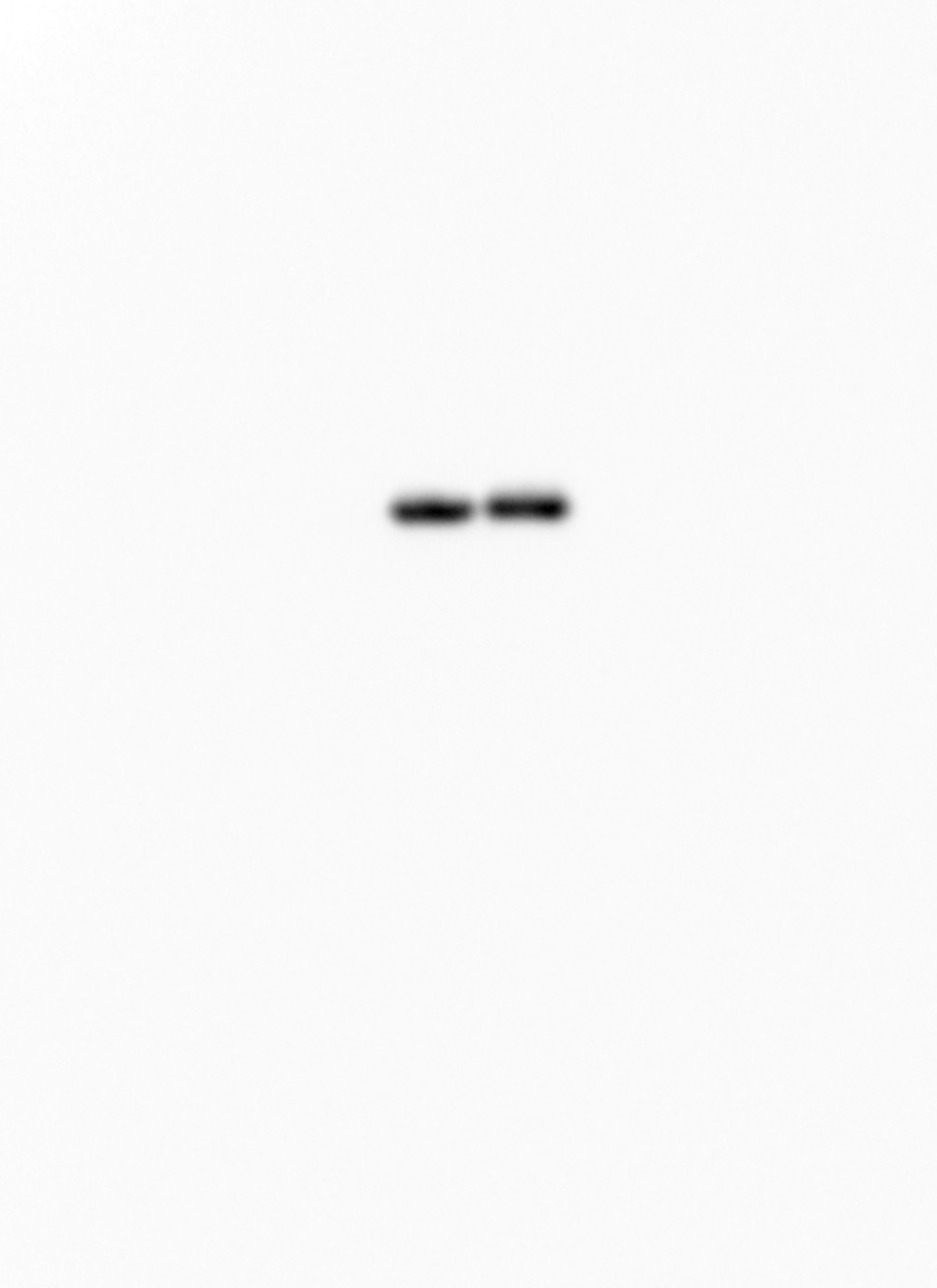

Supplement: Supplemental Information 3 [file peerj-11-15041-s003.zip › Enrichment related gens-raw data1/GAPDH/GAPDH-1/GAPDH-1-1.tif]

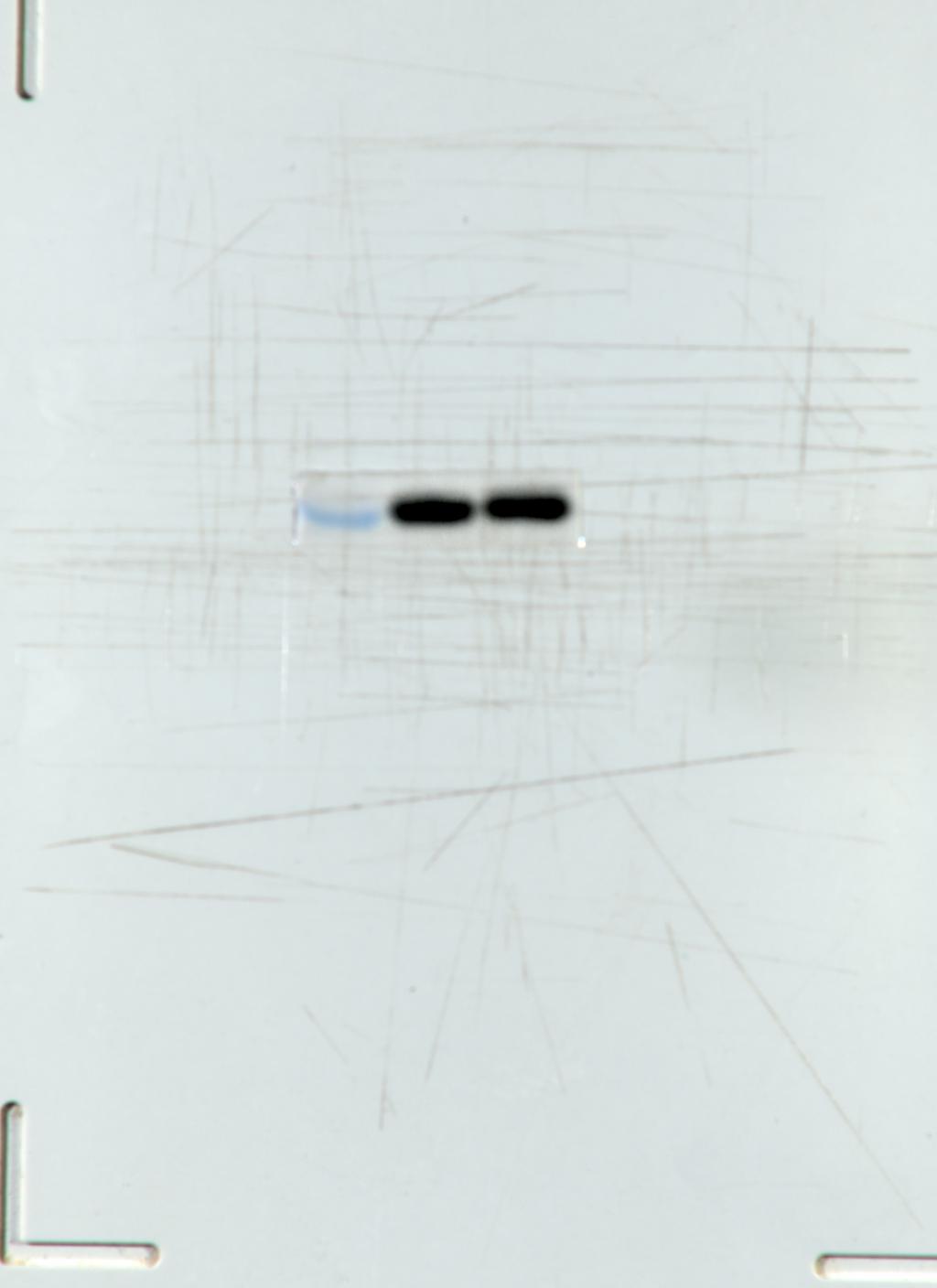

Supplement: Supplemental Information 3 [file peerj-11-15041-s003.zip › Enrichment related gens-raw data1/GAPDH/GAPDH-1/GAPDH-1-2.jpg]

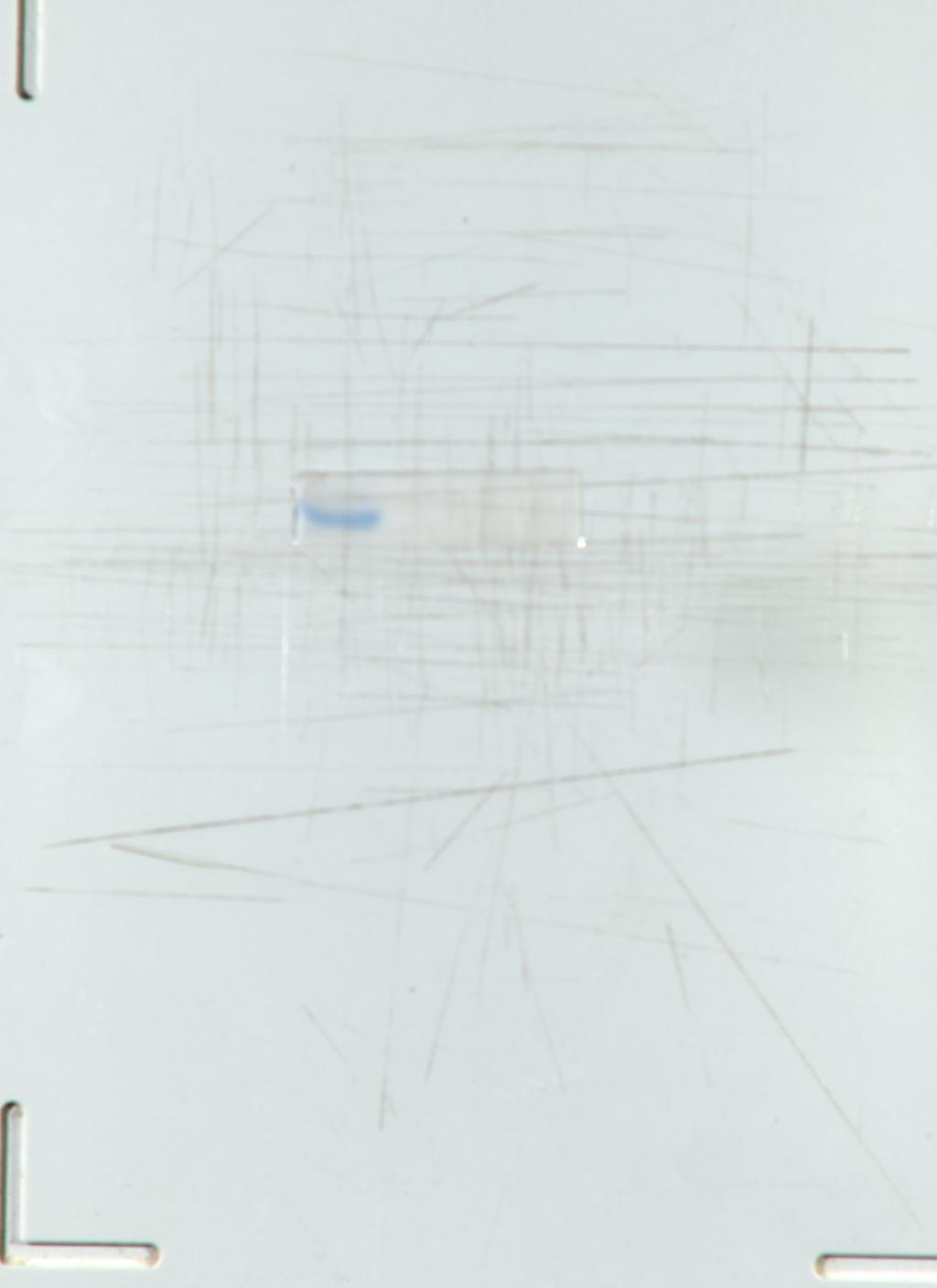

Supplement: Supplemental Information 3 [file peerj-11-15041-s003.zip › Enrichment related gens-raw data1/GAPDH/GAPDH-1/GAPDH-1-3.jpg]

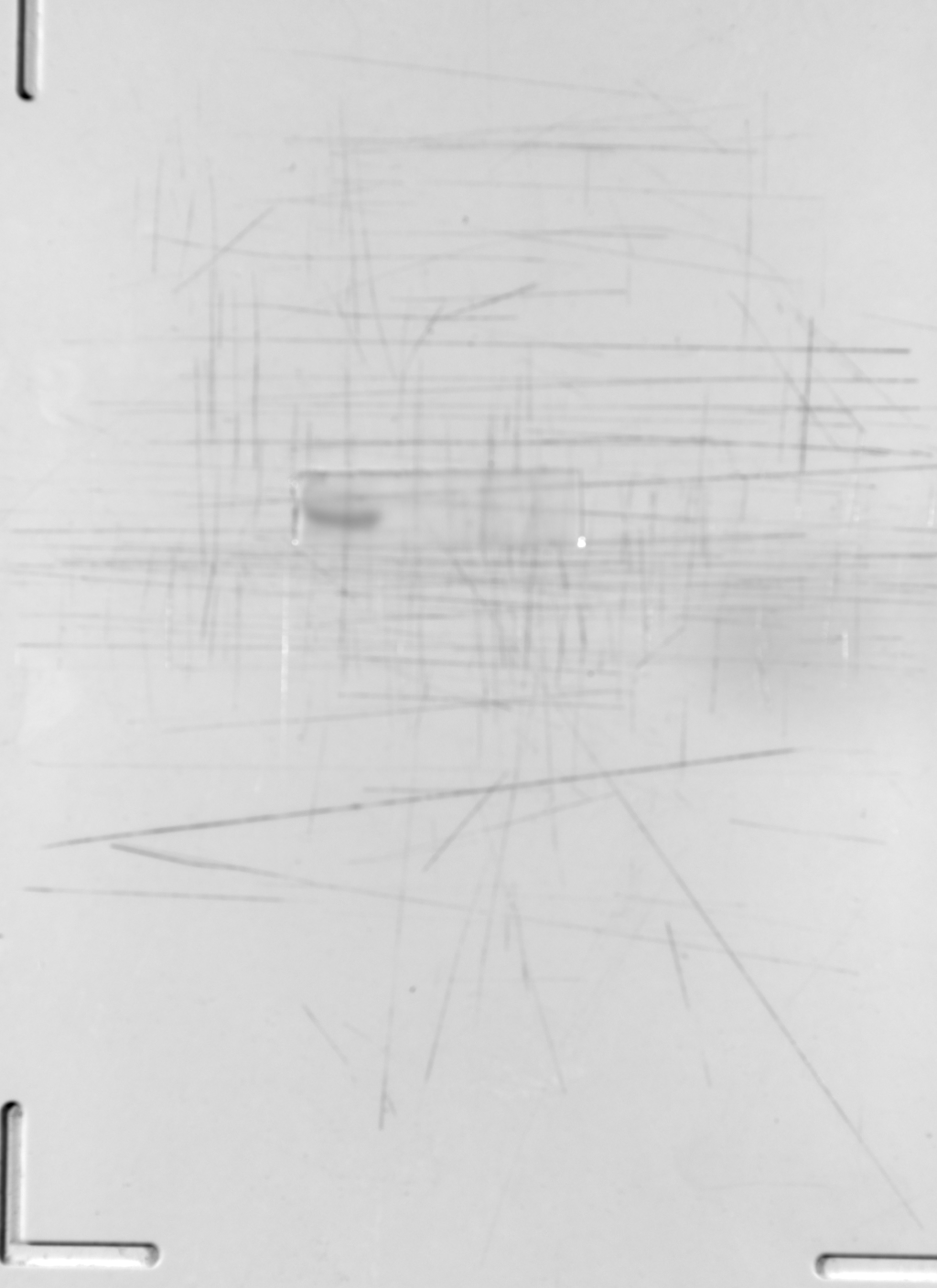

Supplement: Supplemental Information 3 [file peerj-11-15041-s003.zip › Enrichment related gens-raw data1/GAPDH/GAPDH-1/GAPDH-1-4.tif]

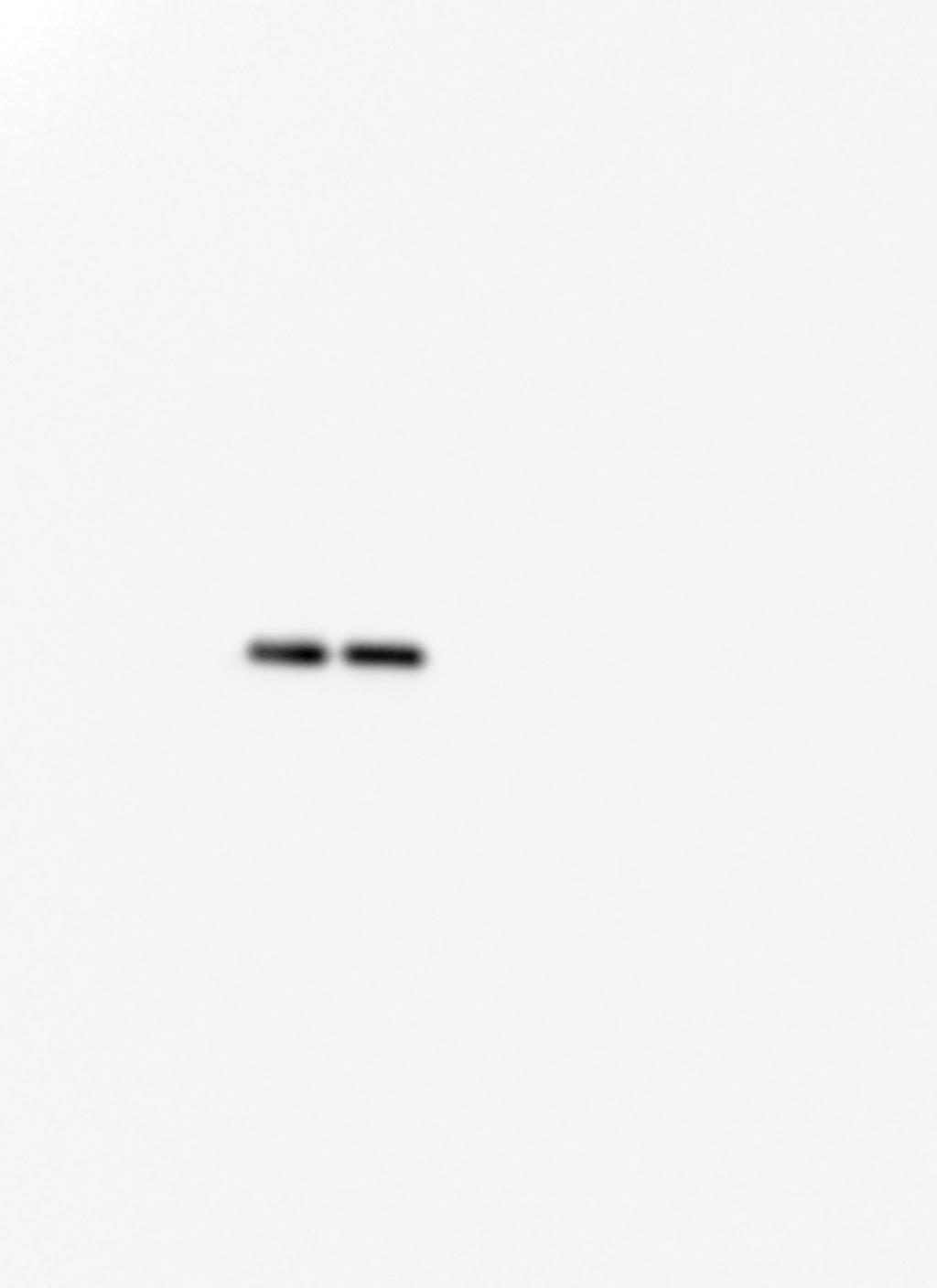

Supplement: Supplemental Information 3 [file peerj-11-15041-s003.zip › Enrichment related gens-raw data1/GAPDH/GAPDH-2/GAPDH-2-1.tif]

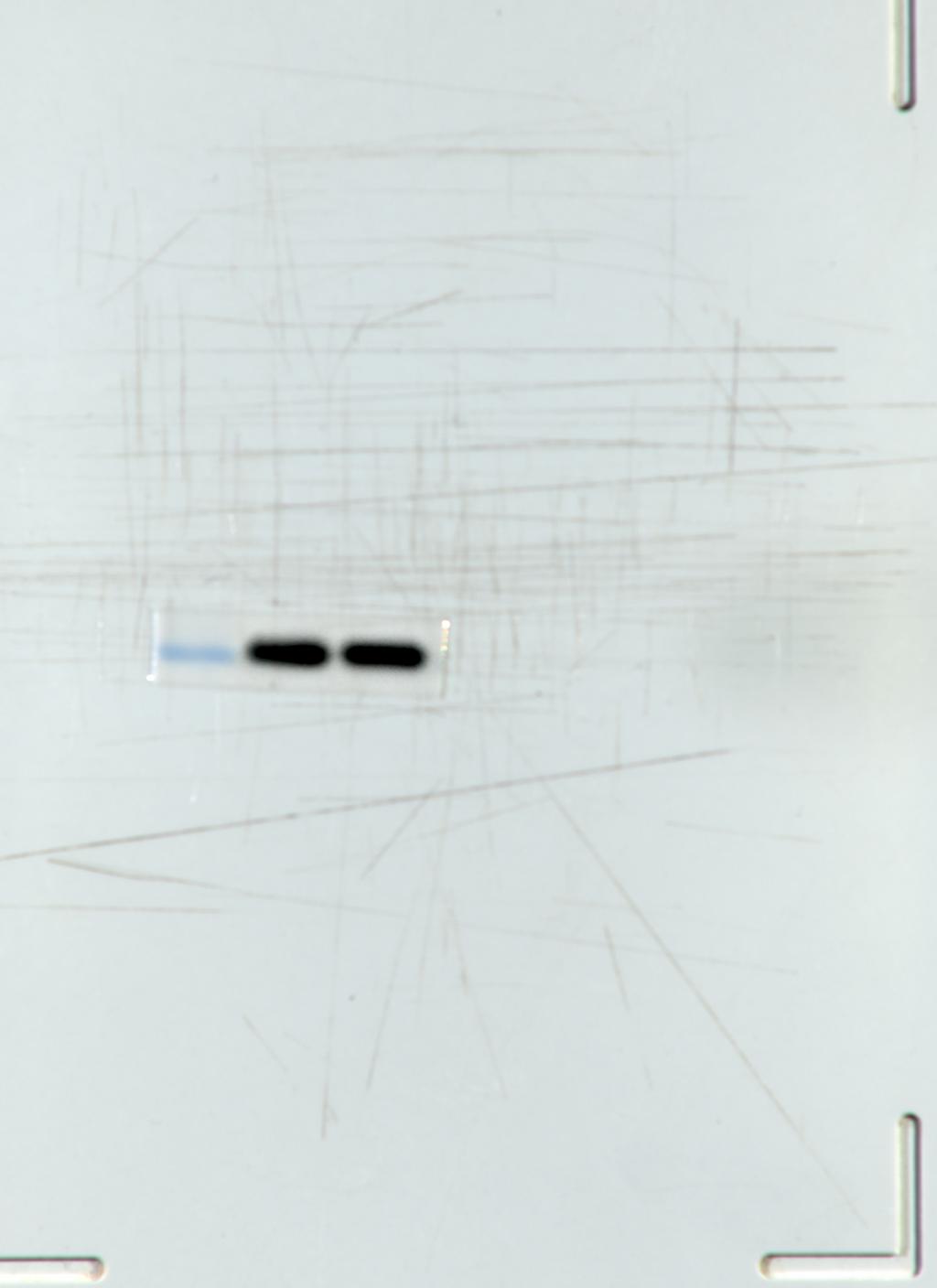

Supplement: Supplemental Information 3 [file peerj-11-15041-s003.zip › Enrichment related gens-raw data1/GAPDH/GAPDH-2/GAPDH-2-2.jpg]

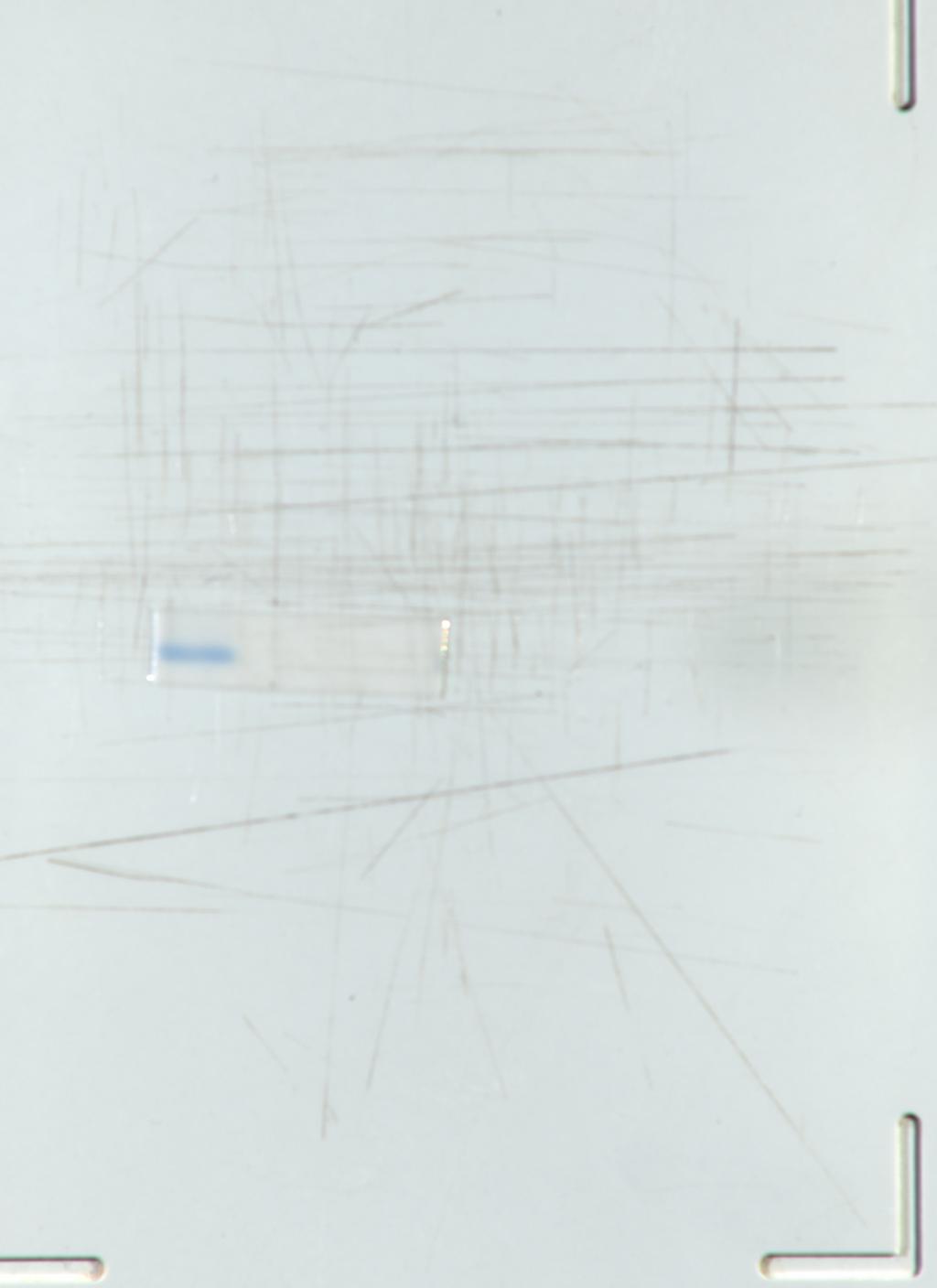

Supplement: Supplemental Information 3 [file peerj-11-15041-s003.zip › Enrichment related gens-raw data1/GAPDH/GAPDH-2/GAPDH-2-3.jpg]

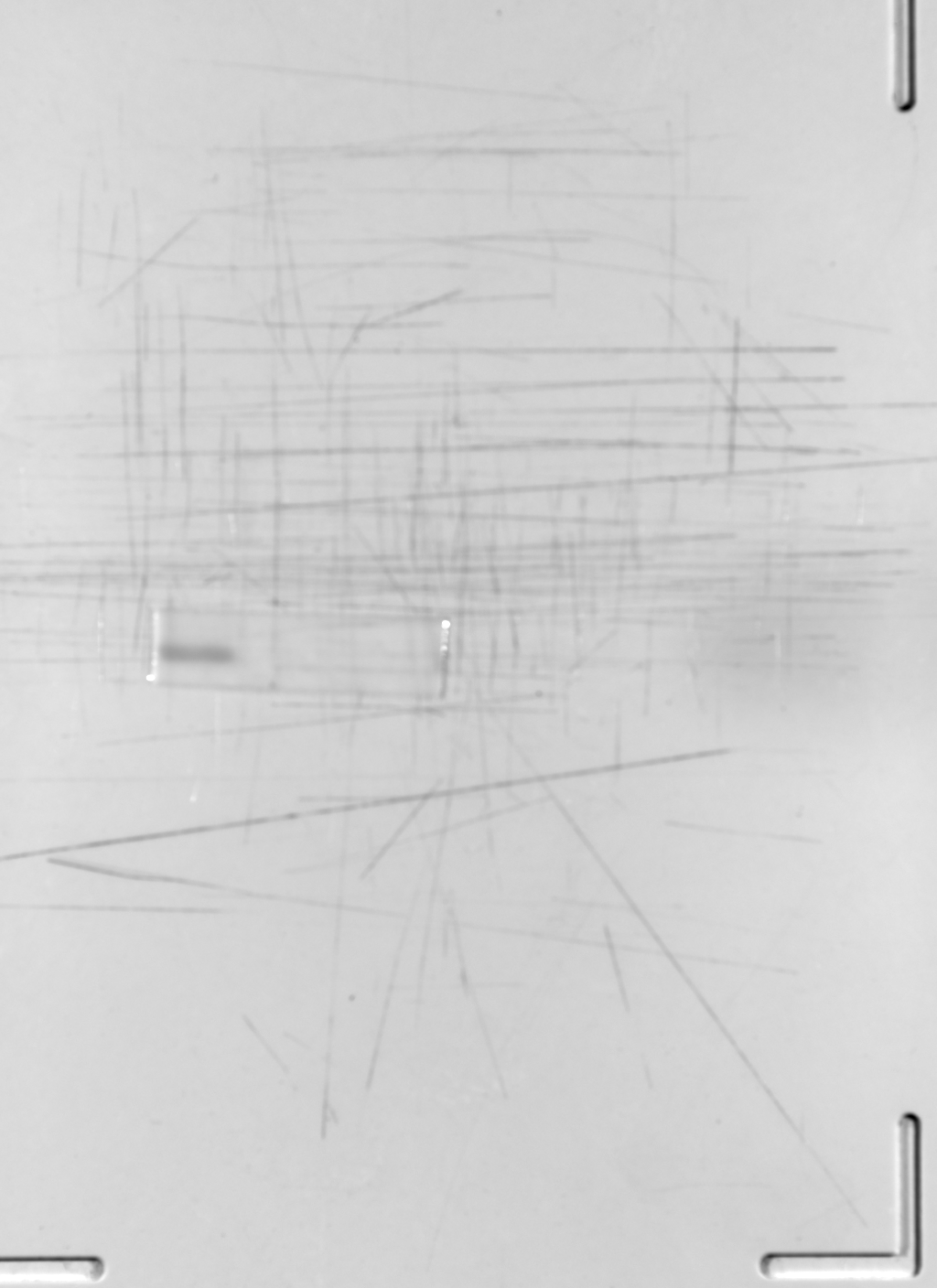

Supplement: Supplemental Information 3 [file peerj-11-15041-s003.zip › Enrichment related gens-raw data1/GAPDH/GAPDH-2/GAPDH-2-4.tif]

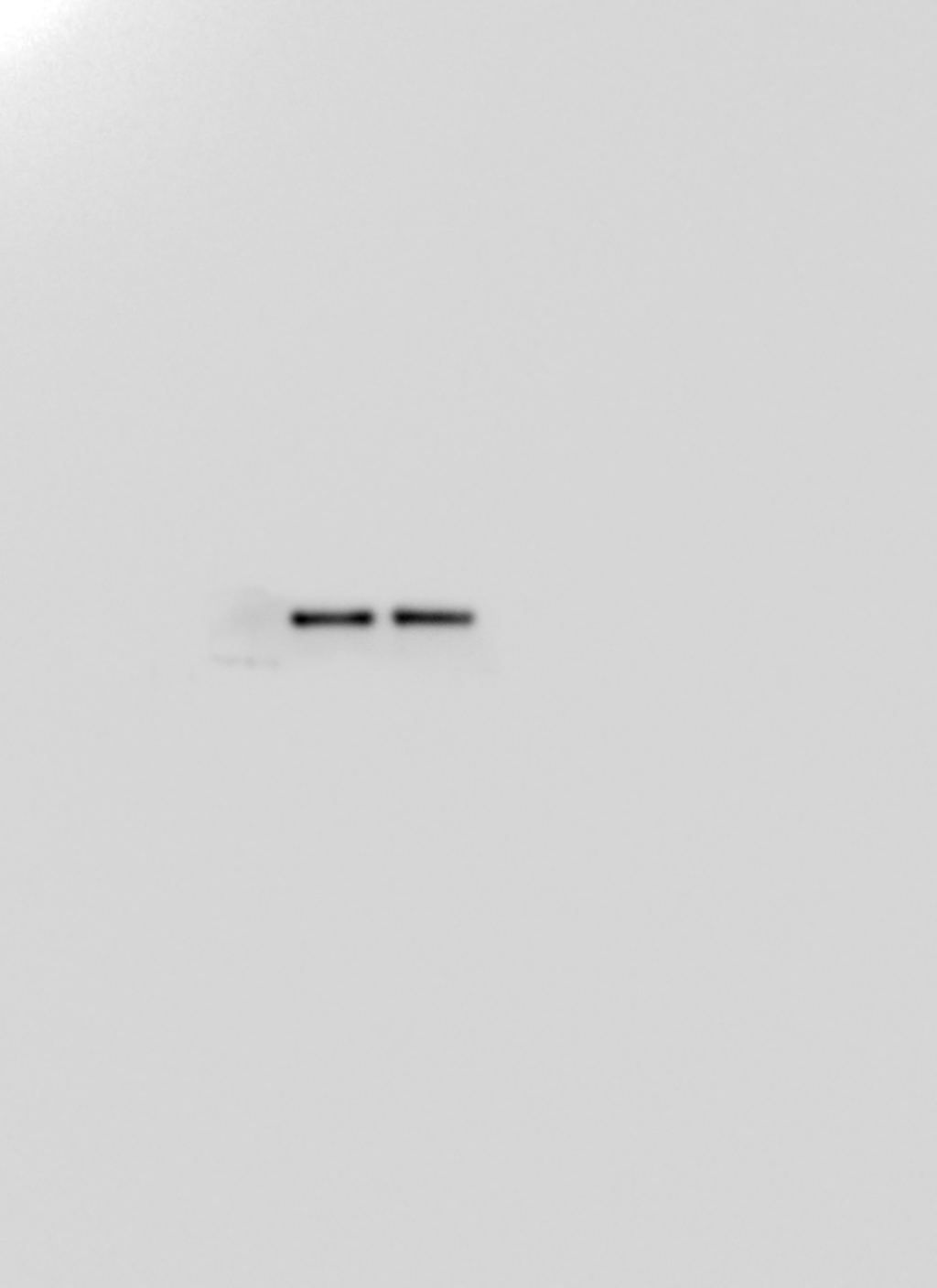

Supplement: Supplemental Information 3 [file peerj-11-15041-s003.zip › Enrichment related gens-raw data1/GAPDH/GAPDH-3/GAPDH-3-1.tif]

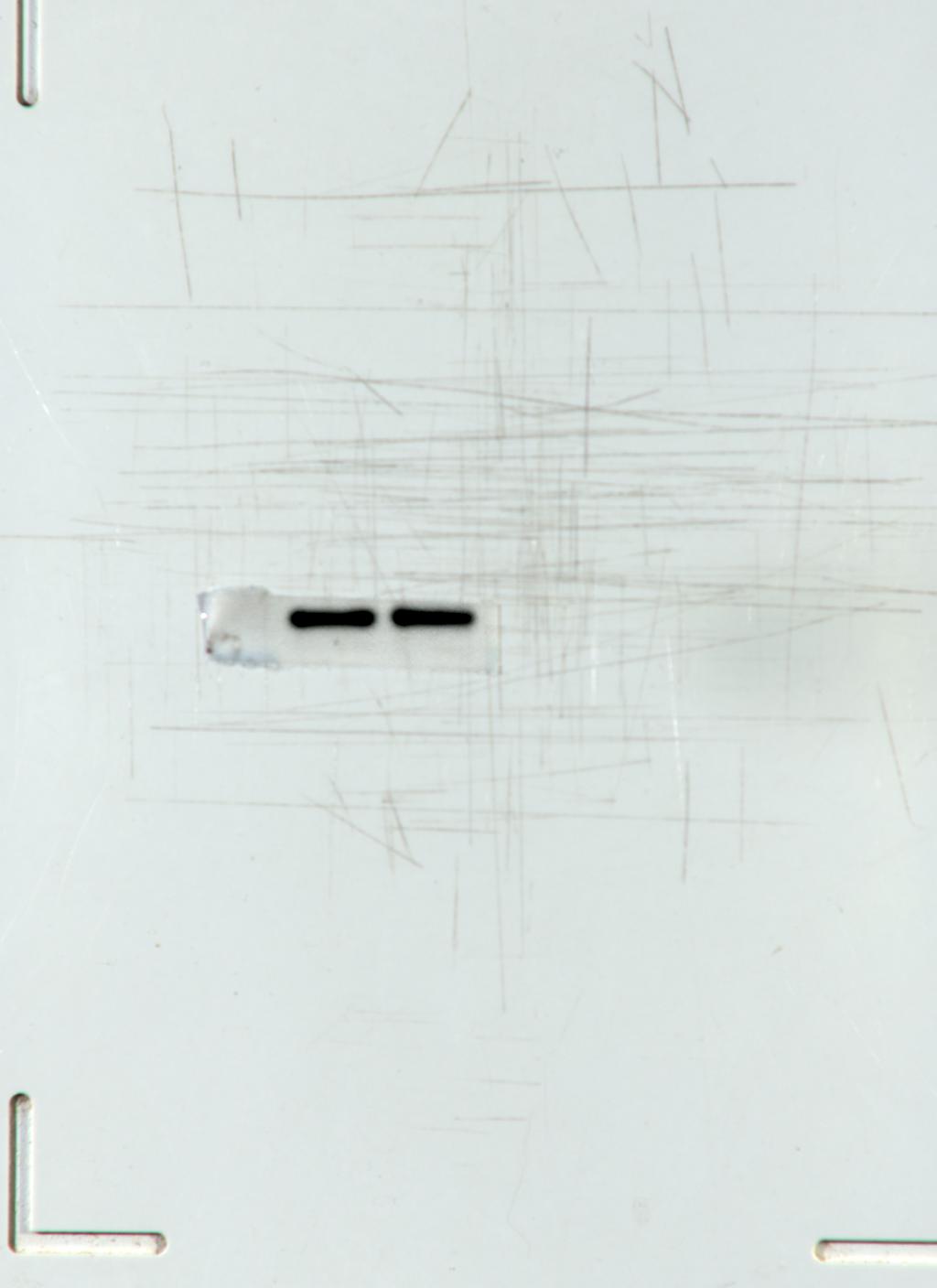

Supplement: Supplemental Information 3 [file peerj-11-15041-s003.zip › Enrichment related gens-raw data1/GAPDH/GAPDH-3/GAPDH-3-2.jpg]

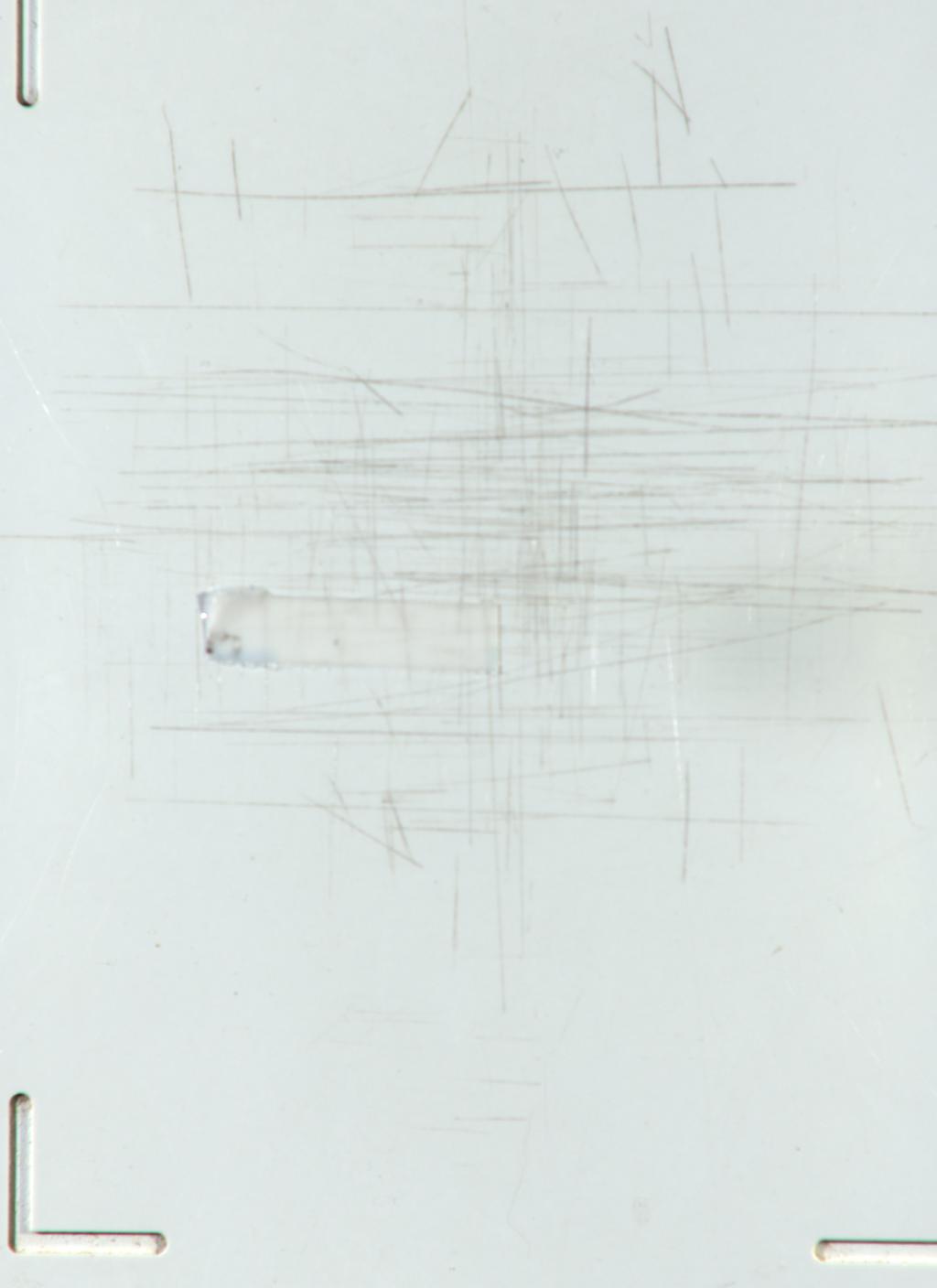

Supplement: Supplemental Information 3 [file peerj-11-15041-s003.zip › Enrichment related gens-raw data1/GAPDH/GAPDH-3/GAPDH-3-3.jpg]

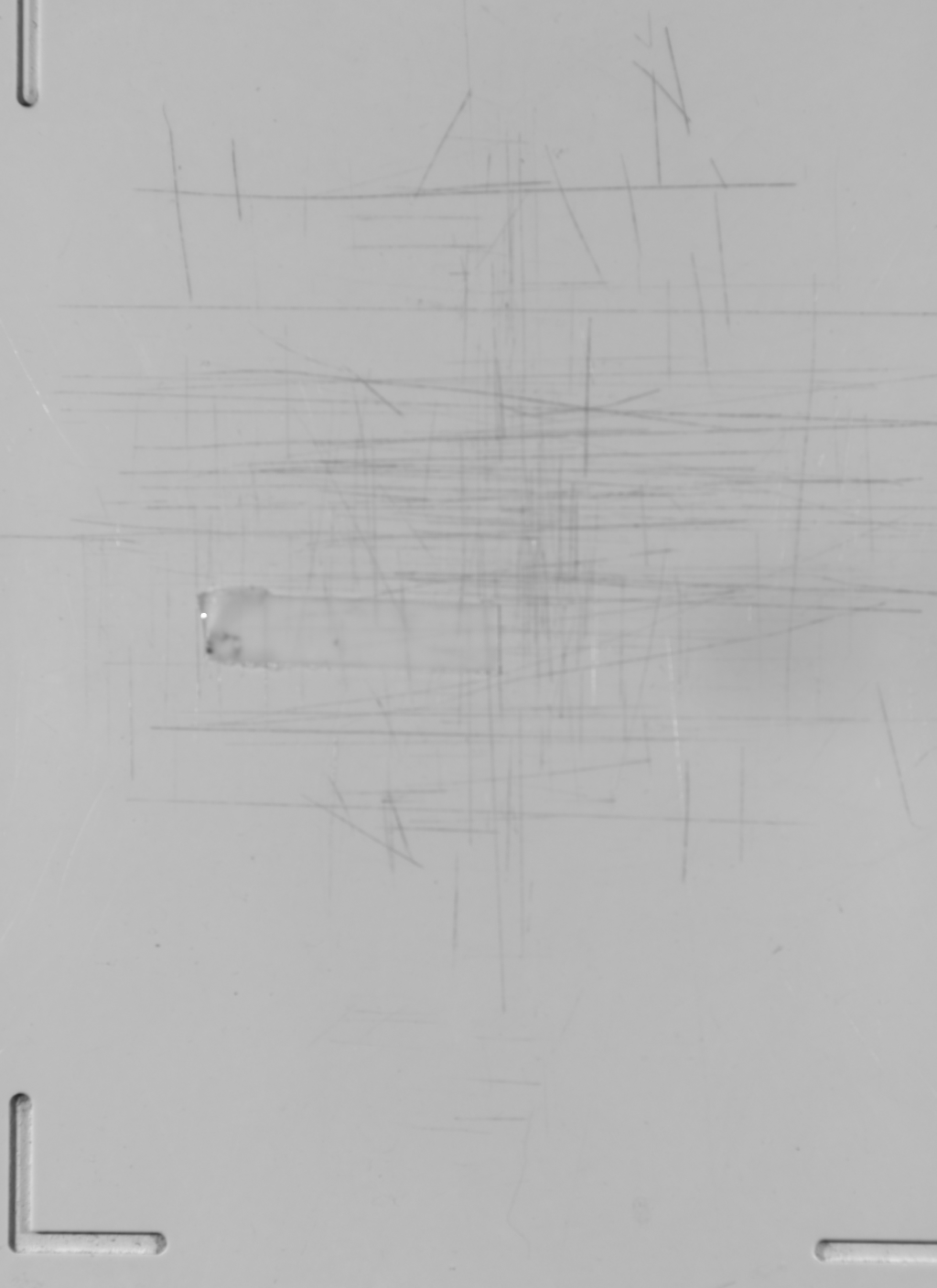

Supplement: Supplemental Information 3 [file peerj-11-15041-s003.zip › Enrichment related gens-raw data1/GAPDH/GAPDH-3/GAPDH-3-4.tif]

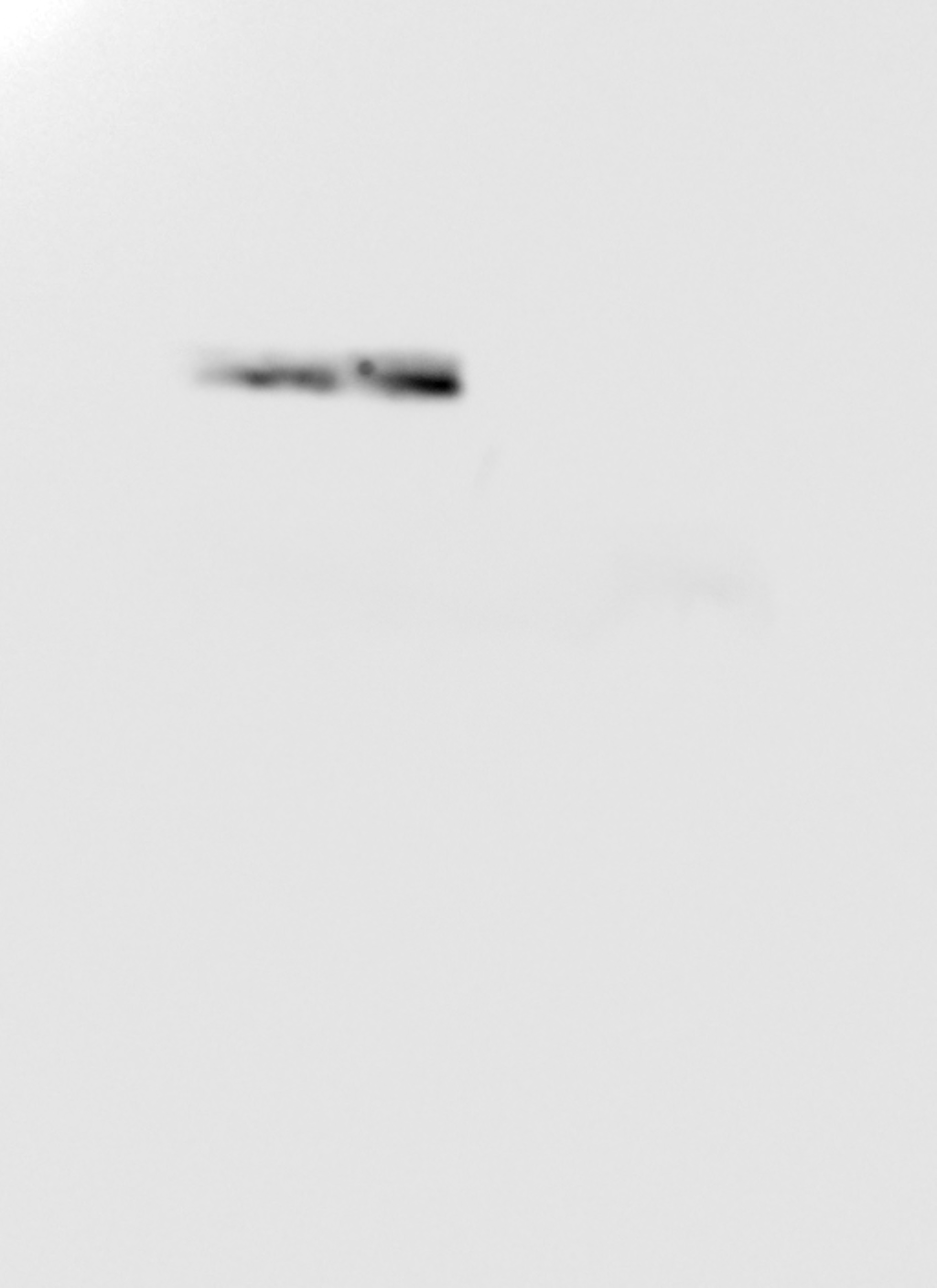

Supplement: Supplemental Information 3 [file peerj-11-15041-s003.zip › Enrichment related gens-raw data1/IL7/IL7-1/IL7-1-1.tif]

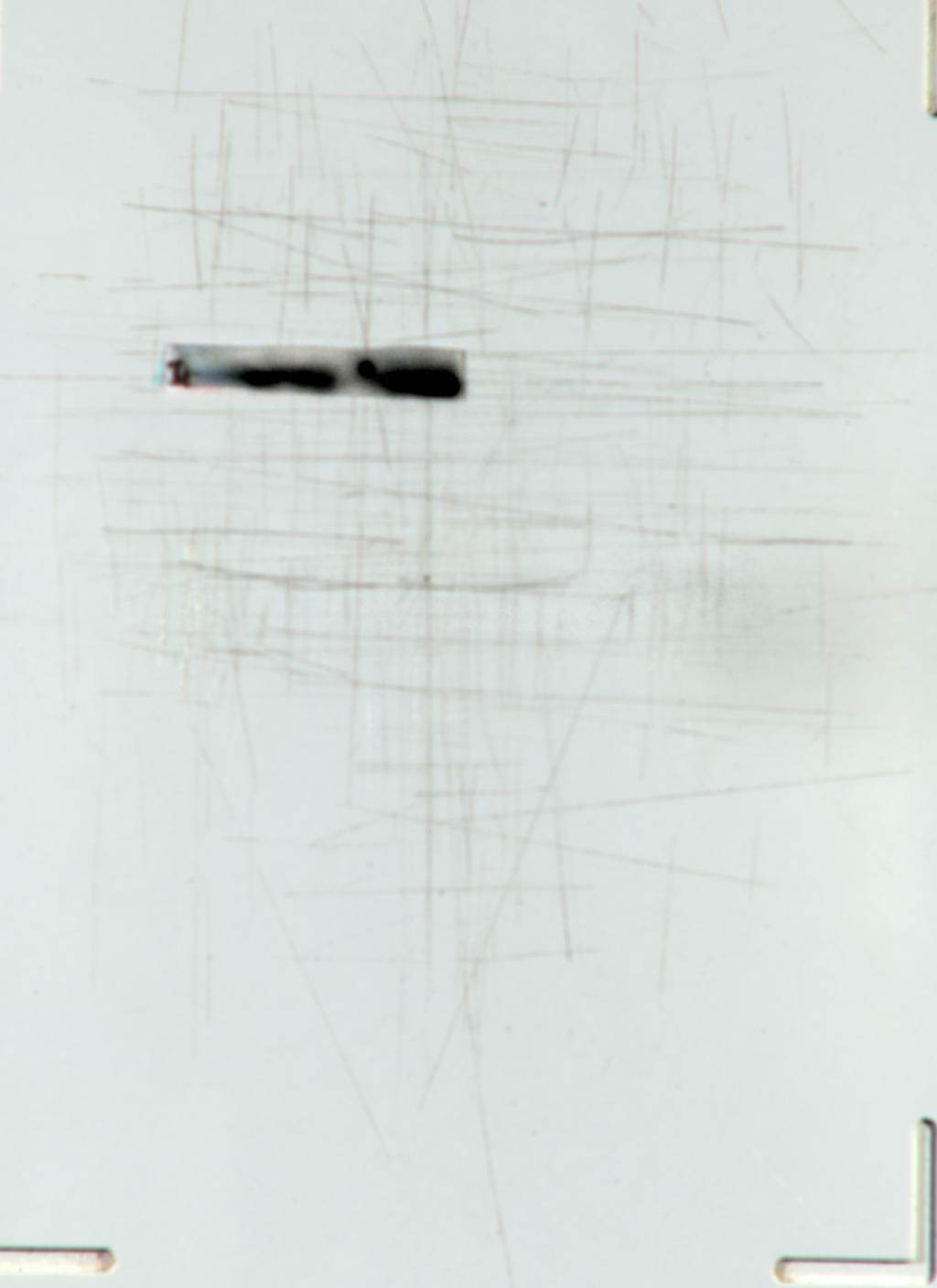

Supplement: Supplemental Information 3 [file peerj-11-15041-s003.zip › Enrichment related gens-raw data1/IL7/IL7-1/IL7-1-2.jpg]

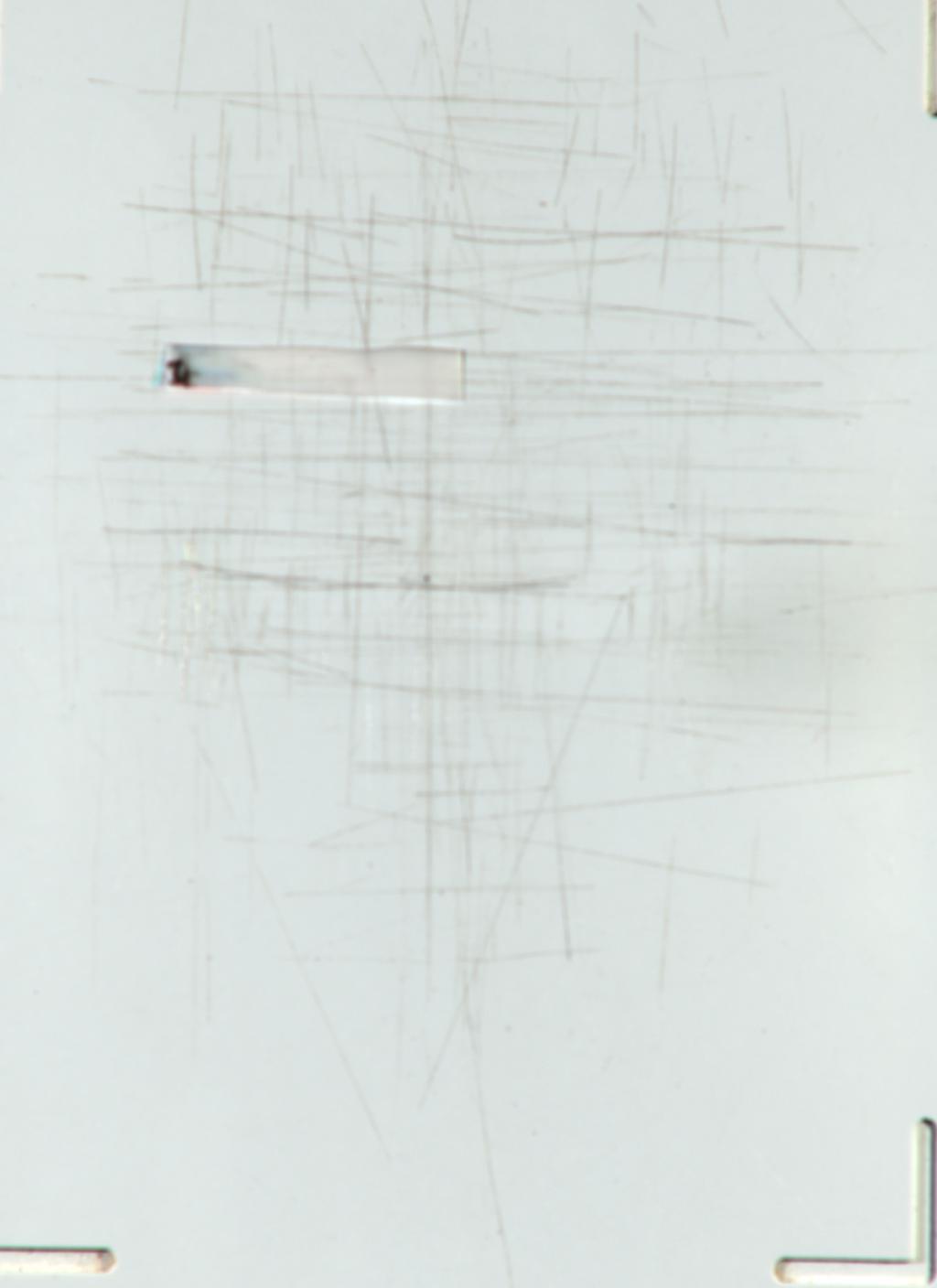

Supplement: Supplemental Information 3 [file peerj-11-15041-s003.zip › Enrichment related gens-raw data1/IL7/IL7-1/IL7-1-3.jpg]

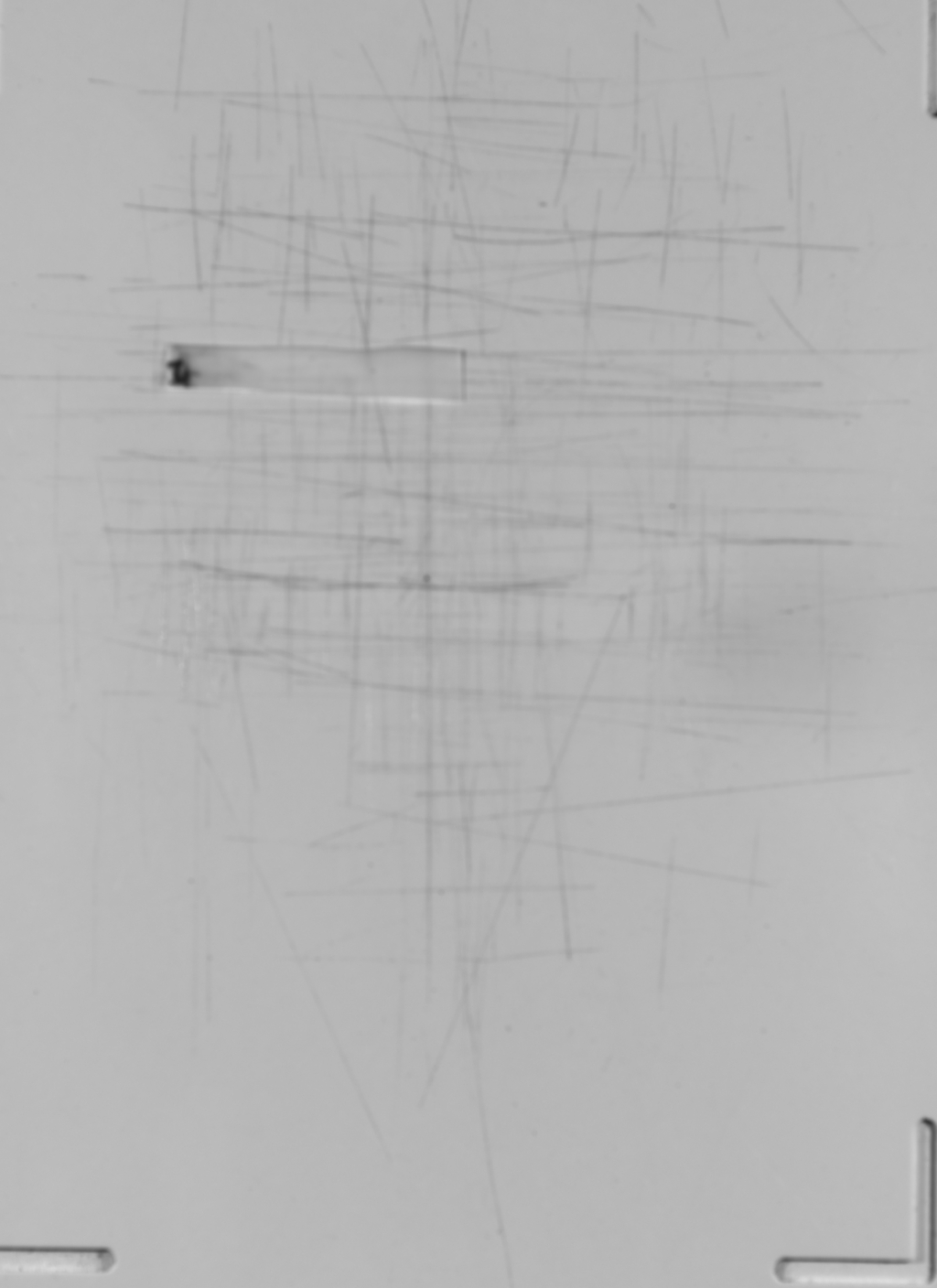

Supplement: Supplemental Information 3 [file peerj-11-15041-s003.zip › Enrichment related gens-raw data1/IL7/IL7-1/IL7-1-4.tif]

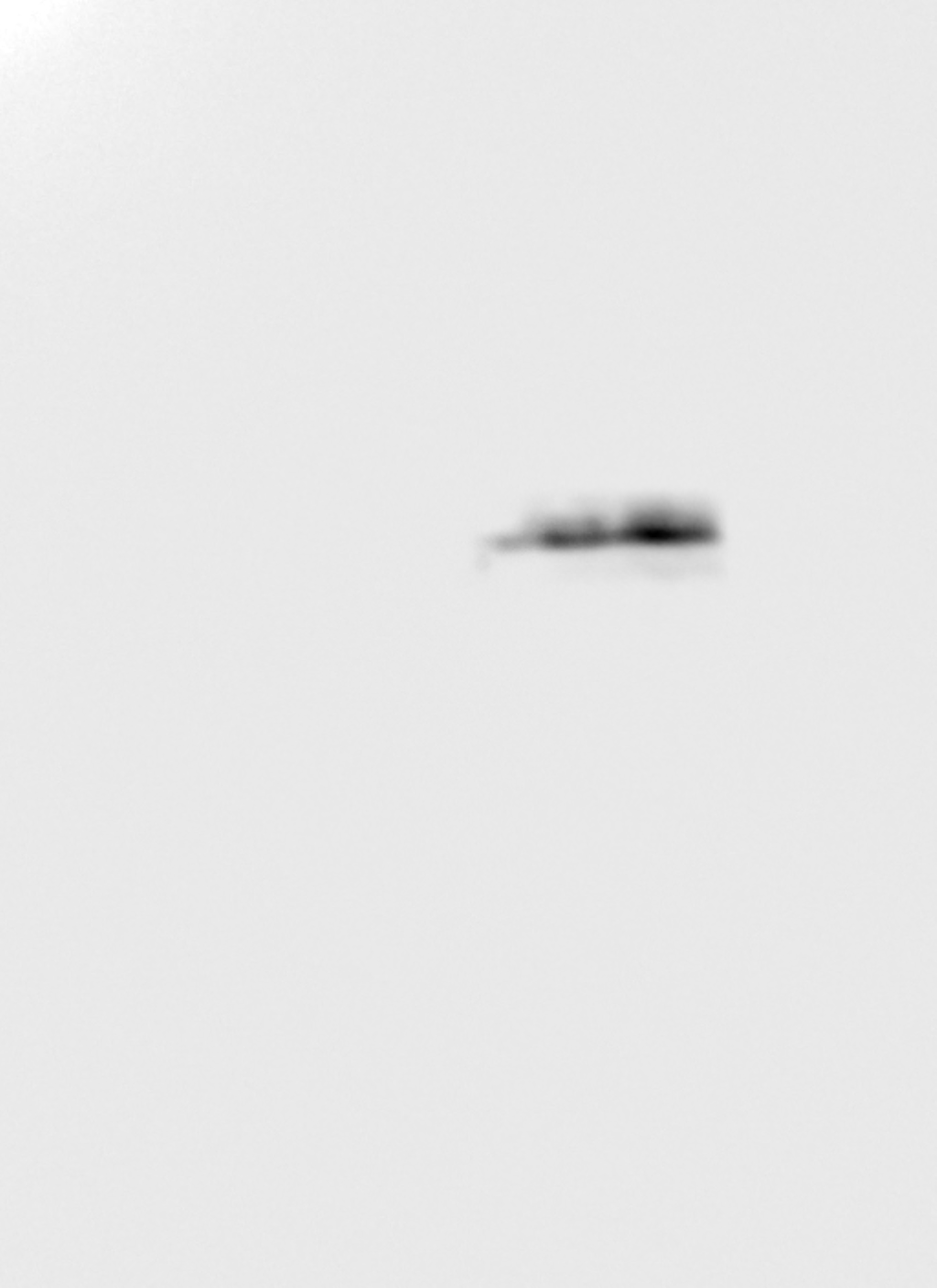

Supplement: Supplemental Information 3 [file peerj-11-15041-s003.zip › Enrichment related gens-raw data1/IL7/IL7-2/IL7-2-1.tif]

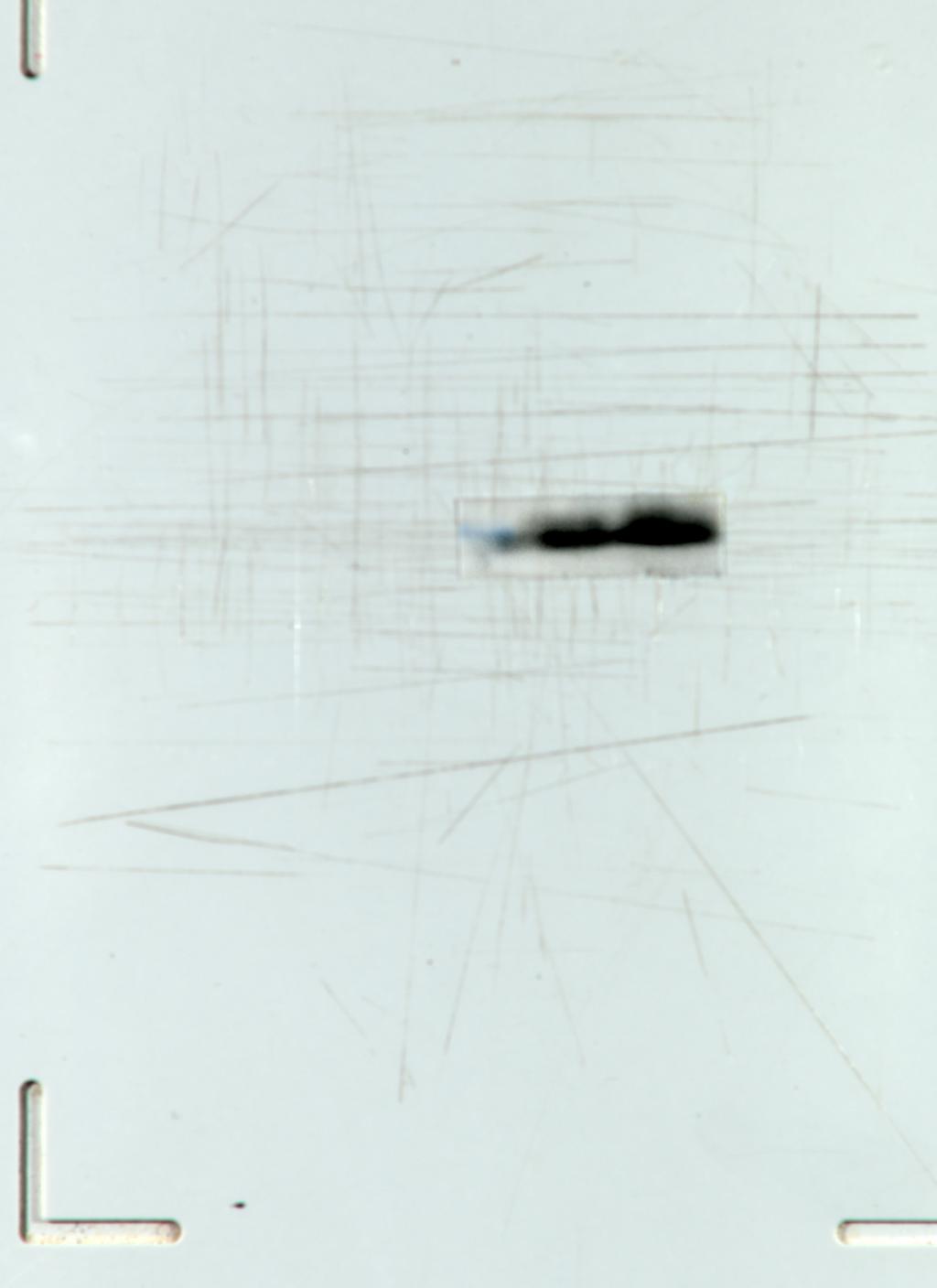

Supplement: Supplemental Information 3 [file peerj-11-15041-s003.zip › Enrichment related gens-raw data1/IL7/IL7-2/IL7-2-2.jpg]

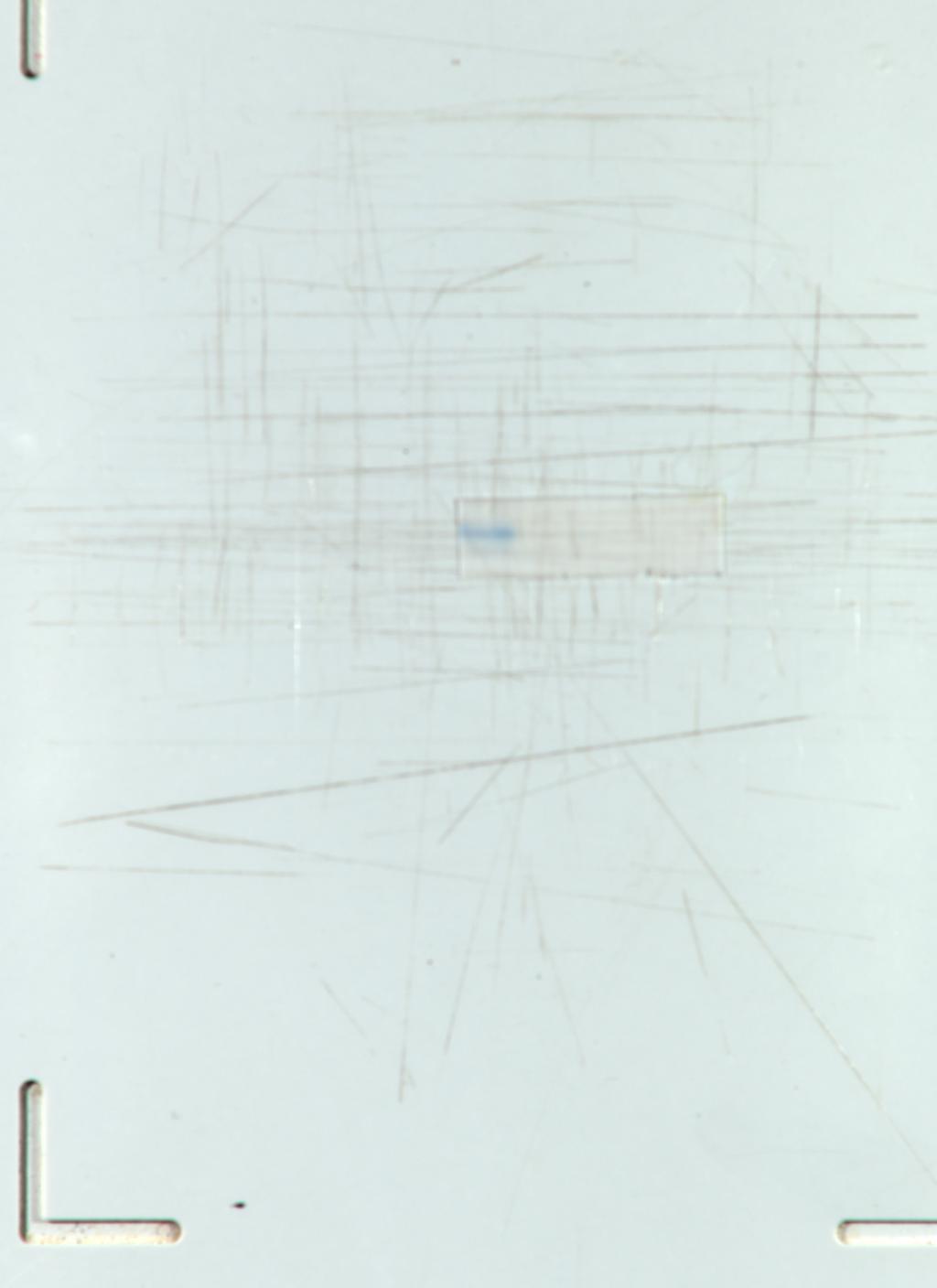

Supplement: Supplemental Information 3 [file peerj-11-15041-s003.zip › Enrichment related gens-raw data1/IL7/IL7-2/IL7-2-3.jpg]

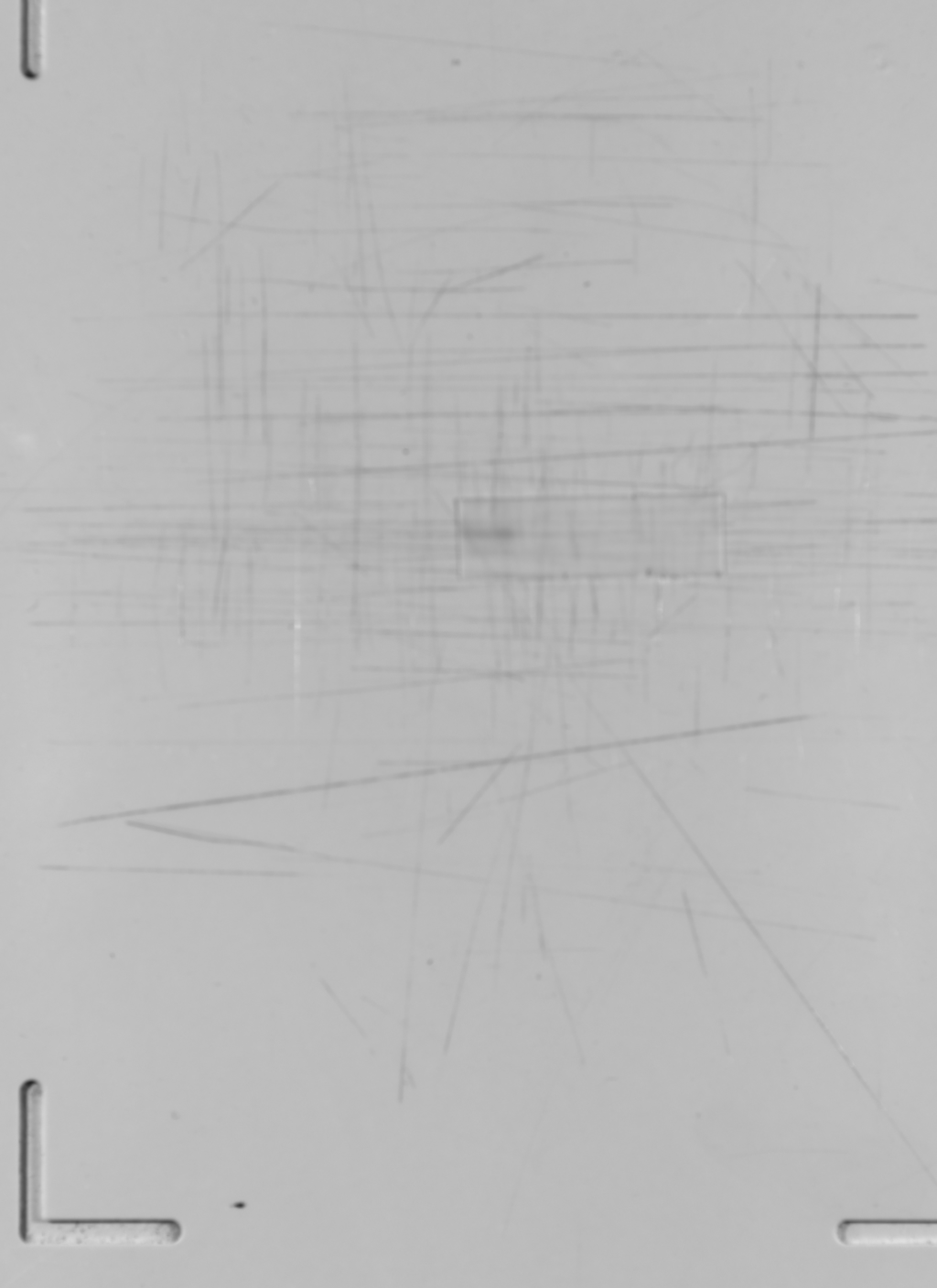

Supplement: Supplemental Information 3 [file peerj-11-15041-s003.zip › Enrichment related gens-raw data1/IL7/IL7-2/IL7-2-4.tif]

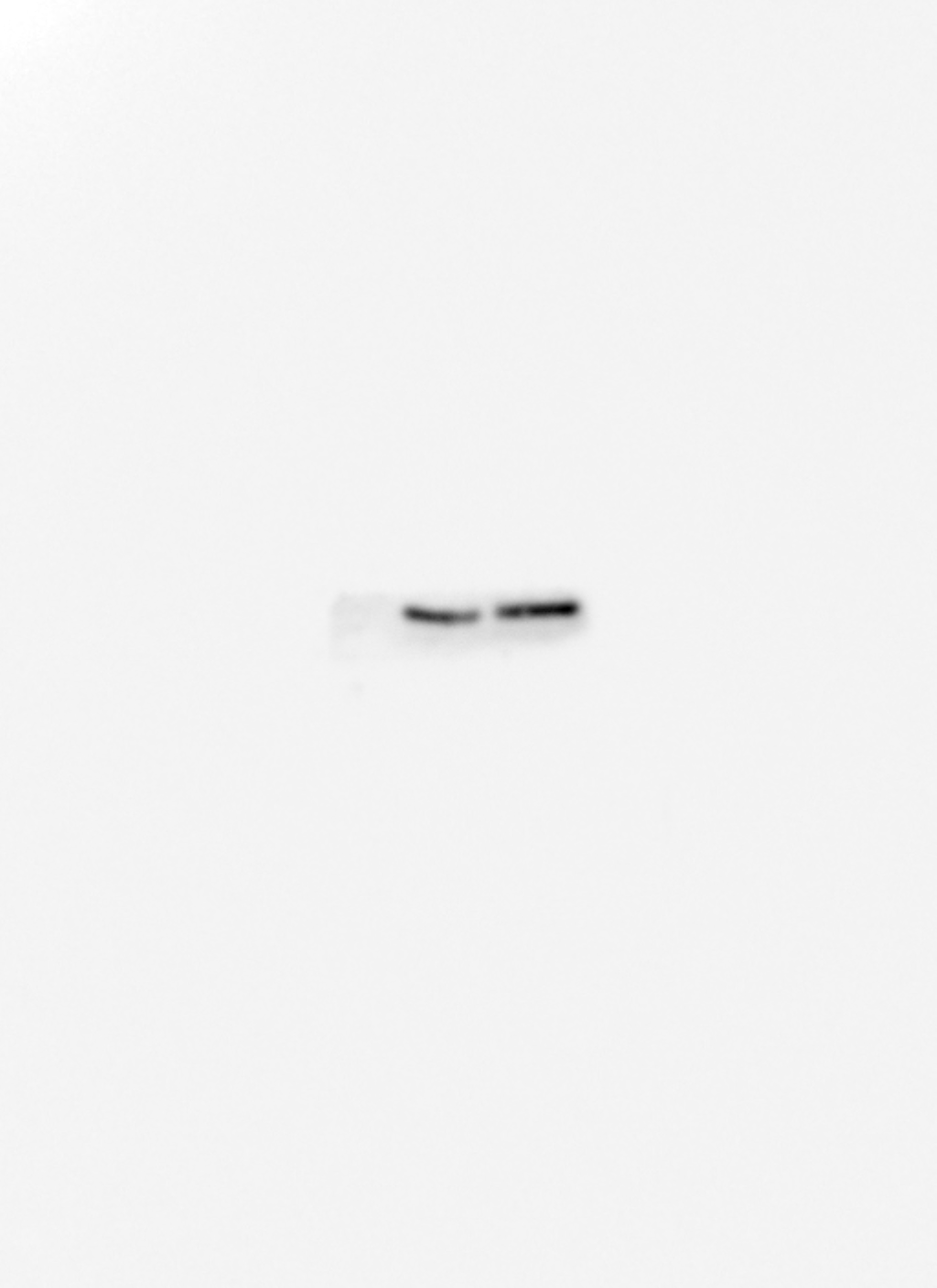

Supplement: Supplemental Information 3 [file peerj-11-15041-s003.zip › Enrichment related gens-raw data1/IL7/IL7-3/IL7-3-1.tif]

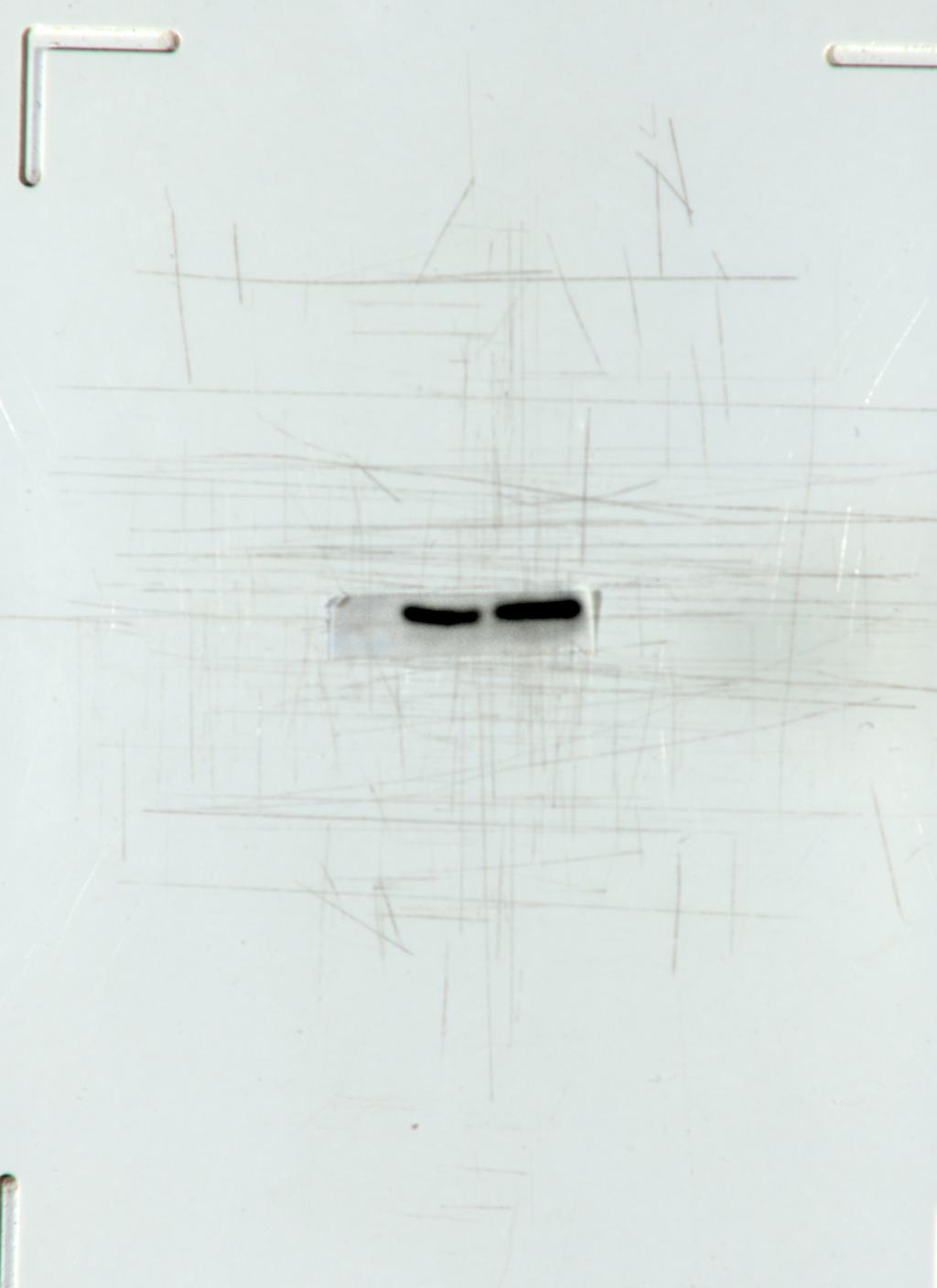

Supplement: Supplemental Information 3 [file peerj-11-15041-s003.zip › Enrichment related gens-raw data1/IL7/IL7-3/IL7-3-2.jpg]

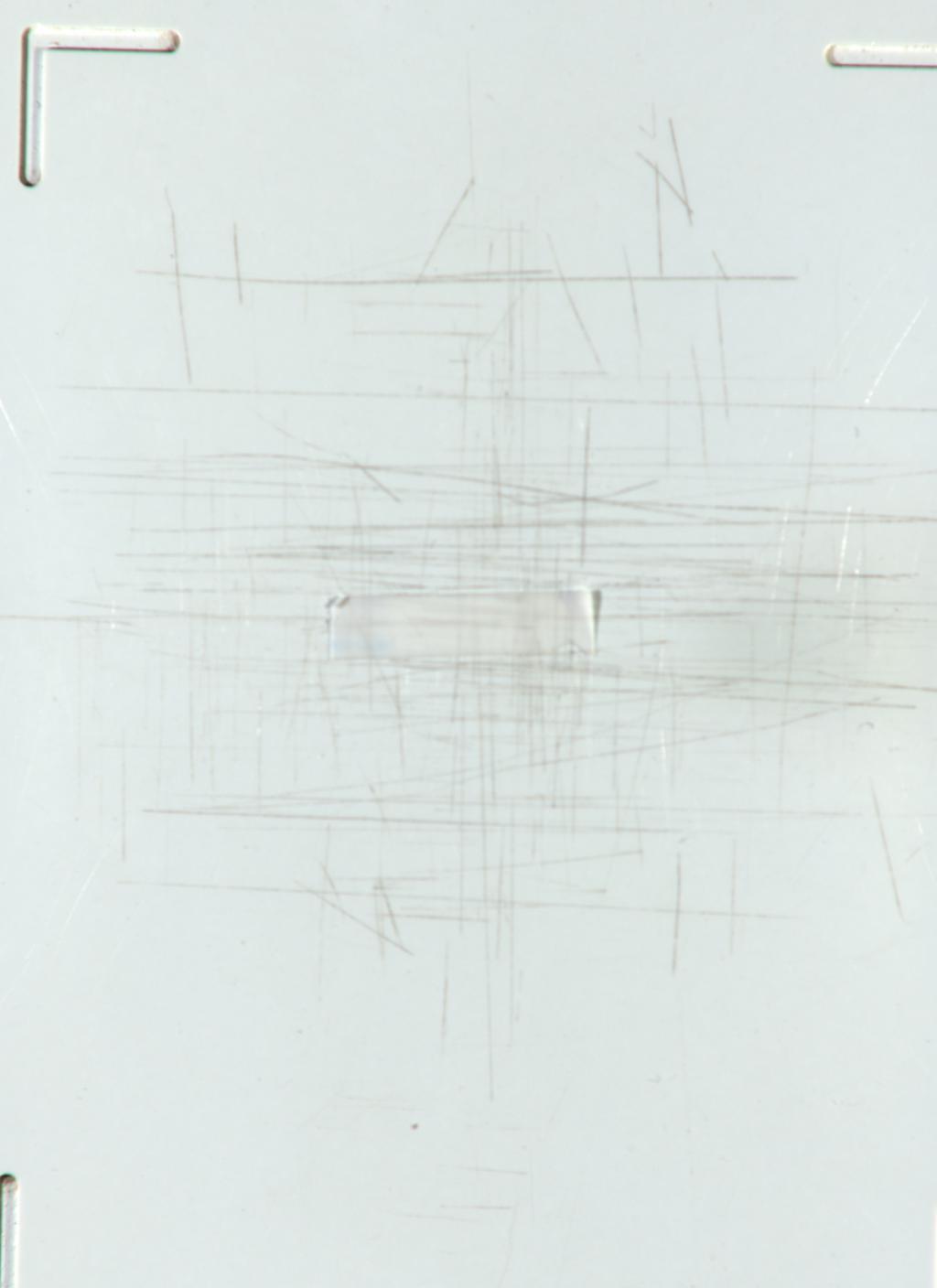

Supplement: Supplemental Information 3 [file peerj-11-15041-s003.zip › Enrichment related gens-raw data1/IL7/IL7-3/IL7-3-3.jpg]

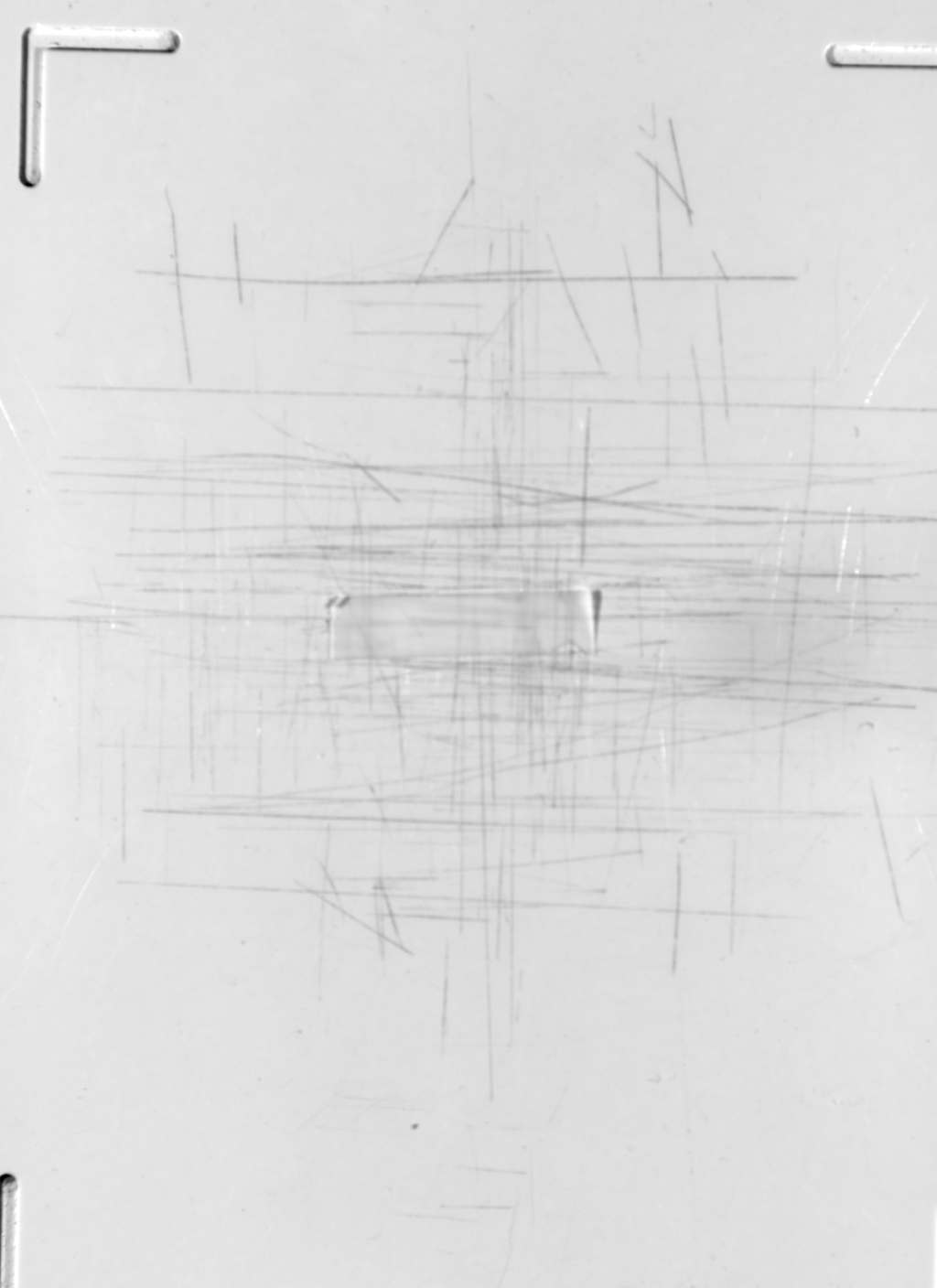

Supplement: Supplemental Information 3 [file peerj-11-15041-s003.zip › Enrichment related gens-raw data1/IL7/IL7-3/IL7-3-4.tif]

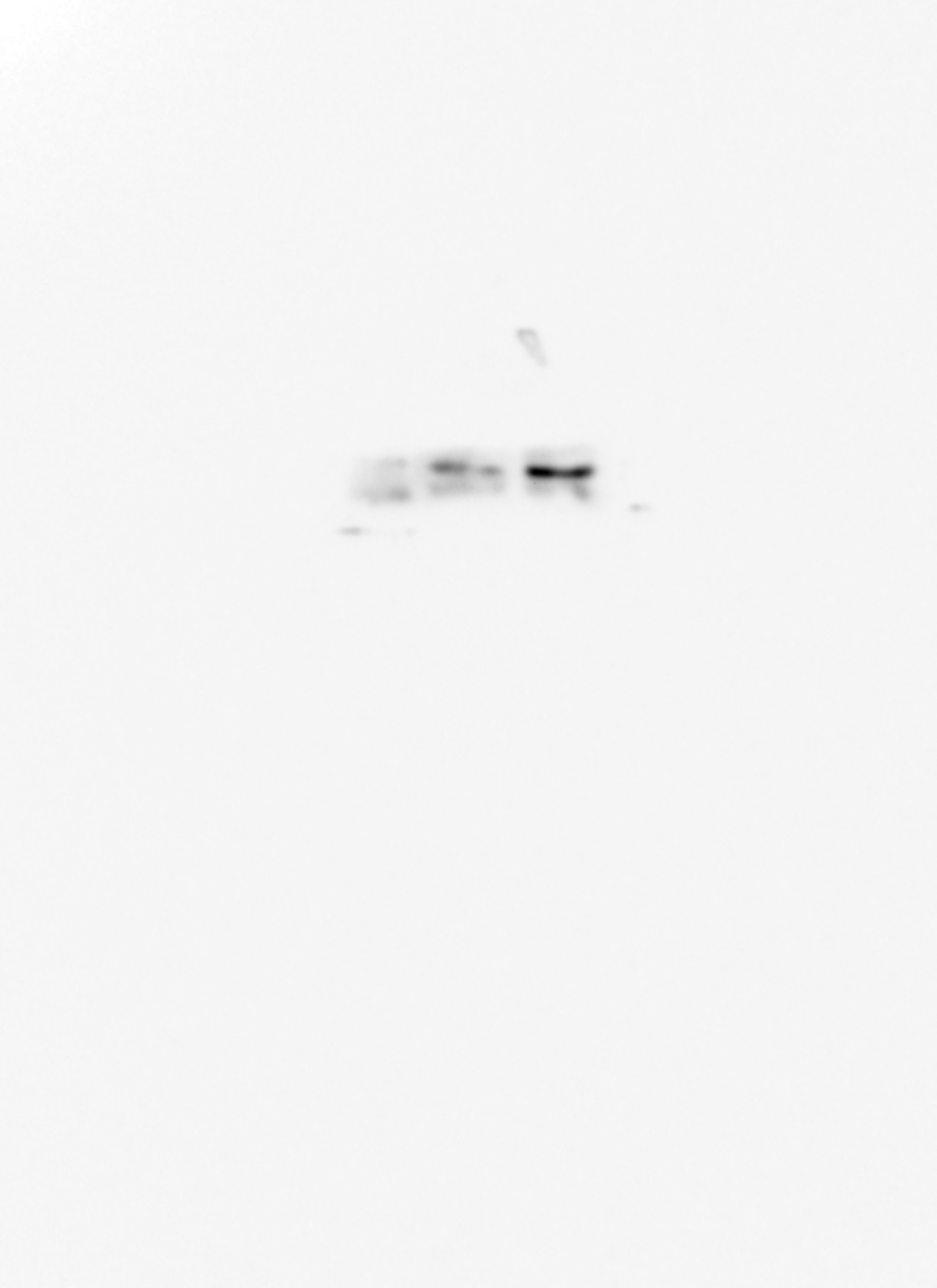

Supplement: Supplemental Information 4 [file peerj-11-15041-s004.zip › Enrichment-related genes-raw data2/AKT/AKT-1/AKT-1-1.tif]

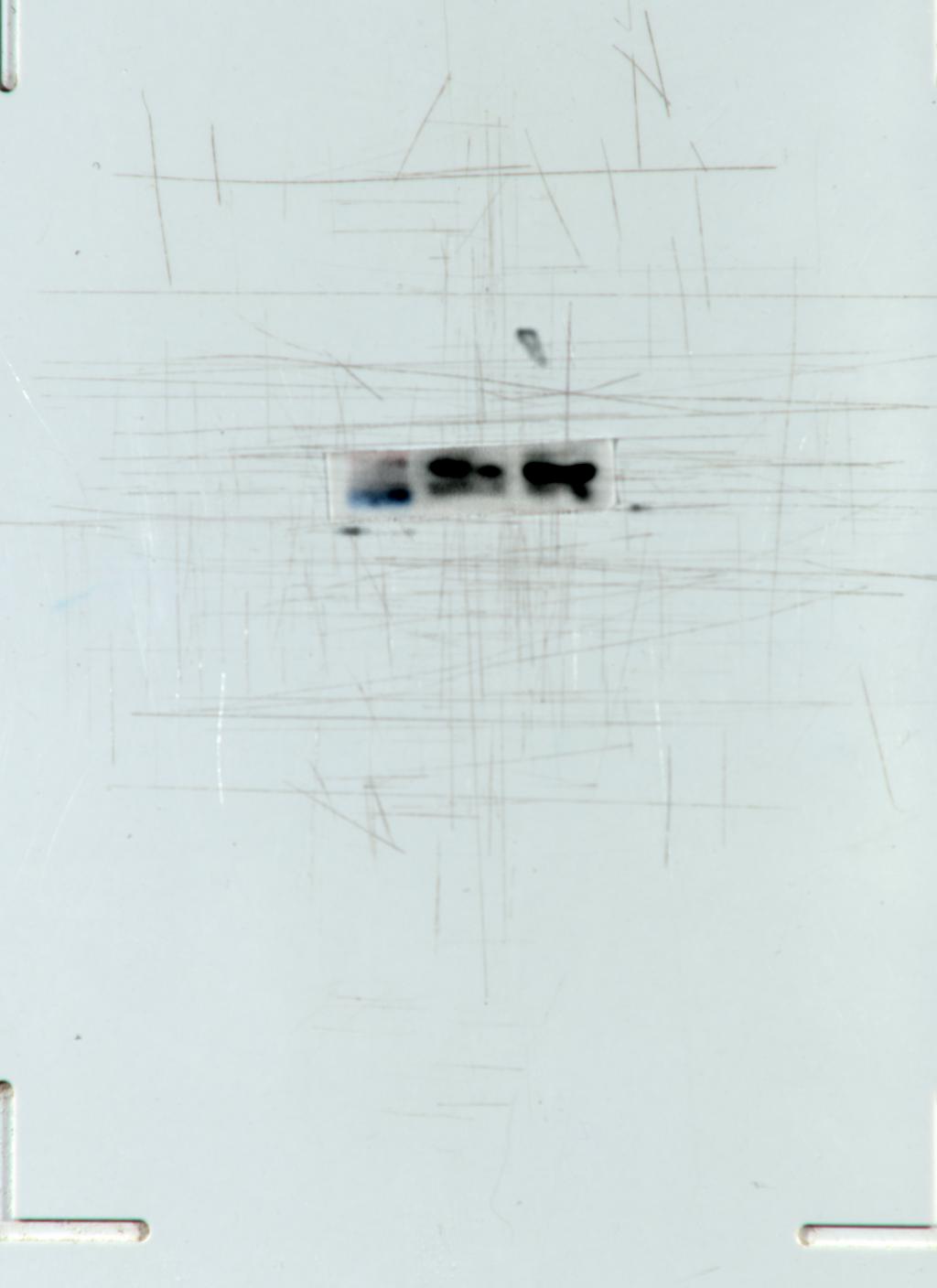

Supplement: Supplemental Information 4 [file peerj-11-15041-s004.zip › Enrichment-related genes-raw data2/AKT/AKT-1/AKT-1-2.jpg]

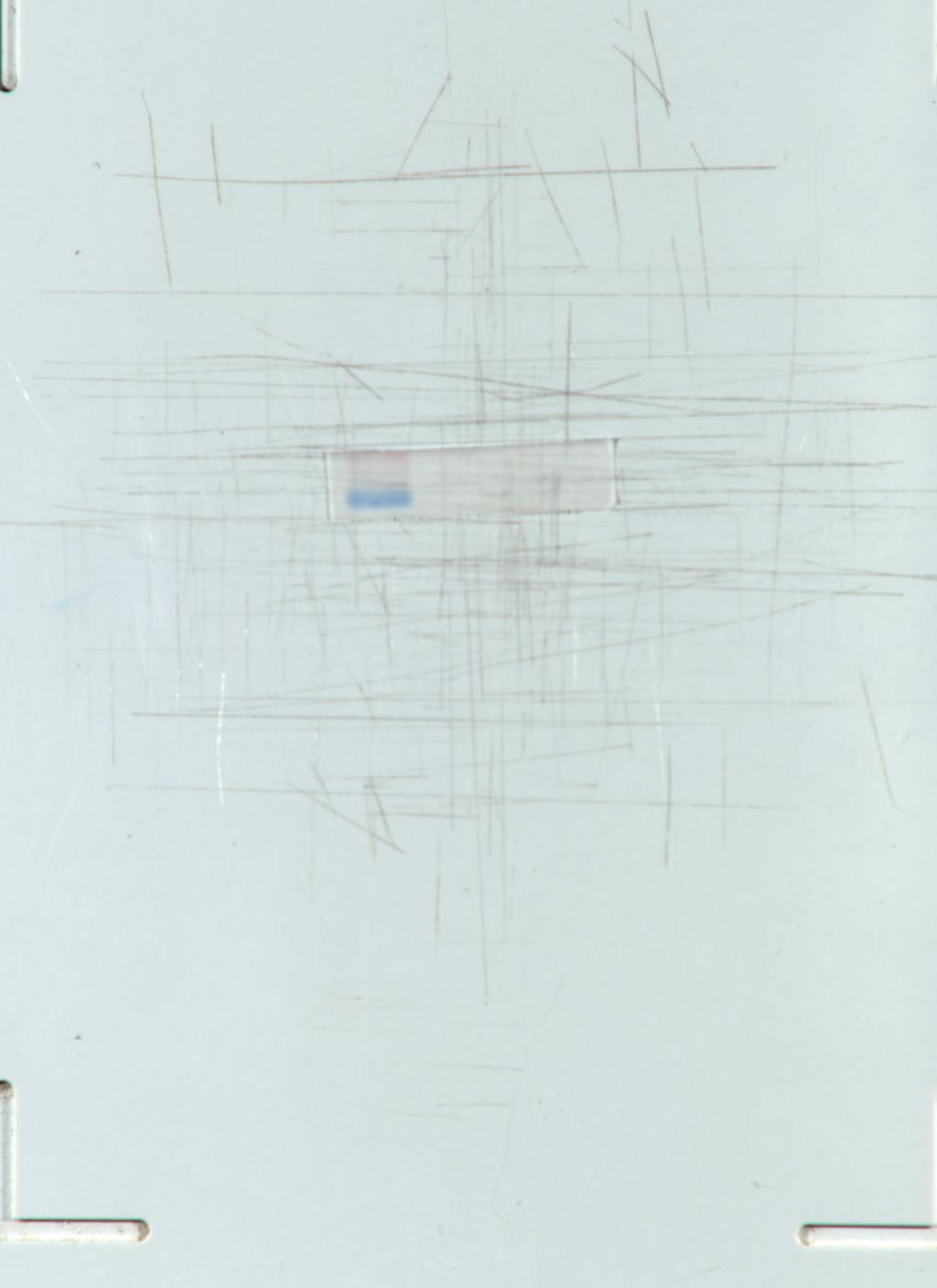

Supplement: Supplemental Information 4 [file peerj-11-15041-s004.zip › Enrichment-related genes-raw data2/AKT/AKT-1/AKT-1-3.jpg]

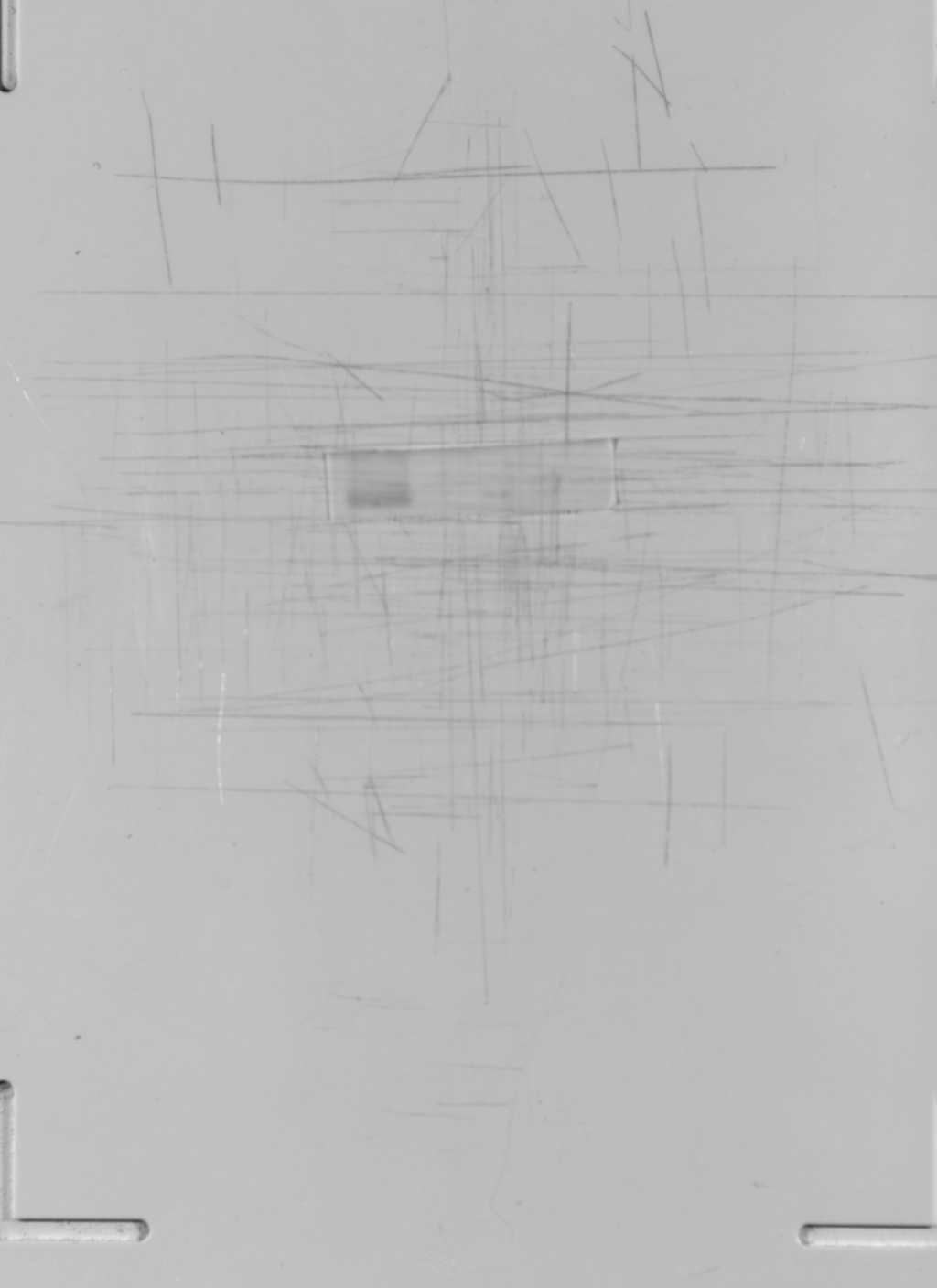

Supplement: Supplemental Information 4 [file peerj-11-15041-s004.zip › Enrichment-related genes-raw data2/AKT/AKT-1/AKT-1-4.tif]

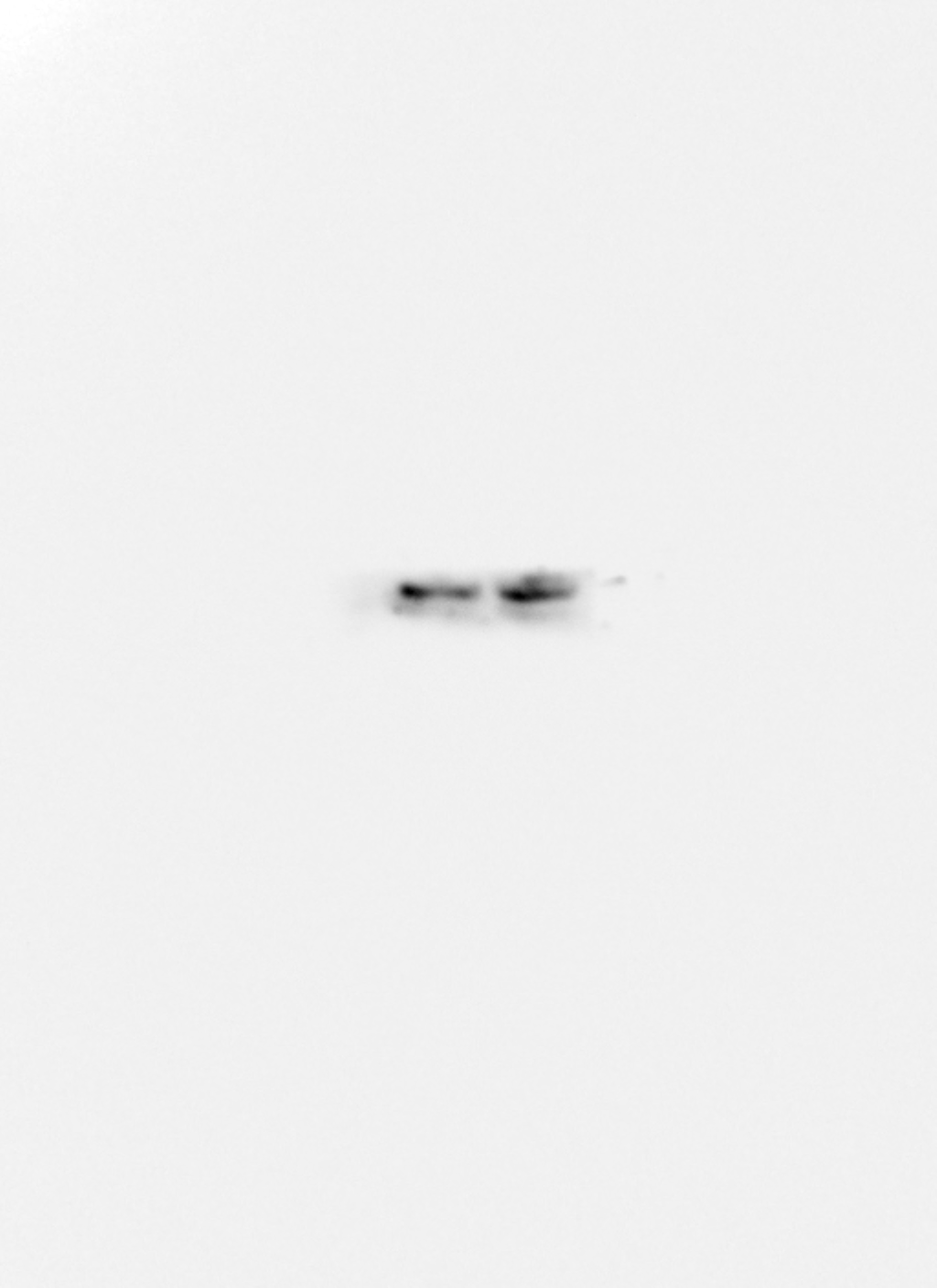

Supplement: Supplemental Information 4 [file peerj-11-15041-s004.zip › Enrichment-related genes-raw data2/AKT/AKT-2/AKT-2-1.tif]

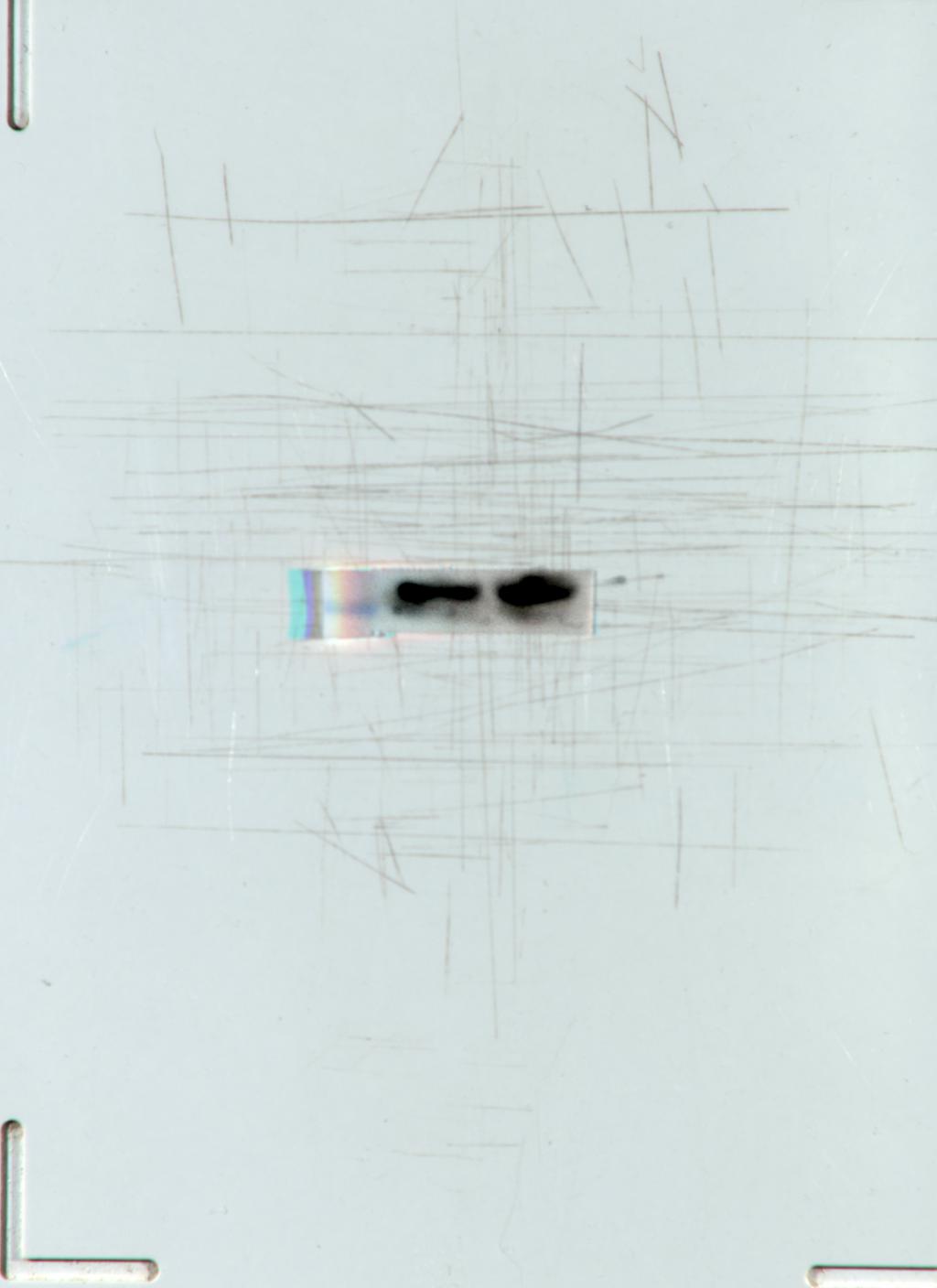

Supplement: Supplemental Information 4 [file peerj-11-15041-s004.zip › Enrichment-related genes-raw data2/AKT/AKT-2/AKT-2-2.jpg]

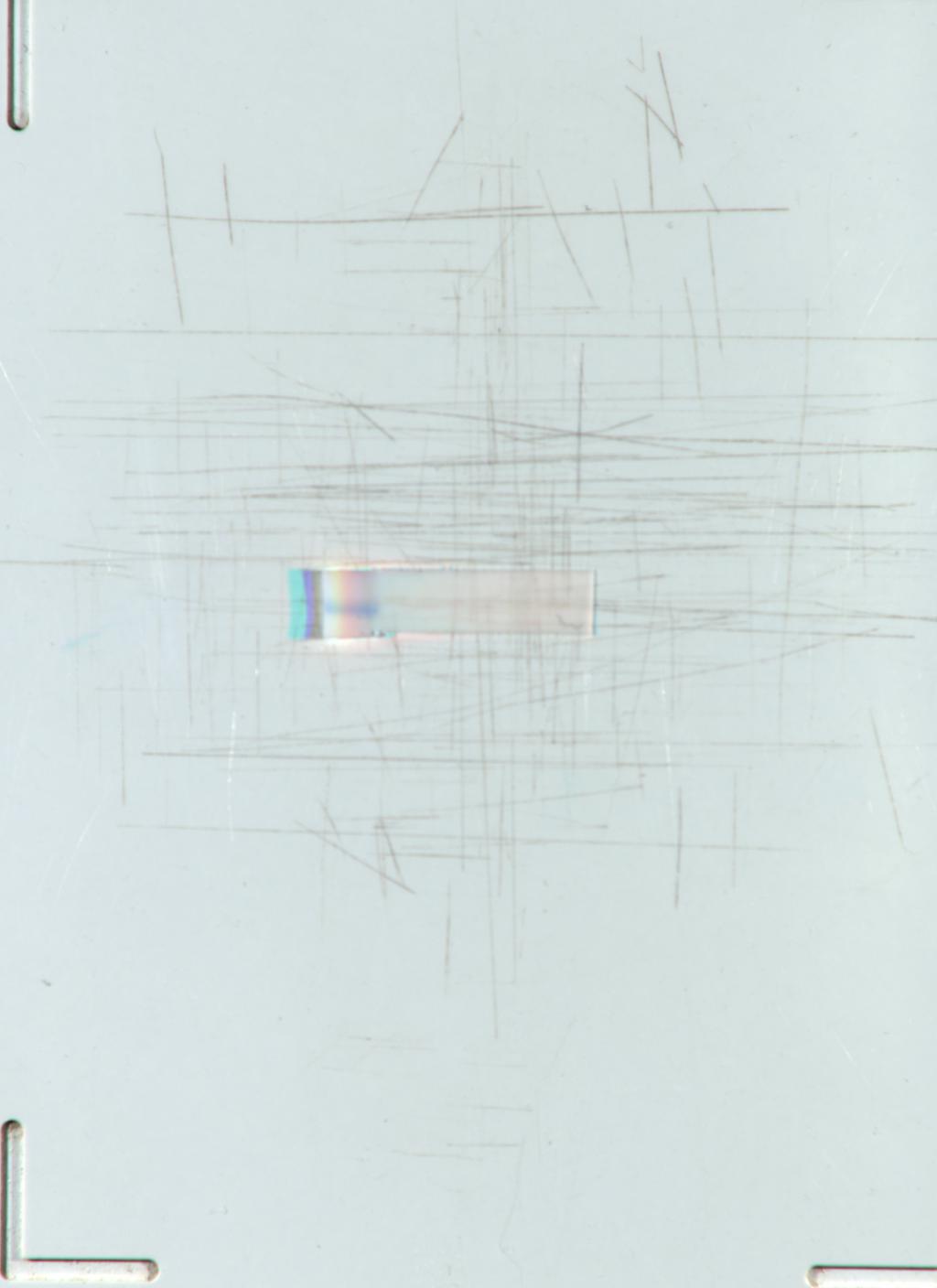

Supplement: Supplemental Information 4 [file peerj-11-15041-s004.zip › Enrichment-related genes-raw data2/AKT/AKT-2/AKT-2-3.jpg]

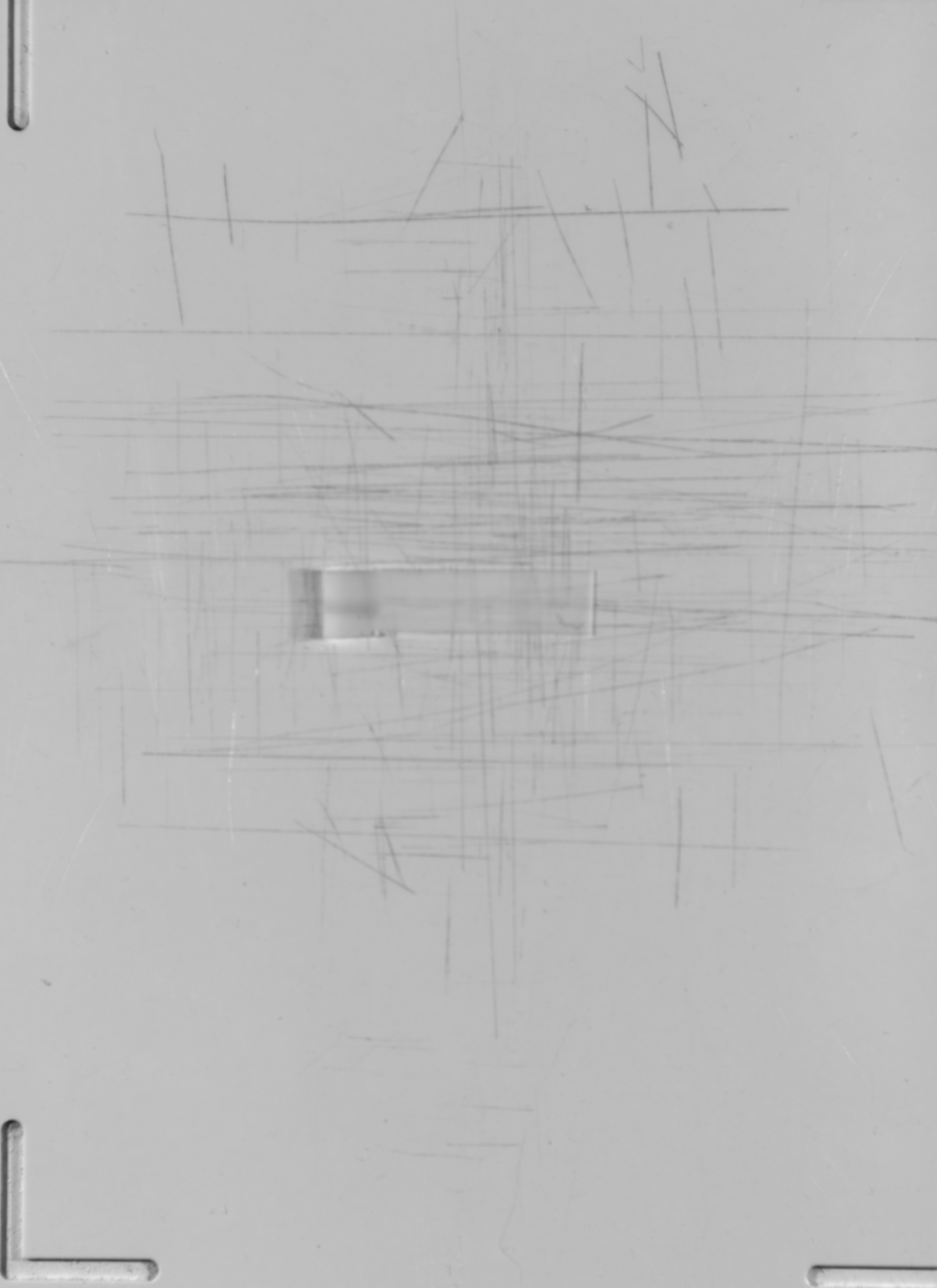

Supplement: Supplemental Information 4 [file peerj-11-15041-s004.zip › Enrichment-related genes-raw data2/AKT/AKT-2/AKT-2-4.tif]

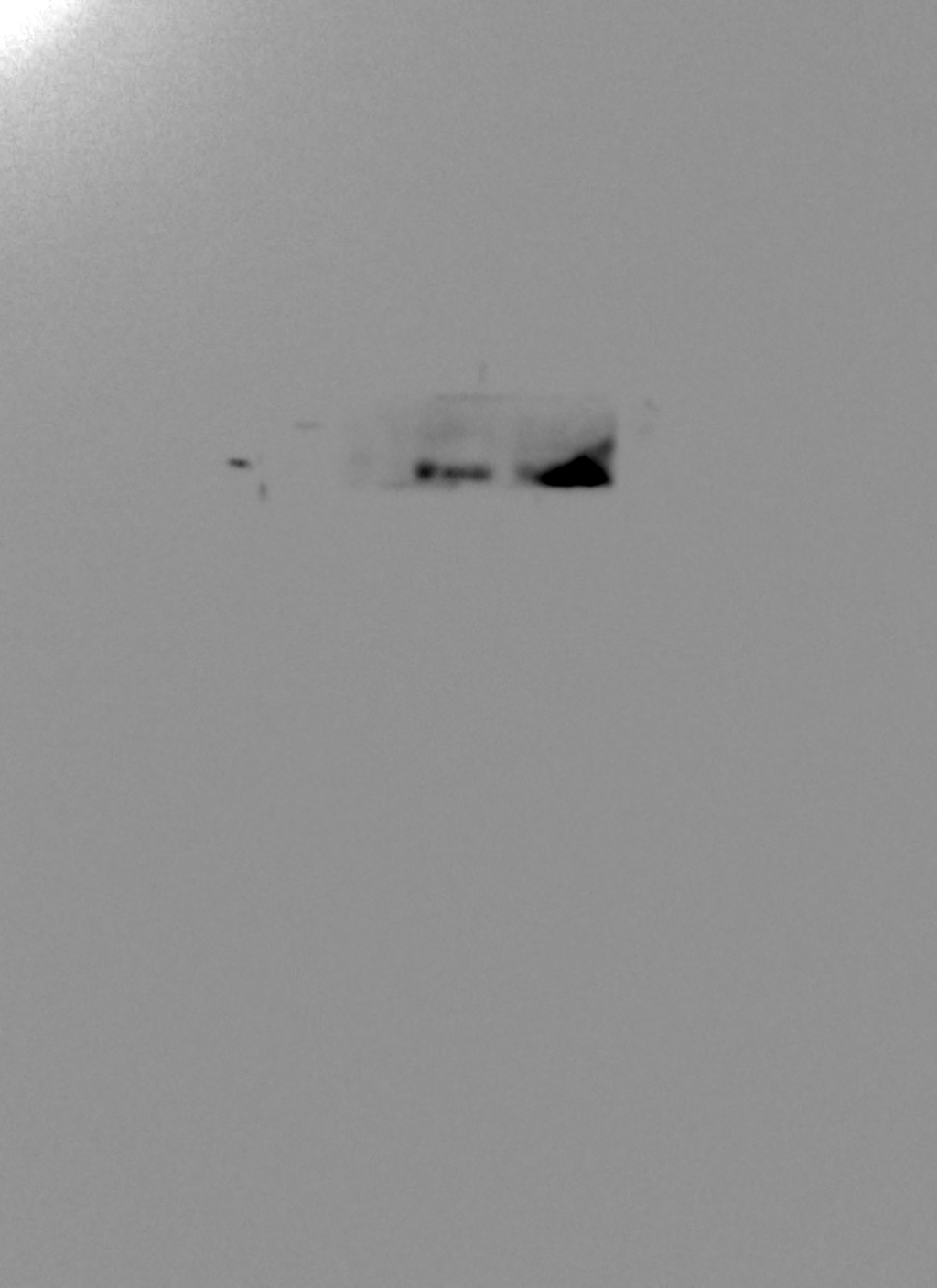

Supplement: Supplemental Information 4 [file peerj-11-15041-s004.zip › Enrichment-related genes-raw data2/AKT/AKT-3/AKT-3-1.tif]

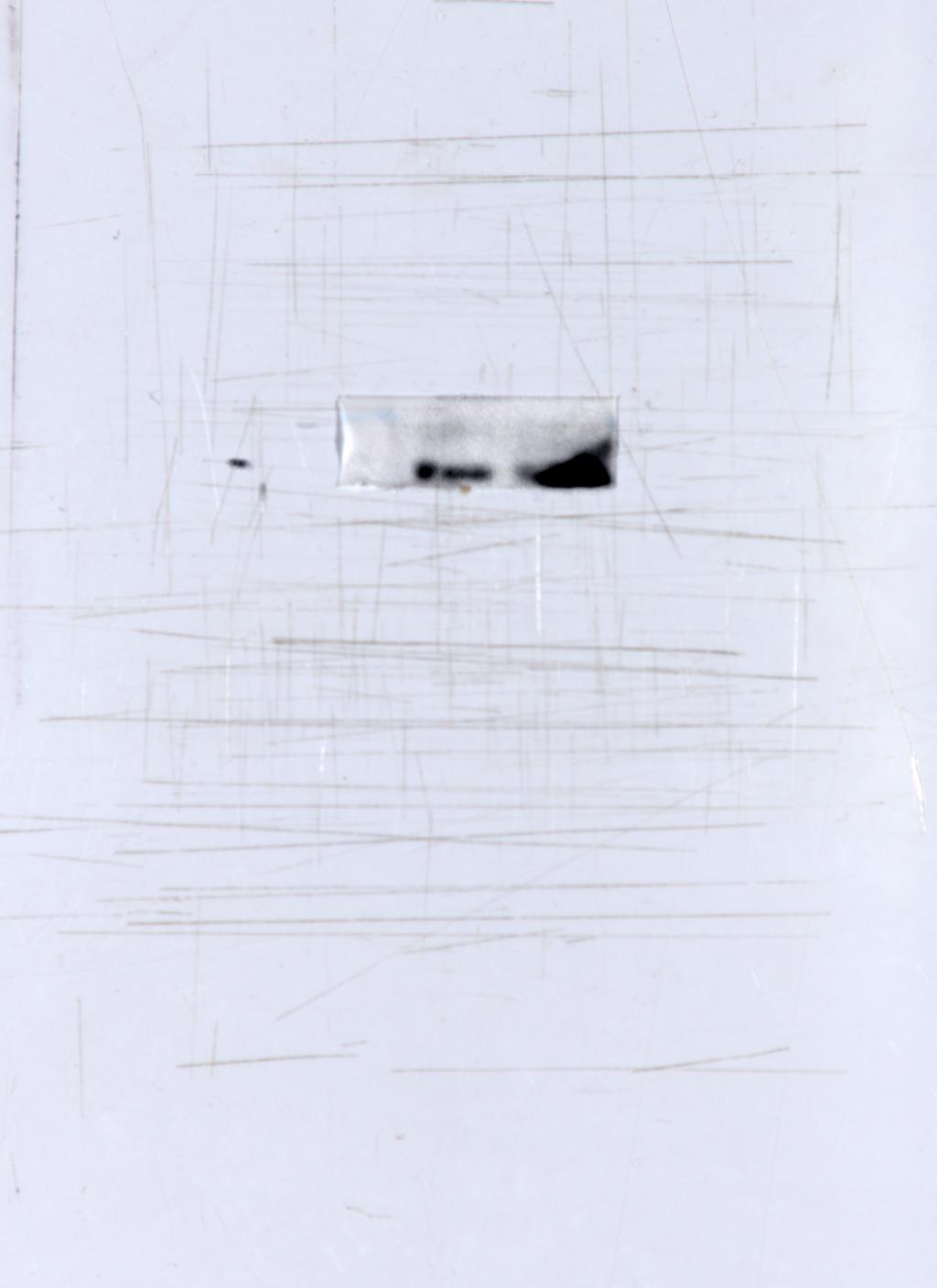

Supplement: Supplemental Information 4 [file peerj-11-15041-s004.zip › Enrichment-related genes-raw data2/AKT/AKT-3/AKT-3-2.jpg]

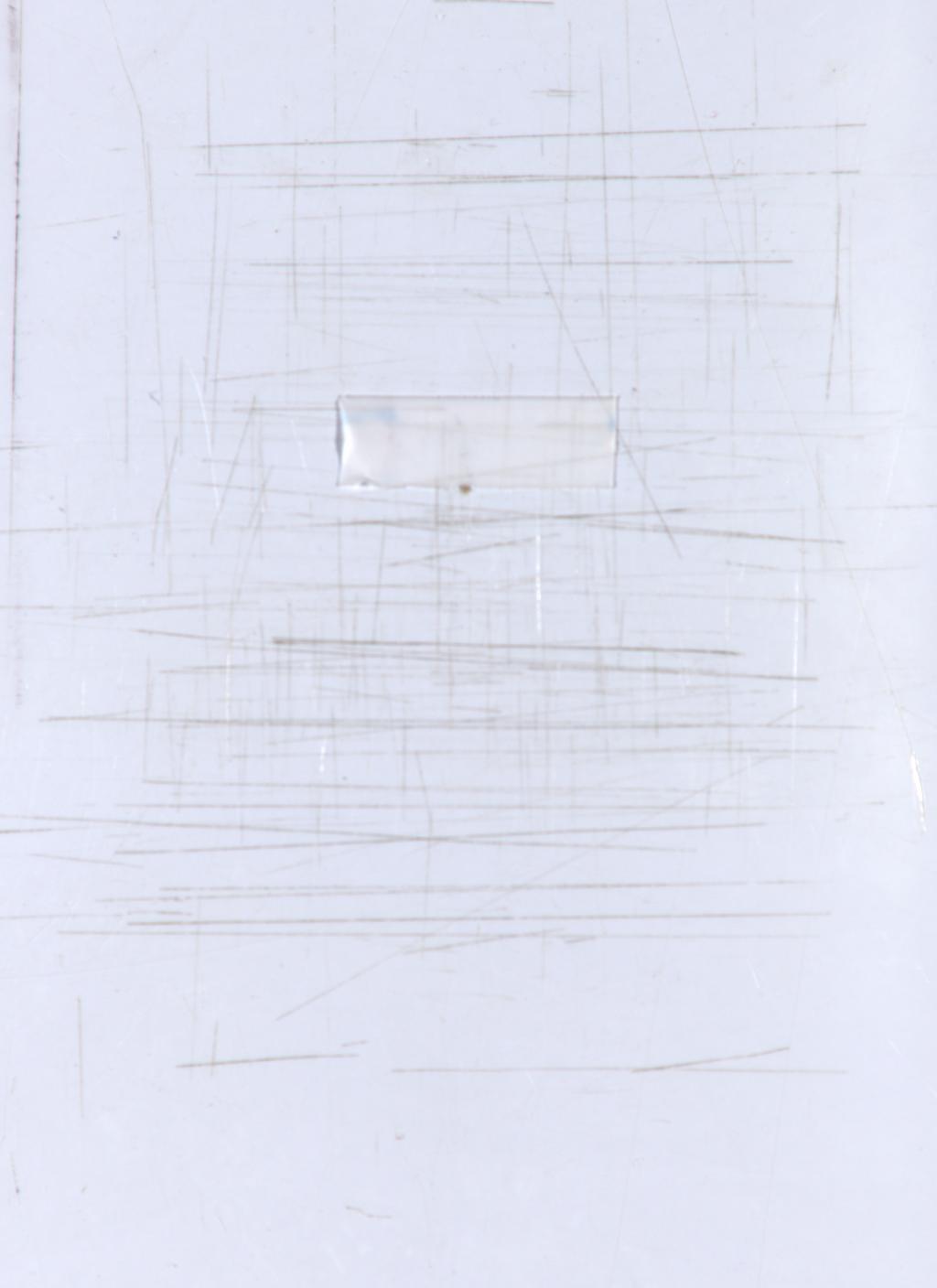

Supplement: Supplemental Information 4 [file peerj-11-15041-s004.zip › Enrichment-related genes-raw data2/AKT/AKT-3/AKT-3-3.jpg]

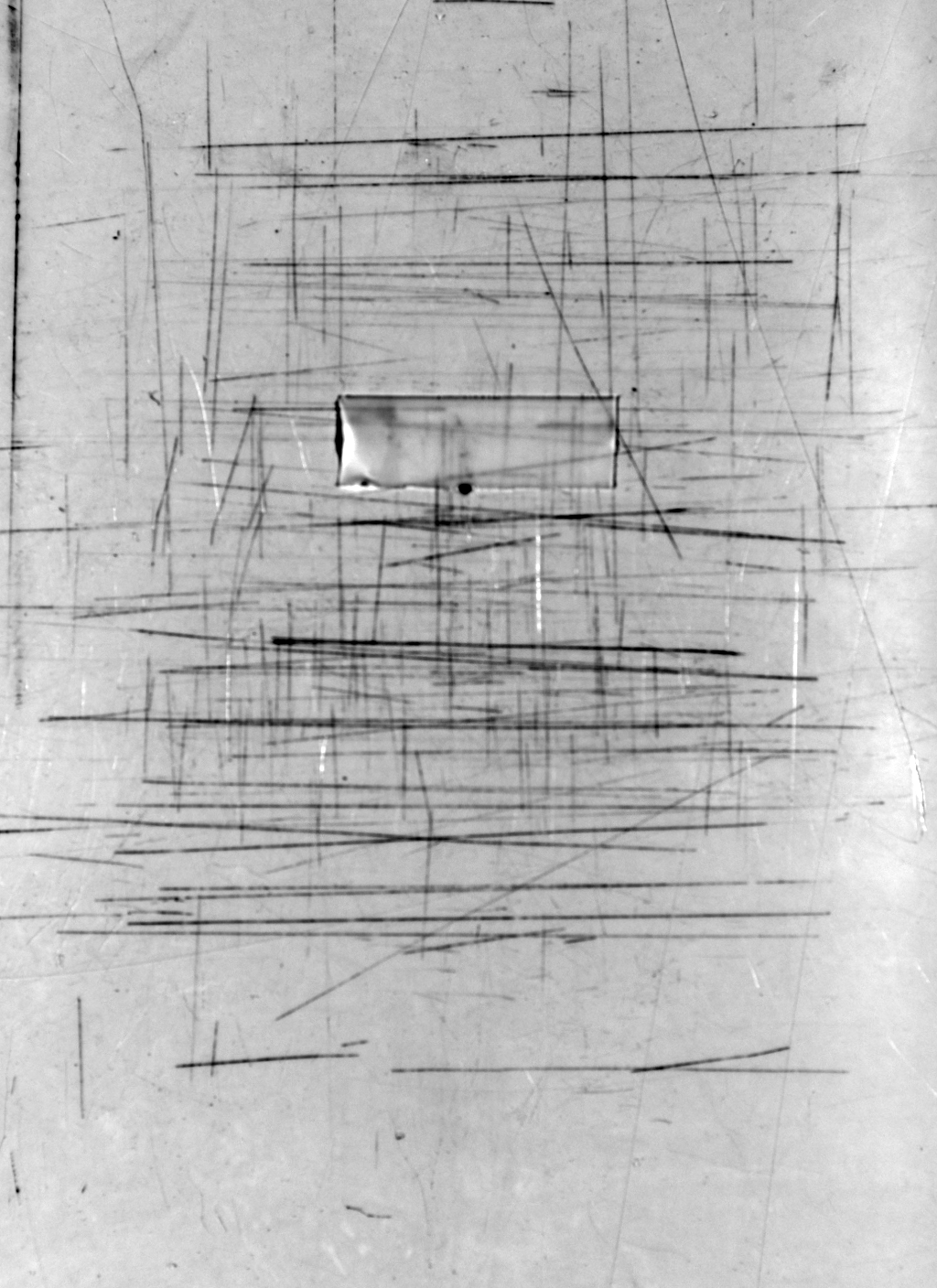

Supplement: Supplemental Information 4 [file peerj-11-15041-s004.zip › Enrichment-related genes-raw data2/AKT/AKT-3/AKT-3-4.tif]

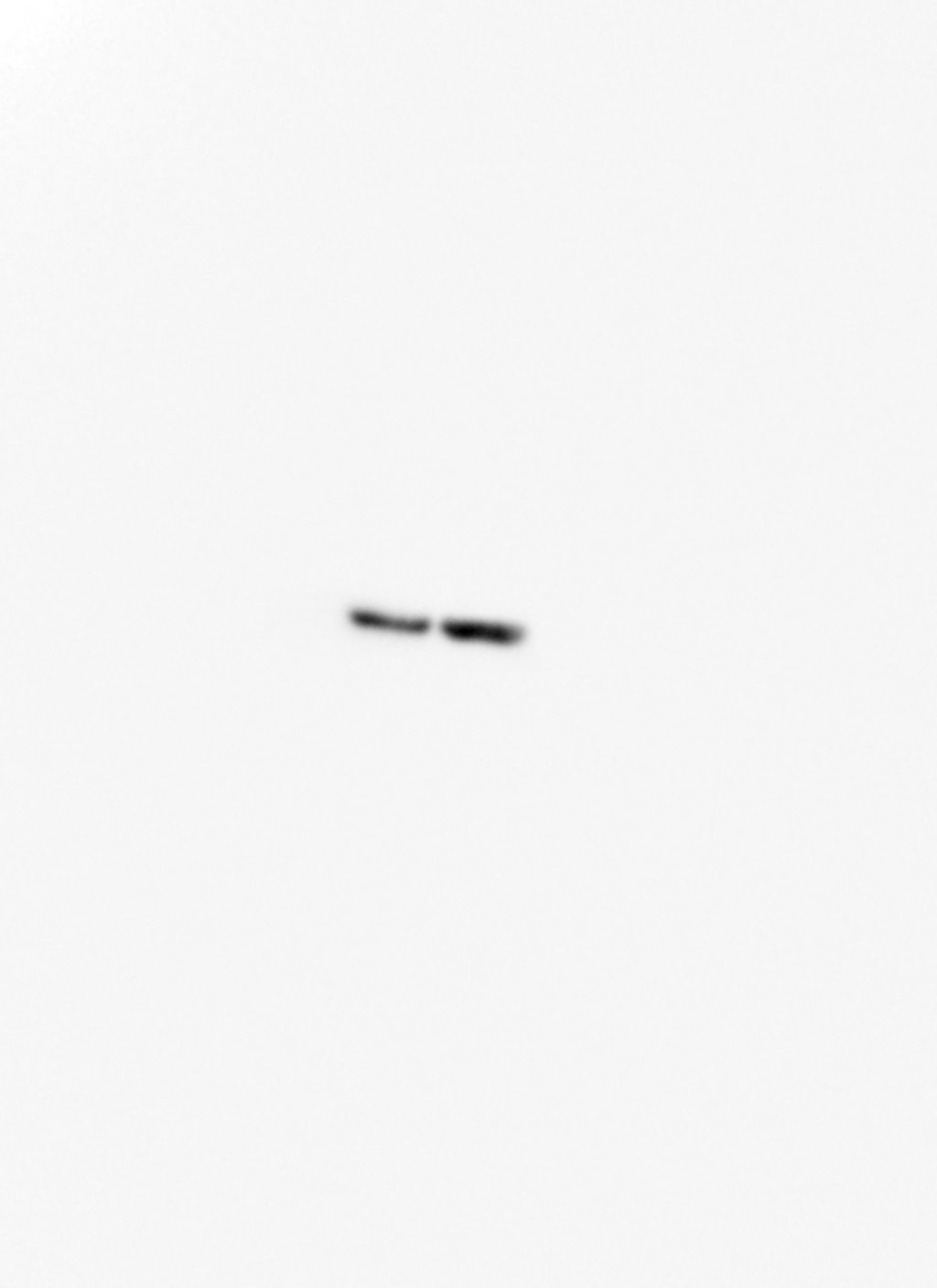

Supplement: Supplemental Information 4 [file peerj-11-15041-s004.zip › Enrichment-related genes-raw data2/PPAR-γ/PPAR-γ-1/PPAR-γ-1-1.tif]

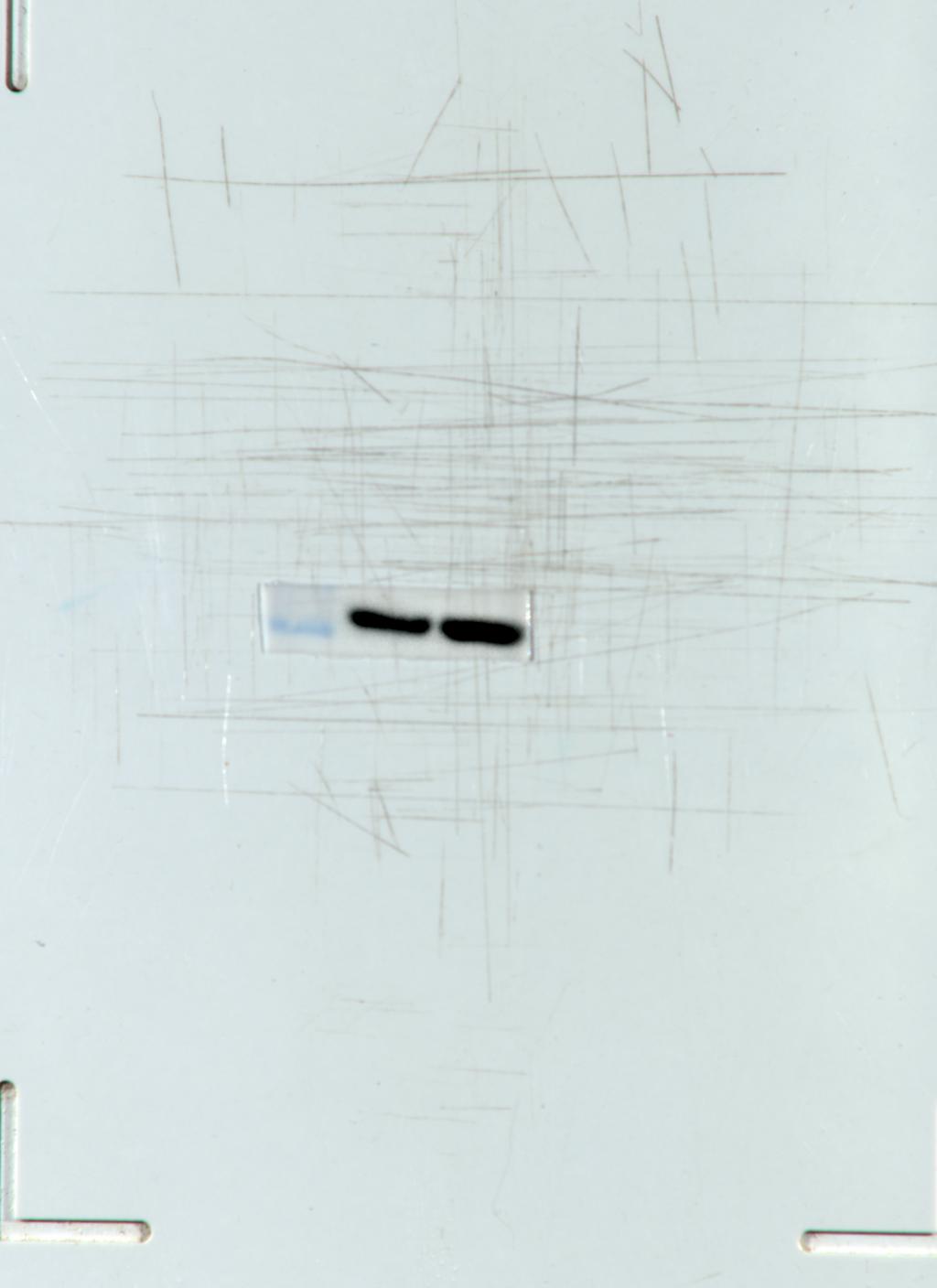

Supplement: Supplemental Information 4 [file peerj-11-15041-s004.zip › Enrichment-related genes-raw data2/PPAR-γ/PPAR-γ-1/PPAR-γ-1-2.jpg]

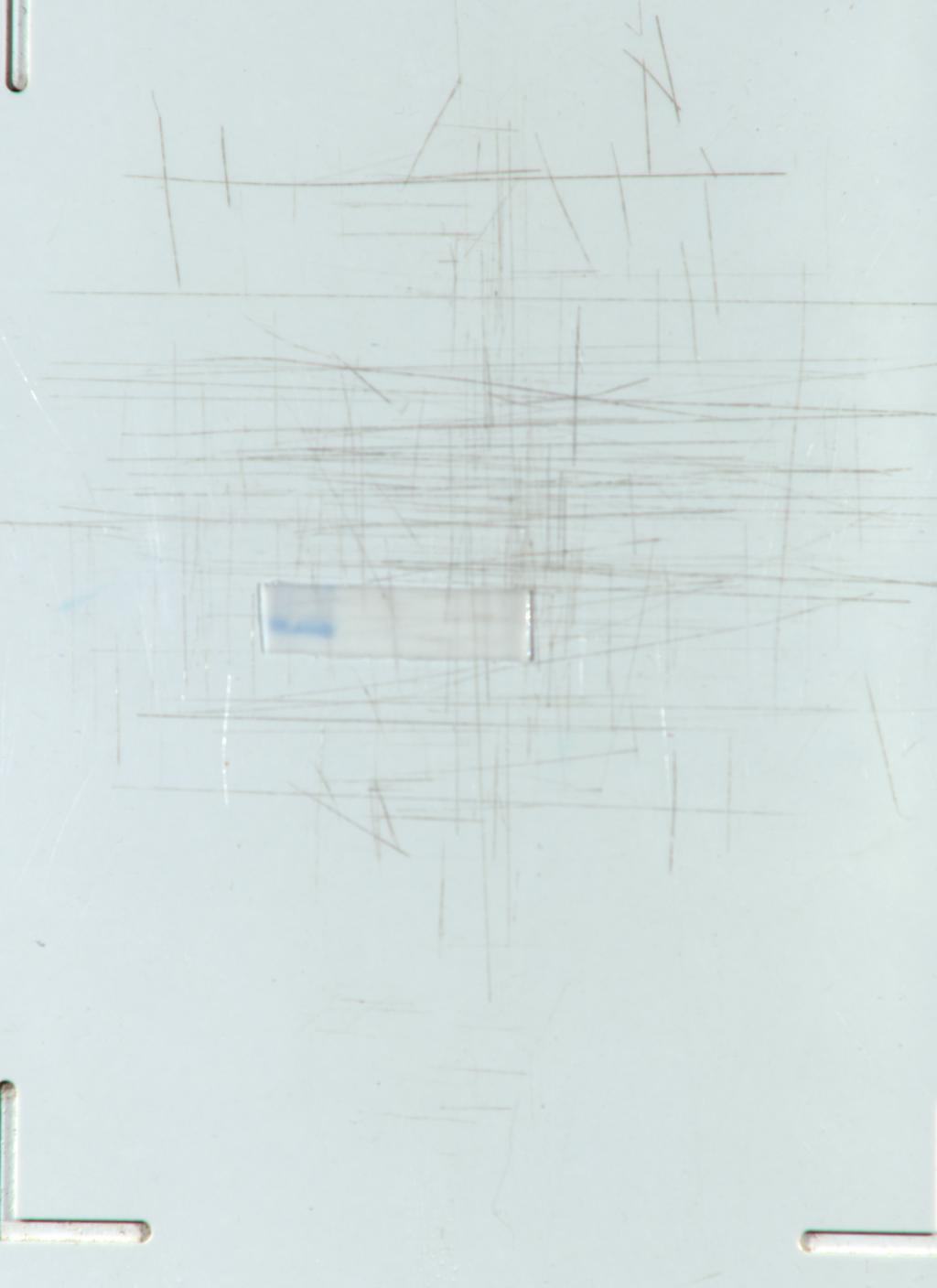

Supplement: Supplemental Information 4 [file peerj-11-15041-s004.zip › Enrichment-related genes-raw data2/PPAR-γ/PPAR-γ-1/PPAR-γ-1-3.jpg]

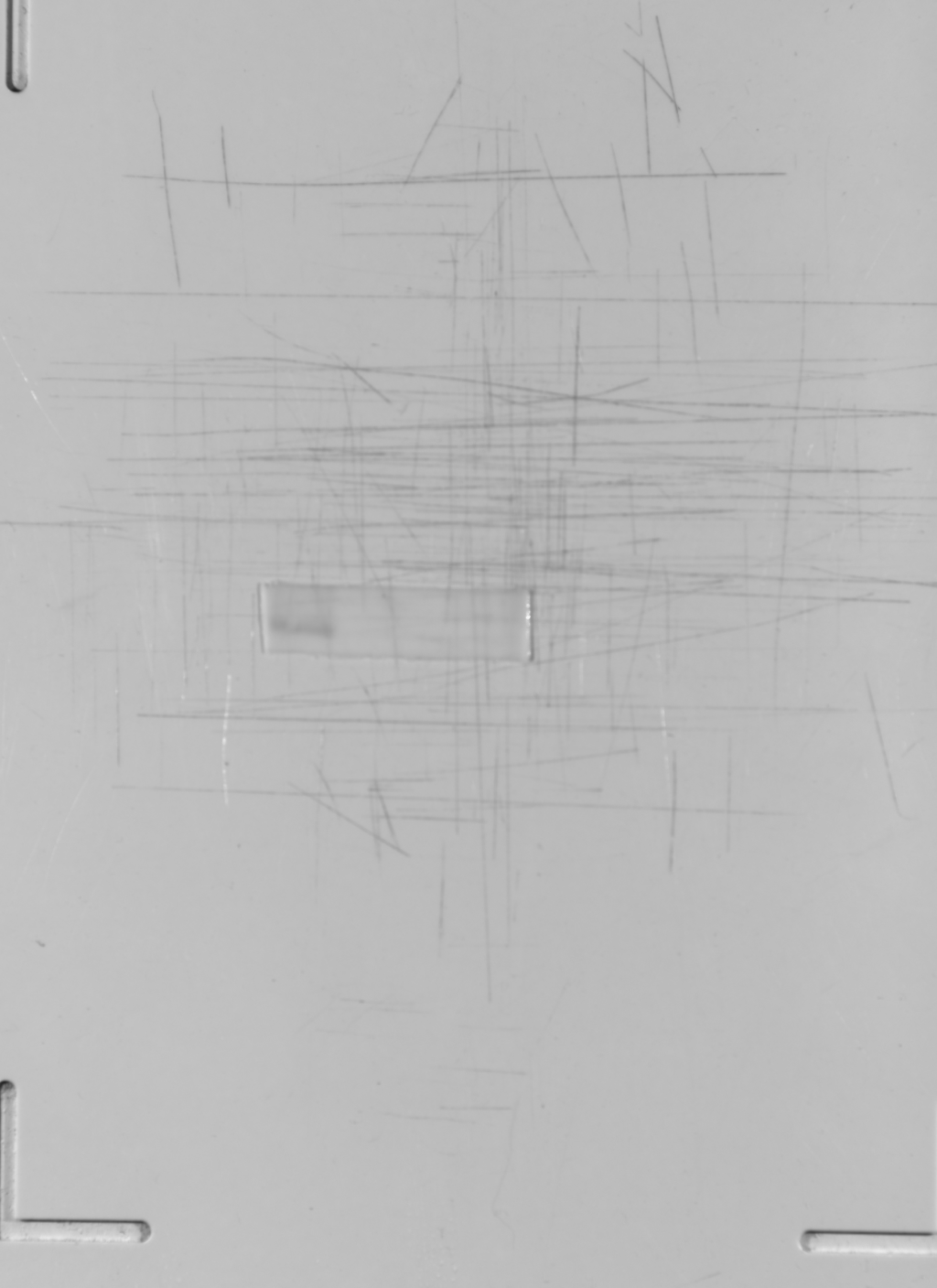

Supplement: Supplemental Information 4 [file peerj-11-15041-s004.zip › Enrichment-related genes-raw data2/PPAR-γ/PPAR-γ-1/PPAR-γ-1-4.tif]

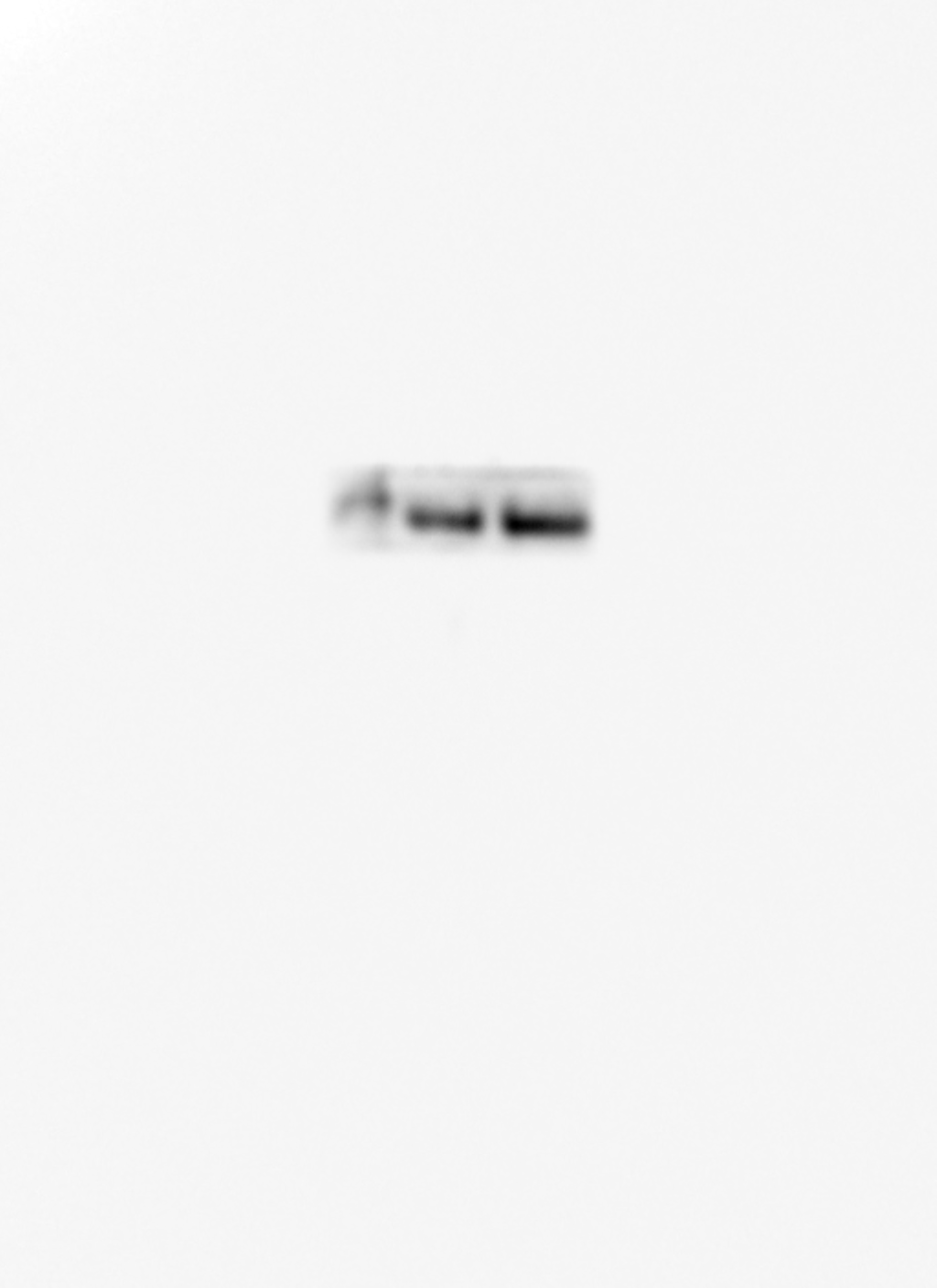

Supplement: Supplemental Information 4 [file peerj-11-15041-s004.zip › Enrichment-related genes-raw data2/PPAR-γ/PPAR-γ-2/PPAR-γ-2-1.tif]

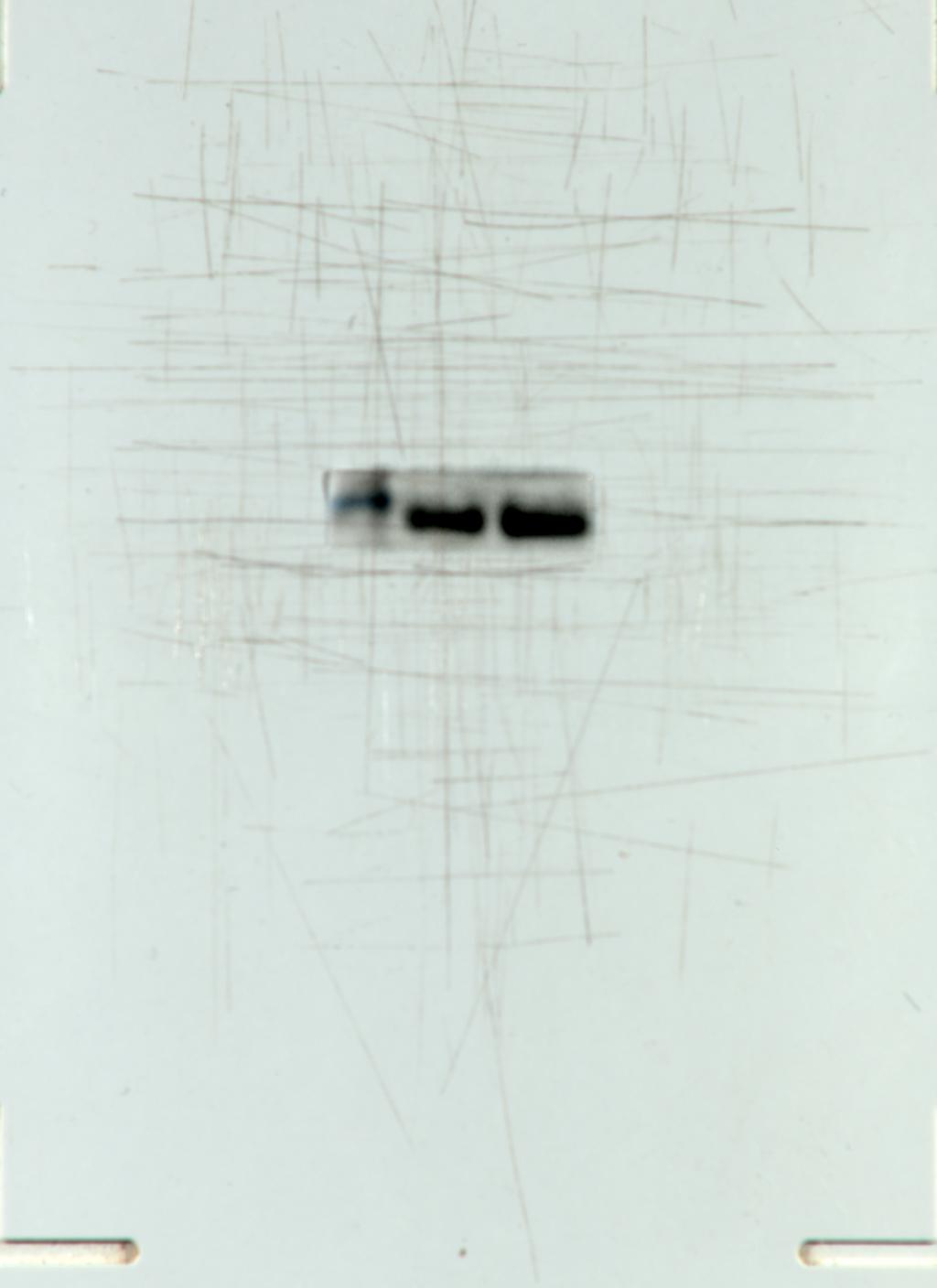

Supplement: Supplemental Information 4 [file peerj-11-15041-s004.zip › Enrichment-related genes-raw data2/PPAR-γ/PPAR-γ-2/PPAR-γ-2-2.jpg]

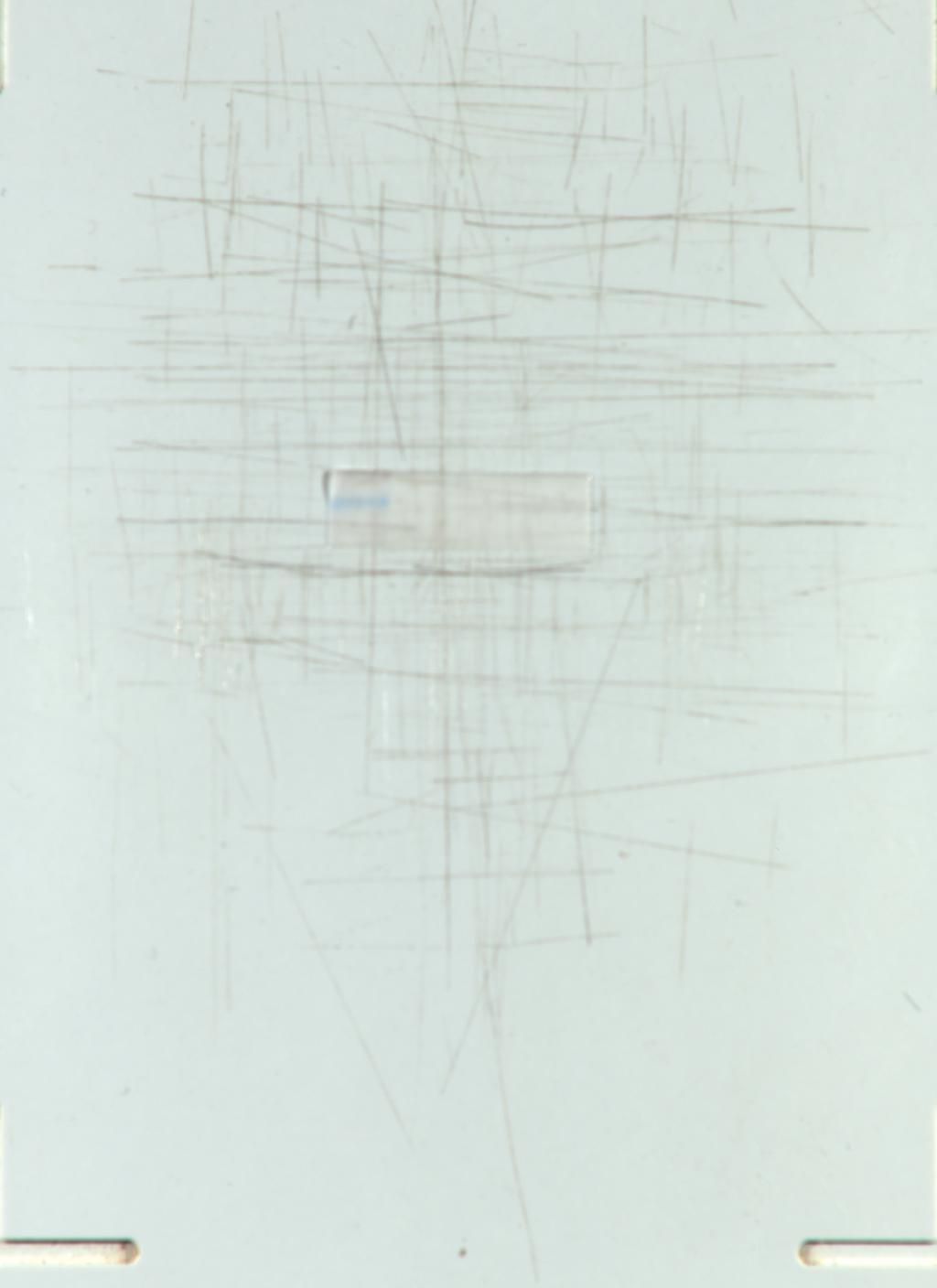

Supplement: Supplemental Information 4 [file peerj-11-15041-s004.zip › Enrichment-related genes-raw data2/PPAR-γ/PPAR-γ-2/PPAR-γ-2-3.jpg]

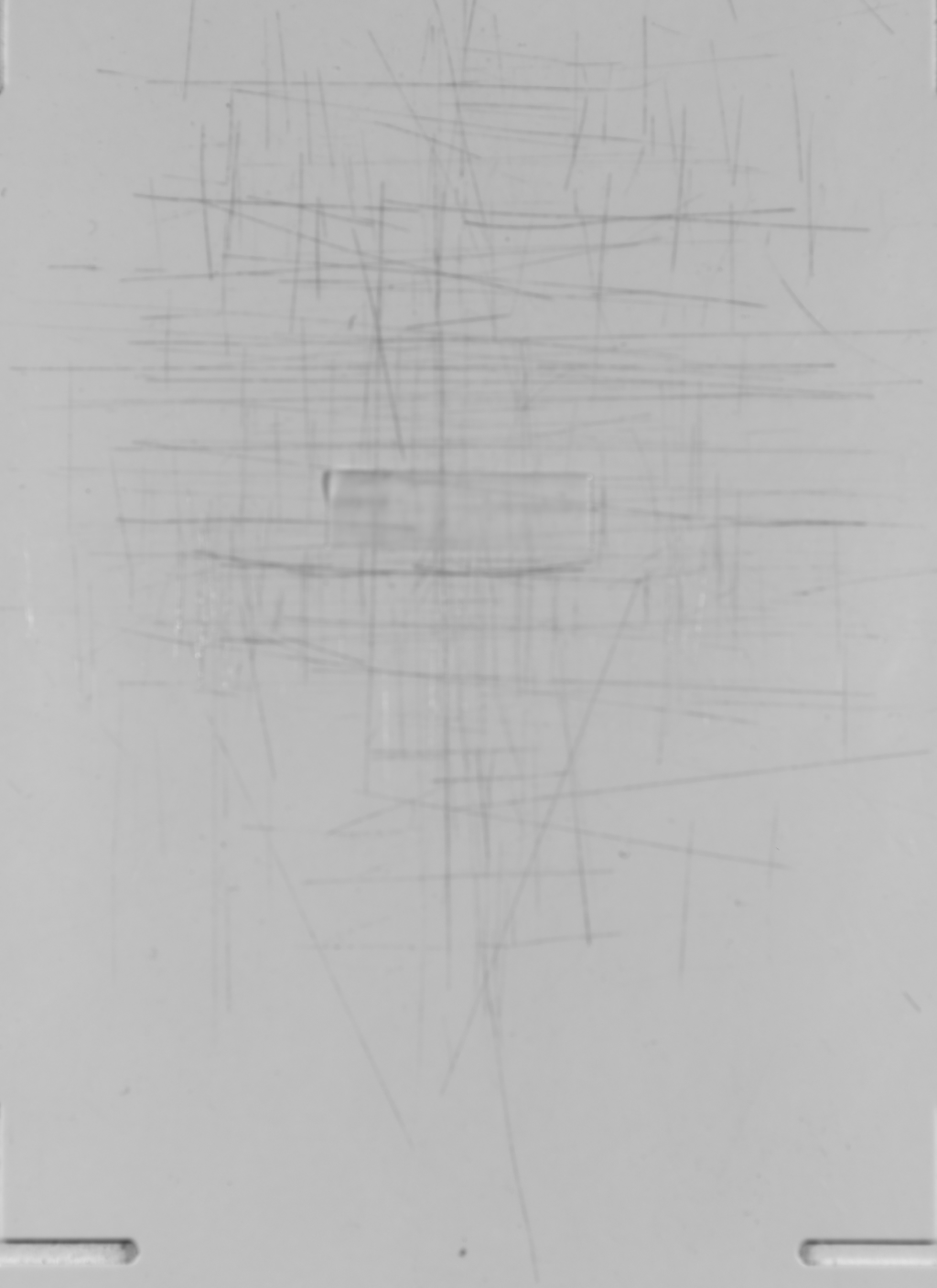

Supplement: Supplemental Information 4 [file peerj-11-15041-s004.zip › Enrichment-related genes-raw data2/PPAR-γ/PPAR-γ-2/PPAR-γ-2-4.tif]

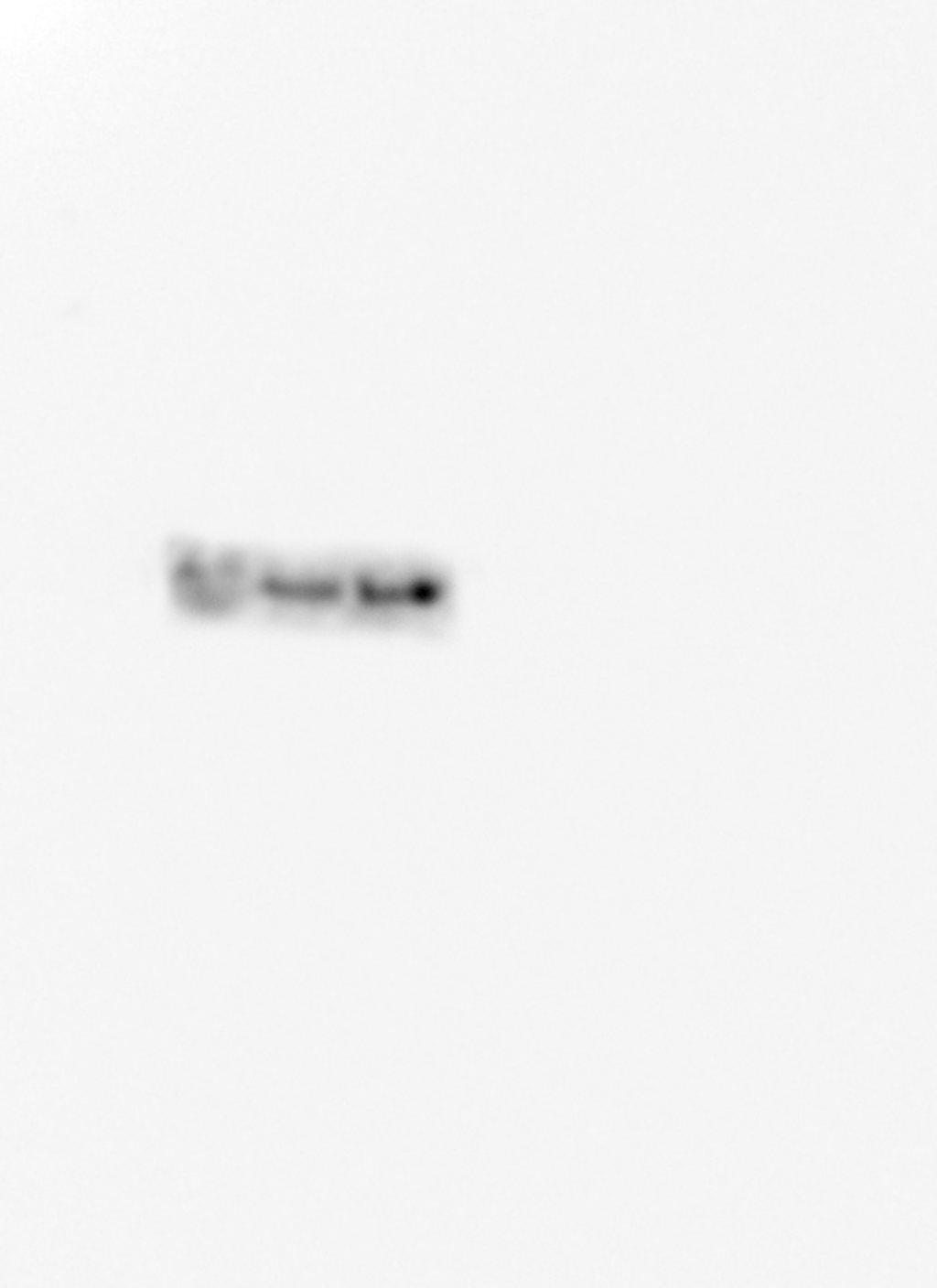

Supplement: Supplemental Information 4 [file peerj-11-15041-s004.zip › Enrichment-related genes-raw data2/PPAR-γ/PPAR-γ-3/PPAR-γ-3-1.tif]

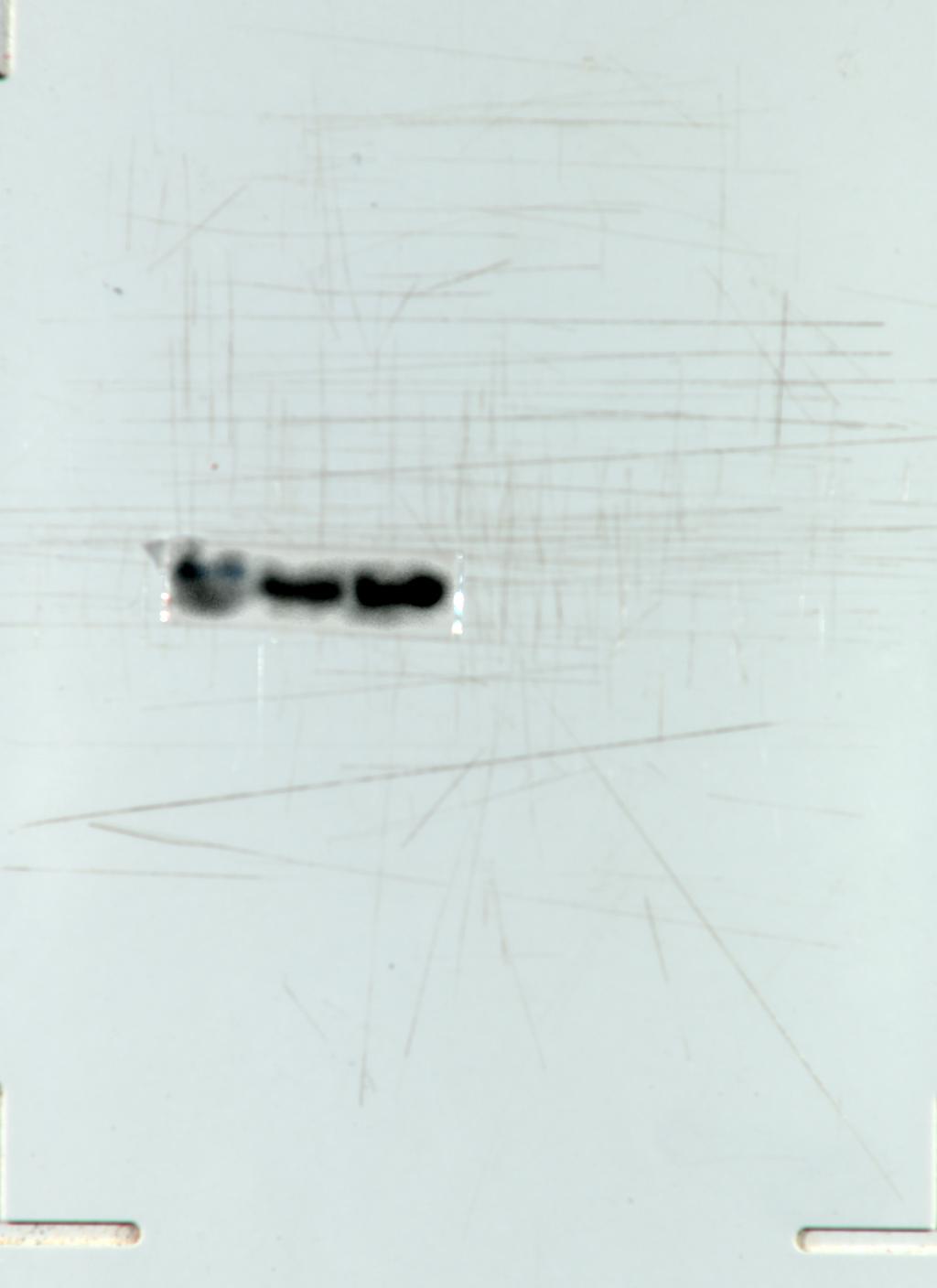

Supplement: Supplemental Information 4 [file peerj-11-15041-s004.zip › Enrichment-related genes-raw data2/PPAR-γ/PPAR-γ-3/PPAR-γ-3-2.jpg]

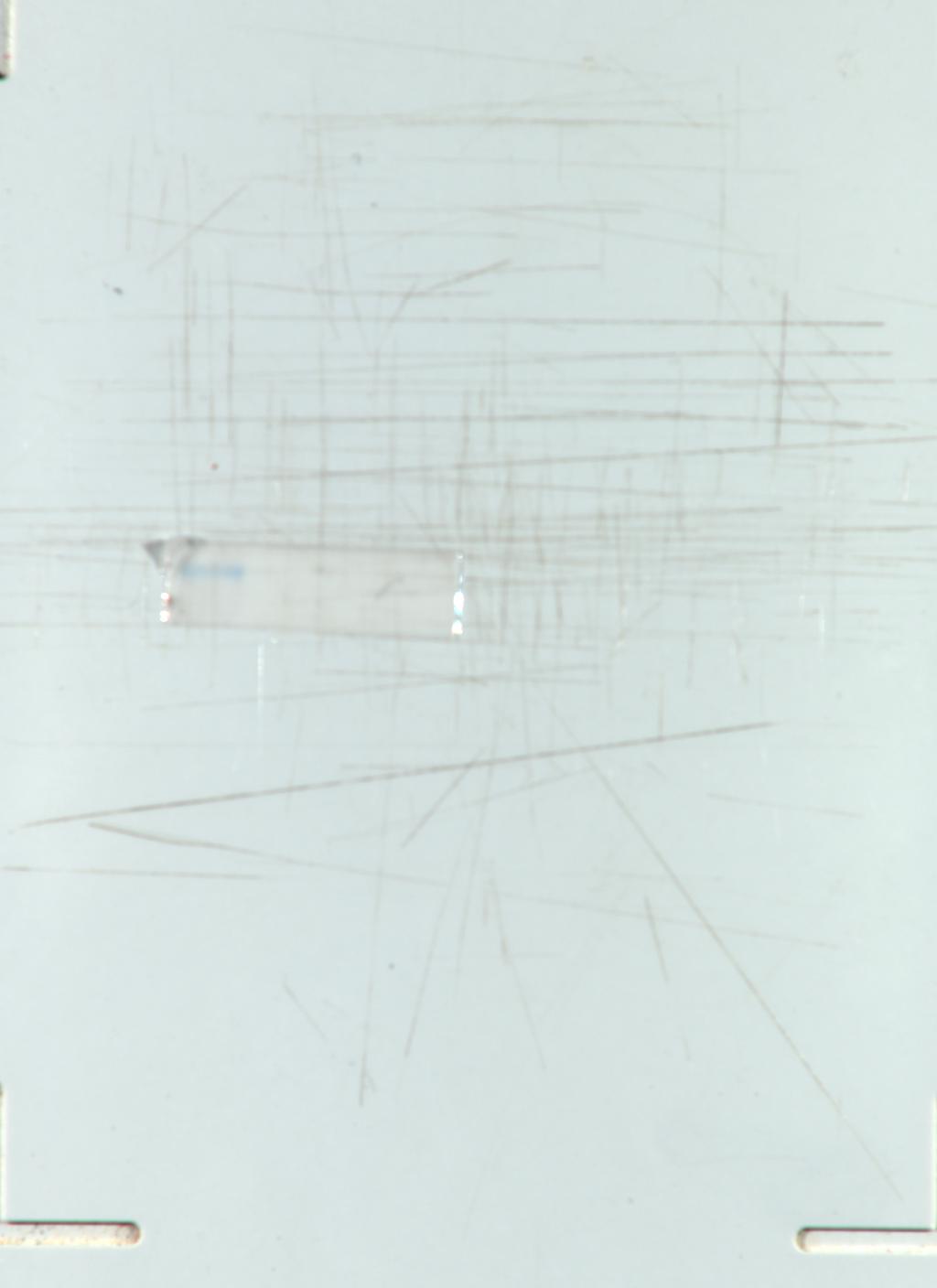

Supplement: Supplemental Information 4 [file peerj-11-15041-s004.zip › Enrichment-related genes-raw data2/PPAR-γ/PPAR-γ-3/PPAR-γ-3-3.jpg]

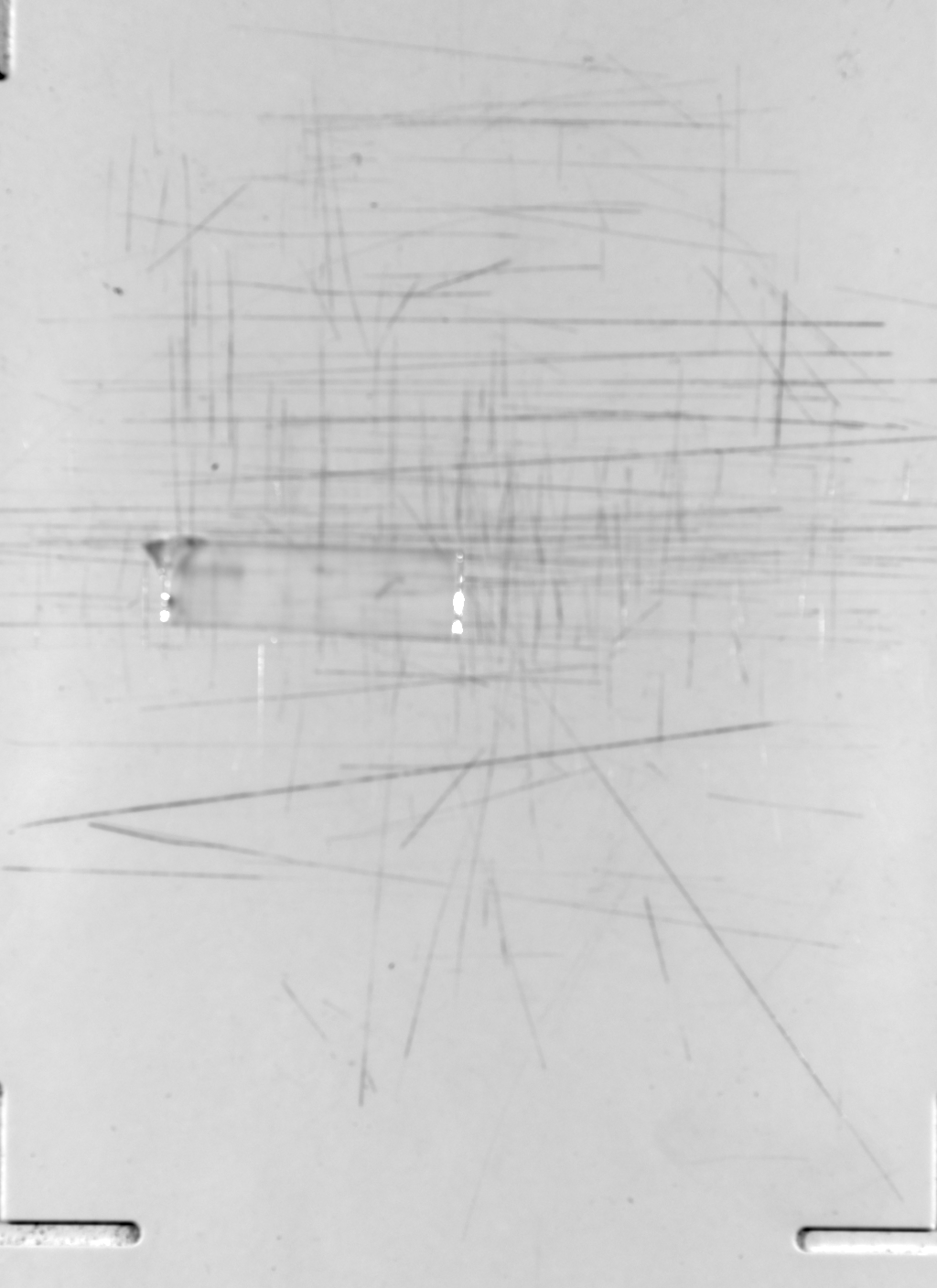

Supplement: Supplemental Information 4 [file peerj-11-15041-s004.zip › Enrichment-related genes-raw data2/PPAR-γ/PPAR-γ-3/PPAR-γ-3-4.tif]

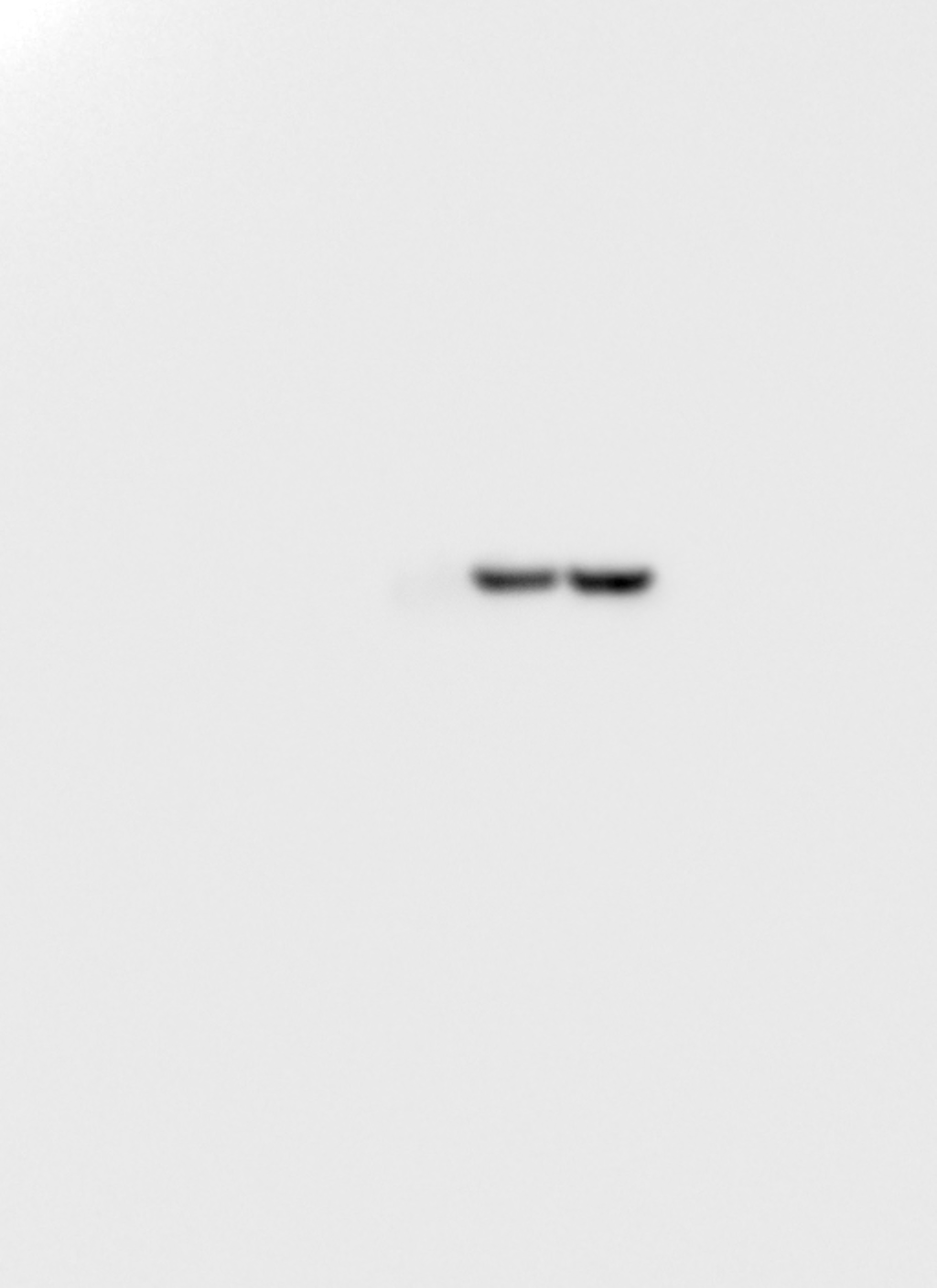

Supplement: Supplemental Information 5 [file peerj-11-15041-s005.zip › Osteoclast-related-genes-raw data1/ACTIN/ACTIN-1/ACTIN-1-1.tif]

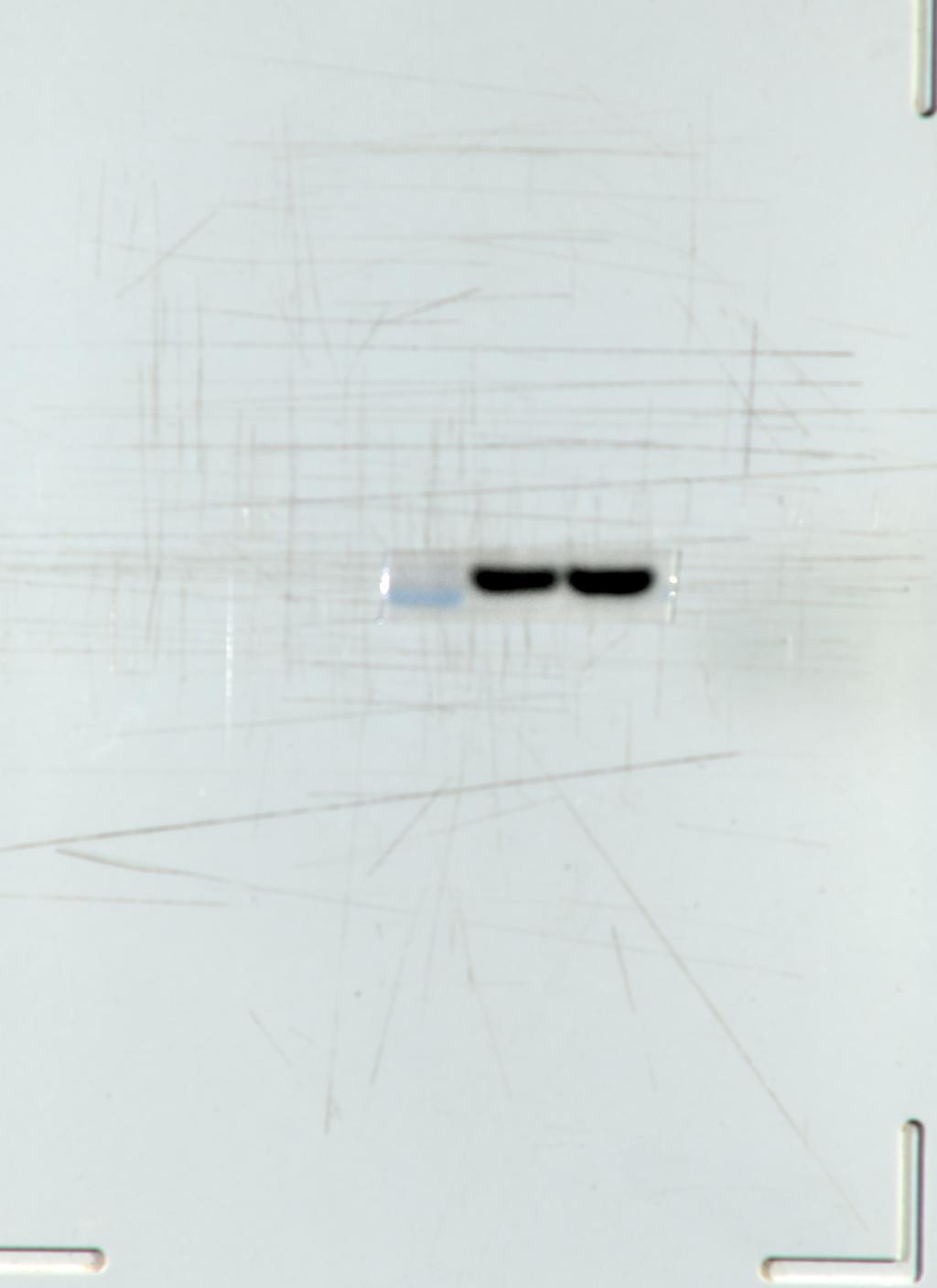

Supplement: Supplemental Information 5 [file peerj-11-15041-s005.zip › Osteoclast-related-genes-raw data1/ACTIN/ACTIN-1/ACTIN-1-2.jpg]

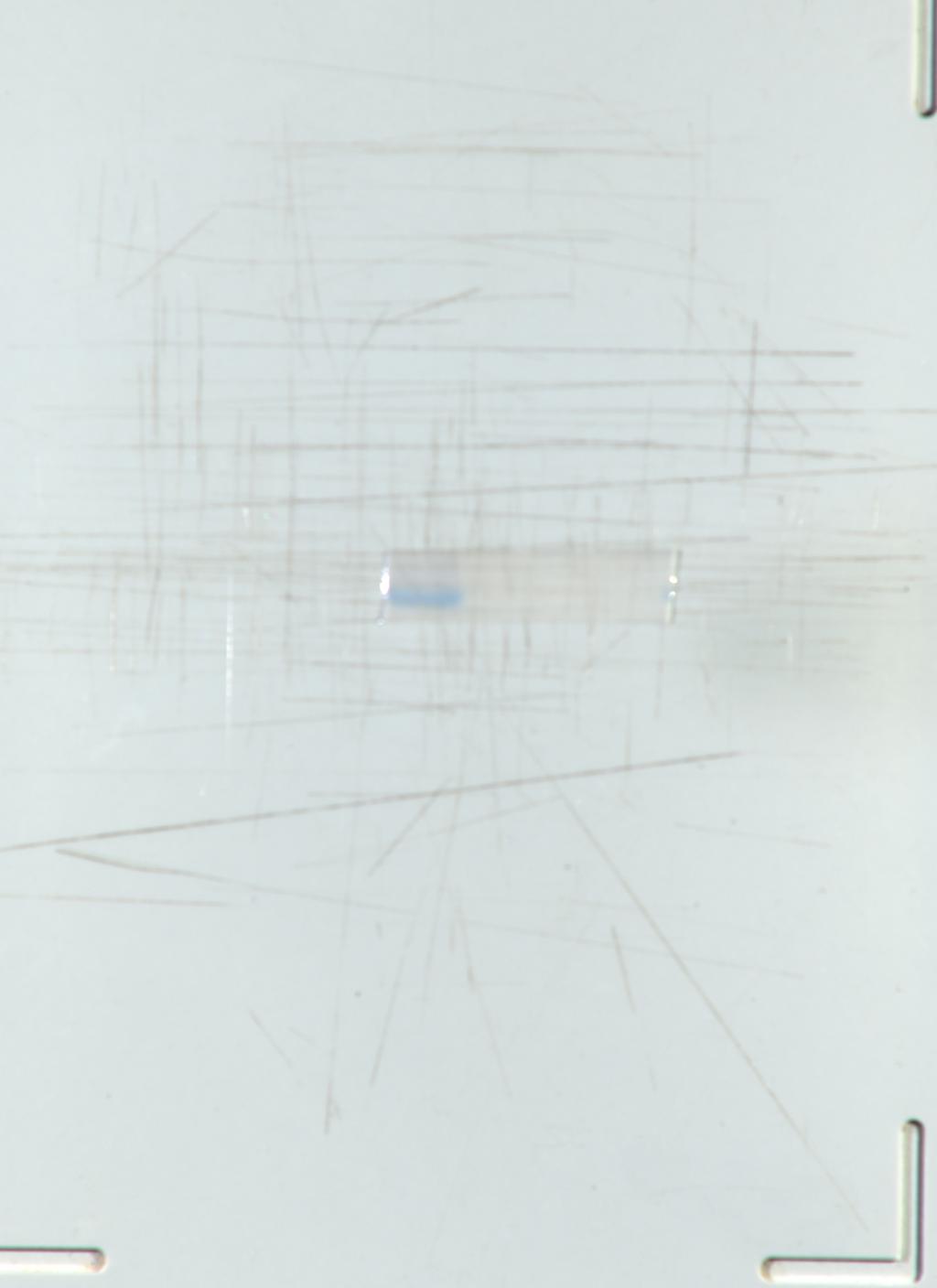

Supplement: Supplemental Information 5 [file peerj-11-15041-s005.zip › Osteoclast-related-genes-raw data1/ACTIN/ACTIN-1/ACTIN-1-3.jpg]

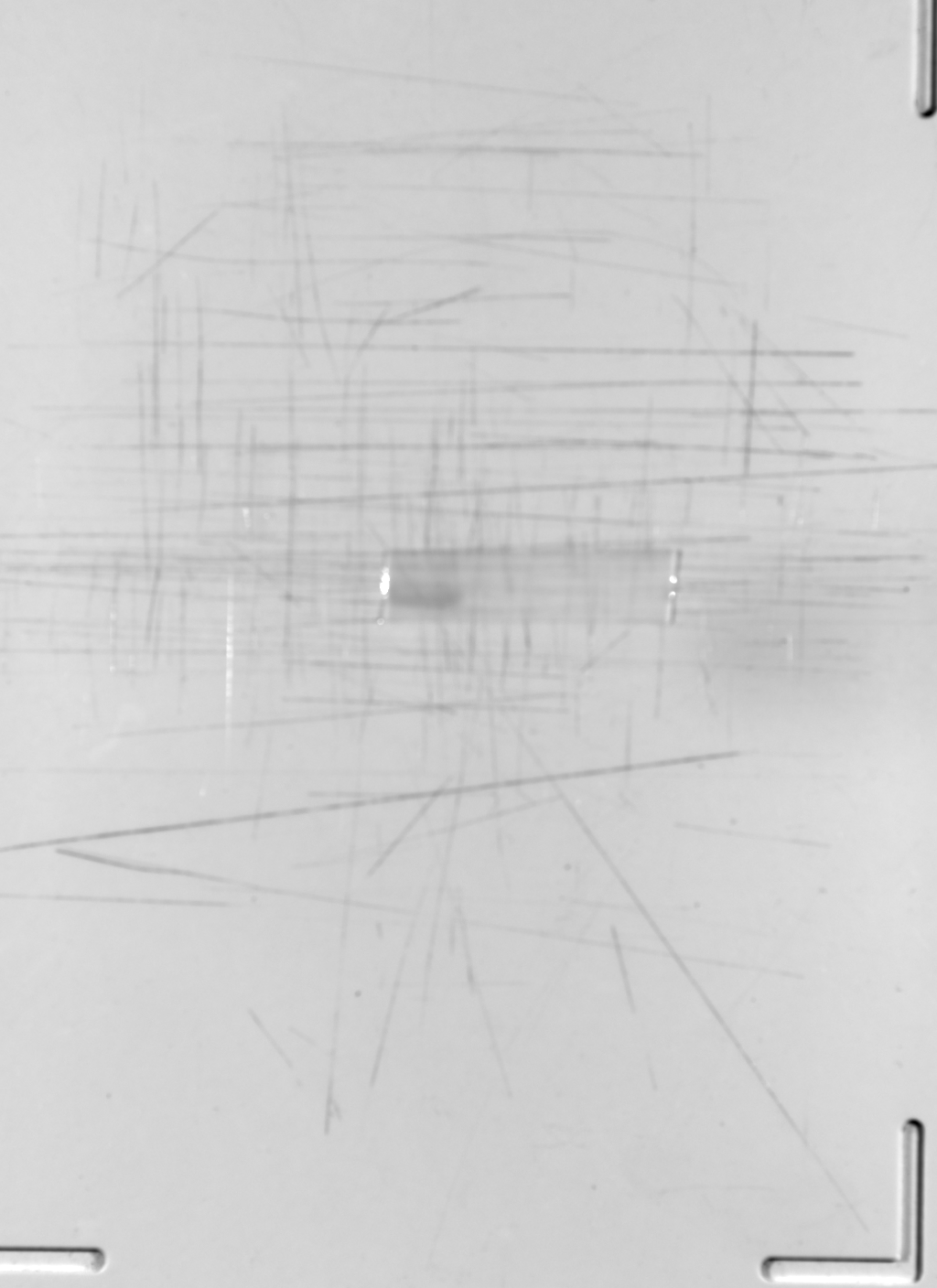

Supplement: Supplemental Information 5 [file peerj-11-15041-s005.zip › Osteoclast-related-genes-raw data1/ACTIN/ACTIN-1/ACTIN-1-4.tif]

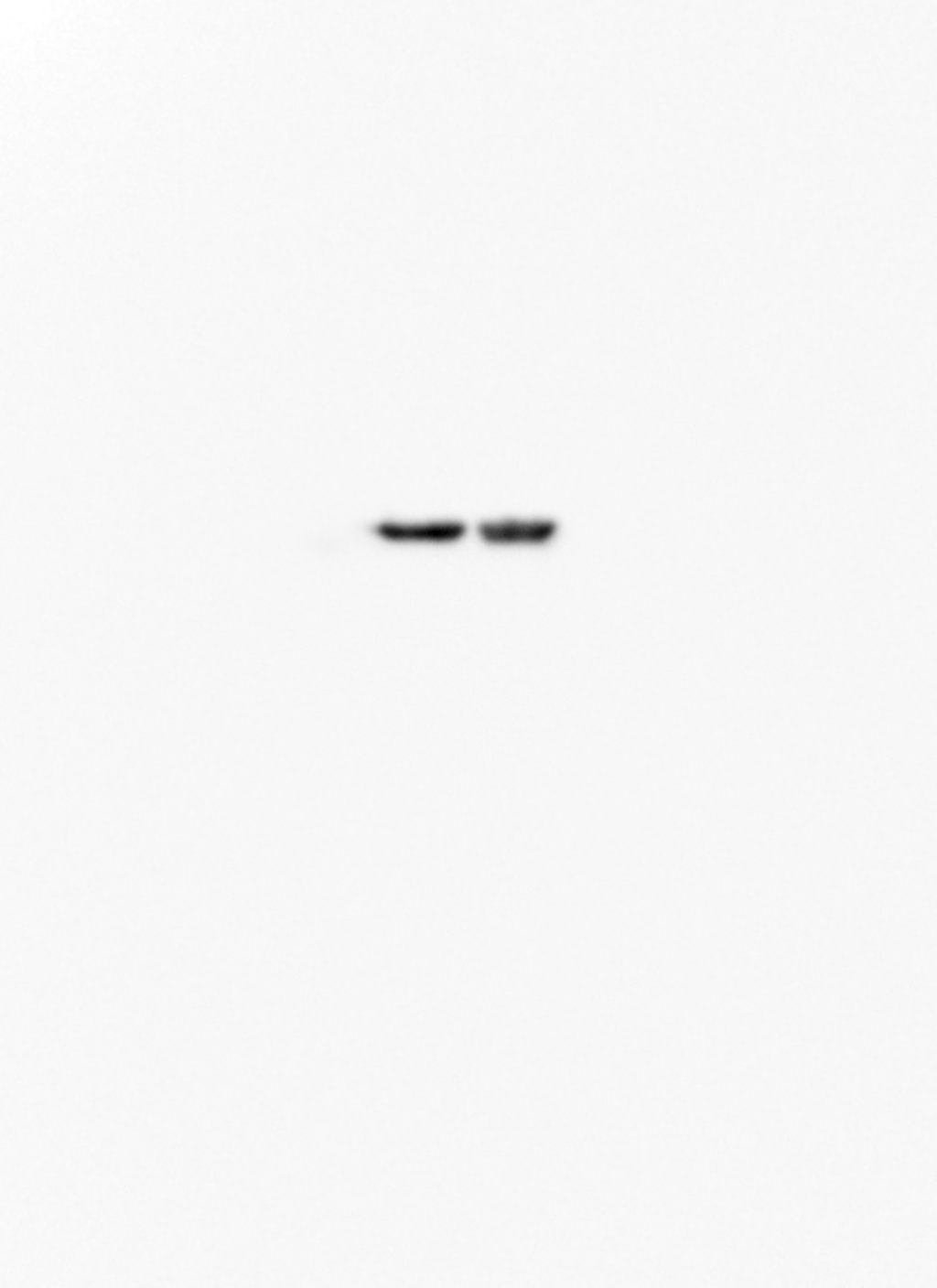

Supplement: Supplemental Information 5 [file peerj-11-15041-s005.zip › Osteoclast-related-genes-raw data1/ACTIN/ACTIN-2/ACTIN-2-1.tif]

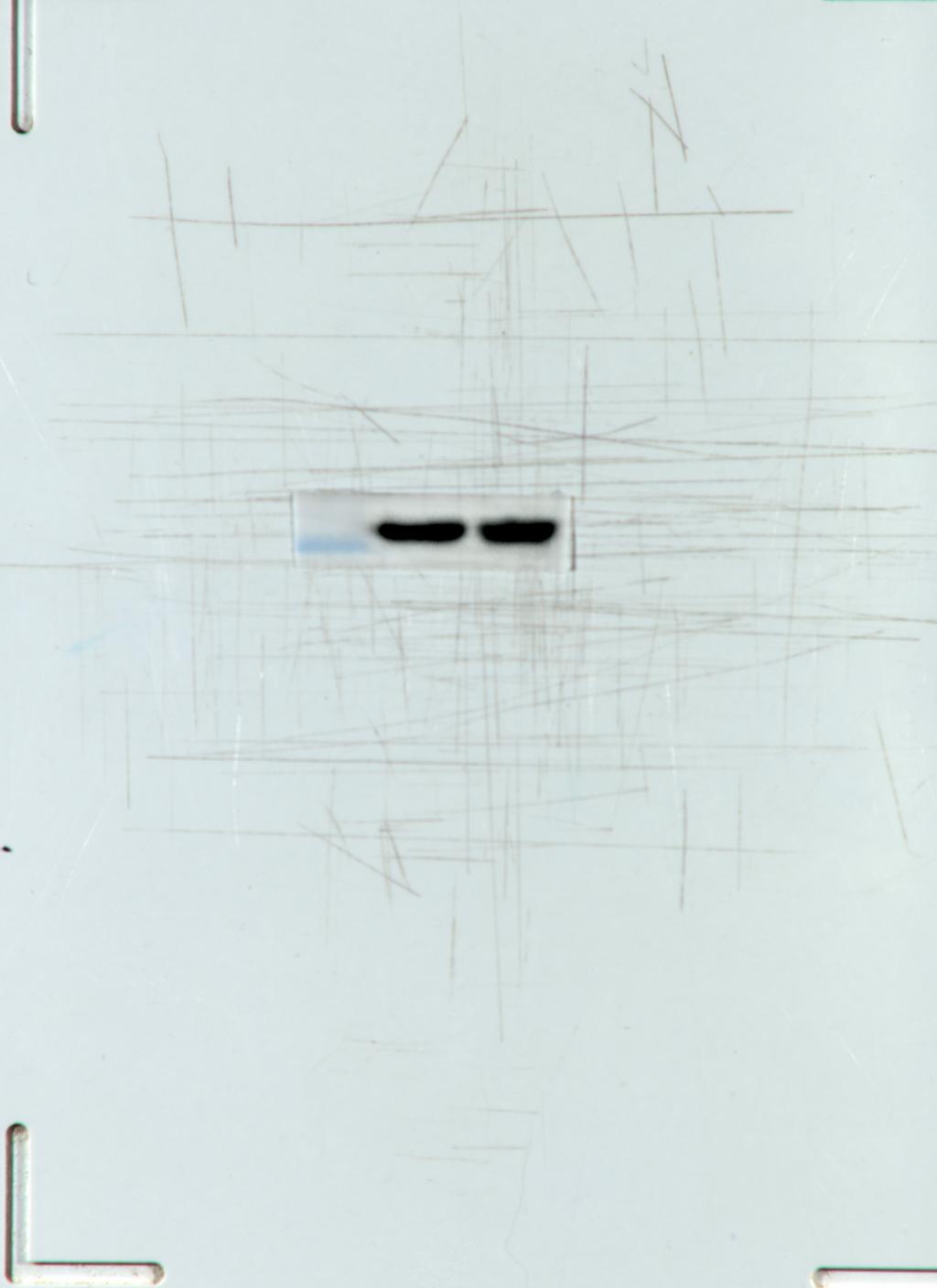

Supplement: Supplemental Information 5 [file peerj-11-15041-s005.zip › Osteoclast-related-genes-raw data1/ACTIN/ACTIN-2/ACTIN-2-2.jpg]

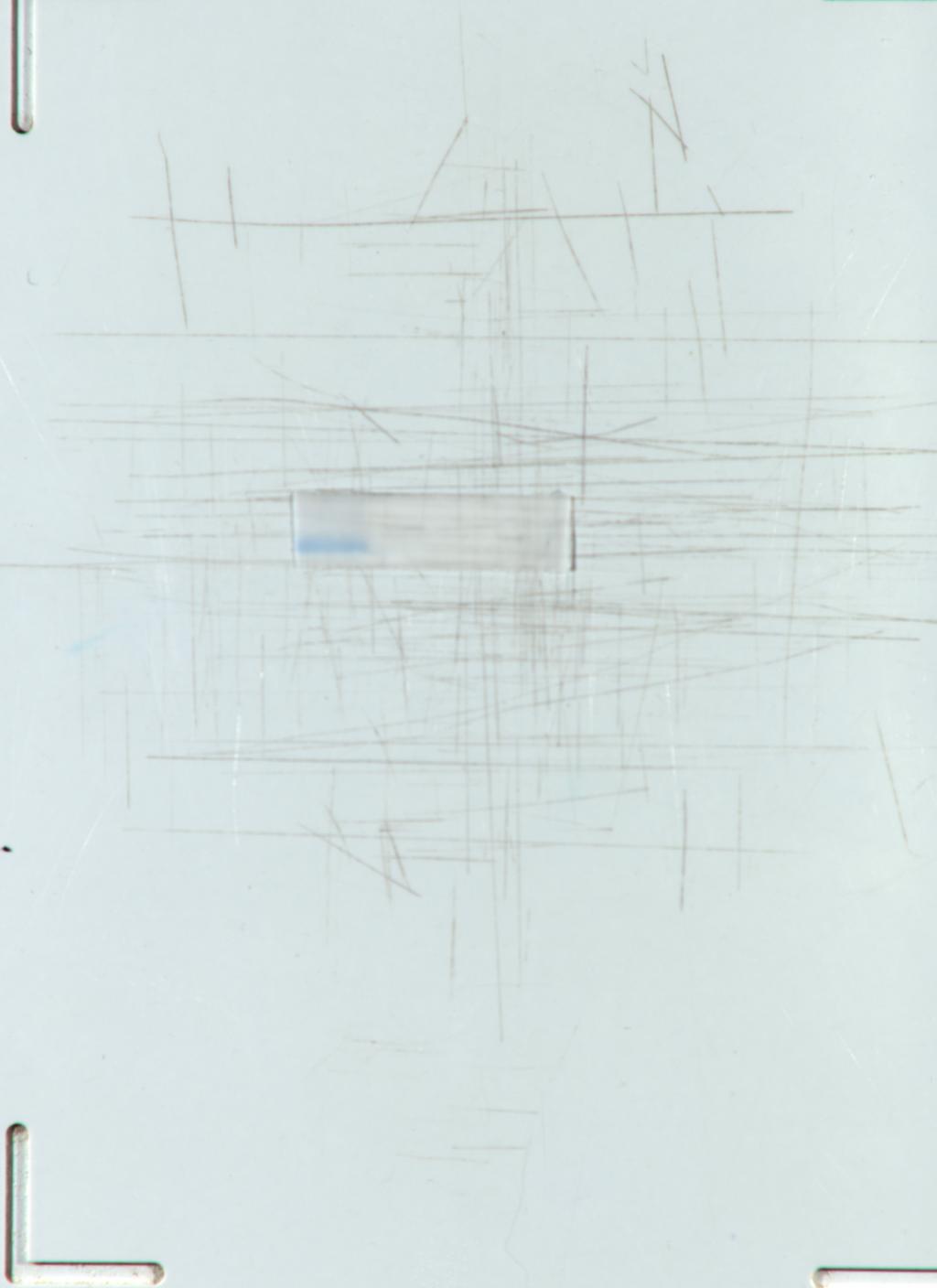

Supplement: Supplemental Information 5 [file peerj-11-15041-s005.zip › Osteoclast-related-genes-raw data1/ACTIN/ACTIN-2/ACTIN-2-3.jpg]

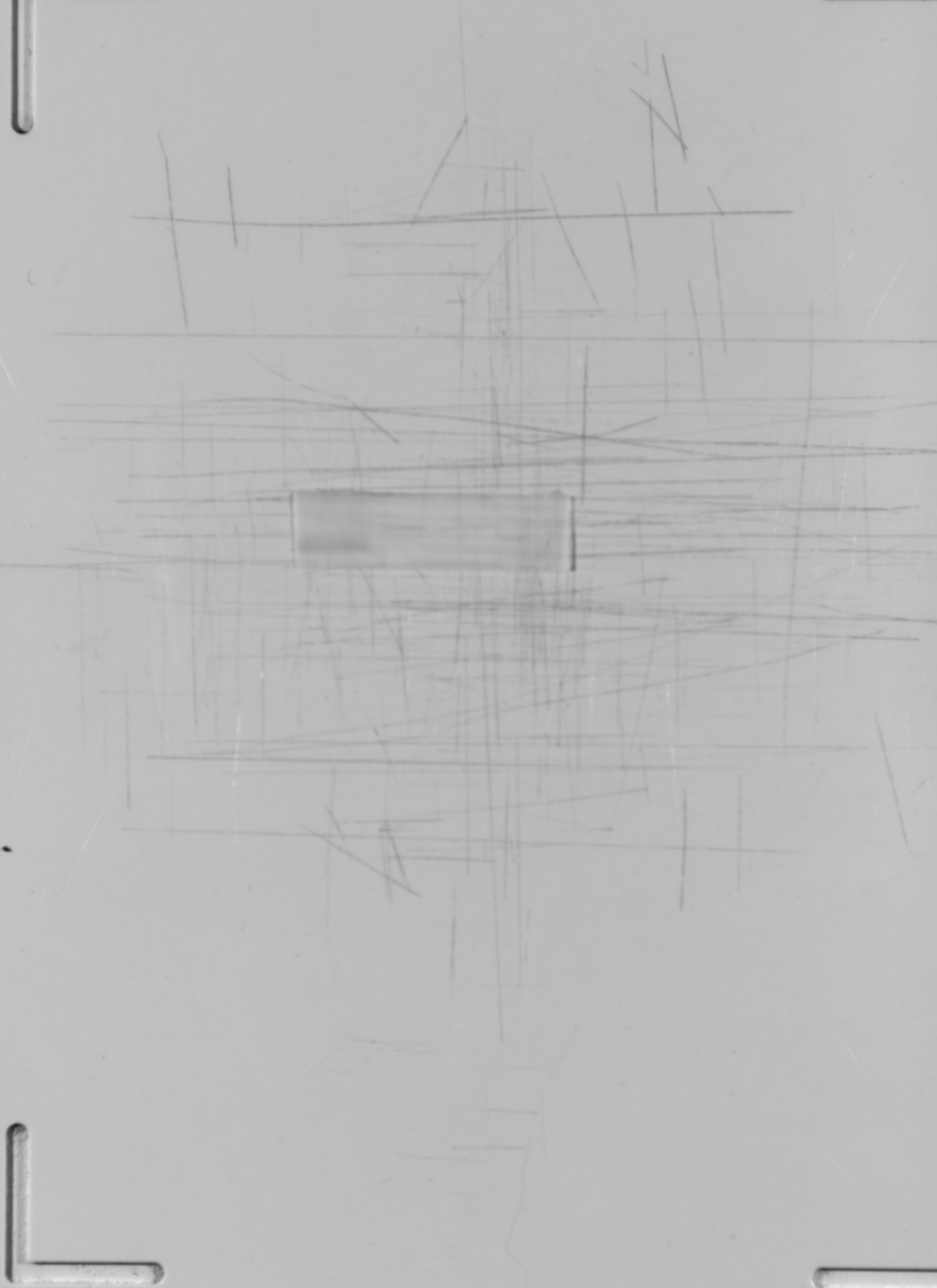

Supplement: Supplemental Information 5 [file peerj-11-15041-s005.zip › Osteoclast-related-genes-raw data1/ACTIN/ACTIN-2/ACTIN-2-4.tif]

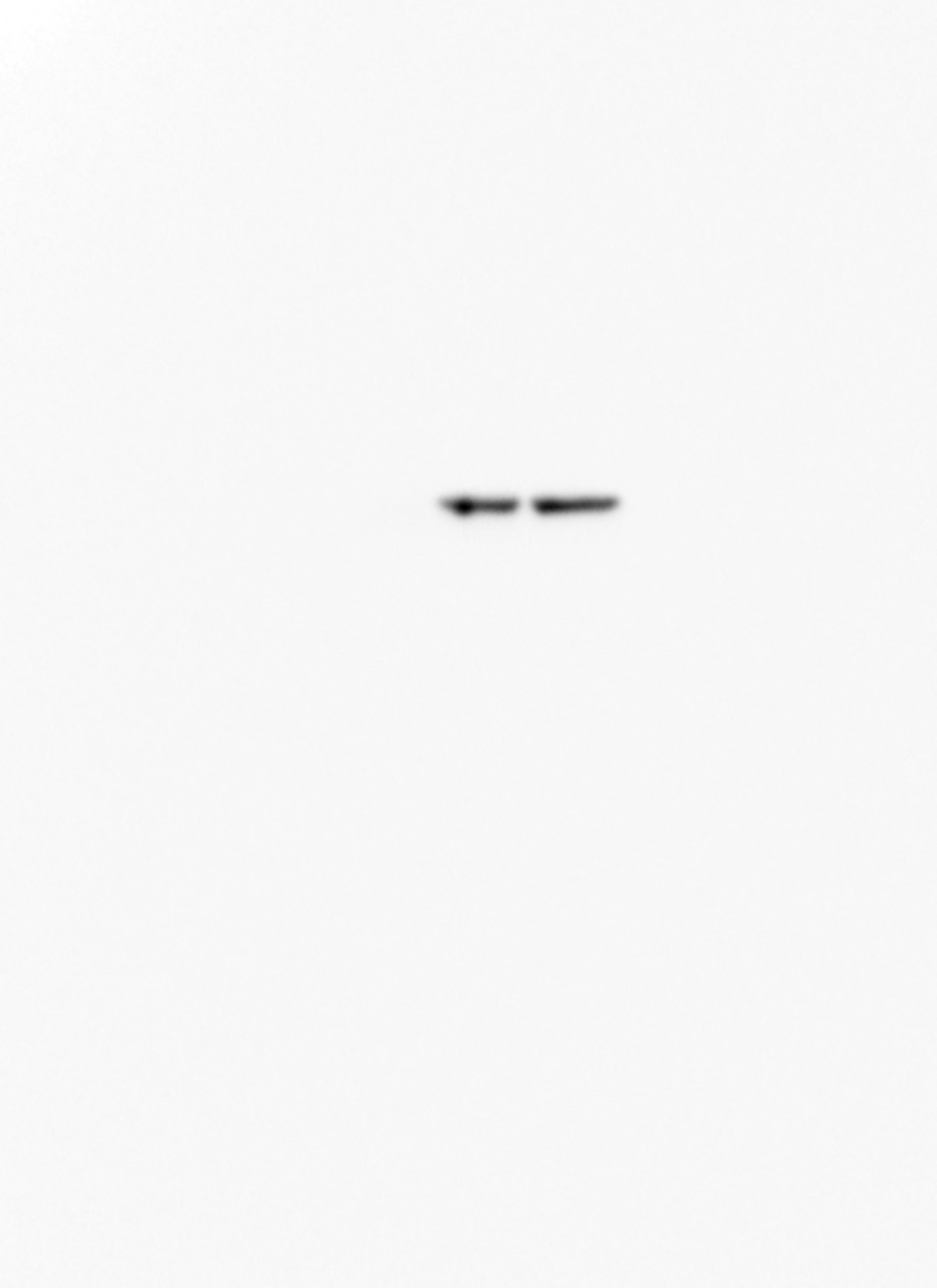

Supplement: Supplemental Information 5 [file peerj-11-15041-s005.zip › Osteoclast-related-genes-raw data1/ACTIN/ACTIN-3/ACTIN-3-1.tif]

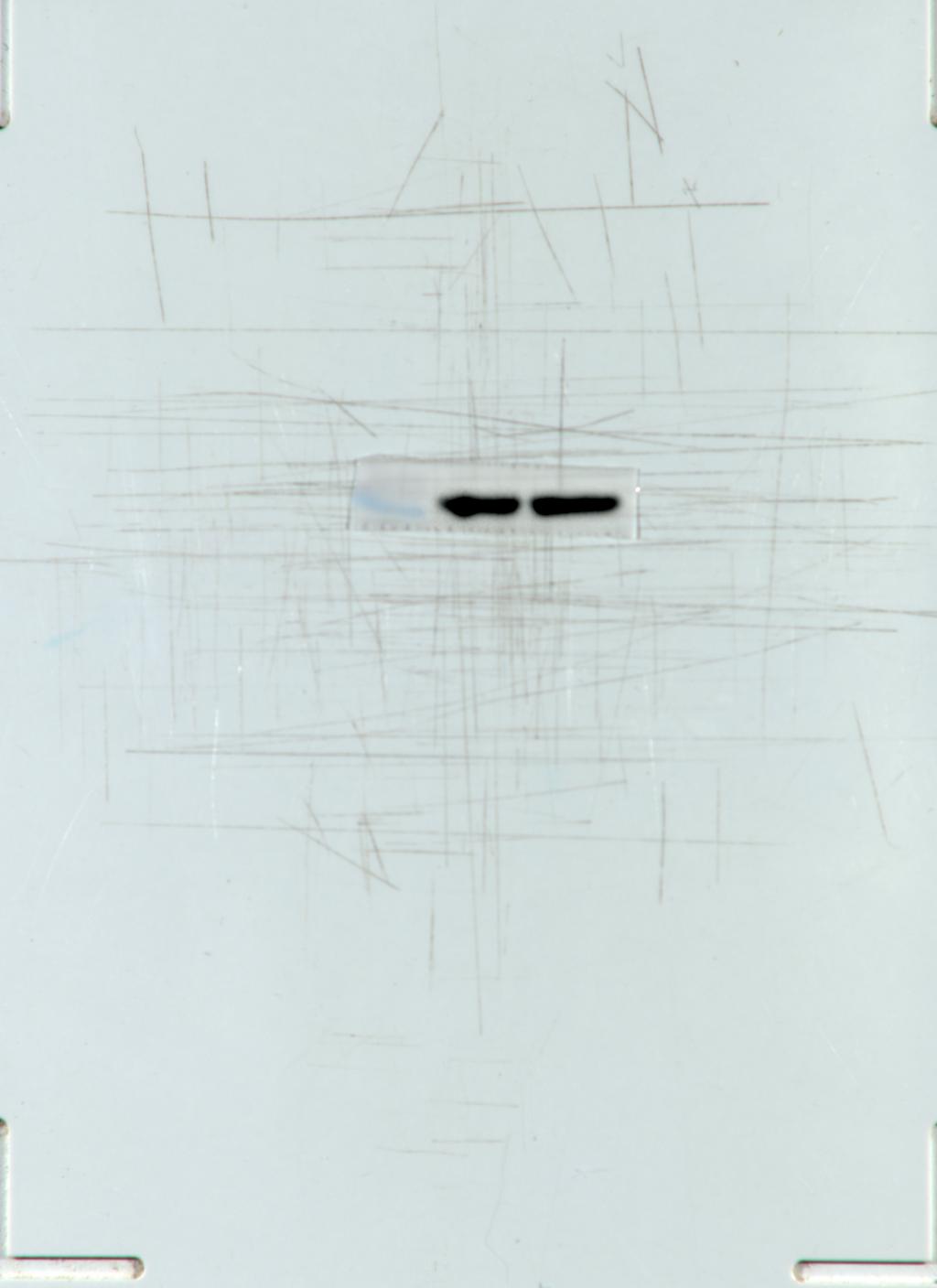

Supplement: Supplemental Information 5 [file peerj-11-15041-s005.zip › Osteoclast-related-genes-raw data1/ACTIN/ACTIN-3/ACTIN-3-2.jpg]

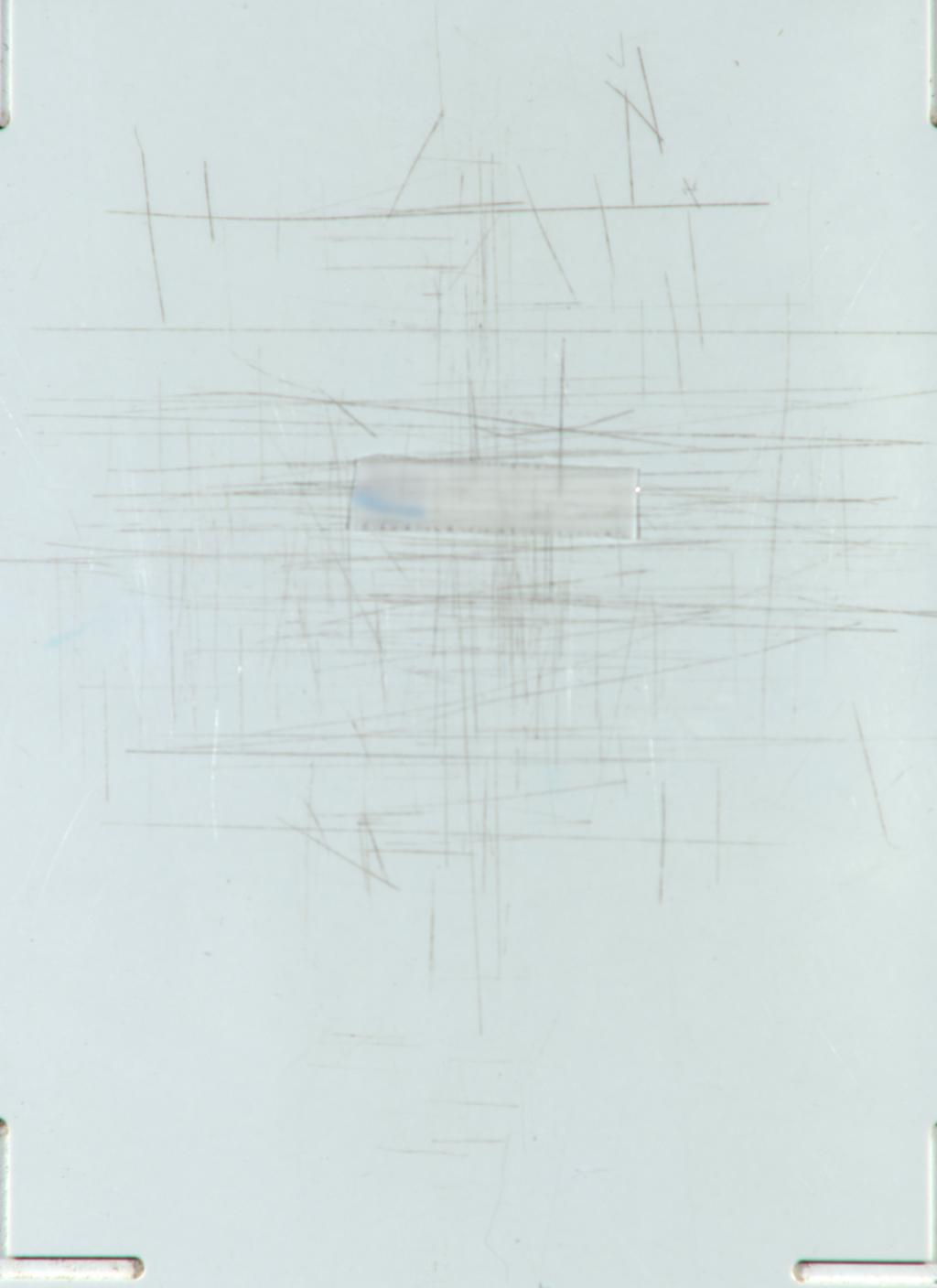

Supplement: Supplemental Information 5 [file peerj-11-15041-s005.zip › Osteoclast-related-genes-raw data1/ACTIN/ACTIN-3/ACTIN-3-3.jpg]

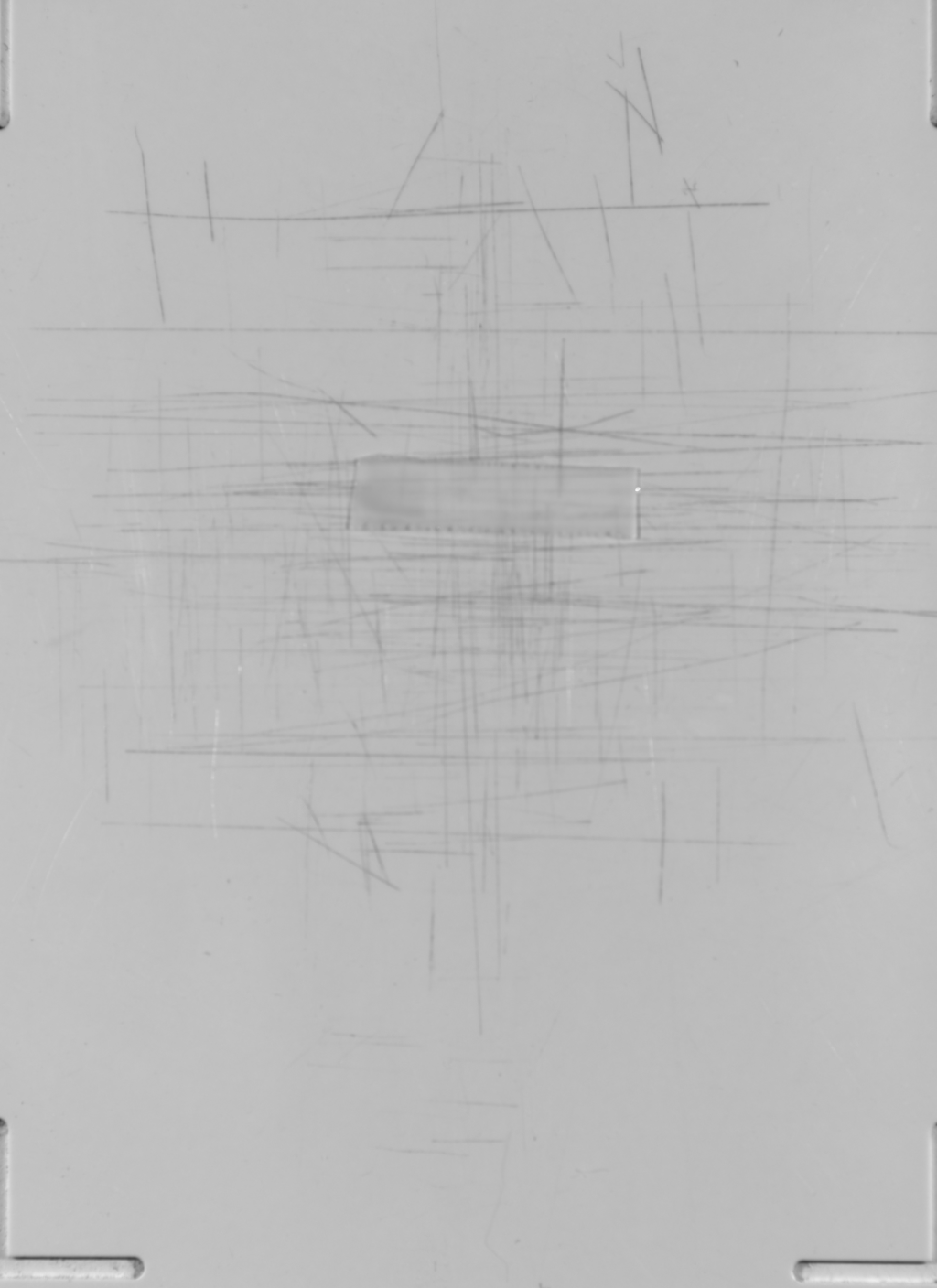

Supplement: Supplemental Information 5 [file peerj-11-15041-s005.zip › Osteoclast-related-genes-raw data1/ACTIN/ACTIN-3/ACTIN-3-4.tif]

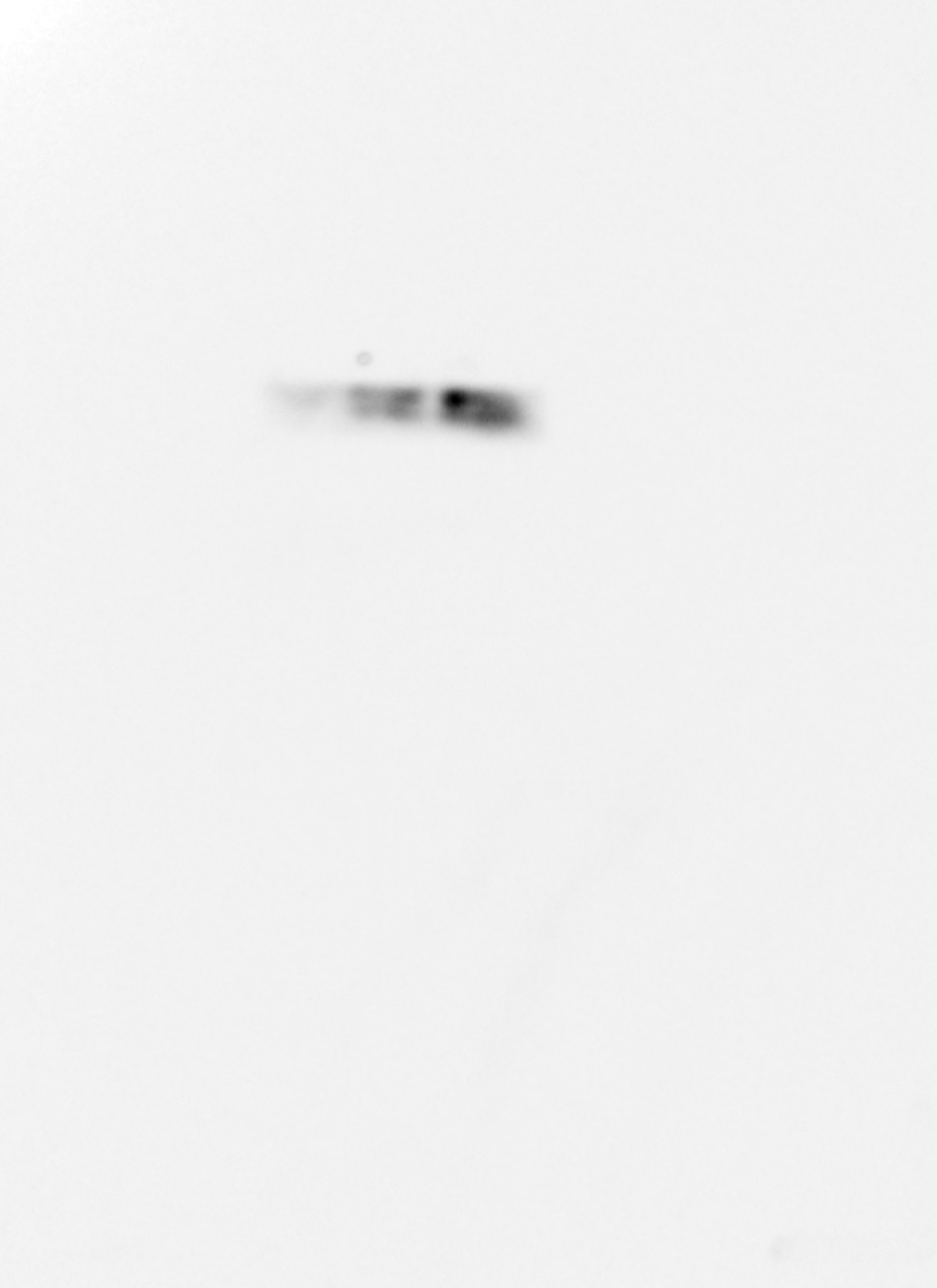

Supplement: Supplemental Information 5 [file peerj-11-15041-s005.zip › Osteoclast-related-genes-raw data1/CTSK/CTSK-1/CTSK-1-1.tif]

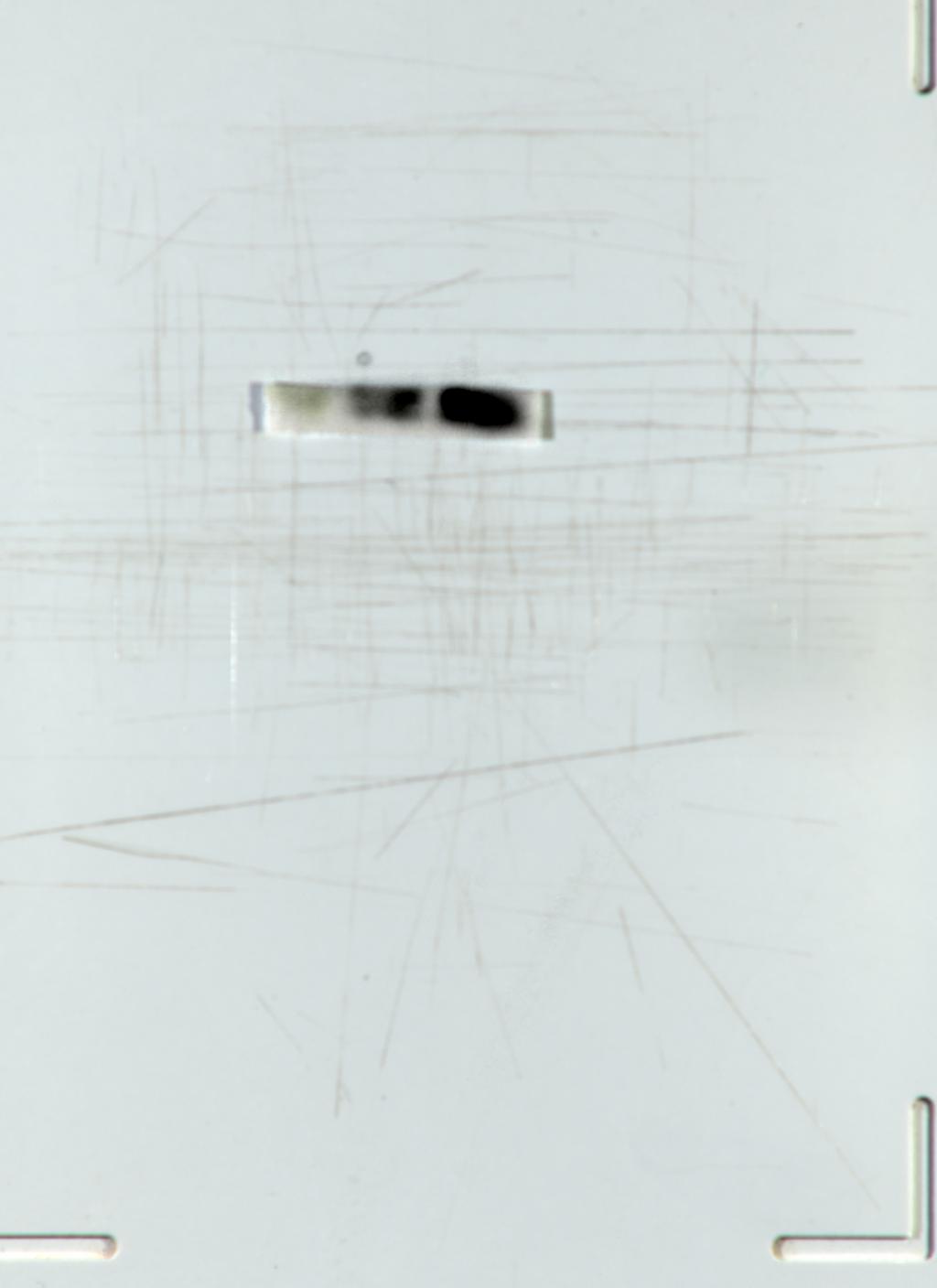

Supplement: Supplemental Information 5 [file peerj-11-15041-s005.zip › Osteoclast-related-genes-raw data1/CTSK/CTSK-1/CTSK-1-2.jpg]

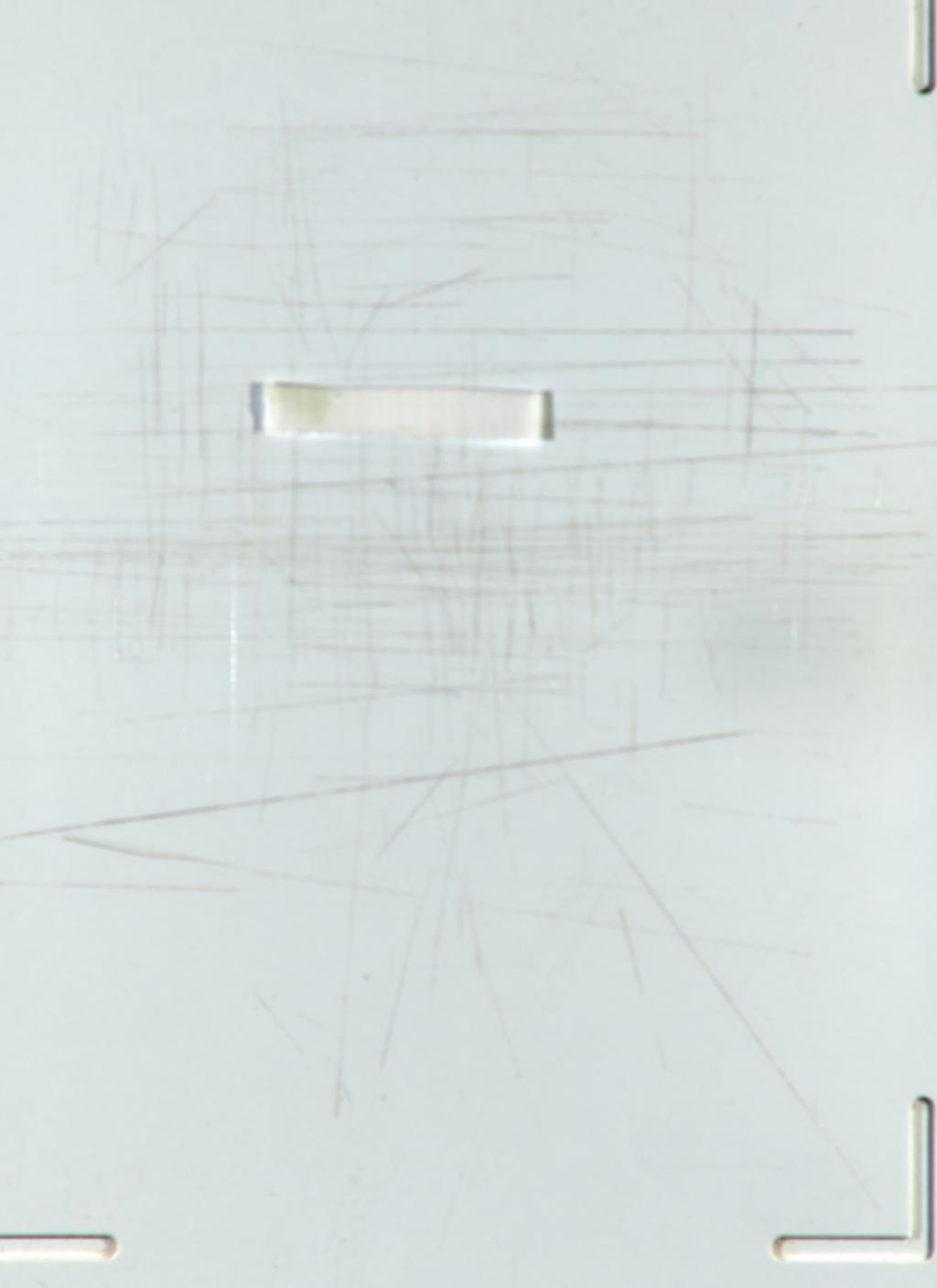

Supplement: Supplemental Information 5 [file peerj-11-15041-s005.zip › Osteoclast-related-genes-raw data1/CTSK/CTSK-1/CTSK-1-3.jpg]

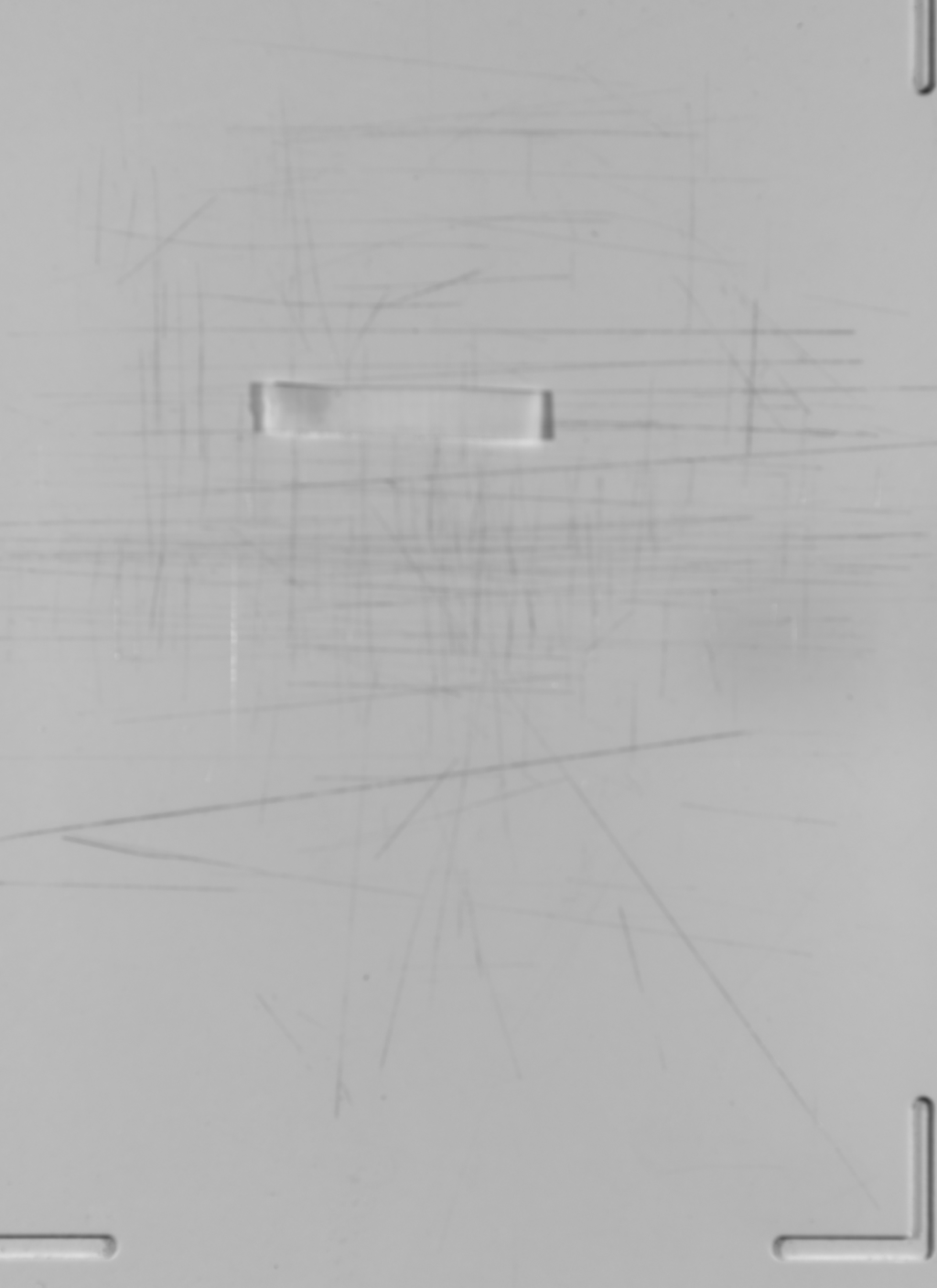

Supplement: Supplemental Information 5 [file peerj-11-15041-s005.zip › Osteoclast-related-genes-raw data1/CTSK/CTSK-1/CTSK-1-4.tif]

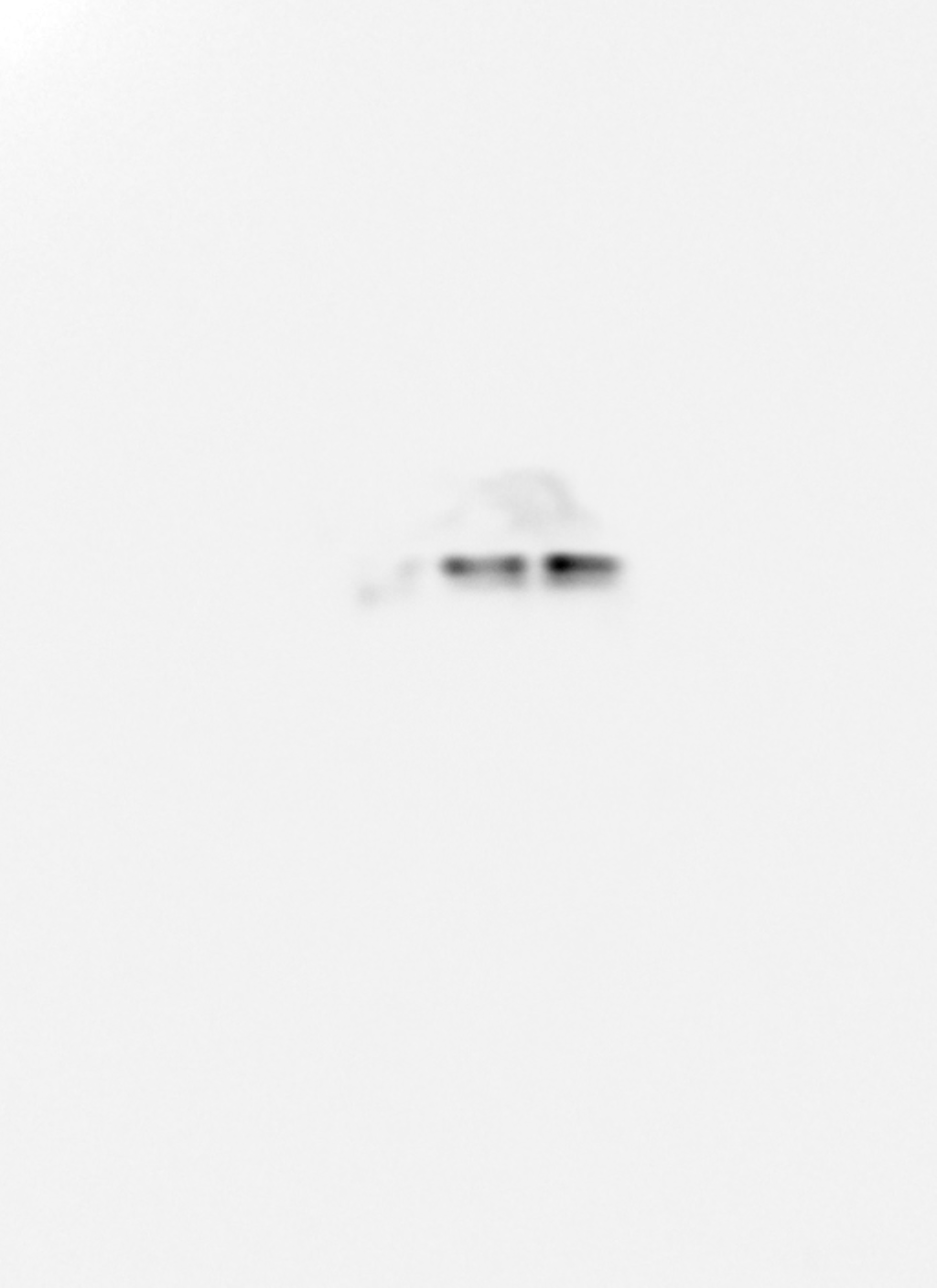

Supplement: Supplemental Information 5 [file peerj-11-15041-s005.zip › Osteoclast-related-genes-raw data1/CTSK/CTSK-2/CTSK-2-1.tif]

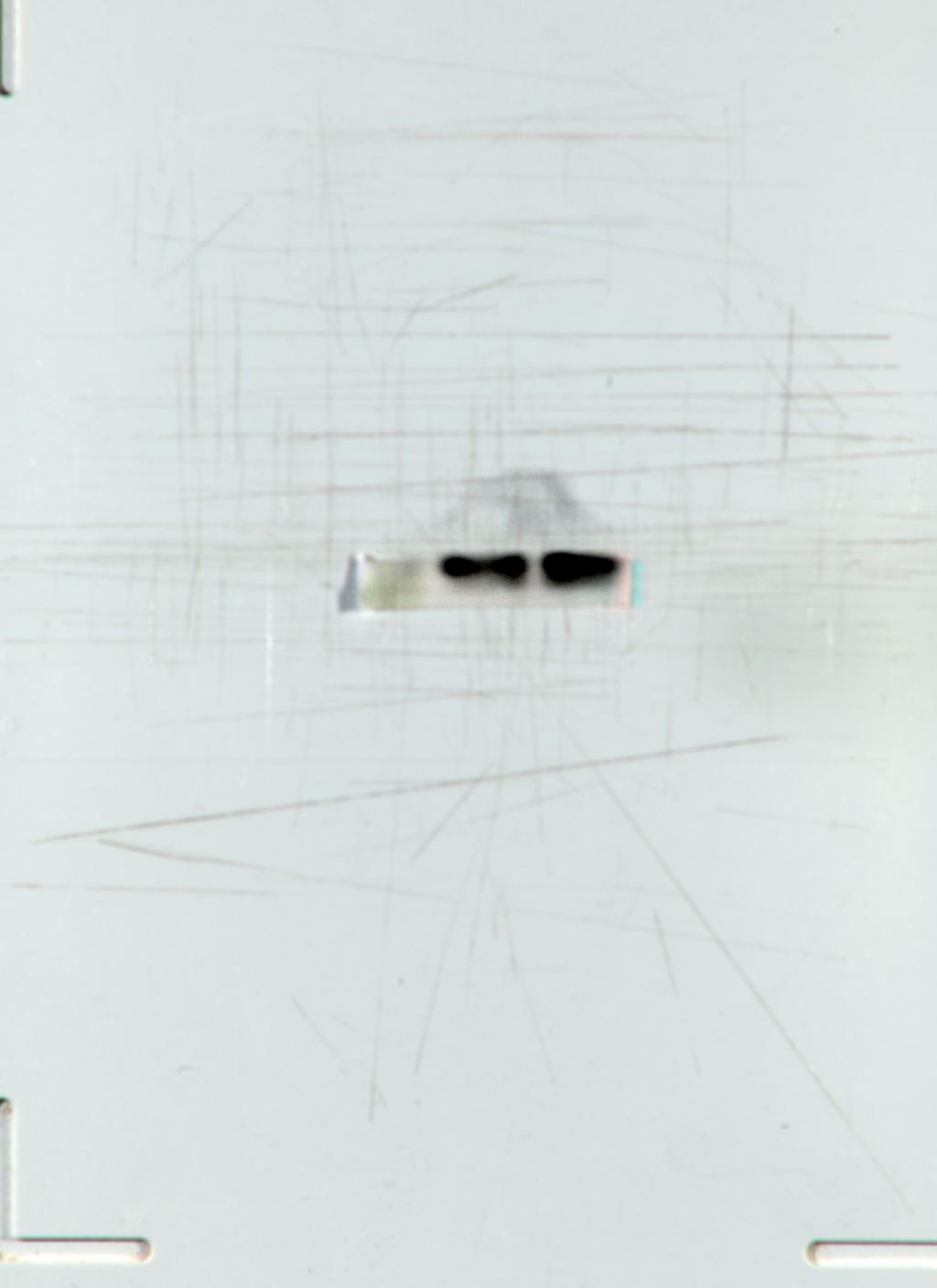

Supplement: Supplemental Information 5 [file peerj-11-15041-s005.zip › Osteoclast-related-genes-raw data1/CTSK/CTSK-2/CTSK-2-2.jpg]

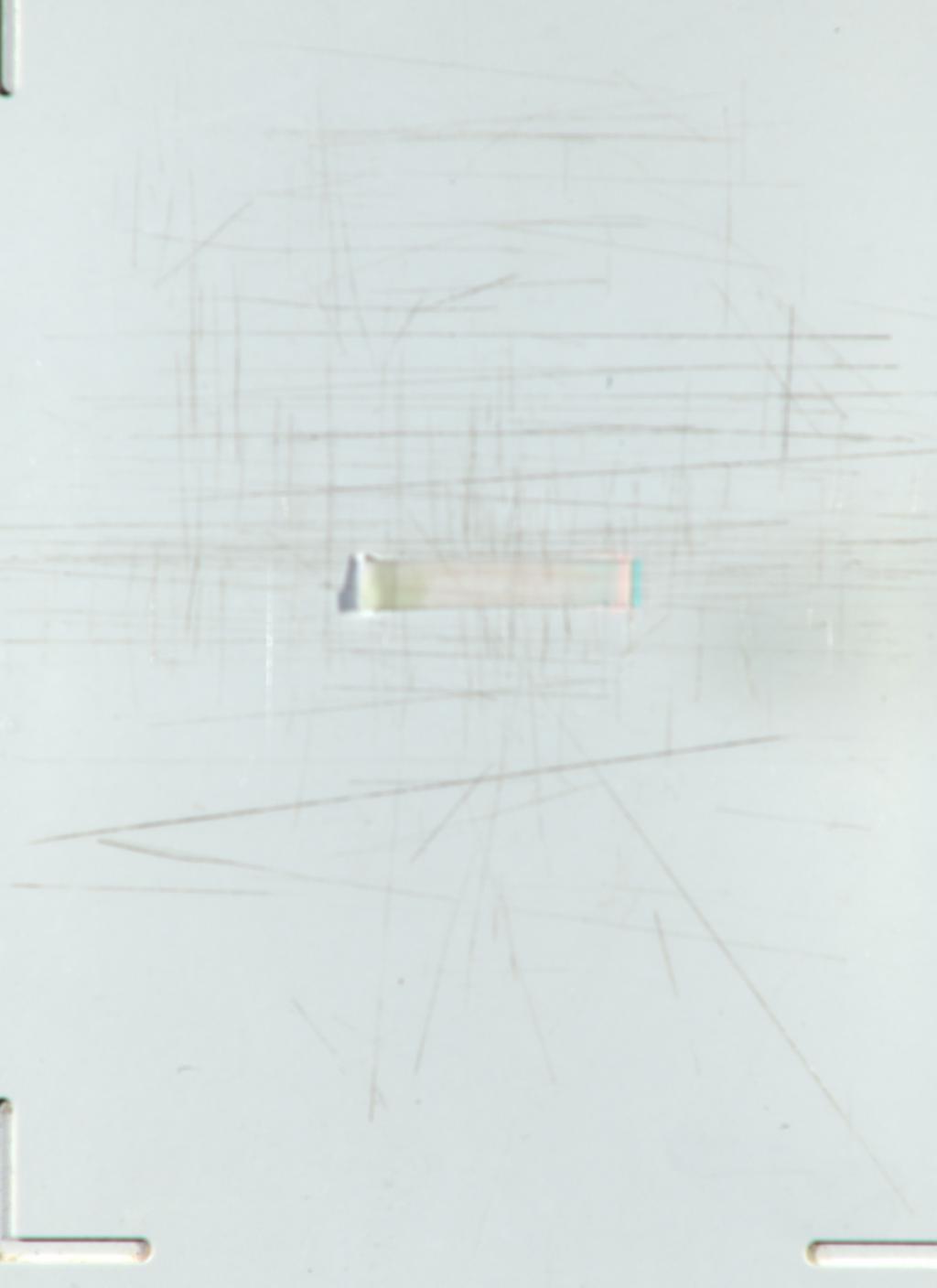

Supplement: Supplemental Information 5 [file peerj-11-15041-s005.zip › Osteoclast-related-genes-raw data1/CTSK/CTSK-2/CTSK-2-3.jpg]

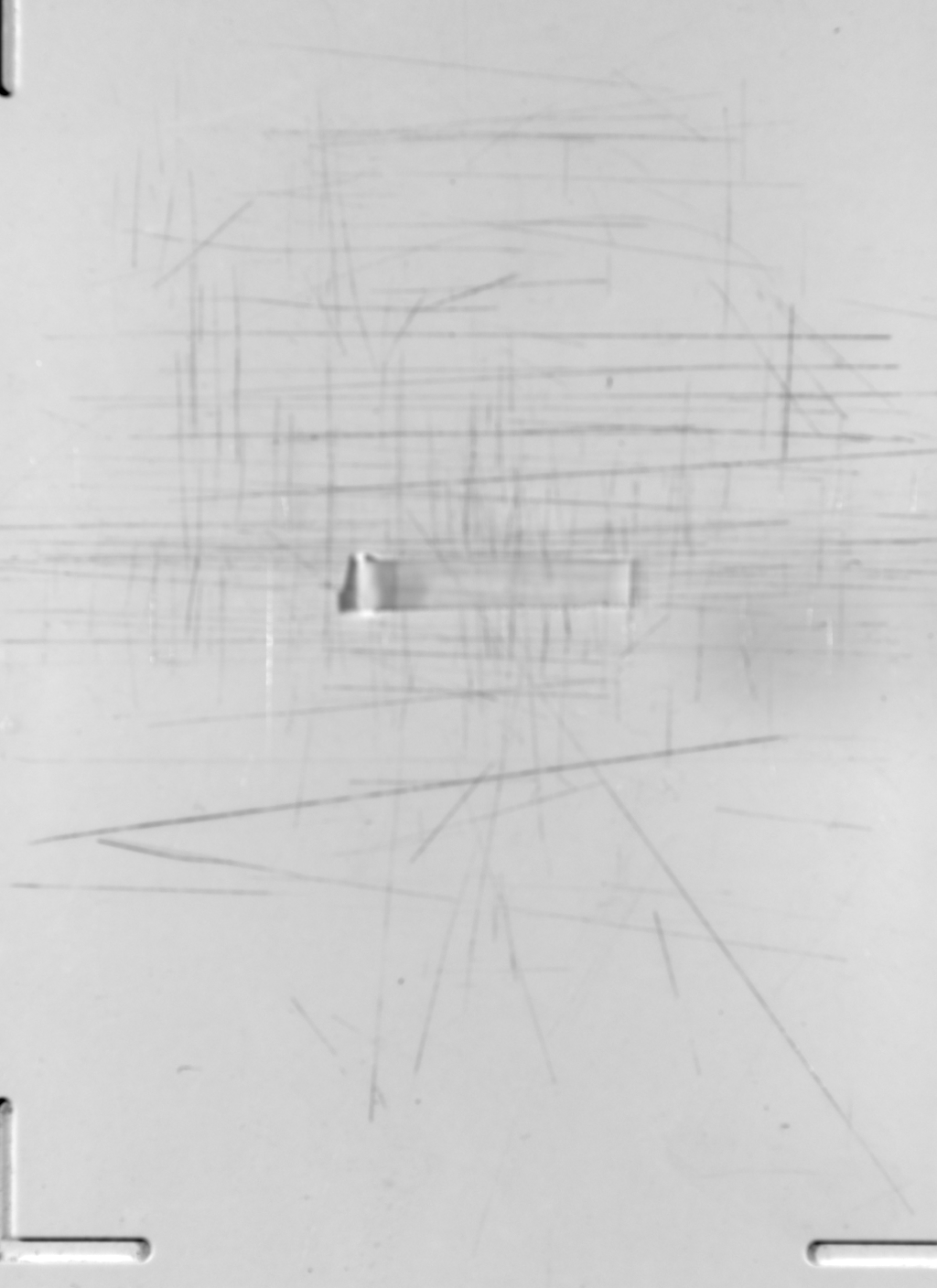

Supplement: Supplemental Information 5 [file peerj-11-15041-s005.zip › Osteoclast-related-genes-raw data1/CTSK/CTSK-2/CTSK-2-4.tif]

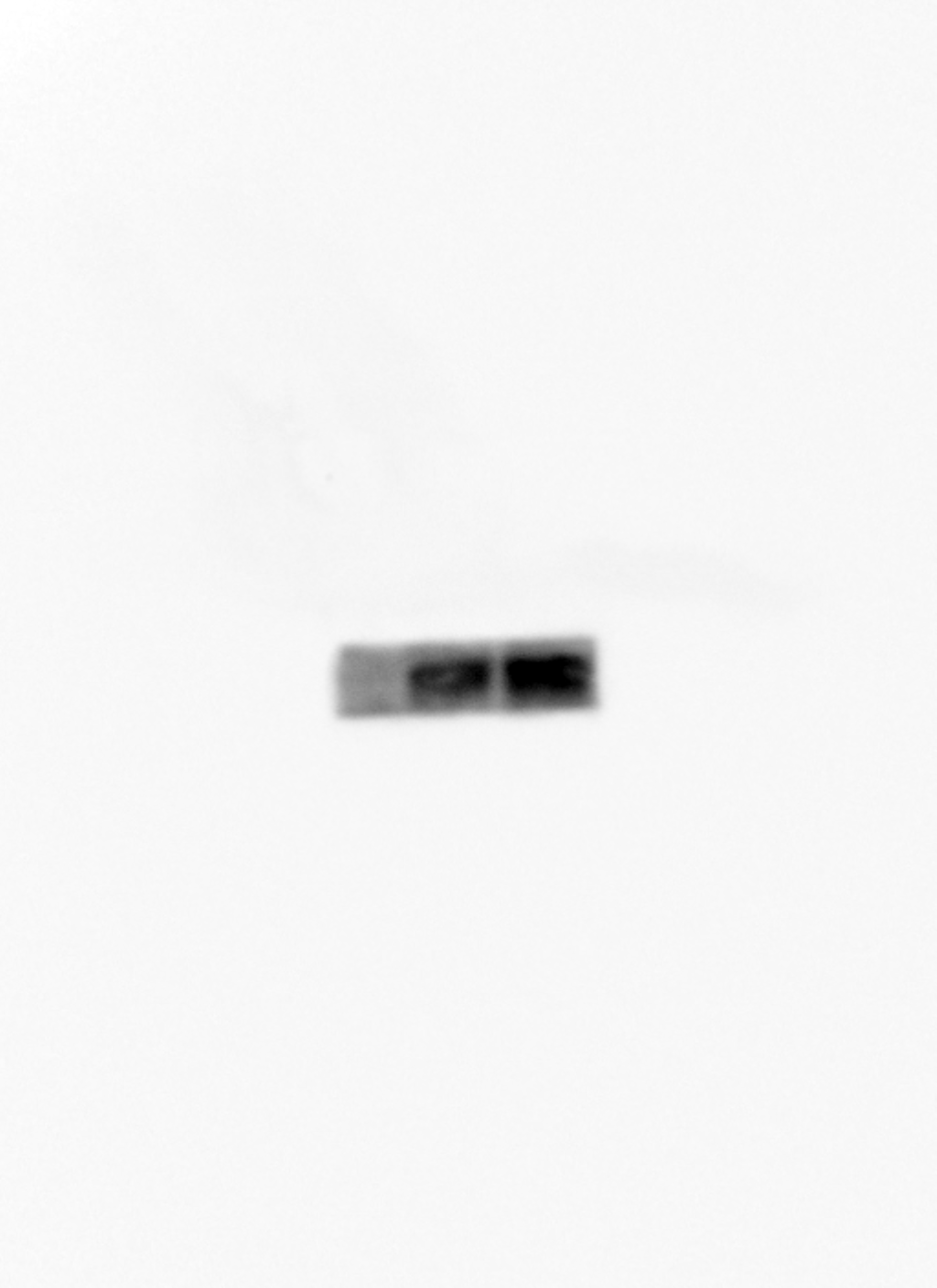

Supplement: Supplemental Information 5 [file peerj-11-15041-s005.zip › Osteoclast-related-genes-raw data1/CTSK/CTSK-3/CTSK-3-1.tif]

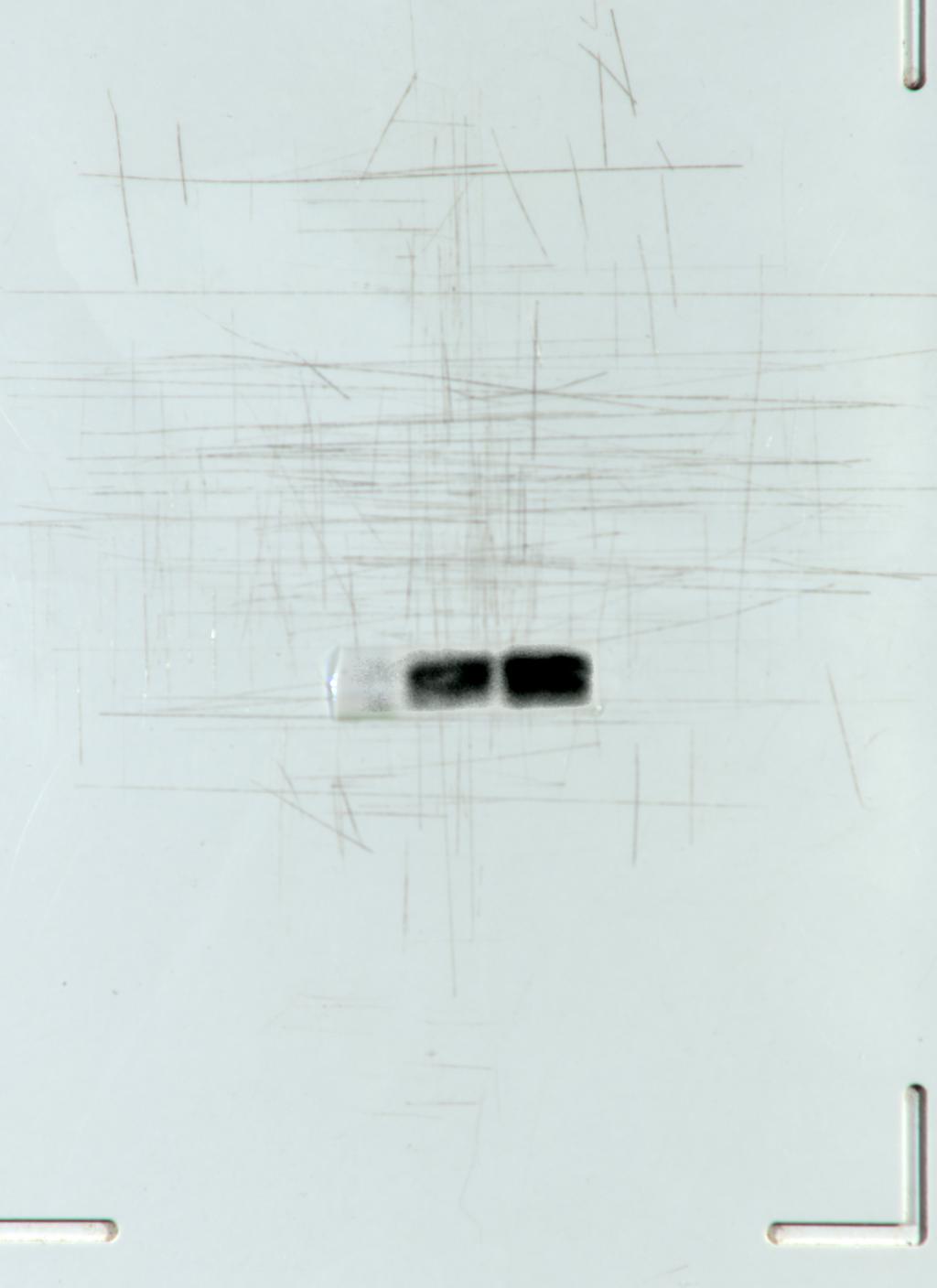

Supplement: Supplemental Information 5 [file peerj-11-15041-s005.zip › Osteoclast-related-genes-raw data1/CTSK/CTSK-3/CTSK-3-2.jpg]

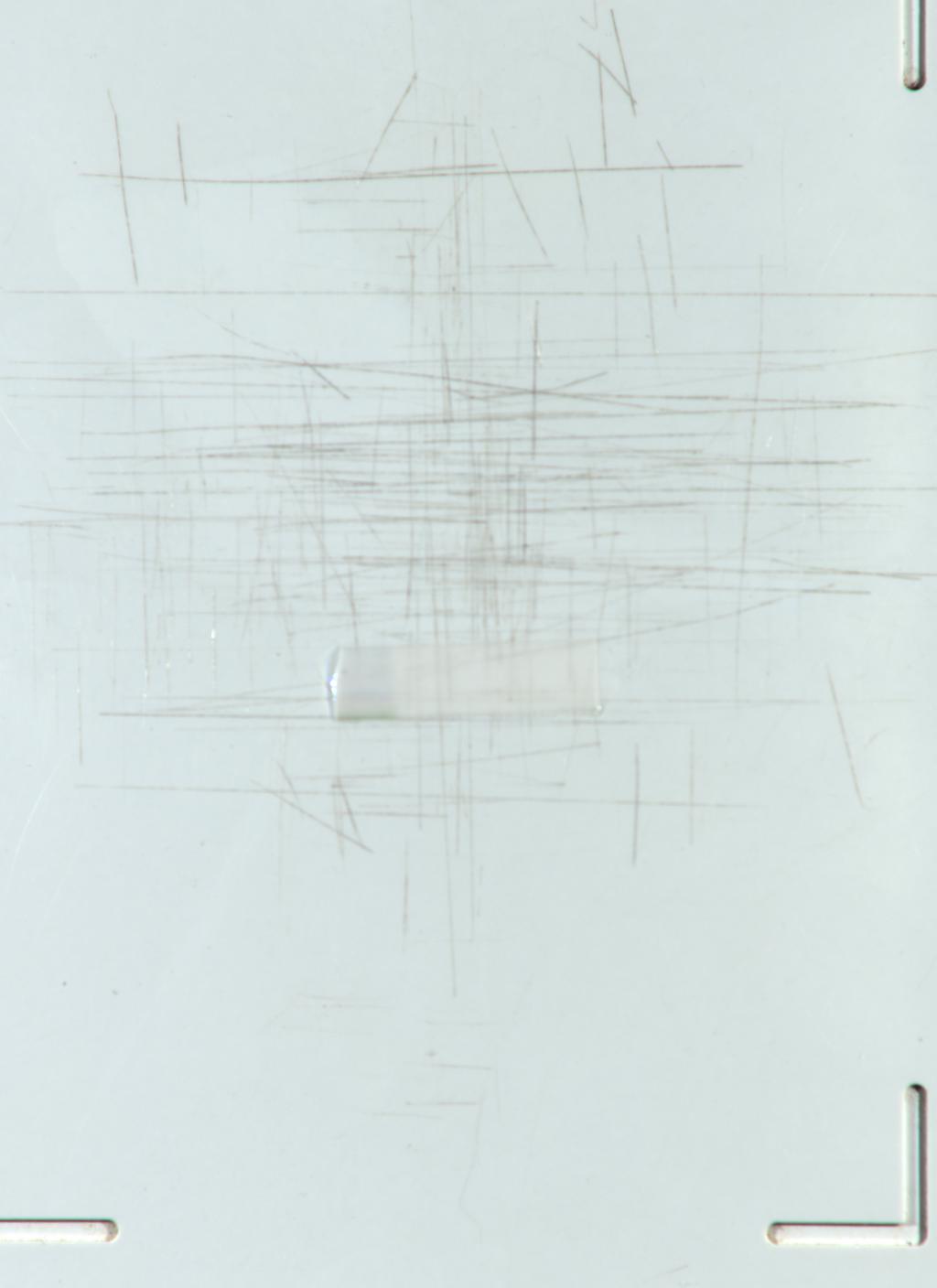

Supplement: Supplemental Information 5 [file peerj-11-15041-s005.zip › Osteoclast-related-genes-raw data1/CTSK/CTSK-3/CTSK-3-3.jpg]

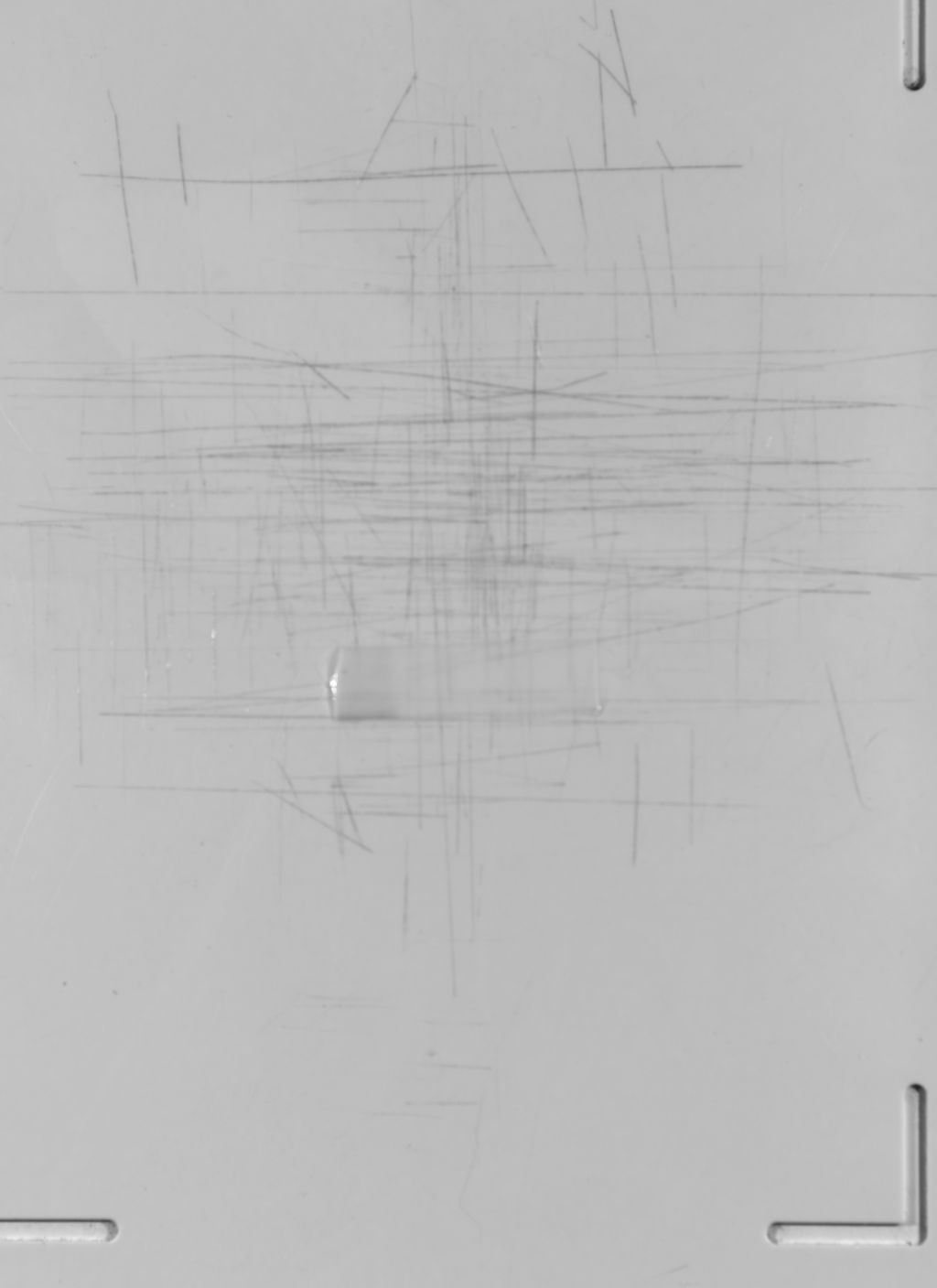

Supplement: Supplemental Information 5 [file peerj-11-15041-s005.zip › Osteoclast-related-genes-raw data1/CTSK/CTSK-3/CTSK-3-4.tif]

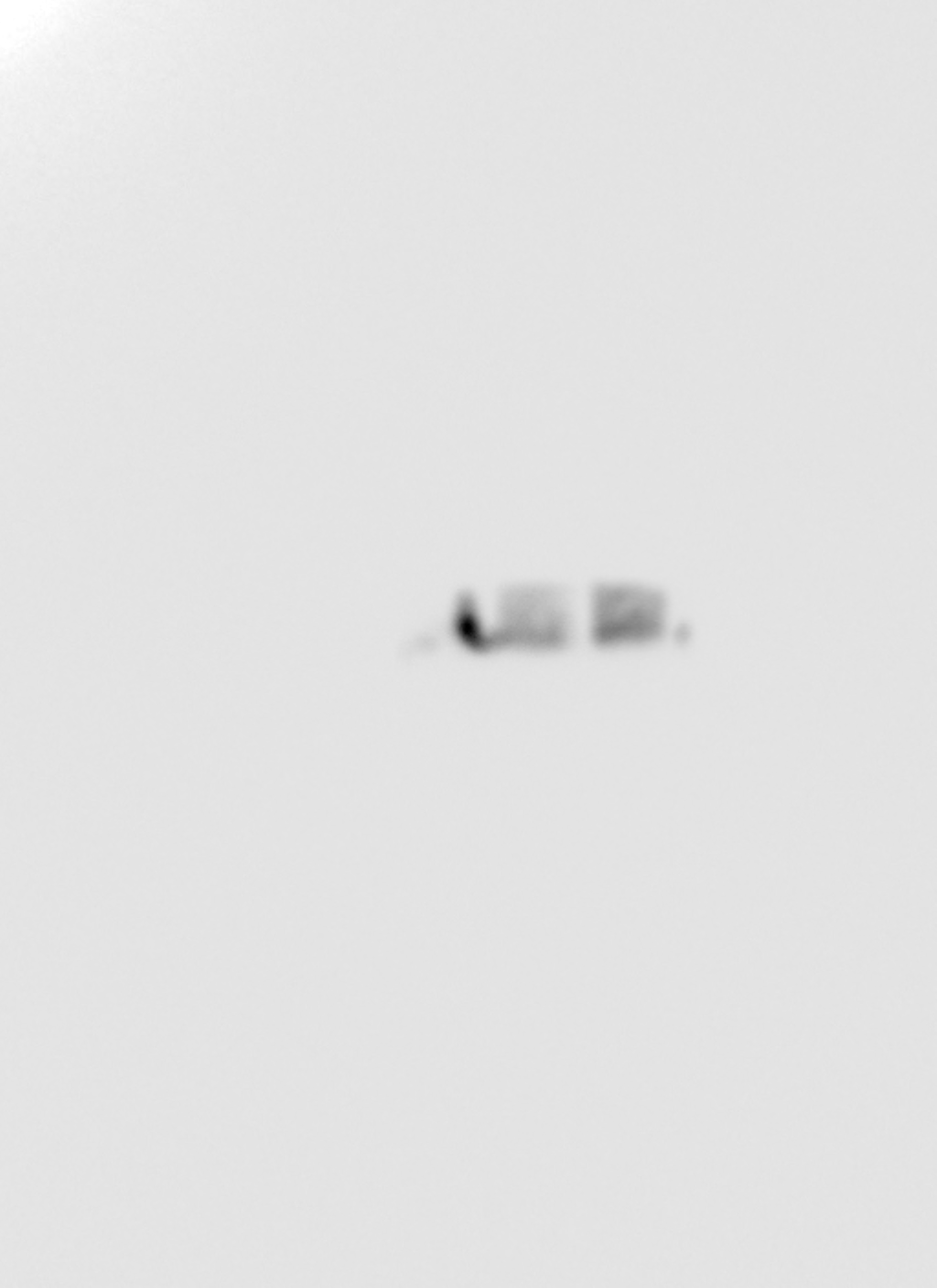

Supplement: Supplemental Information 6 [file peerj-11-15041-s006.zip › Osteoclast-related-genes-raw data2/MMP9/MMP9-1/MMP9-1-1.tif]

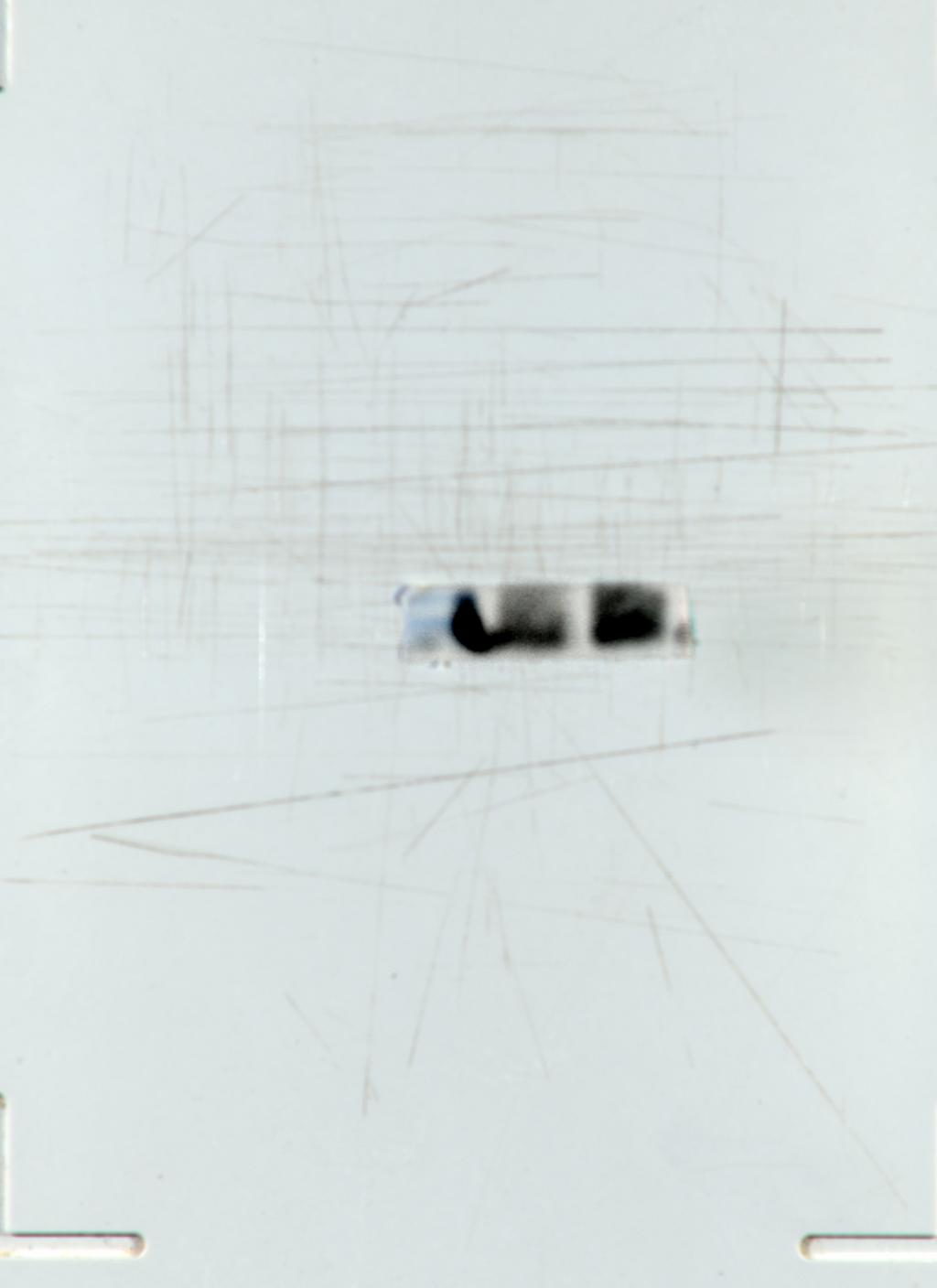

Supplement: Supplemental Information 6 [file peerj-11-15041-s006.zip › Osteoclast-related-genes-raw data2/MMP9/MMP9-1/MMP9-1-2.jpg]

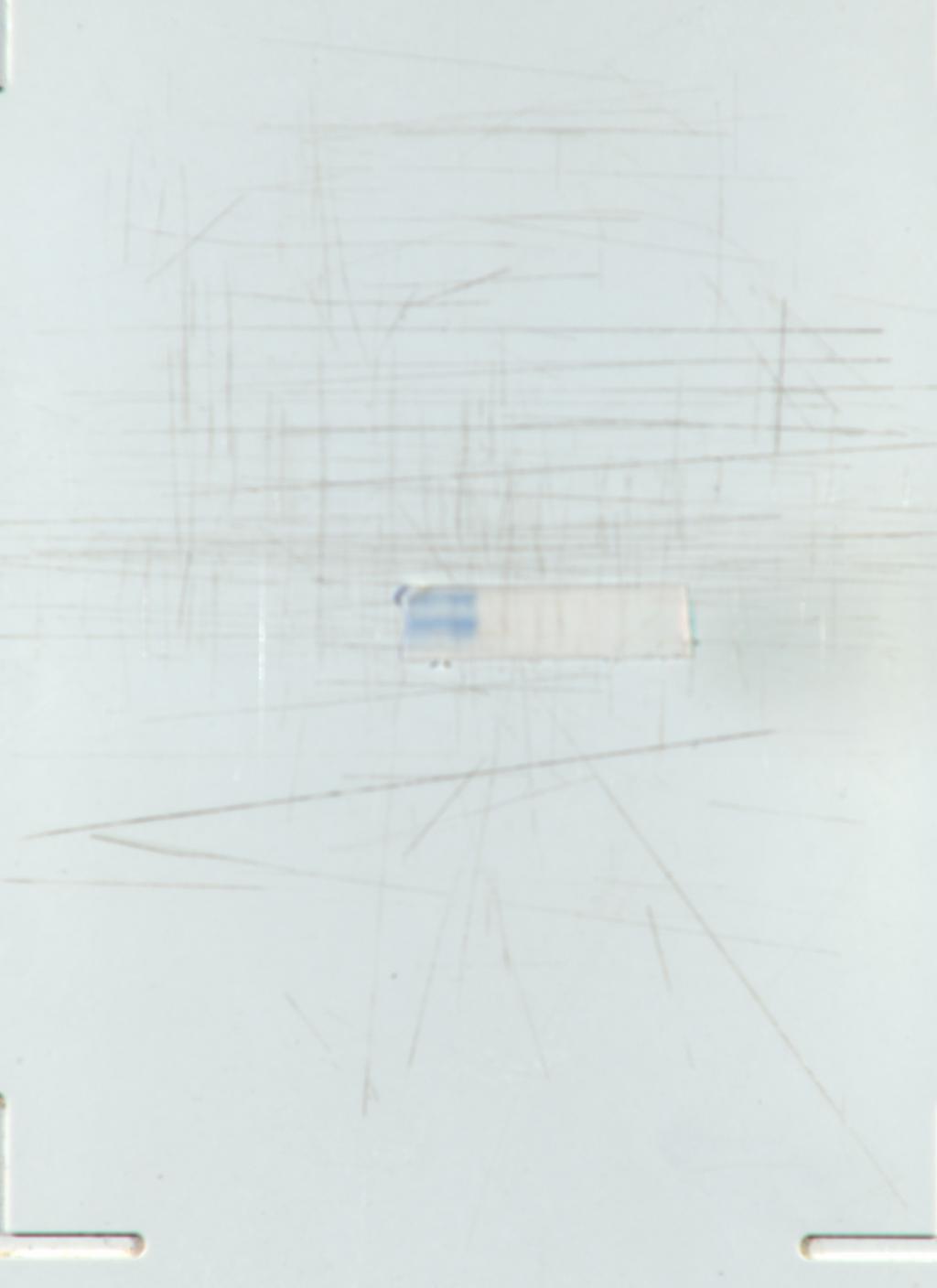

Supplement: Supplemental Information 6 [file peerj-11-15041-s006.zip › Osteoclast-related-genes-raw data2/MMP9/MMP9-1/MMP9-1-3.jpg]

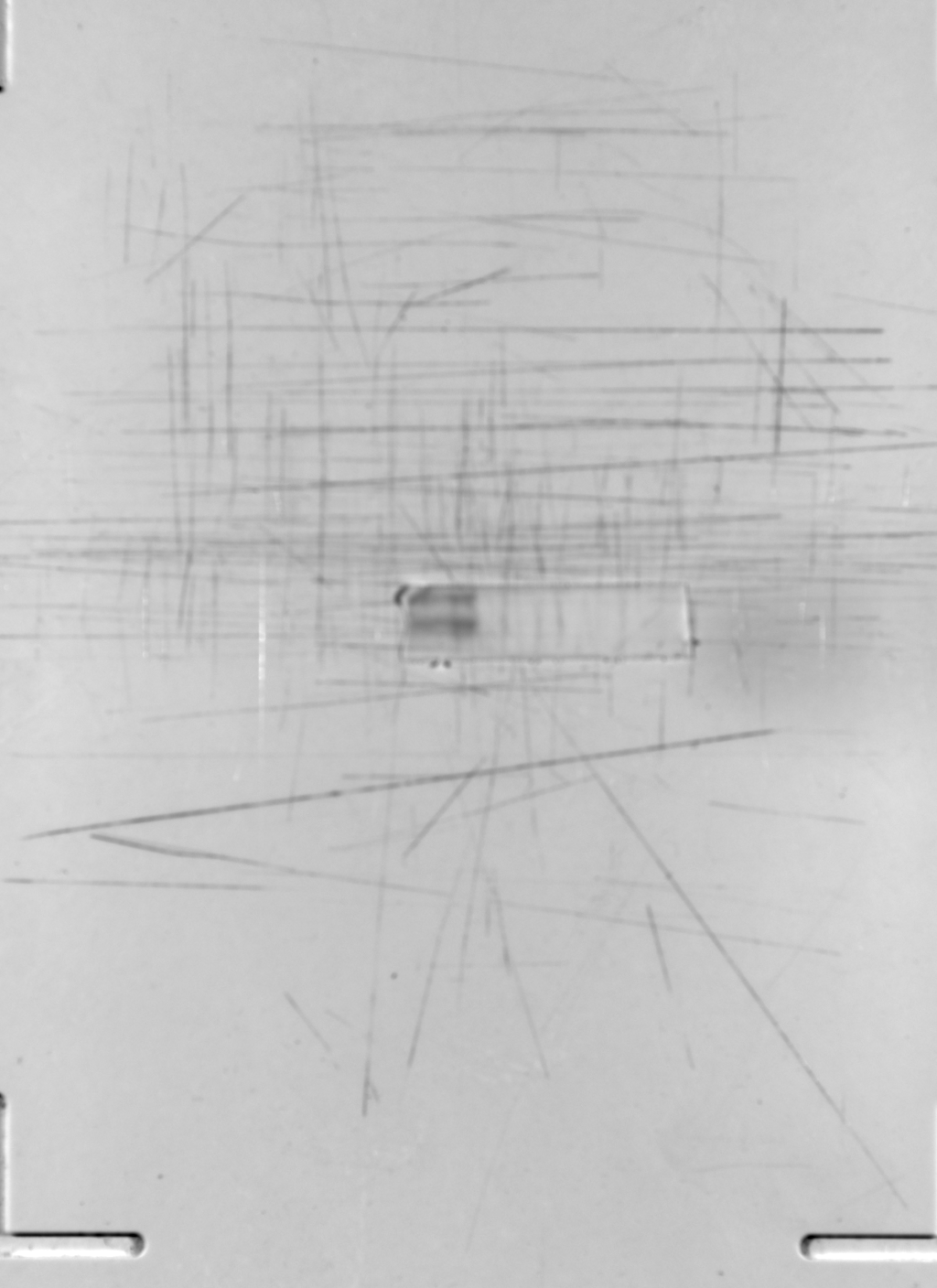

Supplement: Supplemental Information 6 [file peerj-11-15041-s006.zip › Osteoclast-related-genes-raw data2/MMP9/MMP9-1/MMP9-1-4.tif]

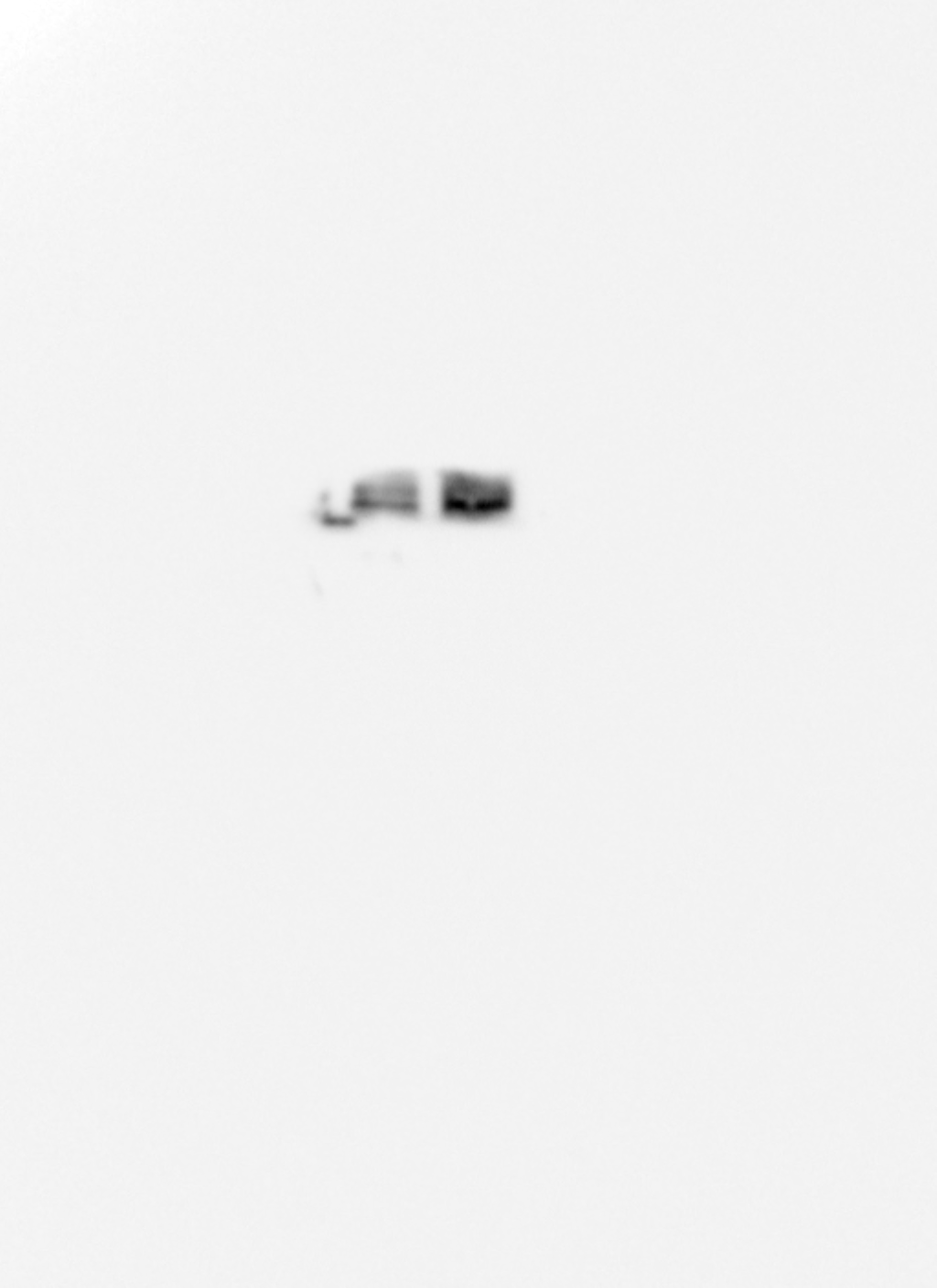

Supplement: Supplemental Information 6 [file peerj-11-15041-s006.zip › Osteoclast-related-genes-raw data2/MMP9/MMP9-2/MMP9-2-1.tif]

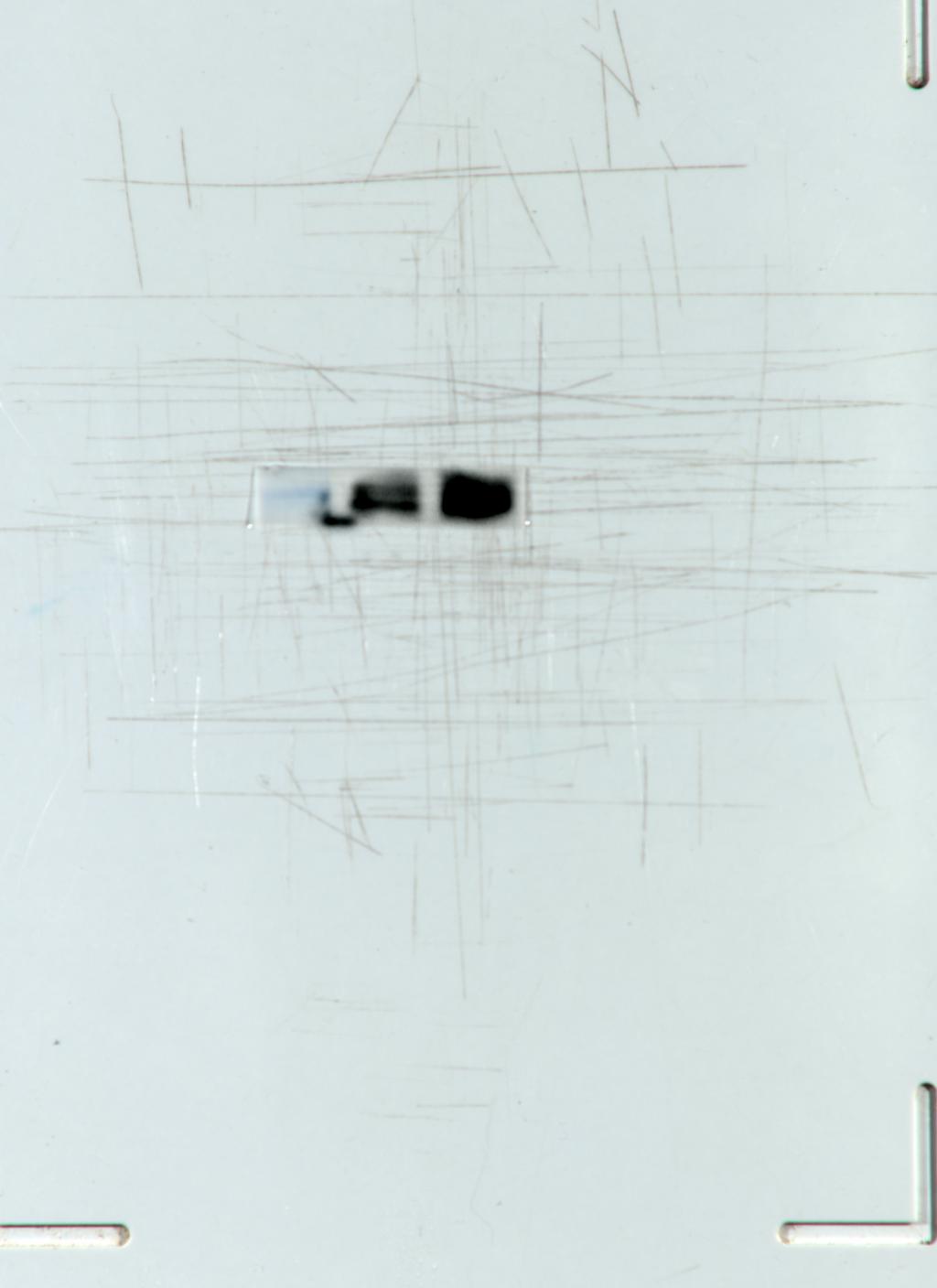

Supplement: Supplemental Information 6 [file peerj-11-15041-s006.zip › Osteoclast-related-genes-raw data2/MMP9/MMP9-2/MMP9-2-2.jpg]

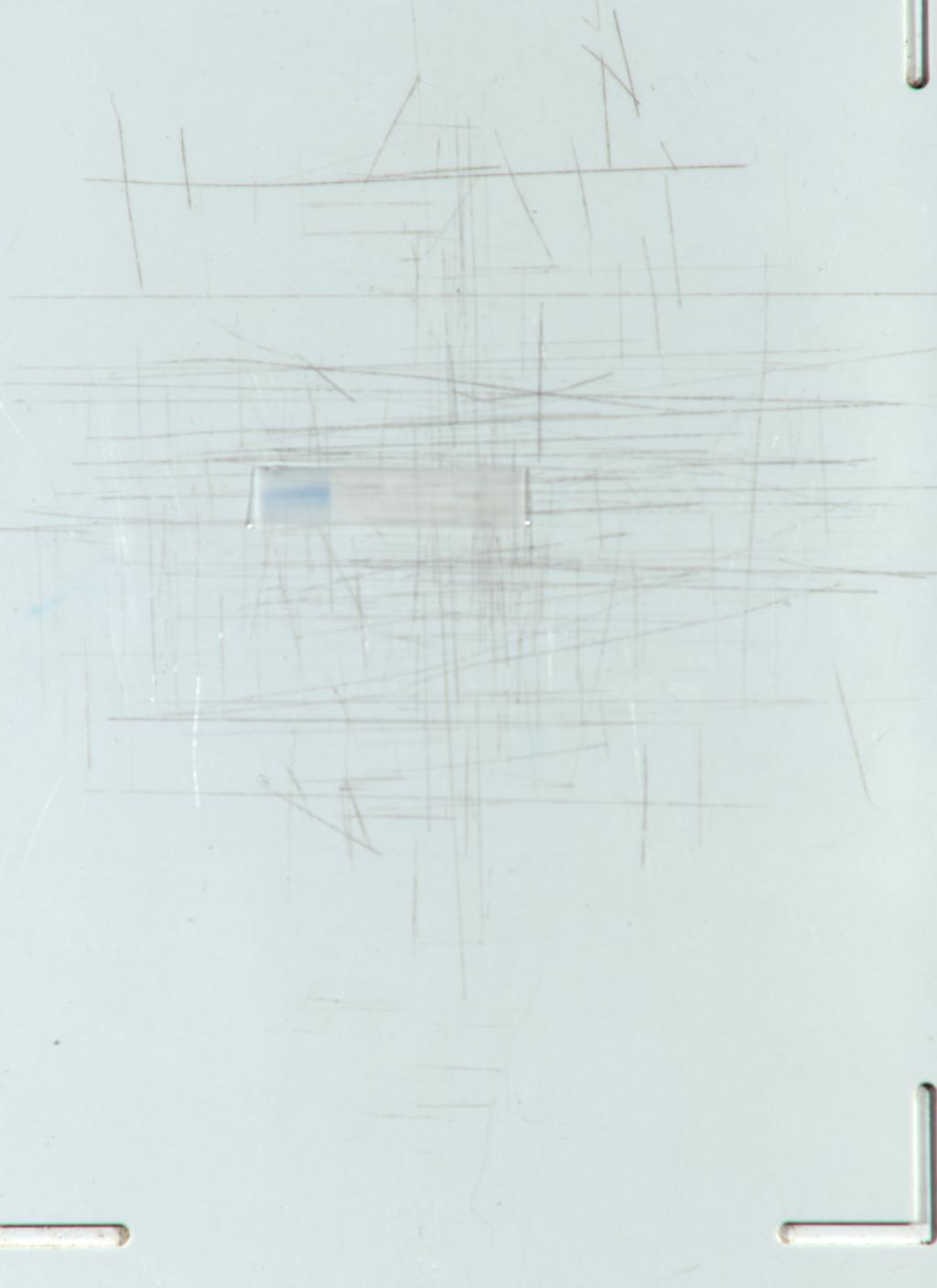

Supplement: Supplemental Information 6 [file peerj-11-15041-s006.zip › Osteoclast-related-genes-raw data2/MMP9/MMP9-2/MMP9-2-3.jpg]

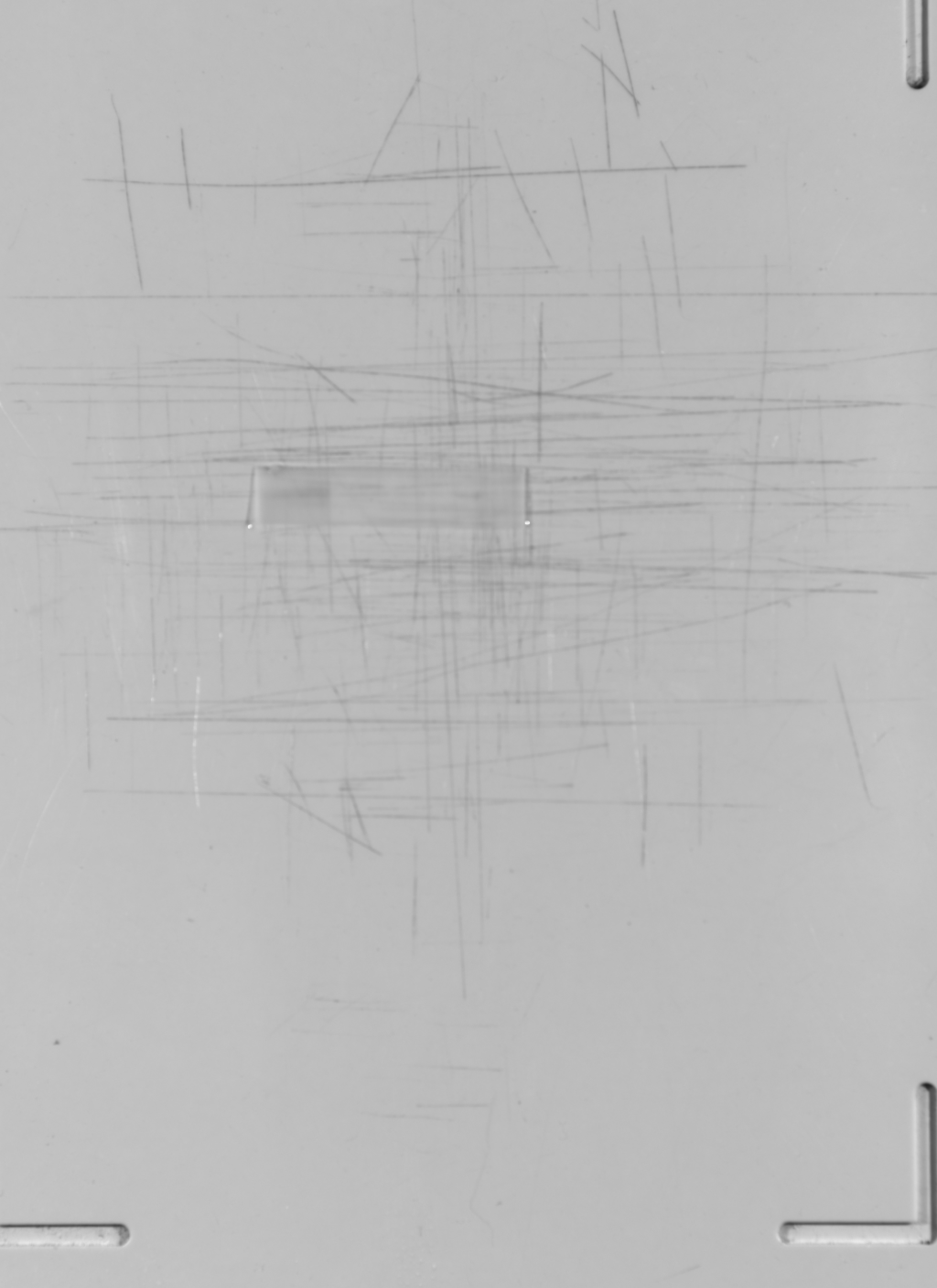

Supplement: Supplemental Information 6 [file peerj-11-15041-s006.zip › Osteoclast-related-genes-raw data2/MMP9/MMP9-2/MMP9-2-4.tif]

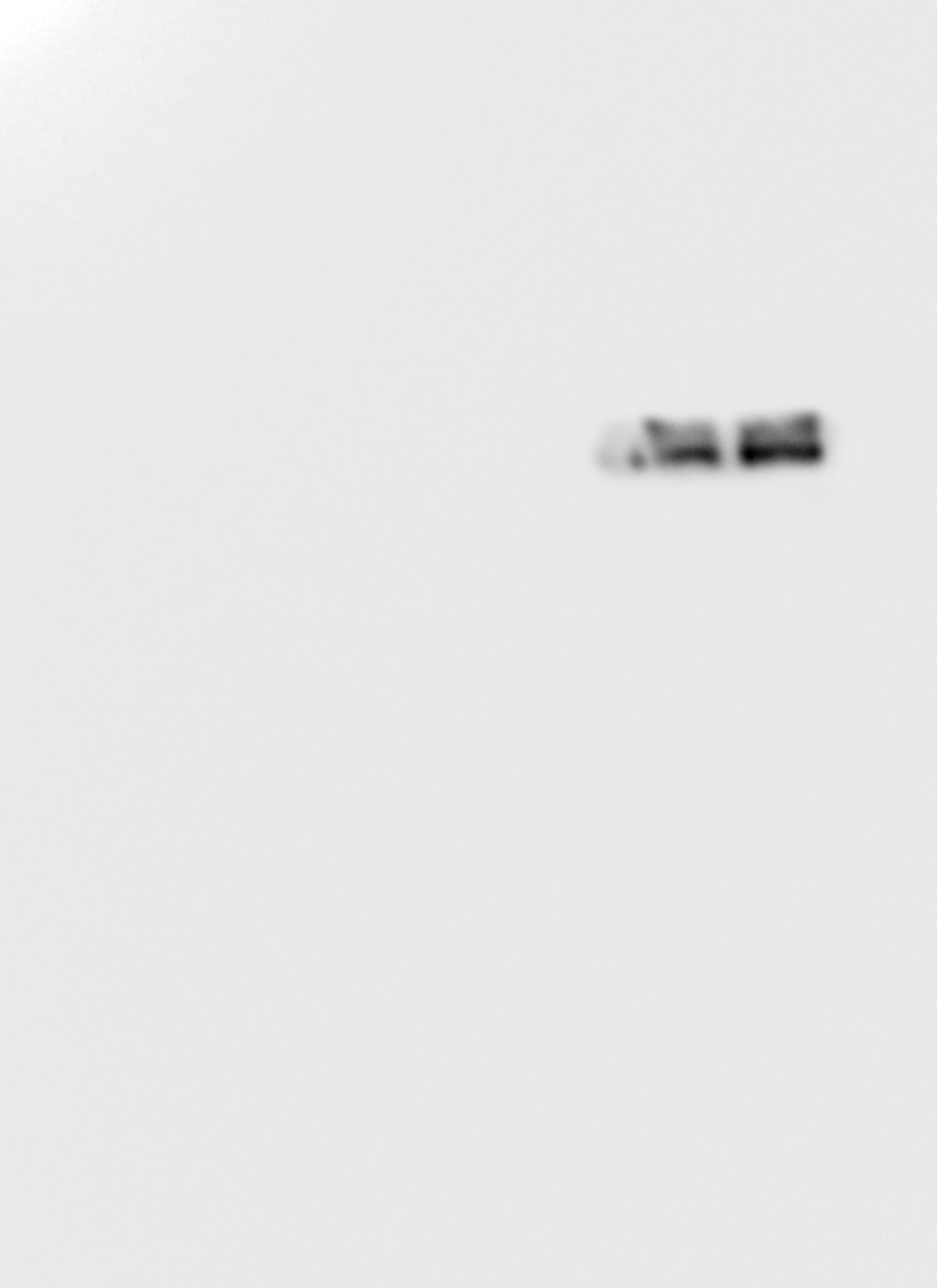

Supplement: Supplemental Information 6 [file peerj-11-15041-s006.zip › Osteoclast-related-genes-raw data2/MMP9/MMP9-3/MMP9-3-1.tif]

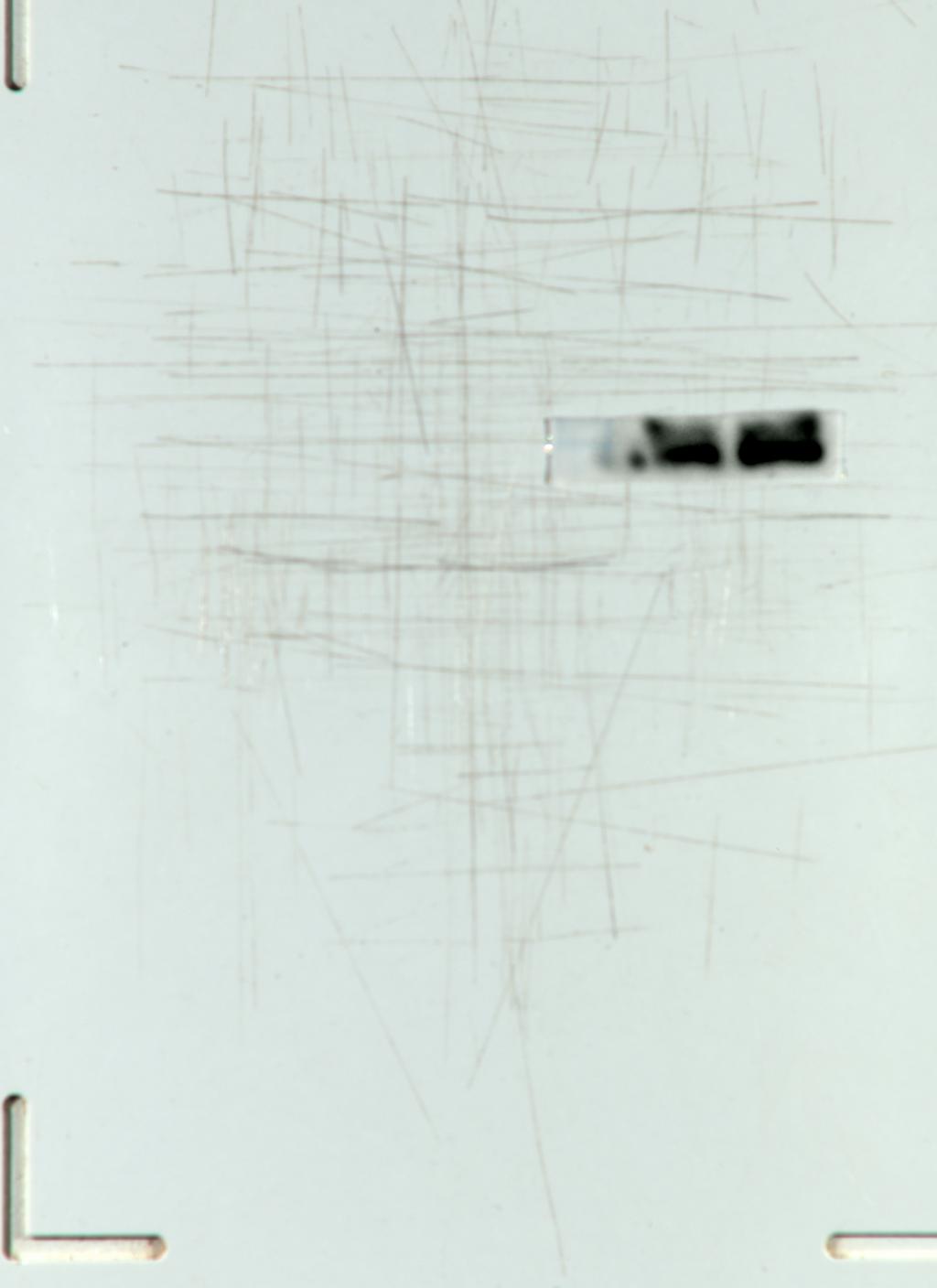

Supplement: Supplemental Information 6 [file peerj-11-15041-s006.zip › Osteoclast-related-genes-raw data2/MMP9/MMP9-3/MMP9-3-2.jpg]

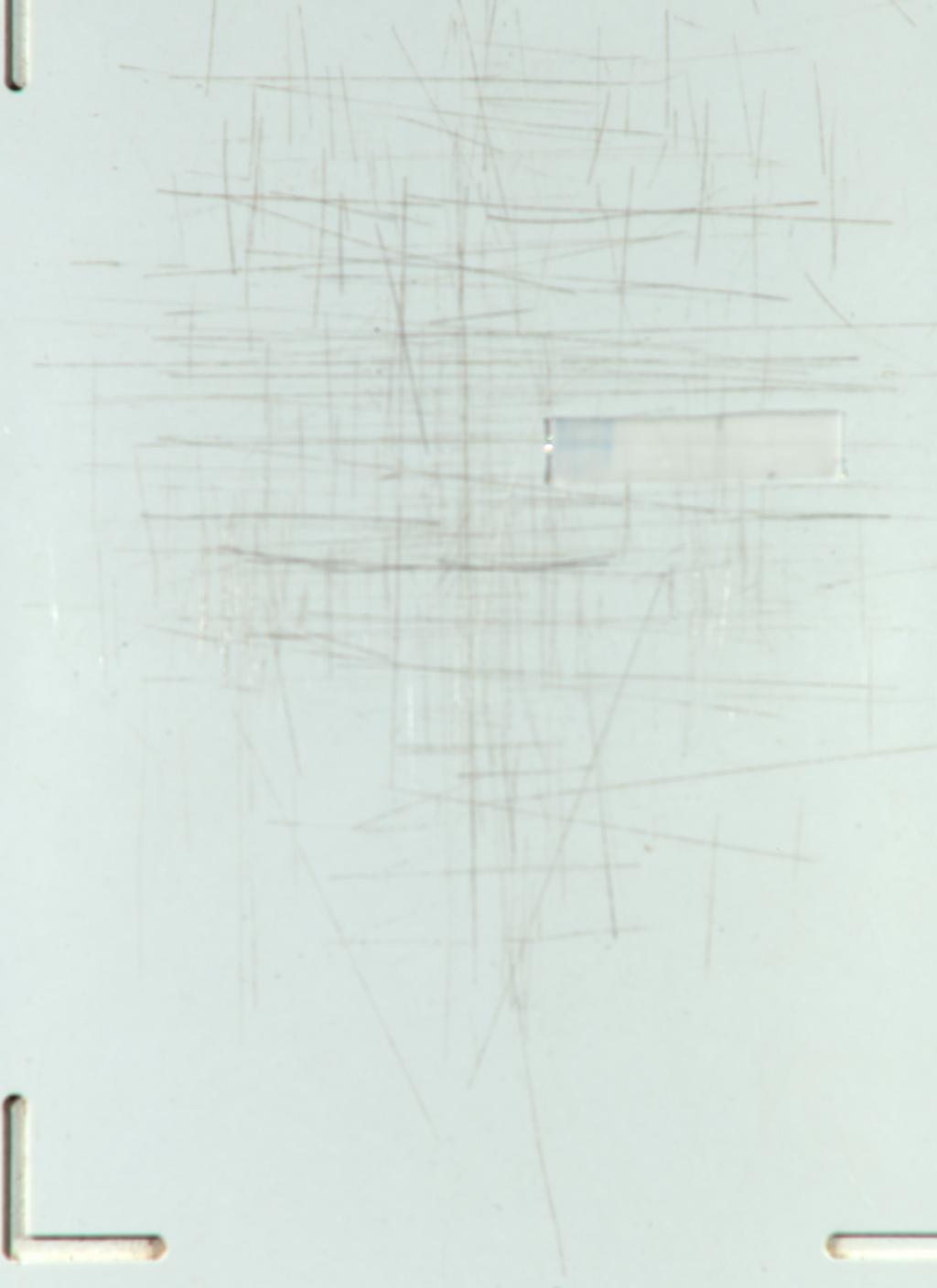

Supplement: Supplemental Information 6 [file peerj-11-15041-s006.zip › Osteoclast-related-genes-raw data2/MMP9/MMP9-3/MMP9-3-3.jpg]

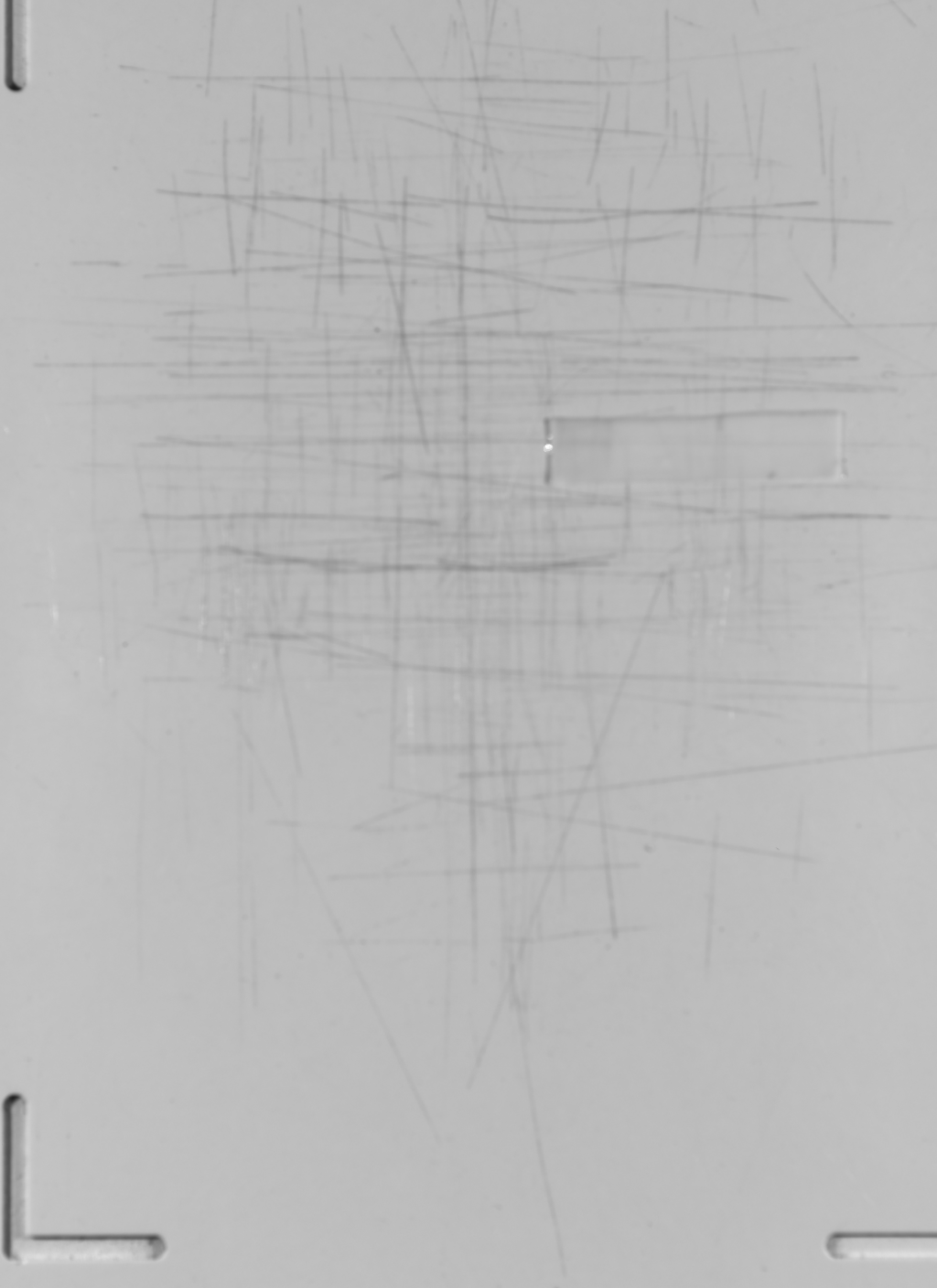

Supplement: Supplemental Information 6 [file peerj-11-15041-s006.zip › Osteoclast-related-genes-raw data2/MMP9/MMP9-3/MMP9-3-4.tif]

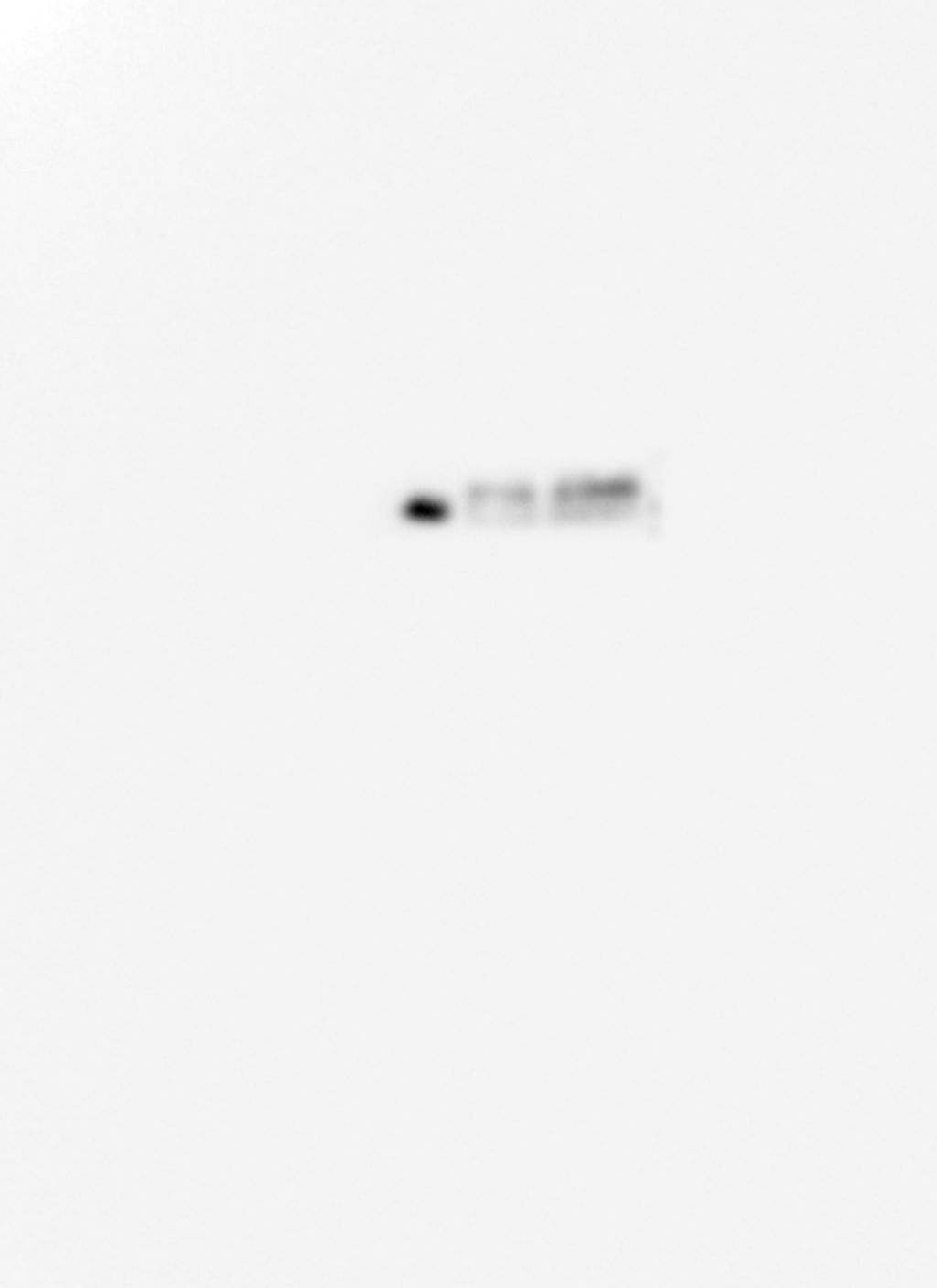

Supplement: Supplemental Information 6 [file peerj-11-15041-s006.zip › Osteoclast-related-genes-raw data2/NFATC1/NFATC1-1/NFATC1-1-1.tif]

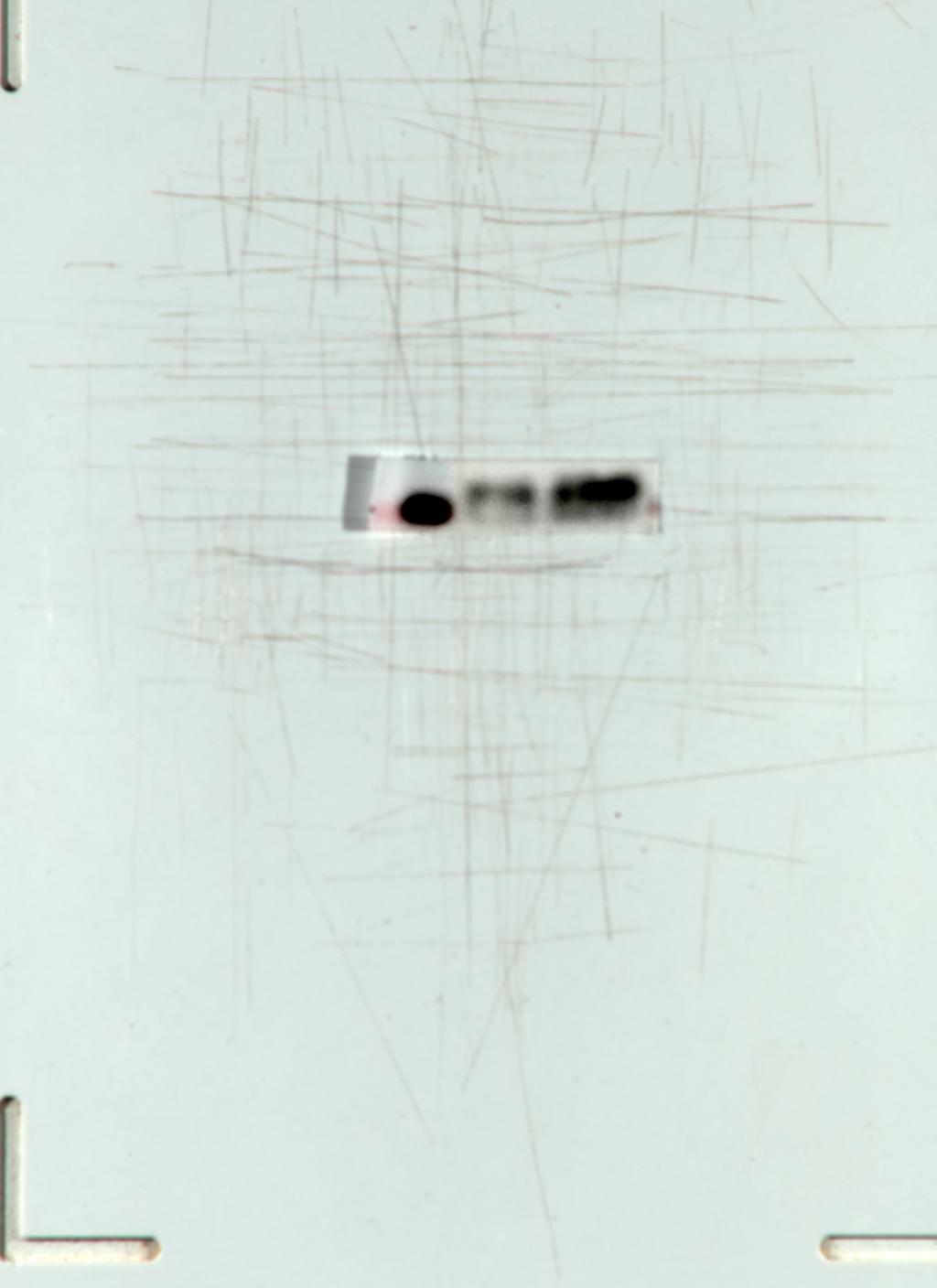

Supplement: Supplemental Information 6 [file peerj-11-15041-s006.zip › Osteoclast-related-genes-raw data2/NFATC1/NFATC1-1/NFATC1-1-2.jpg]

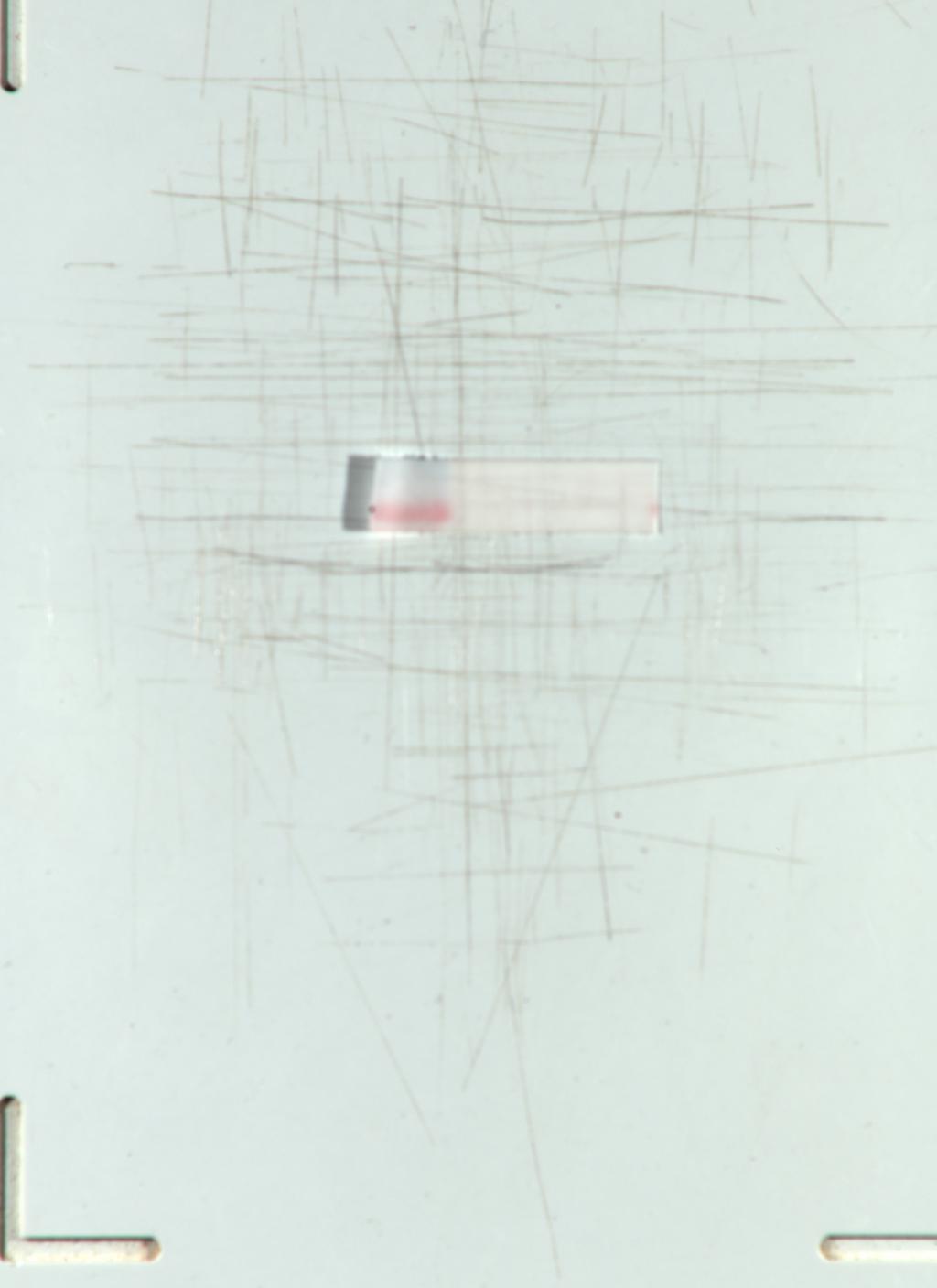

Supplement: Supplemental Information 6 [file peerj-11-15041-s006.zip › Osteoclast-related-genes-raw data2/NFATC1/NFATC1-1/NFATC1-1-3.jpg]

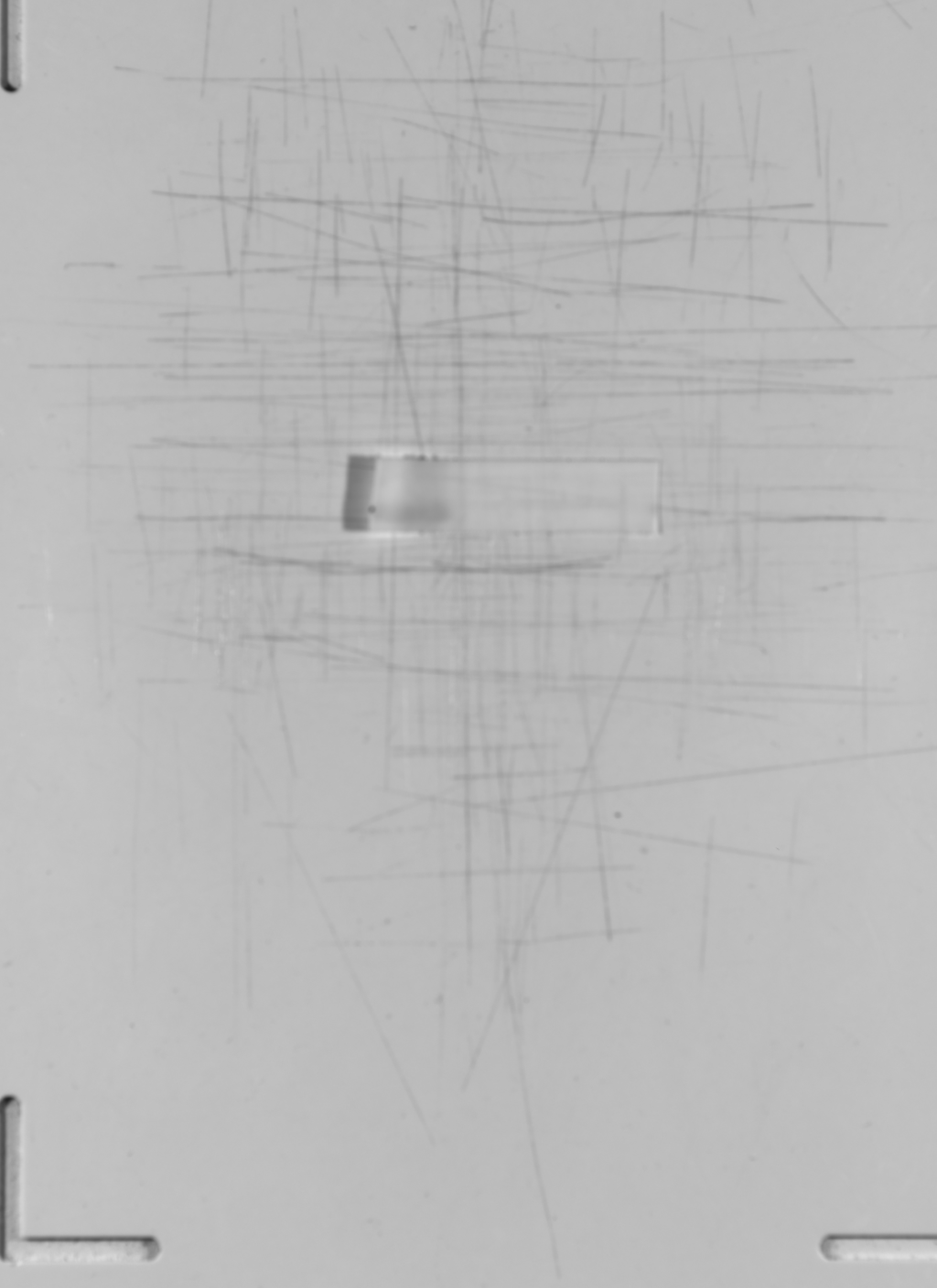

Supplement: Supplemental Information 6 [file peerj-11-15041-s006.zip › Osteoclast-related-genes-raw data2/NFATC1/NFATC1-1/NFATC1-1-4.tif]

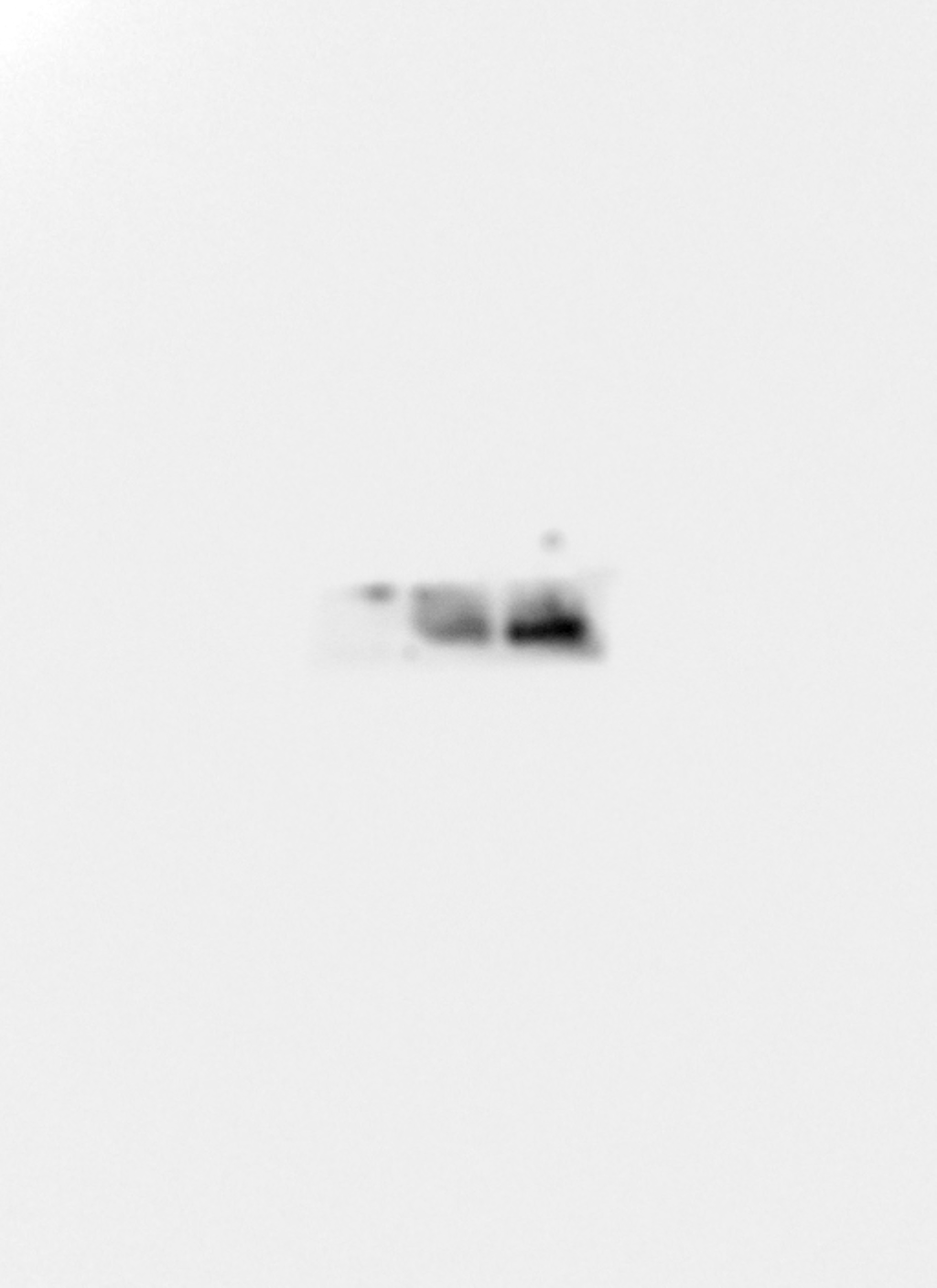

Supplement: Supplemental Information 6 [file peerj-11-15041-s006.zip › Osteoclast-related-genes-raw data2/NFATC1/NFATC1-2/NFATC1-2-1.tif]

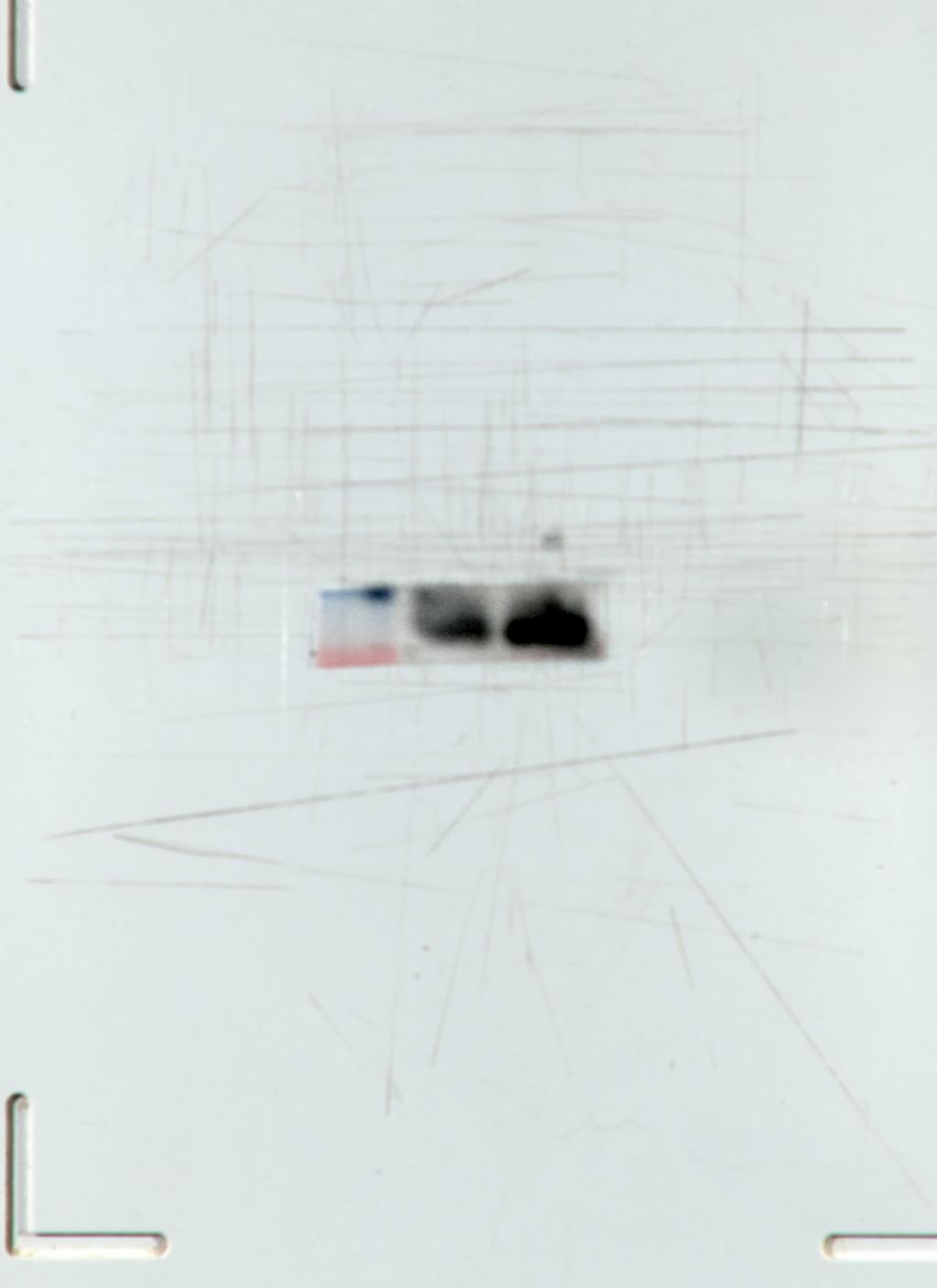

Supplement: Supplemental Information 6 [file peerj-11-15041-s006.zip › Osteoclast-related-genes-raw data2/NFATC1/NFATC1-2/NFATC1-2-2.jpg]

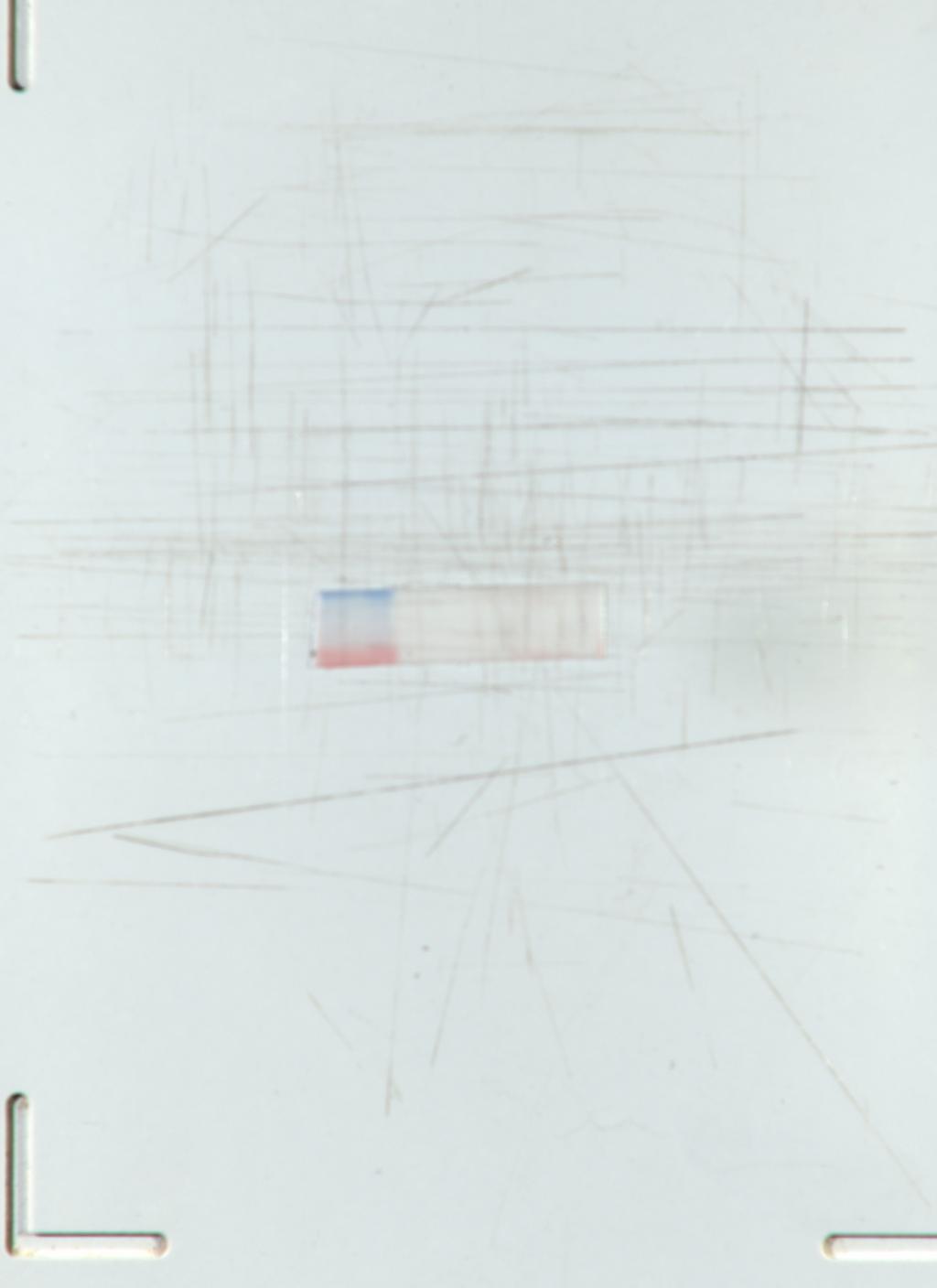

Supplement: Supplemental Information 6 [file peerj-11-15041-s006.zip › Osteoclast-related-genes-raw data2/NFATC1/NFATC1-2/NFATC1-2-3.jpg]

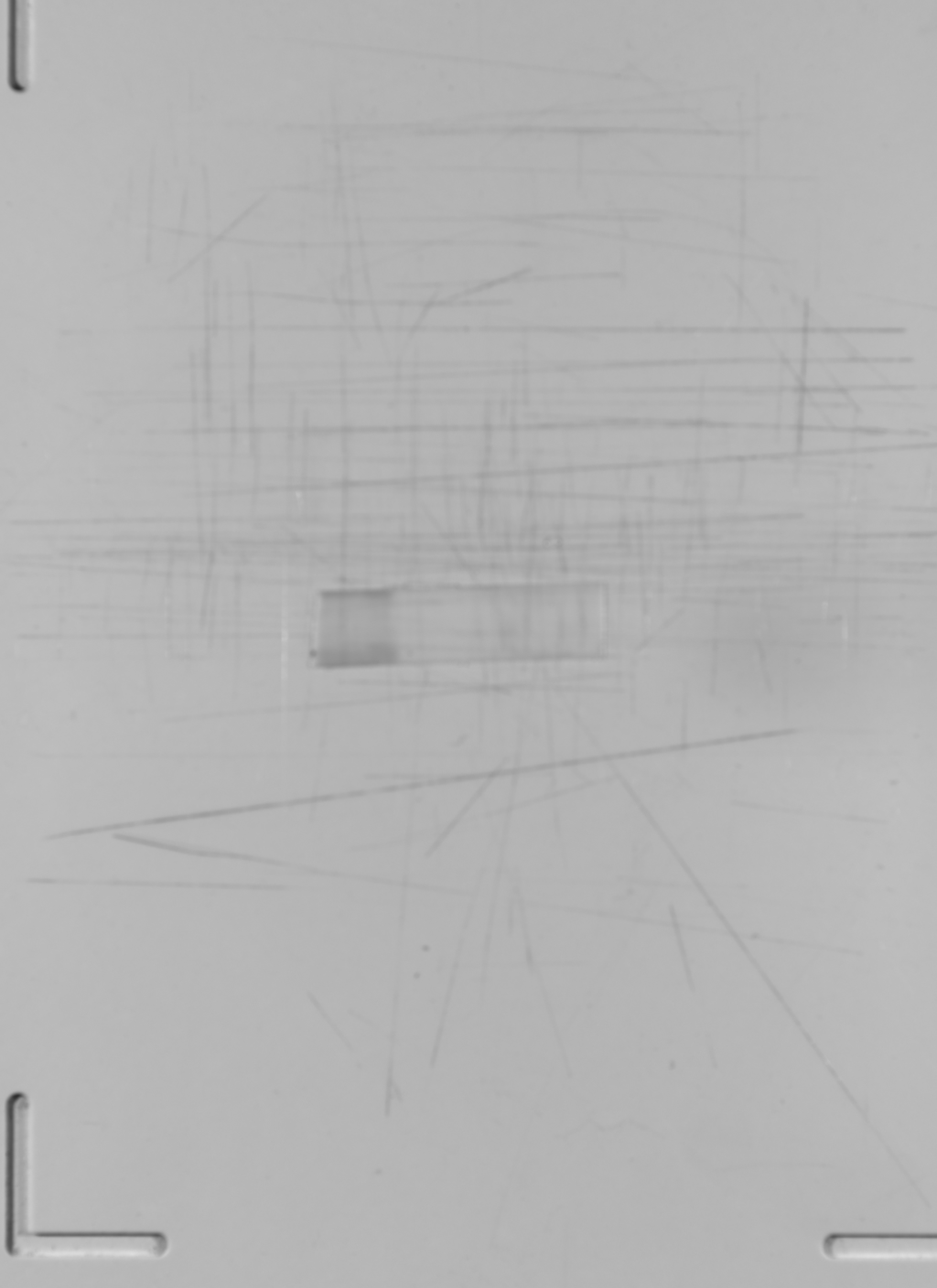

Supplement: Supplemental Information 6 [file peerj-11-15041-s006.zip › Osteoclast-related-genes-raw data2/NFATC1/NFATC1-2/NFATC1-2-4.tif]

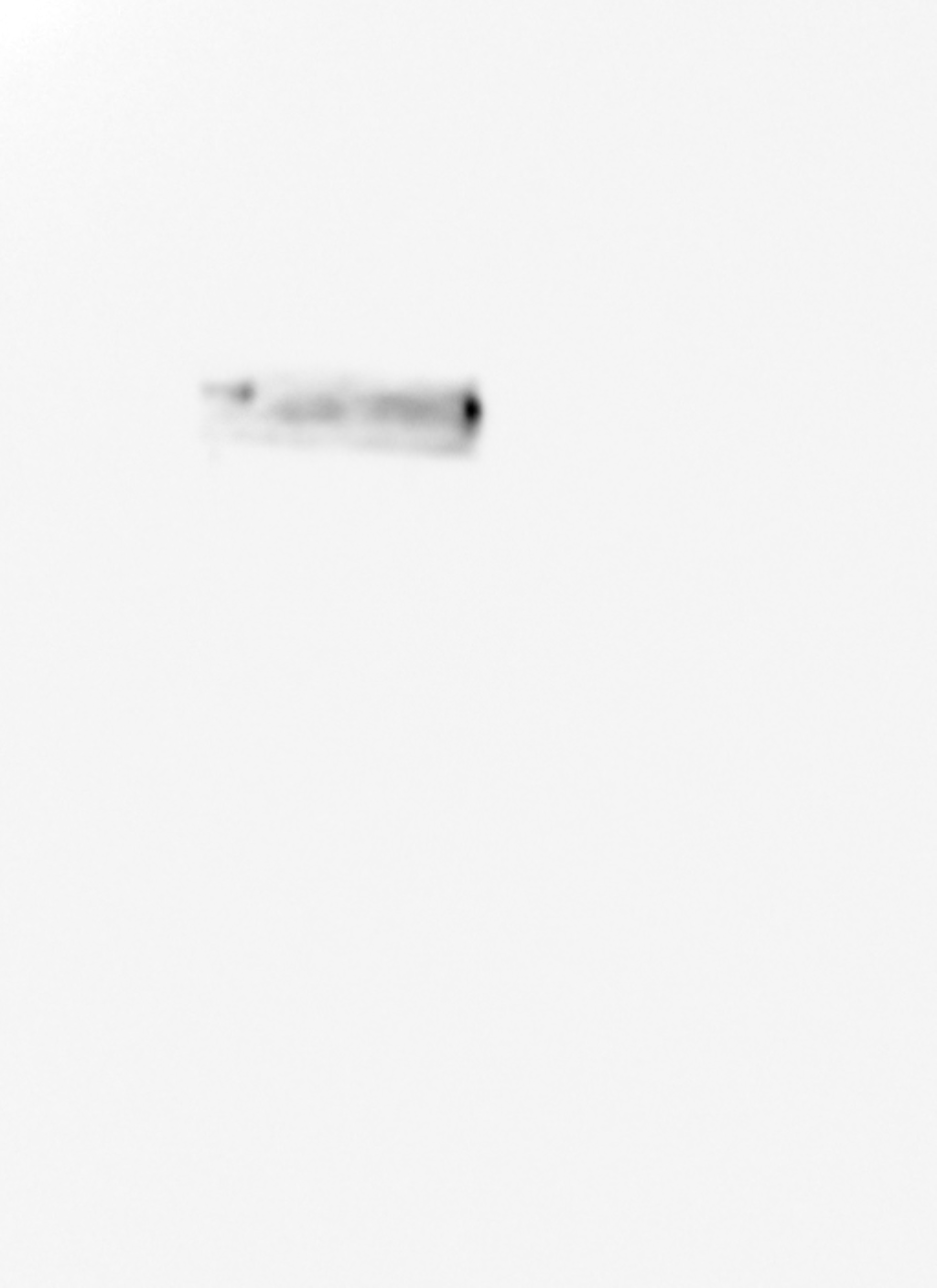

Supplement: Supplemental Information 6 [file peerj-11-15041-s006.zip › Osteoclast-related-genes-raw data2/NFATC1/NFATC1-3/NFATC1-3-1.tif]

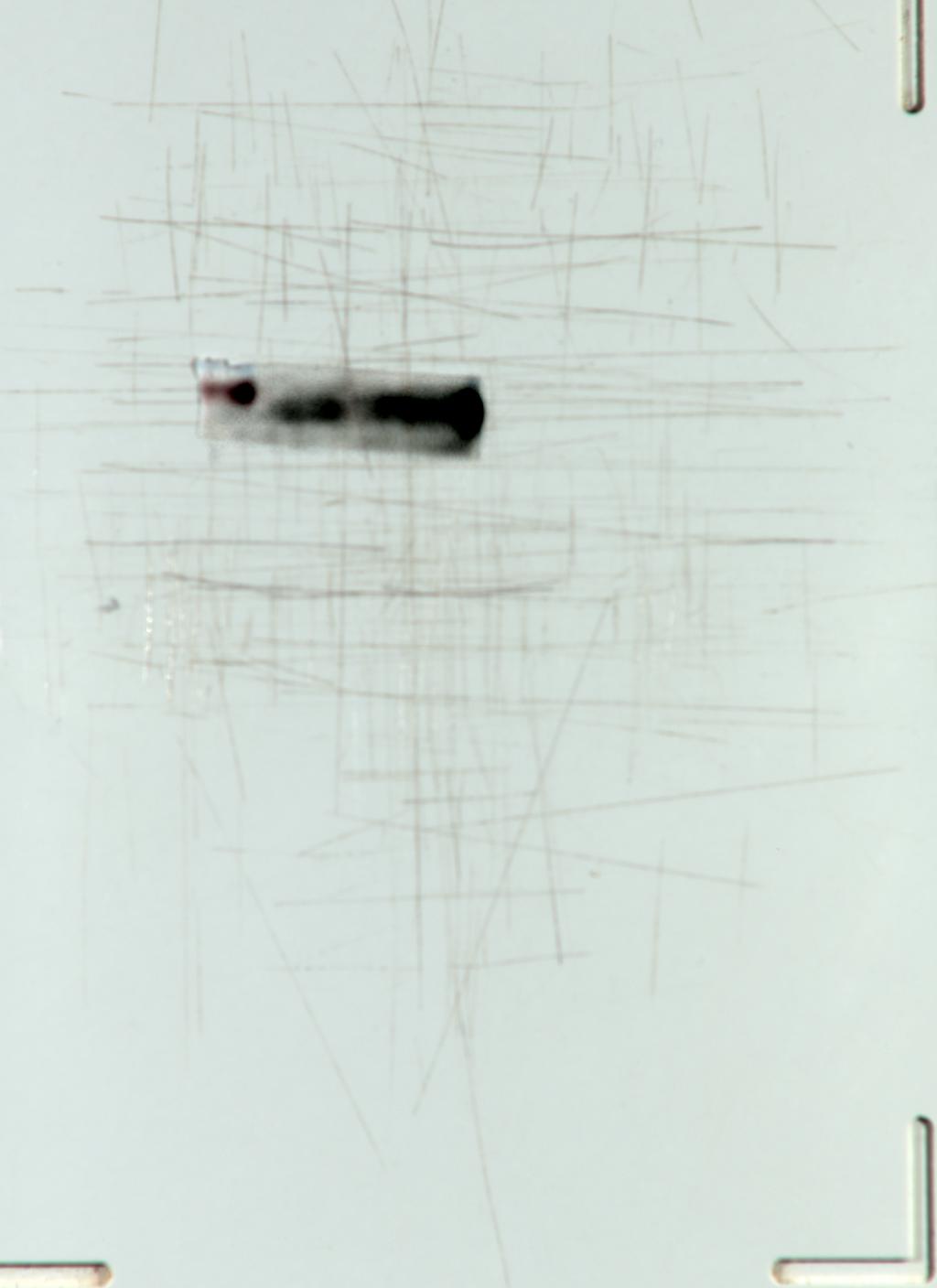

Supplement: Supplemental Information 6 [file peerj-11-15041-s006.zip › Osteoclast-related-genes-raw data2/NFATC1/NFATC1-3/NFATC1-3-2.jpg]

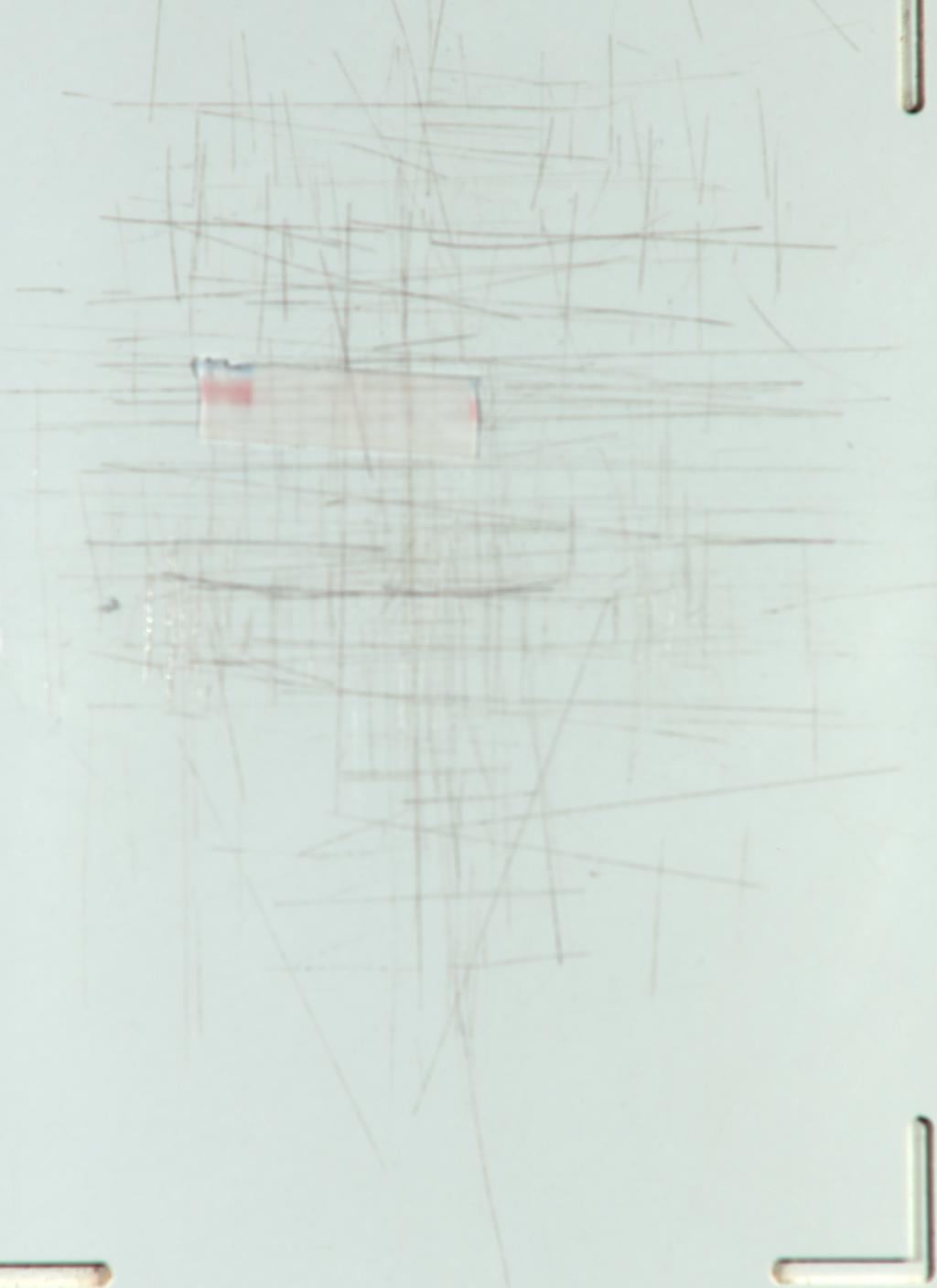

Supplement: Supplemental Information 6 [file peerj-11-15041-s006.zip › Osteoclast-related-genes-raw data2/NFATC1/NFATC1-3/NFATC1-3-3.jpg]

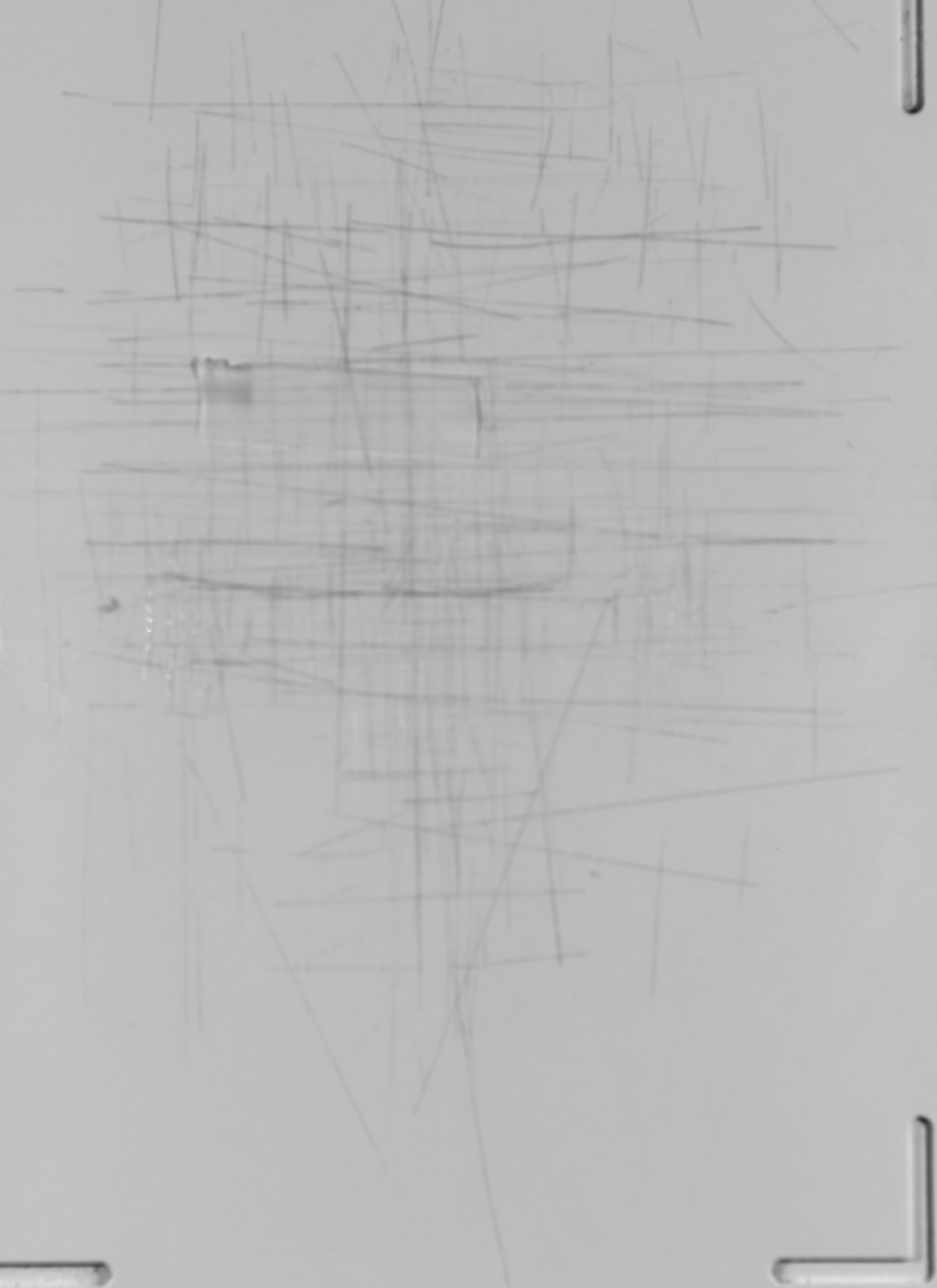

Supplement: Supplemental Information 6 [file peerj-11-15041-s006.zip › Osteoclast-related-genes-raw data2/NFATC1/NFATC1-3/NFATC1-3-4.tif]

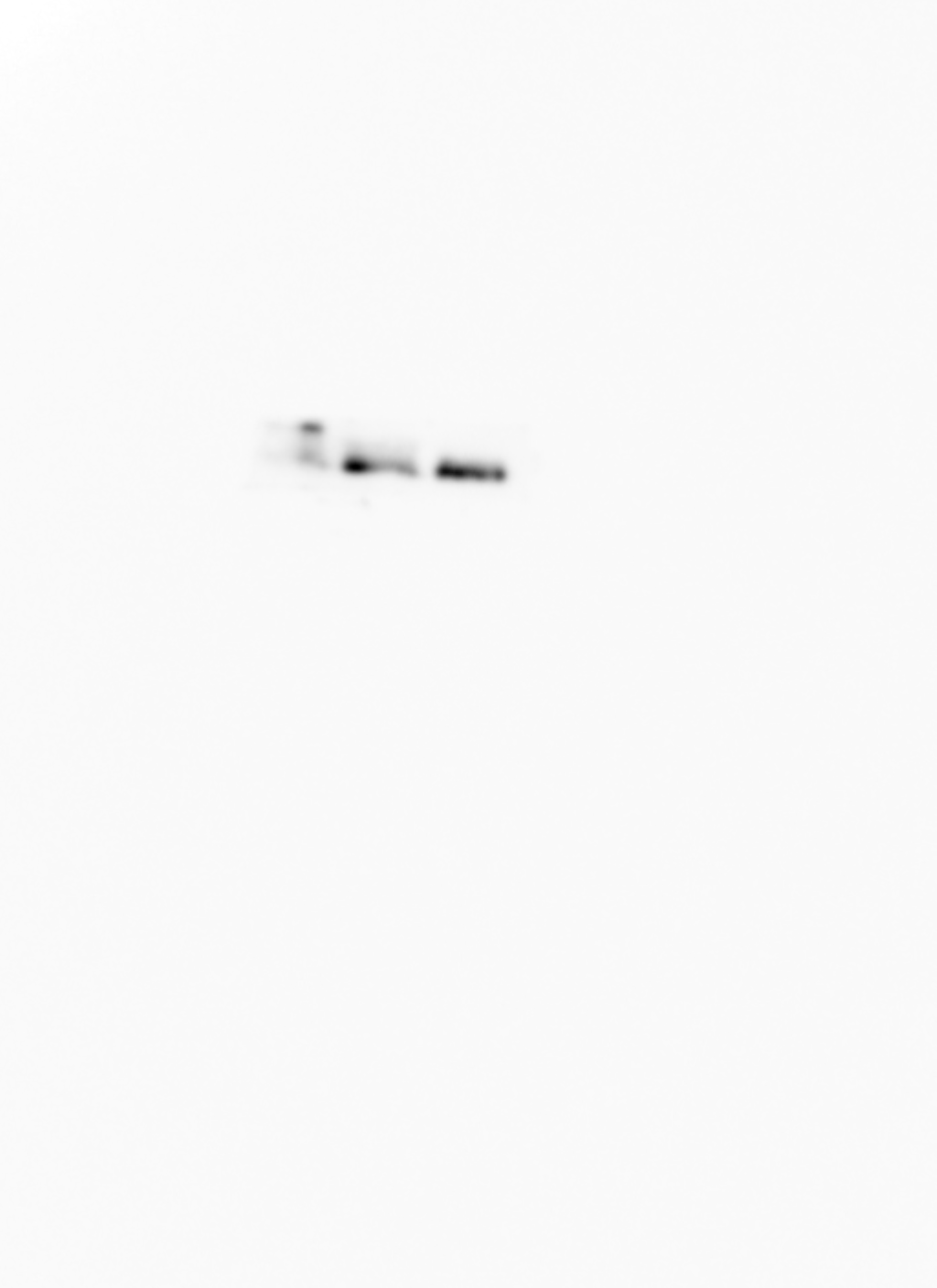

Supplement: Supplemental Information 7 [file peerj-11-15041-s007.zip › Osteoclast-related-genes-raw data3/C-FOS/C-FOS-1/C-FOS-1-1.tif]

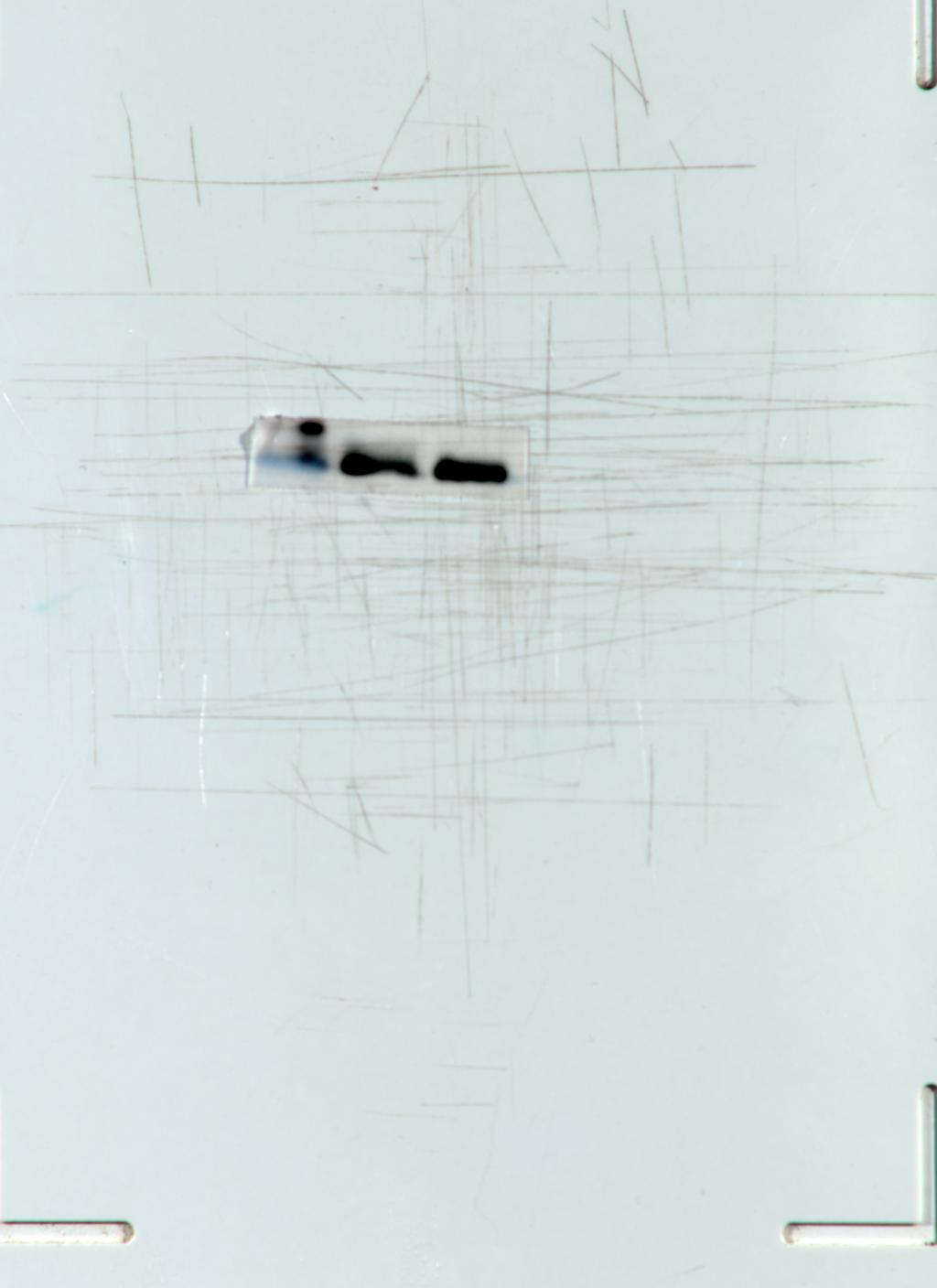

Supplement: Supplemental Information 7 [file peerj-11-15041-s007.zip › Osteoclast-related-genes-raw data3/C-FOS/C-FOS-1/C-FOS-1-2.jpg]

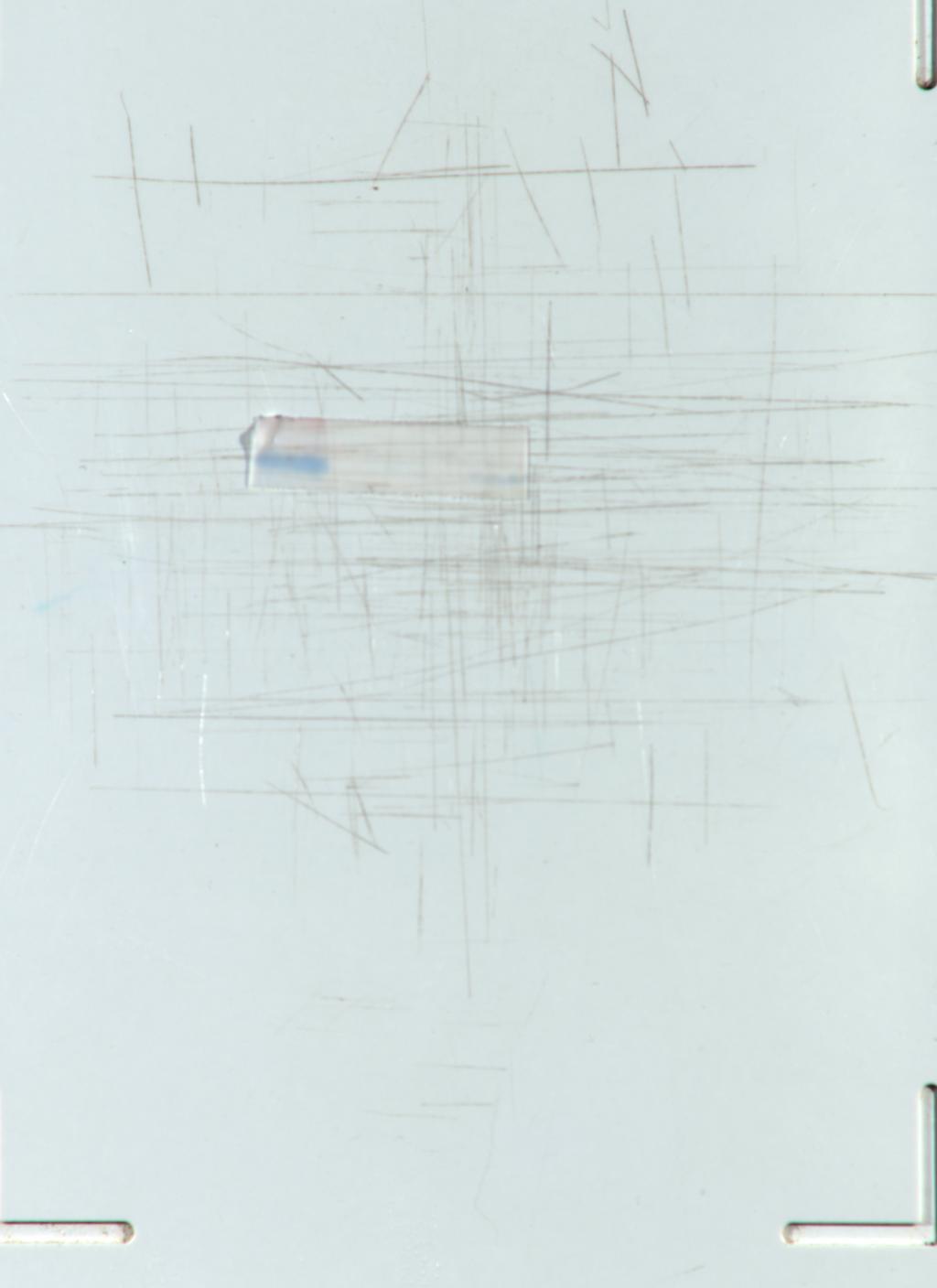

Supplement: Supplemental Information 7 [file peerj-11-15041-s007.zip › Osteoclast-related-genes-raw data3/C-FOS/C-FOS-1/C-FOS-1-3.jpg]
