# Supplementary figures and images for: CYP27A1 deficiency promoted osteoclast differentiation (part 2 of 3)
Source: PeerJ. 2023 Mar 3;11:e15041. doi: 10.7717/peerj.15041 (PMC9987298; doi:10.7717/peerj.15041)

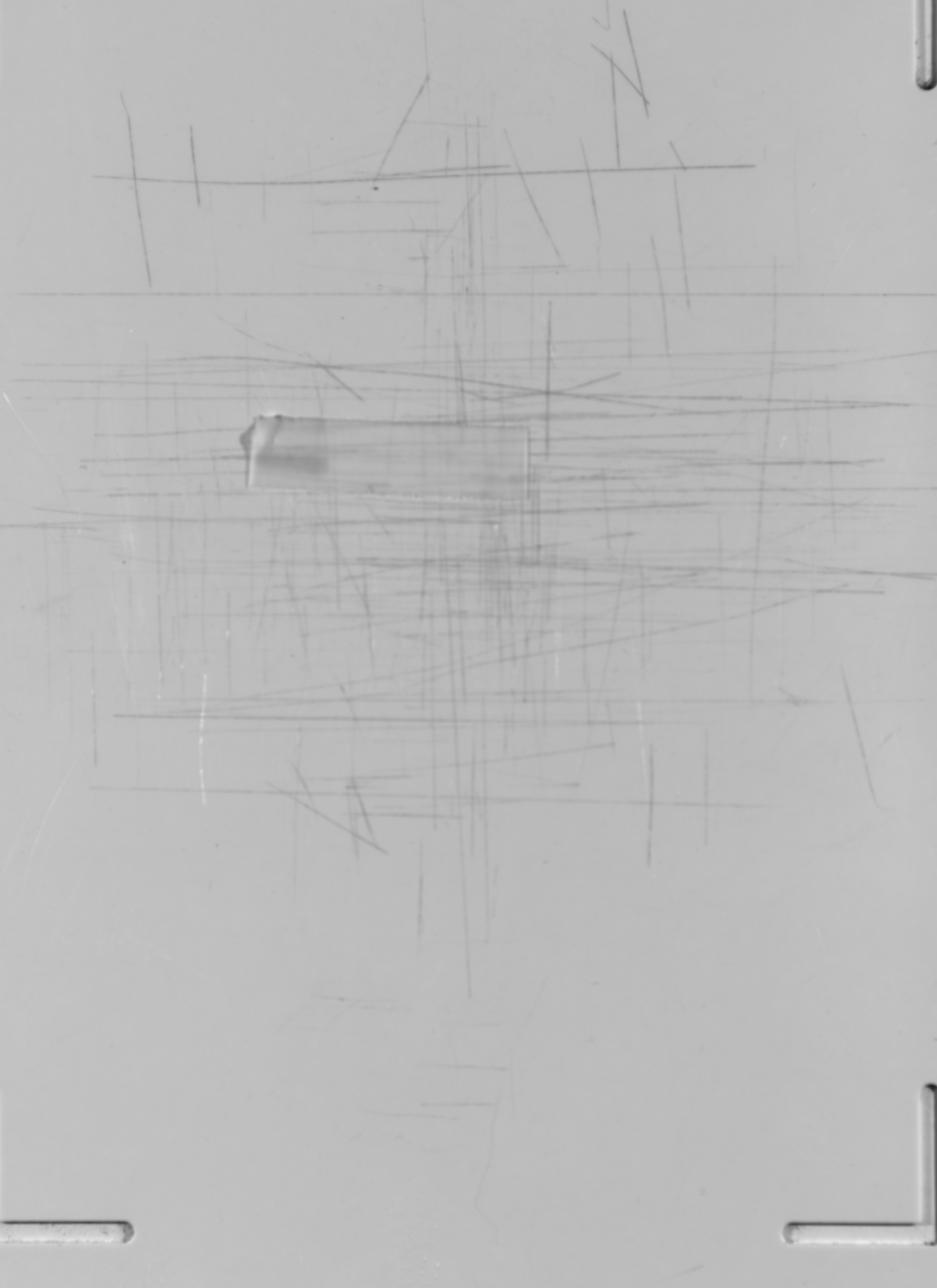

Supplement: Supplemental Information 7 [file peerj-11-15041-s007.zip › Osteoclast-related-genes-raw data3/C-FOS/C-FOS-1/C-FOS-1-4.tif]

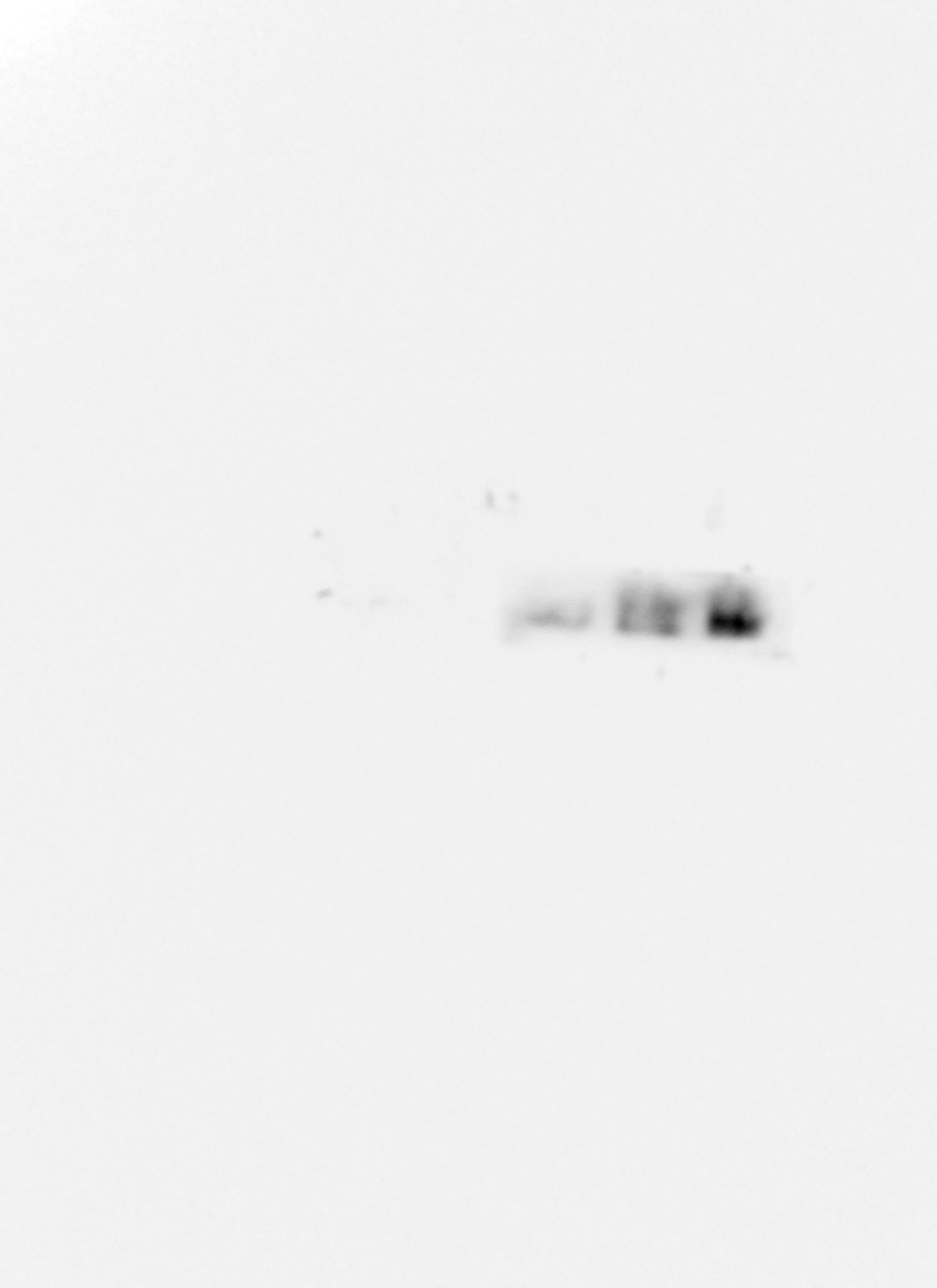

Supplement: Supplemental Information 7 [file peerj-11-15041-s007.zip › Osteoclast-related-genes-raw data3/C-FOS/C-FOS-2/C-FOS-2-1.tif]

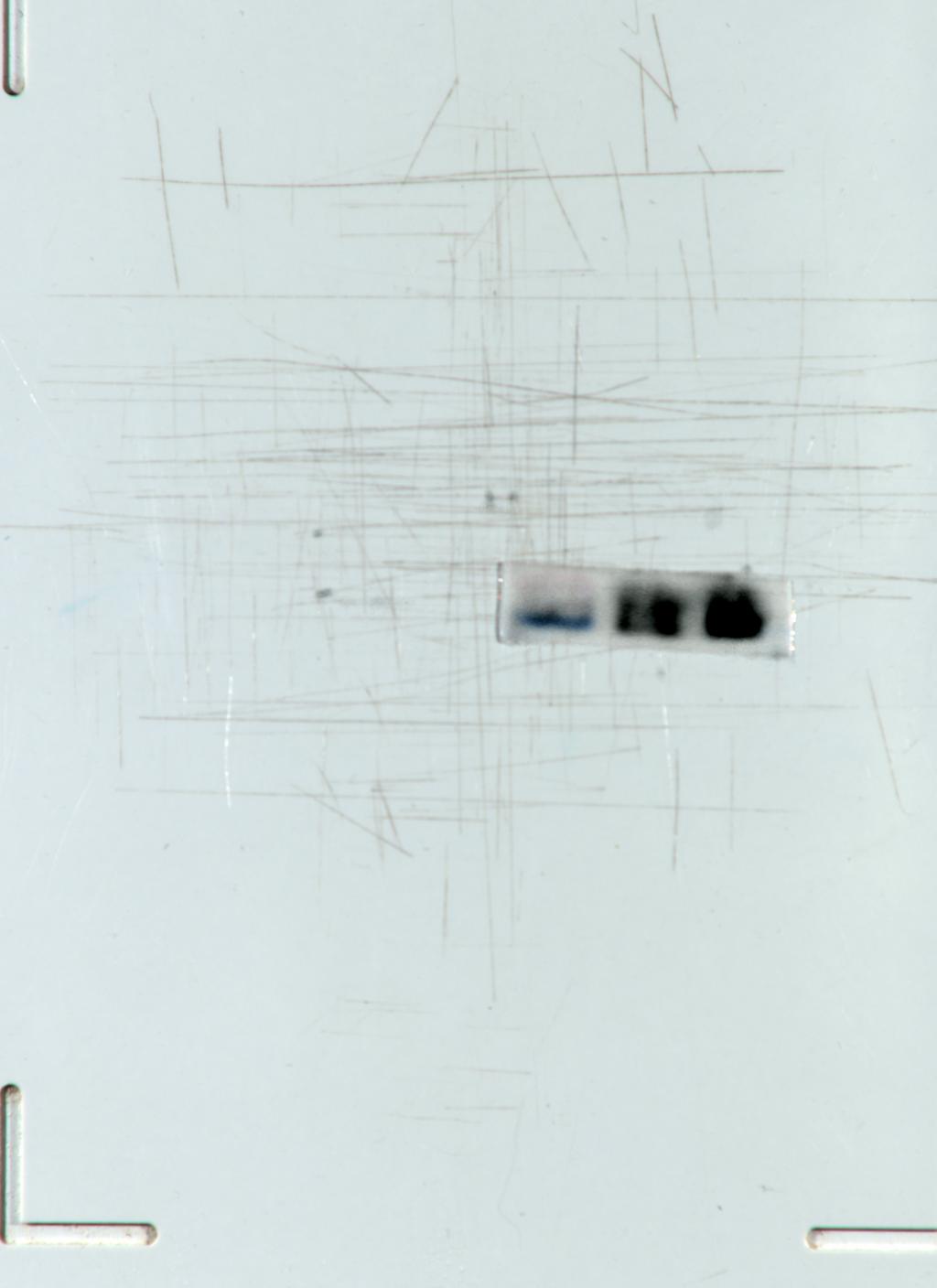

Supplement: Supplemental Information 7 [file peerj-11-15041-s007.zip › Osteoclast-related-genes-raw data3/C-FOS/C-FOS-2/C-FOS-2-2.jpg]

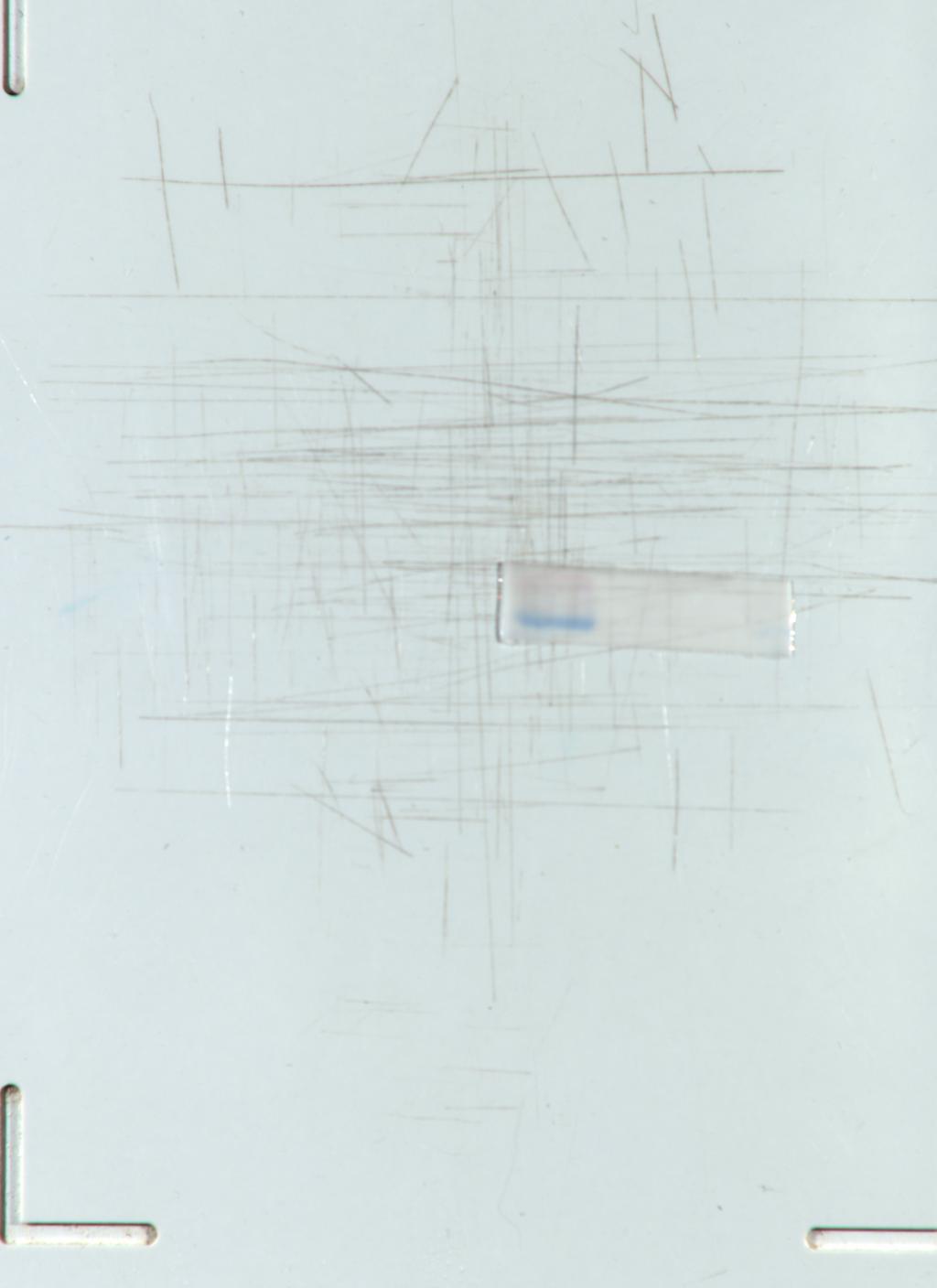

Supplement: Supplemental Information 7 [file peerj-11-15041-s007.zip › Osteoclast-related-genes-raw data3/C-FOS/C-FOS-2/C-FOS-2-3.jpg]

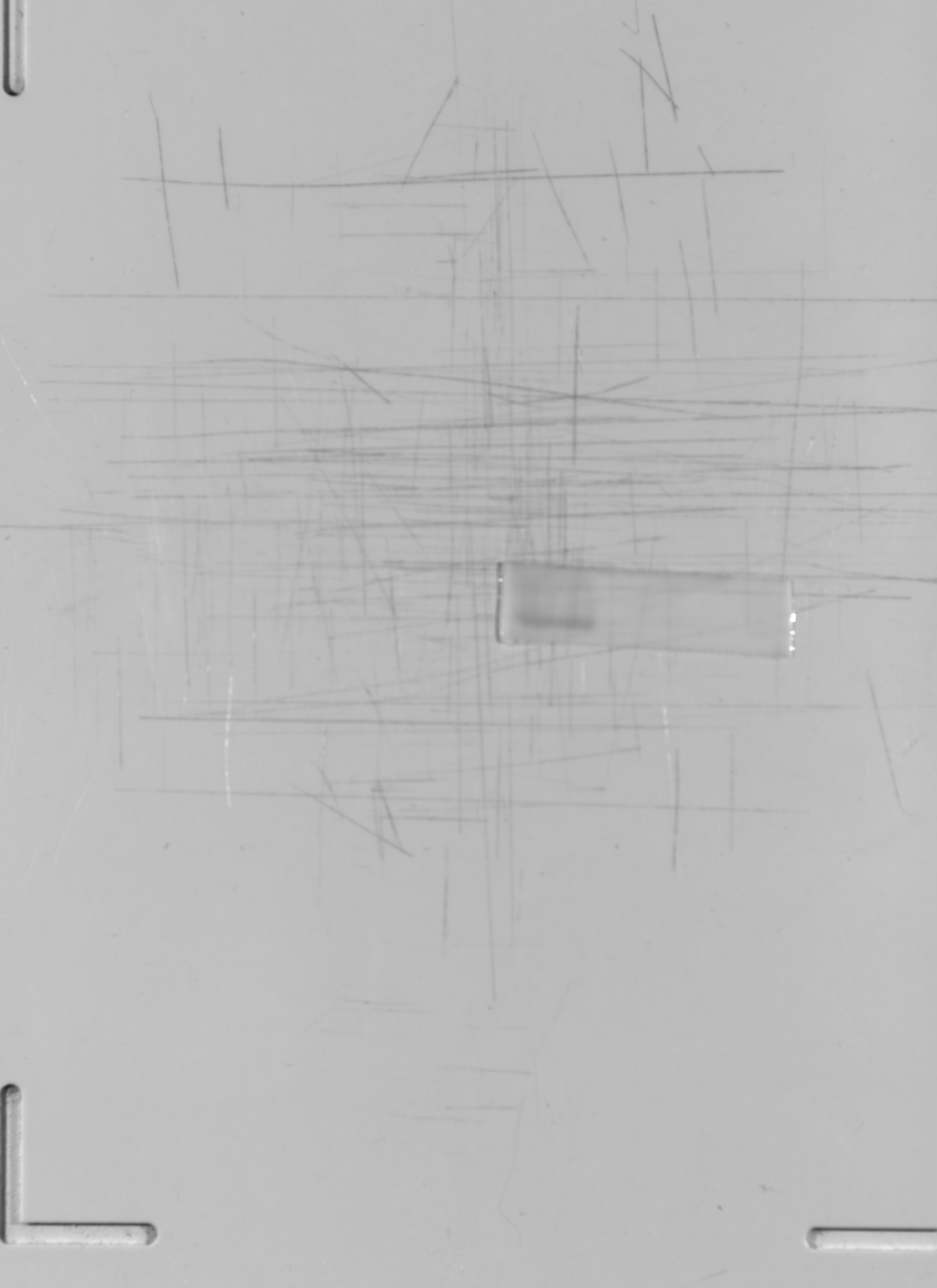

Supplement: Supplemental Information 7 [file peerj-11-15041-s007.zip › Osteoclast-related-genes-raw data3/C-FOS/C-FOS-2/C-FOS-2-3.tif]

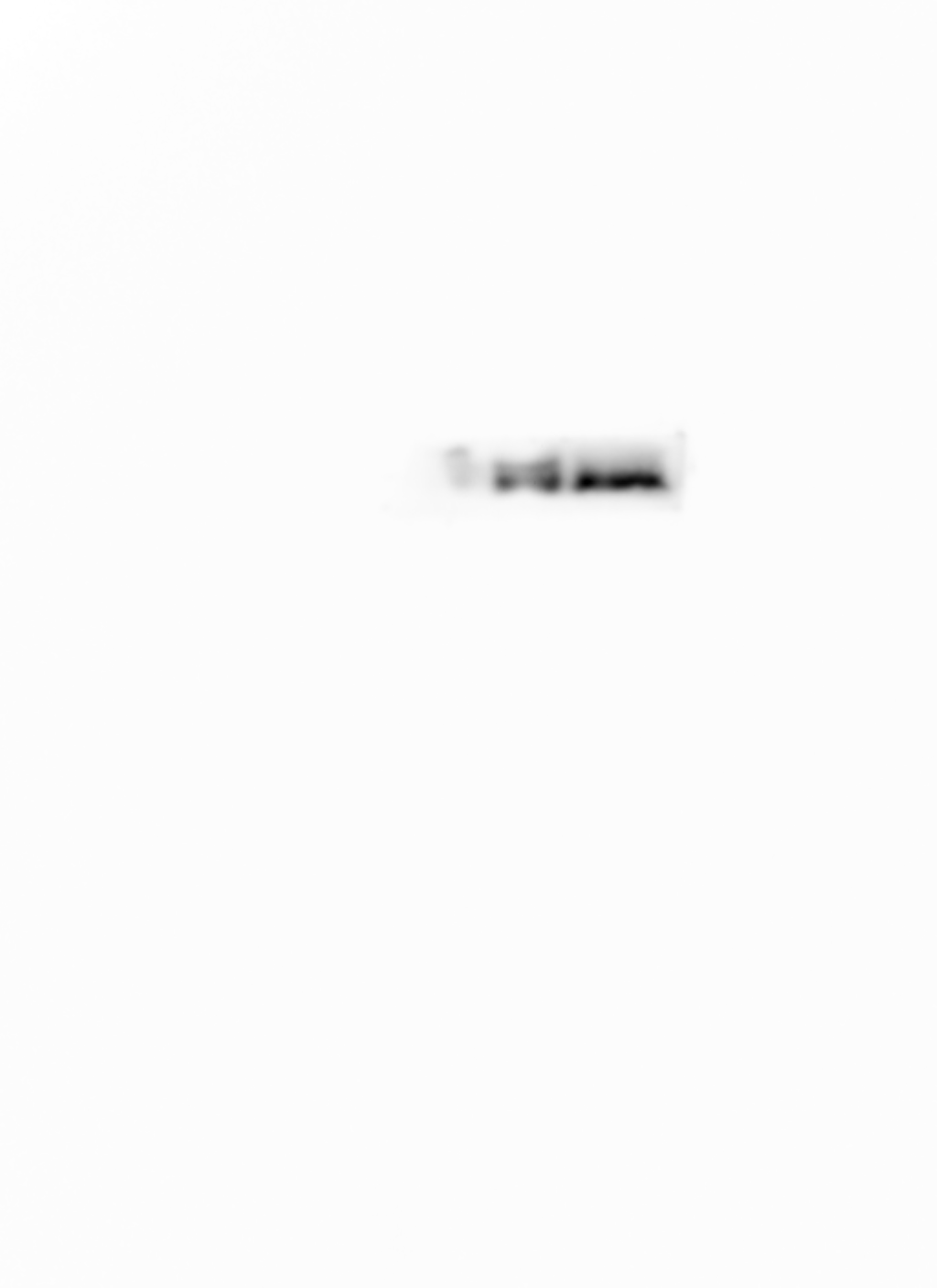

Supplement: Supplemental Information 7 [file peerj-11-15041-s007.zip › Osteoclast-related-genes-raw data3/C-FOS/C-FOS-3/C-FOS-3-1.tif]

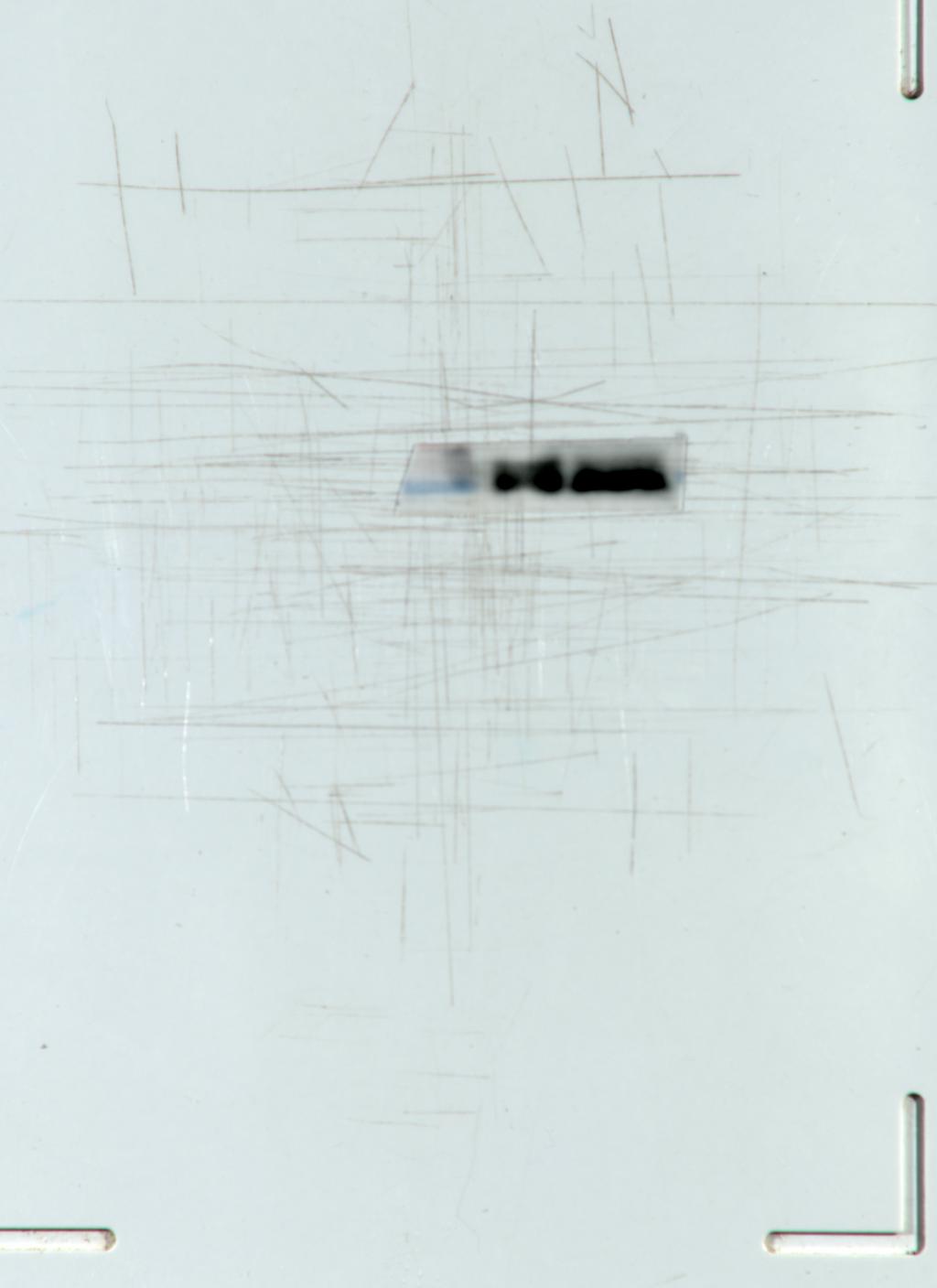

Supplement: Supplemental Information 7 [file peerj-11-15041-s007.zip › Osteoclast-related-genes-raw data3/C-FOS/C-FOS-3/C-FOS-3-2.jpg]

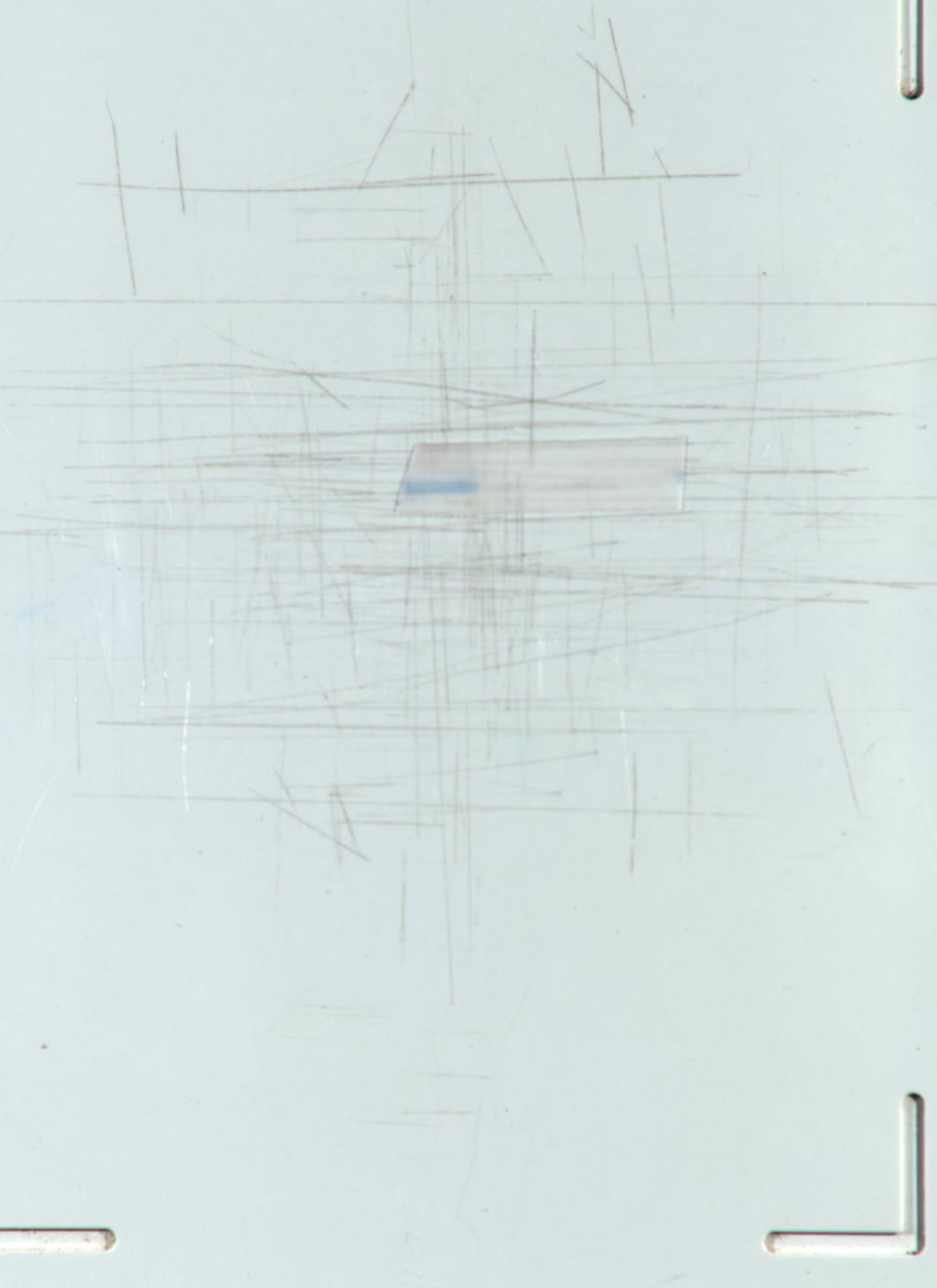

Supplement: Supplemental Information 7 [file peerj-11-15041-s007.zip › Osteoclast-related-genes-raw data3/C-FOS/C-FOS-3/C-FOS-3-3.jpg]

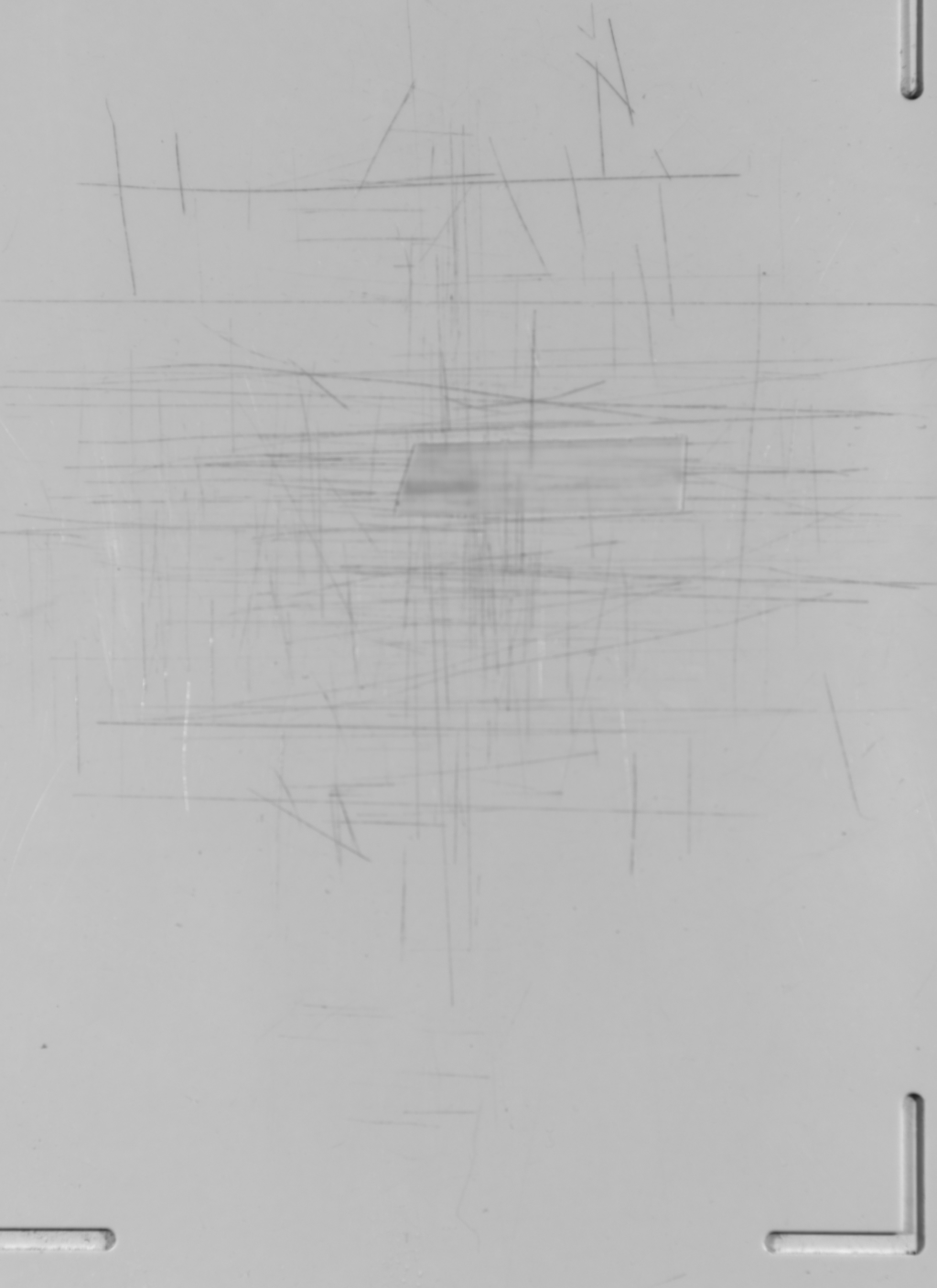

Supplement: Supplemental Information 7 [file peerj-11-15041-s007.zip › Osteoclast-related-genes-raw data3/C-FOS/C-FOS-3/C-FOS-3-4.tif]

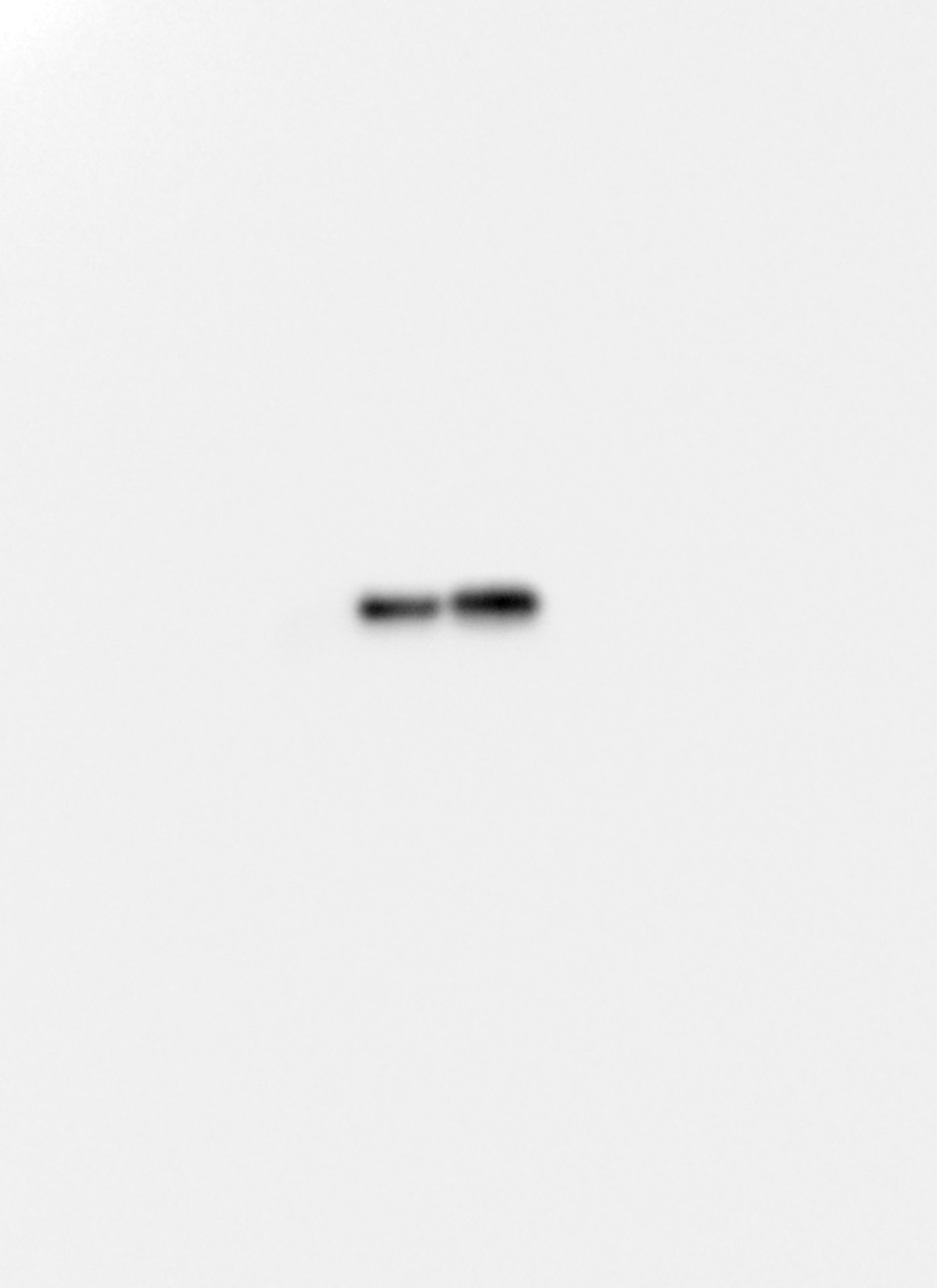

Supplement: Supplemental Information 7 [file peerj-11-15041-s007.zip › Osteoclast-related-genes-raw data3/TRAP/TRAP-1/TRAP-1-1.tif]

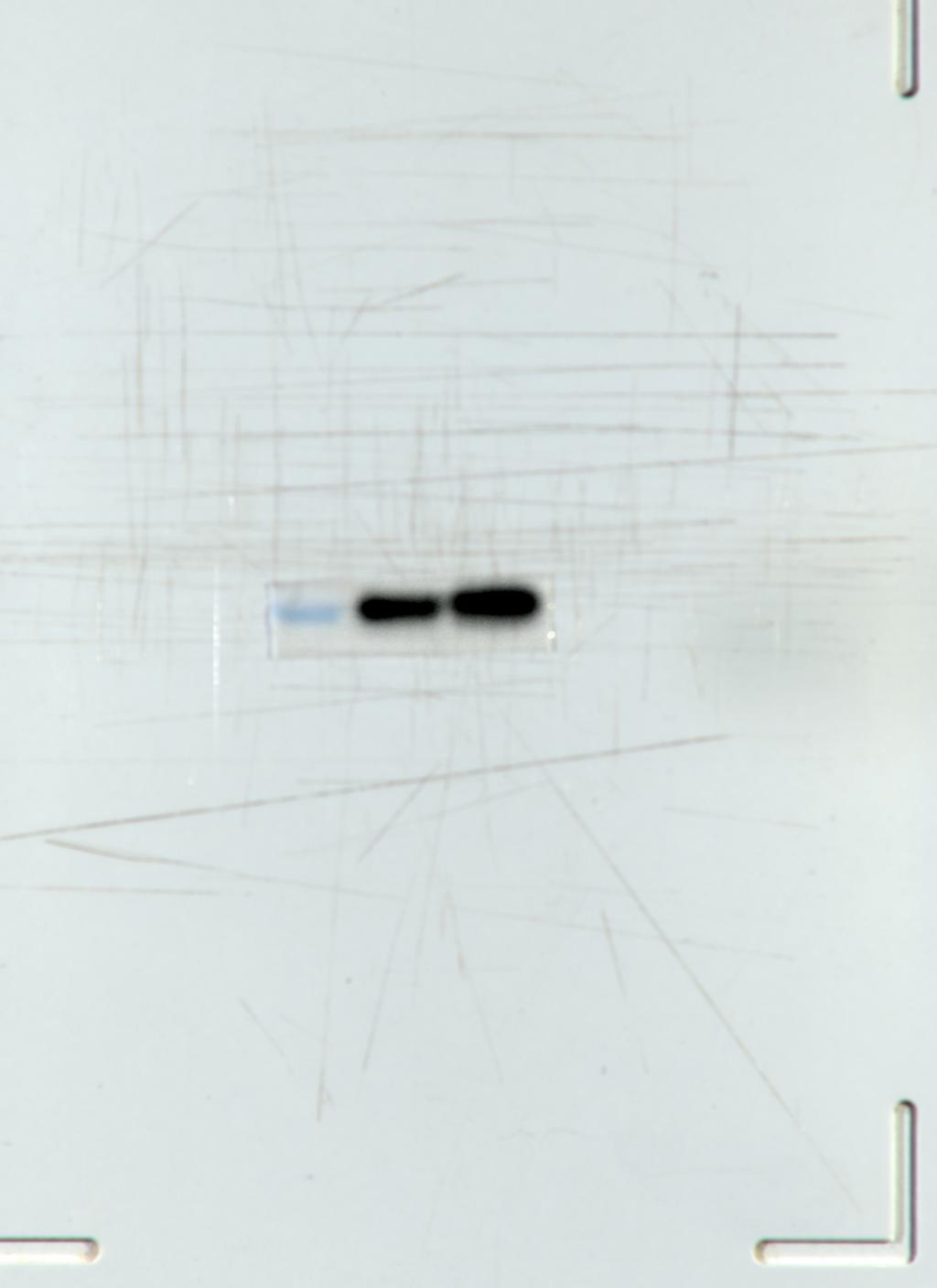

Supplement: Supplemental Information 7 [file peerj-11-15041-s007.zip › Osteoclast-related-genes-raw data3/TRAP/TRAP-1/TRAP-1-2.jpg]

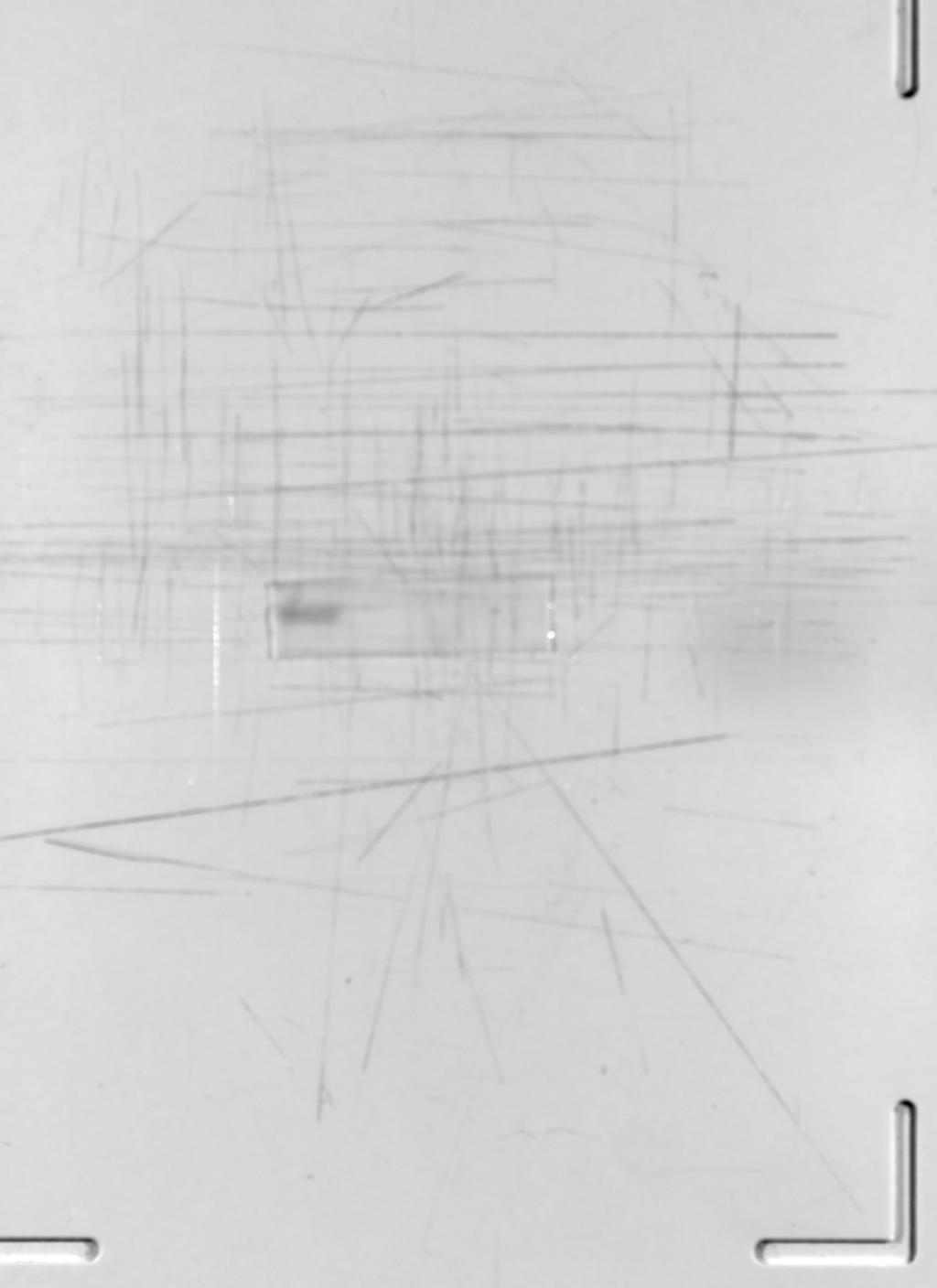

Supplement: Supplemental Information 7 [file peerj-11-15041-s007.zip › Osteoclast-related-genes-raw data3/TRAP/TRAP-1/TRAP-1-3.tif]

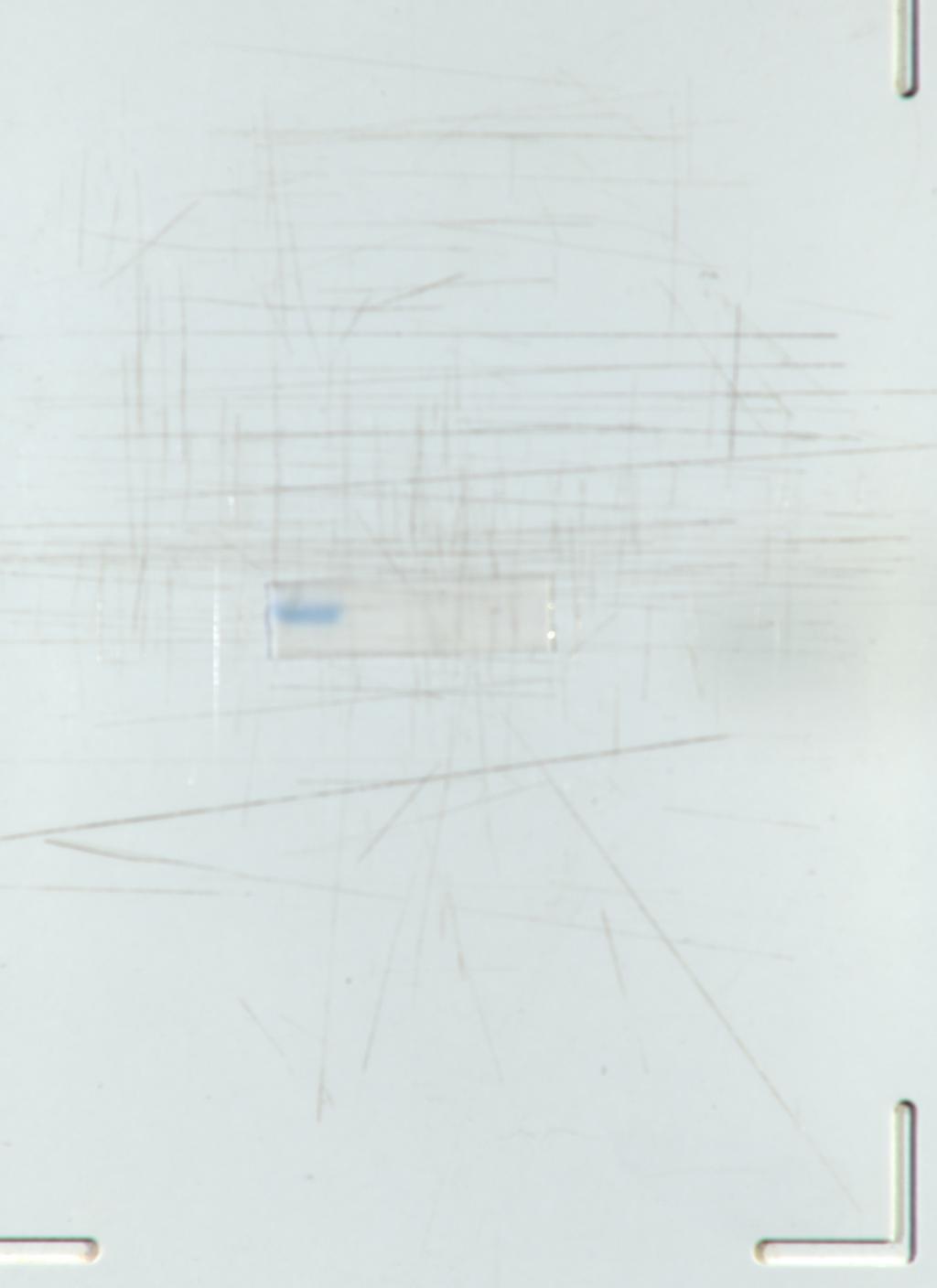

Supplement: Supplemental Information 7 [file peerj-11-15041-s007.zip › Osteoclast-related-genes-raw data3/TRAP/TRAP-1/TRAP-1-4.jpg]

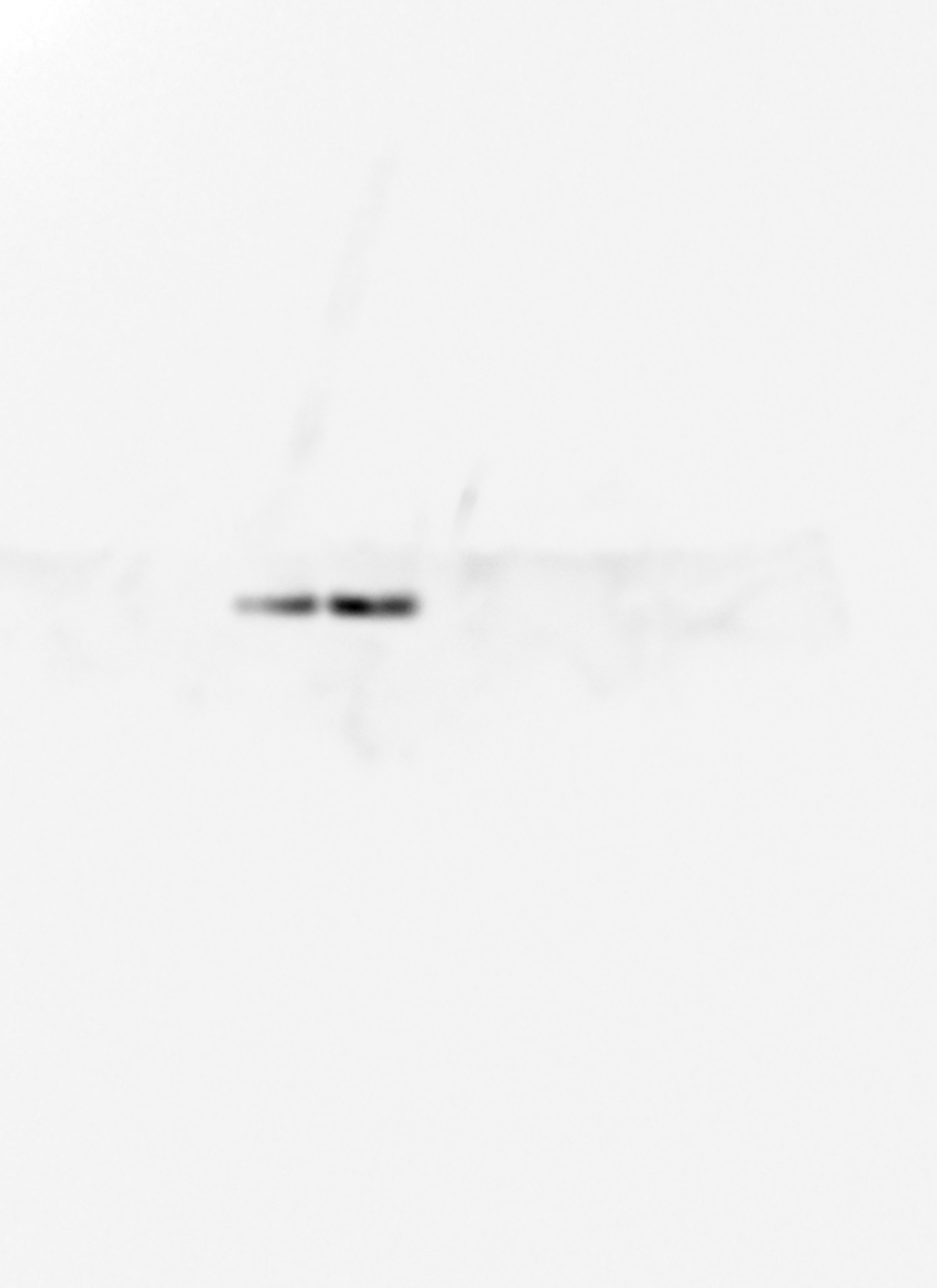

Supplement: Supplemental Information 7 [file peerj-11-15041-s007.zip › Osteoclast-related-genes-raw data3/TRAP/TRAP-2/TRAP-2-1.tif]

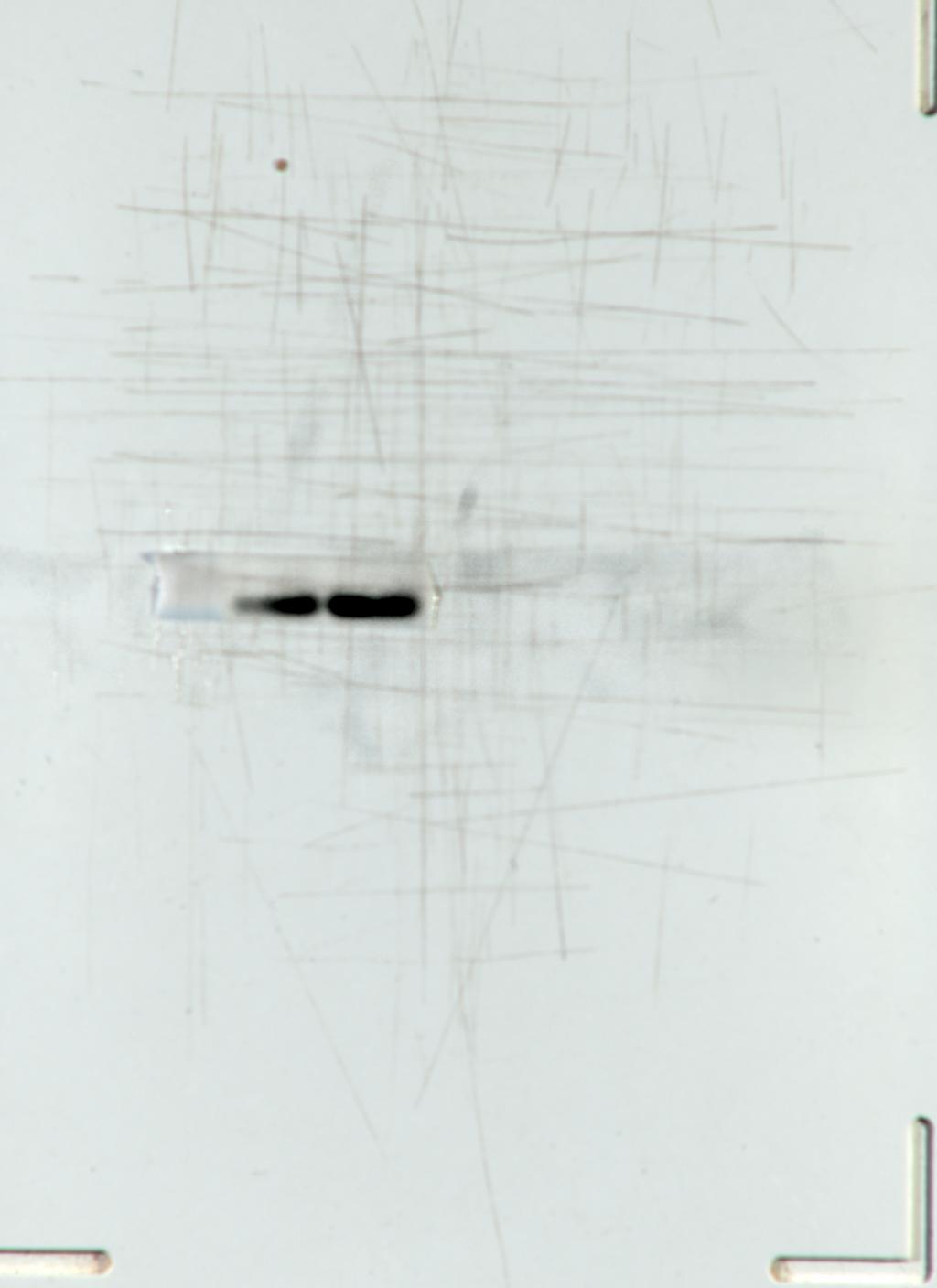

Supplement: Supplemental Information 7 [file peerj-11-15041-s007.zip › Osteoclast-related-genes-raw data3/TRAP/TRAP-2/TRAP-2-2.jpg]

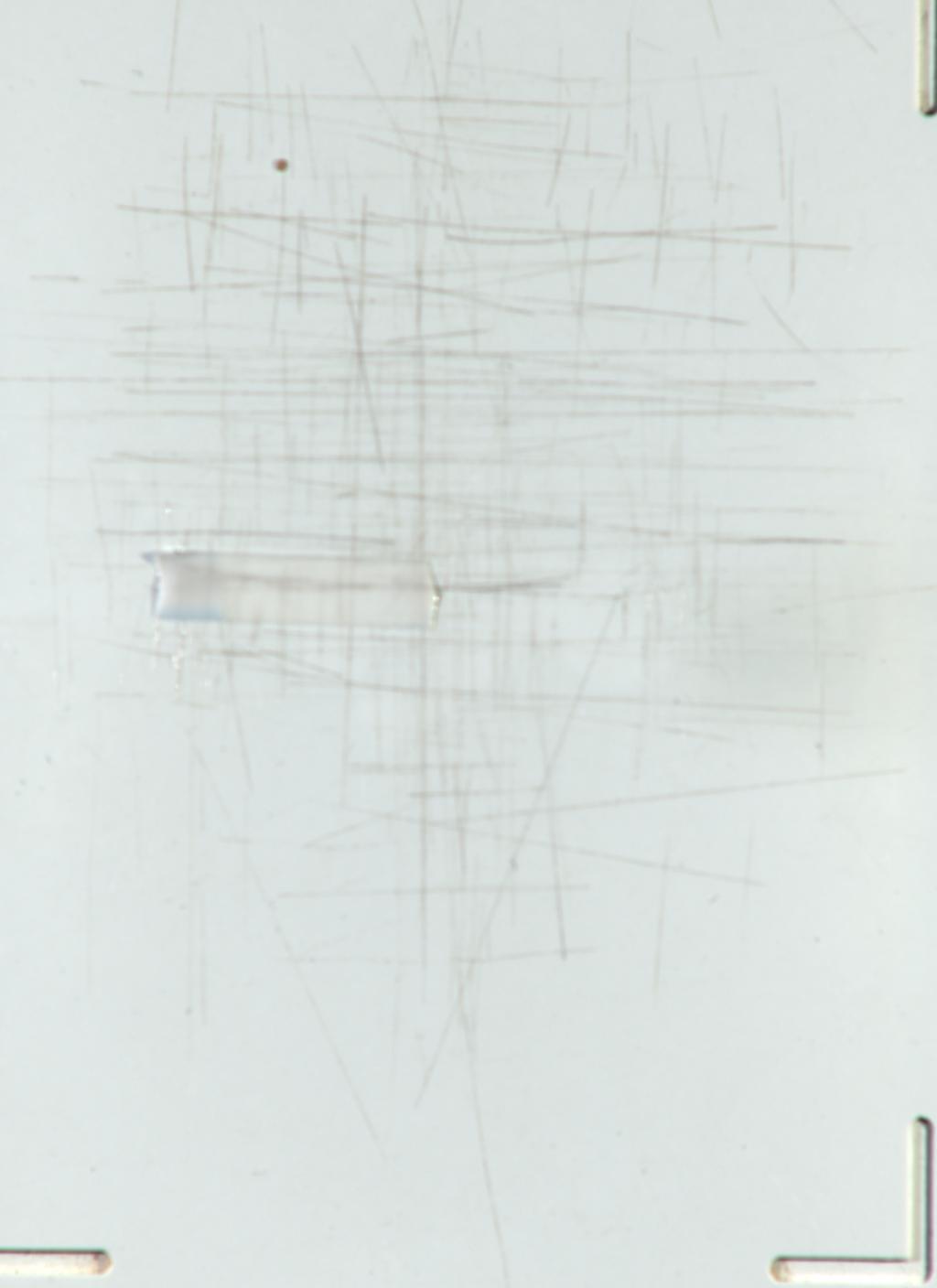

Supplement: Supplemental Information 7 [file peerj-11-15041-s007.zip › Osteoclast-related-genes-raw data3/TRAP/TRAP-2/TRAP-2-3.jpg]

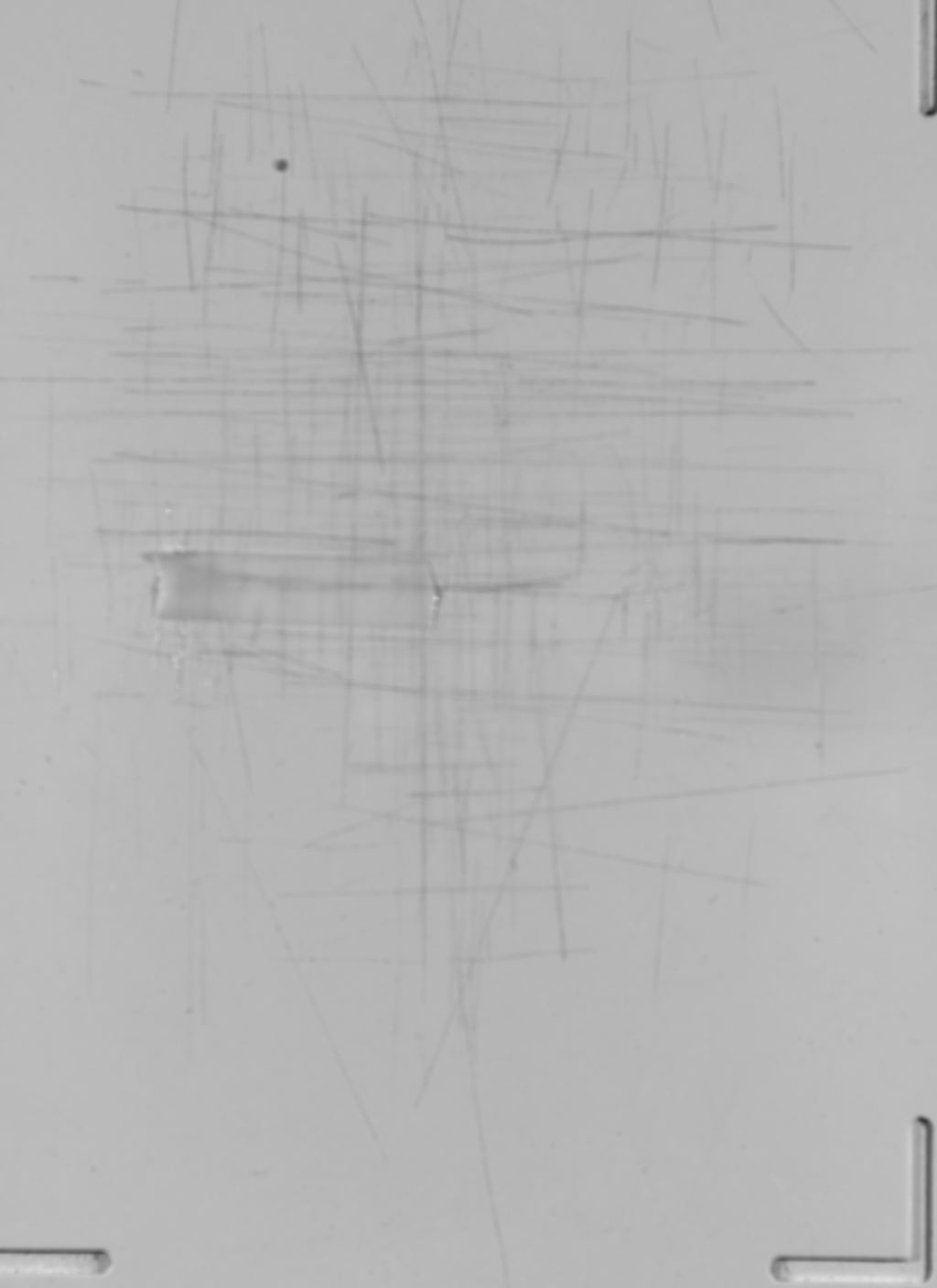

Supplement: Supplemental Information 7 [file peerj-11-15041-s007.zip › Osteoclast-related-genes-raw data3/TRAP/TRAP-2/TRAP-2-4.tif]

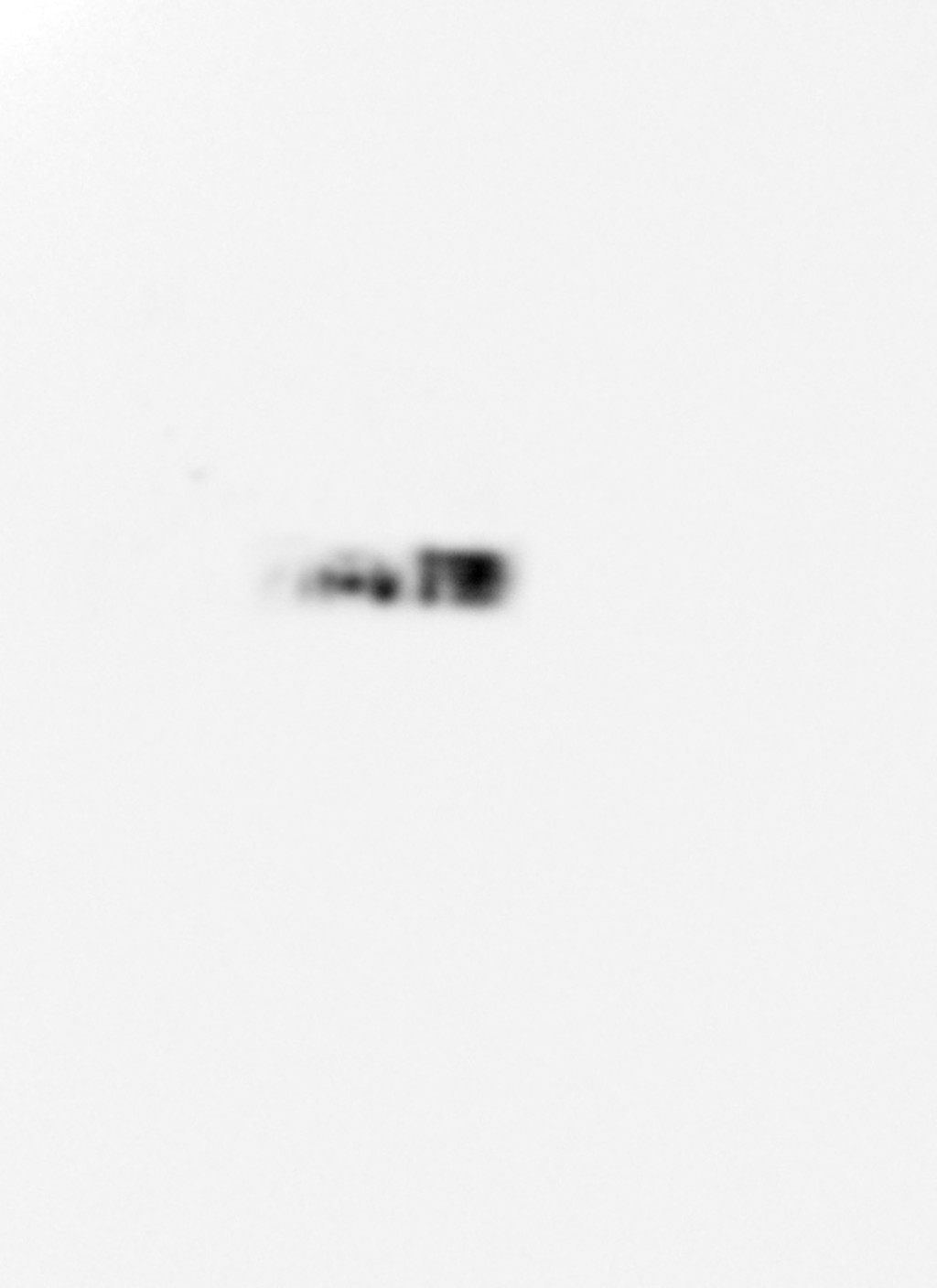

Supplement: Supplemental Information 7 [file peerj-11-15041-s007.zip › Osteoclast-related-genes-raw data3/TRAP/TRAP-3/TRAP-3-1.tif]

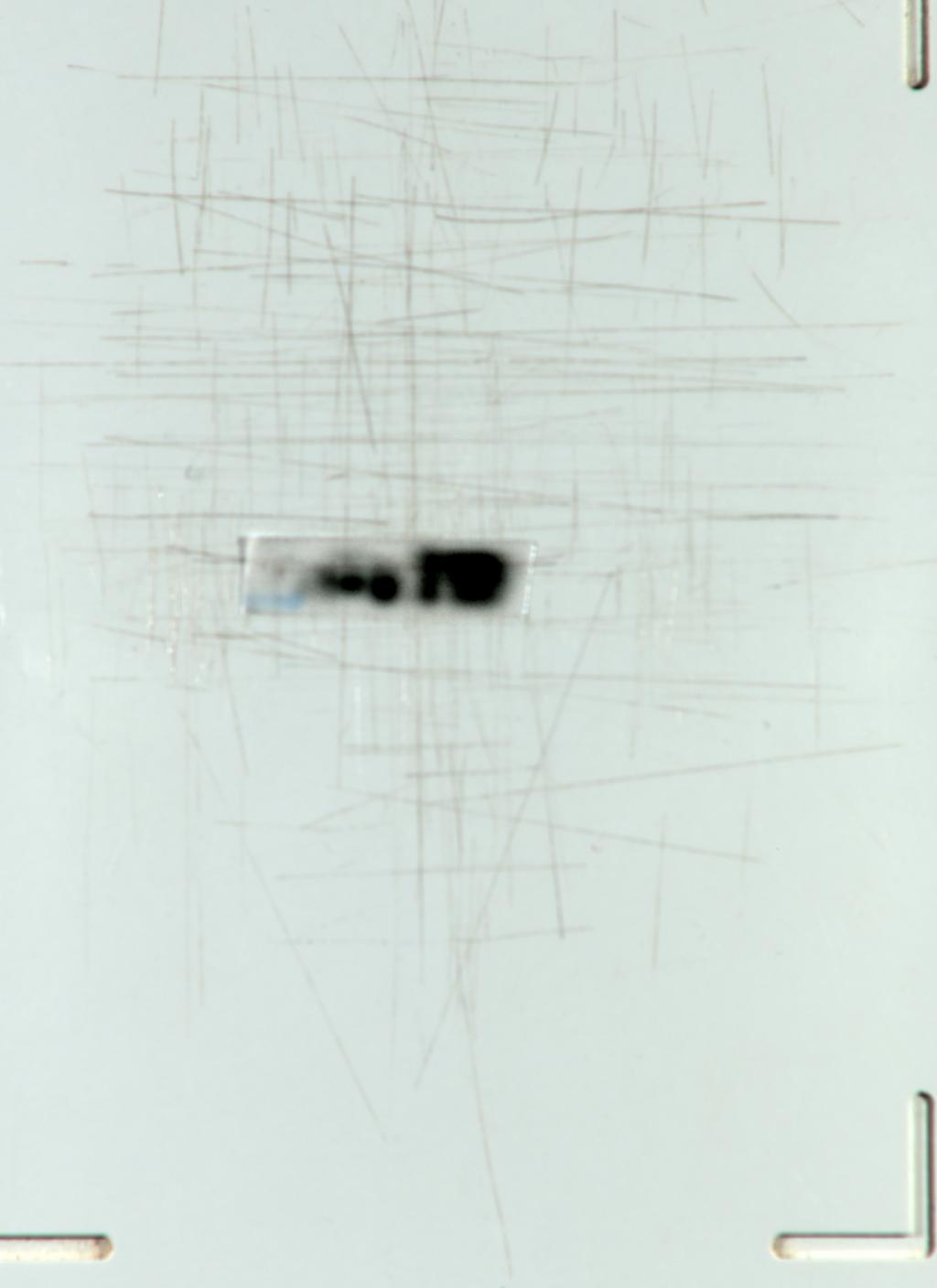

Supplement: Supplemental Information 7 [file peerj-11-15041-s007.zip › Osteoclast-related-genes-raw data3/TRAP/TRAP-3/TRAP-3-2.jpg]

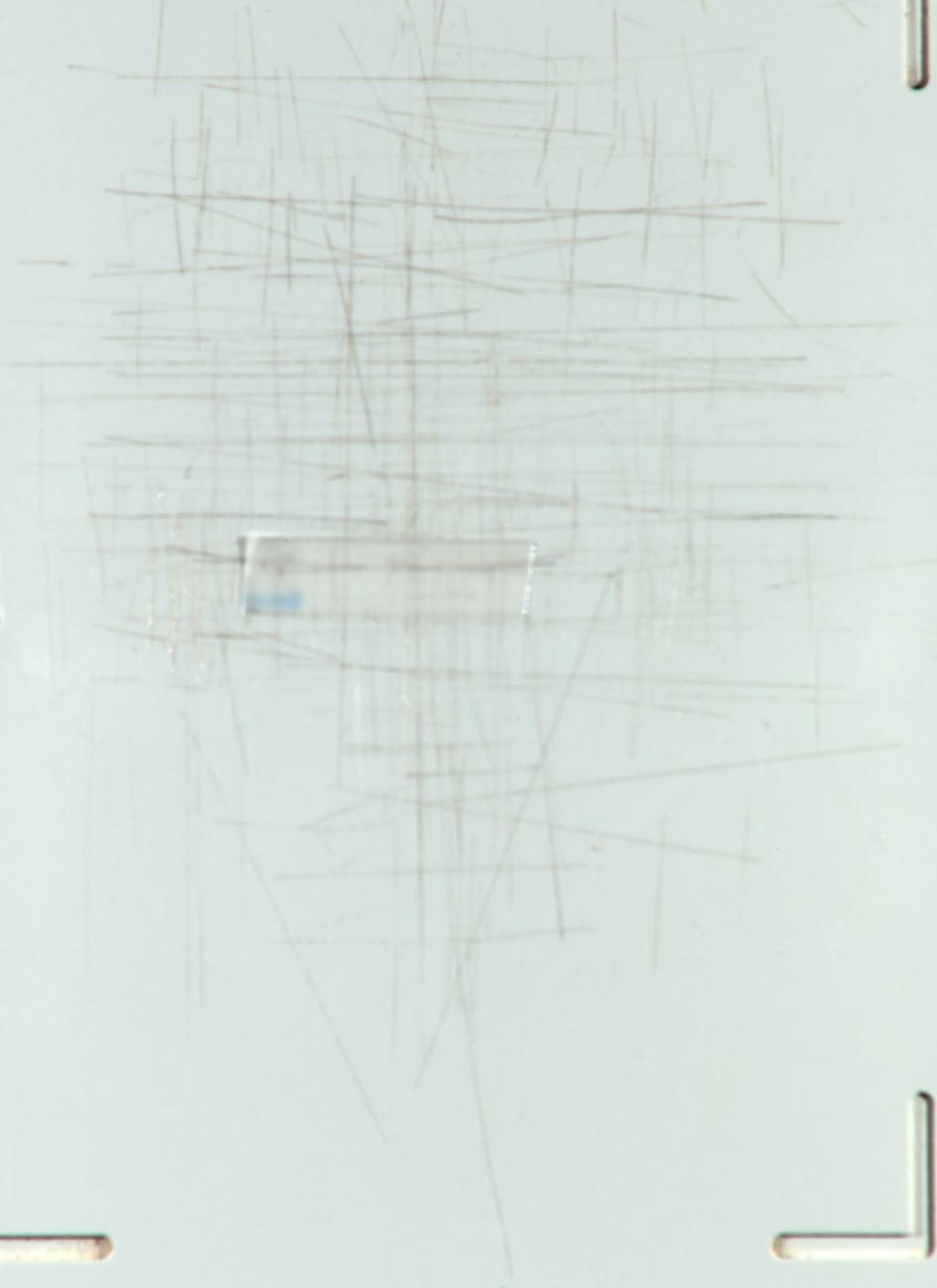

Supplement: Supplemental Information 7 [file peerj-11-15041-s007.zip › Osteoclast-related-genes-raw data3/TRAP/TRAP-3/TRAP-3-3.jpg]

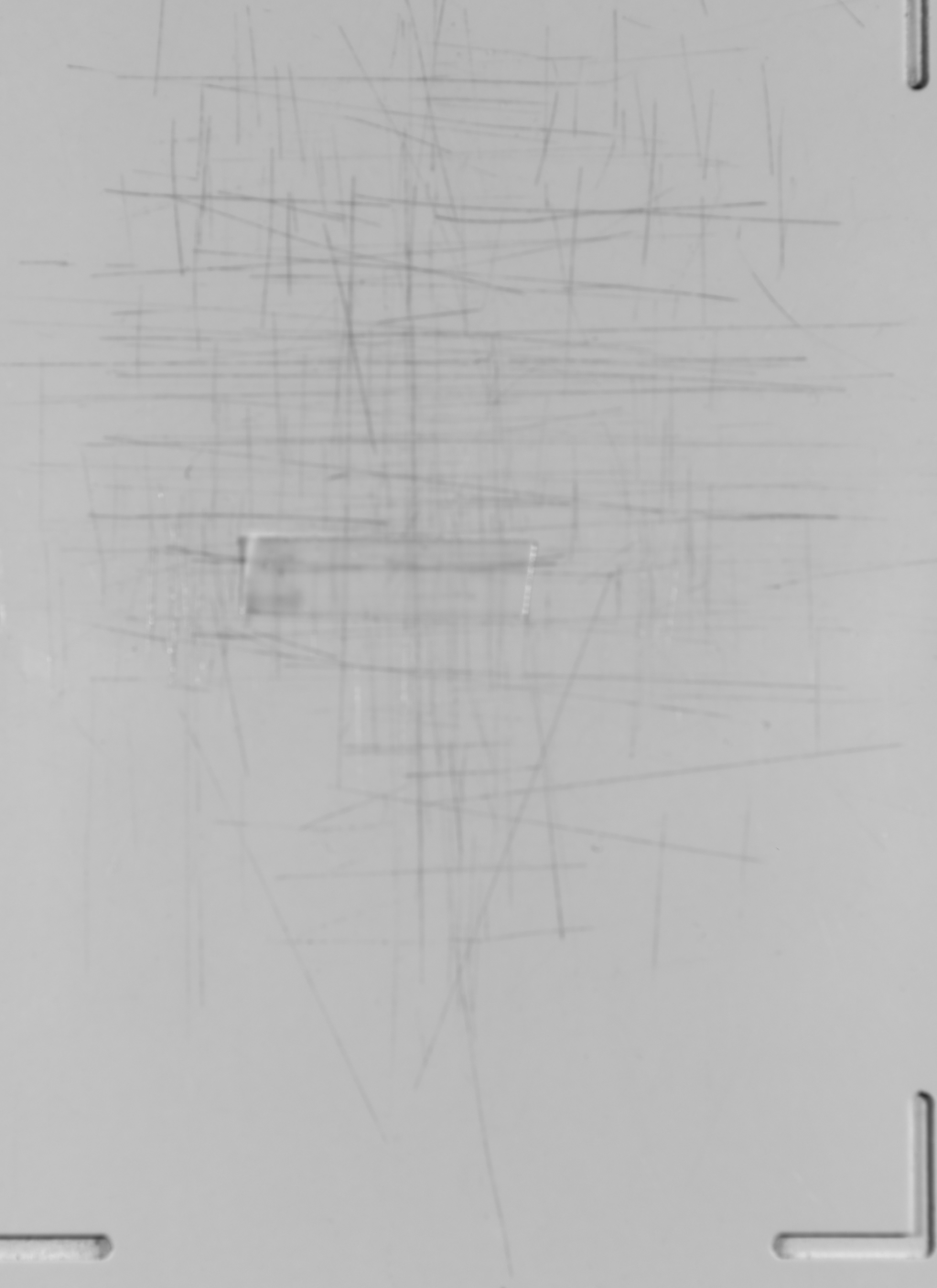

Supplement: Supplemental Information 7 [file peerj-11-15041-s007.zip › Osteoclast-related-genes-raw data3/TRAP/TRAP-3/TRAP-3-4.tif]

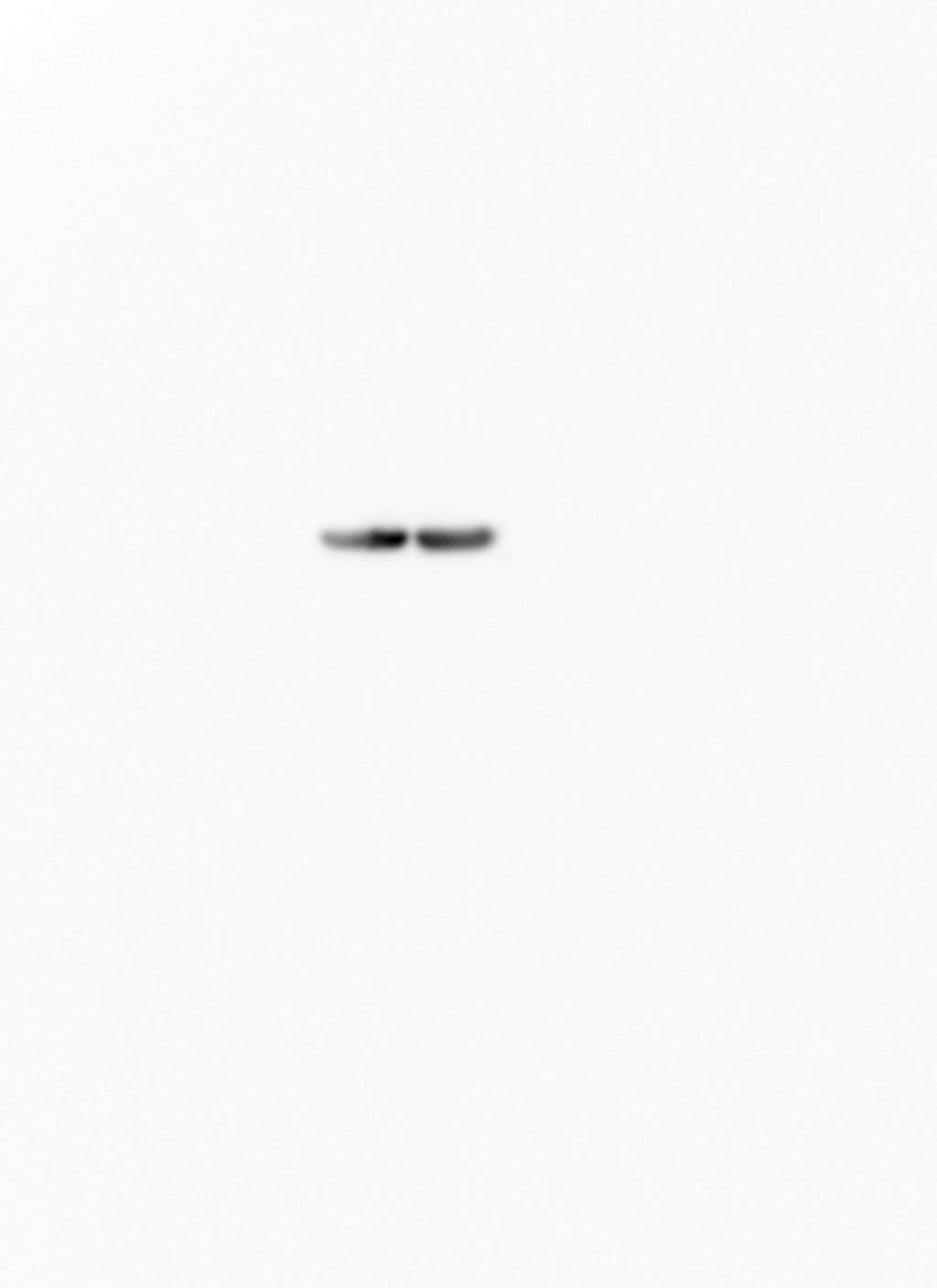

Supplement: Supplemental Information 8 [file peerj-11-15041-s008.zip › Transcriptome-related genes-raw data1/ACTIN/ACTIN-1/ACTIN-1-1.tif]

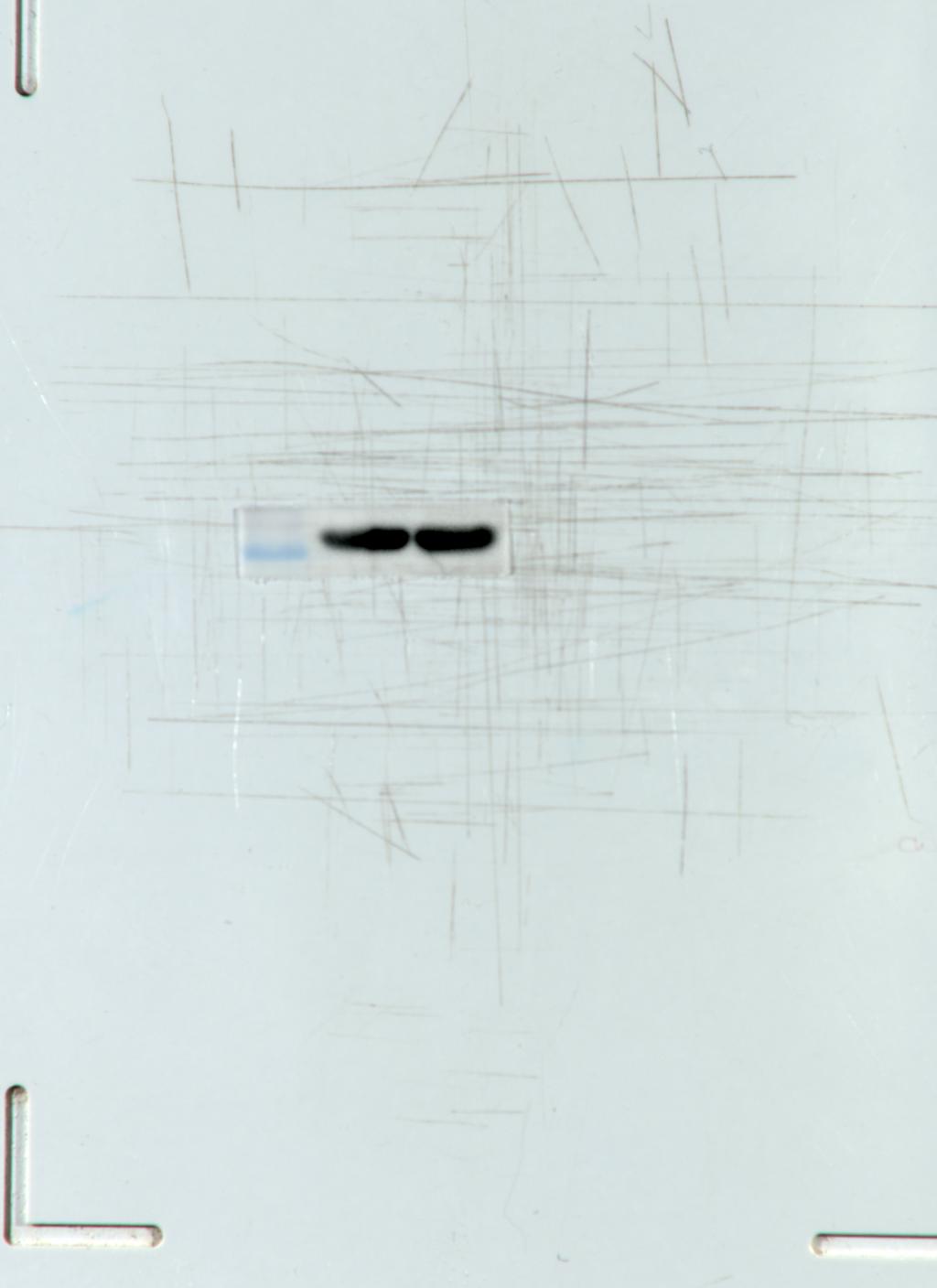

Supplement: Supplemental Information 8 [file peerj-11-15041-s008.zip › Transcriptome-related genes-raw data1/ACTIN/ACTIN-1/ACTIN-1-2.jpg]

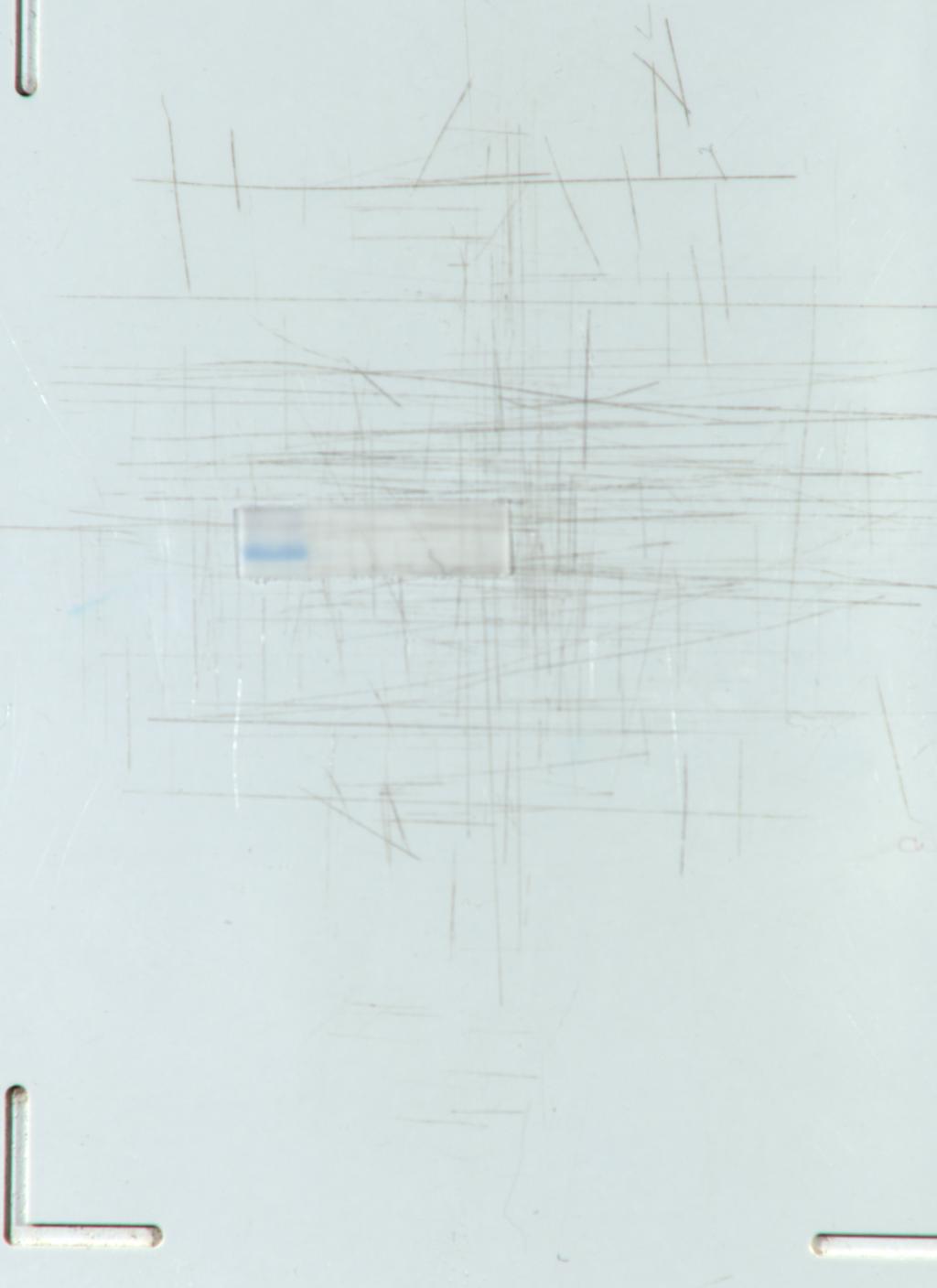

Supplement: Supplemental Information 8 [file peerj-11-15041-s008.zip › Transcriptome-related genes-raw data1/ACTIN/ACTIN-1/ACTIN-1-3.jpg]

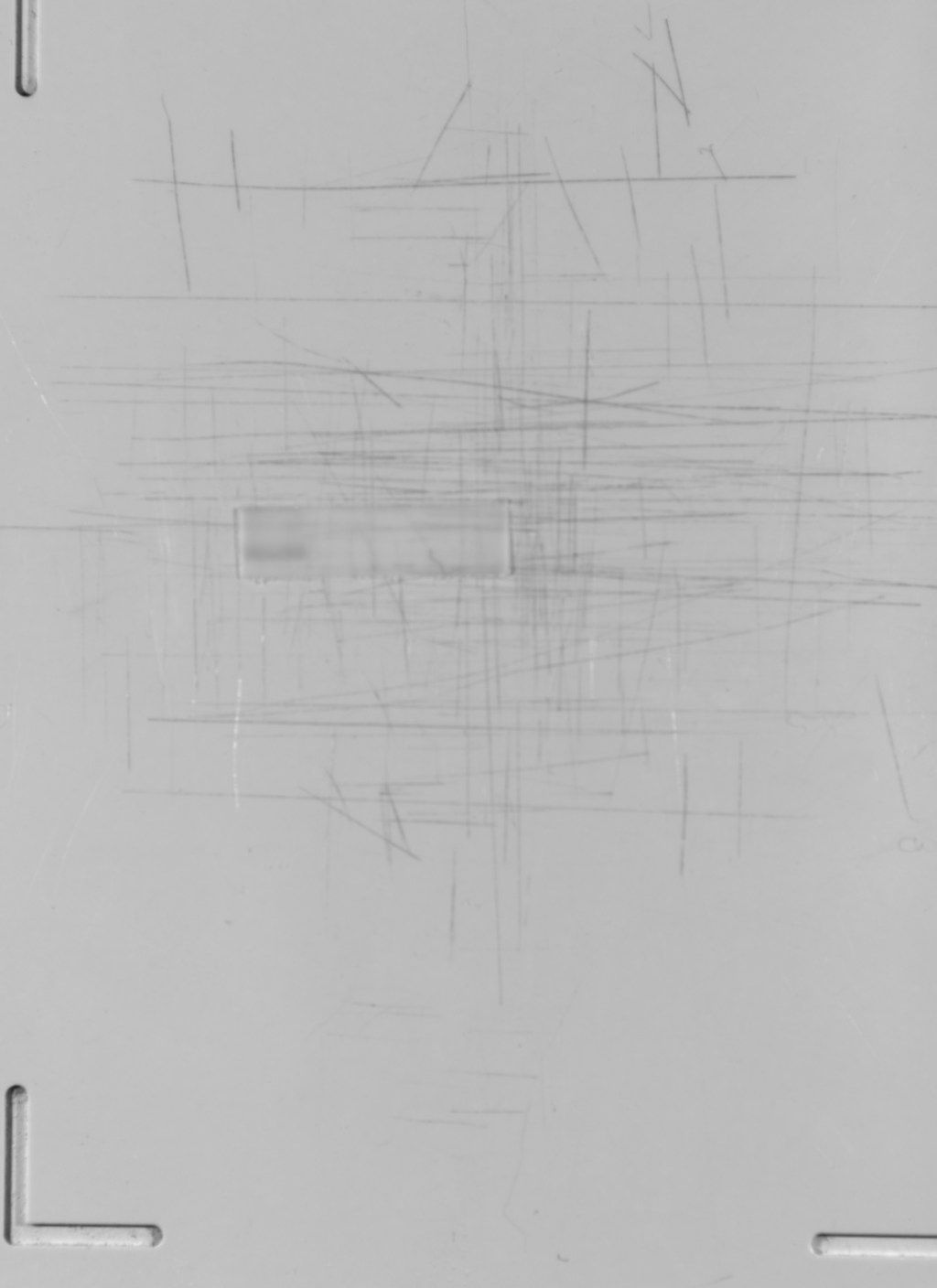

Supplement: Supplemental Information 8 [file peerj-11-15041-s008.zip › Transcriptome-related genes-raw data1/ACTIN/ACTIN-1/ACTIN-1-4.tif]

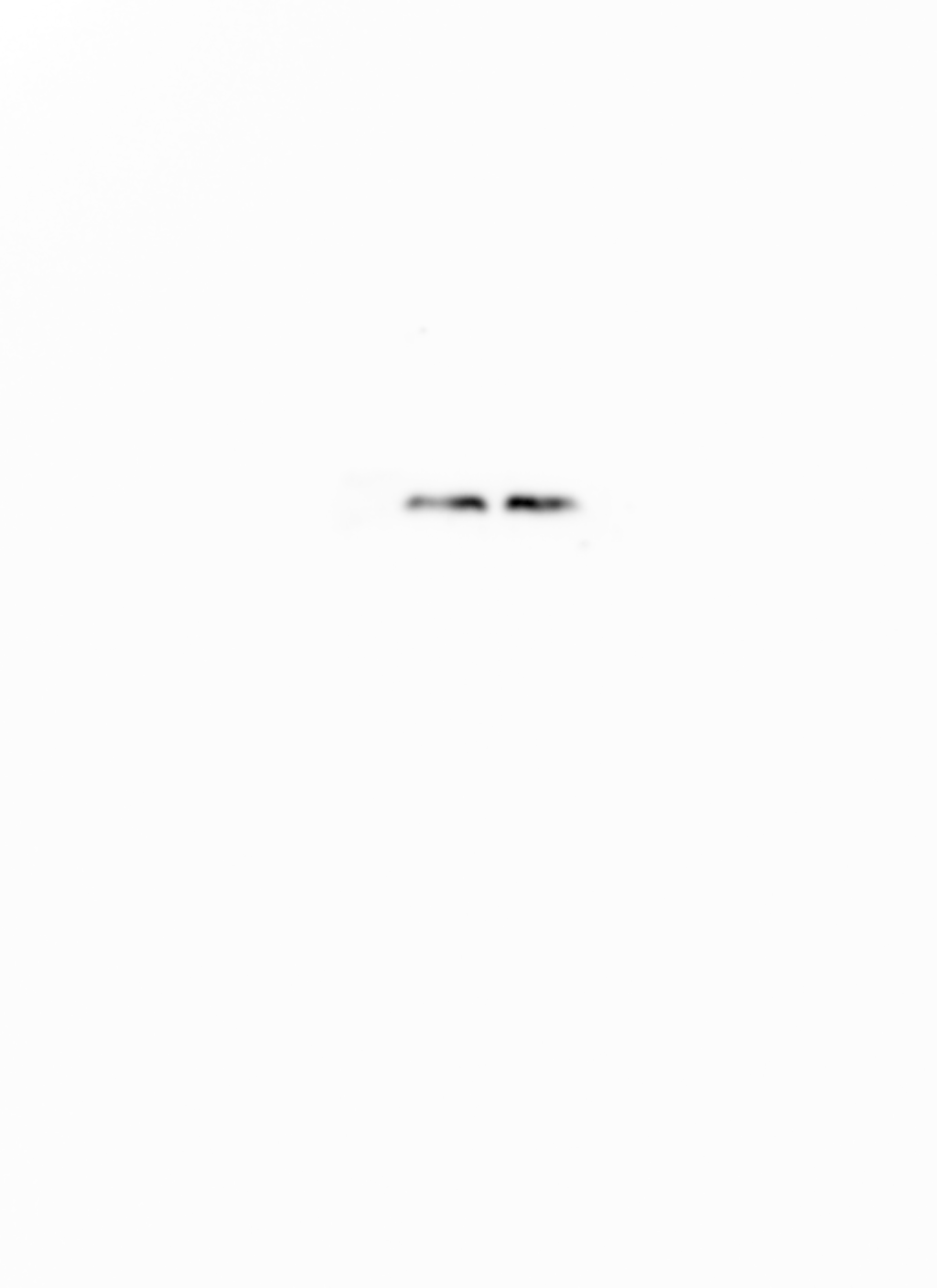

Supplement: Supplemental Information 8 [file peerj-11-15041-s008.zip › Transcriptome-related genes-raw data1/ACTIN/ACTIN-2/ACTIN-2-1.tif]

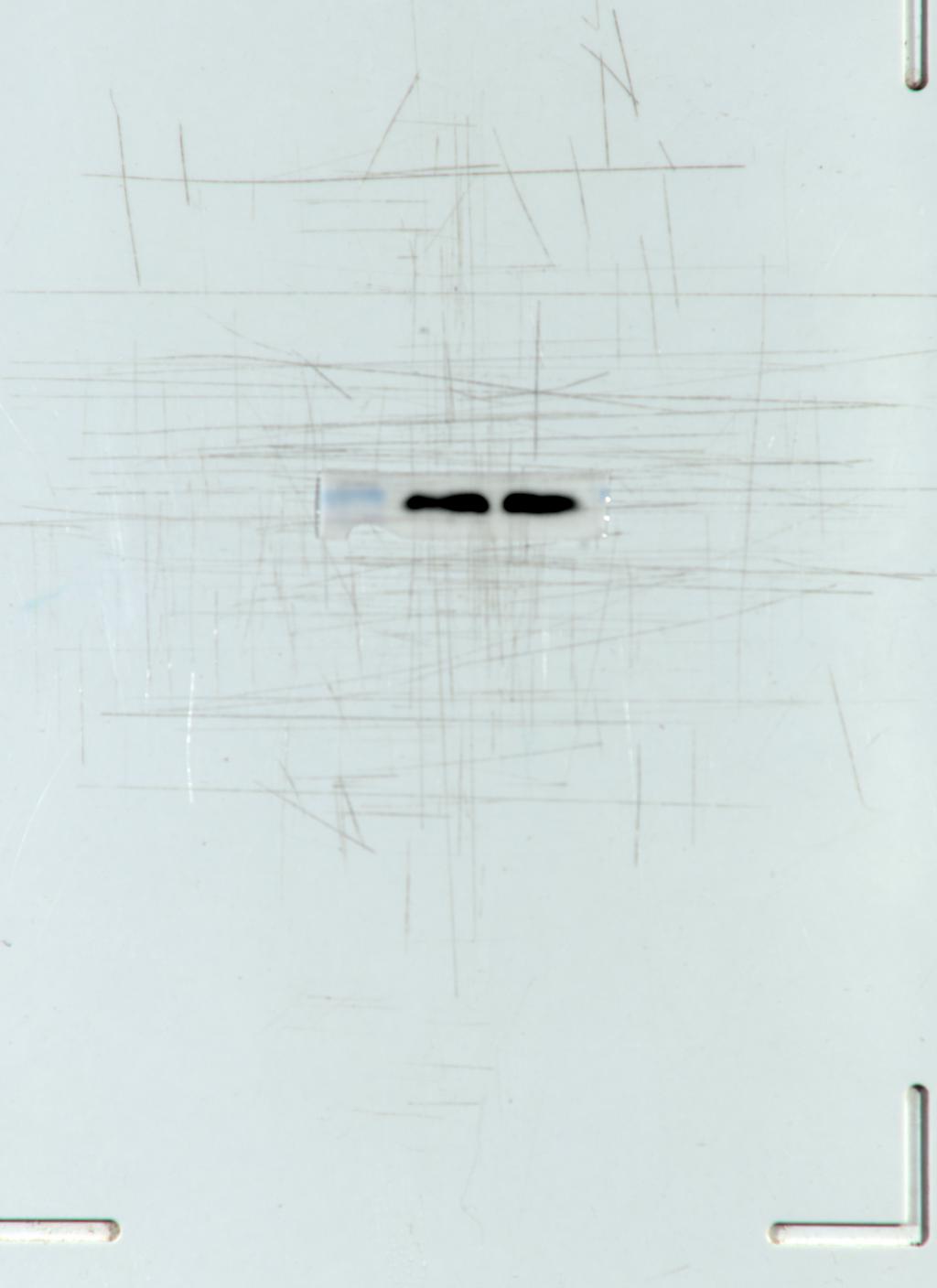

Supplement: Supplemental Information 8 [file peerj-11-15041-s008.zip › Transcriptome-related genes-raw data1/ACTIN/ACTIN-2/ACTIN-2-2.jpg]

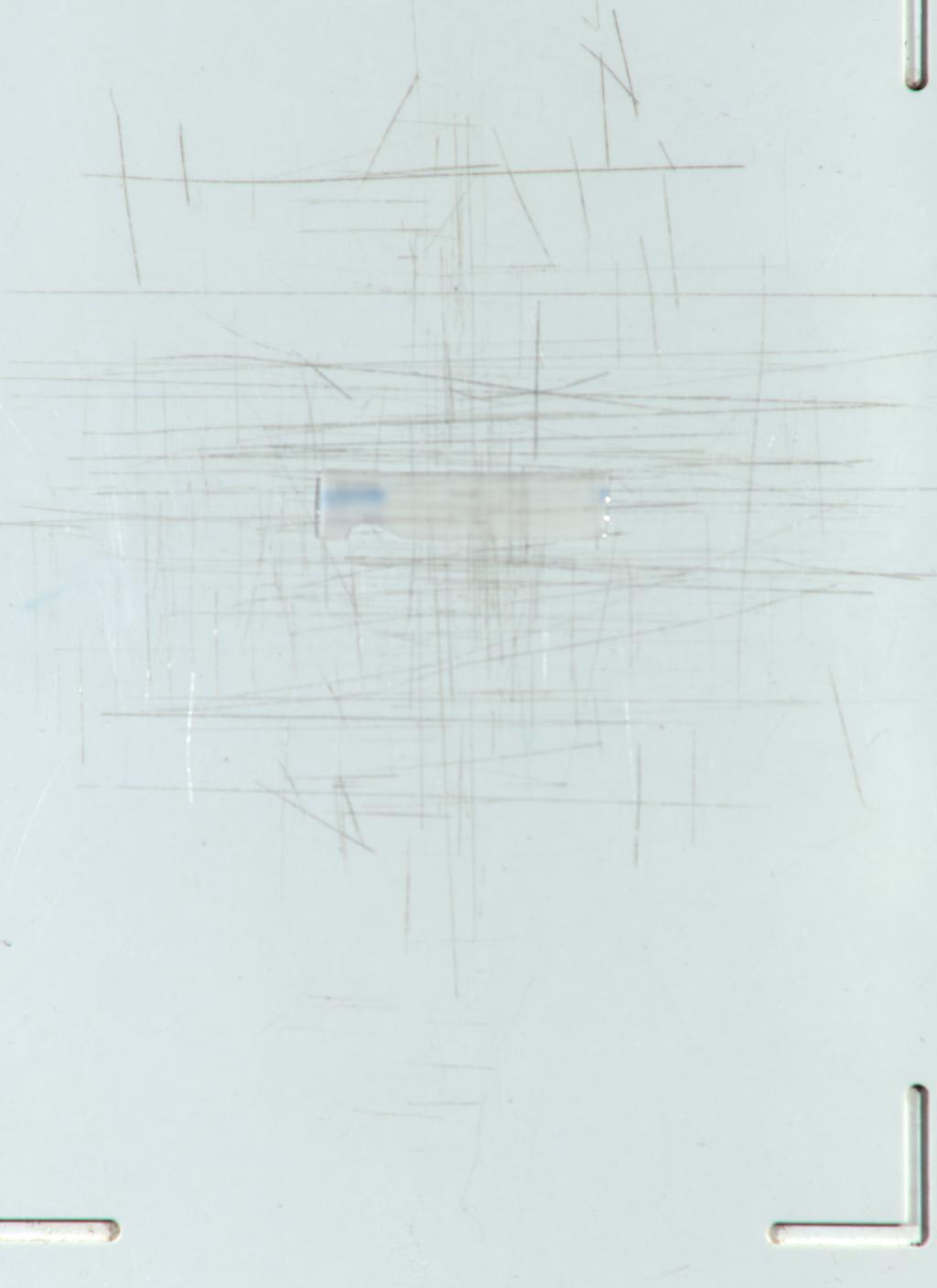

Supplement: Supplemental Information 8 [file peerj-11-15041-s008.zip › Transcriptome-related genes-raw data1/ACTIN/ACTIN-2/ACTIN-2-3.jpg]

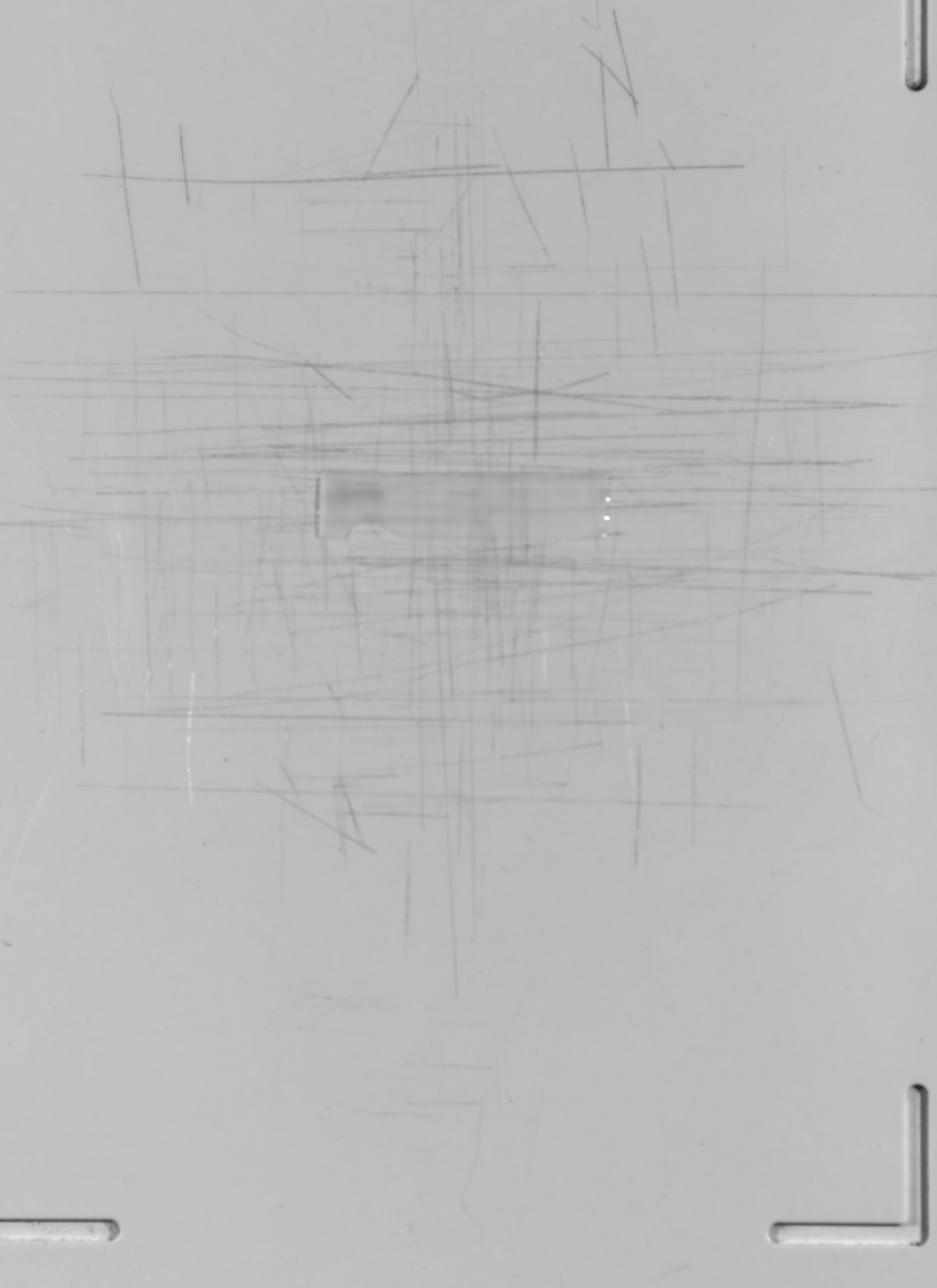

Supplement: Supplemental Information 8 [file peerj-11-15041-s008.zip › Transcriptome-related genes-raw data1/ACTIN/ACTIN-2/ACTIN-2-4.tif]

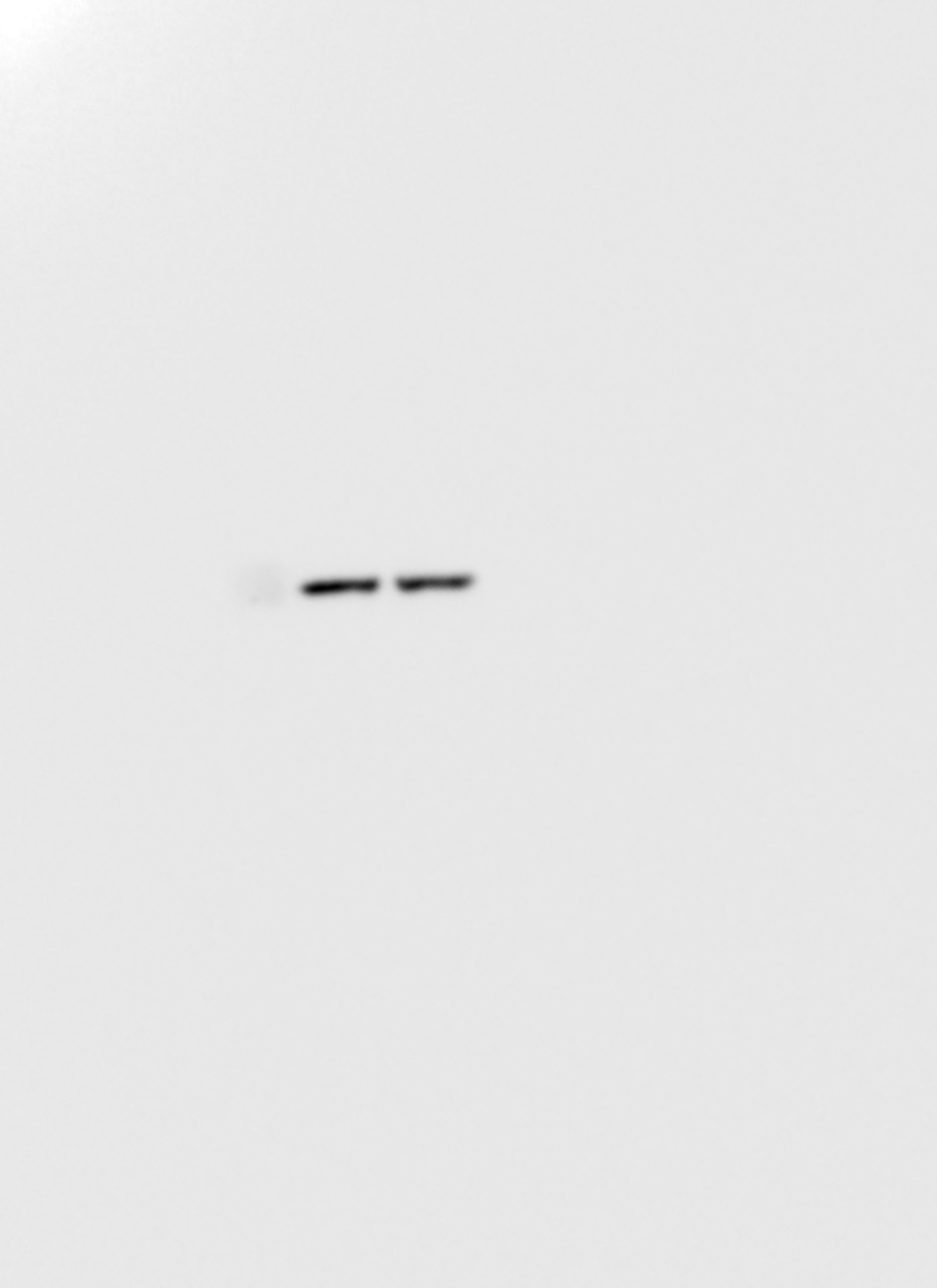

Supplement: Supplemental Information 8 [file peerj-11-15041-s008.zip › Transcriptome-related genes-raw data1/ACTIN/ACTIN-3/ACTIN-3-1.tif]

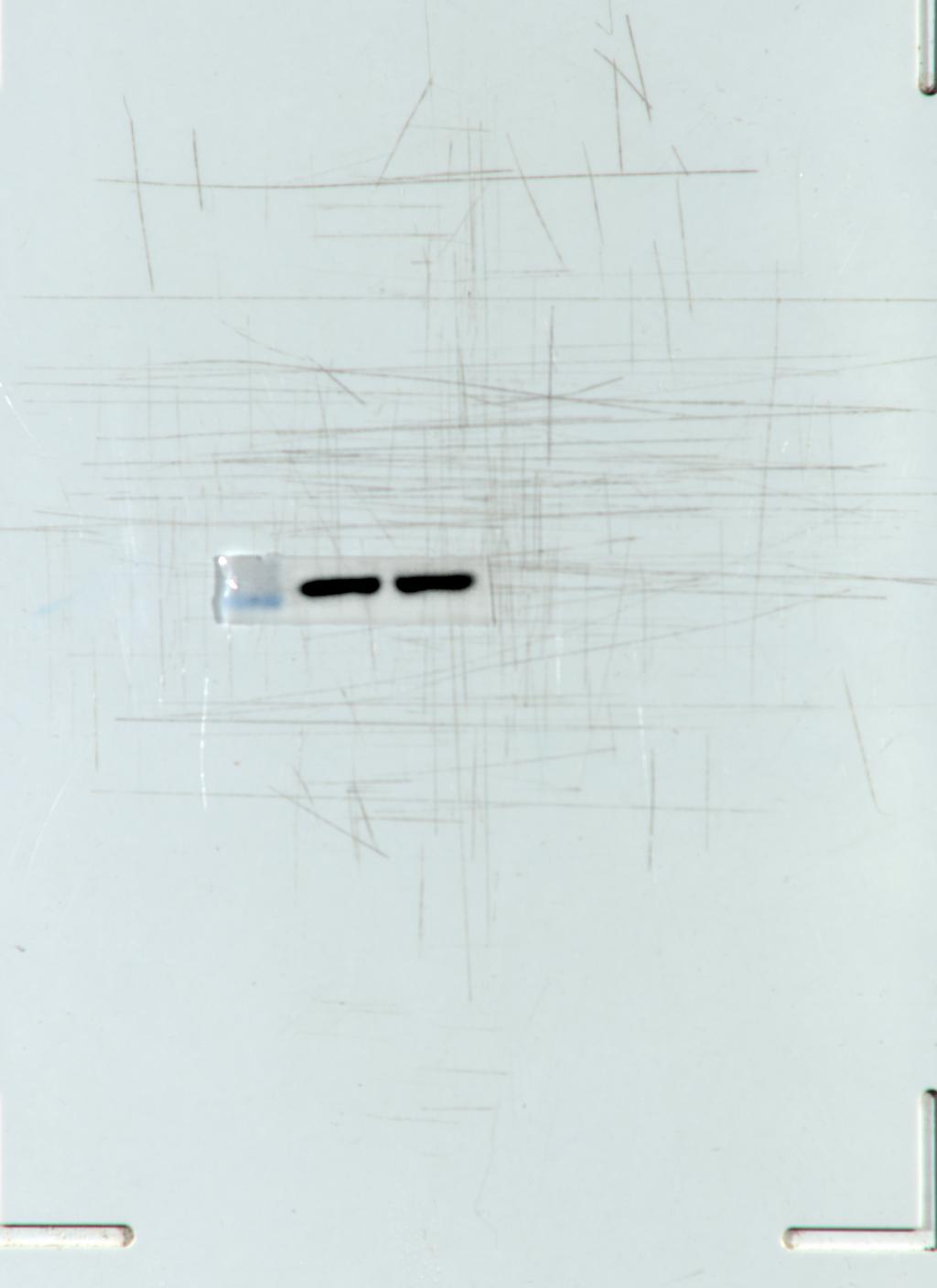

Supplement: Supplemental Information 8 [file peerj-11-15041-s008.zip › Transcriptome-related genes-raw data1/ACTIN/ACTIN-3/ACTIN-3-2.jpg]

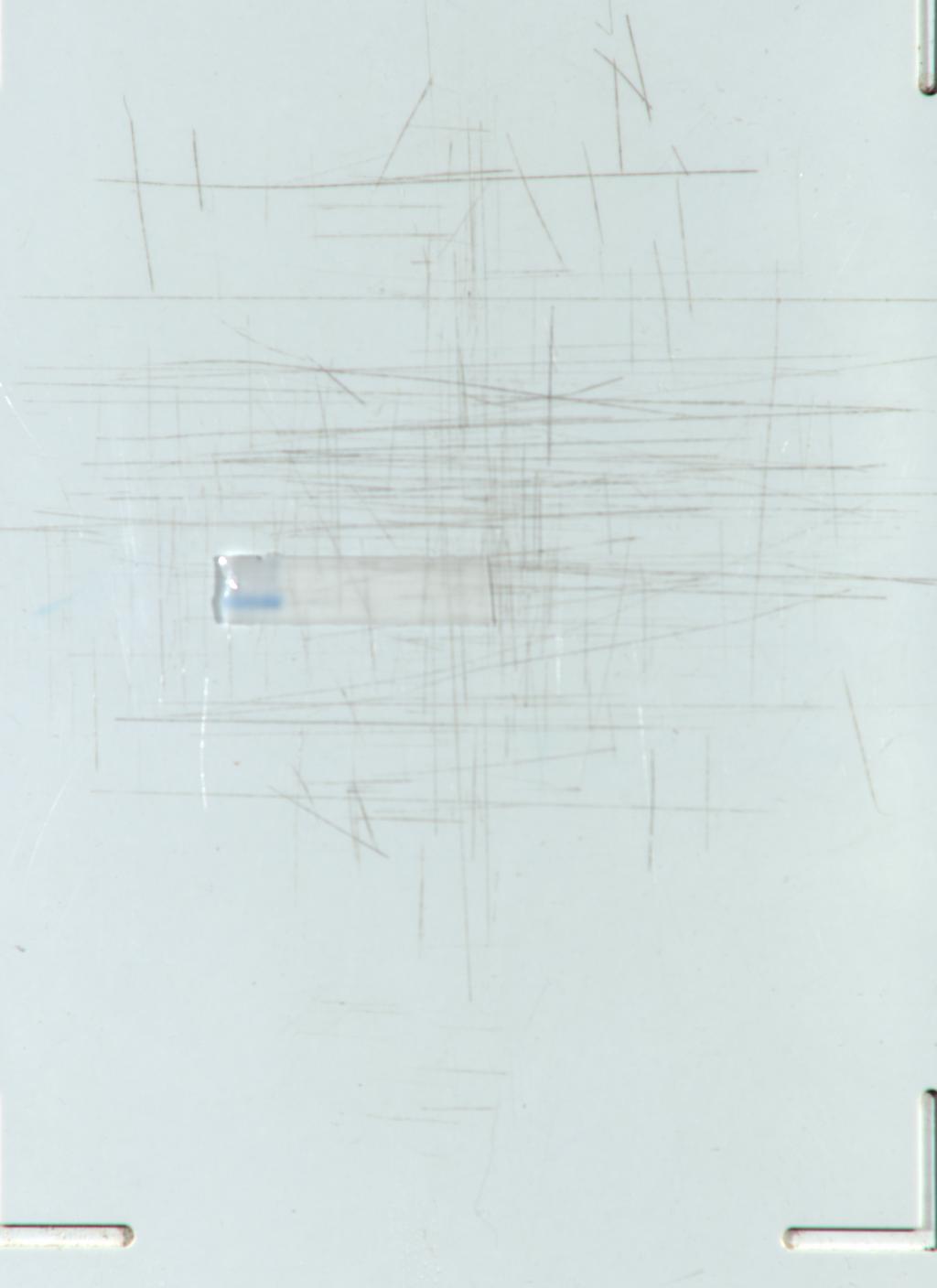

Supplement: Supplemental Information 8 [file peerj-11-15041-s008.zip › Transcriptome-related genes-raw data1/ACTIN/ACTIN-3/ACTIN-3-3.jpg]

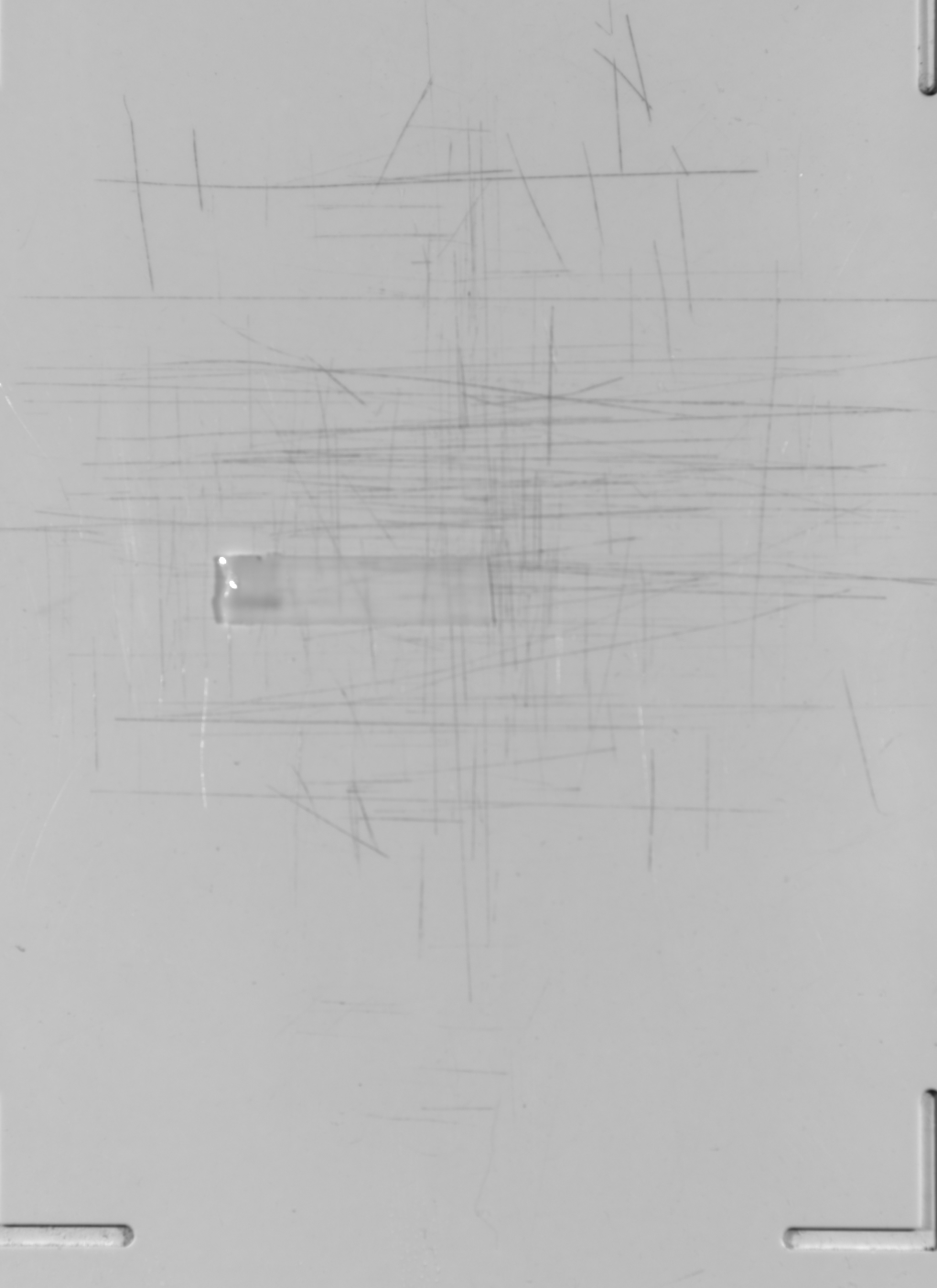

Supplement: Supplemental Information 8 [file peerj-11-15041-s008.zip › Transcriptome-related genes-raw data1/ACTIN/ACTIN-3/ACTIN-3-4.tif]

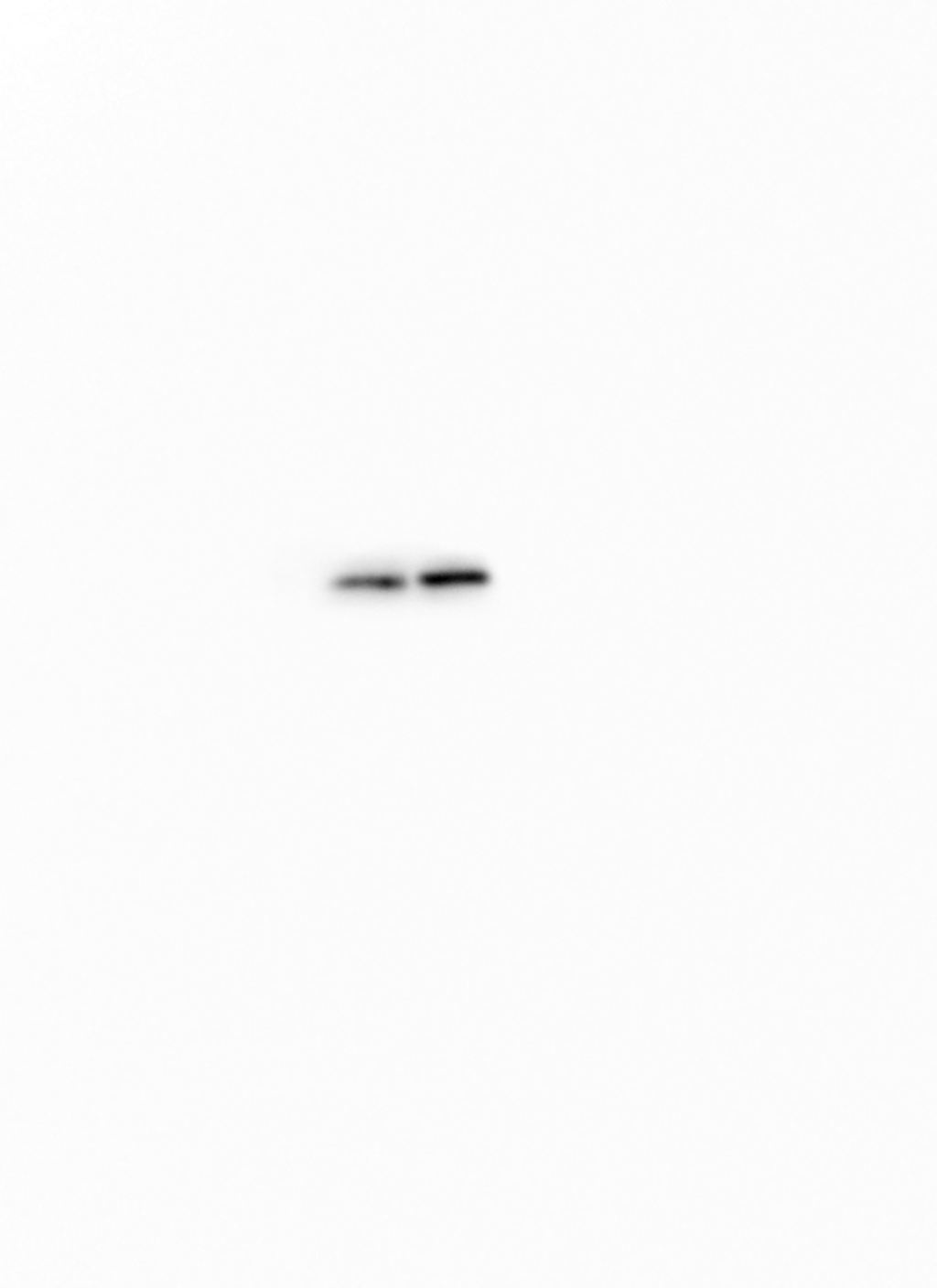

Supplement: Supplemental Information 8 [file peerj-11-15041-s008.zip › Transcriptome-related genes-raw data1/GAPDH/GAPDH-1/GAPDH-1-1.tif]

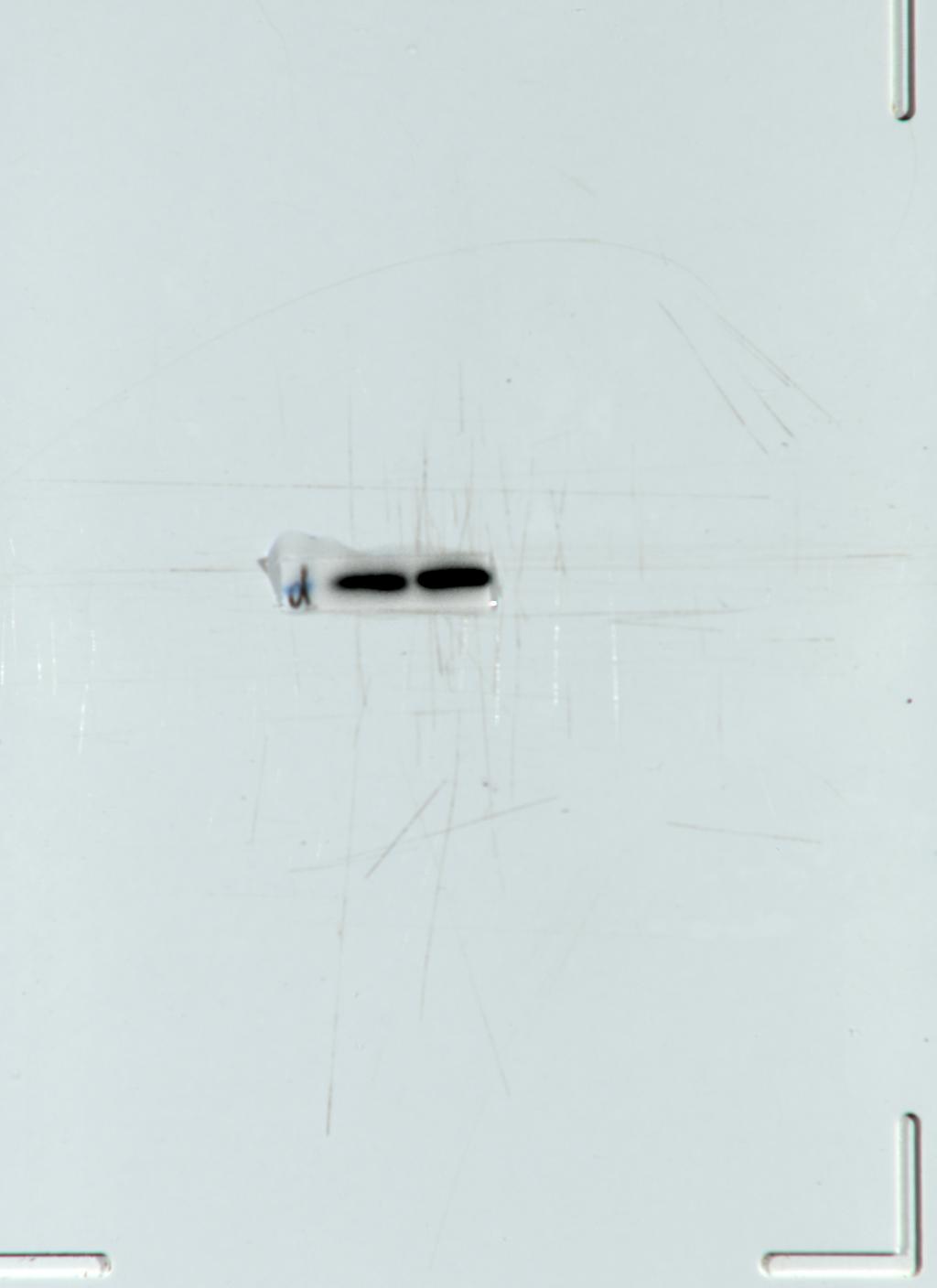

Supplement: Supplemental Information 8 [file peerj-11-15041-s008.zip › Transcriptome-related genes-raw data1/GAPDH/GAPDH-1/GAPDH-1-2.jpg]

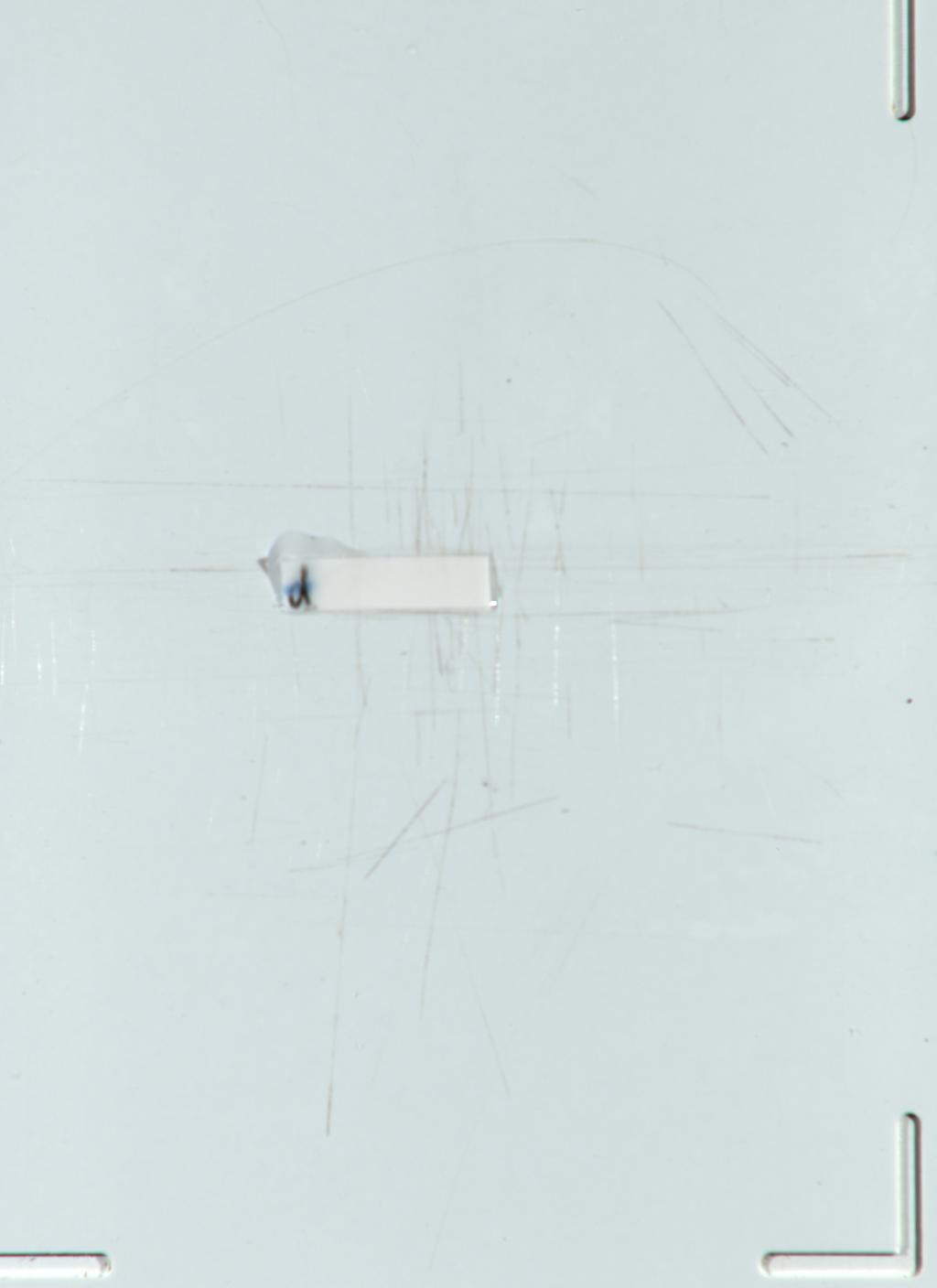

Supplement: Supplemental Information 8 [file peerj-11-15041-s008.zip › Transcriptome-related genes-raw data1/GAPDH/GAPDH-1/GAPDH-1-3.jpg]

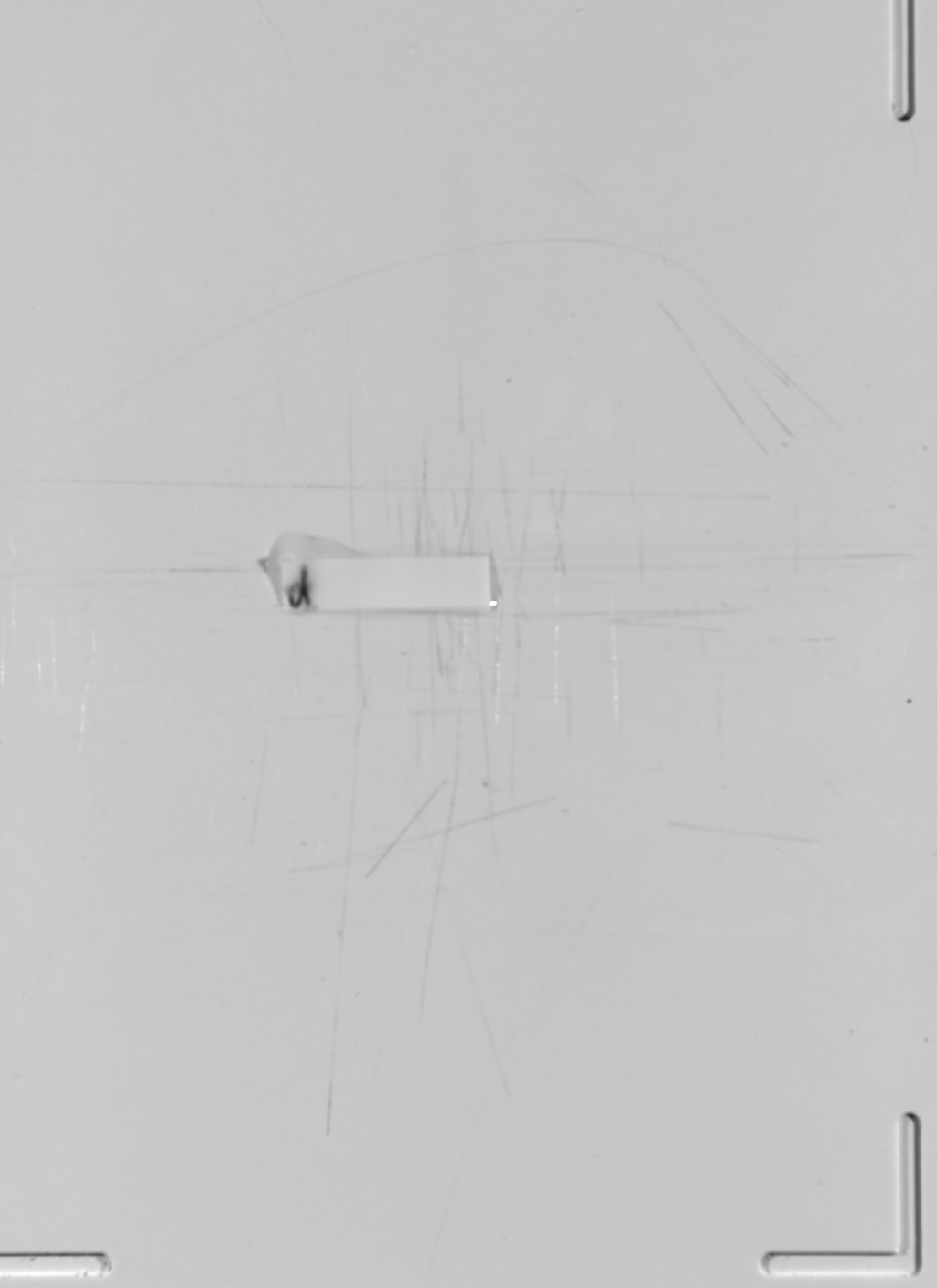

Supplement: Supplemental Information 8 [file peerj-11-15041-s008.zip › Transcriptome-related genes-raw data1/GAPDH/GAPDH-1/GAPDH-1-4.tif]

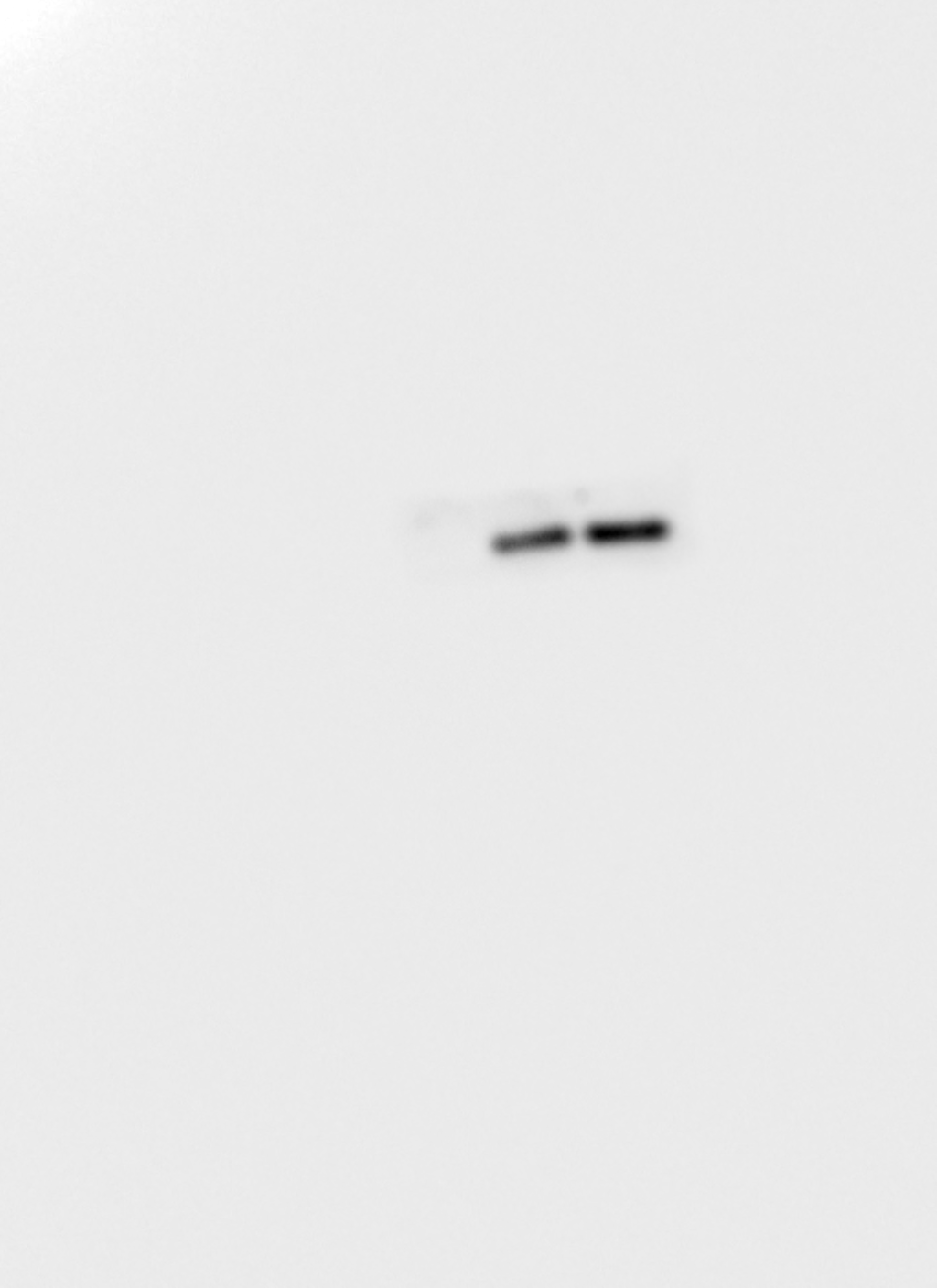

Supplement: Supplemental Information 8 [file peerj-11-15041-s008.zip › Transcriptome-related genes-raw data1/GAPDH/GAPDH-2/GAPDH-2-1.tif]

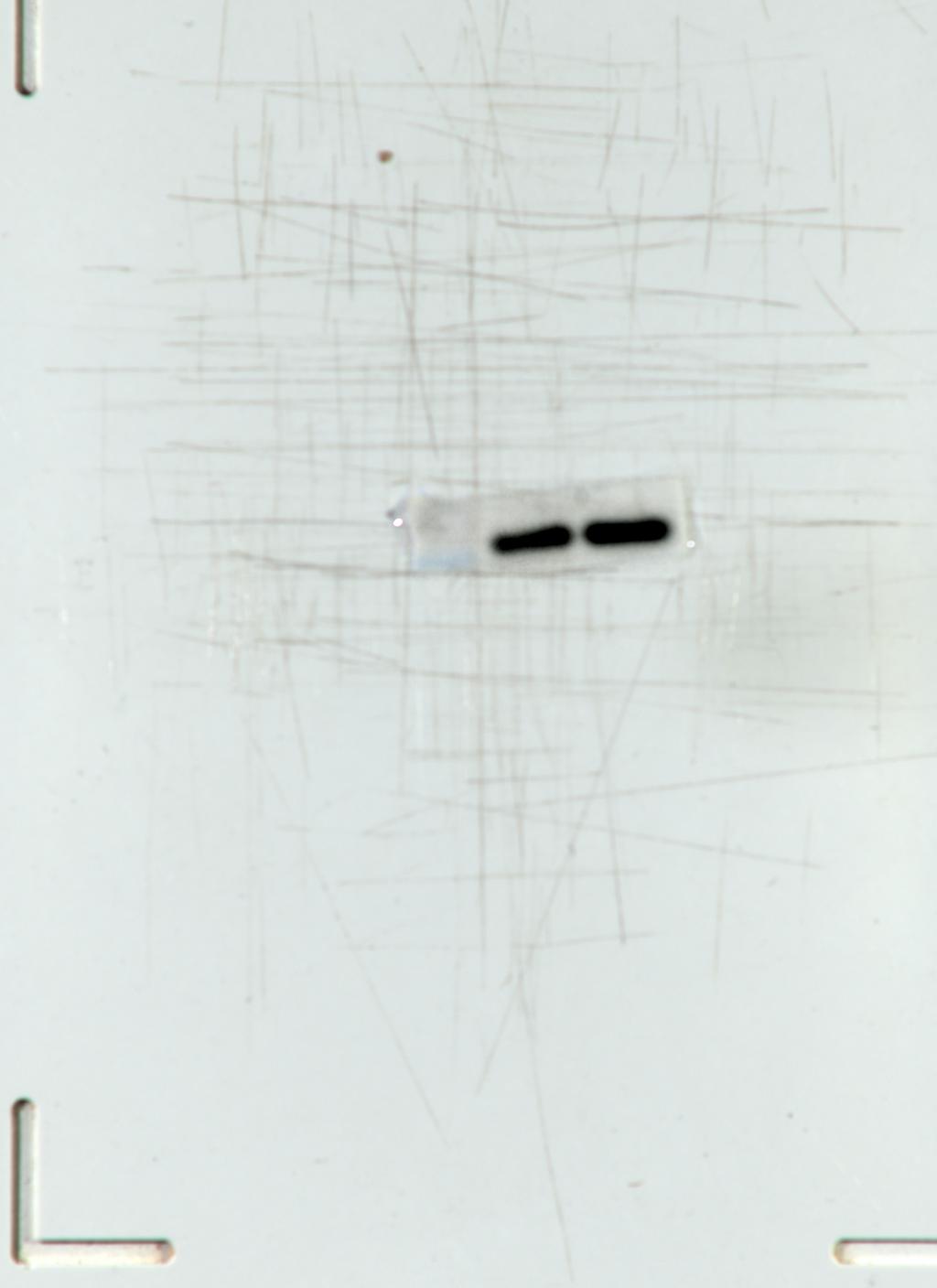

Supplement: Supplemental Information 8 [file peerj-11-15041-s008.zip › Transcriptome-related genes-raw data1/GAPDH/GAPDH-2/GAPDH-2-2.jpg]

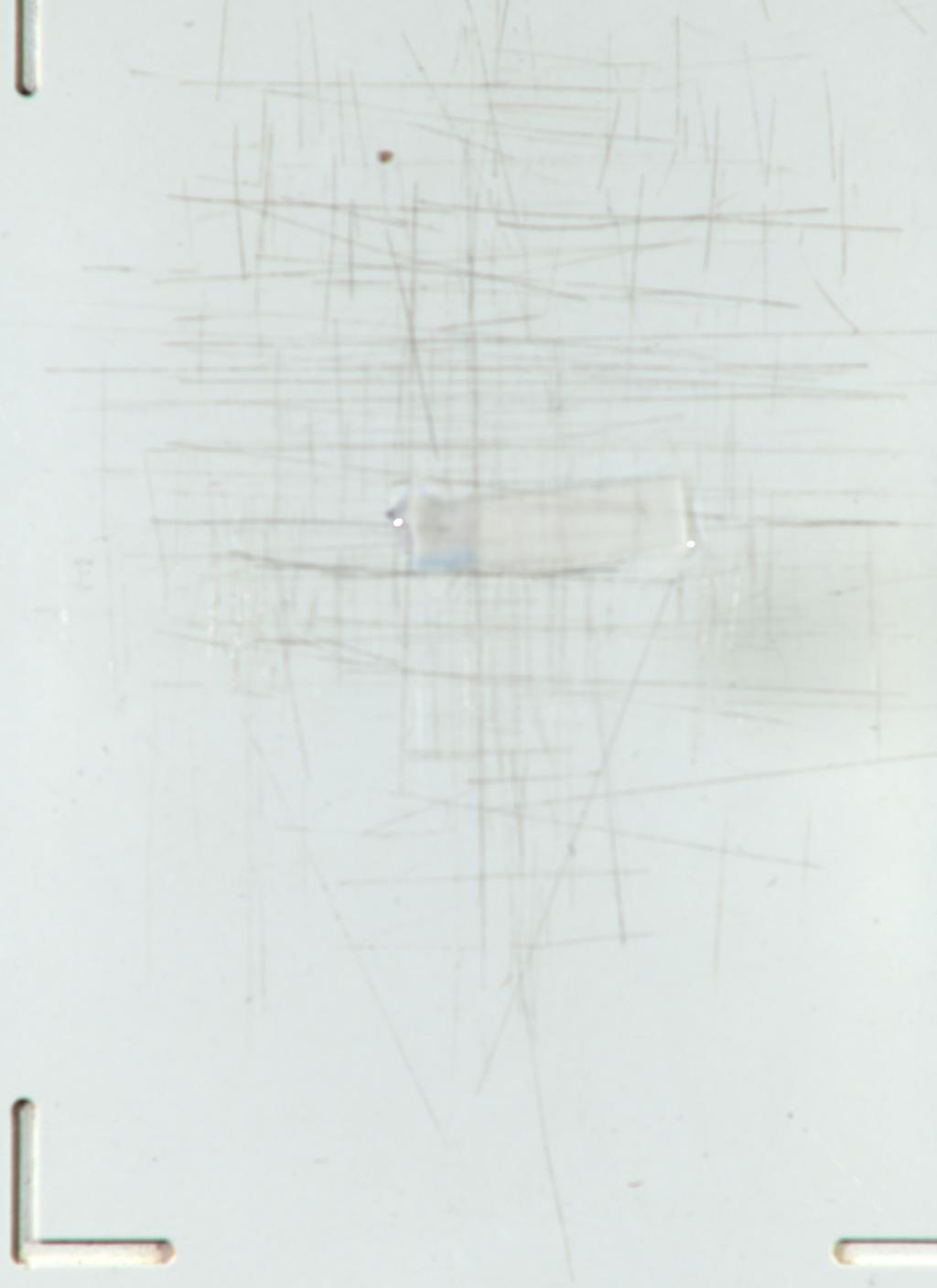

Supplement: Supplemental Information 8 [file peerj-11-15041-s008.zip › Transcriptome-related genes-raw data1/GAPDH/GAPDH-2/GAPDH-2-3.jpg]

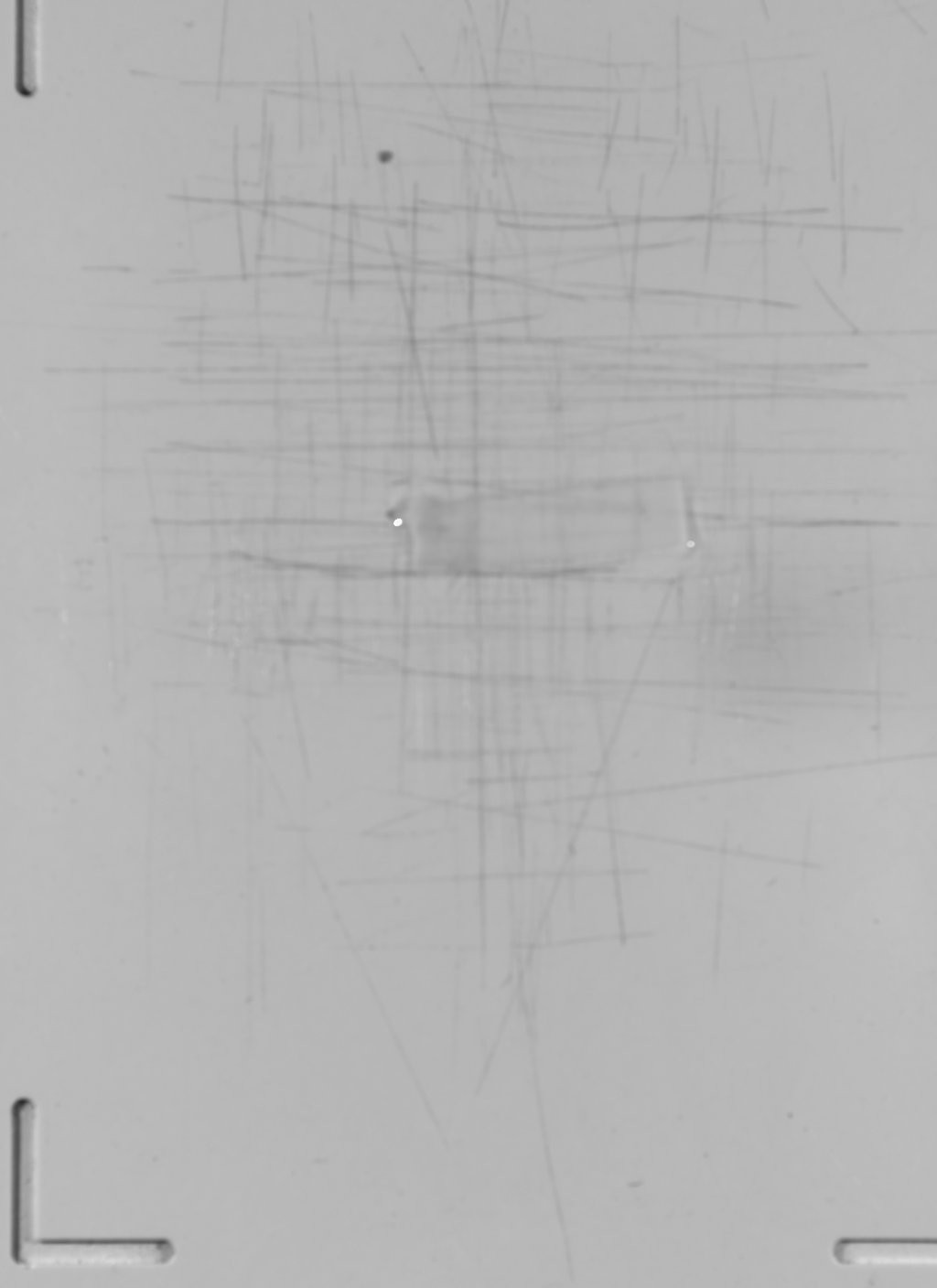

Supplement: Supplemental Information 8 [file peerj-11-15041-s008.zip › Transcriptome-related genes-raw data1/GAPDH/GAPDH-2/GAPDH-2-4.tif]

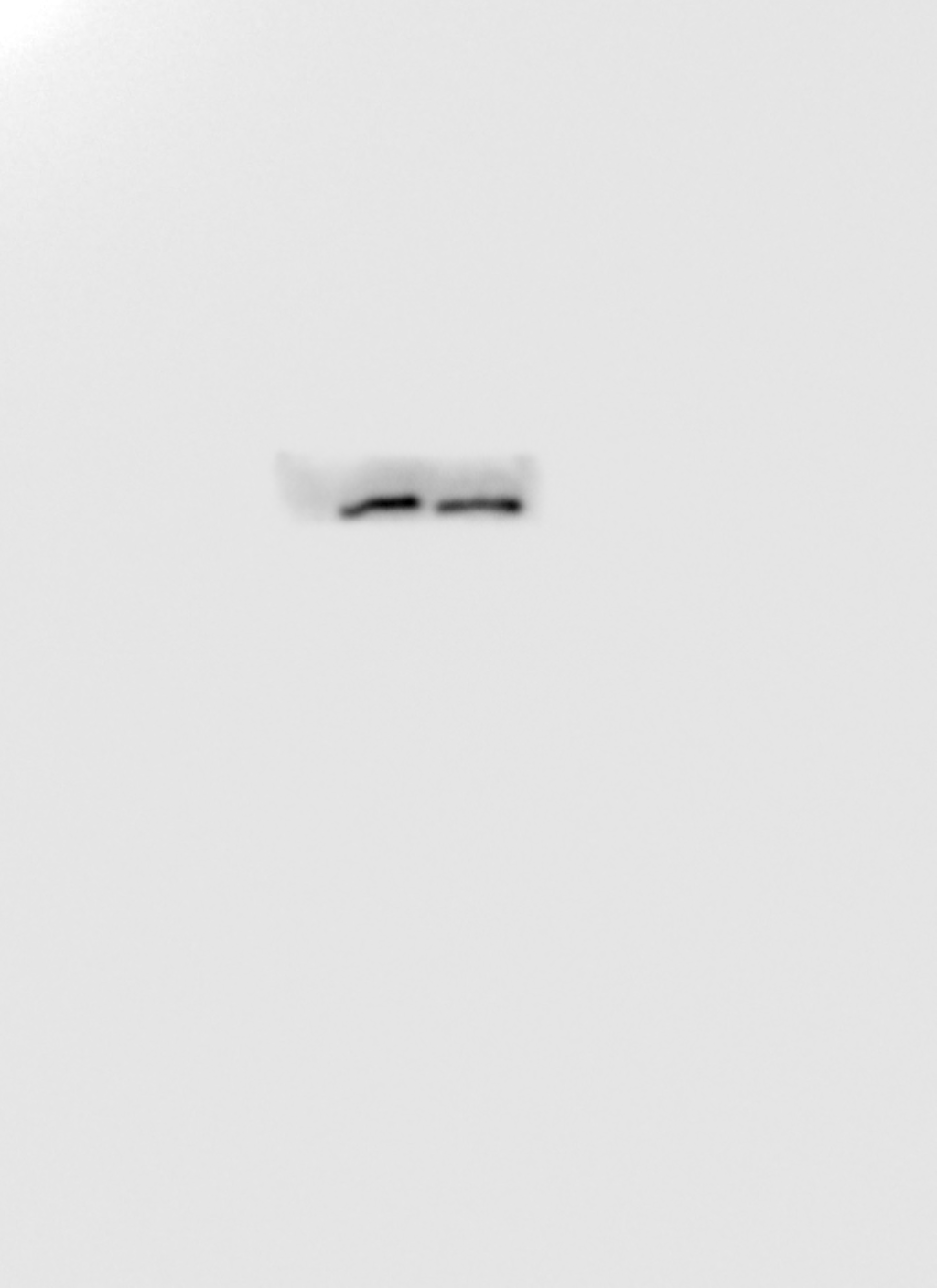

Supplement: Supplemental Information 8 [file peerj-11-15041-s008.zip › Transcriptome-related genes-raw data1/GAPDH/GAPDH-3/GAPDH-3-1.tif]

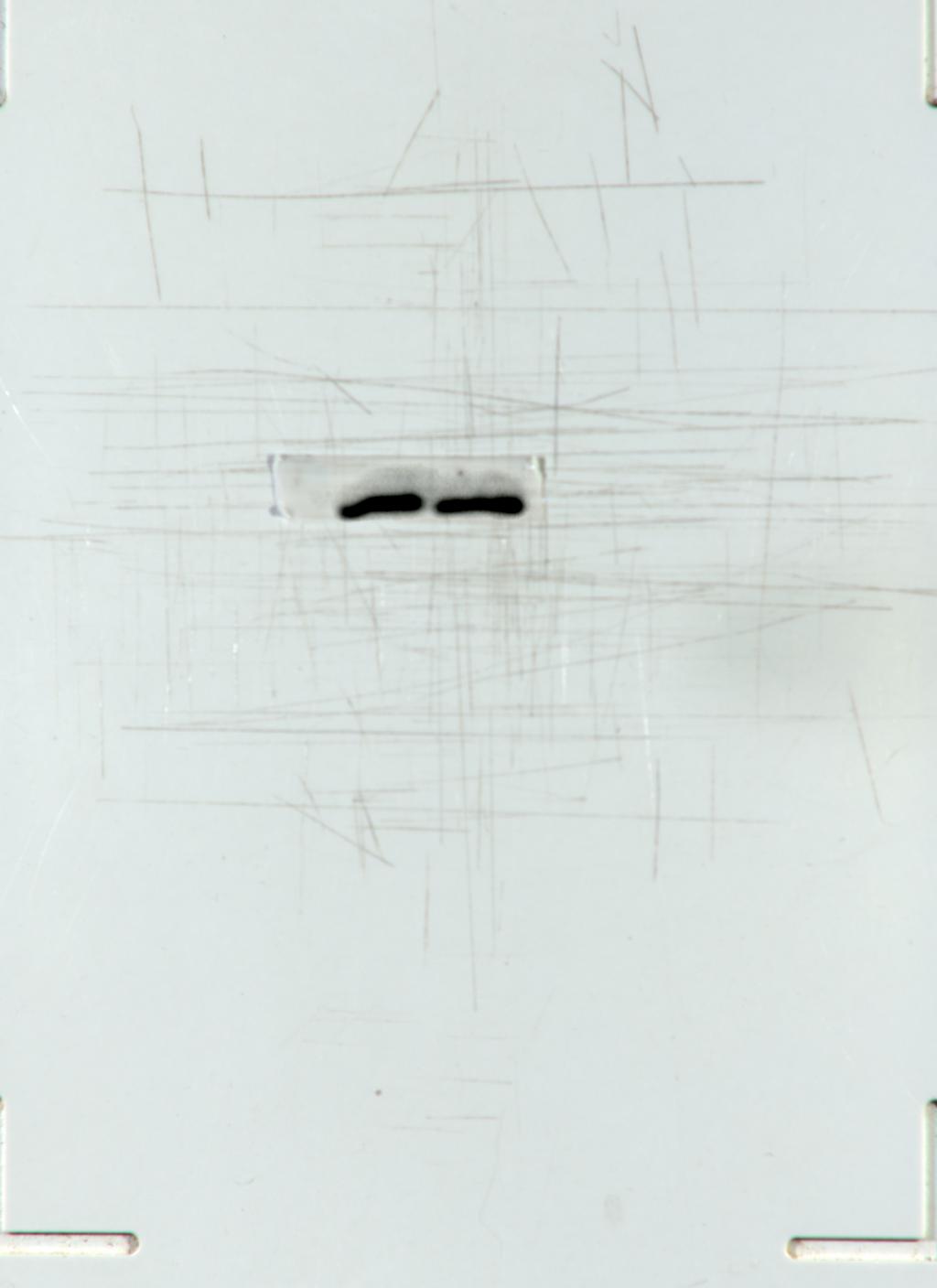

Supplement: Supplemental Information 8 [file peerj-11-15041-s008.zip › Transcriptome-related genes-raw data1/GAPDH/GAPDH-3/GAPDH-3-2.jpg]

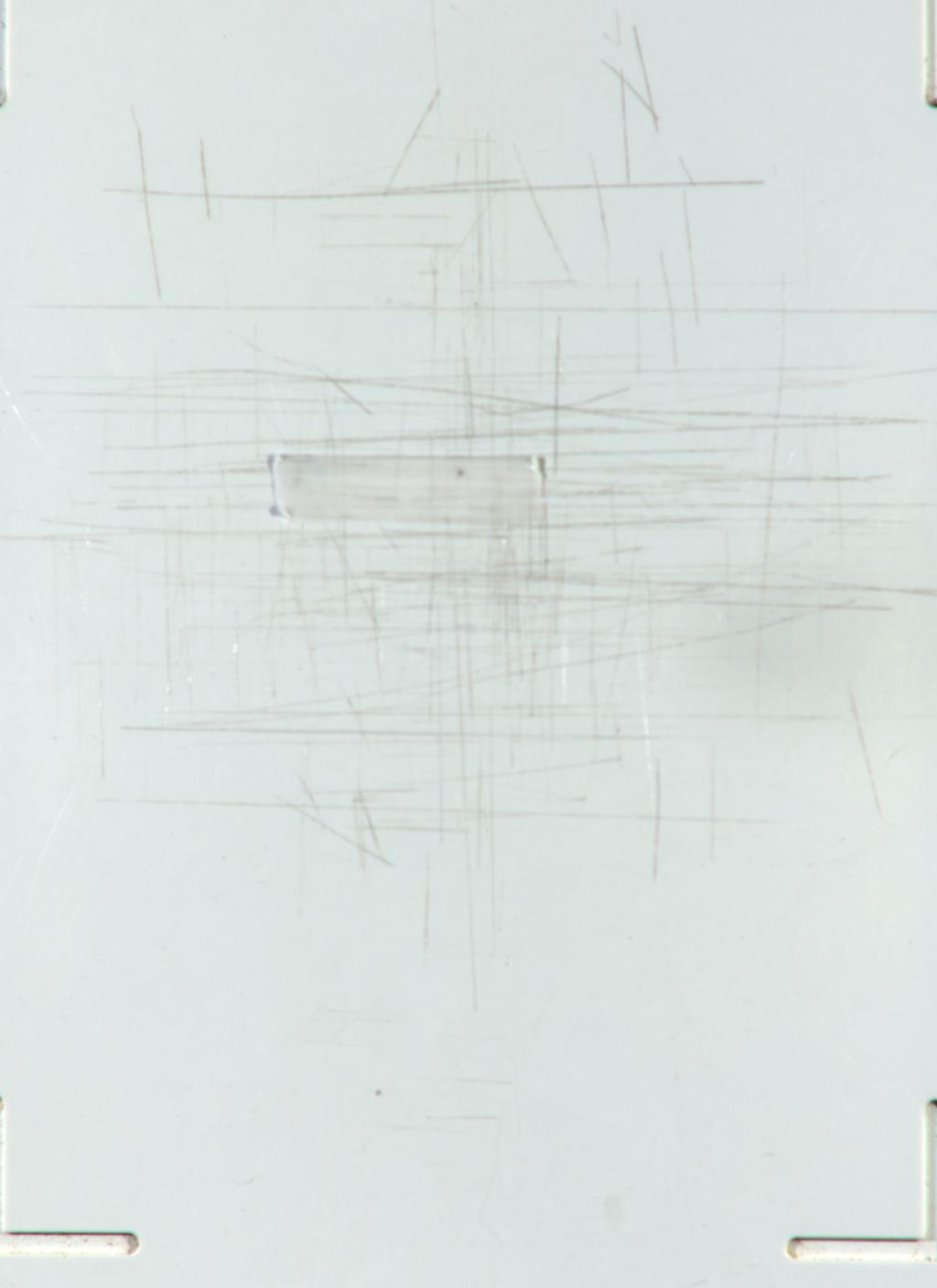

Supplement: Supplemental Information 8 [file peerj-11-15041-s008.zip › Transcriptome-related genes-raw data1/GAPDH/GAPDH-3/GAPDH-3-3.jpg]

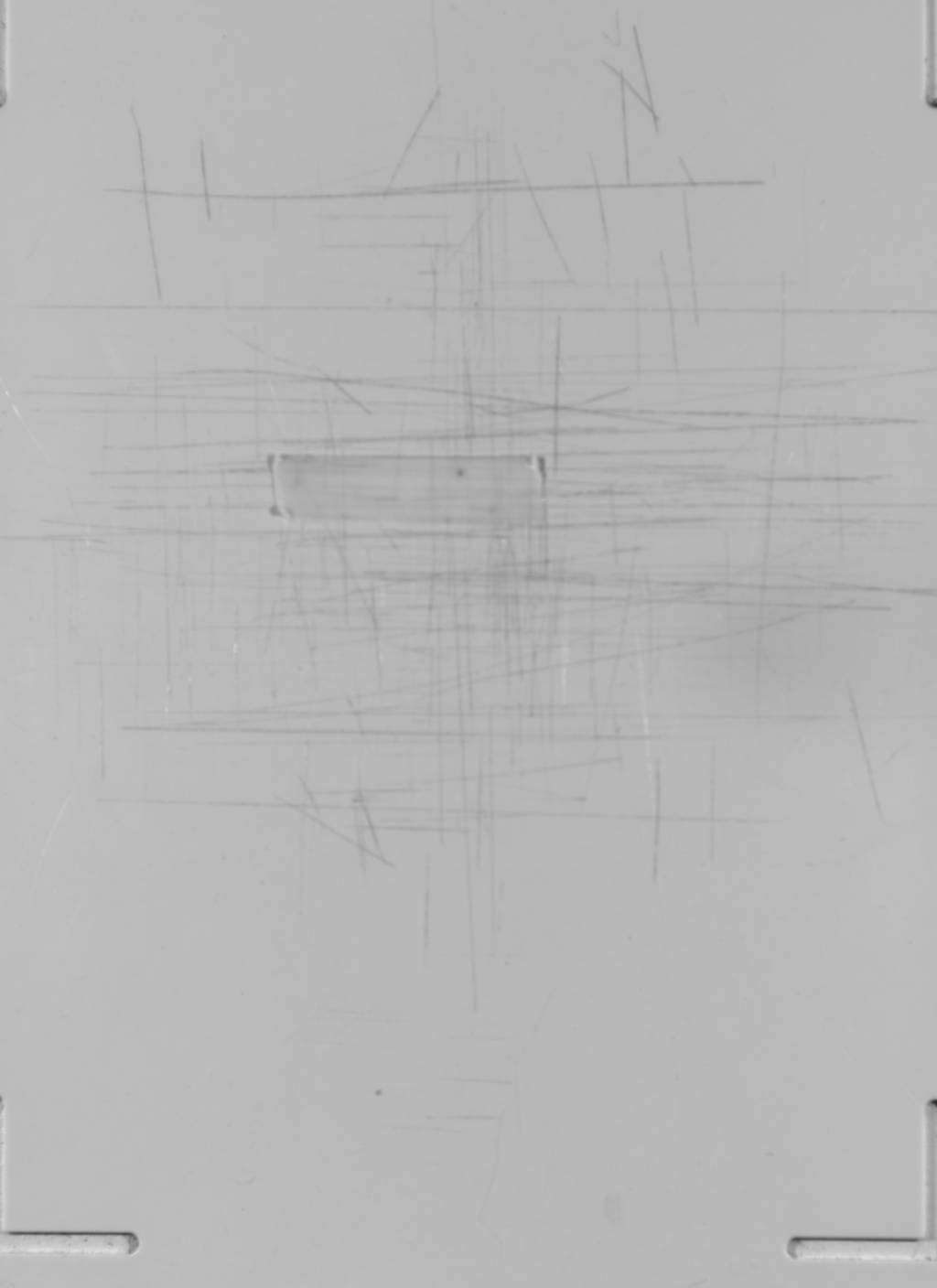

Supplement: Supplemental Information 8 [file peerj-11-15041-s008.zip › Transcriptome-related genes-raw data1/GAPDH/GAPDH-3/GAPDH-3-4.tif]

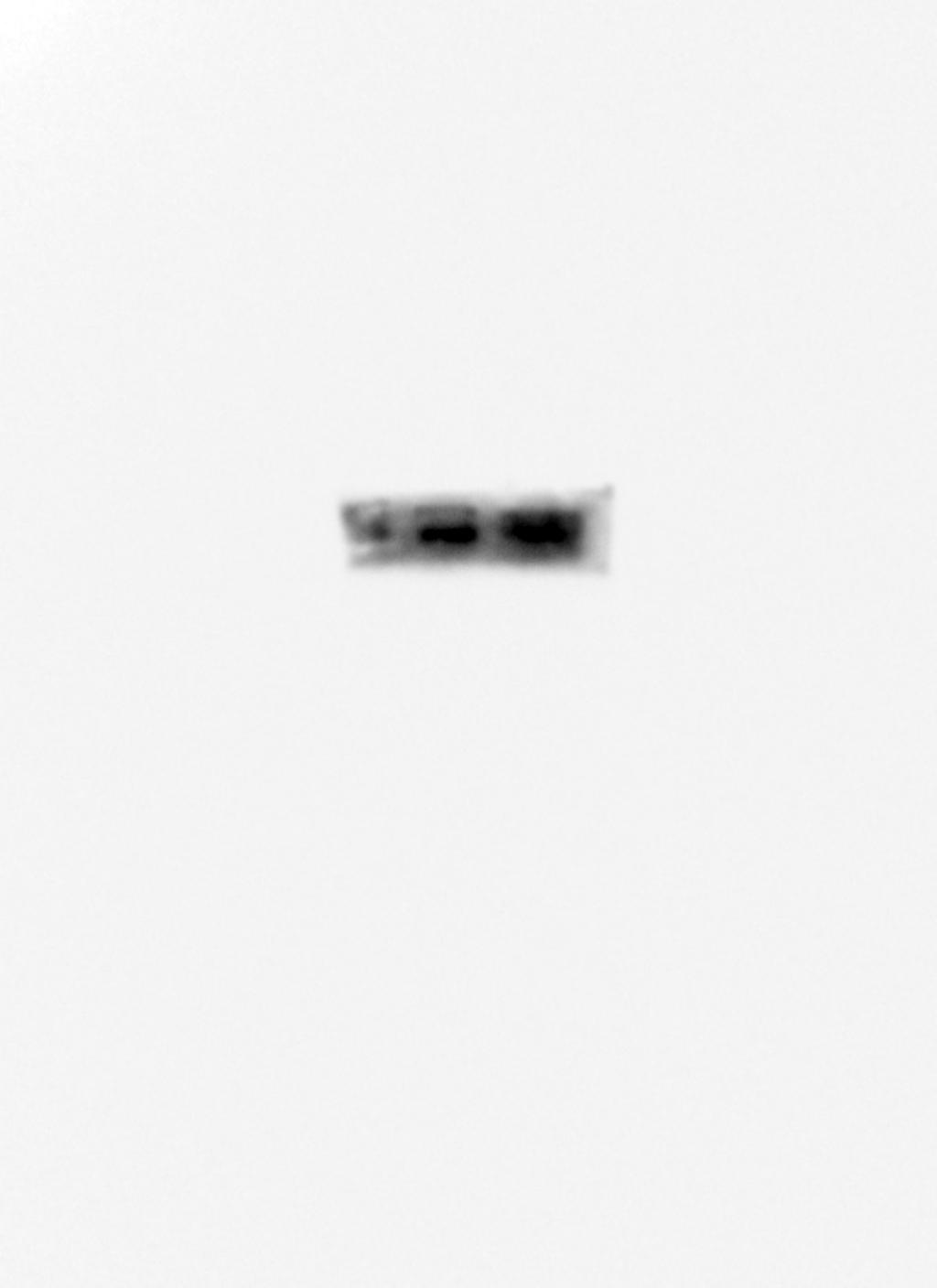

Supplement: Supplemental Information 9 [file peerj-11-15041-s009.zip › Transcriptome-related genes-raw data2/BGN/BGN-1/BGN-1-1.tif]

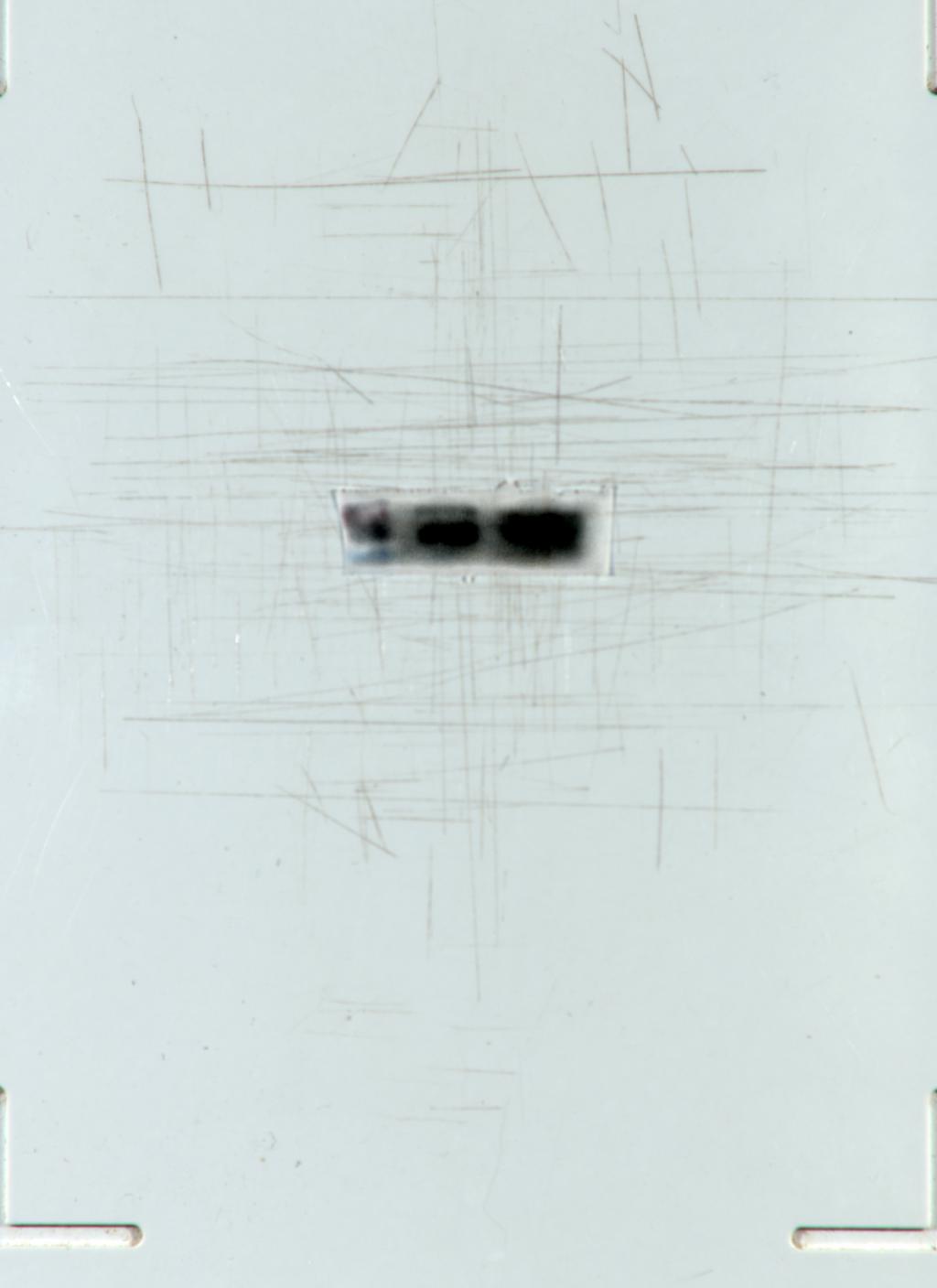

Supplement: Supplemental Information 9 [file peerj-11-15041-s009.zip › Transcriptome-related genes-raw data2/BGN/BGN-1/BGN-1-2.jpg]

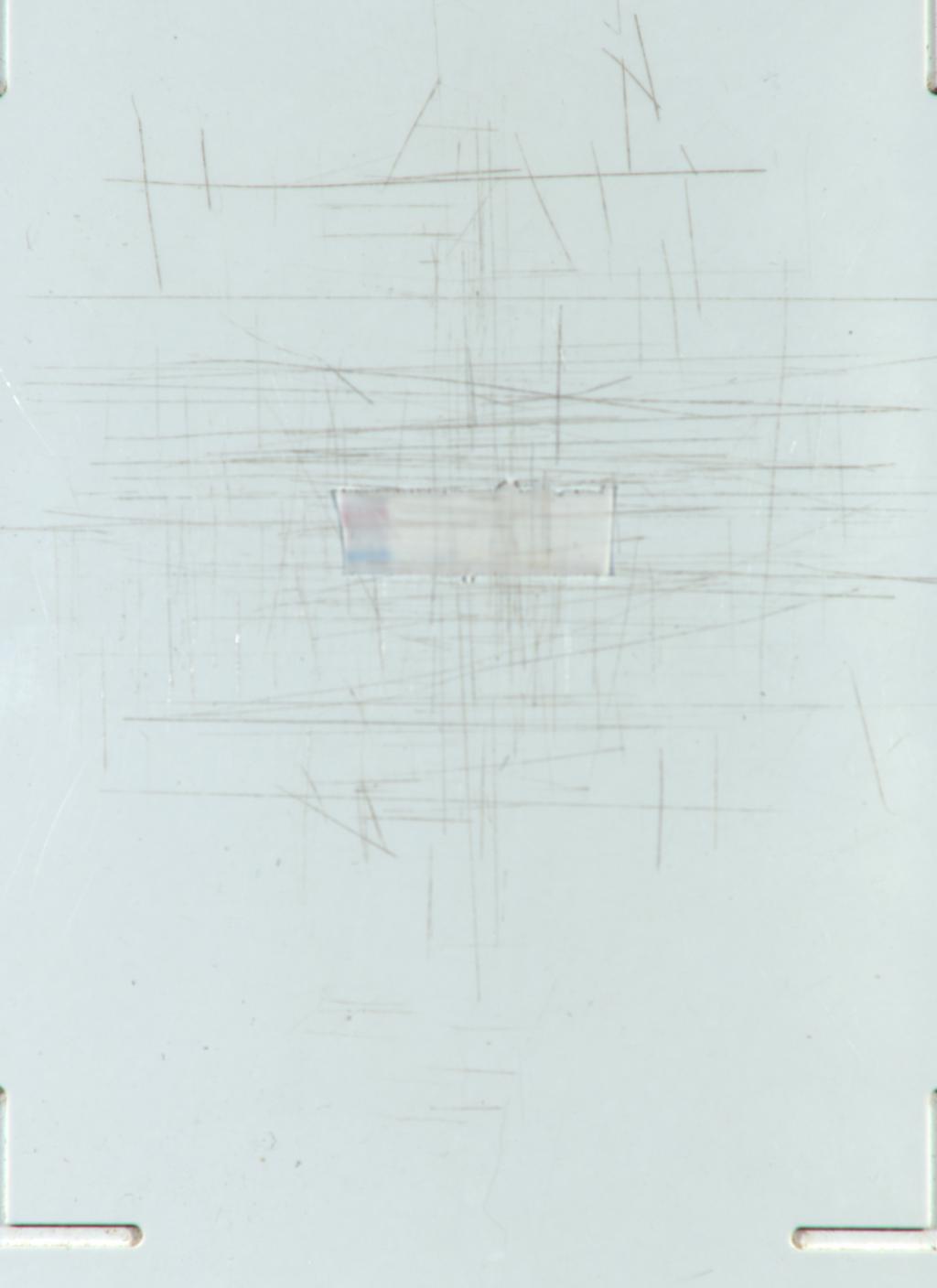

Supplement: Supplemental Information 9 [file peerj-11-15041-s009.zip › Transcriptome-related genes-raw data2/BGN/BGN-1/BGN-1-3.jpg]

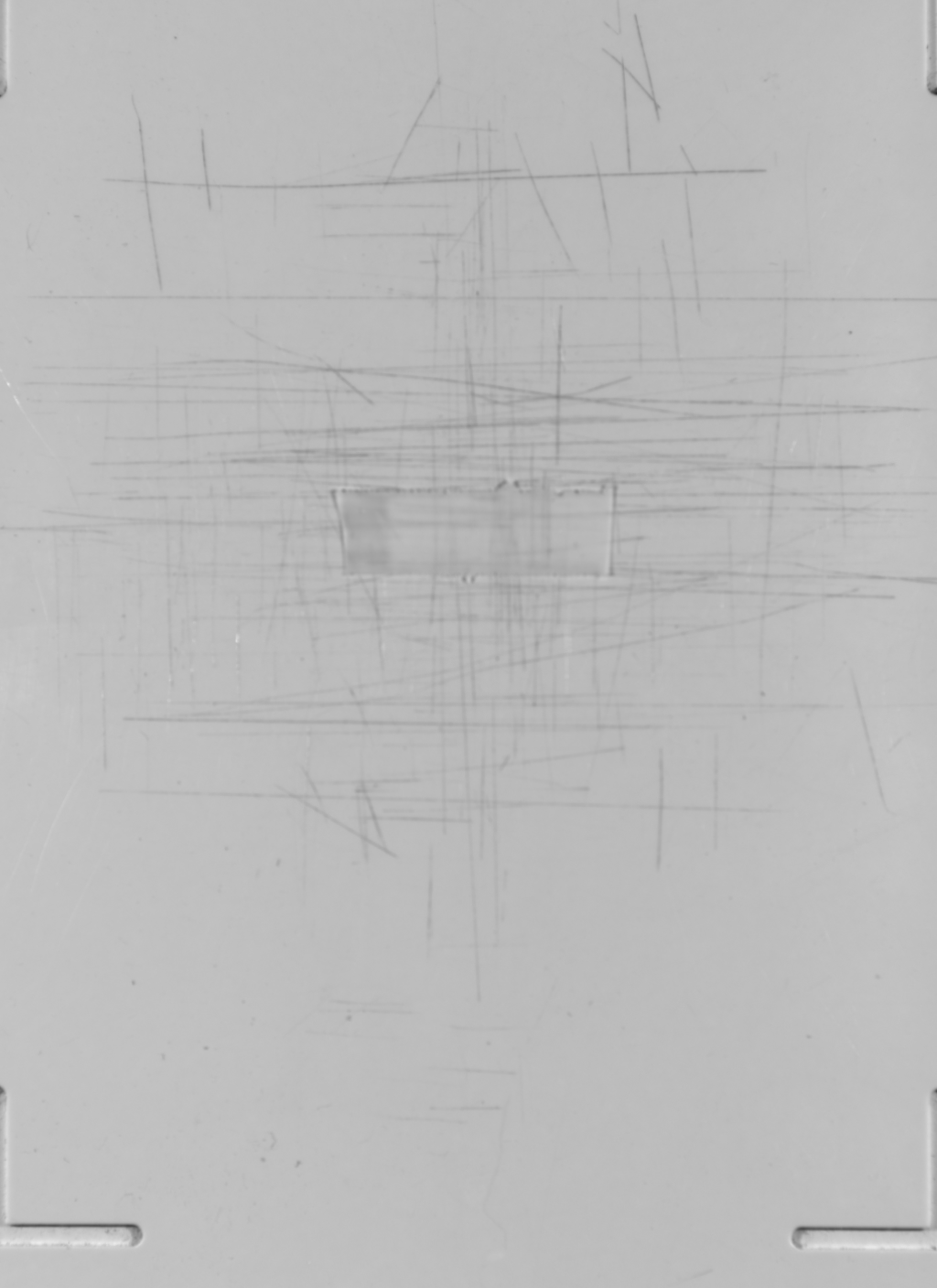

Supplement: Supplemental Information 9 [file peerj-11-15041-s009.zip › Transcriptome-related genes-raw data2/BGN/BGN-1/BGN-1-4.tif]

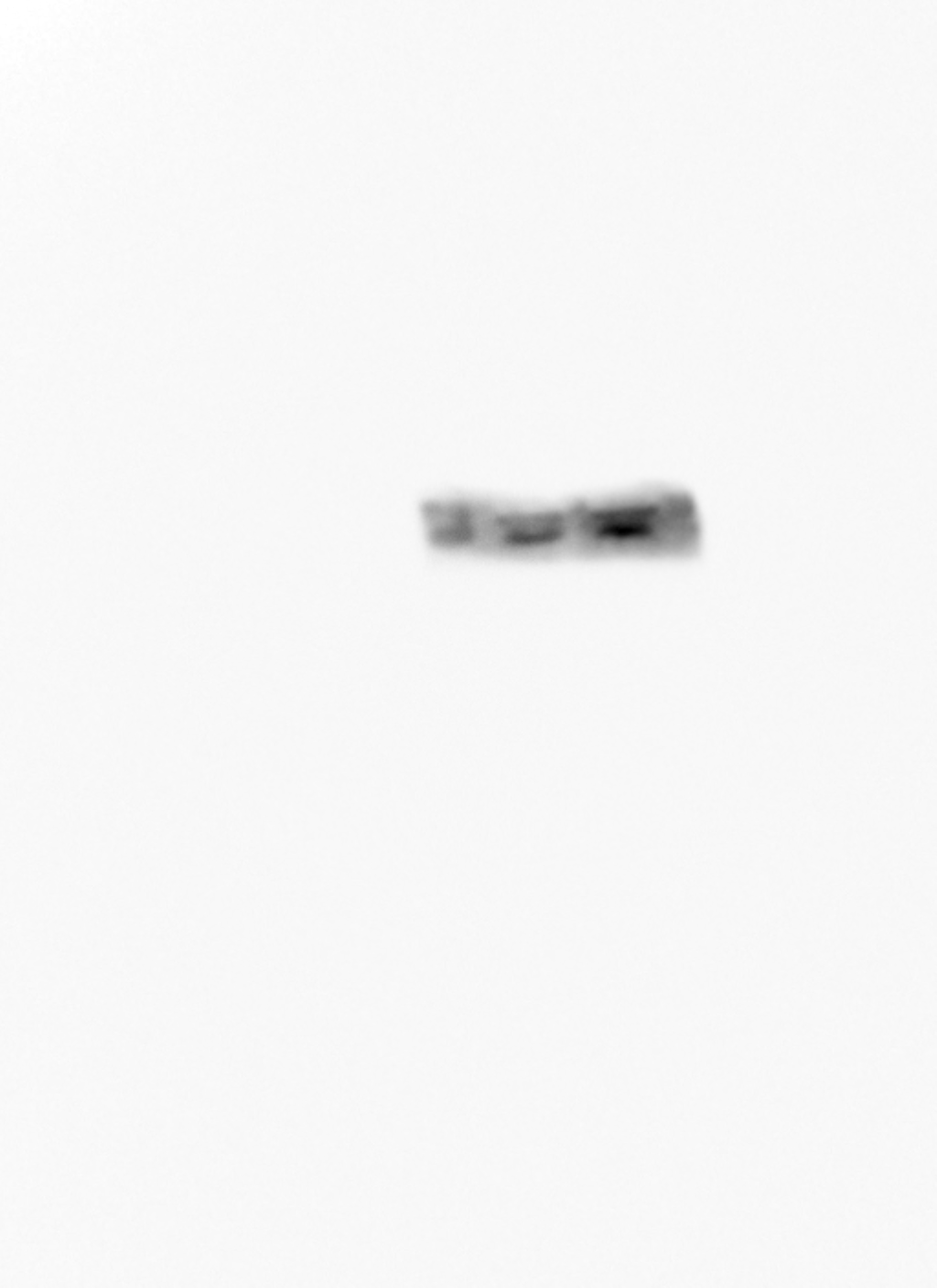

Supplement: Supplemental Information 9 [file peerj-11-15041-s009.zip › Transcriptome-related genes-raw data2/BGN/BGN-2/BGN-2-1.tif]

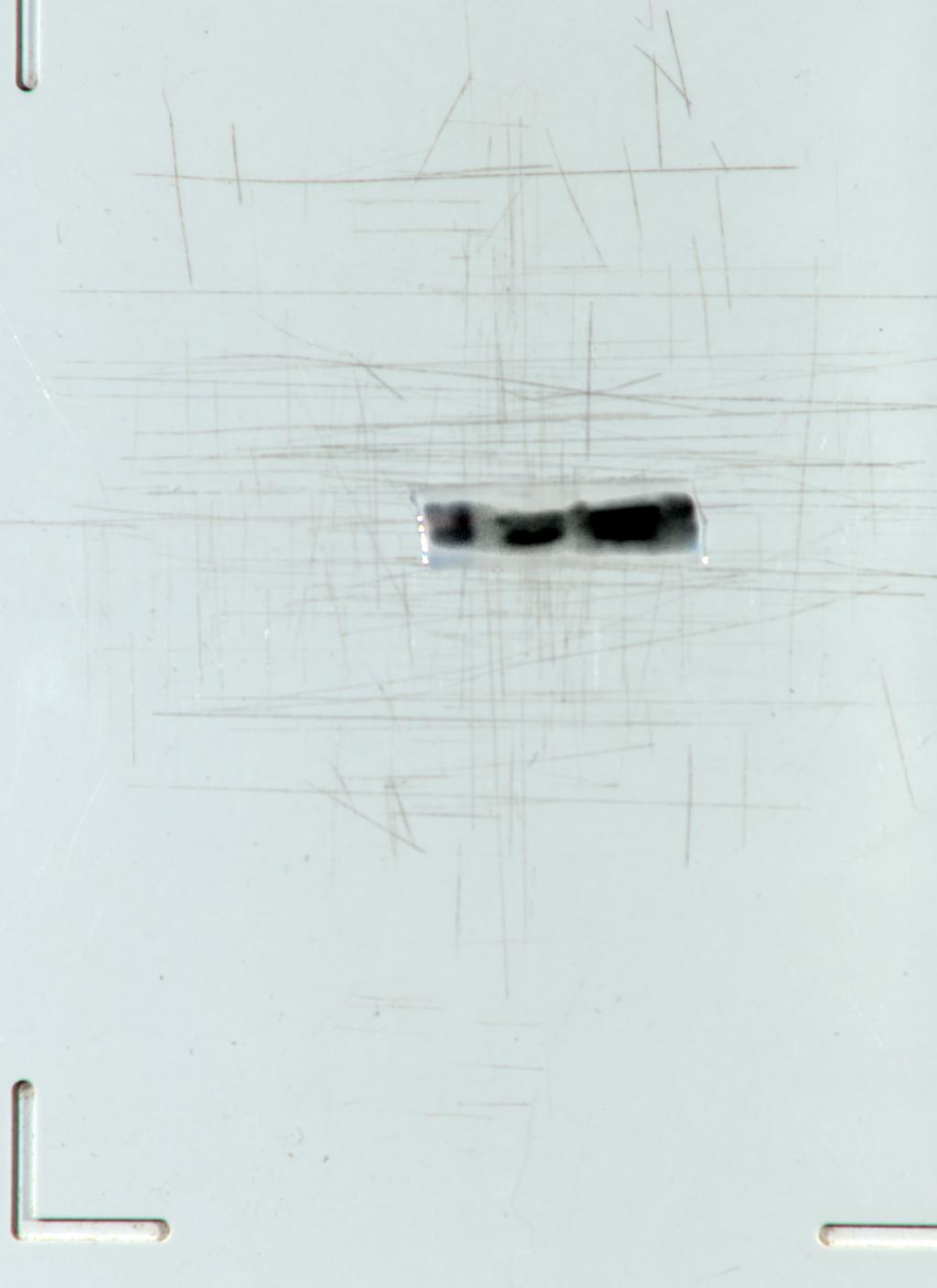

Supplement: Supplemental Information 9 [file peerj-11-15041-s009.zip › Transcriptome-related genes-raw data2/BGN/BGN-2/BGN-2-2.jpg]

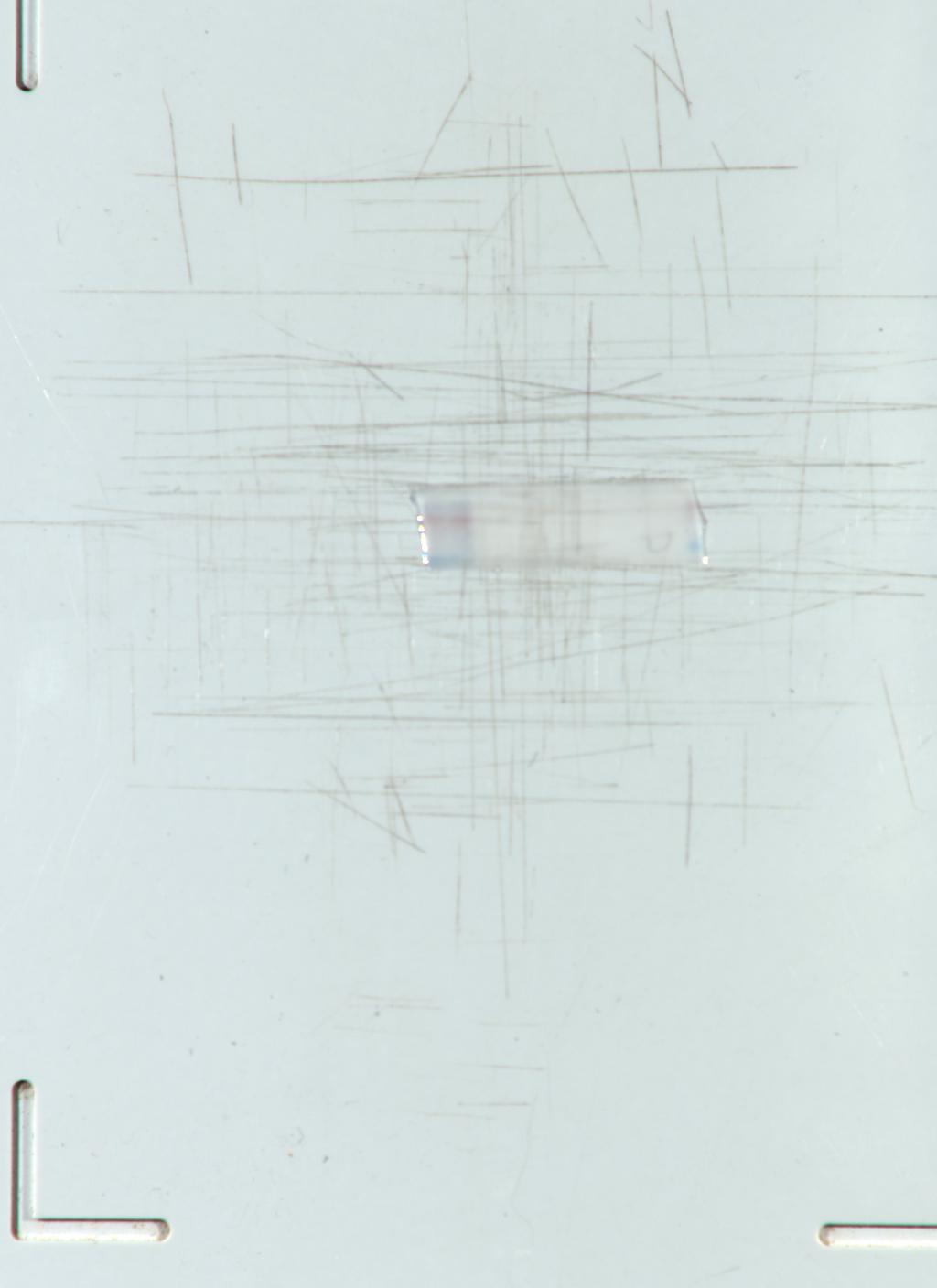

Supplement: Supplemental Information 9 [file peerj-11-15041-s009.zip › Transcriptome-related genes-raw data2/BGN/BGN-2/BGN-2-3.jpg]

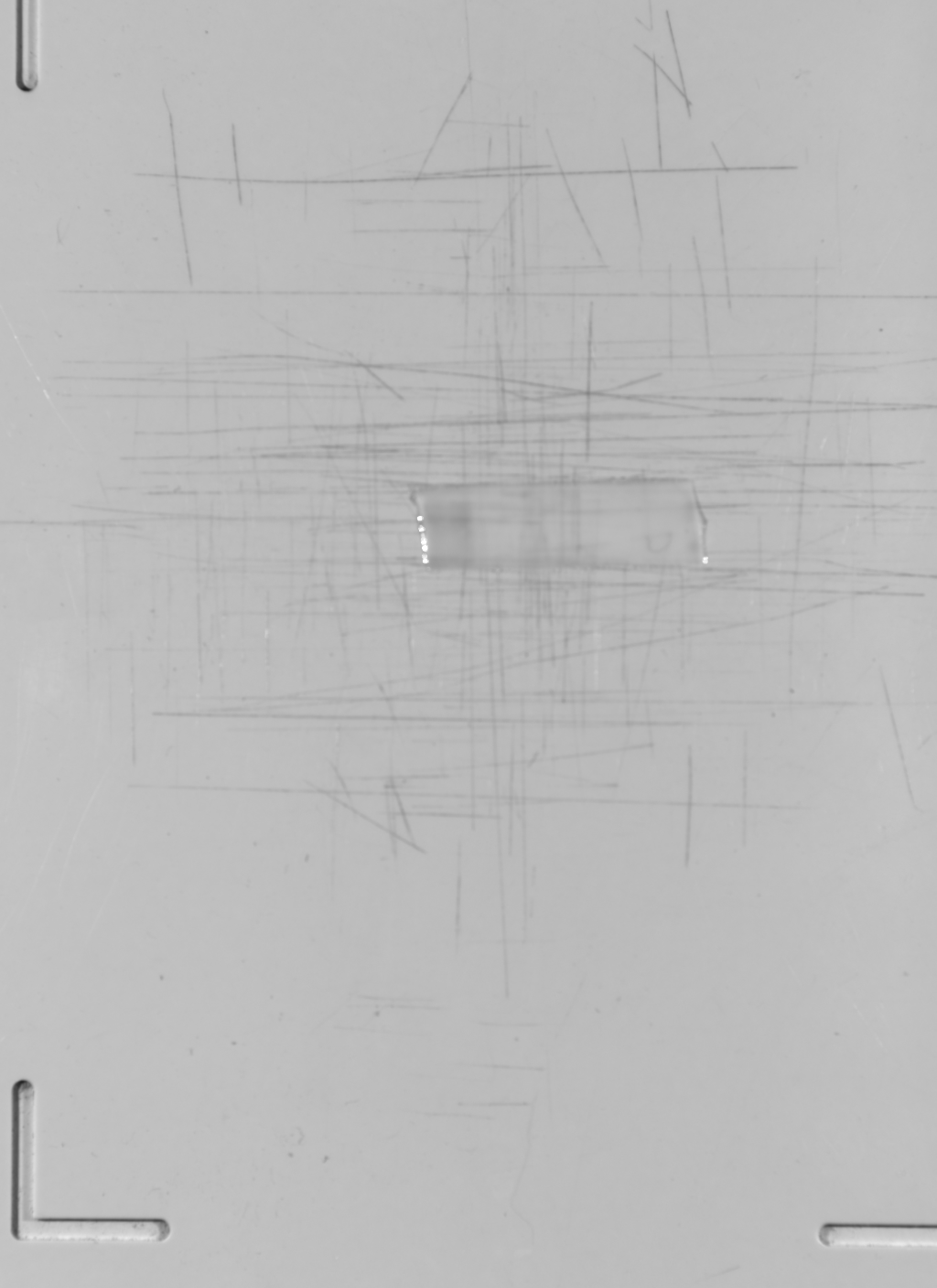

Supplement: Supplemental Information 9 [file peerj-11-15041-s009.zip › Transcriptome-related genes-raw data2/BGN/BGN-2/BGN-2-4.tif]

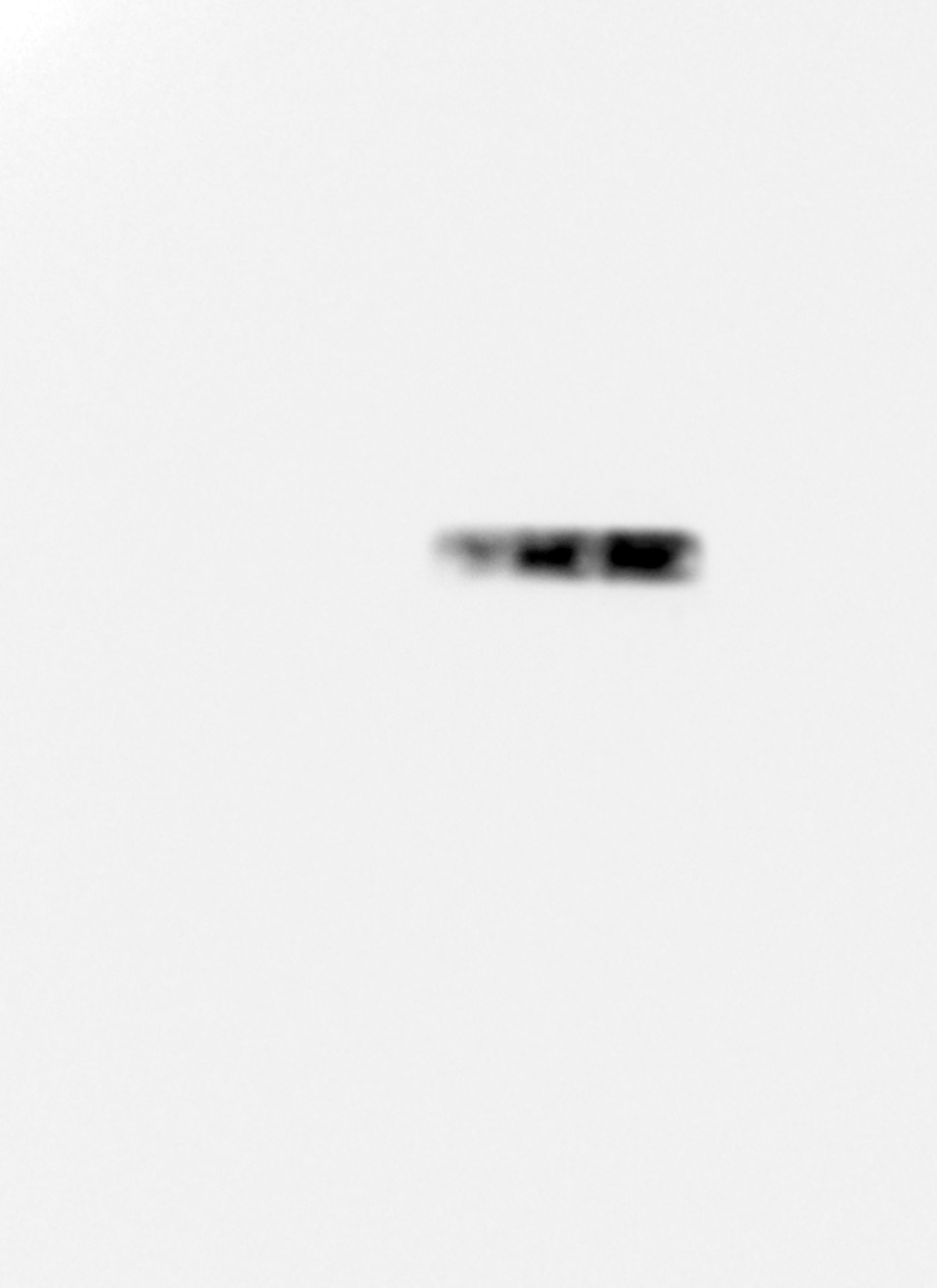

Supplement: Supplemental Information 9 [file peerj-11-15041-s009.zip › Transcriptome-related genes-raw data2/BGN/BGN-3/BGN-3-1.tif]

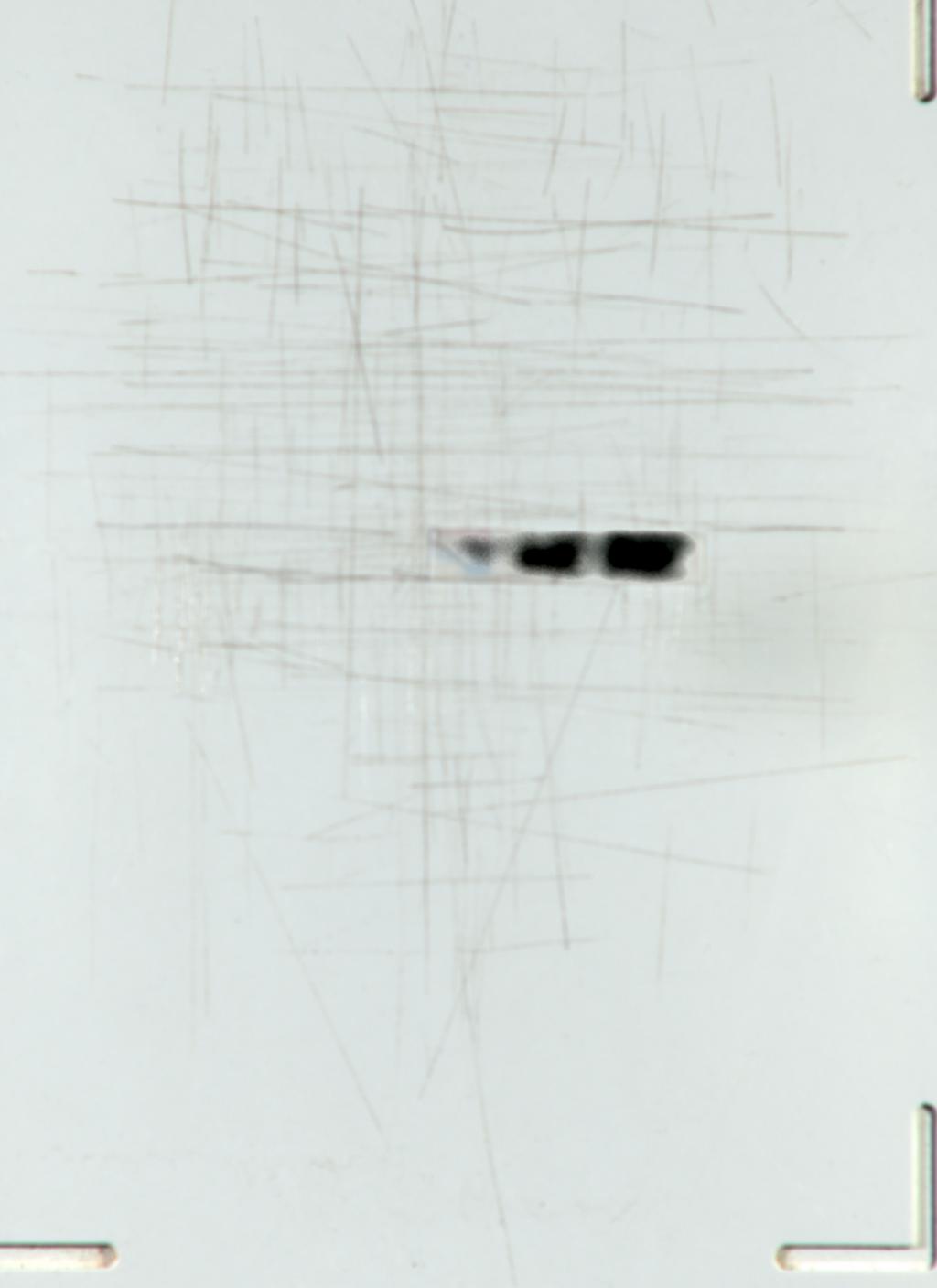

Supplement: Supplemental Information 9 [file peerj-11-15041-s009.zip › Transcriptome-related genes-raw data2/BGN/BGN-3/BGN-3-2.jpg]

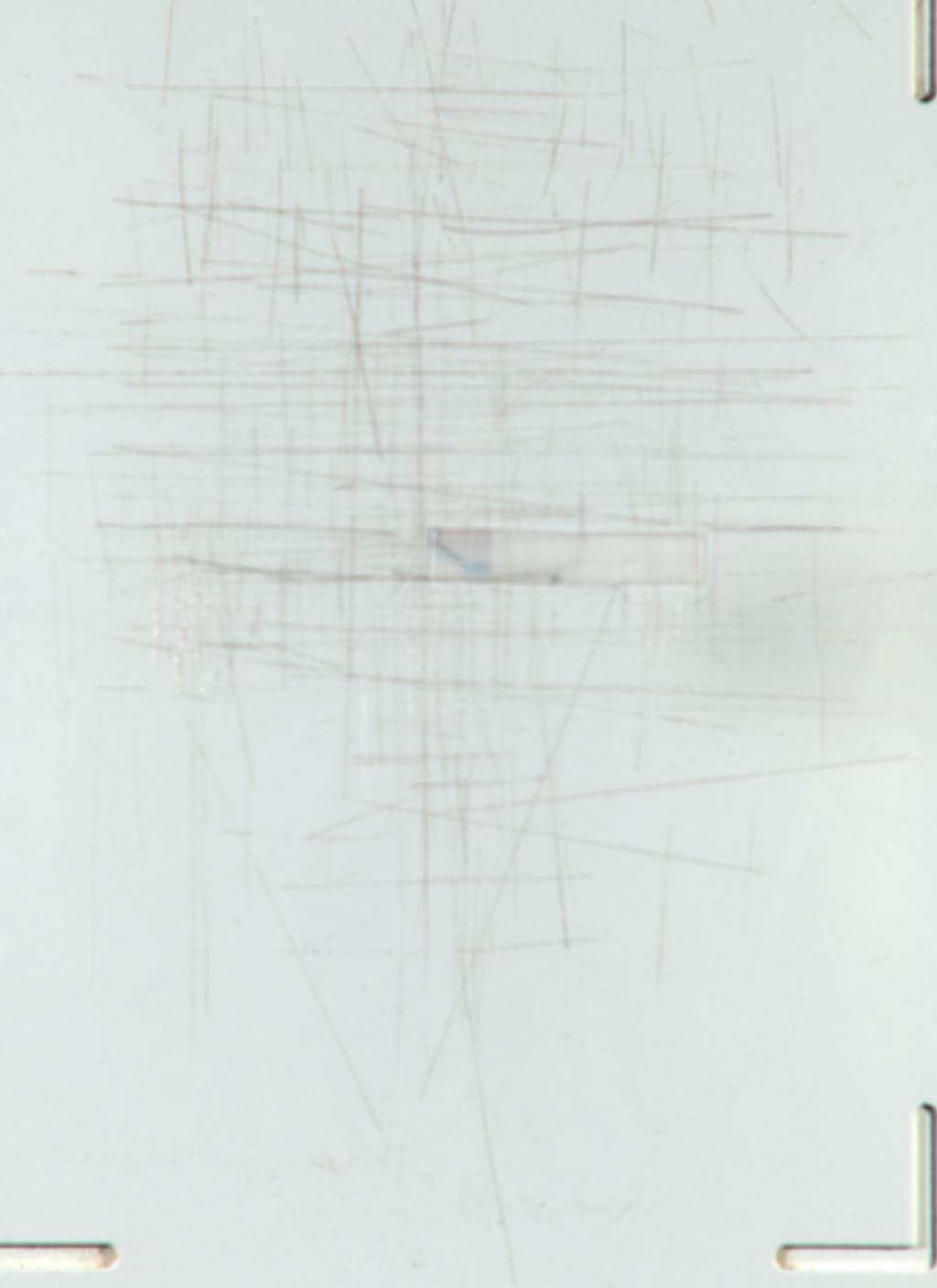

Supplement: Supplemental Information 9 [file peerj-11-15041-s009.zip › Transcriptome-related genes-raw data2/BGN/BGN-3/BGN-3-3.jpg]

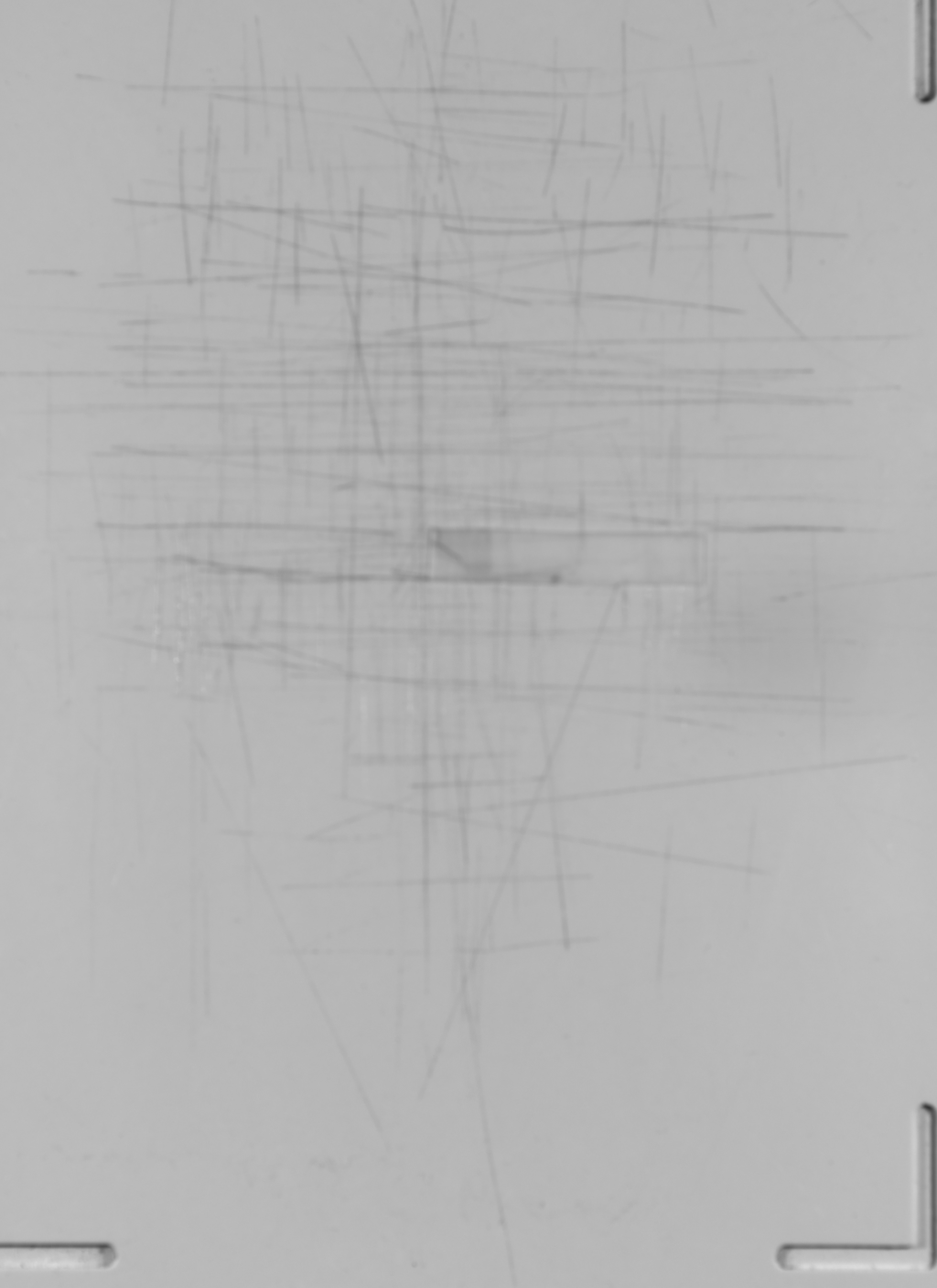

Supplement: Supplemental Information 9 [file peerj-11-15041-s009.zip › Transcriptome-related genes-raw data2/BGN/BGN-3/BGN-3-4.tif]

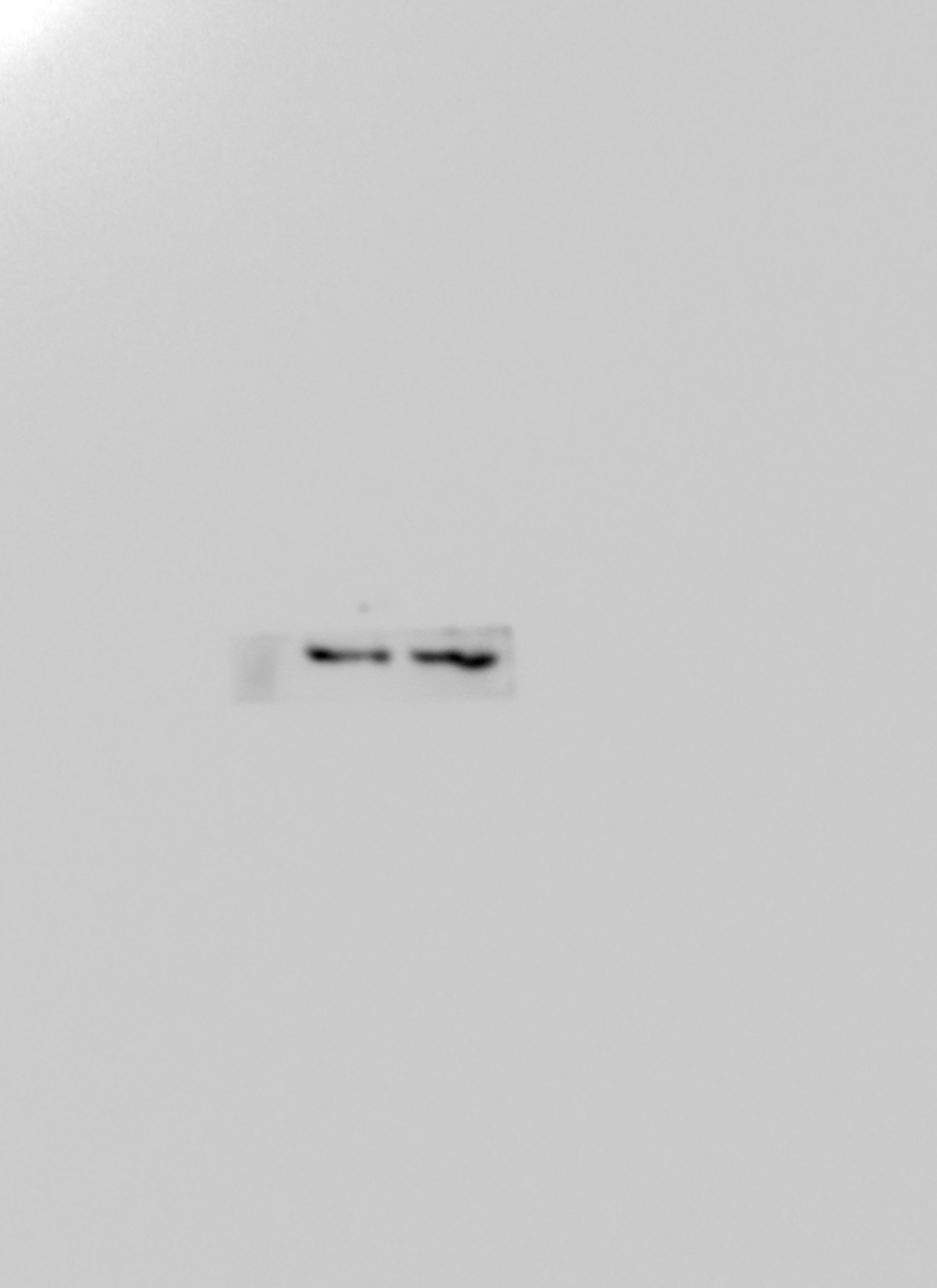

Supplement: Supplemental Information 9 [file peerj-11-15041-s009.zip › Transcriptome-related genes-raw data2/SPARC/SPARC-1/SPARC-1-1.tif]

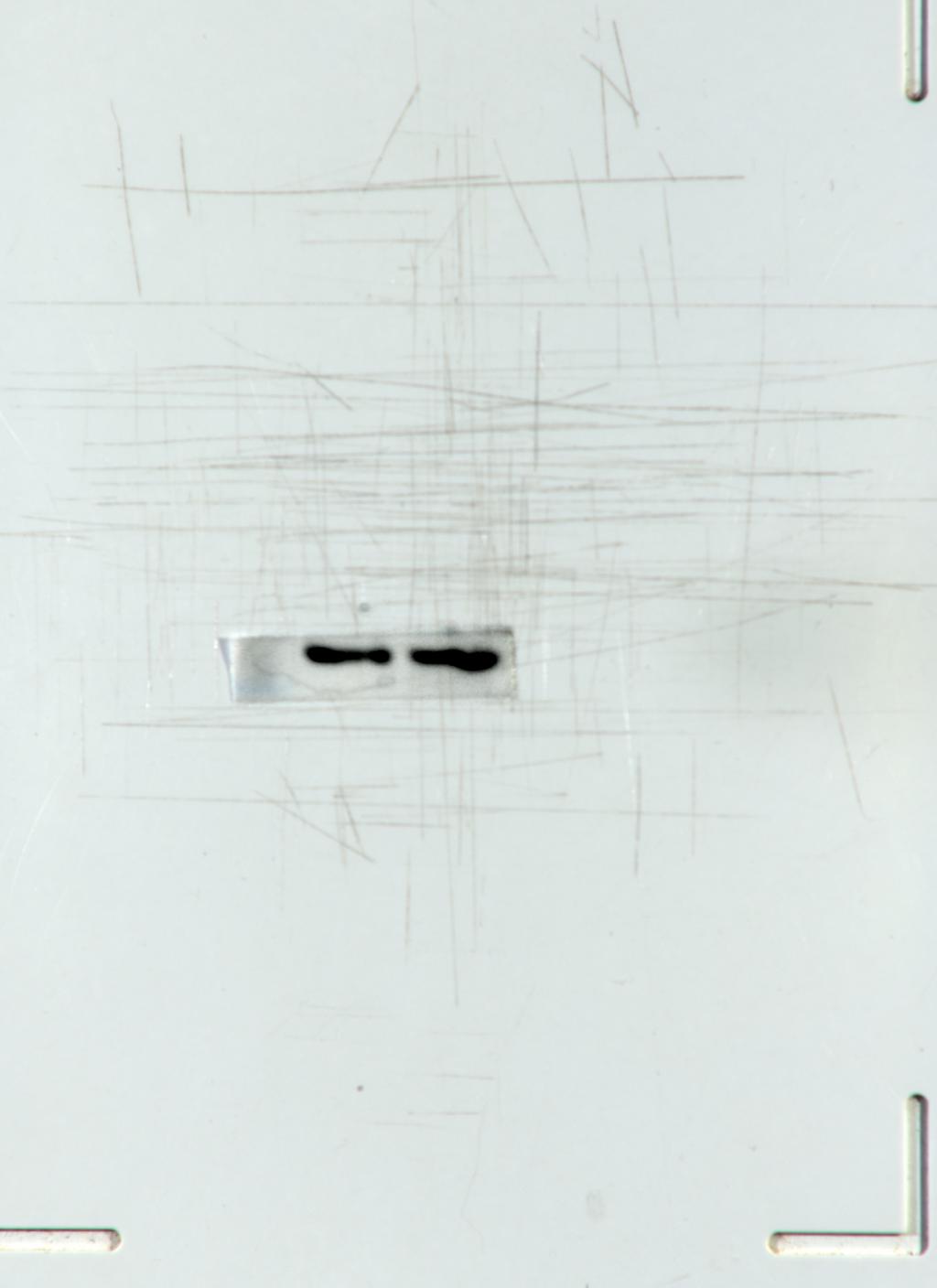

Supplement: Supplemental Information 9 [file peerj-11-15041-s009.zip › Transcriptome-related genes-raw data2/SPARC/SPARC-1/SPARC-1-2.jpg]

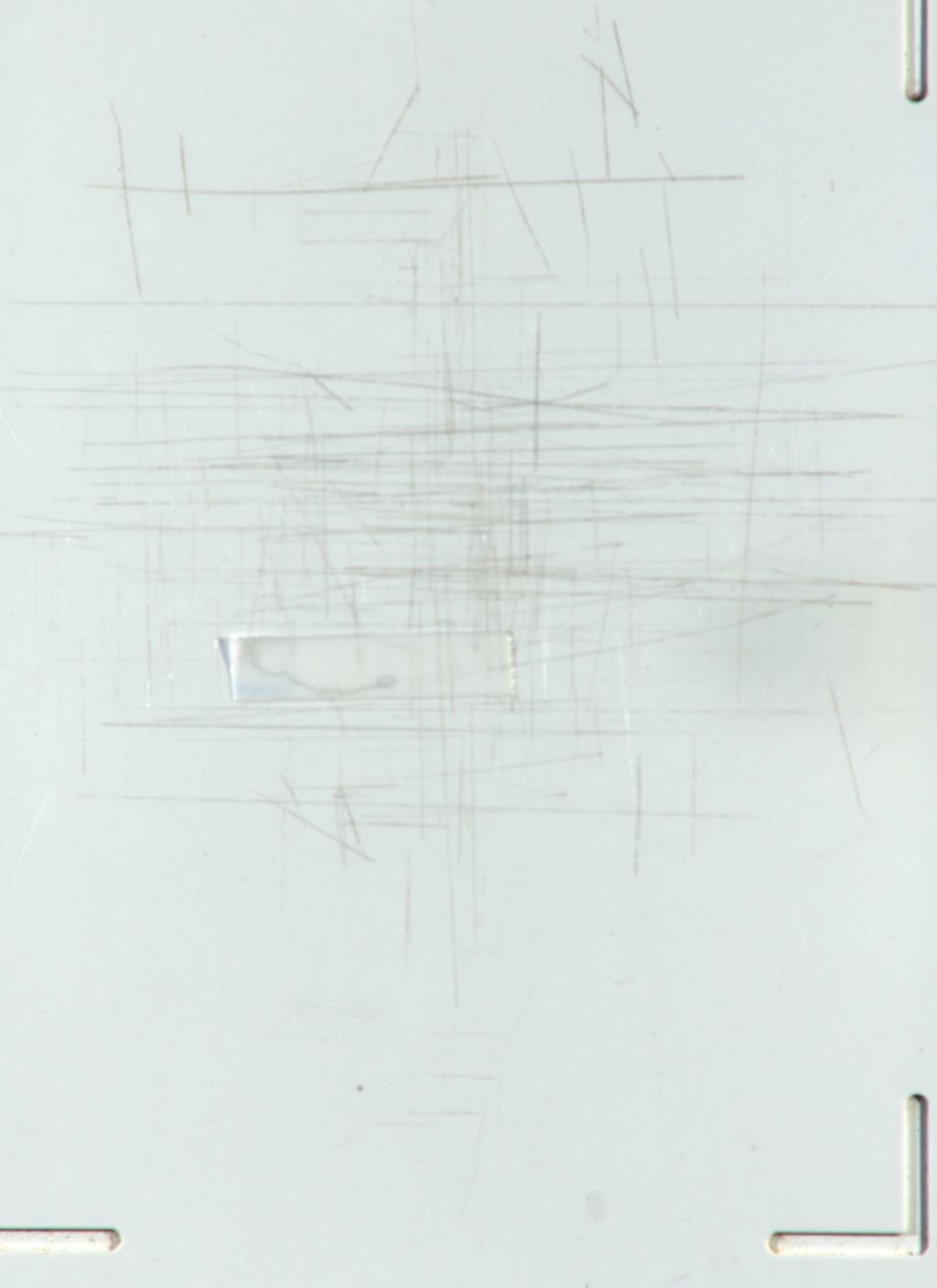

Supplement: Supplemental Information 9 [file peerj-11-15041-s009.zip › Transcriptome-related genes-raw data2/SPARC/SPARC-1/SPARC-1-3.jpg]

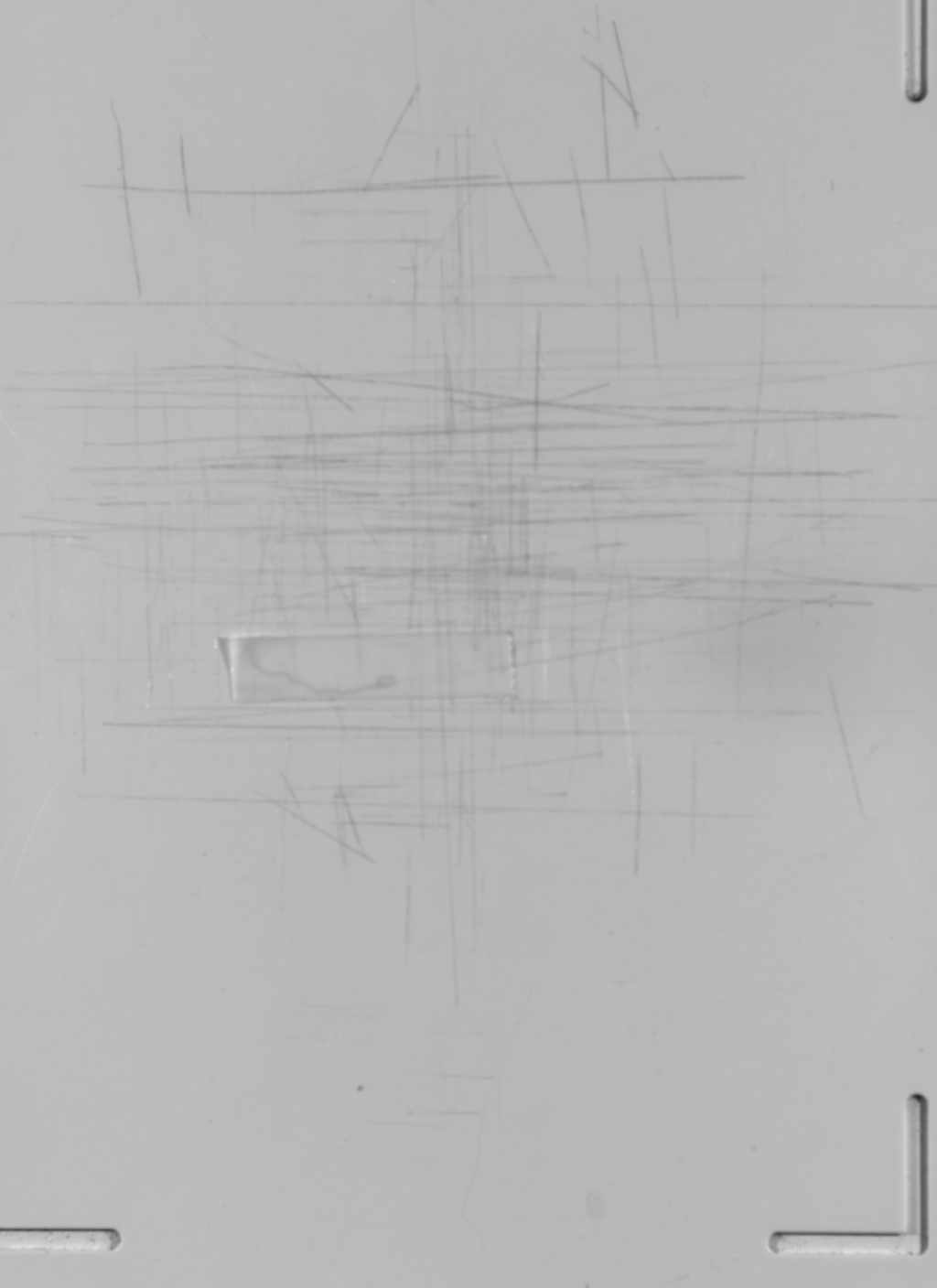

Supplement: Supplemental Information 9 [file peerj-11-15041-s009.zip › Transcriptome-related genes-raw data2/SPARC/SPARC-1/SPARC-1-4.tif]

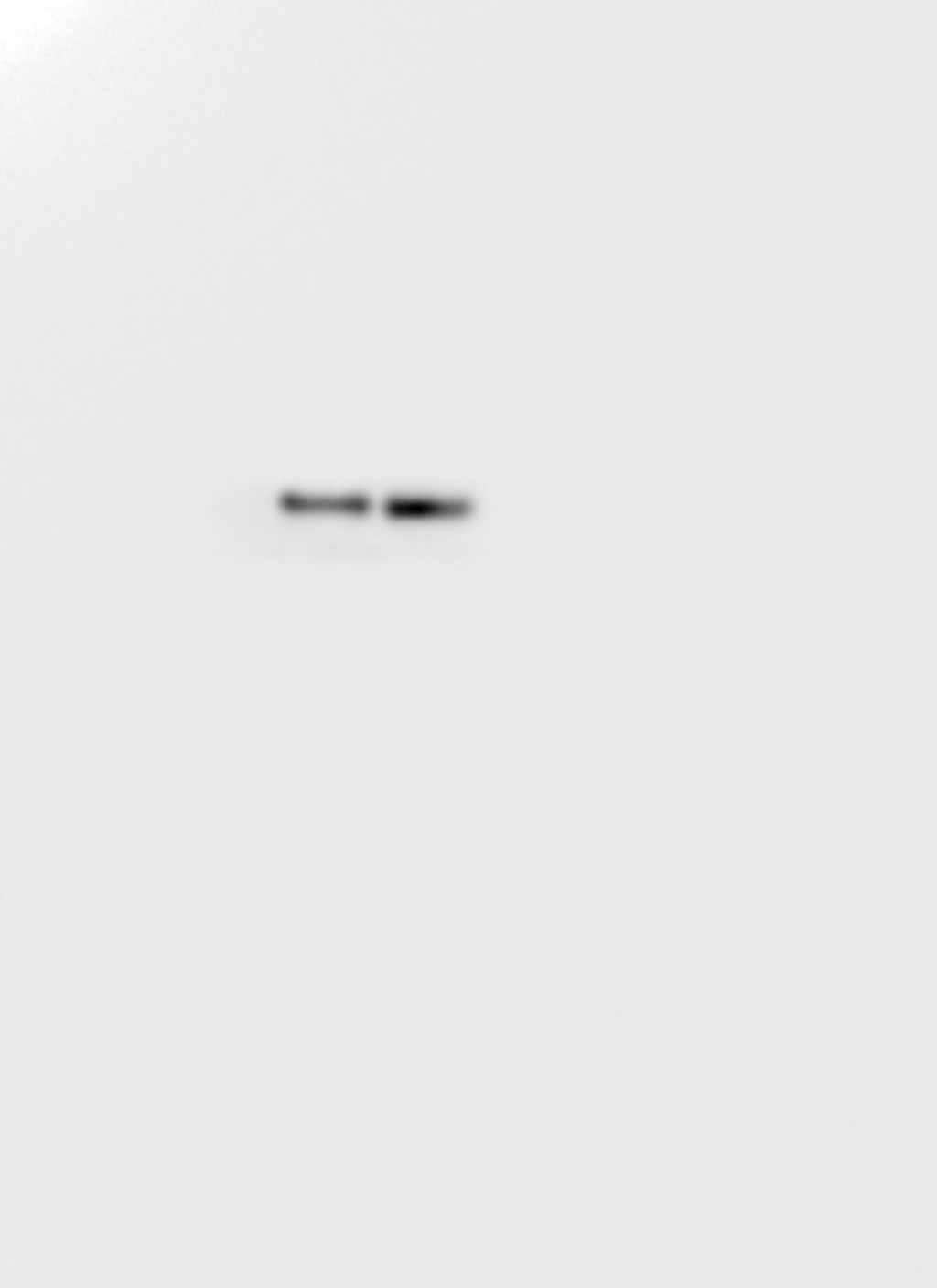

Supplement: Supplemental Information 9 [file peerj-11-15041-s009.zip › Transcriptome-related genes-raw data2/SPARC/SPARC-2/SPARC-2-1.tif]

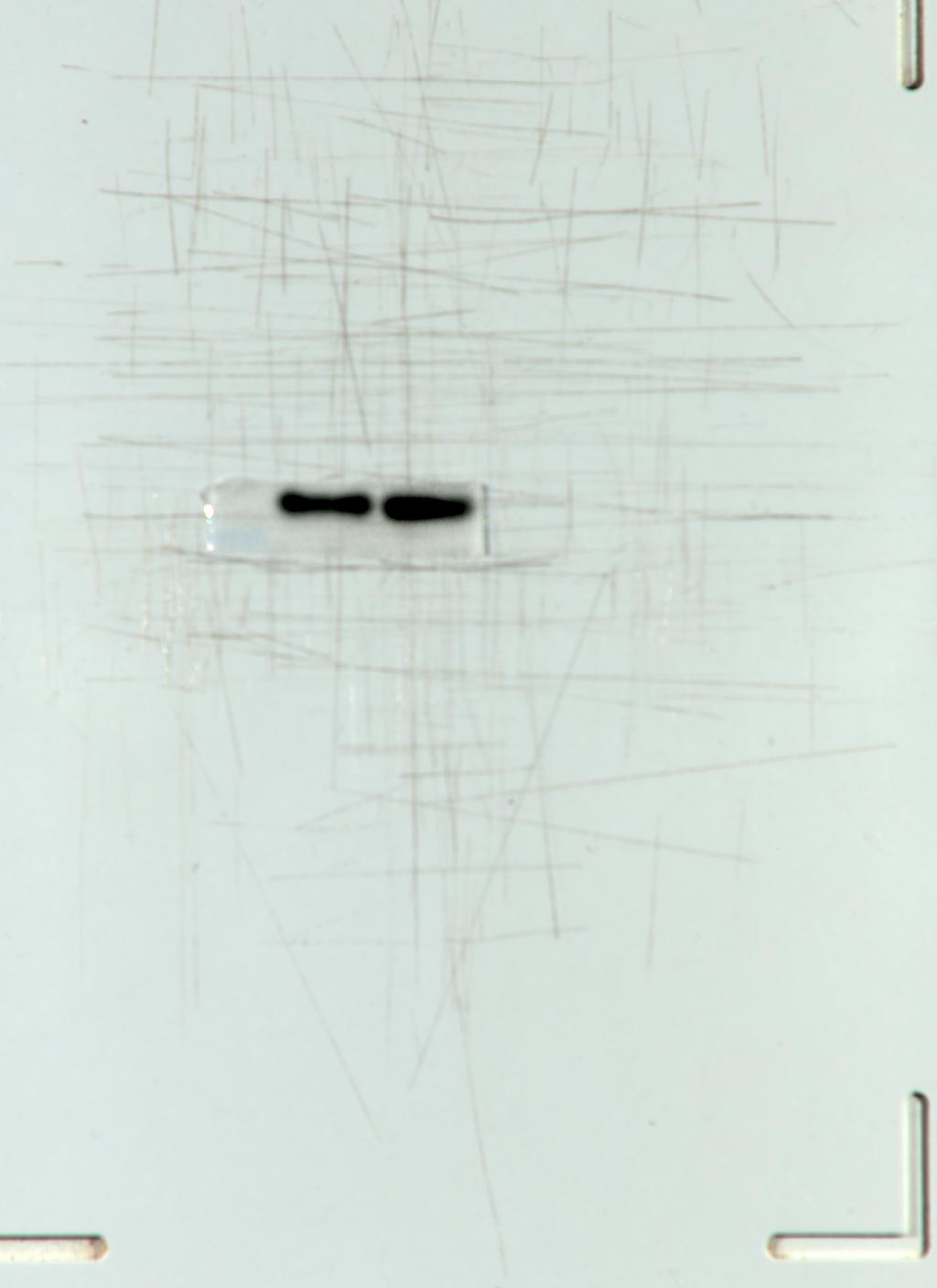

Supplement: Supplemental Information 9 [file peerj-11-15041-s009.zip › Transcriptome-related genes-raw data2/SPARC/SPARC-2/SPARC-2-2.jpg]

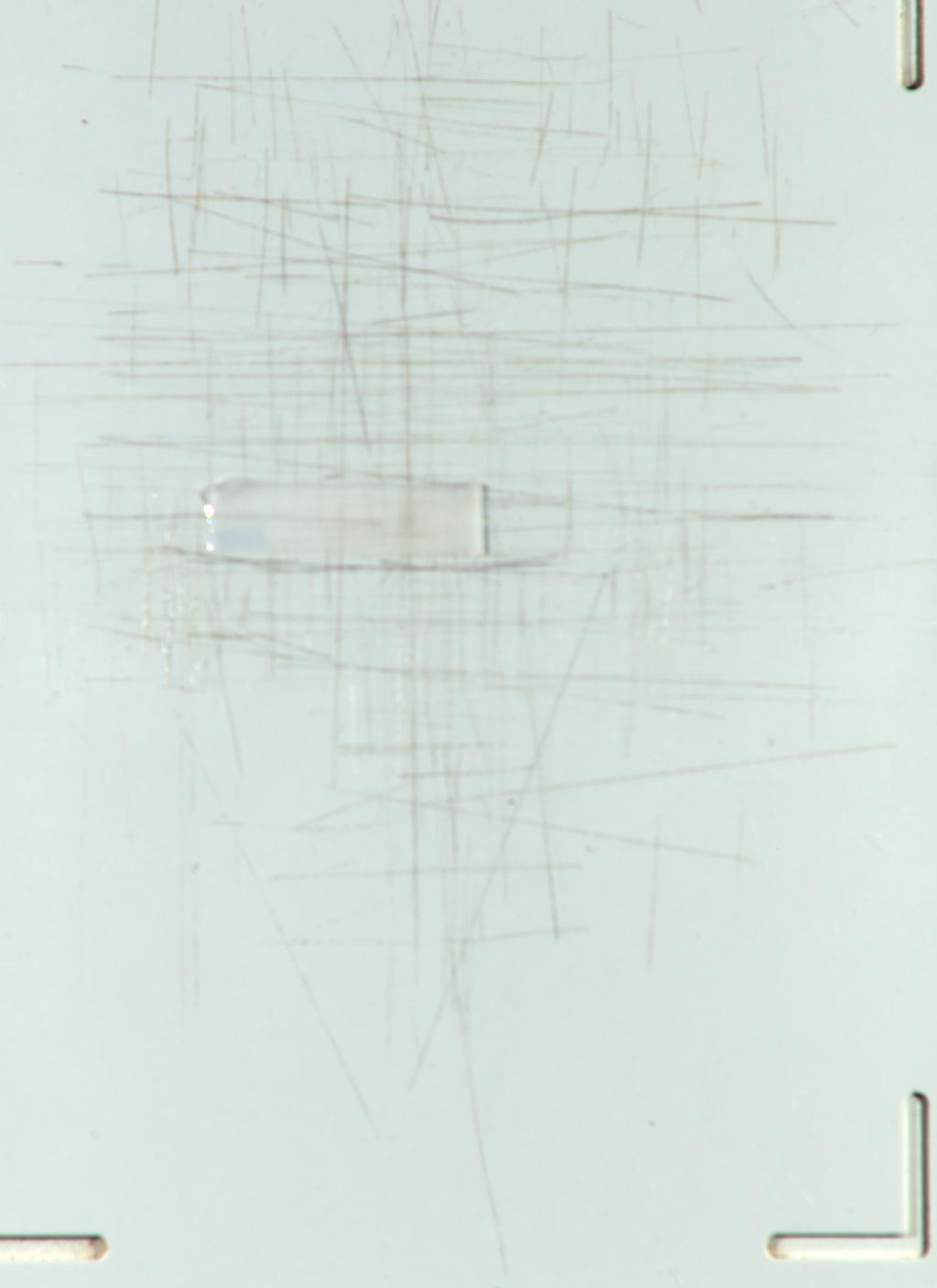

Supplement: Supplemental Information 9 [file peerj-11-15041-s009.zip › Transcriptome-related genes-raw data2/SPARC/SPARC-2/SPARC-2-3.jpg]

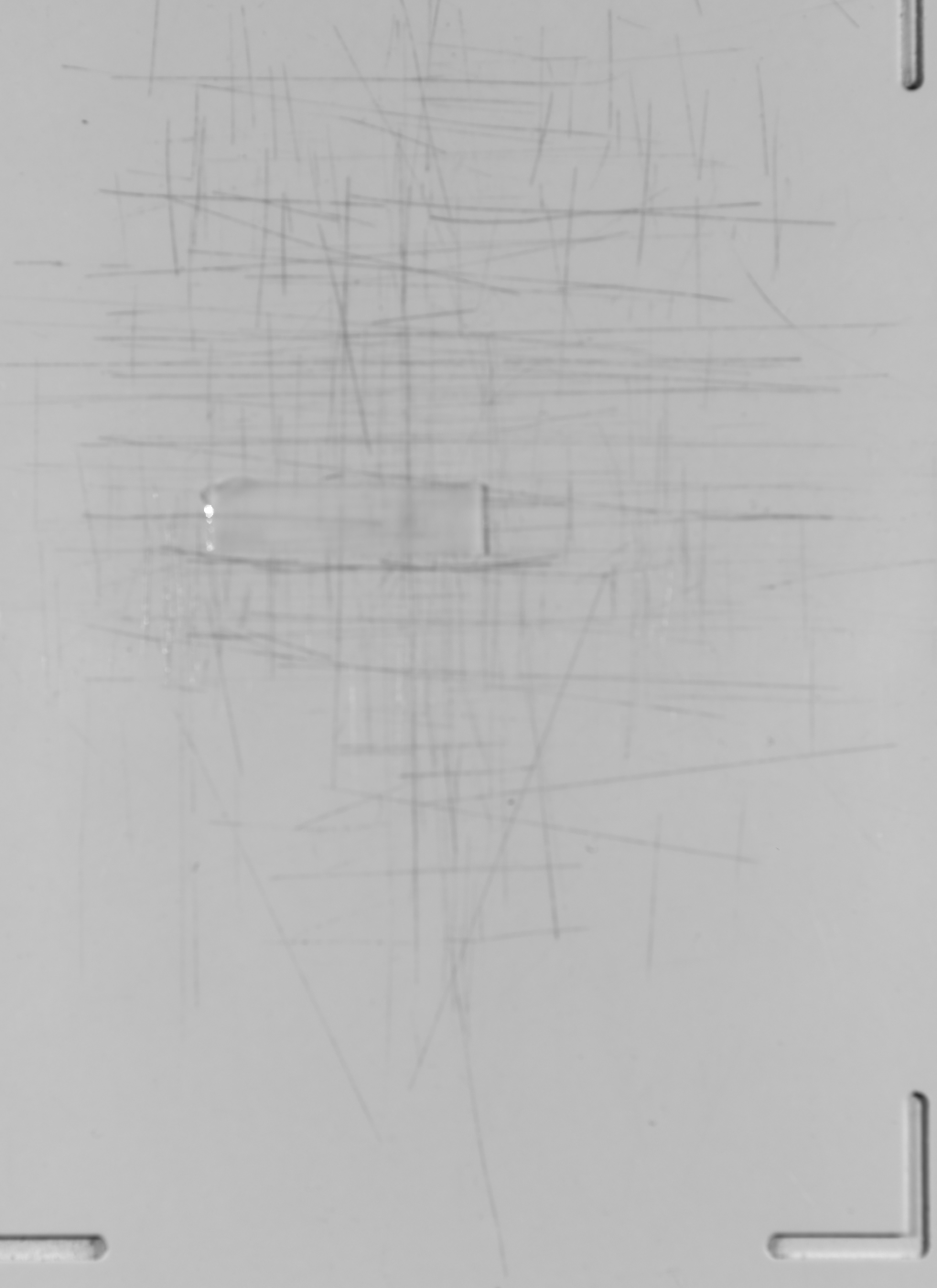

Supplement: Supplemental Information 9 [file peerj-11-15041-s009.zip › Transcriptome-related genes-raw data2/SPARC/SPARC-2/SPARC-2-4.tif]

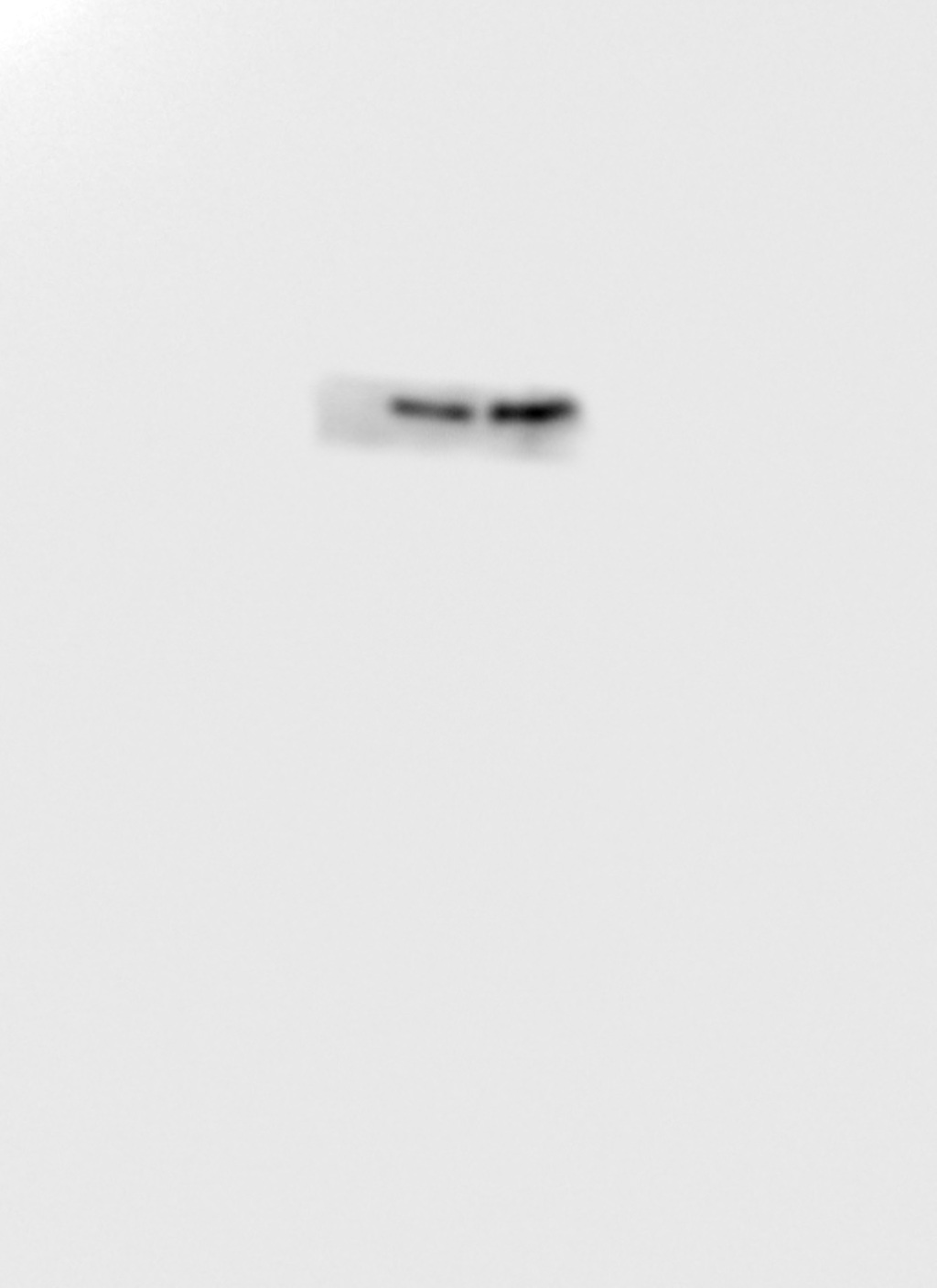

Supplement: Supplemental Information 9 [file peerj-11-15041-s009.zip › Transcriptome-related genes-raw data2/SPARC/SPARC-3/SPARC-3-1.tif]

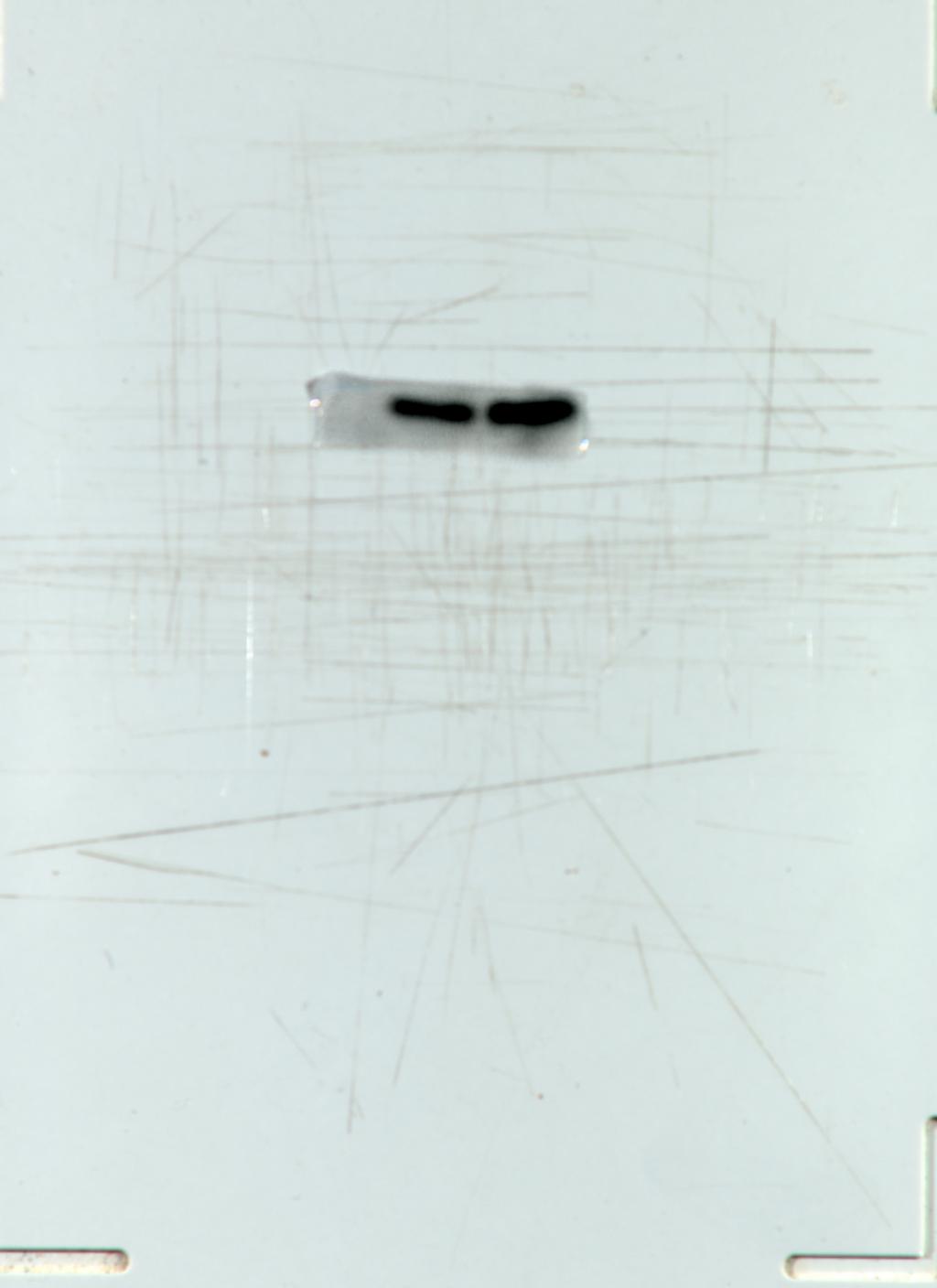

Supplement: Supplemental Information 9 [file peerj-11-15041-s009.zip › Transcriptome-related genes-raw data2/SPARC/SPARC-3/SPARC-3-2.jpg]

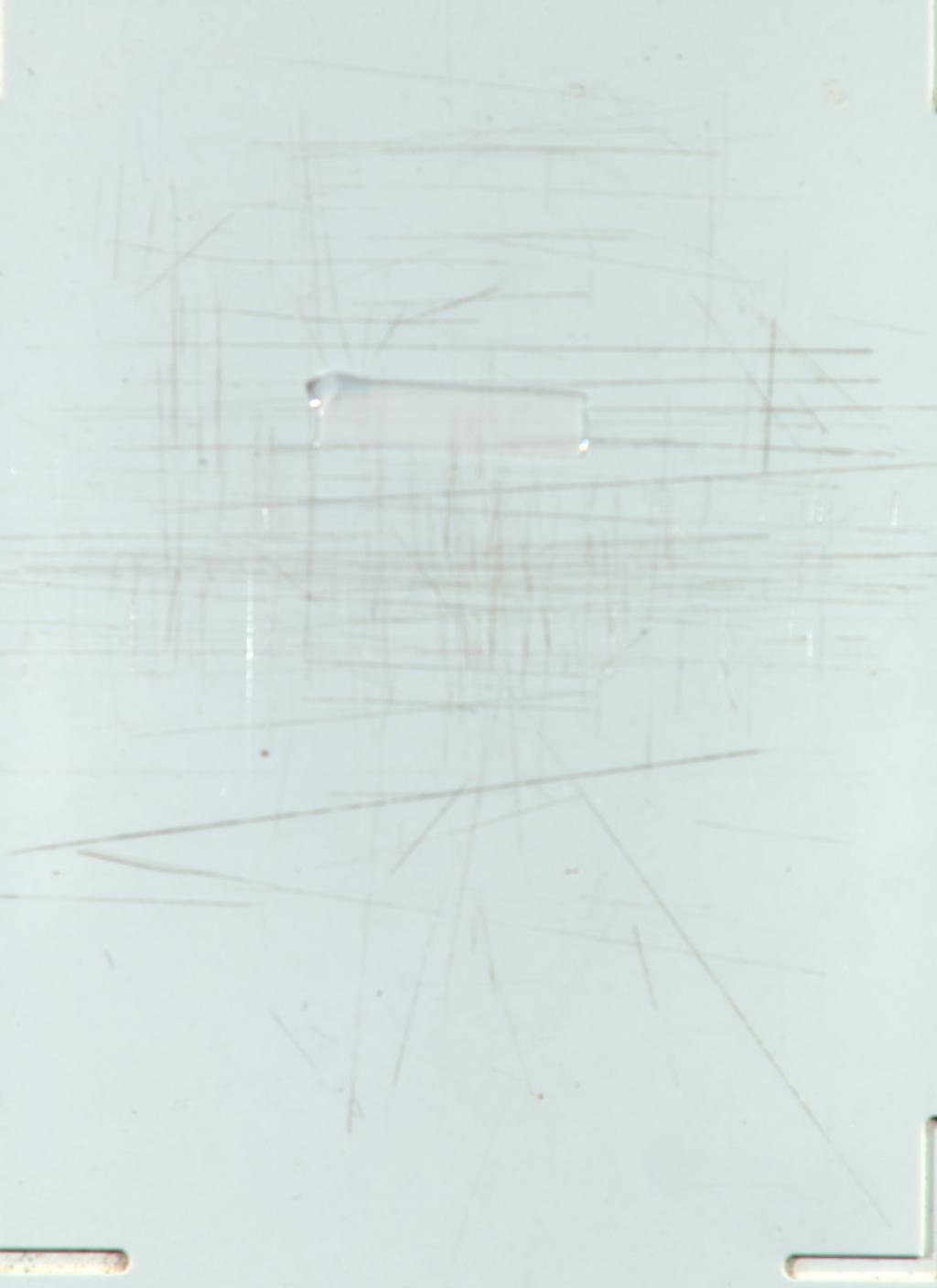

Supplement: Supplemental Information 9 [file peerj-11-15041-s009.zip › Transcriptome-related genes-raw data2/SPARC/SPARC-3/SPARC-3-3.jpg]

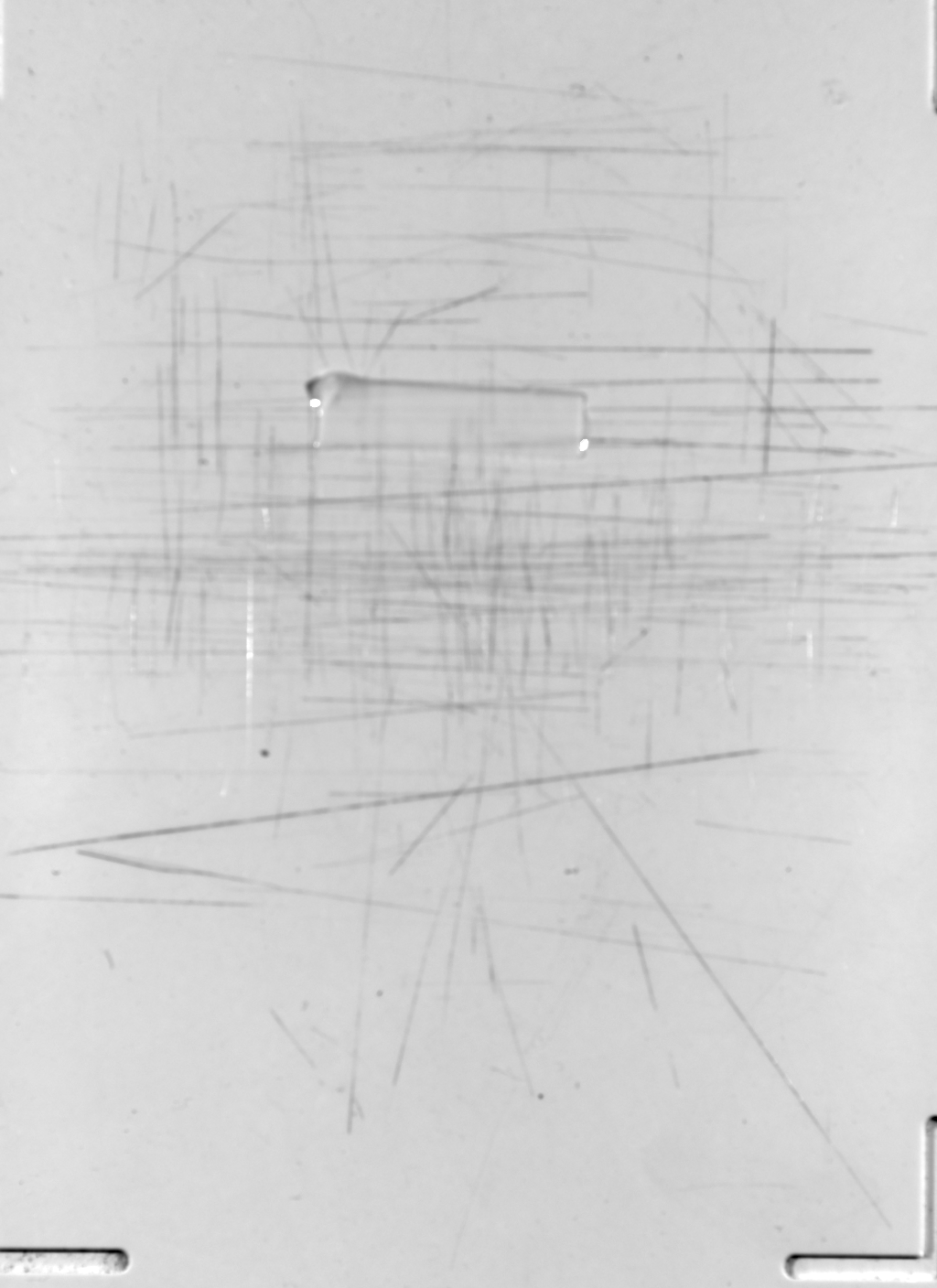

Supplement: Supplemental Information 9 [file peerj-11-15041-s009.zip › Transcriptome-related genes-raw data2/SPARC/SPARC-3/SPARC-3-4.tif]

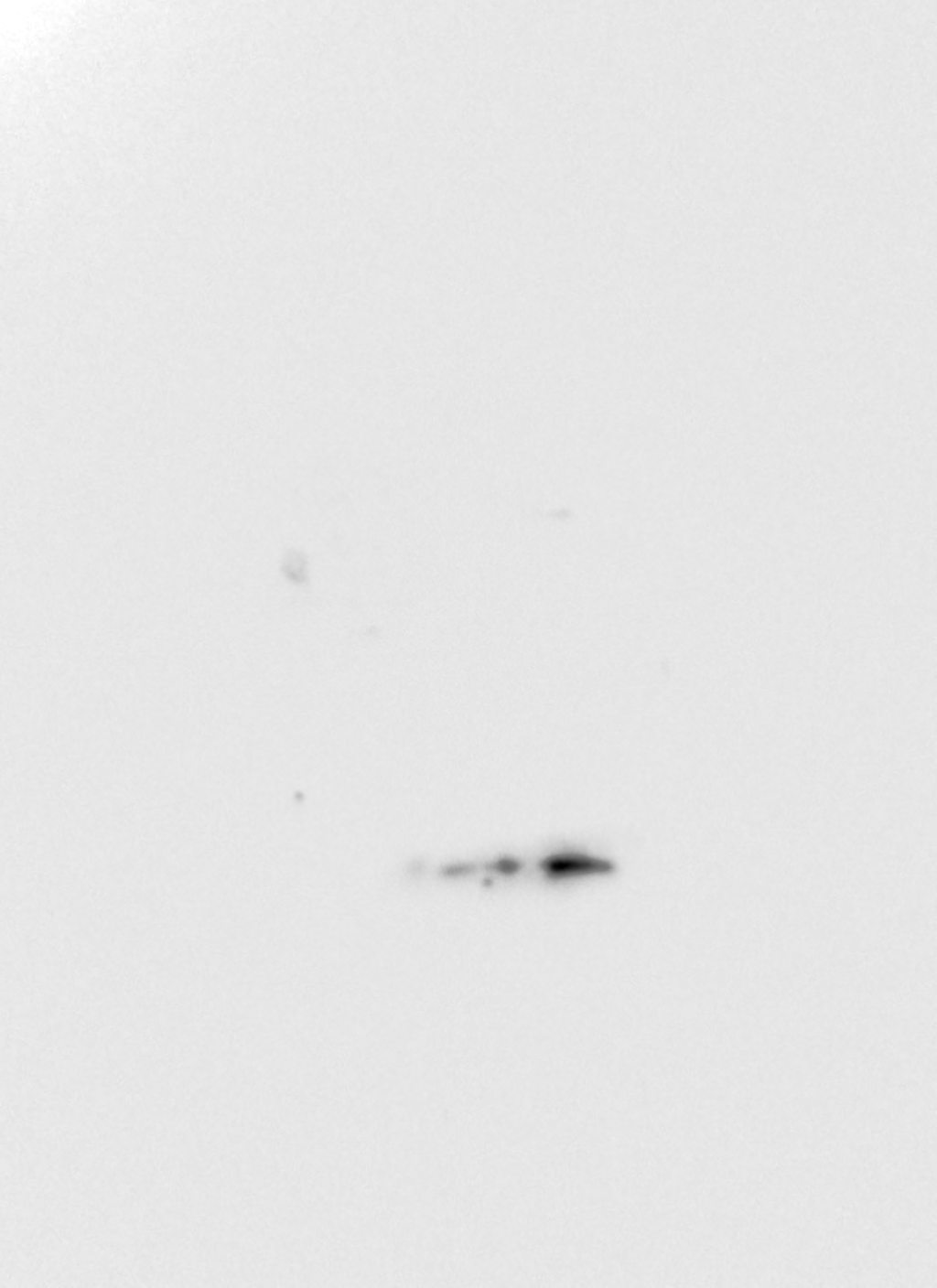

Supplement: Supplemental Information 10 [file peerj-11-15041-s010.zip › Transcriptome-related genes-raw data3/COL1A2/COL1A2-1/COL1A2-1-1.tif]

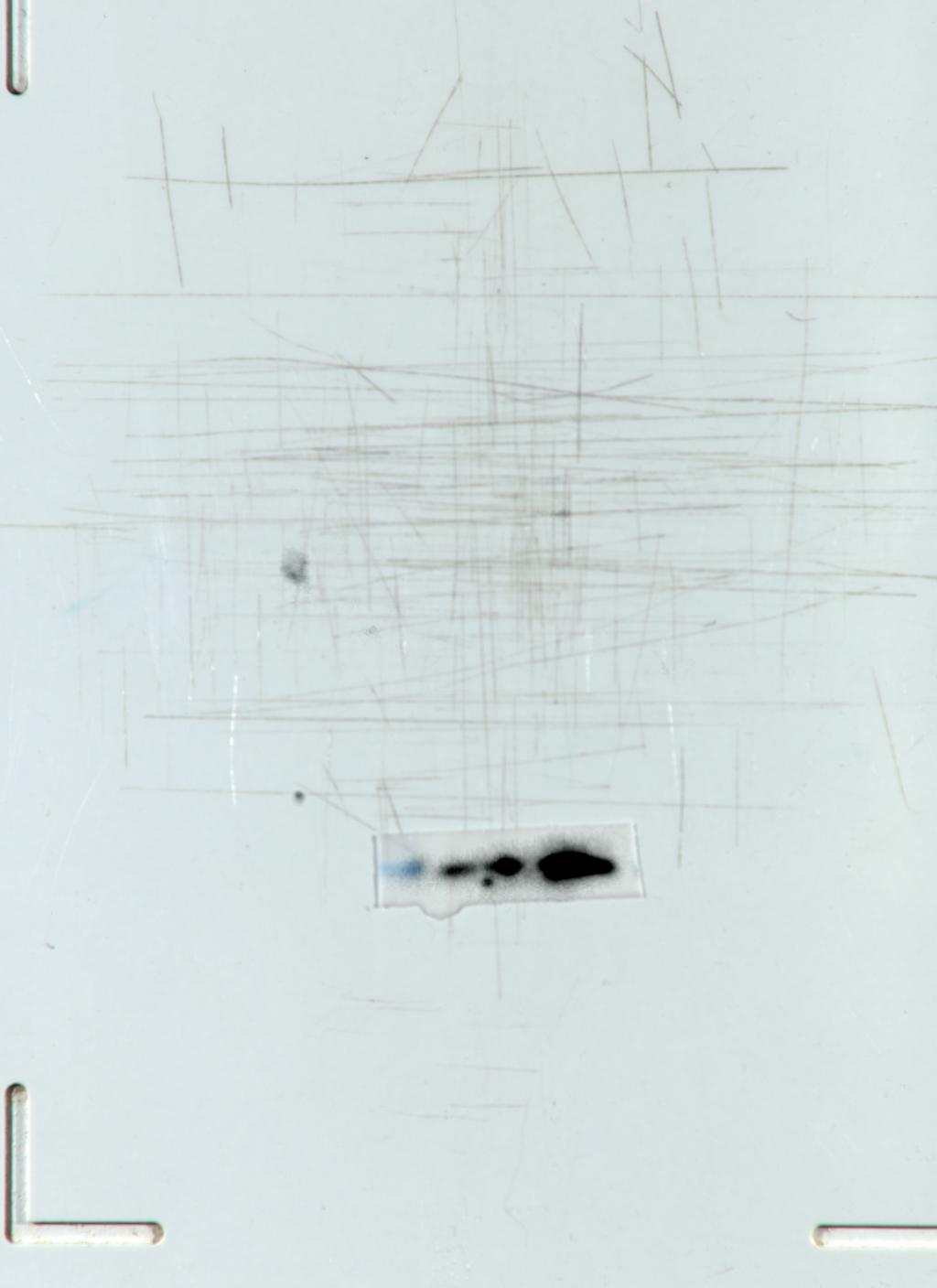

Supplement: Supplemental Information 10 [file peerj-11-15041-s010.zip › Transcriptome-related genes-raw data3/COL1A2/COL1A2-1/COL1A2-1-2.jpg]

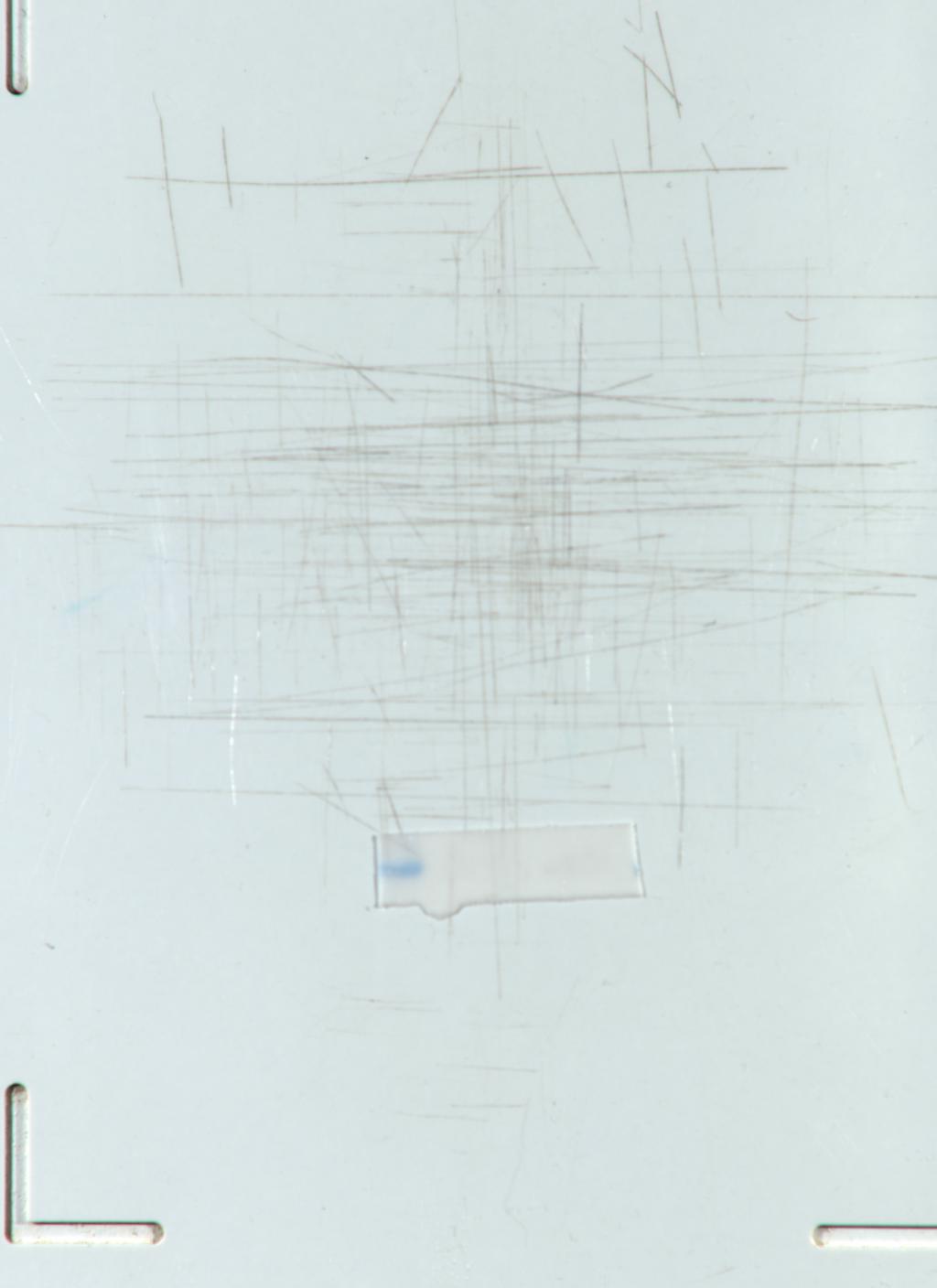

Supplement: Supplemental Information 10 [file peerj-11-15041-s010.zip › Transcriptome-related genes-raw data3/COL1A2/COL1A2-1/COL1A2-1-3.jpg]

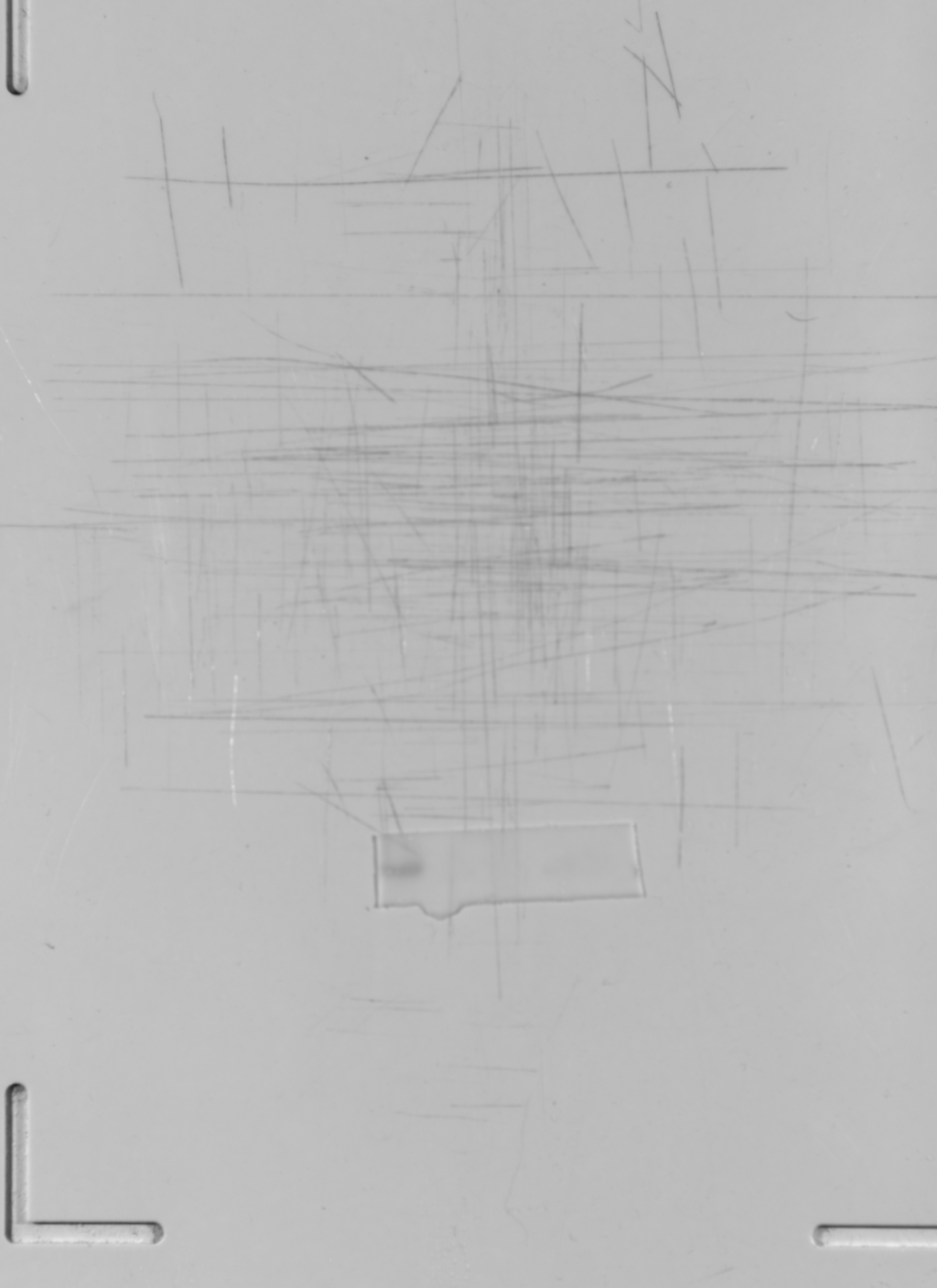

Supplement: Supplemental Information 10 [file peerj-11-15041-s010.zip › Transcriptome-related genes-raw data3/COL1A2/COL1A2-1/COL1A2-1-4.tif]

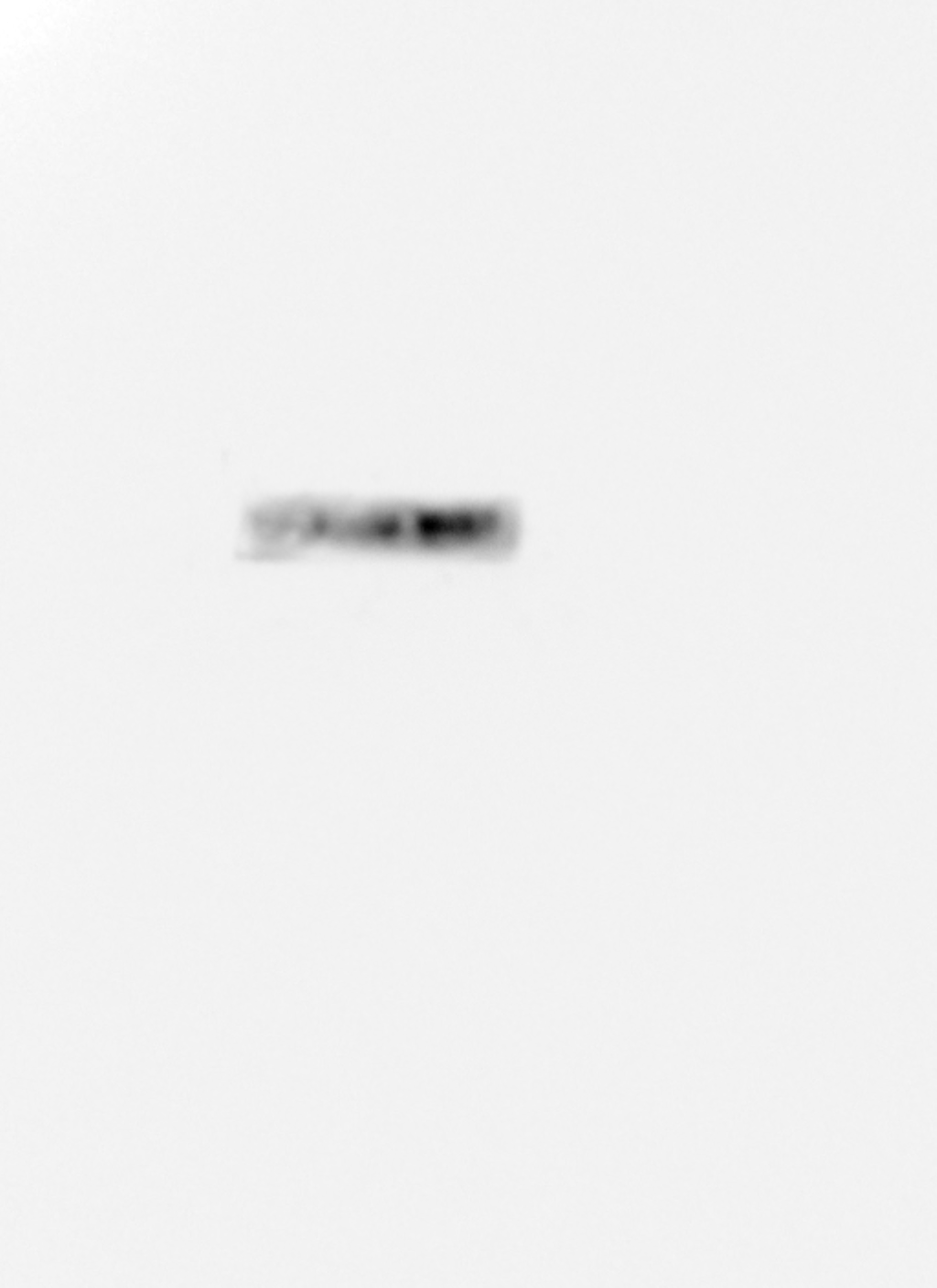

Supplement: Supplemental Information 10 [file peerj-11-15041-s010.zip › Transcriptome-related genes-raw data3/COL1A2/COL1A2-2/COL1A2-2-1.tif]

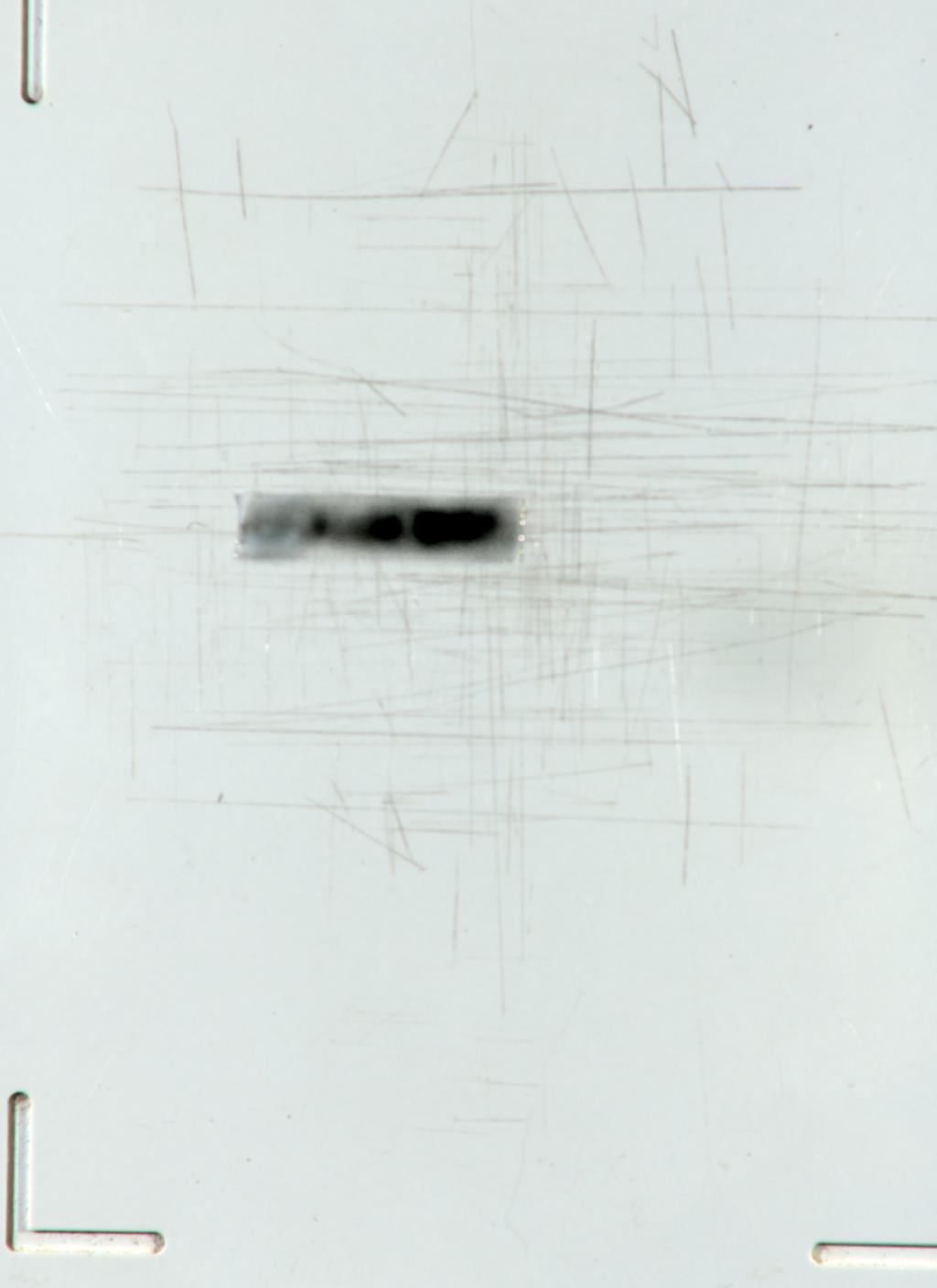

Supplement: Supplemental Information 10 [file peerj-11-15041-s010.zip › Transcriptome-related genes-raw data3/COL1A2/COL1A2-2/COL1A2-2-2.jpg]

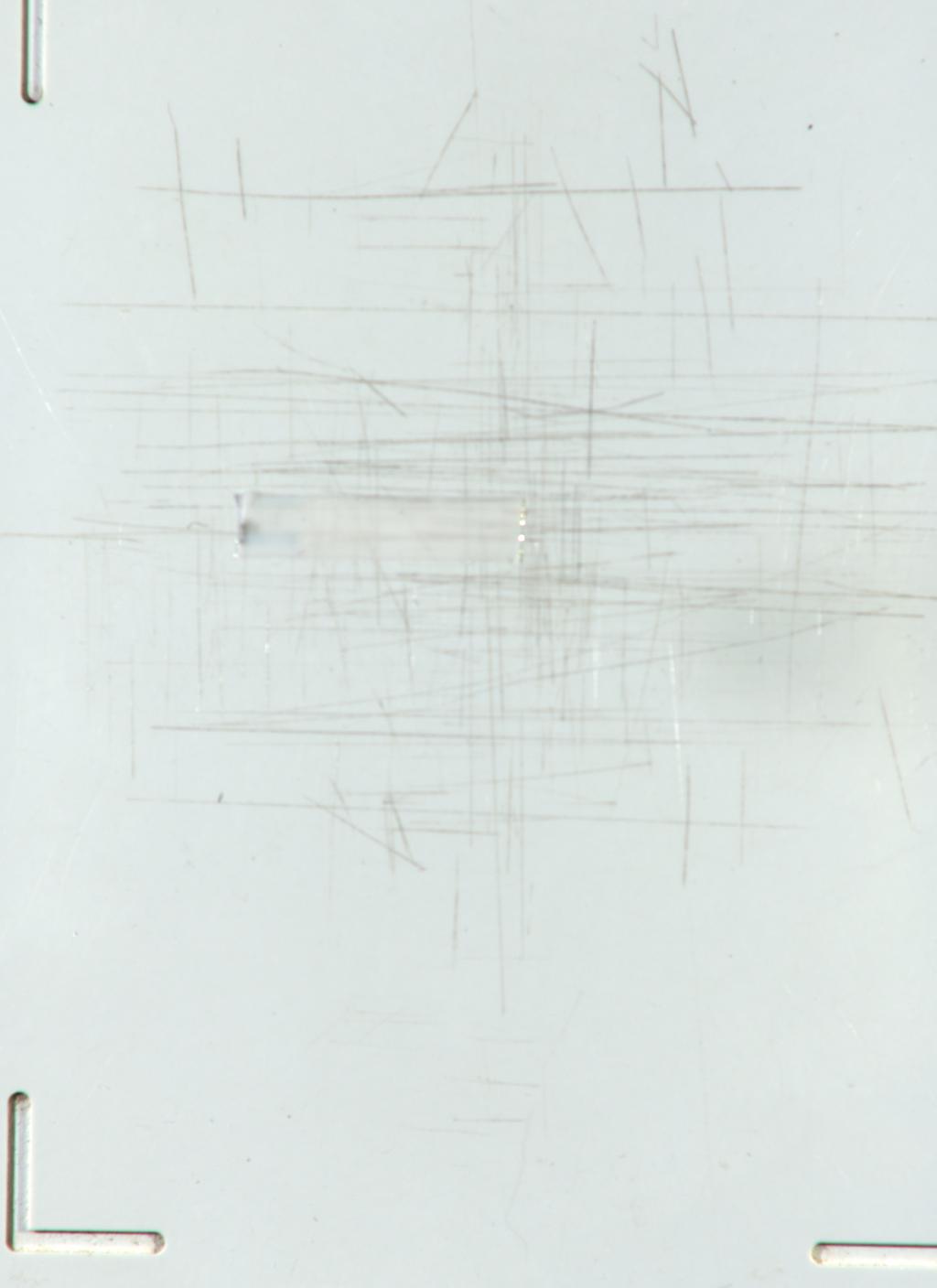

Supplement: Supplemental Information 10 [file peerj-11-15041-s010.zip › Transcriptome-related genes-raw data3/COL1A2/COL1A2-2/COL1A2-2-3.jpg]

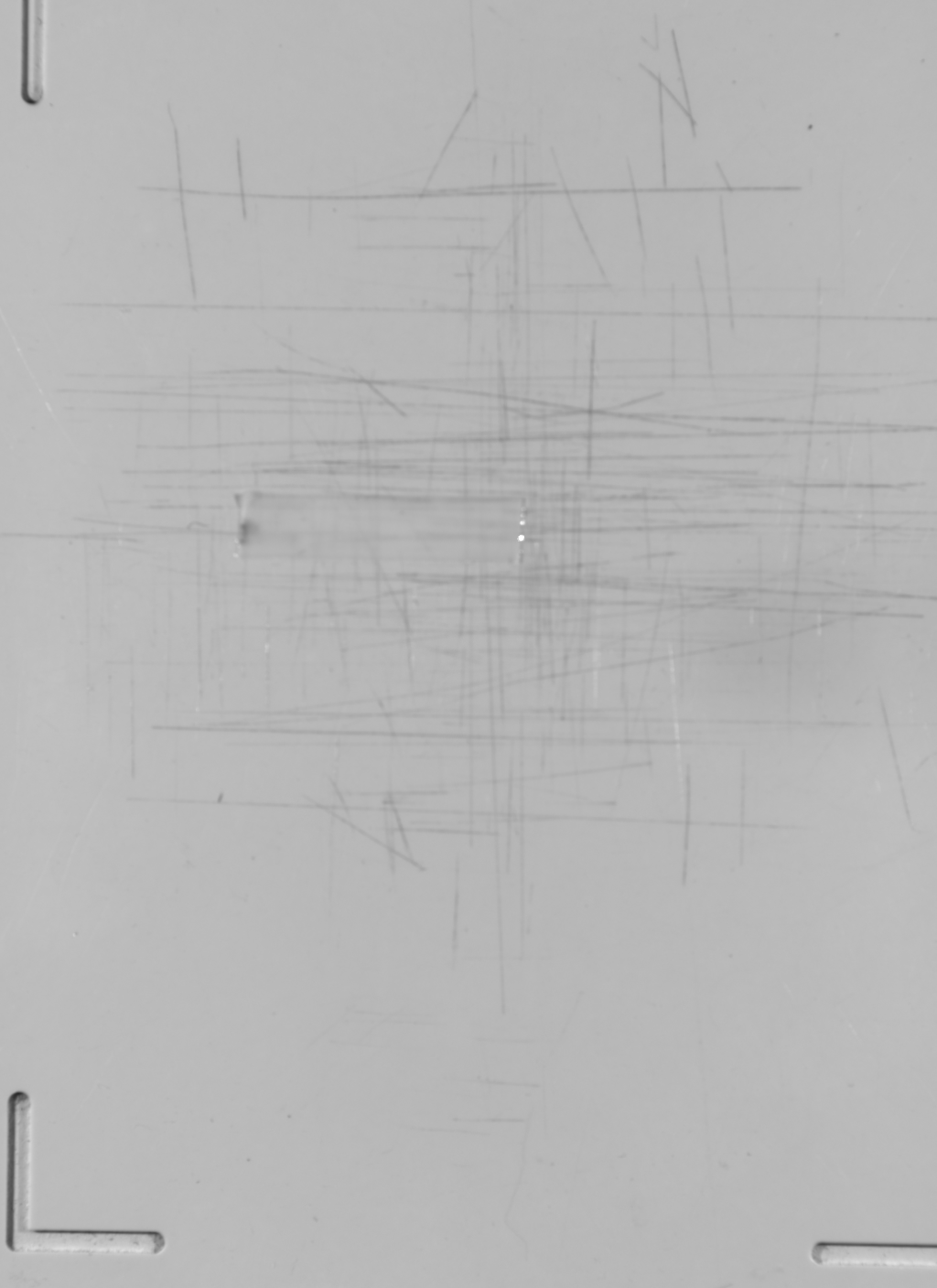

Supplement: Supplemental Information 10 [file peerj-11-15041-s010.zip › Transcriptome-related genes-raw data3/COL1A2/COL1A2-2/COL1A2-2-4.tif]

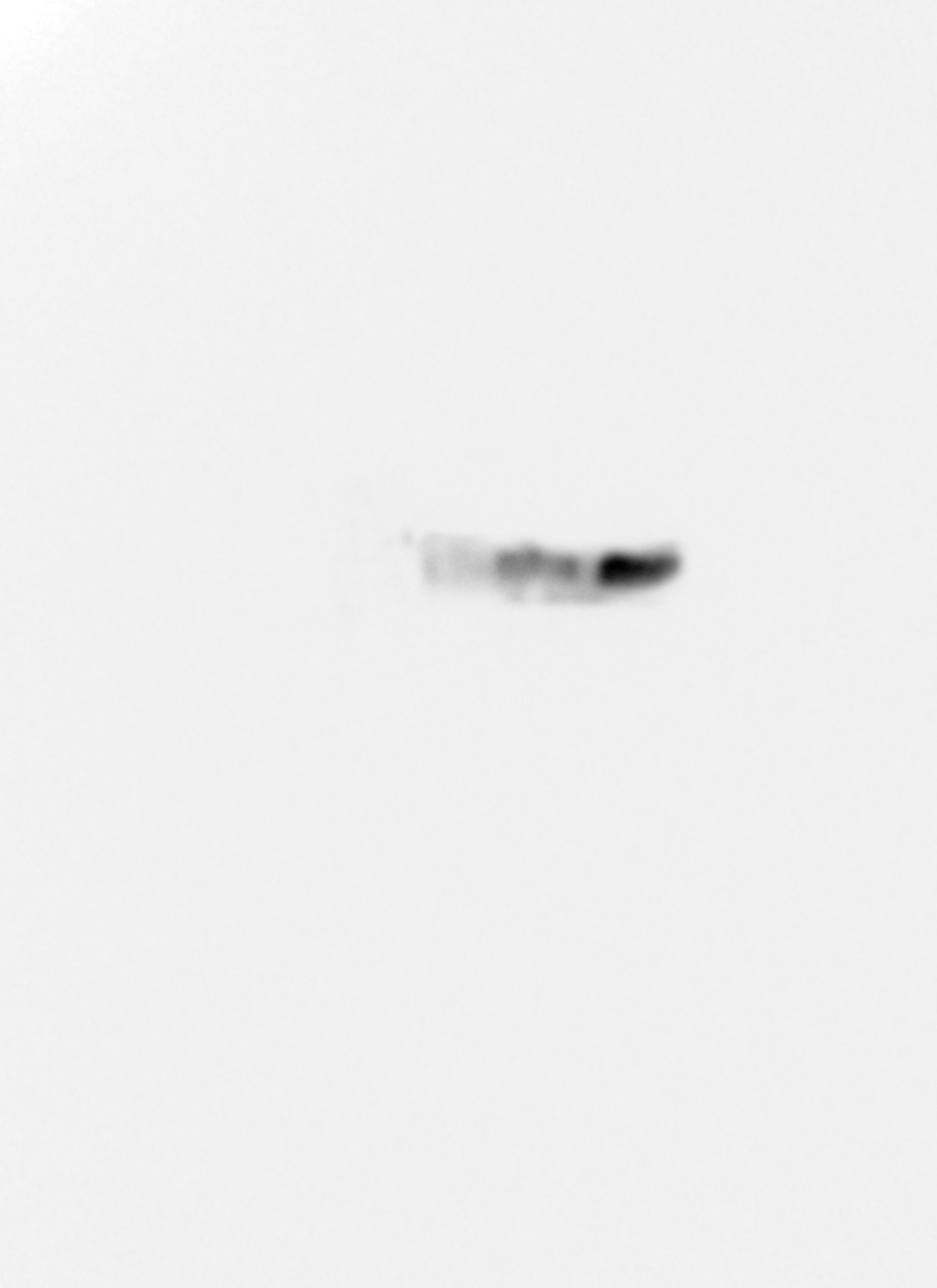

Supplement: Supplemental Information 10 [file peerj-11-15041-s010.zip › Transcriptome-related genes-raw data3/COL1A2/COL1A2-3/COL1A2-3-1.tif]

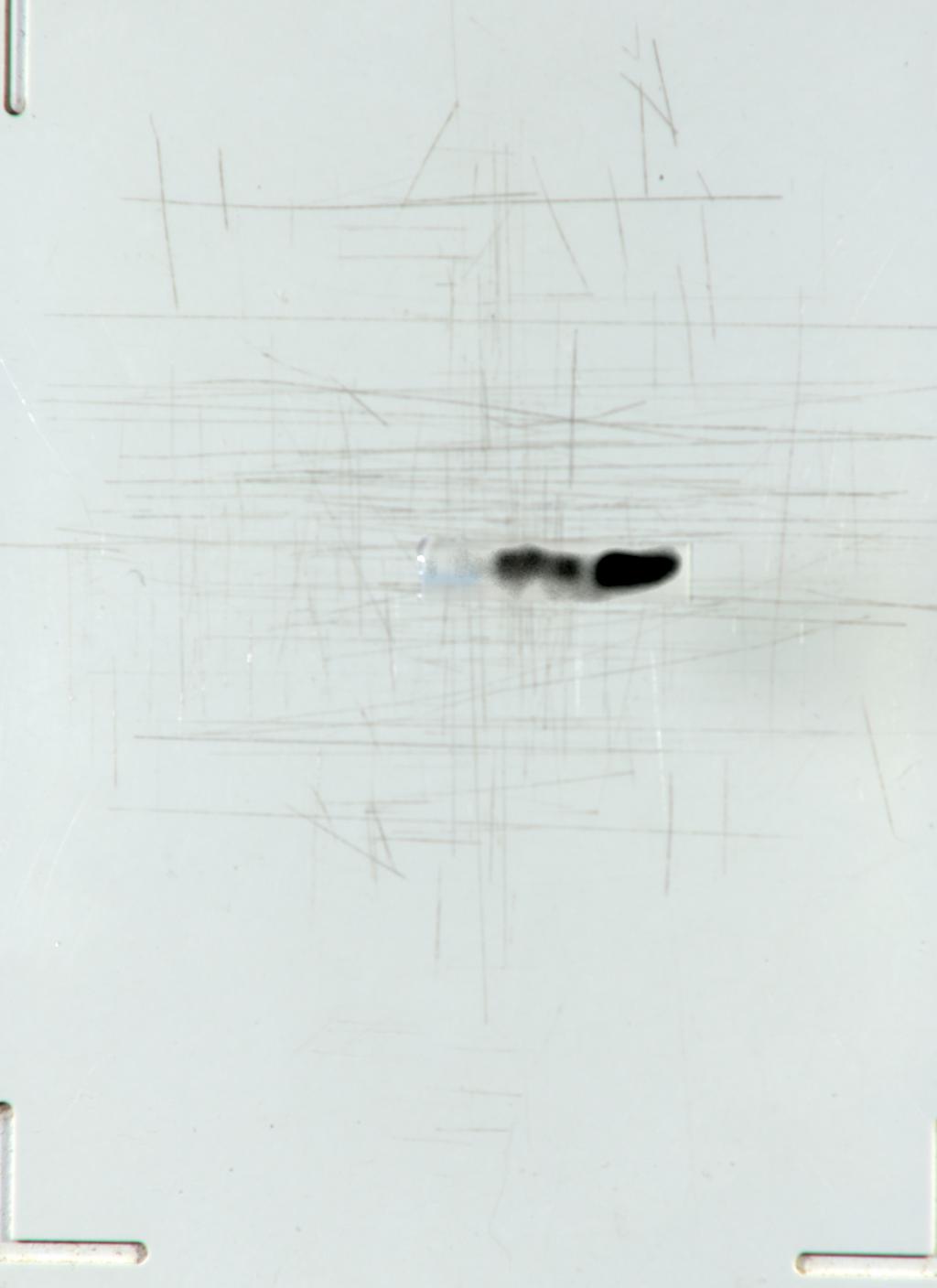

Supplement: Supplemental Information 10 [file peerj-11-15041-s010.zip › Transcriptome-related genes-raw data3/COL1A2/COL1A2-3/COL1A2-3-2.jpg]

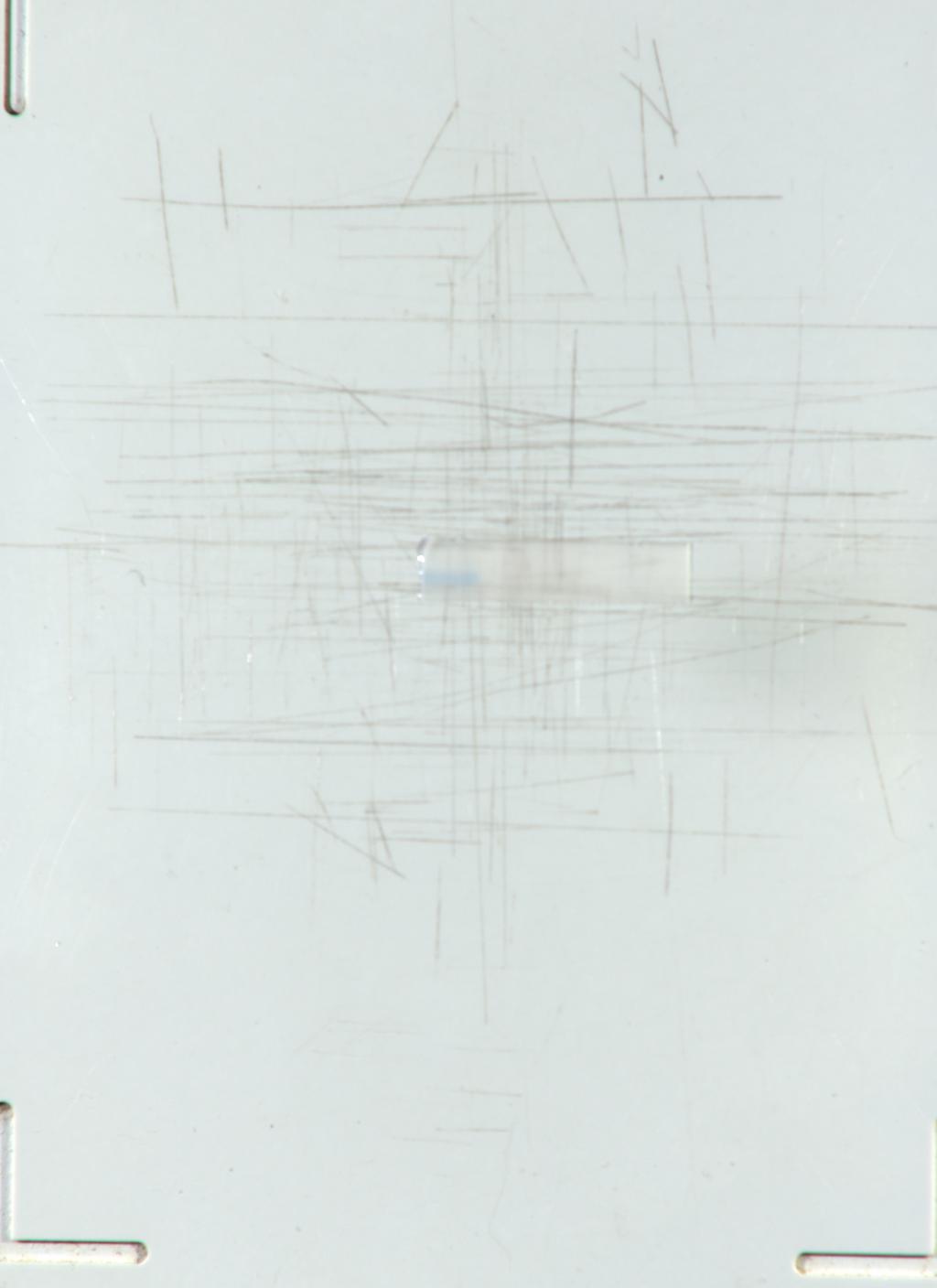

Supplement: Supplemental Information 10 [file peerj-11-15041-s010.zip › Transcriptome-related genes-raw data3/COL1A2/COL1A2-3/COL1A2-3-3.jpg]

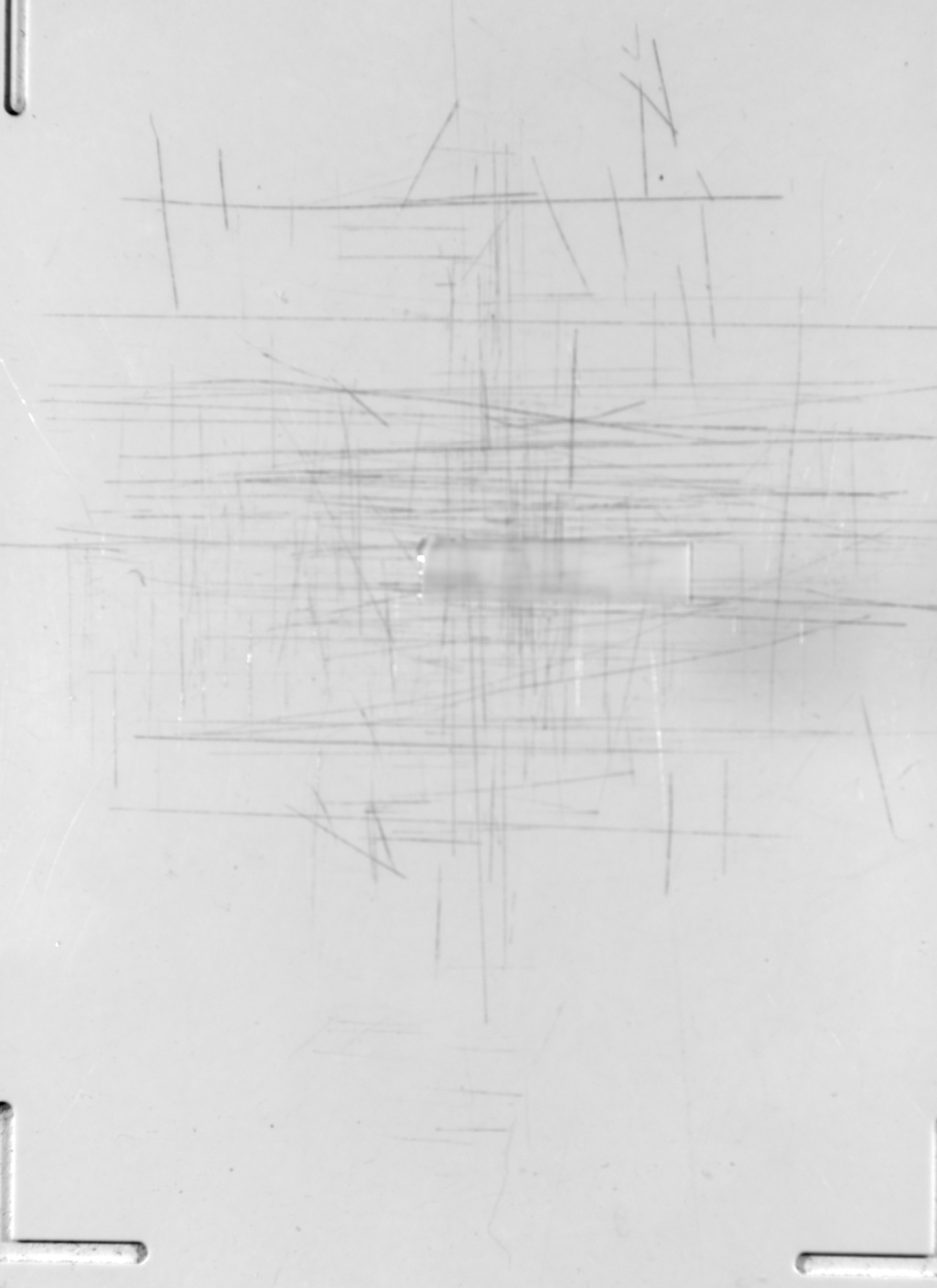

Supplement: Supplemental Information 10 [file peerj-11-15041-s010.zip › Transcriptome-related genes-raw data3/COL1A2/COL1A2-3/COL1A2-3-4.tif]

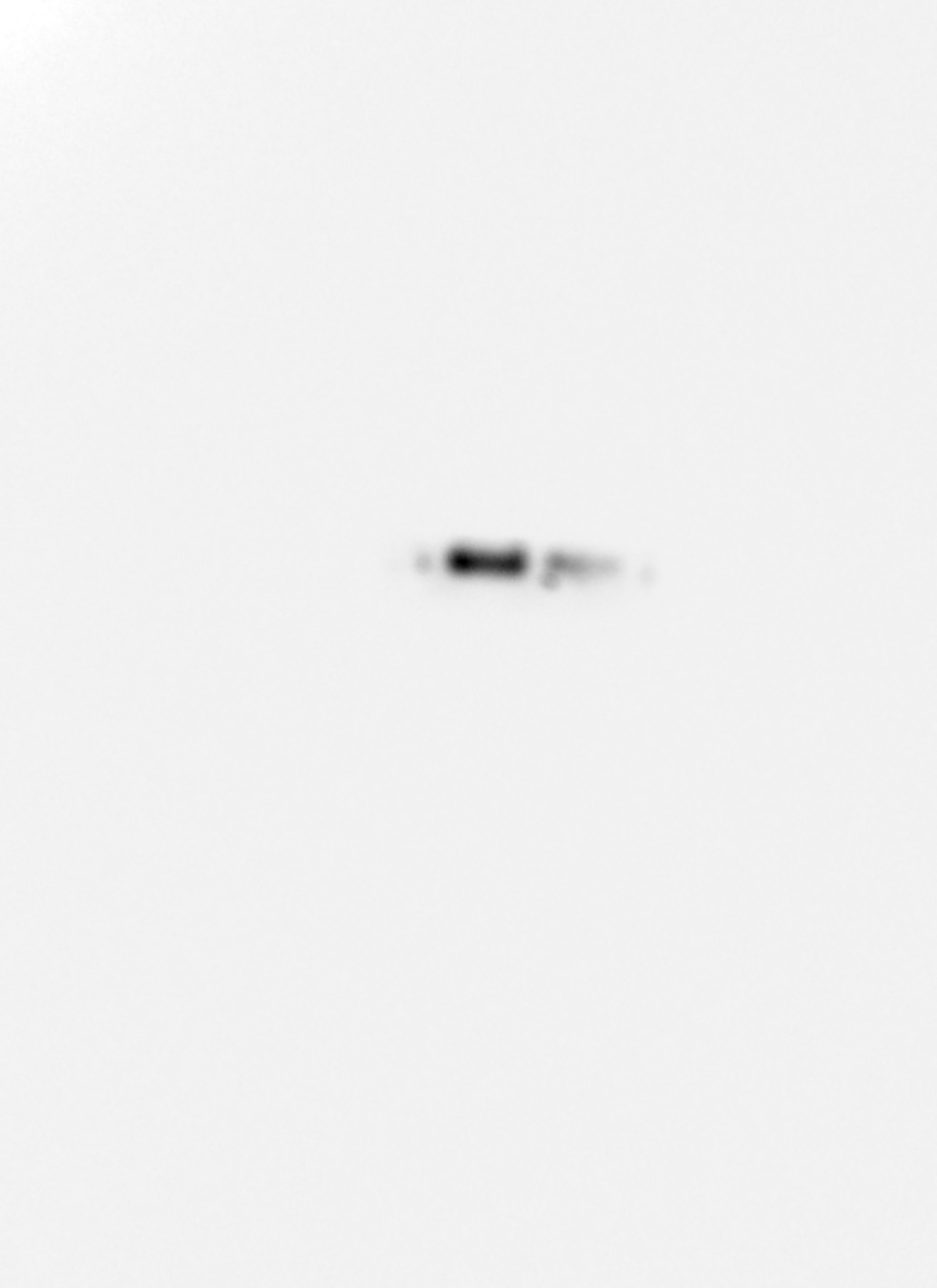

Supplement: Supplemental Information 10 [file peerj-11-15041-s010.zip › Transcriptome-related genes-raw data3/CYP27A1/CYP27A1-1/CYP27A1-1-1.tif]

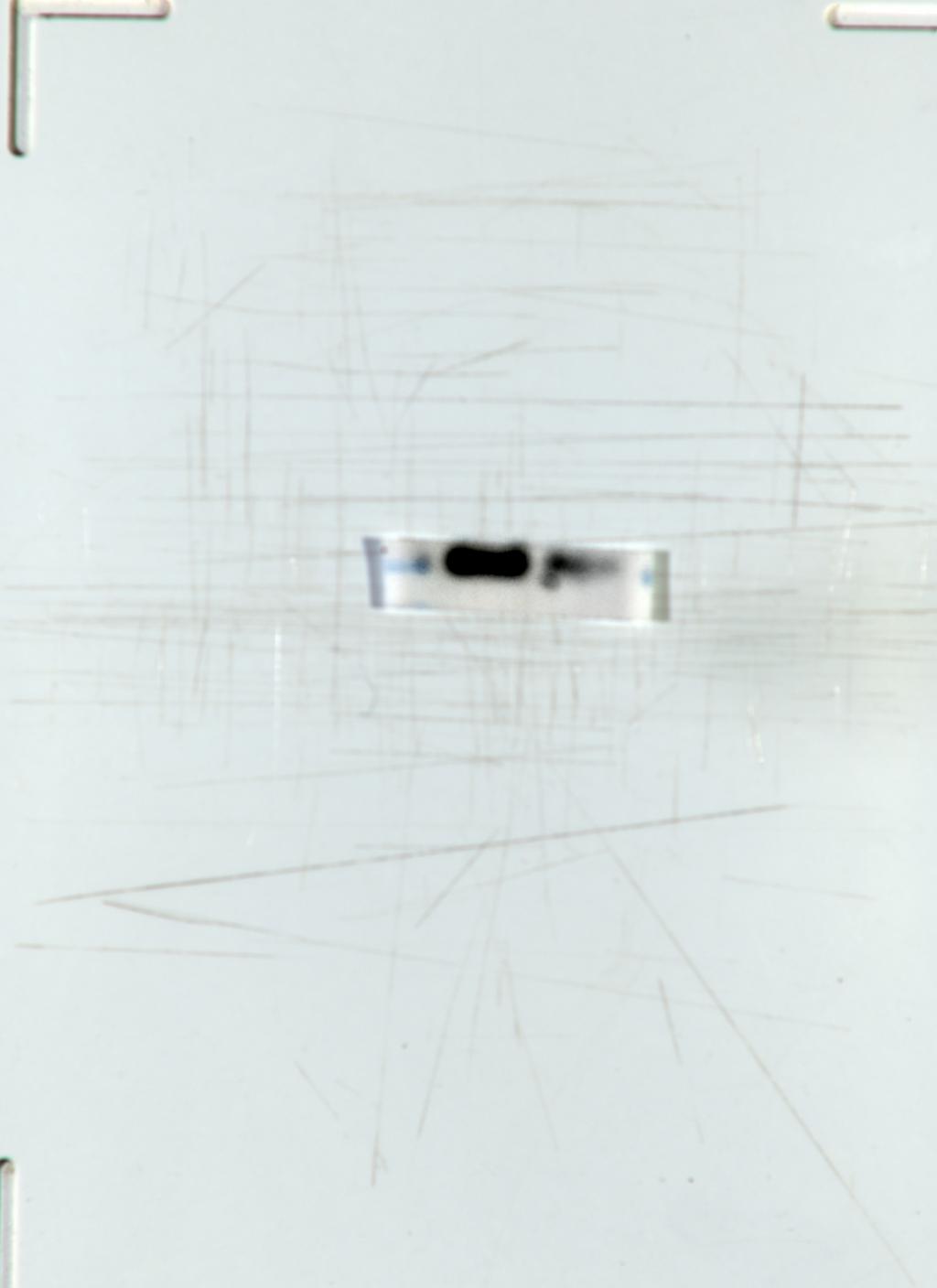

Supplement: Supplemental Information 10 [file peerj-11-15041-s010.zip › Transcriptome-related genes-raw data3/CYP27A1/CYP27A1-1/CYP27A1-1-2.jpg]

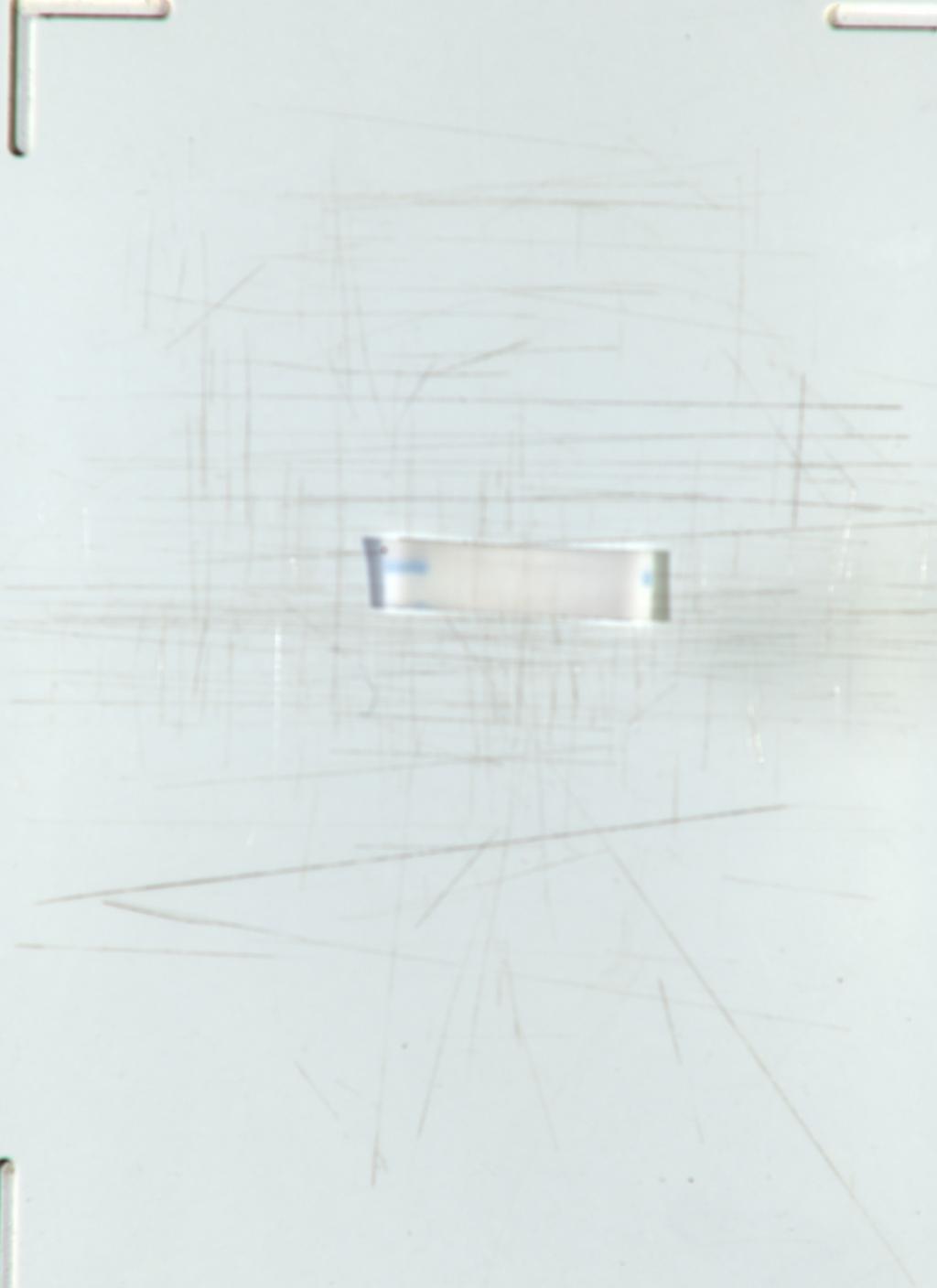

Supplement: Supplemental Information 10 [file peerj-11-15041-s010.zip › Transcriptome-related genes-raw data3/CYP27A1/CYP27A1-1/CYP27A1-1-3.jpg]

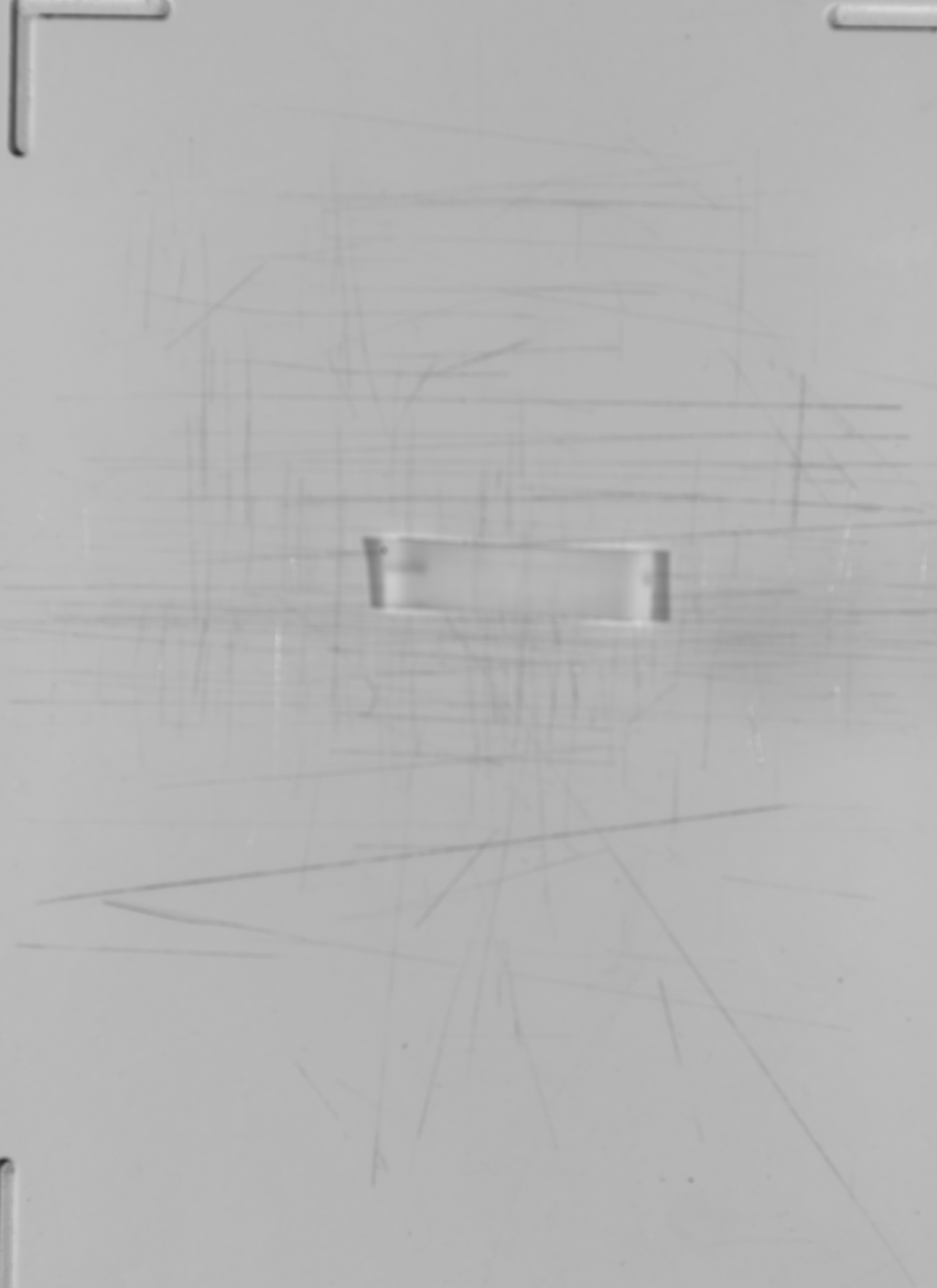

Supplement: Supplemental Information 10 [file peerj-11-15041-s010.zip › Transcriptome-related genes-raw data3/CYP27A1/CYP27A1-1/CYP27A1-1-4.tif]

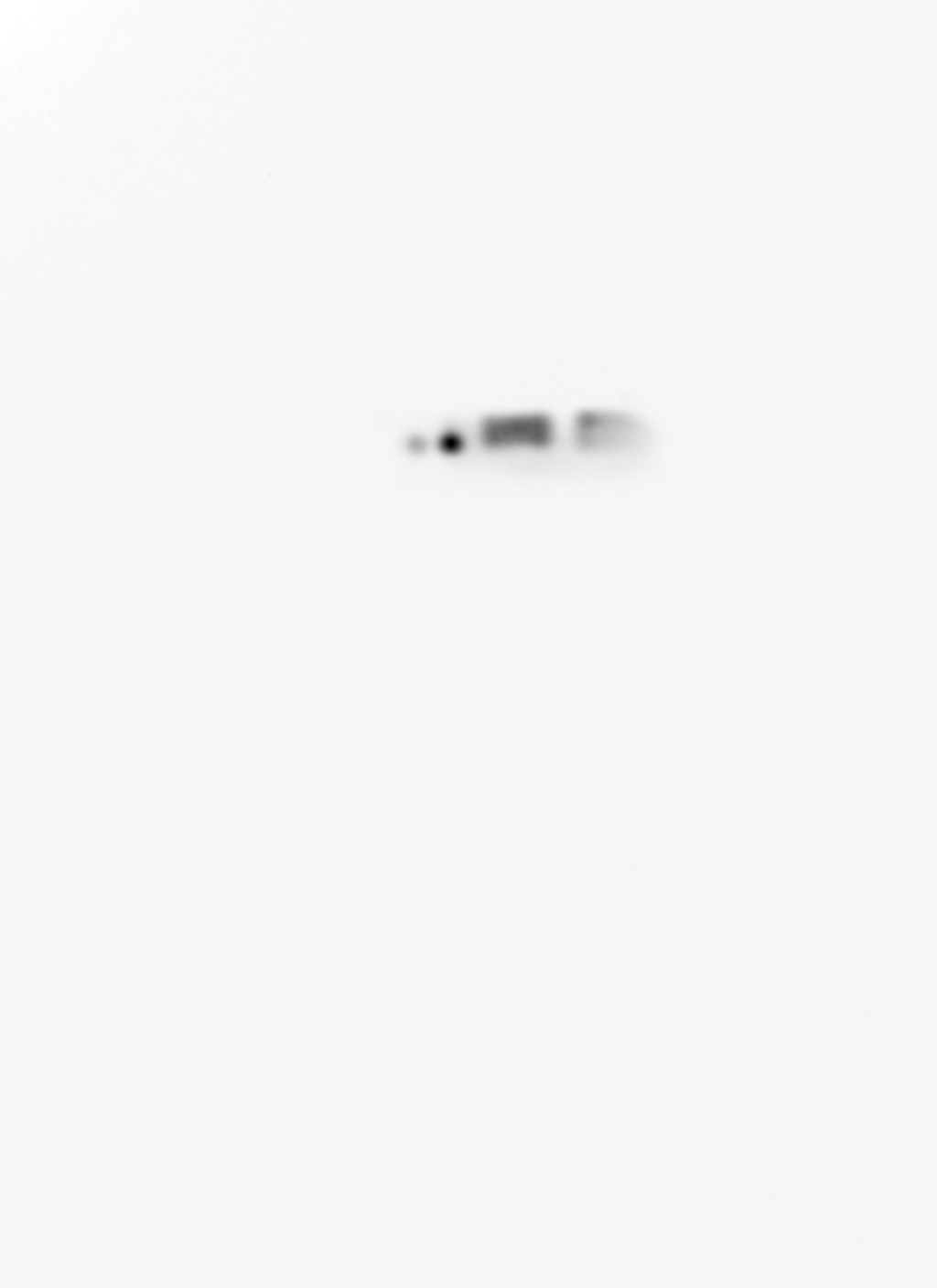

Supplement: Supplemental Information 10 [file peerj-11-15041-s010.zip › Transcriptome-related genes-raw data3/CYP27A1/CYP27A1-2/CYP27A1-2-1.tif]

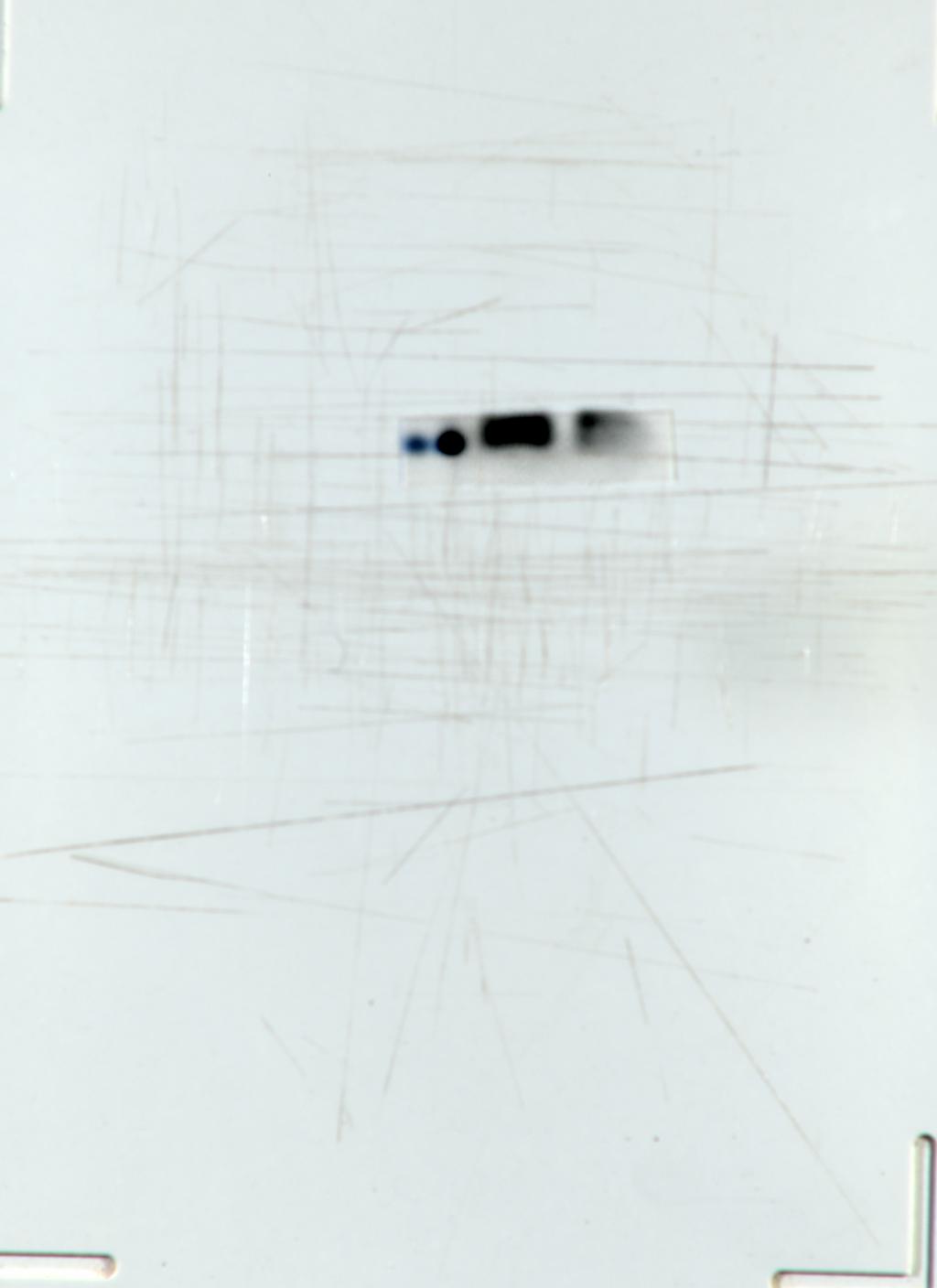

Supplement: Supplemental Information 10 [file peerj-11-15041-s010.zip › Transcriptome-related genes-raw data3/CYP27A1/CYP27A1-2/CYP27A1-2-2.jpg]

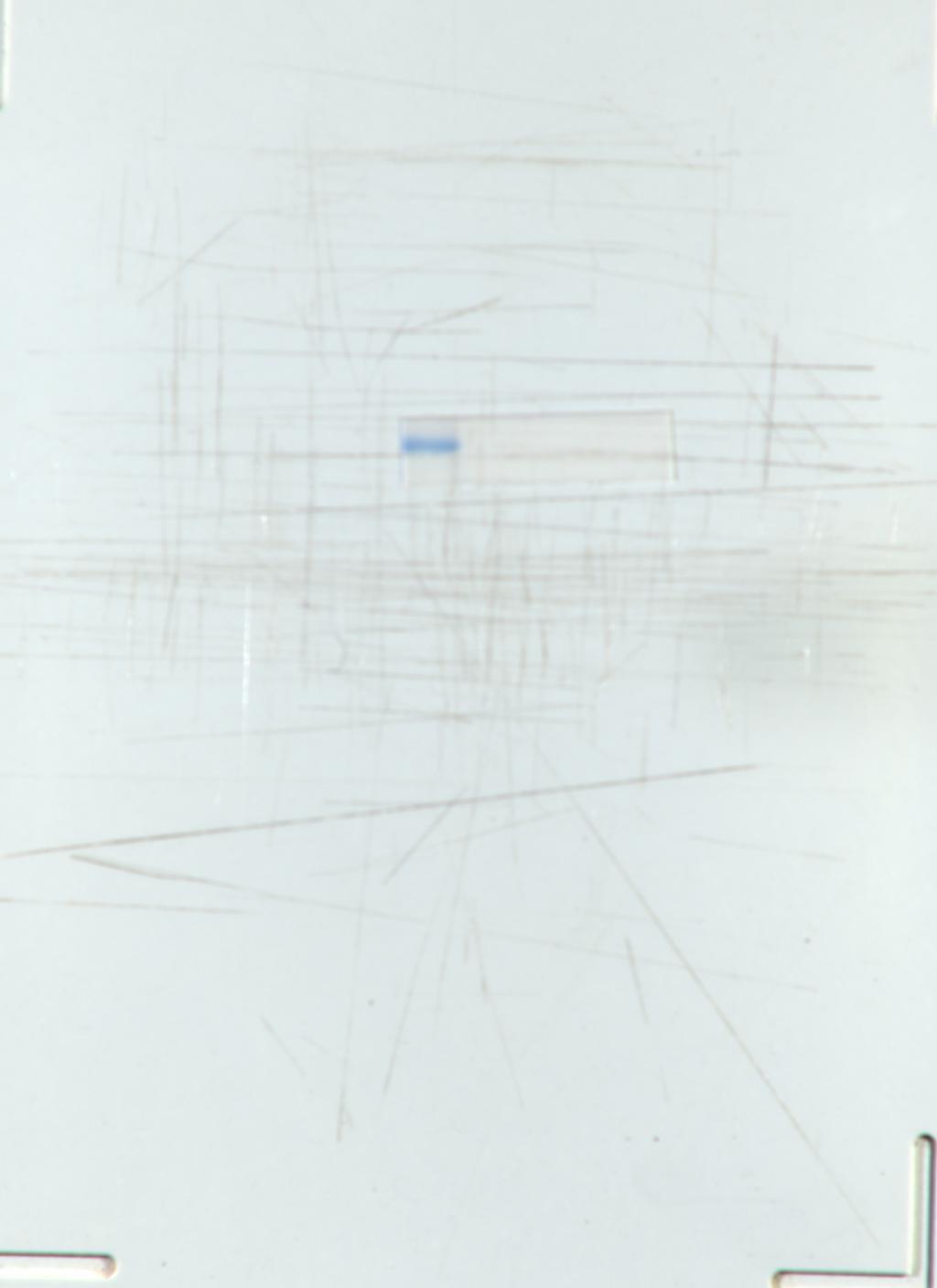

Supplement: Supplemental Information 10 [file peerj-11-15041-s010.zip › Transcriptome-related genes-raw data3/CYP27A1/CYP27A1-2/CYP27A1-2-3.jpg]

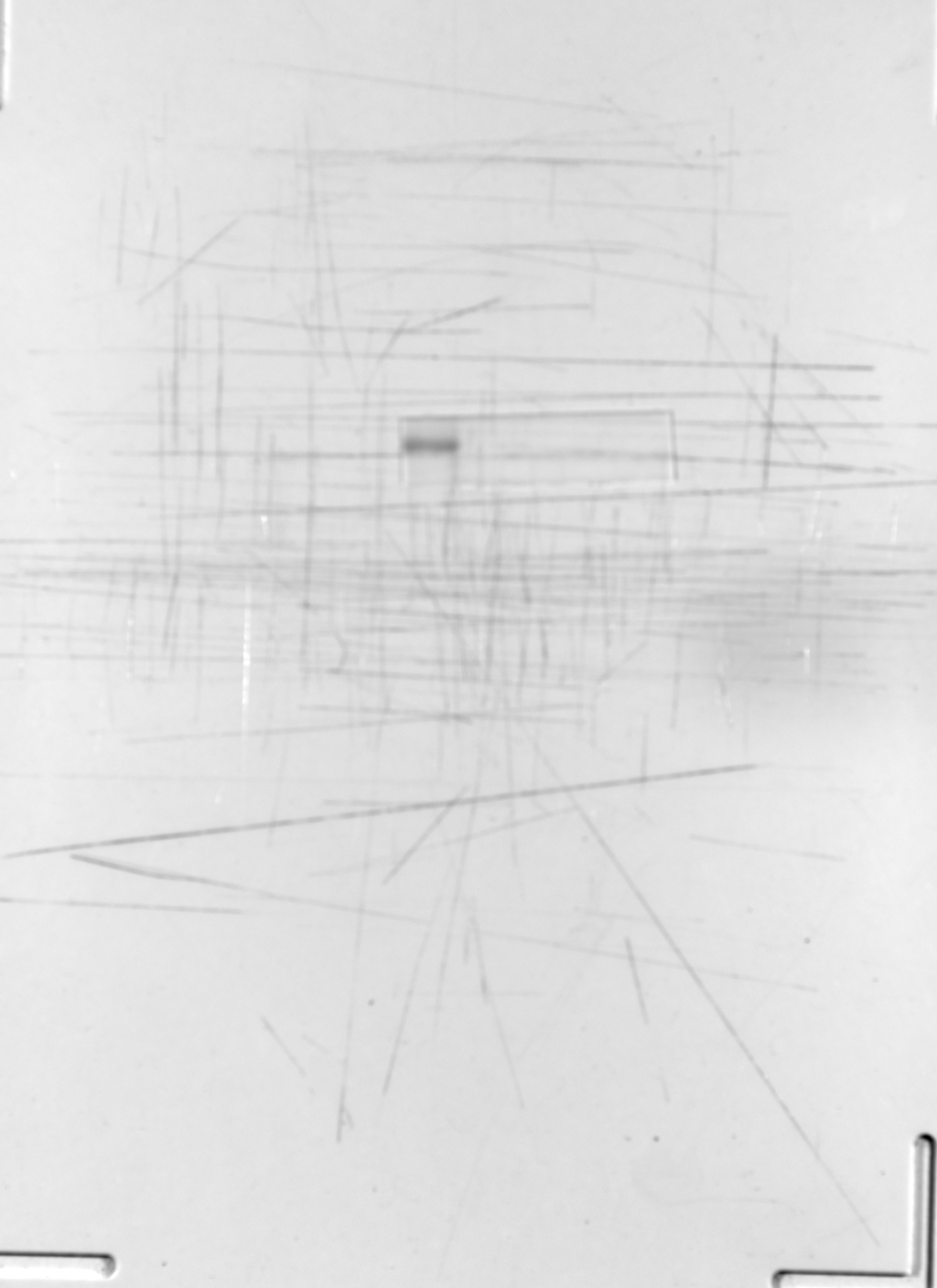

Supplement: Supplemental Information 10 [file peerj-11-15041-s010.zip › Transcriptome-related genes-raw data3/CYP27A1/CYP27A1-2/CYP27A1-2-4.tif]

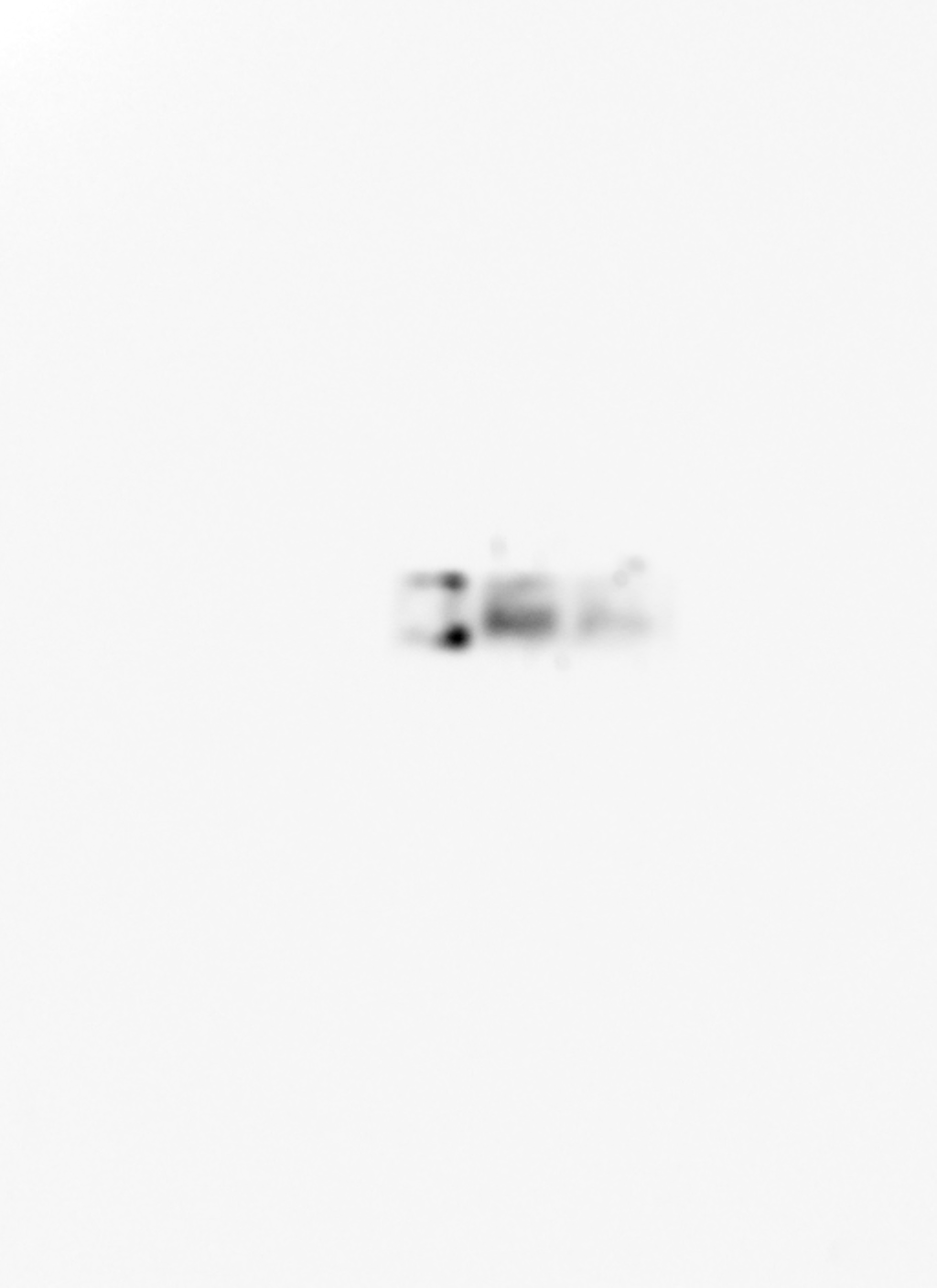

Supplement: Supplemental Information 10 [file peerj-11-15041-s010.zip › Transcriptome-related genes-raw data3/CYP27A1/CYP27A1-3/CYP27A1-3-1.tif]

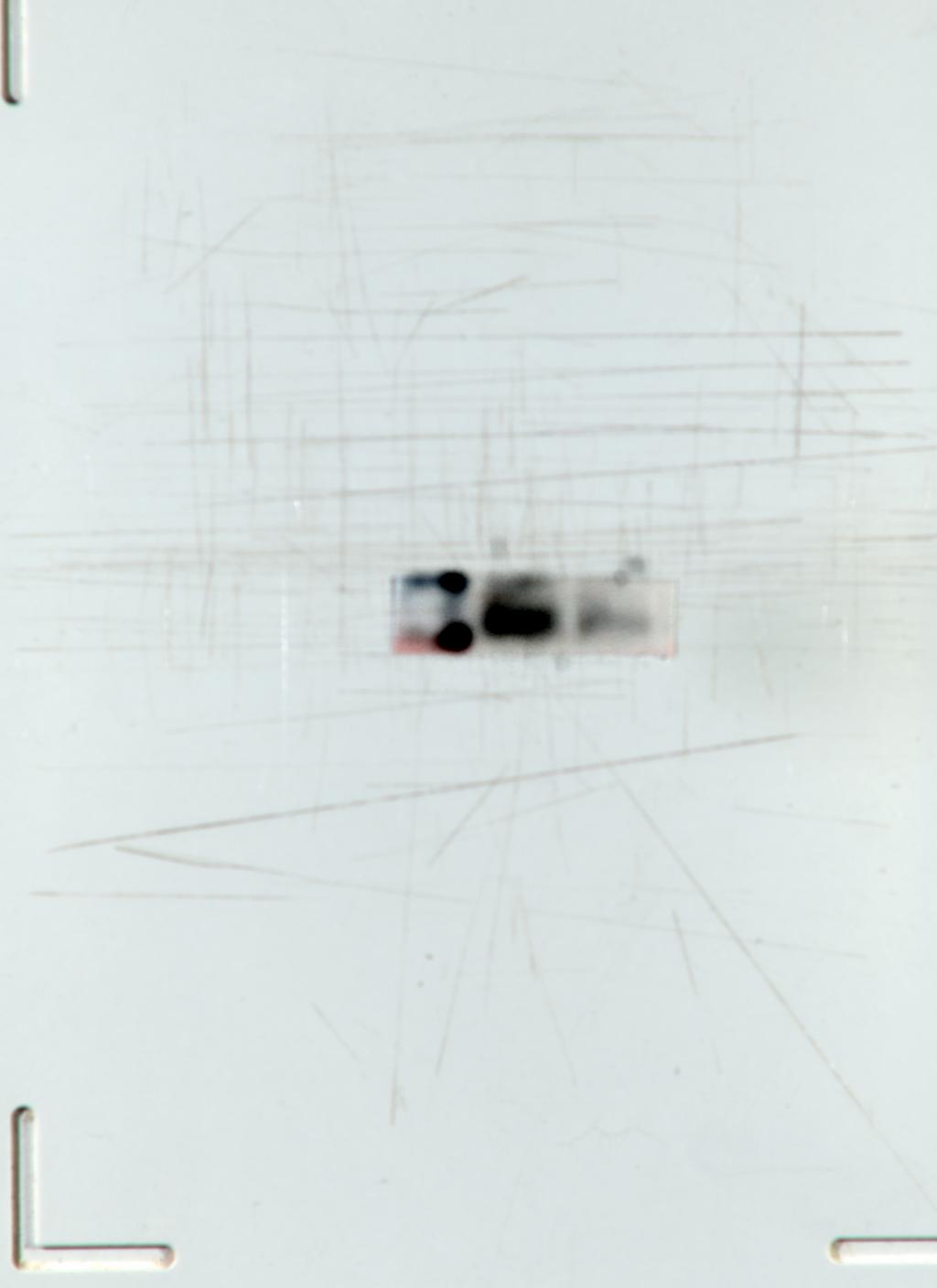

Supplement: Supplemental Information 10 [file peerj-11-15041-s010.zip › Transcriptome-related genes-raw data3/CYP27A1/CYP27A1-3/CYP27A1-3-2.jpg]

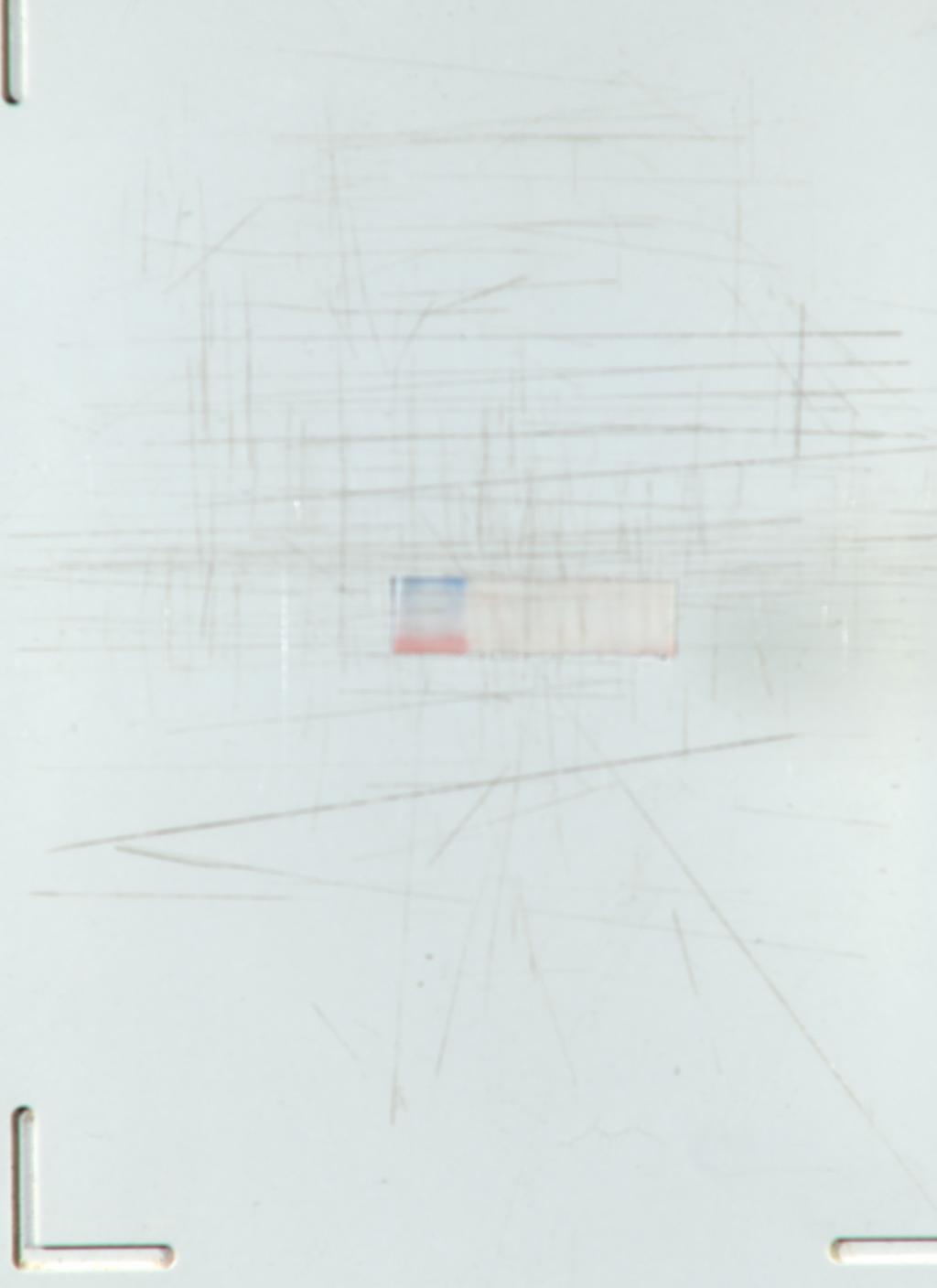

Supplement: Supplemental Information 10 [file peerj-11-15041-s010.zip › Transcriptome-related genes-raw data3/CYP27A1/CYP27A1-3/CYP27A1-3-3.jpg]

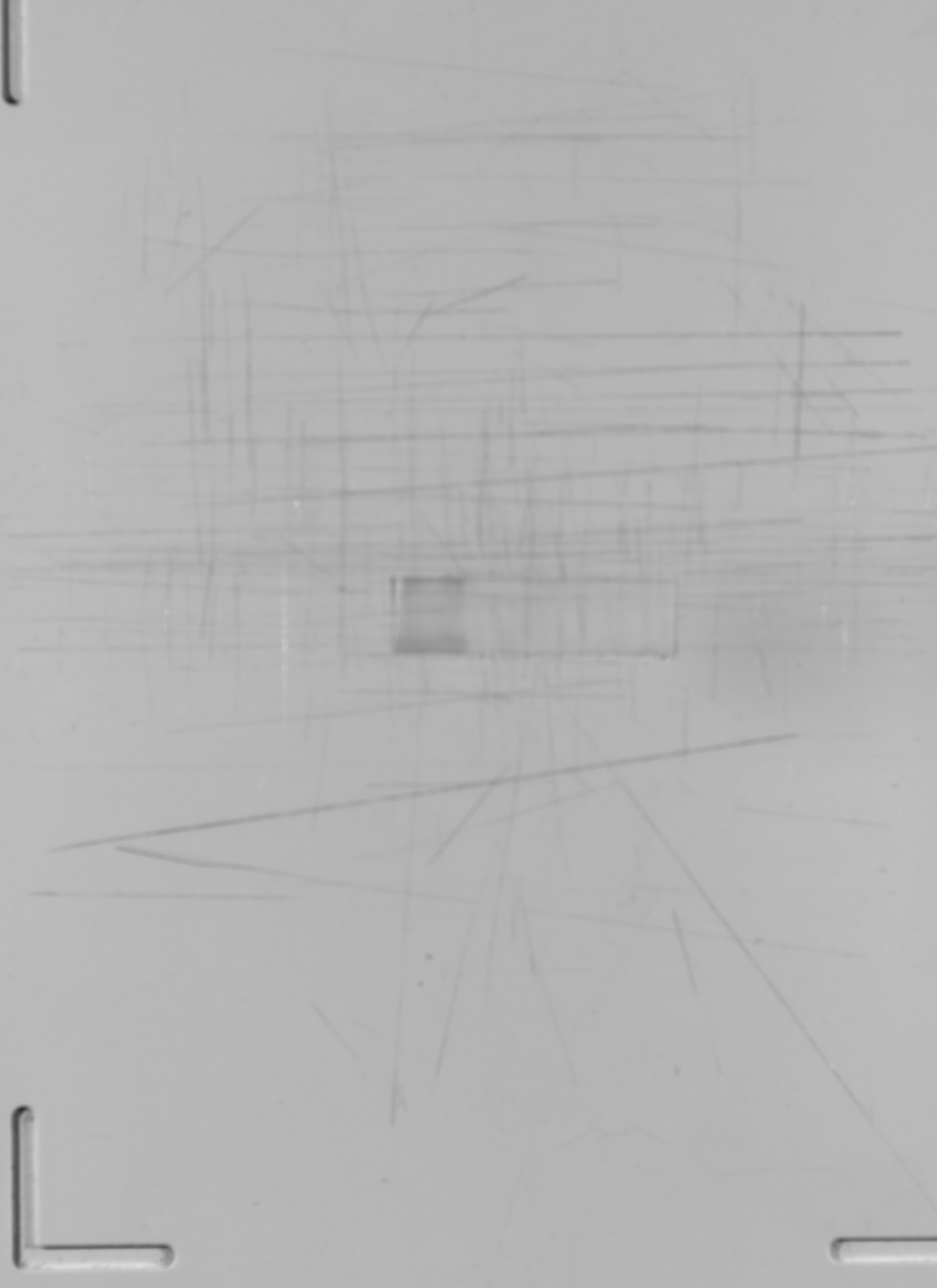

Supplement: Supplemental Information 10 [file peerj-11-15041-s010.zip › Transcriptome-related genes-raw data3/CYP27A1/CYP27A1-3/CYP27A1-3-4.tif]

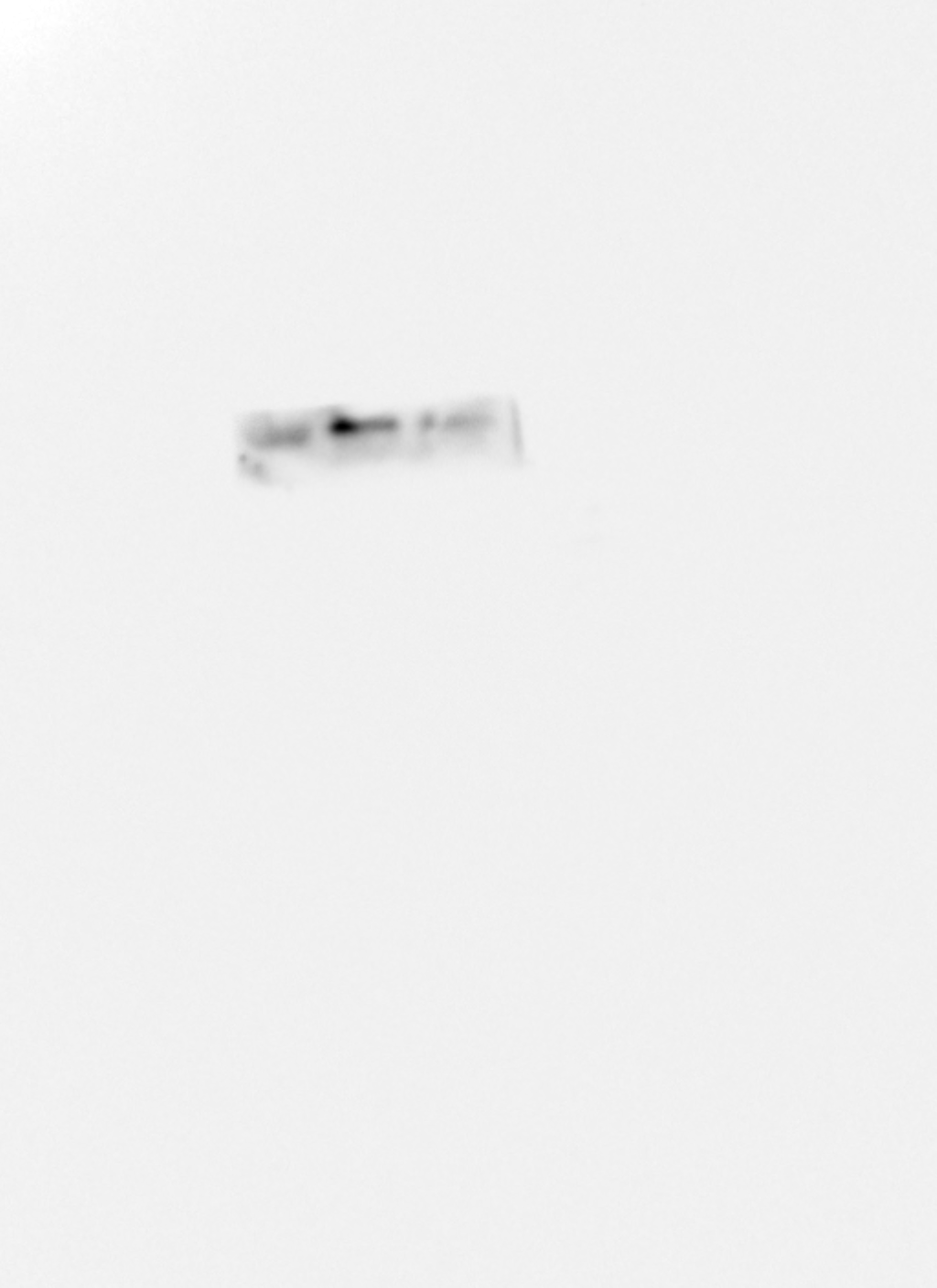

Supplement: Supplemental Information 11 [file peerj-11-15041-s011.zip › Transcriptome-related genes-raw data4/ELANE/ELANE-1/ELANE-1-1.tif]

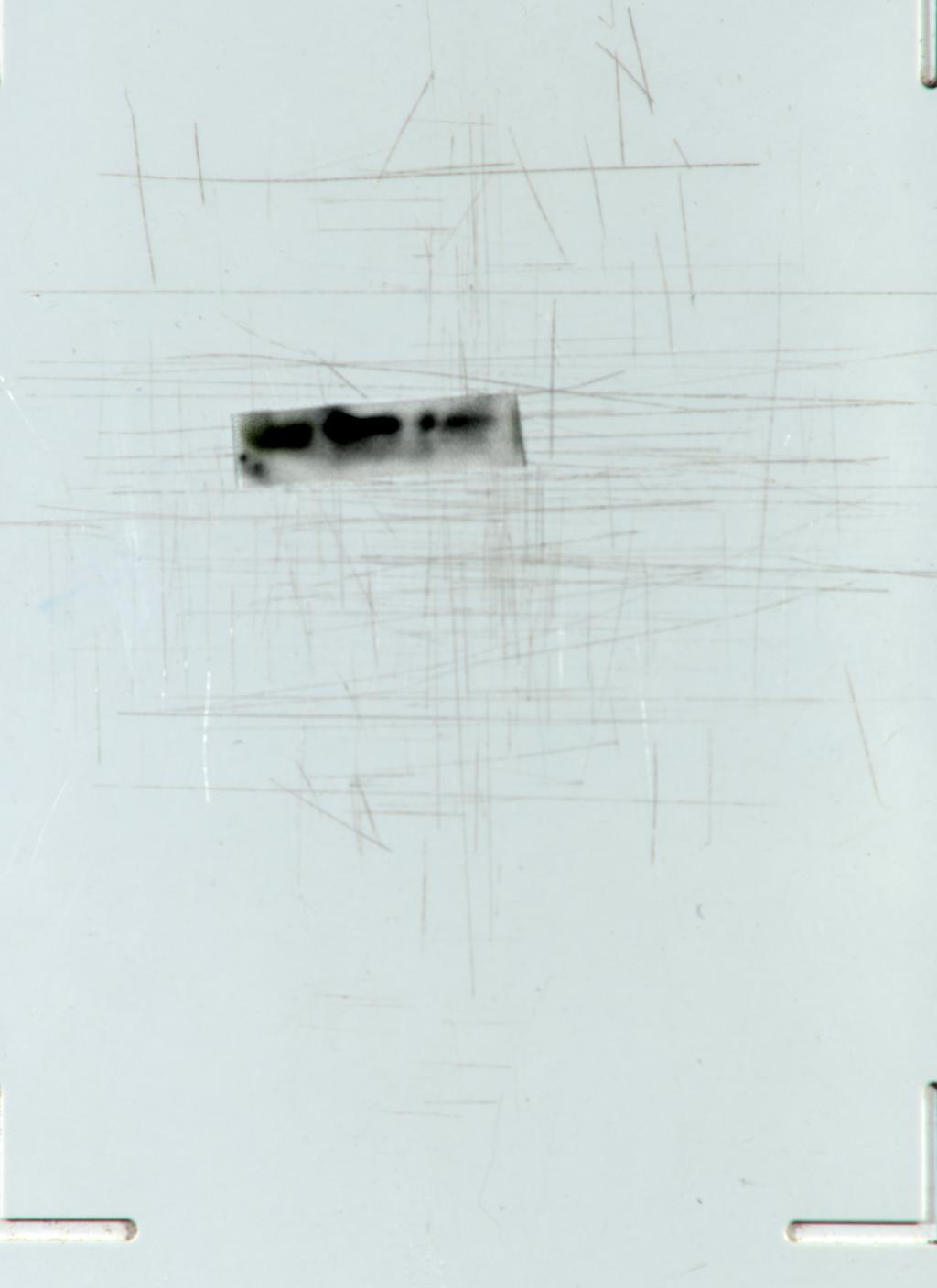

Supplement: Supplemental Information 11 [file peerj-11-15041-s011.zip › Transcriptome-related genes-raw data4/ELANE/ELANE-1/ELANE-1-2.jpg]

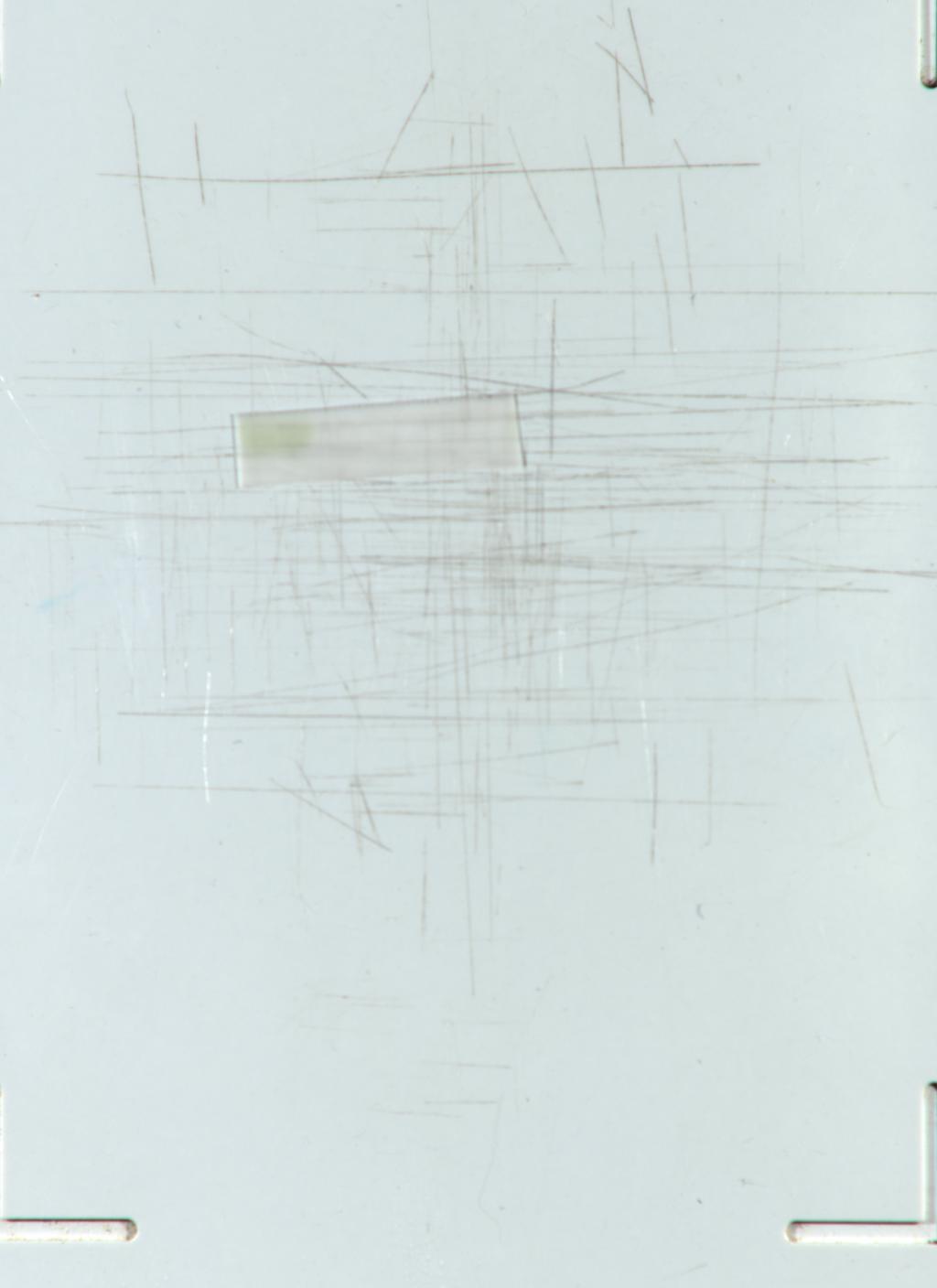

Supplement: Supplemental Information 11 [file peerj-11-15041-s011.zip › Transcriptome-related genes-raw data4/ELANE/ELANE-1/ELANE-1-3.jpg]

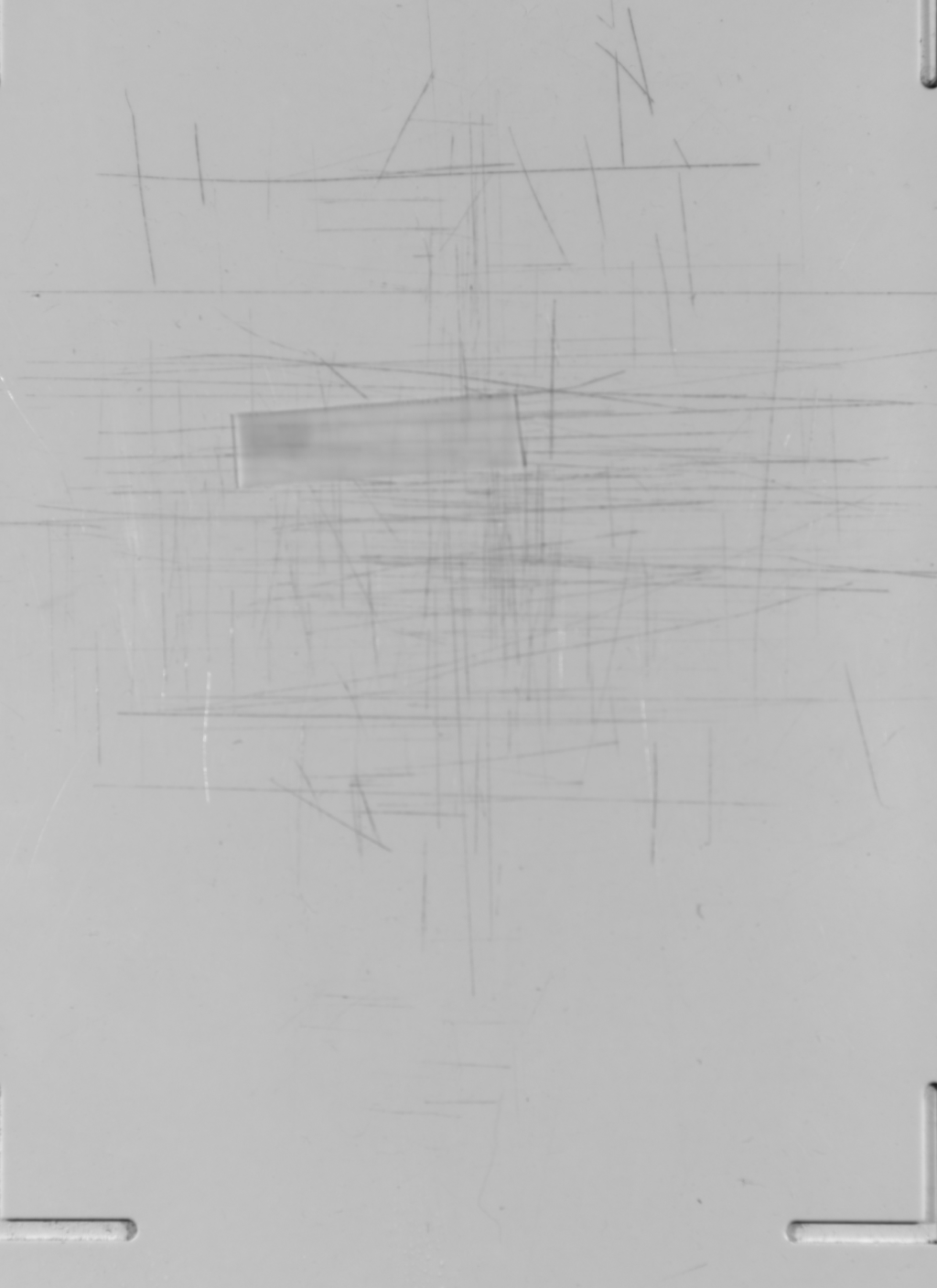

Supplement: Supplemental Information 11 [file peerj-11-15041-s011.zip › Transcriptome-related genes-raw data4/ELANE/ELANE-1/ELANE-1-4.tif]

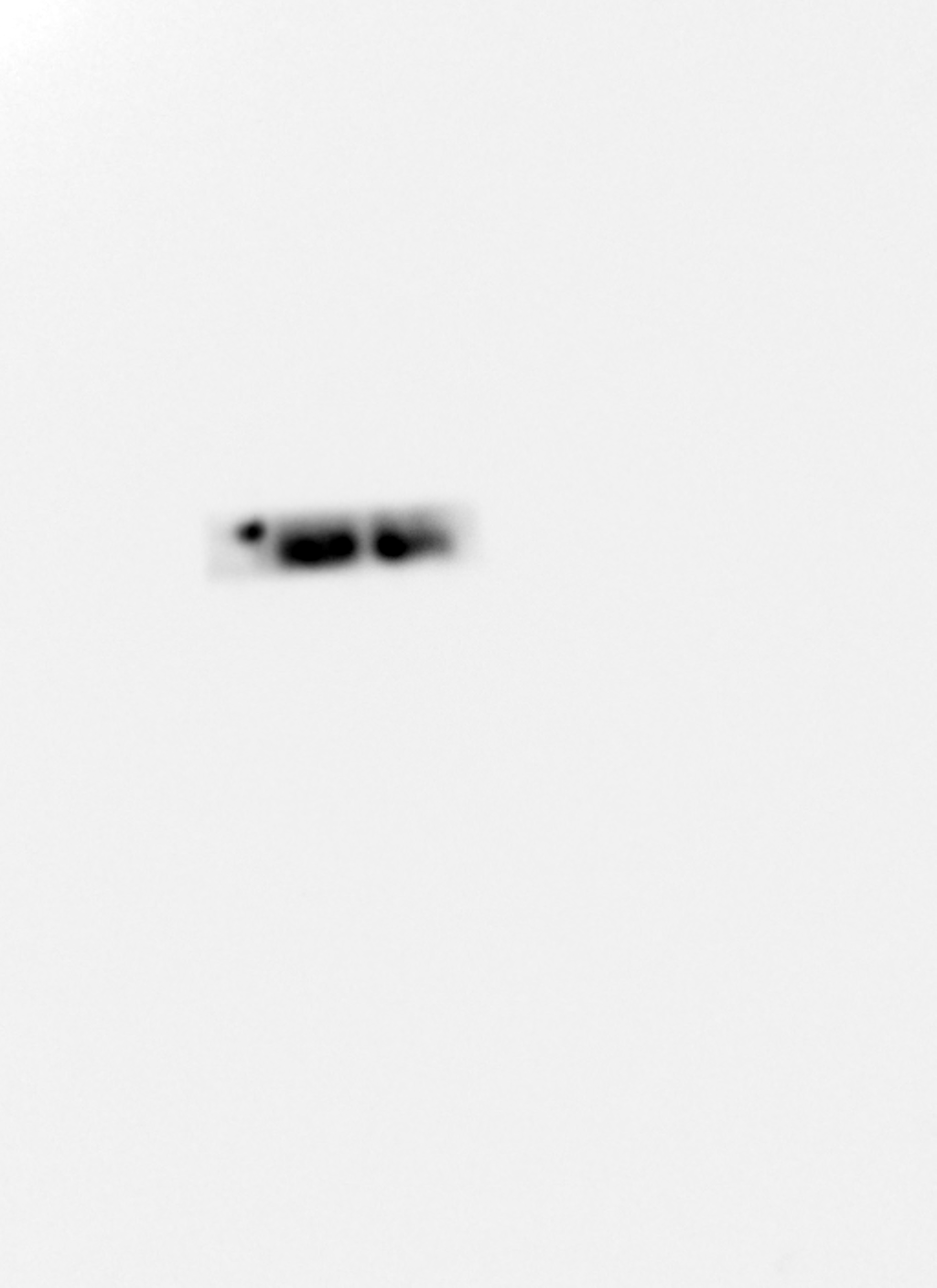

Supplement: Supplemental Information 11 [file peerj-11-15041-s011.zip › Transcriptome-related genes-raw data4/ELANE/ELANE-2/ELANE-2-1.tif]

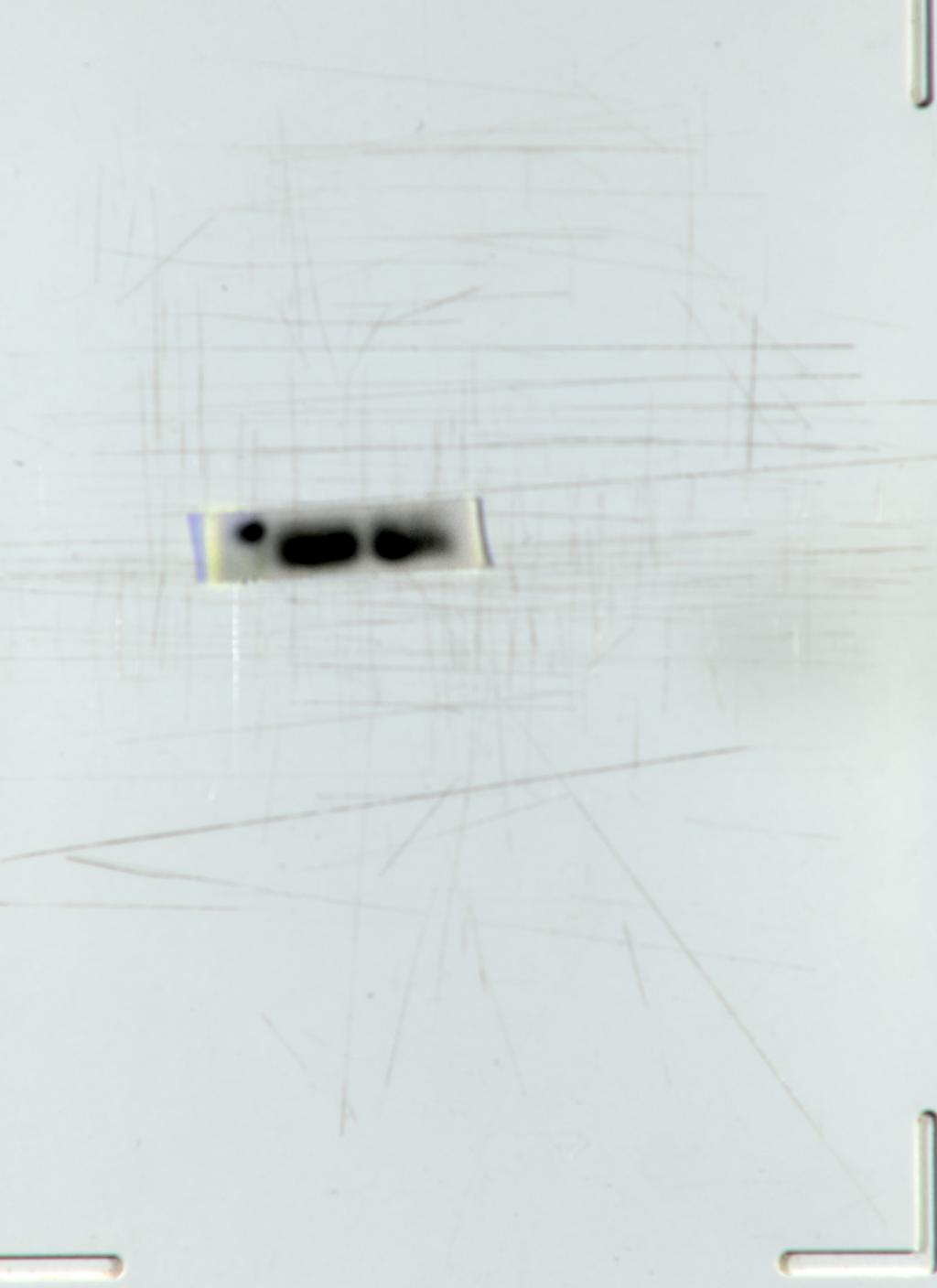

Supplement: Supplemental Information 11 [file peerj-11-15041-s011.zip › Transcriptome-related genes-raw data4/ELANE/ELANE-2/ELANE-2-2.jpg]

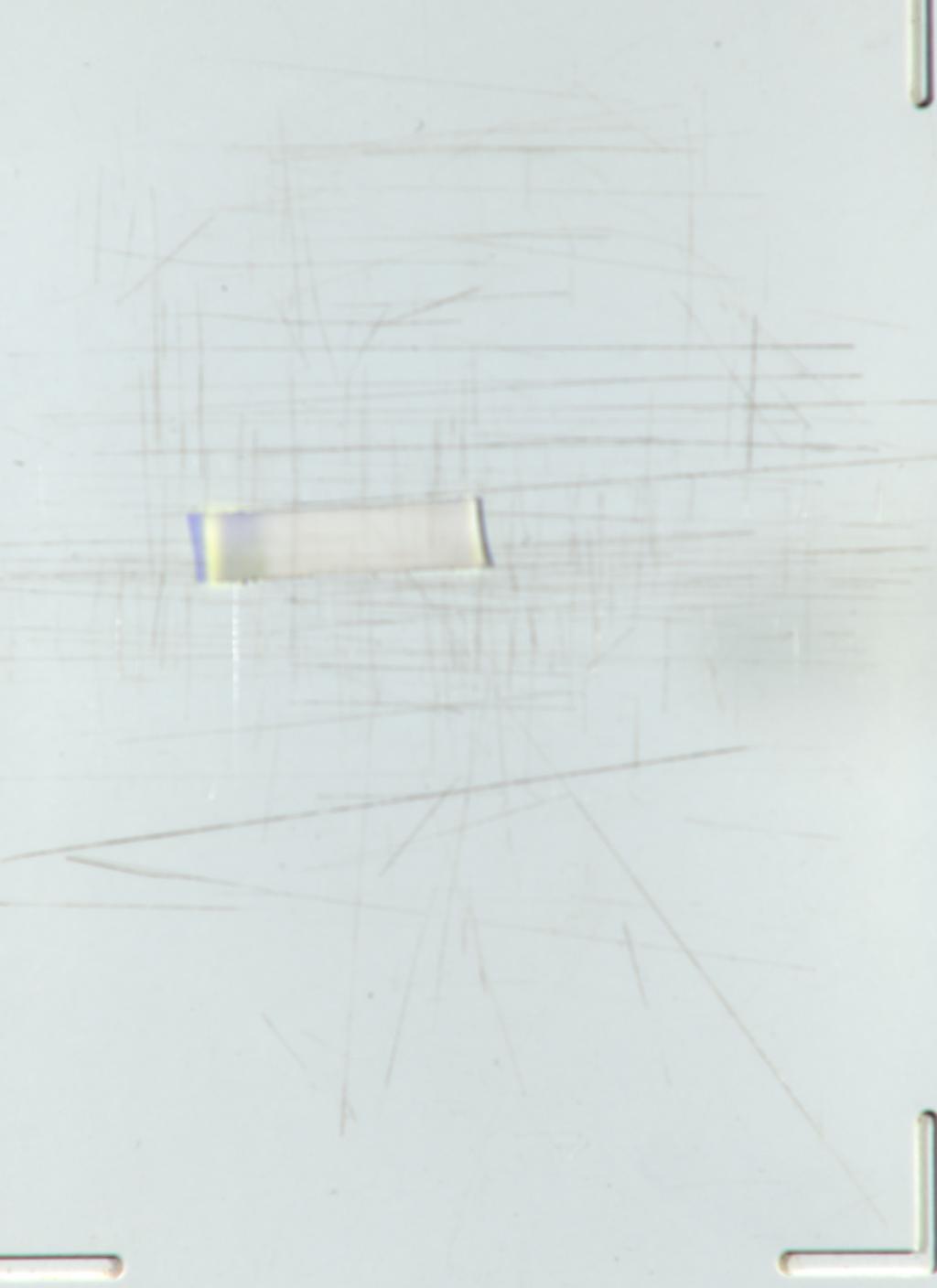

Supplement: Supplemental Information 11 [file peerj-11-15041-s011.zip › Transcriptome-related genes-raw data4/ELANE/ELANE-2/ELANE-2-3.jpg]
