# Supplementary figures and images for: CYP27A1 deficiency promoted osteoclast differentiation (part 3 of 3)
Source: PeerJ. 2023 Mar 3;11:e15041. doi: 10.7717/peerj.15041 (PMC9987298; doi:10.7717/peerj.15041)

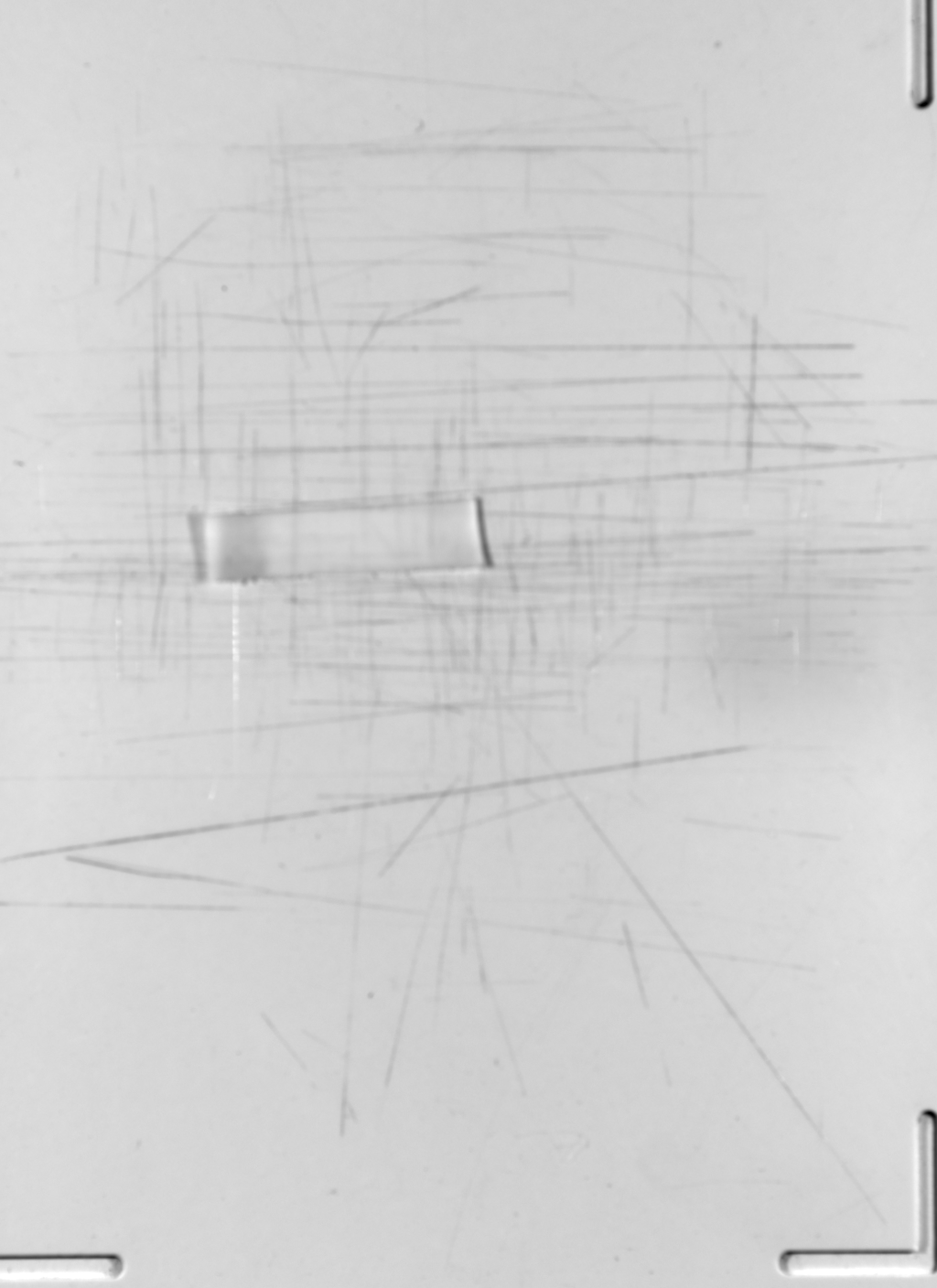

Supplement: Supplemental Information 11 [file peerj-11-15041-s011.zip › Transcriptome-related genes-raw data4/ELANE/ELANE-2/ELANE-2-4.tif]

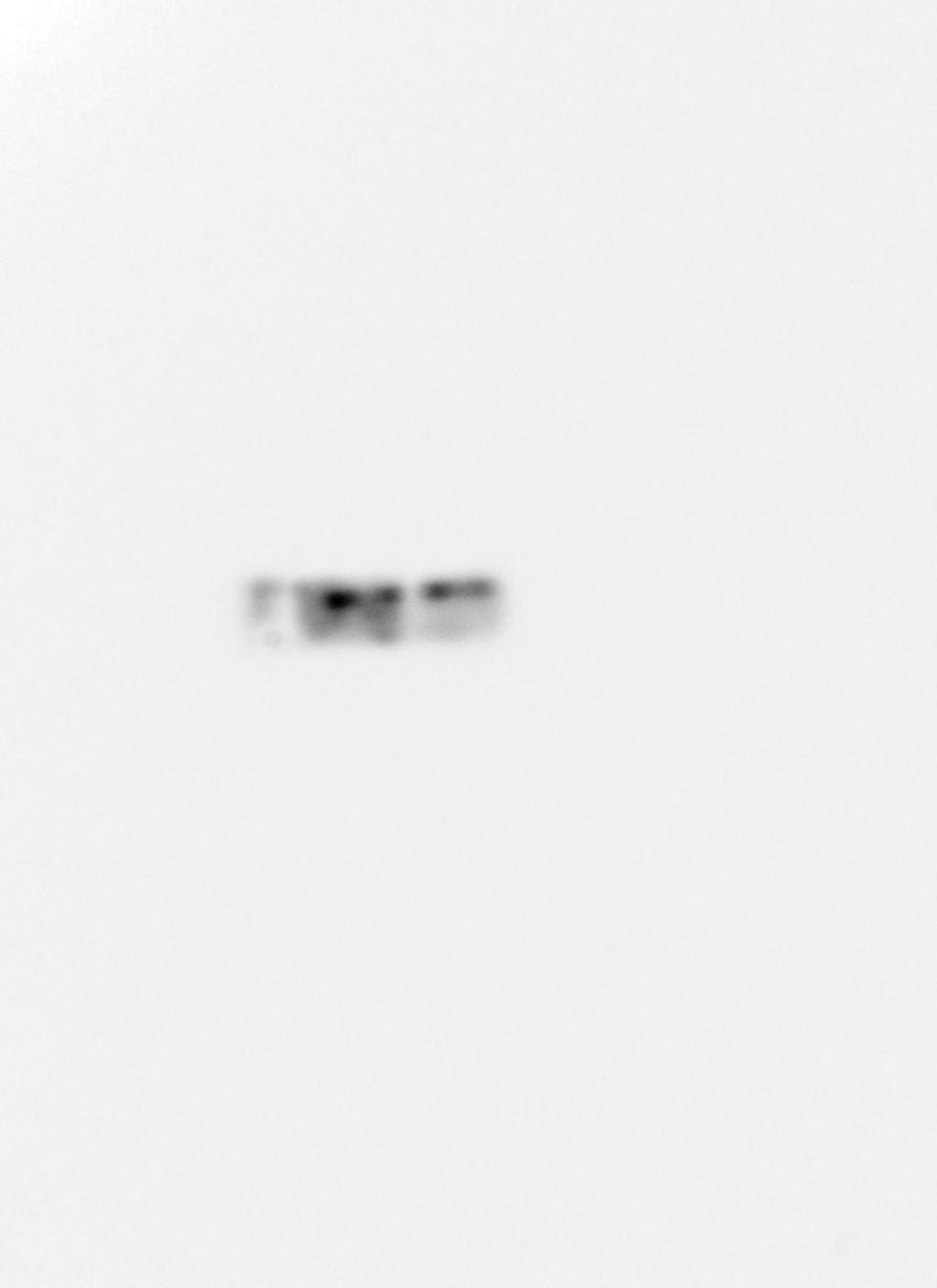

Supplement: Supplemental Information 11 [file peerj-11-15041-s011.zip › Transcriptome-related genes-raw data4/ELANE/ELANE-3/ELANE-3-1.tif]

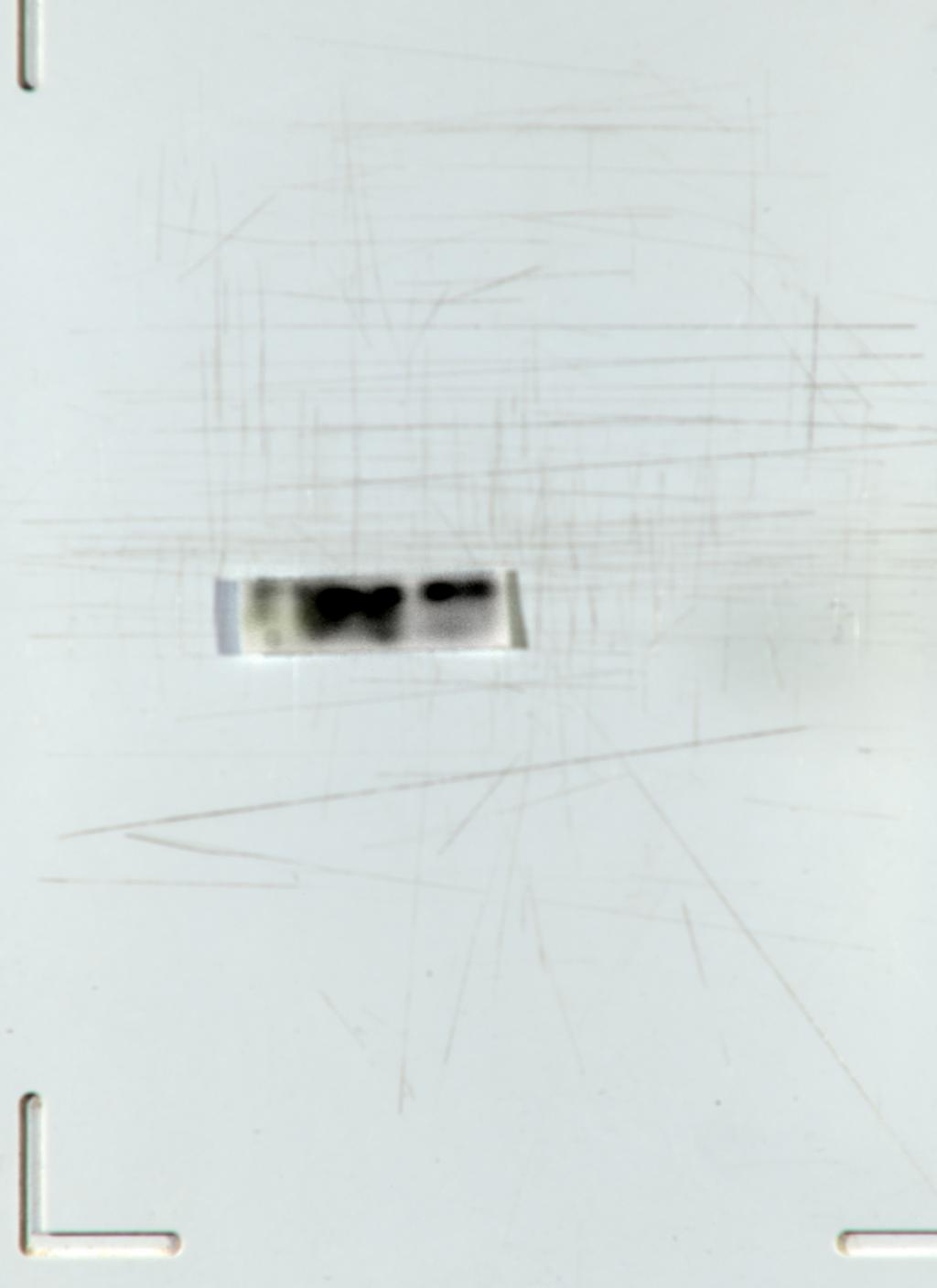

Supplement: Supplemental Information 11 [file peerj-11-15041-s011.zip › Transcriptome-related genes-raw data4/ELANE/ELANE-3/ELANE-3-2.jpg]

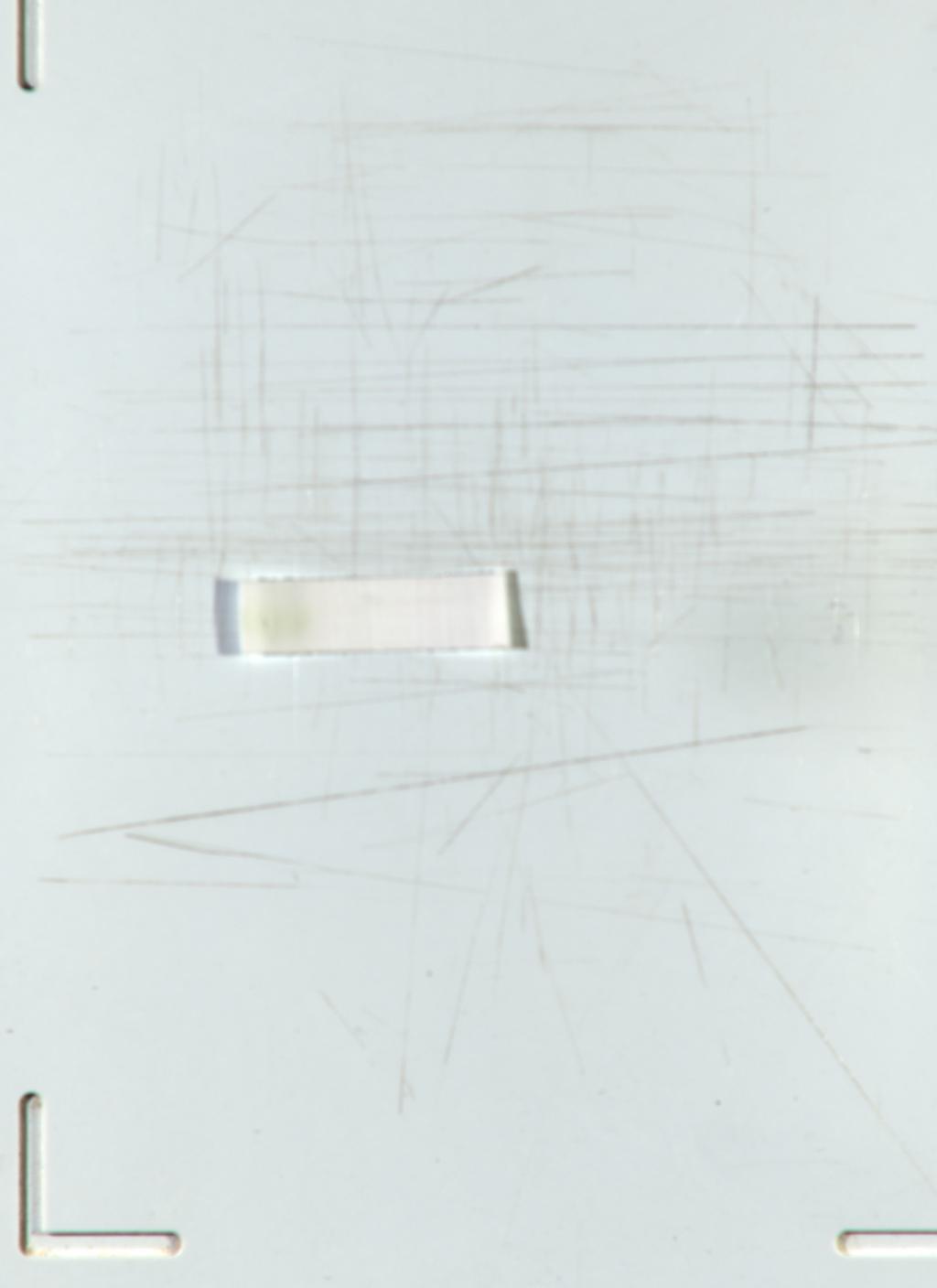

Supplement: Supplemental Information 11 [file peerj-11-15041-s011.zip › Transcriptome-related genes-raw data4/ELANE/ELANE-3/ELANE-3-3.jpg]

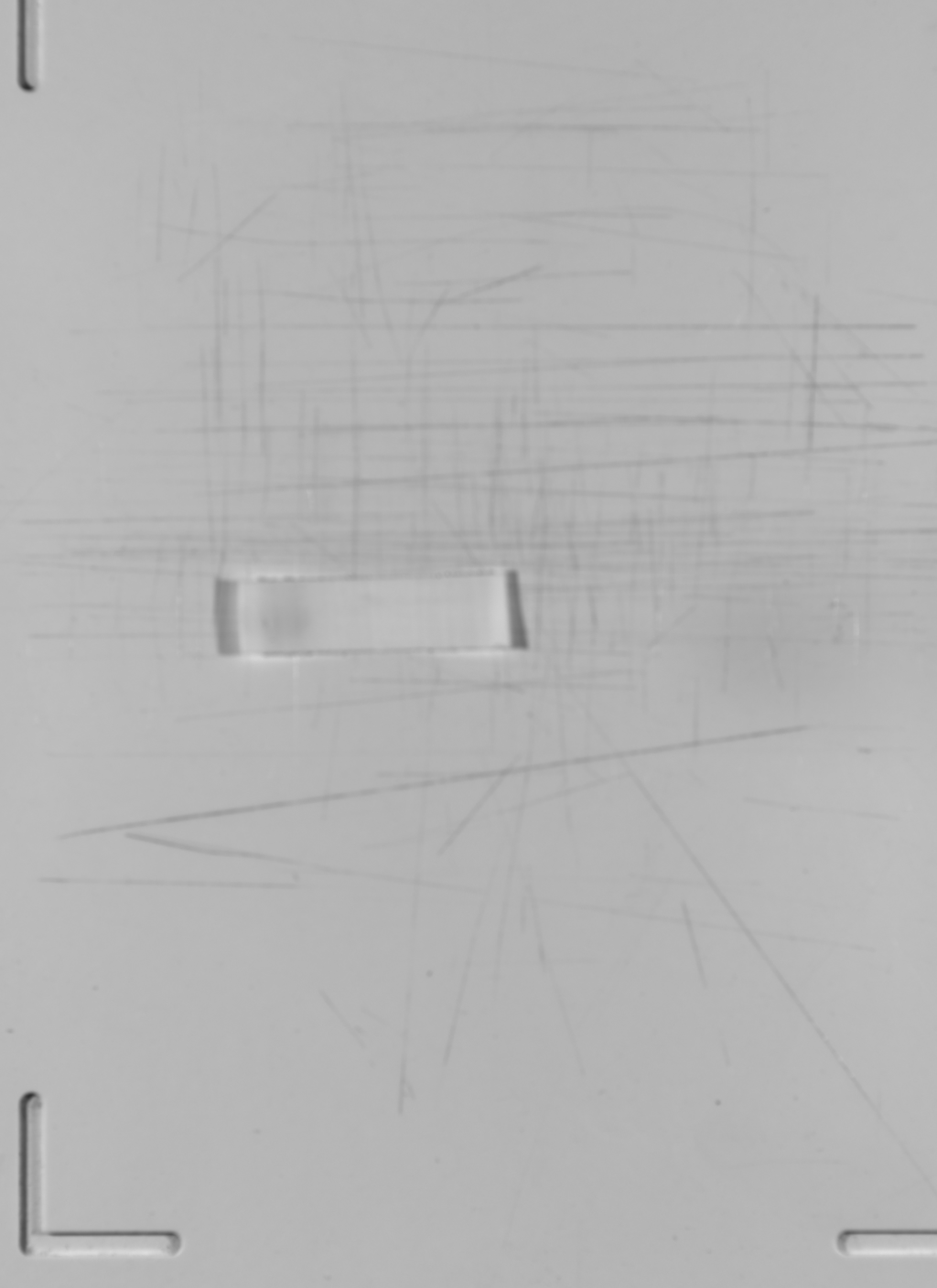

Supplement: Supplemental Information 11 [file peerj-11-15041-s011.zip › Transcriptome-related genes-raw data4/ELANE/ELANE-3/ELANE-3-4.tif]

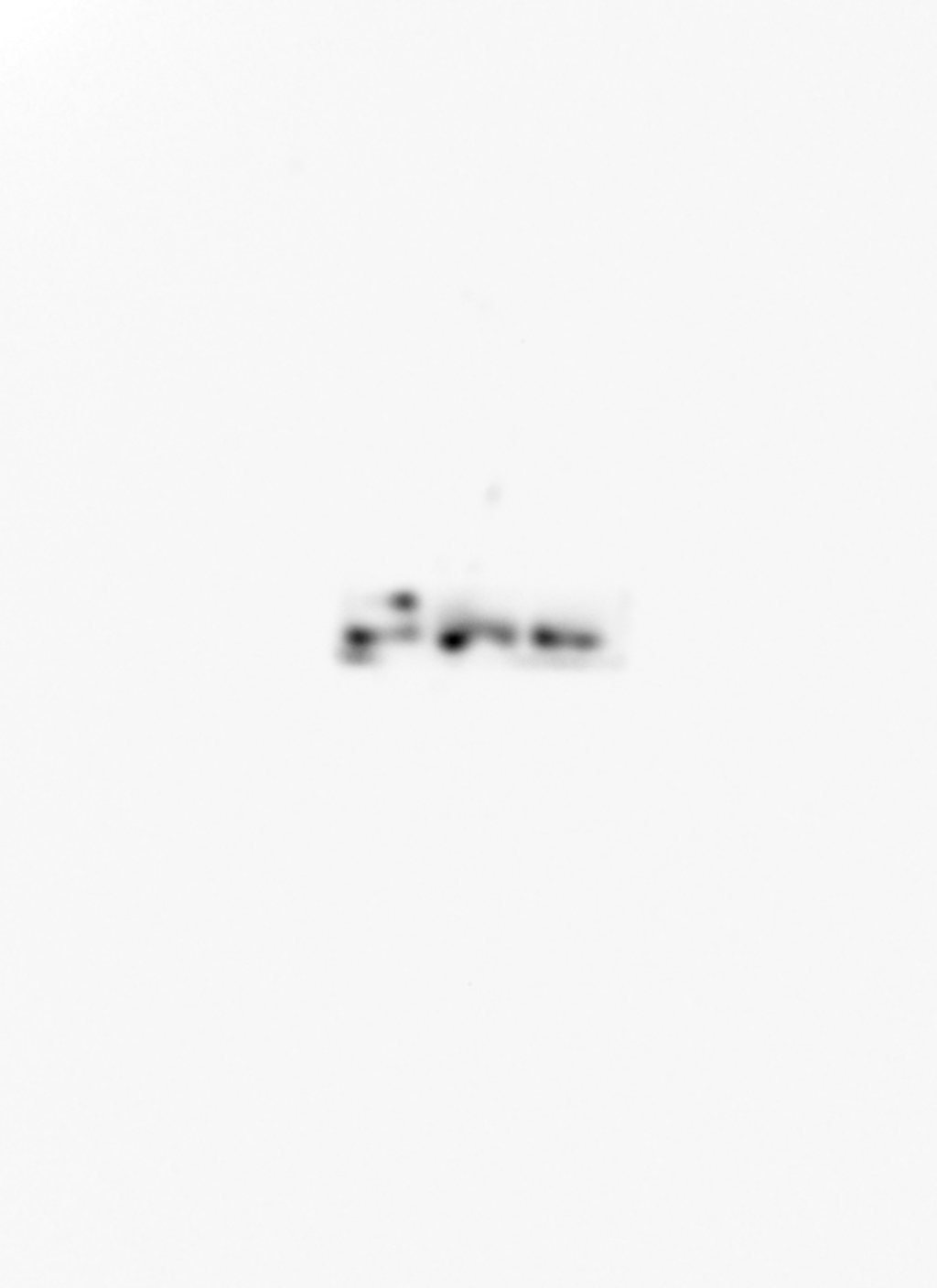

Supplement: Supplemental Information 11 [file peerj-11-15041-s011.zip › Transcriptome-related genes-raw data4/S100A9/S100A9-1/S100A9-1-1.tif]

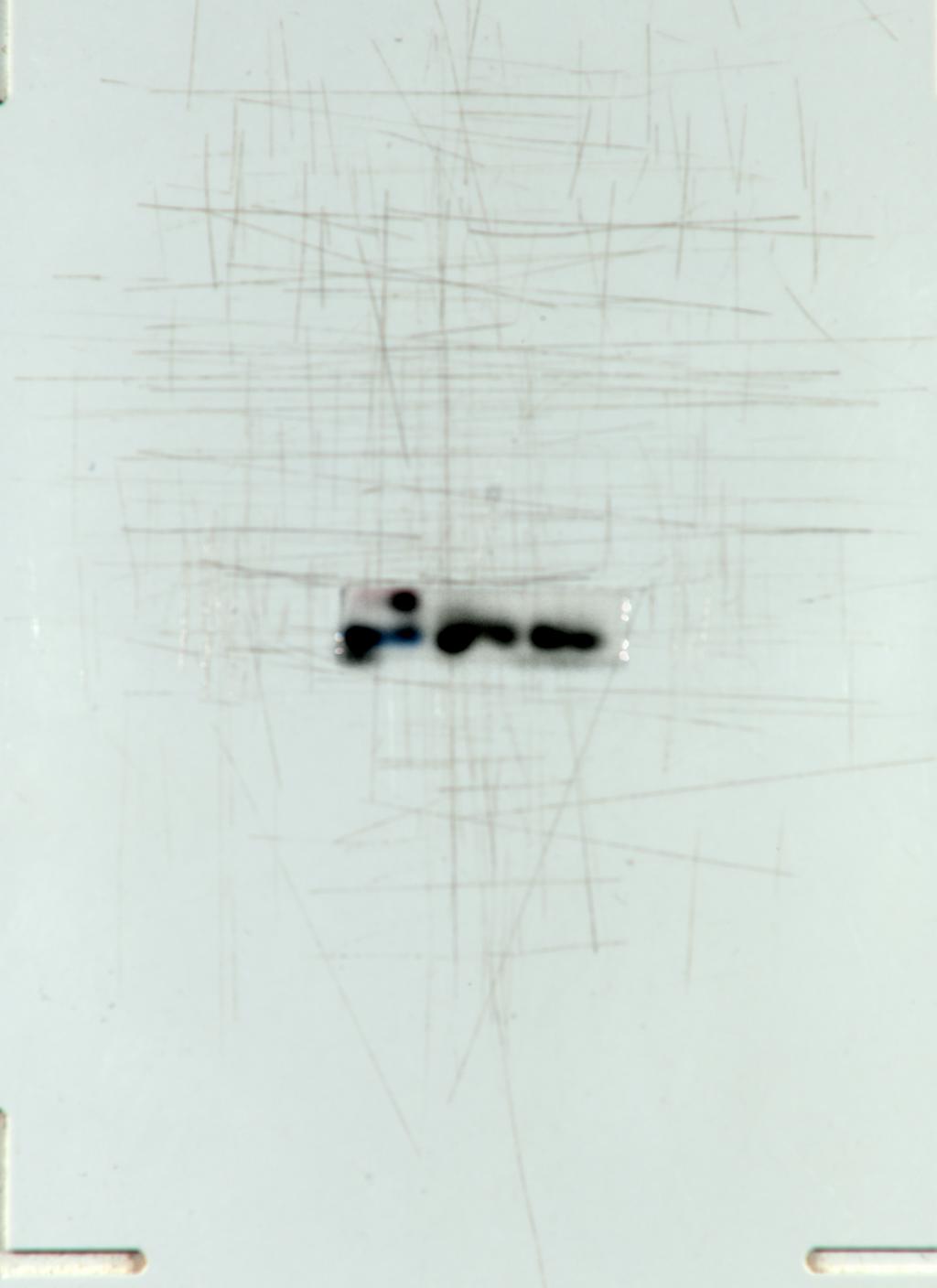

Supplement: Supplemental Information 11 [file peerj-11-15041-s011.zip › Transcriptome-related genes-raw data4/S100A9/S100A9-1/S100A9-1-2.jpg]

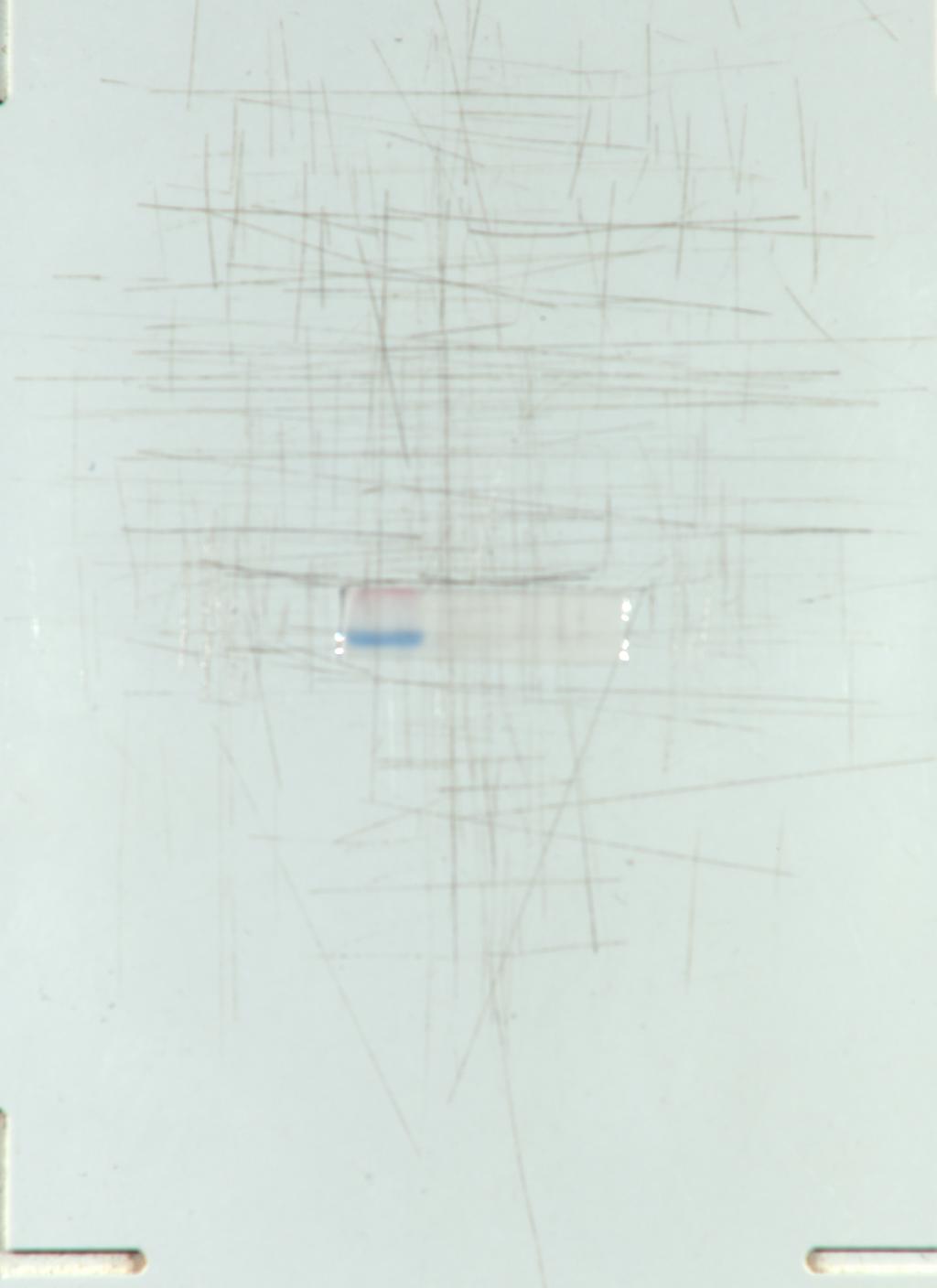

Supplement: Supplemental Information 11 [file peerj-11-15041-s011.zip › Transcriptome-related genes-raw data4/S100A9/S100A9-1/S100A9-1-3.jpg]

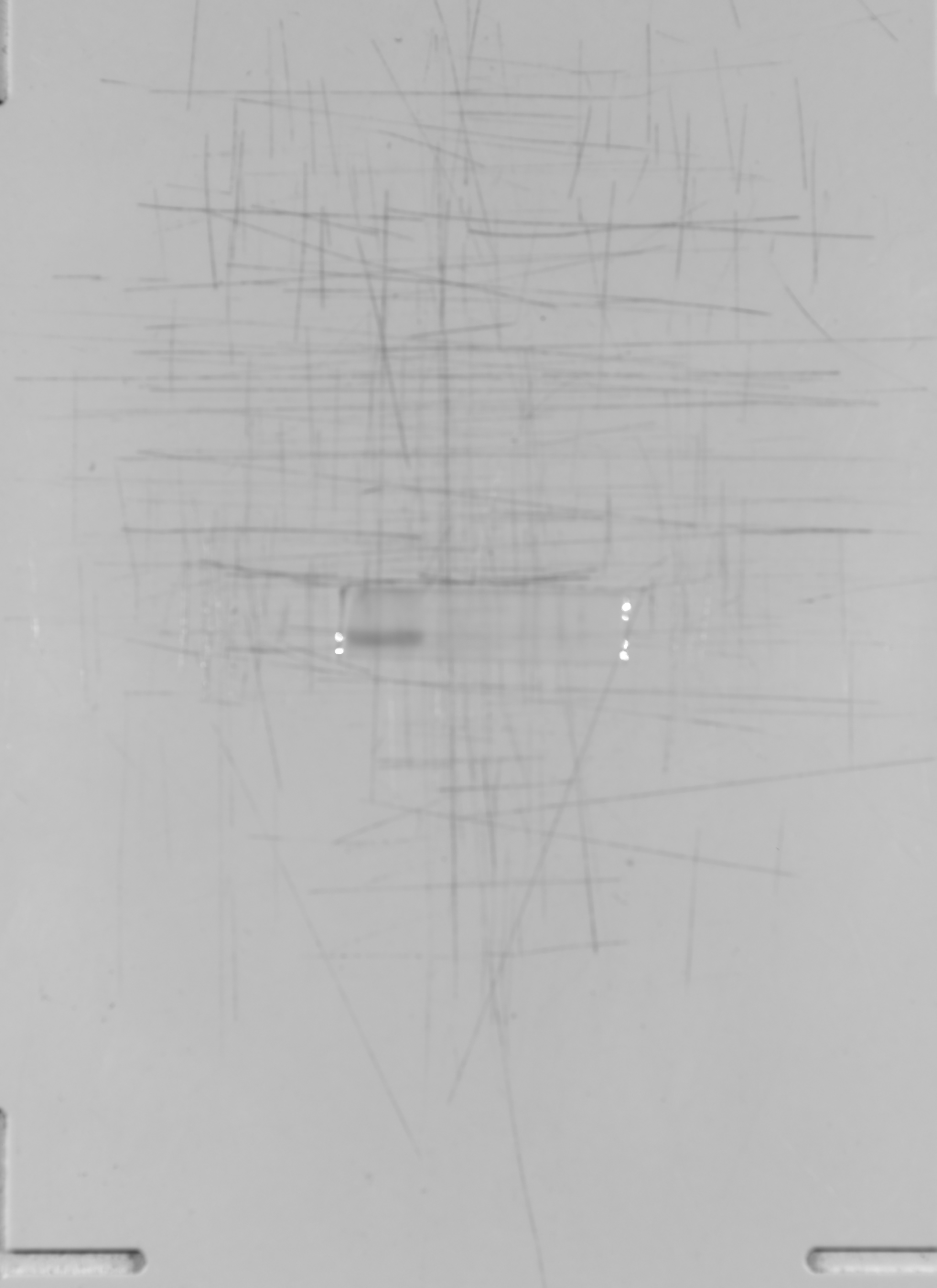

Supplement: Supplemental Information 11 [file peerj-11-15041-s011.zip › Transcriptome-related genes-raw data4/S100A9/S100A9-1/S100A9-1-4.tif]

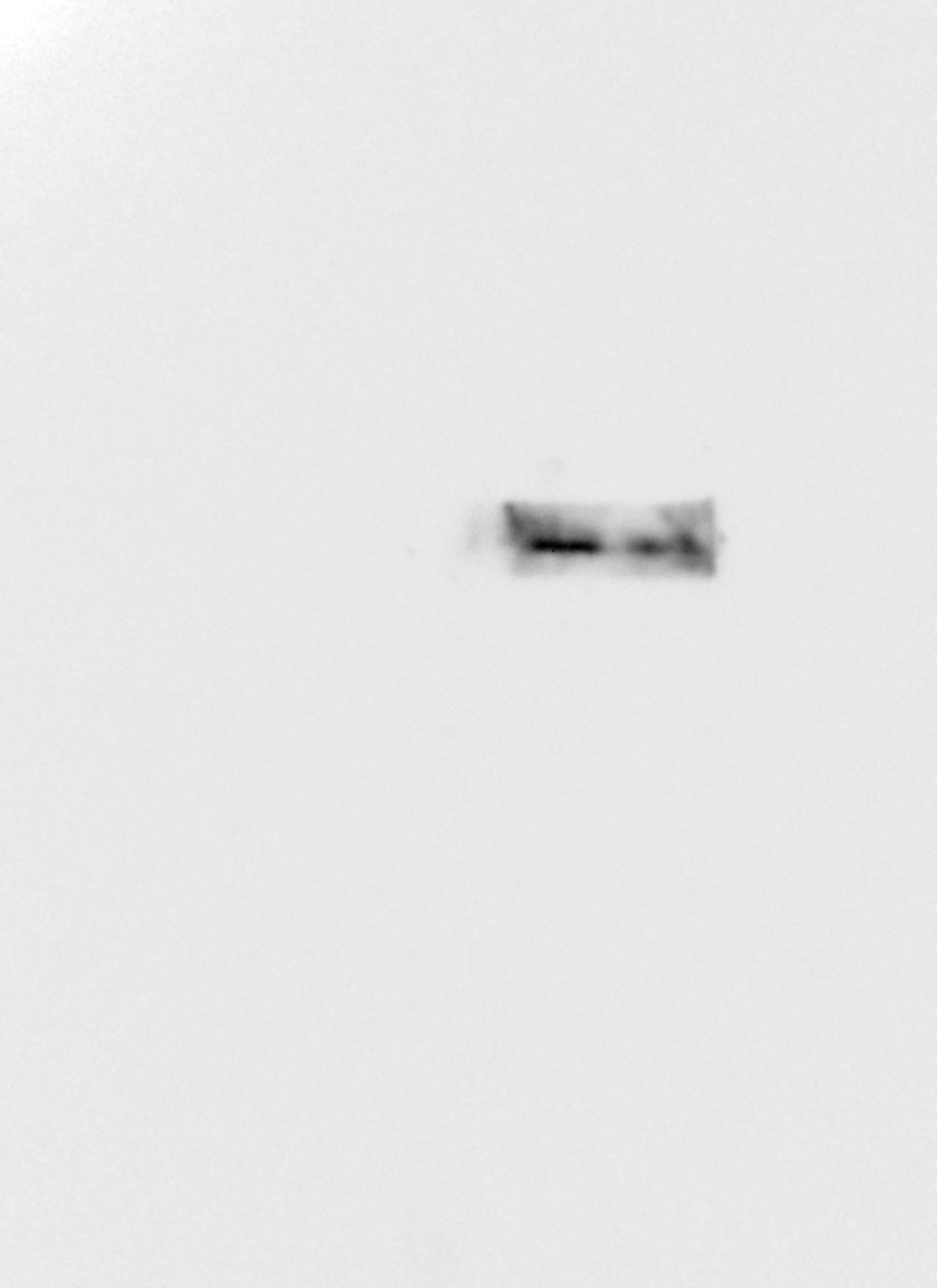

Supplement: Supplemental Information 11 [file peerj-11-15041-s011.zip › Transcriptome-related genes-raw data4/S100A9/S100A9-2/S100A9-2-1.tif]

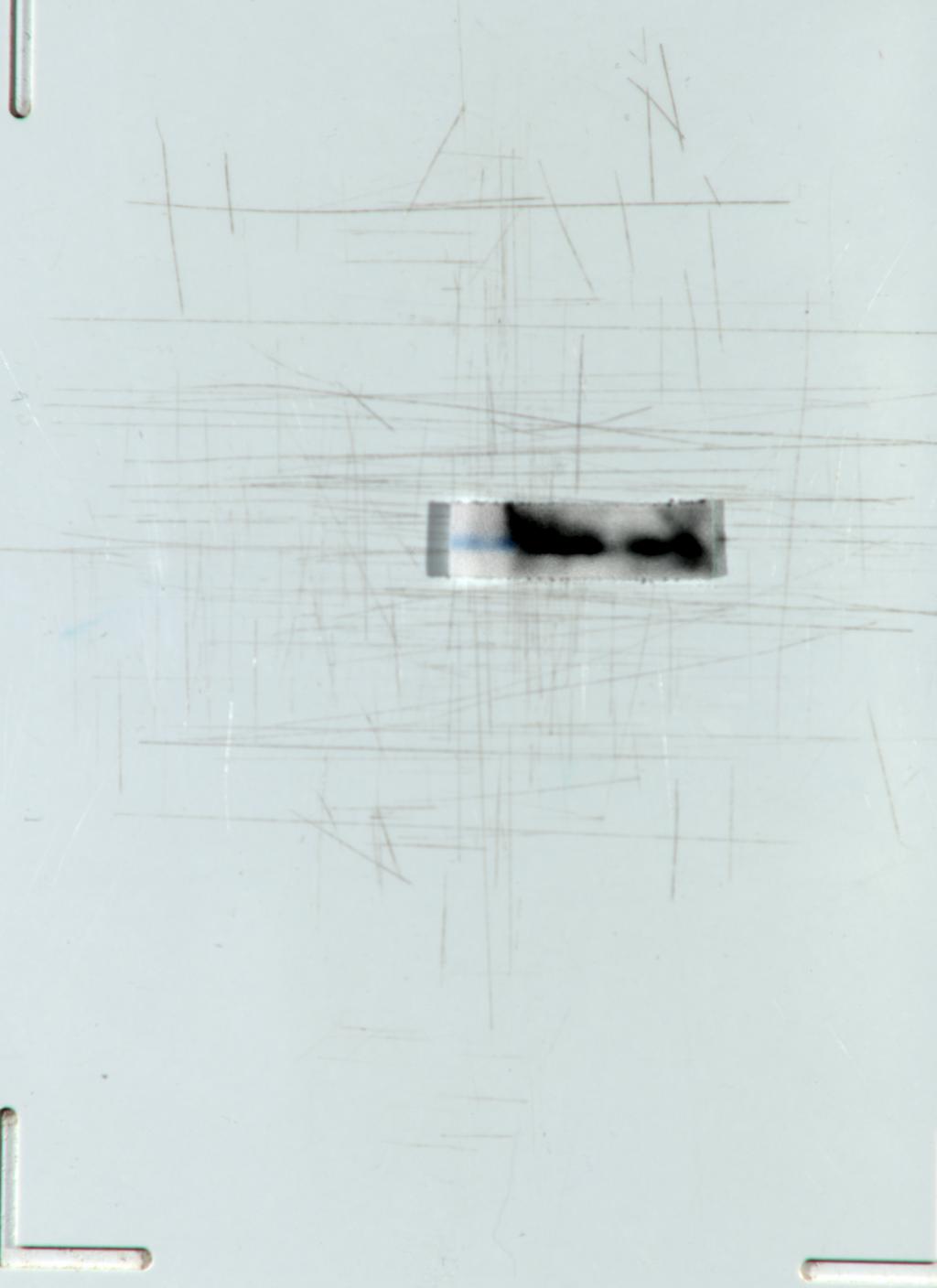

Supplement: Supplemental Information 11 [file peerj-11-15041-s011.zip › Transcriptome-related genes-raw data4/S100A9/S100A9-2/S100A9-2-2.jpg]

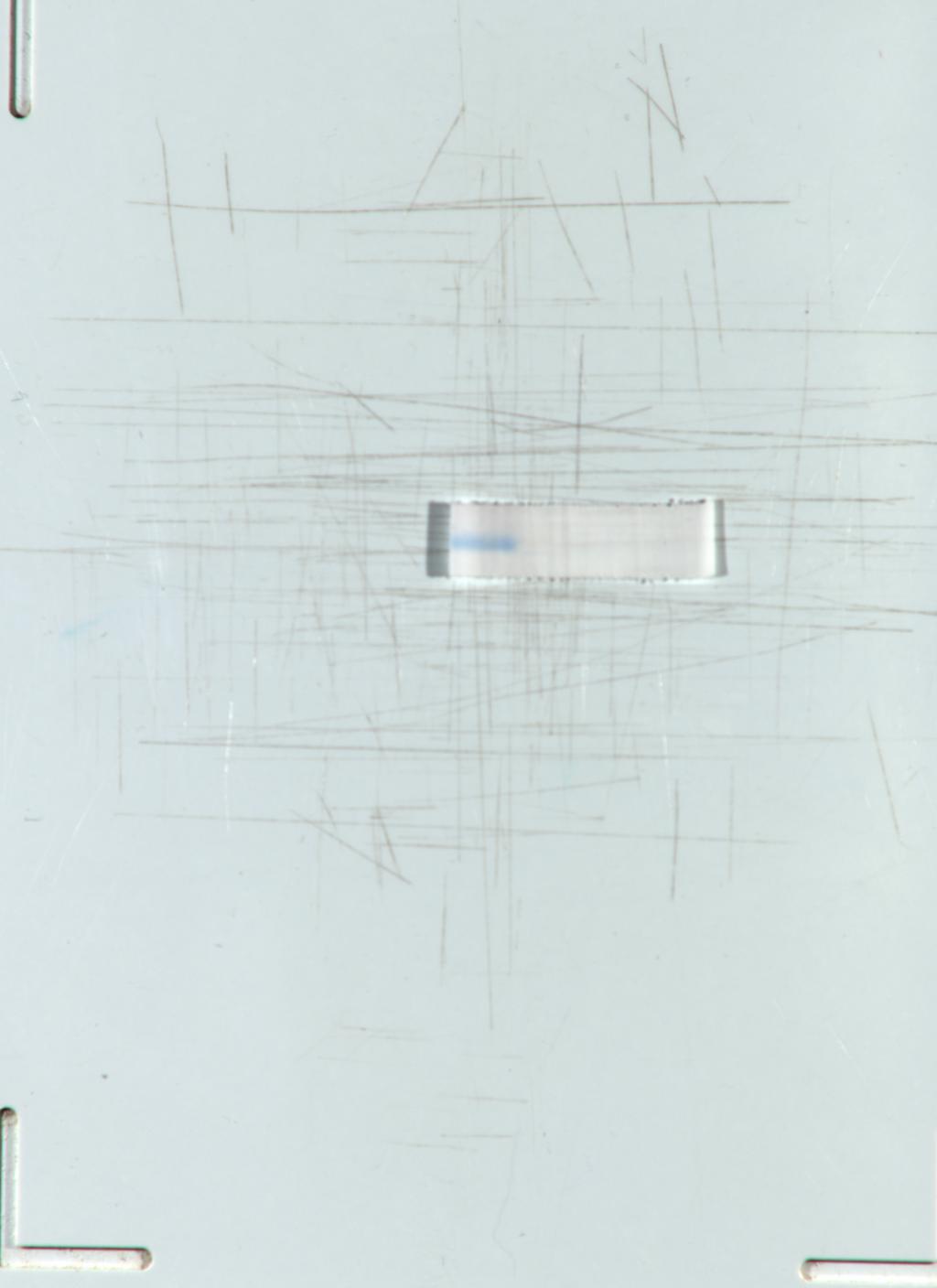

Supplement: Supplemental Information 11 [file peerj-11-15041-s011.zip › Transcriptome-related genes-raw data4/S100A9/S100A9-2/S100A9-2-3.jpg]

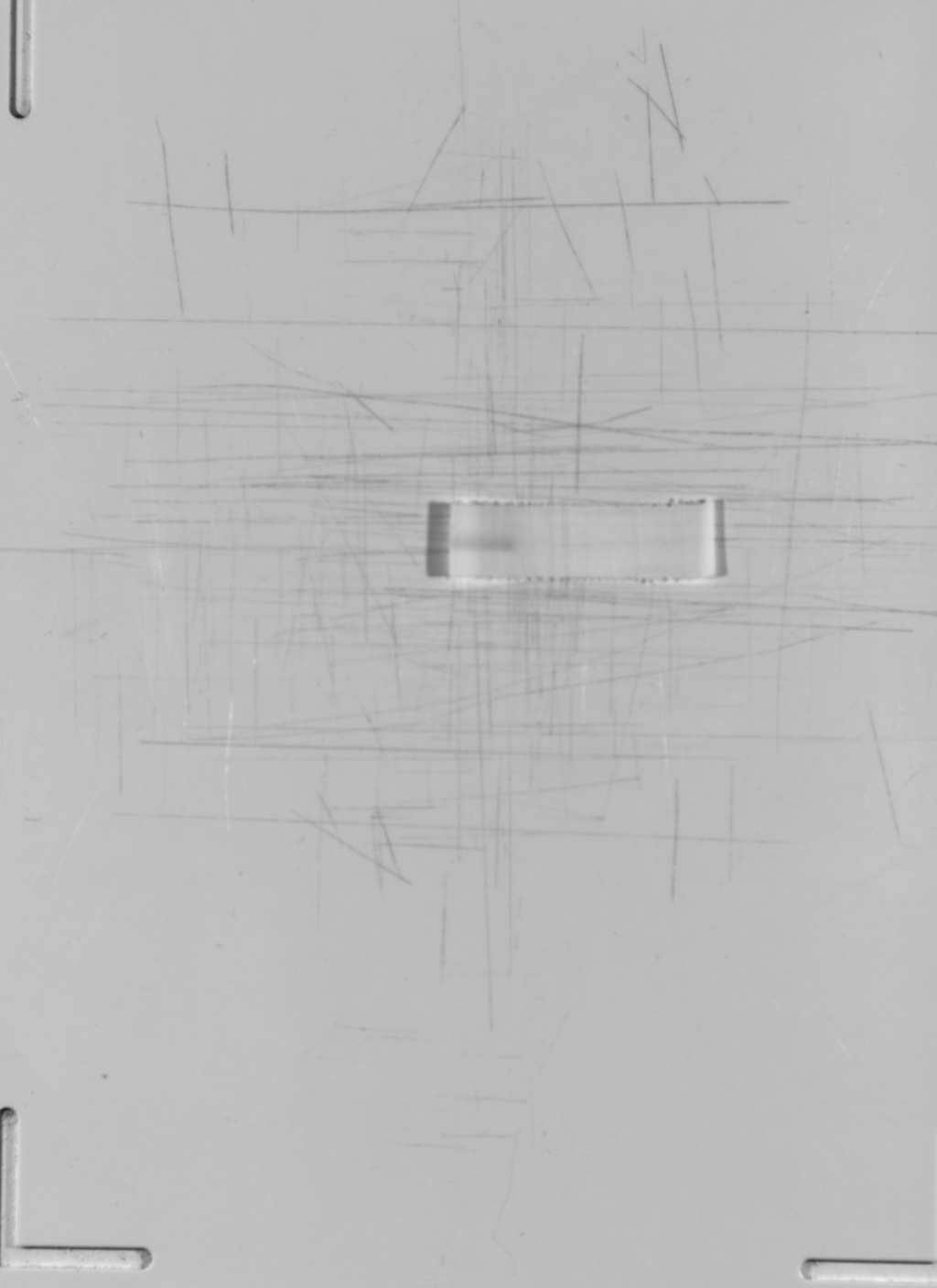

Supplement: Supplemental Information 11 [file peerj-11-15041-s011.zip › Transcriptome-related genes-raw data4/S100A9/S100A9-2/S100A9-2-4.tif]

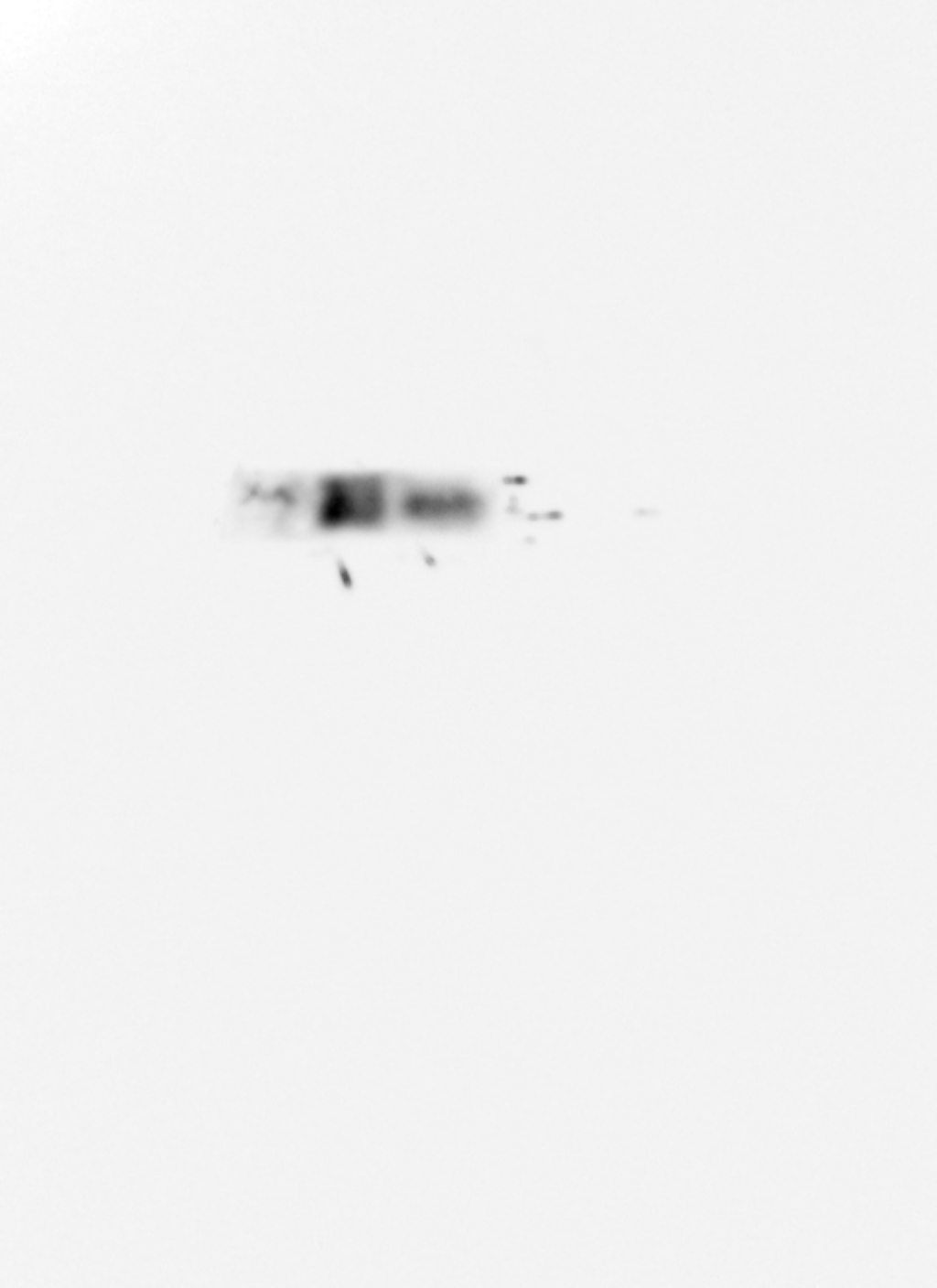

Supplement: Supplemental Information 11 [file peerj-11-15041-s011.zip › Transcriptome-related genes-raw data4/S100A9/S100A9-3/S100A9-3-1.tif]

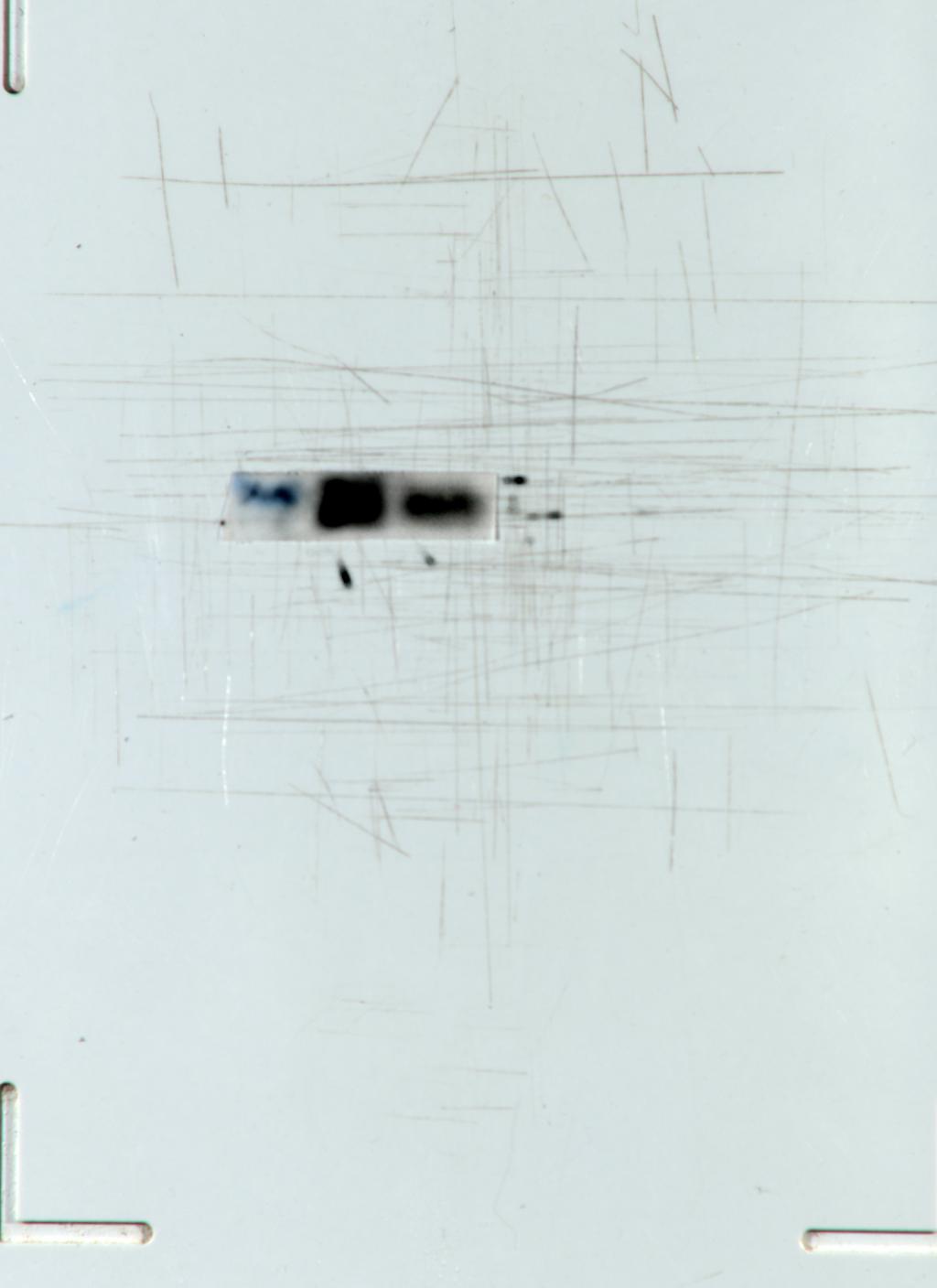

Supplement: Supplemental Information 11 [file peerj-11-15041-s011.zip › Transcriptome-related genes-raw data4/S100A9/S100A9-3/S100A9-3-2.jpg]

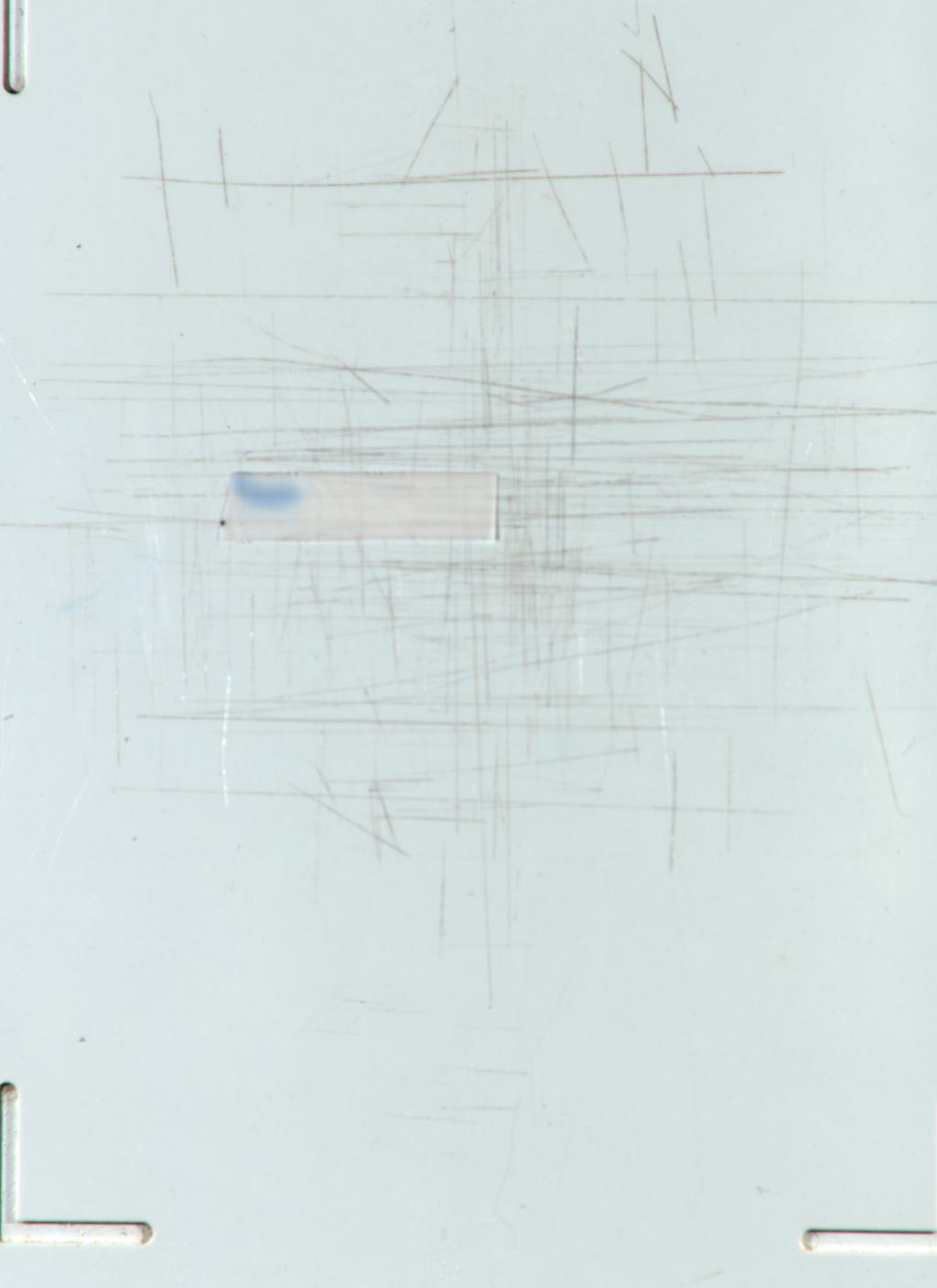

Supplement: Supplemental Information 11 [file peerj-11-15041-s011.zip › Transcriptome-related genes-raw data4/S100A9/S100A9-3/S100A9-3-3.jpg]

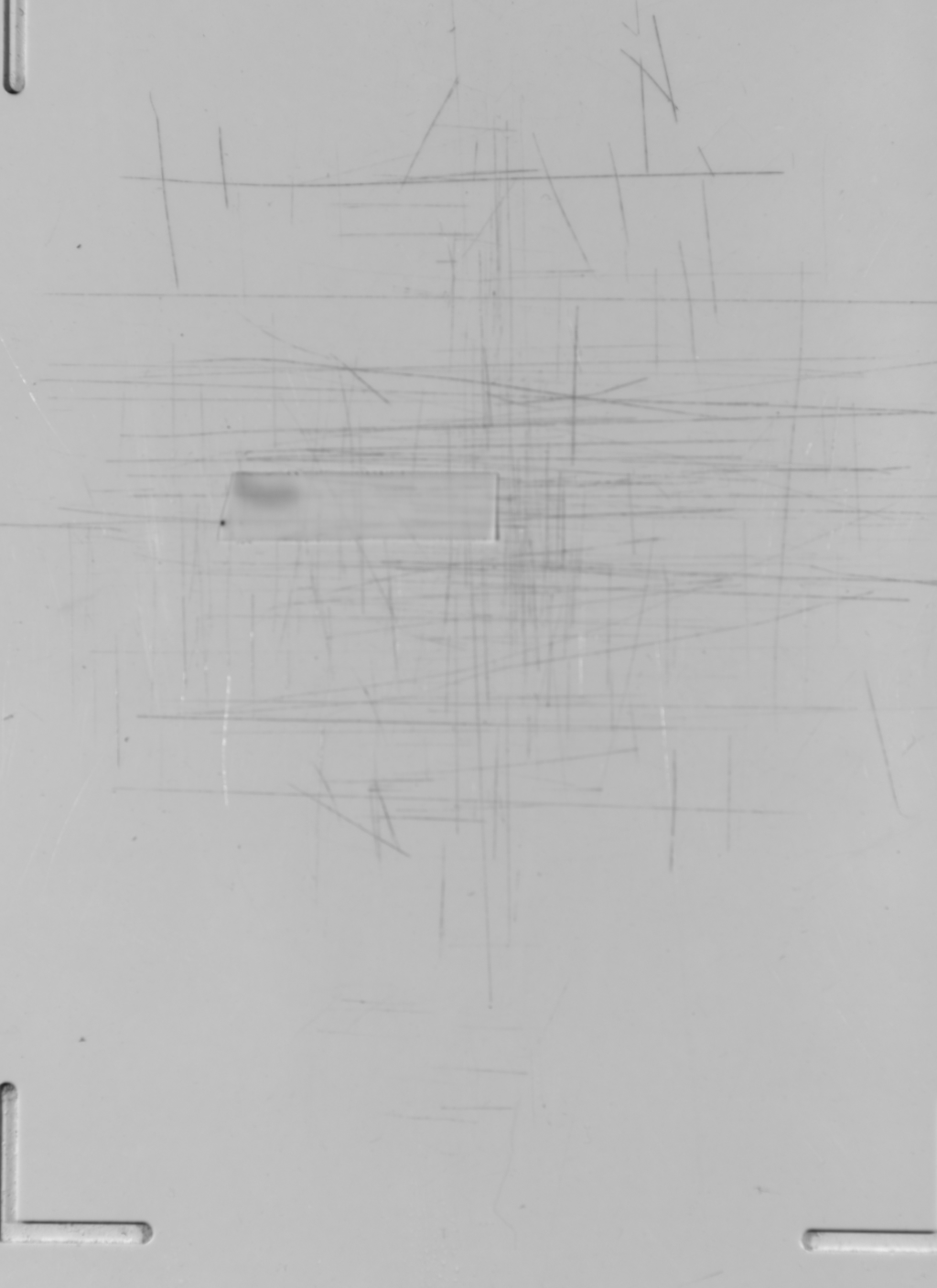

Supplement: Supplemental Information 11 [file peerj-11-15041-s011.zip › Transcriptome-related genes-raw data4/S100A9/S100A9-3/S100A9-3-4.tif]

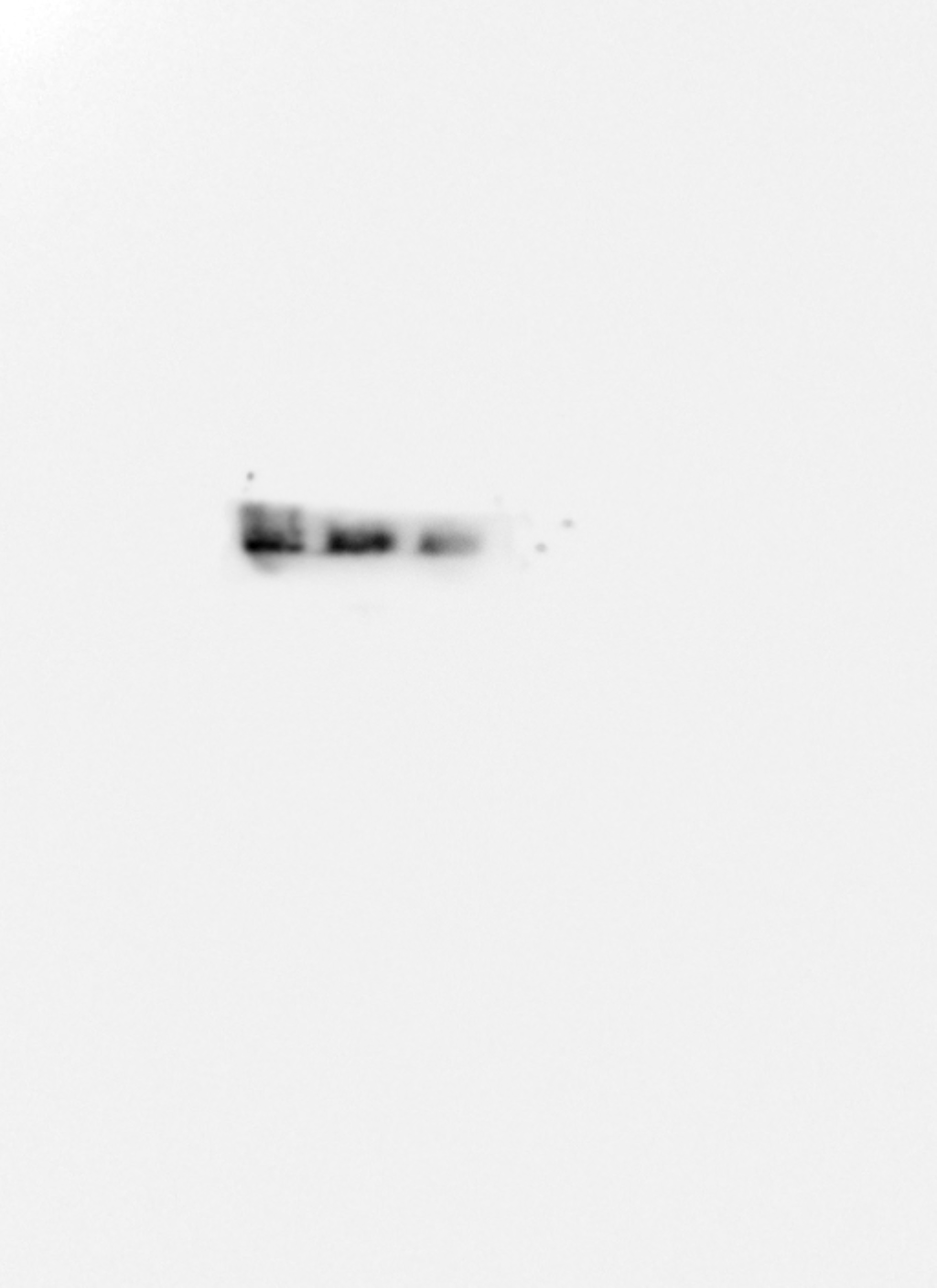

Supplement: Supplemental Information 12 [file peerj-11-15041-s012.zip › Transcriptome-related genes-raw data5/LY6C2/LY6C2-1/LY6C2-1-1.tif]

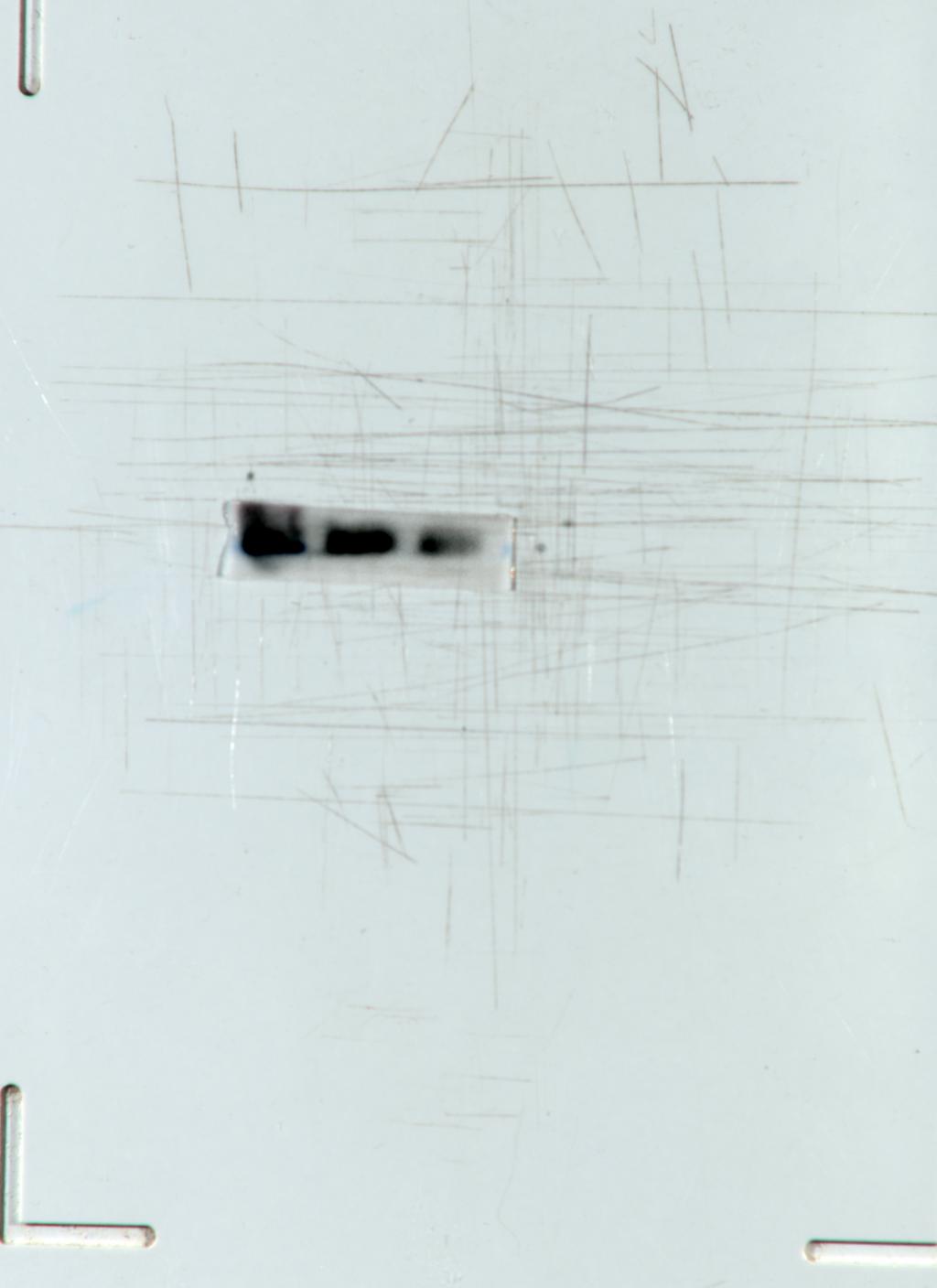

Supplement: Supplemental Information 12 [file peerj-11-15041-s012.zip › Transcriptome-related genes-raw data5/LY6C2/LY6C2-1/LY6C2-1-2.jpg]

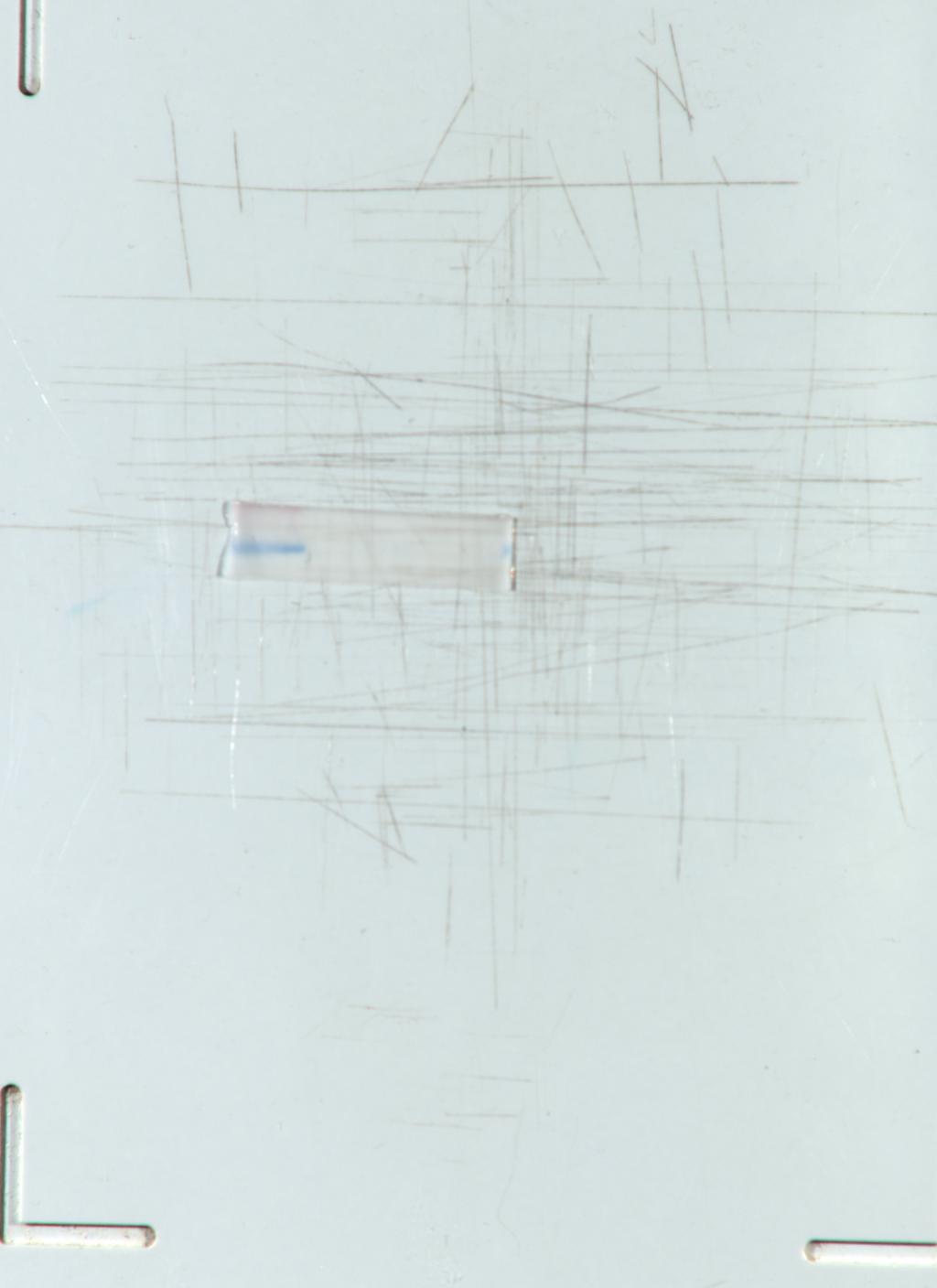

Supplement: Supplemental Information 12 [file peerj-11-15041-s012.zip › Transcriptome-related genes-raw data5/LY6C2/LY6C2-1/LY6C2-1-3.jpg]

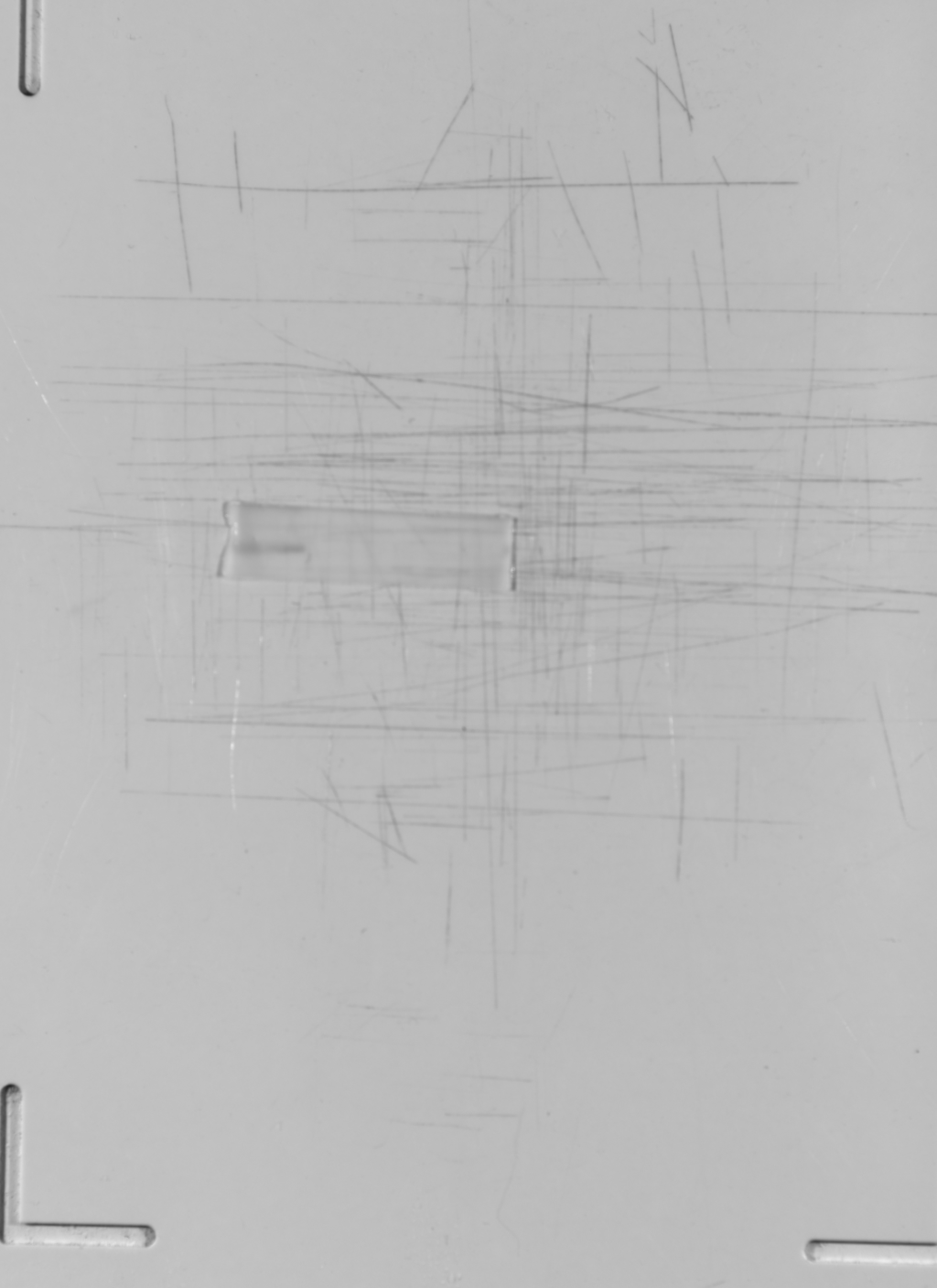

Supplement: Supplemental Information 12 [file peerj-11-15041-s012.zip › Transcriptome-related genes-raw data5/LY6C2/LY6C2-1/LY6C2-1-4.tif]

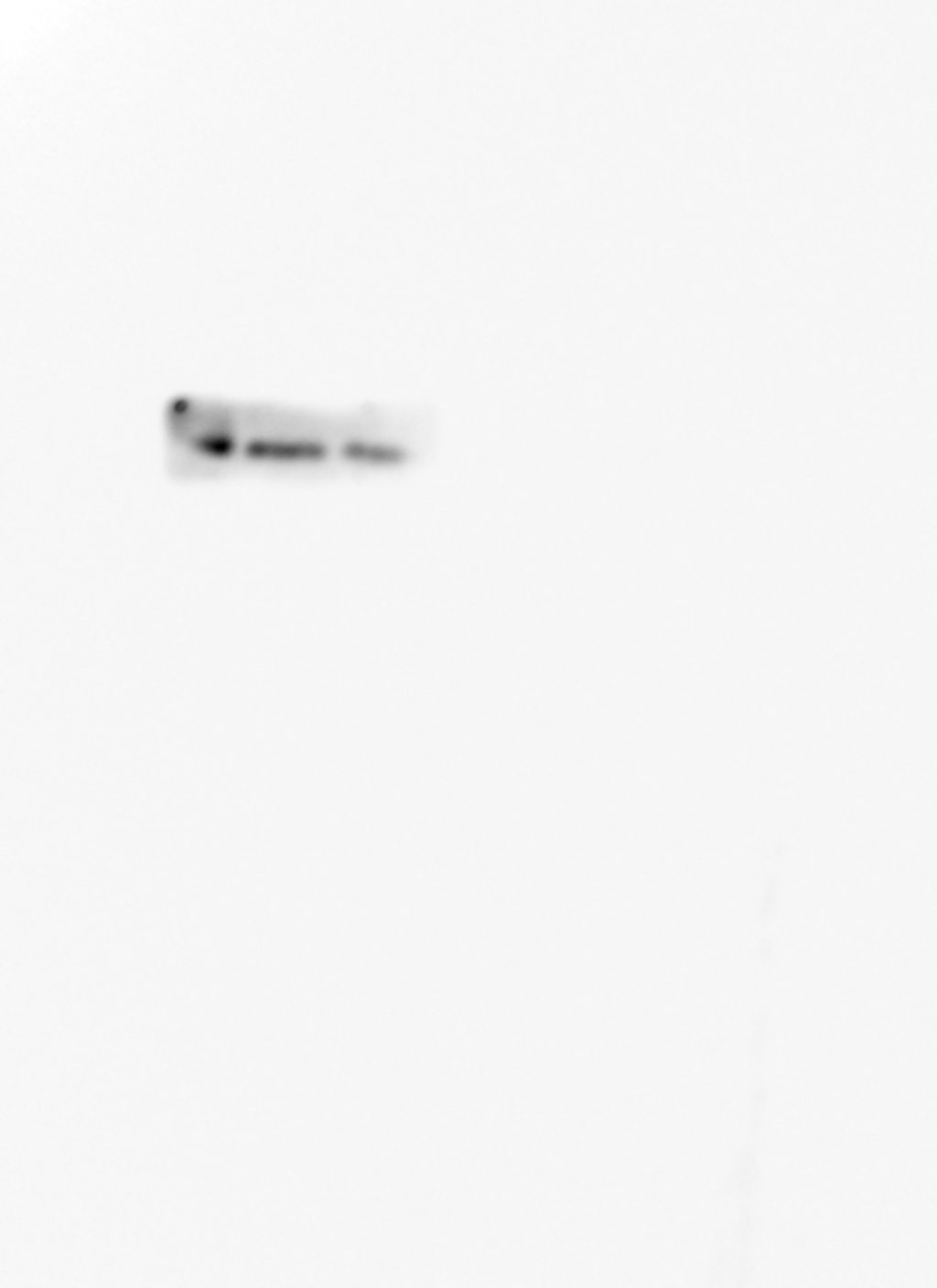

Supplement: Supplemental Information 12 [file peerj-11-15041-s012.zip › Transcriptome-related genes-raw data5/LY6C2/LY6C2-2/LY6C2-2-1.tif]

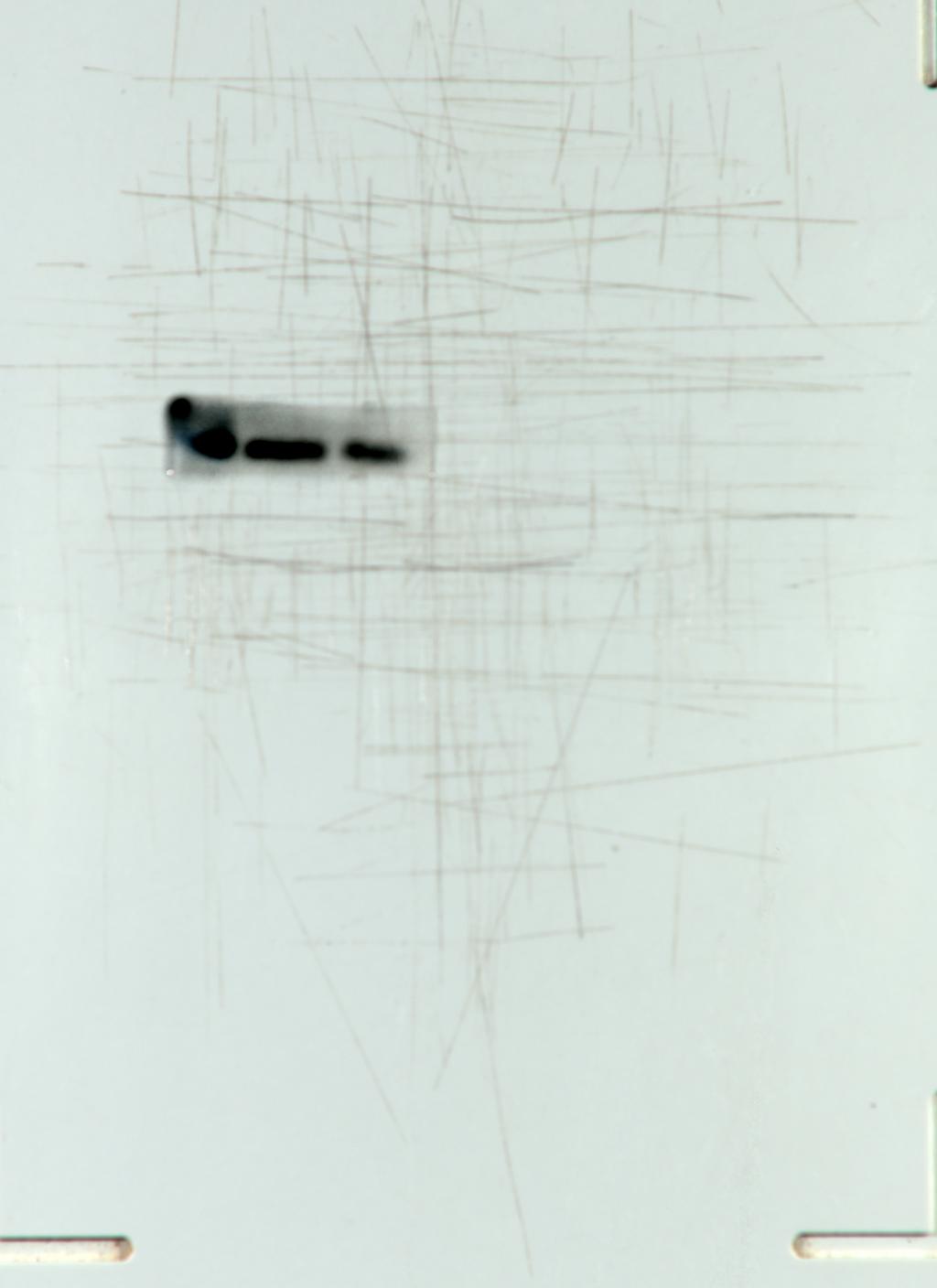

Supplement: Supplemental Information 12 [file peerj-11-15041-s012.zip › Transcriptome-related genes-raw data5/LY6C2/LY6C2-2/LY6C2-2-2.jpg]

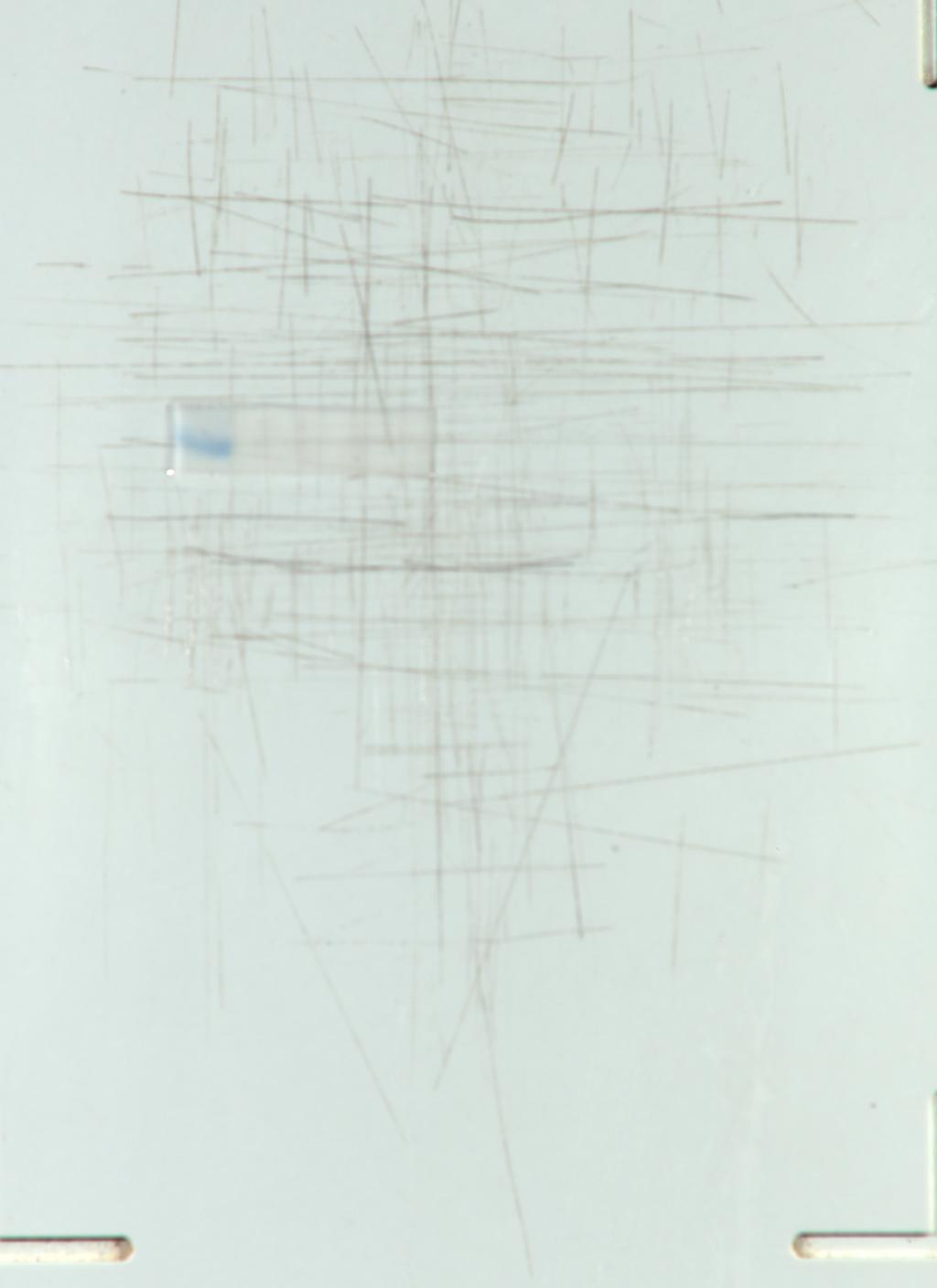

Supplement: Supplemental Information 12 [file peerj-11-15041-s012.zip › Transcriptome-related genes-raw data5/LY6C2/LY6C2-2/LY6C2-2-3.jpg]

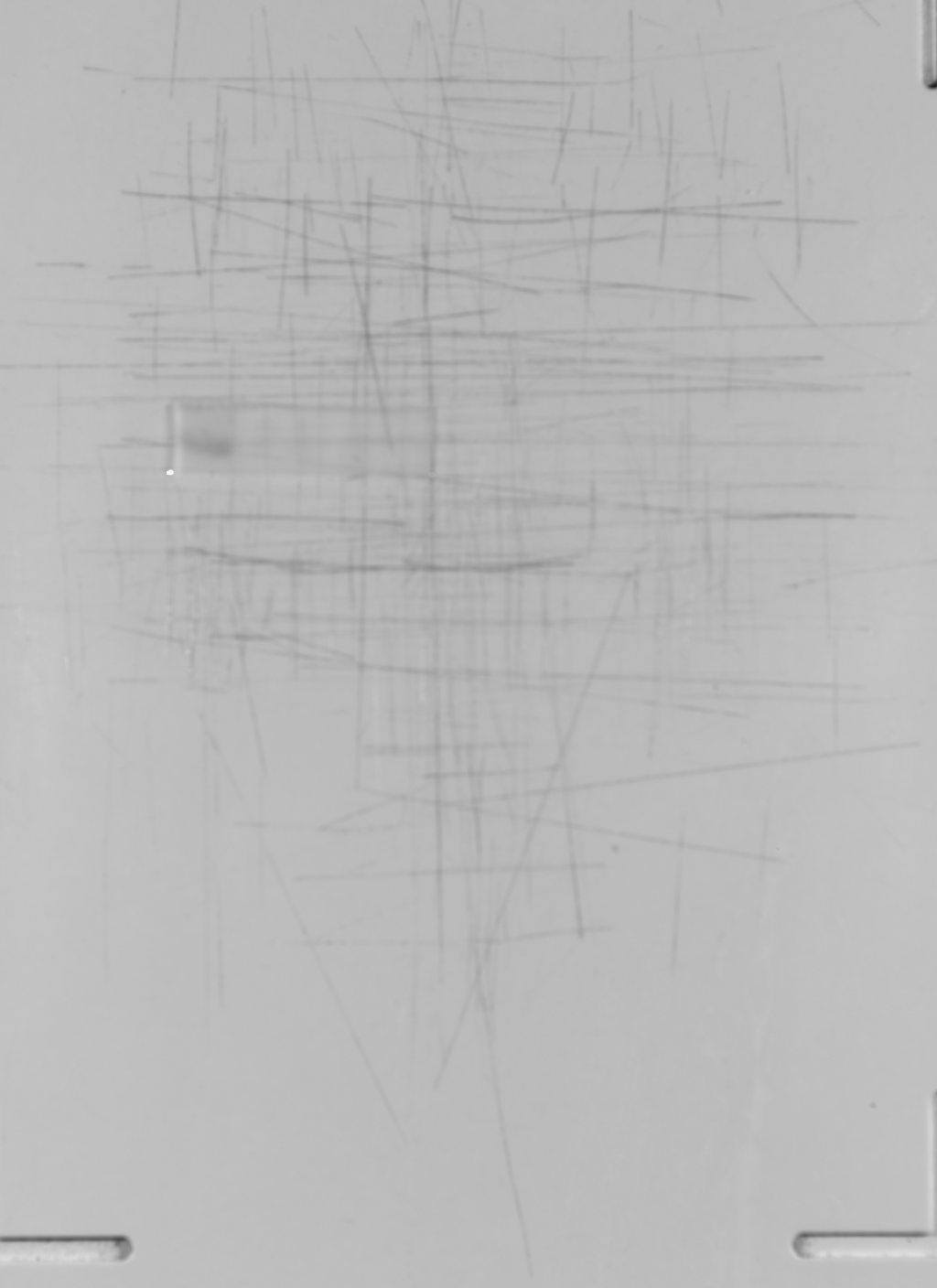

Supplement: Supplemental Information 12 [file peerj-11-15041-s012.zip › Transcriptome-related genes-raw data5/LY6C2/LY6C2-2/LY6C2-2-4.tif]

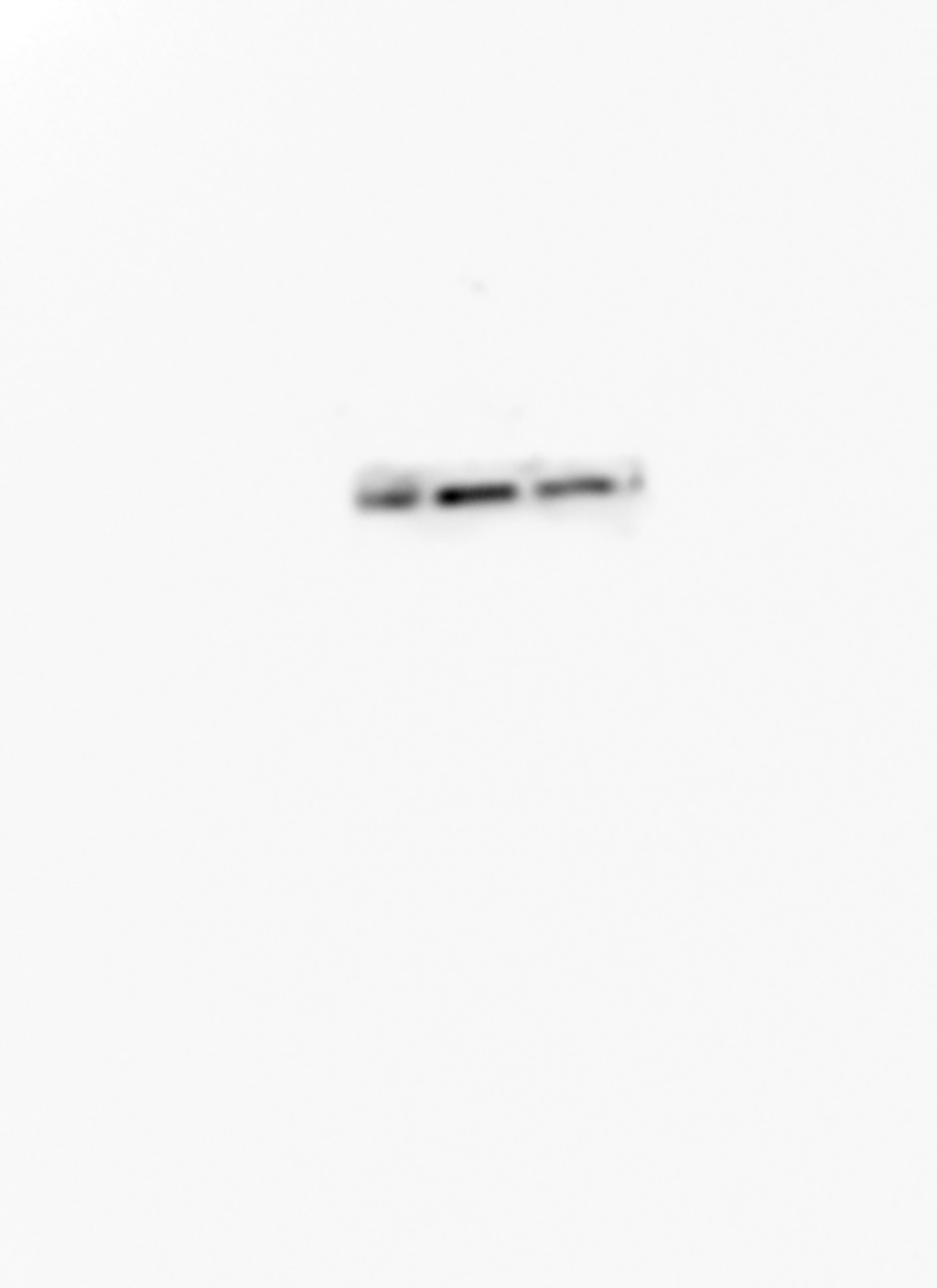

Supplement: Supplemental Information 12 [file peerj-11-15041-s012.zip › Transcriptome-related genes-raw data5/LY6C2/LY6C2-3/LY6C2-3-1.tif]

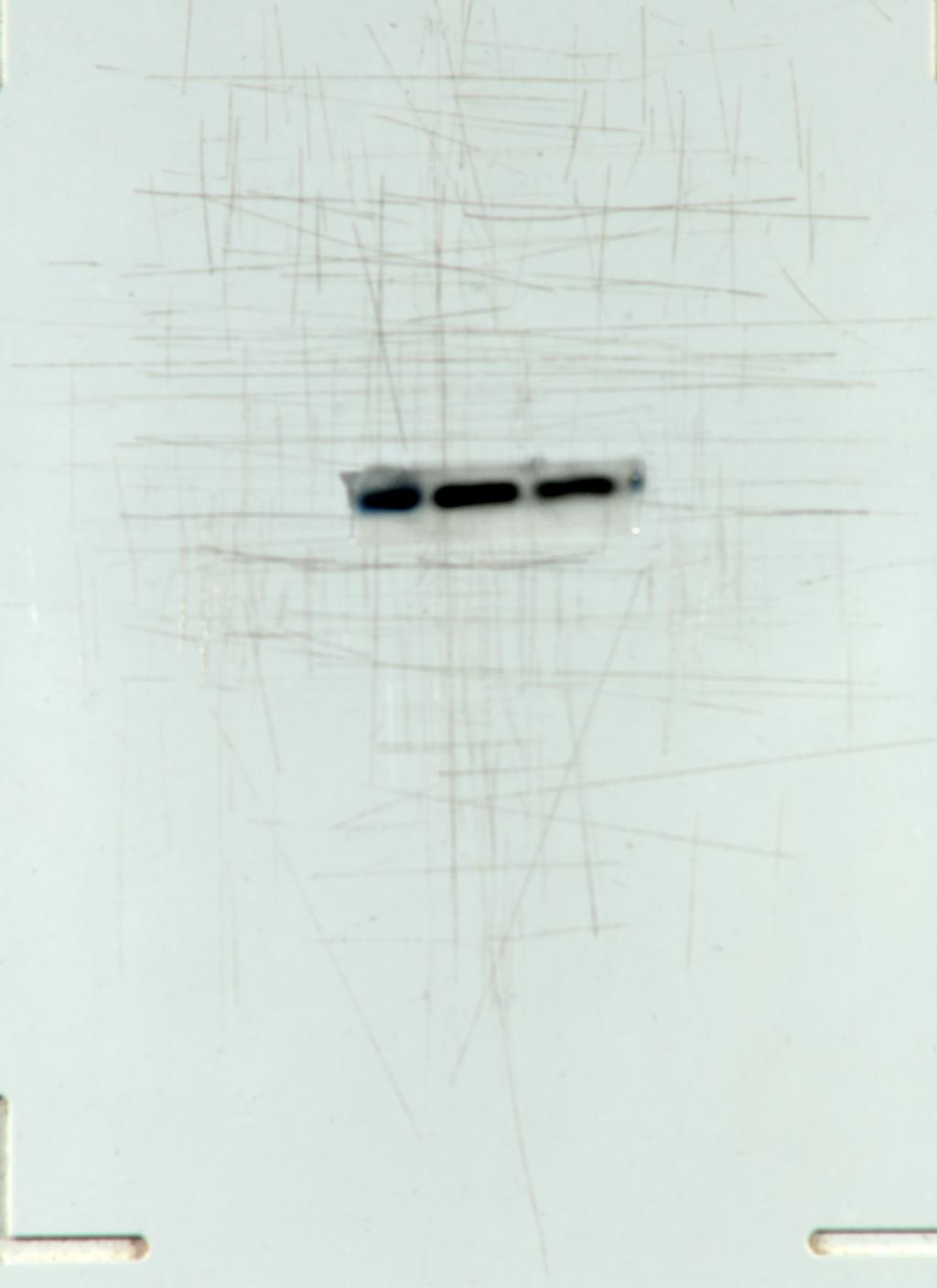

Supplement: Supplemental Information 12 [file peerj-11-15041-s012.zip › Transcriptome-related genes-raw data5/LY6C2/LY6C2-3/LY6C2-3-2.jpg]

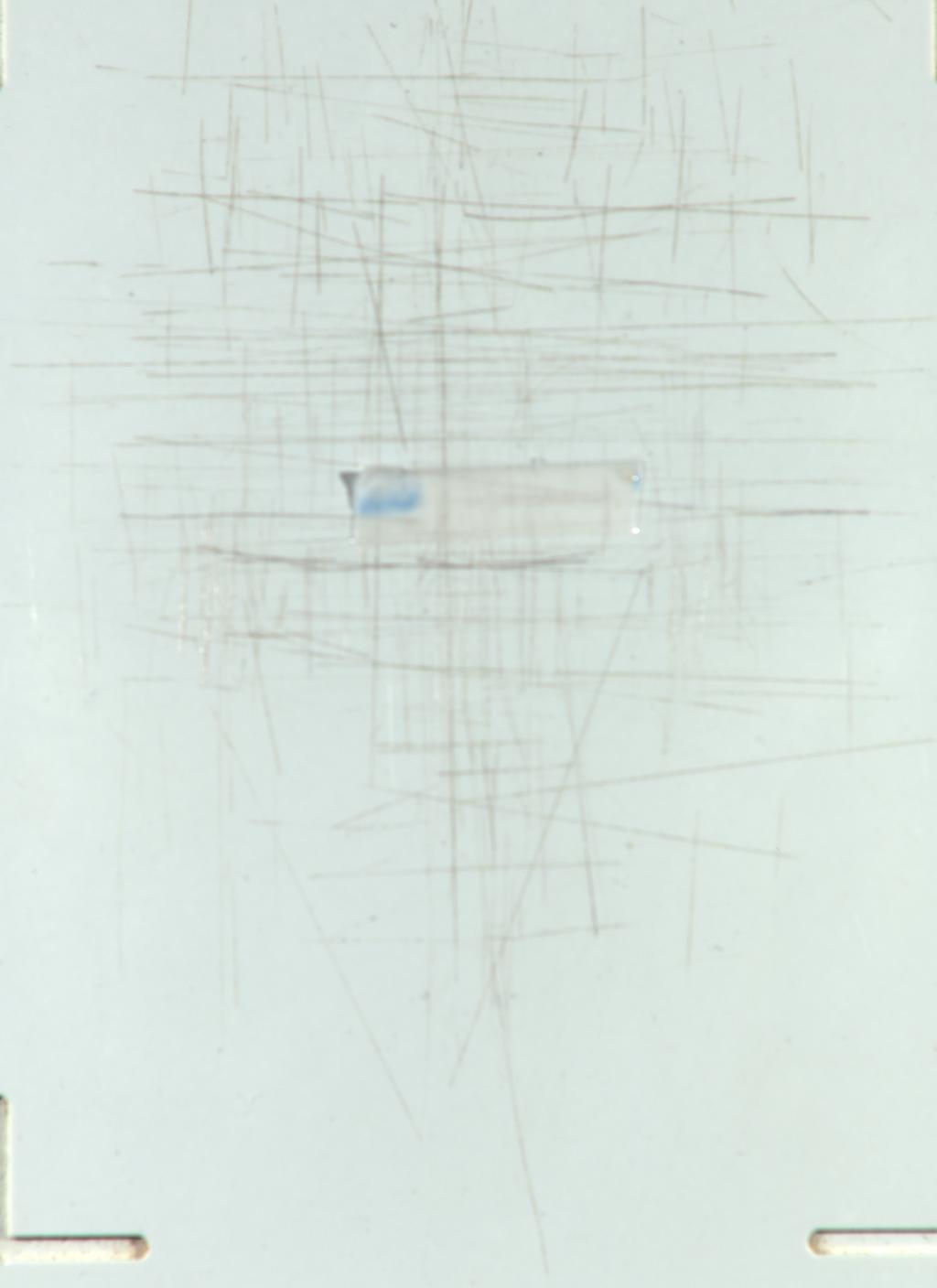

Supplement: Supplemental Information 12 [file peerj-11-15041-s012.zip › Transcriptome-related genes-raw data5/LY6C2/LY6C2-3/LY6C2-3-3.jpg]

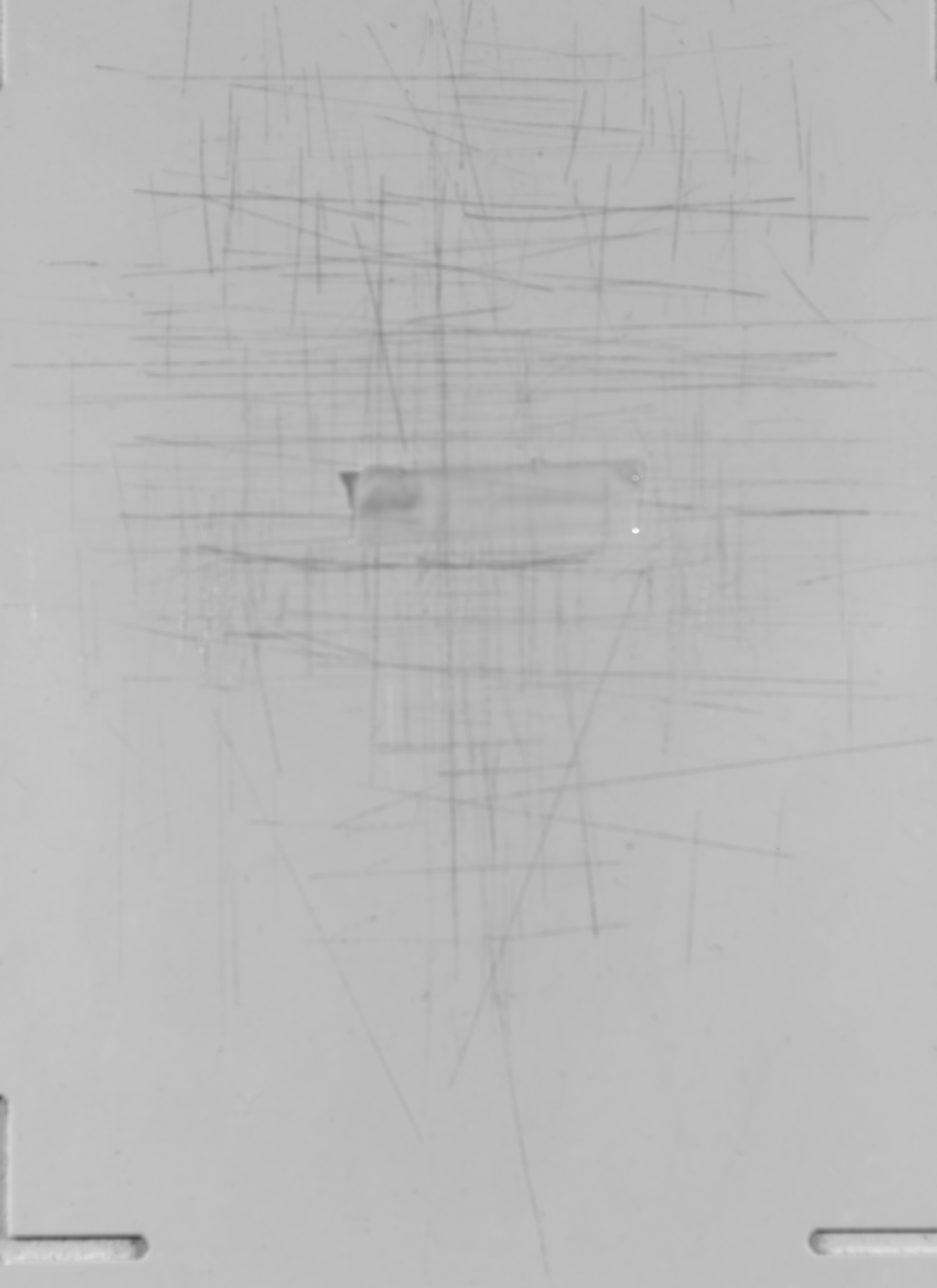

Supplement: Supplemental Information 12 [file peerj-11-15041-s012.zip › Transcriptome-related genes-raw data5/LY6C2/LY6C2-3/LY6C2-3-4.tif]
